# Germanium-integrated exciplex host for high-performance narrowband OLEDs with mitigated efficiency roll-off

**DOI:** 10.1038/s41467-026-76914-5

**Published:** 2026-08-20

**Authors:** Xu Zhang, Zhanxiang Chen, Manli Huang, Jiahui Liu, Siyuan He, Cheng Zhong, Jingsheng Miao, Xue Zhou, Yu Gu, Chuluo Yang

**Affiliations:** 1 Shenzhen Key Laboratory of New Display and Storage Materials, College of Materials Science and Engineering, Shenzhen University, Shenzhen, P. R. China; 2 Hubei Key Lab on Organic and Polymeric Optoelectronic Materials, Department of Chemistry, Wuhan University, Wuhan, P. R. China; 3 Wuhan China Star Optoelectronics Semiconductor Display Technology Co. Ltd, Wuhan, P. R. China

**Keywords:** Organic LEDs, Lasers, LEDs and light sources

## Abstract

Multi-resonance thermally activated delayed fluorescence (MR-TADF) materials open a heavy-metal-free avenue toward highly efficient, narrowband organic light-emitting diodes (OLEDs). However, their slow reverse intersystem crossing (RISC) kinetics lead to severe efficiency roll-off at practical driving currents, and existing strategies to improve RISC often require emitter-specific chemical modifications. Here we demonstrate a general strategy by integrating heavy-atom germanium into hole-transporting/electron-transporting hosts to construct an exciplex system that accelerates spin-flip dynamics. This design enhances RISC rates in diverse MR-TADF emitters via Förster resonance energy transfer without altering their electronic structure. The resulting blue and green OLEDs achieve external quantum efficiencies exceeding 40% with markedly suppressed efficiency roll-off relative to silicon- and carbon-based counterparts. Notably, the green device exhibits a half-lifetime of 241 h at an initial luminance of 1000 cd m^−2^. This general host-guest strategy advances MR-TADF systems toward practical high-performance displays.

## Abstract (web summary)

Efficiency roll-off in OLEDs is a challenge with multi-resonance thermally activated delayed fluorescence (MR-TADF) materials. Here, the authors add heavy-atom germanium into exciplex hosts to improve reverse intersystem crossing rates in diverse MR-TADF emitters without electronic structure changes.

## Introduction

Organic light-emitting diodes (OLEDs) combining high efficiency and narrowband emission are critical for meeting the growing demand for high-quality displays^1–3^. Multi-resonance thermally activated delayed fluorescence (MR-TADF) emitters, which enable efficient exciton utilization and narrowband emission, have emerged as promising alternatives to phosphorescent emitters reliant on heavy metals^4,5^. Pioneering work by Hatakeyama et al. demonstrated that MR-TADF emitters minimize structural reorganization and reduce the singlet-triplet energy gap through short-range charge-transfer transitions, achieving high device efficiency and narrow emission in OLEDs^6,7^. However, most high-performance MR-TADF materials still suffer from limited reverse intersystem crossing (RISC) rates, leading to severe efficiency roll-off at elevated driving currents^8,9^.

Addressing efficiency roll-off in OLEDs requires accelerating exciton consumption, particularly of dark triplet excitons, to suppress detrimental exciton-exciton and exciton-polaron interactions^10–15^. In MR-TADF systems, introducing heavy atoms into the molecular framework^16–18^ or periphery^19–21^ can enhance spin-orbit coupling, thereby accelerating the RISC process^22^. This approach has enabled RISC rates exceeding 10^6^ s^−1^, with reported green-emitting devices achieving a state-of-the-art external quantum efficiency (EQE) of 36.8% alongside reduced roll-off^16^. Despite these significant advances, heavy atom-modified emitters remain limited in their adaptability, typically being used on a case-by-case. We conceive a general strategy by integrating the heavy atom into the host molecular structure, which may apply to a broad range of emitters. Recent research has also focused on exciplex host systems formed by combining hole-transport (HT) and electron-transport (ET) materials^23–25^. These hosts improve charge balance in devices and, when paired with TADF sensitizers and terminal fluorescent emitters, enable high-efficiency OLEDs with suppressed efficiency roll-off. However, such systems typically require simultaneous co-evaporation of four independent components, complicating fabrication processes and demanding stringent control over deposition equipment. In this context, we are driven to develop an exciplex host with accelerated RISC that maintains balanced exciton transport and enables direct energy transfer to the terminal emitter without intermediate sensitization. This not only reduces the triplet population of MR-TADF emitters without requiring TADF sensitizers, but also delivers exceptional electroluminescent (EL) performance. Both blue and green OLEDs employing these hosts demonstrate high external quantum efficiencies over 40% alongside significantly reduced efficiency roll-off, showcasing their potential for simplified, high-performance device architectures^26,27^.

## Results

### Molecular design and photophysical properties

**Fig. 1:**
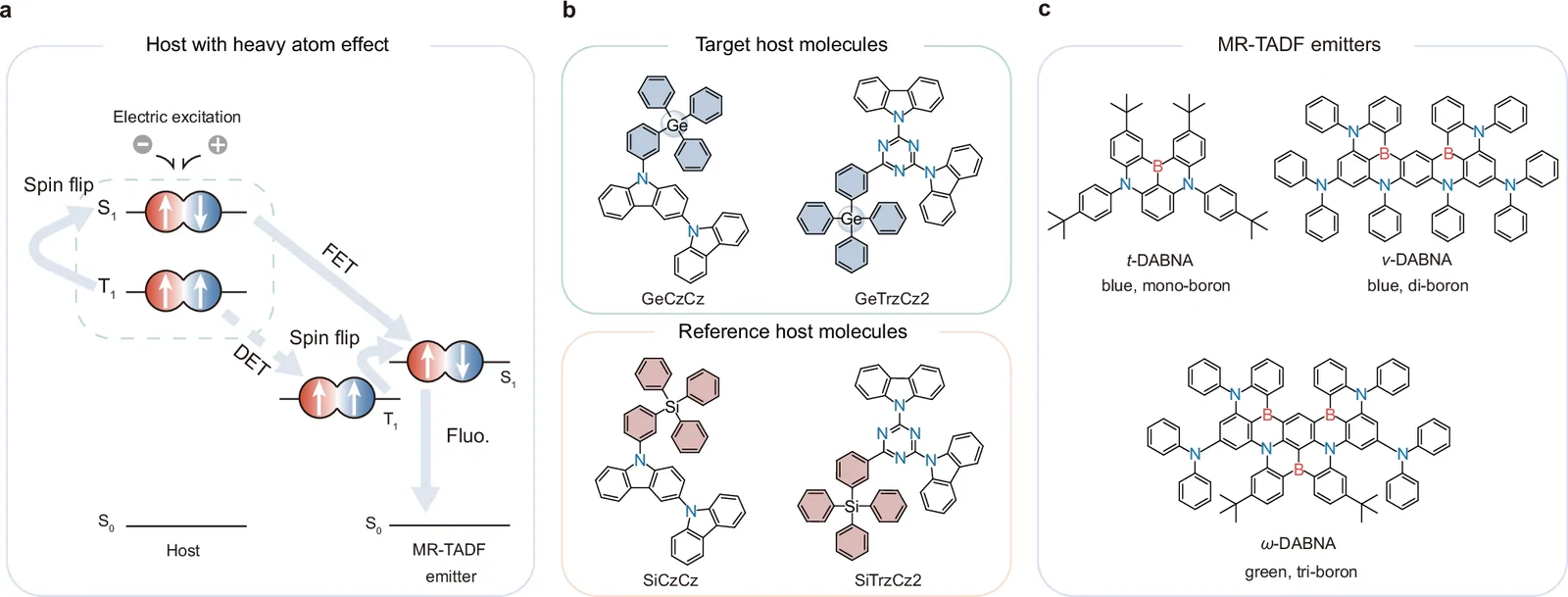
Host-guest system design. **a** Conceptual illustration of reverse intersystem crossing (RISC), Förster-type energy transfer (FET) and Dexter-type energy transfer (DET) processes in electroluminescence devices. S_0_, S_1_, and T_1_ denote the ground state, lowest singlet excited state, and lowest triplet excited state, respectively. Fluo. represents fluorescence emission. **b** Chemical structures of the target germanium-based hosts (GeCzCz, GeTrzCz2) and reference silicon-based hosts (SiCzCz, SiTrzCz2). **c** Chemical structures of the MR-TADF emitters used in this study.

The host-guest system developed in this work is illustrated in Fig. 1a. Our design leverages a host with a heavy atom effect to enable rapid triplet exciton upconversion and subsequent energy transfer to the MR-TADF guest (see Fig. 1b, c for the chemical structures). Building on previously reported hosts with bulky triphenylsilyl units^28^, specifically SiCzCz (9-(3-(triphenylsilyl)phenyl)-9*H*-3,9’-bicarbazole) and SiTrzCz2 (9,9’-(6-(3-(triphenylsilyl)phenyl)-1,3,5-triazine-2,4-diyl)bis(9*H*-carbazole)), we replaced silicon (atomic number *Z* = 14) with germanium (*Z* = 32) to boost spin-orbit coupling in the resulting exciplex. This substitution also minimally affected emission color, triplet energy (T_1_), and geometric configuration of the HT/ET host pair (as detailed below).

With the established design strategy in place, we synthesized 9-(3-(triphenylgermyl)phenyl)-9*H*-3,9’-bicarbazole (GeCzCz) via the Buchwald-Hartwig coupling reaction and 9,9’-(6-(3-(triphenylgermyl)phenyl)-1,3,5-triazine-2,4-diyl)bis(9*H*-carbazole) (GeTrzCz2) via the Suzuki-Miyaura cross-coupling reaction (Supplementary Note 1 and Supplementary Figs. 1–12). Single-crystal X-ray diffraction analysis (Supplementary Figs. 13 and 14) revealed that the C–Ge bond length (~1.95 Å) is slightly longer than the C–Si bond (~1.88 Å), and that GeCzCz and GeTrzCz2 exhibit increased crystal densities, suggesting germanium incorporation promotes closer molecular packing through stronger intermolecular dispersion interactions (Supplementary Table 1), which may in turn help to suppress the non-radiative decay. In both GeCzCz and its silicon analogue (SiCzCz), no significant intra- or intermolecular π-π interactions were observed; instead, both structures displayed multiple intermolecular C–H···π interactions between phenyl rings of the triphenylgermyl (or triphenylsilyl) unit and hydrogens on adjacent phenyl rings, as well as between carbazole rings and hydrogens on phenyl rings of the triphenylgermyl (or triphenylsilyl) group. Similarly, the GeTrzCz2 crystal structure exhibited intermolecular interactions comparable to those of SiTrzCz2, including π–π stacking between triazine and carbazole rings, as well as between adjacent carbazole rings. These structural similarities between germanium- and silicon-based analogues enable direct investigation of heavy-atom effects on excited states, while minimizing confounding factors such as variations in triplet-state energy or charge transport properties.

**Fig. 2:**
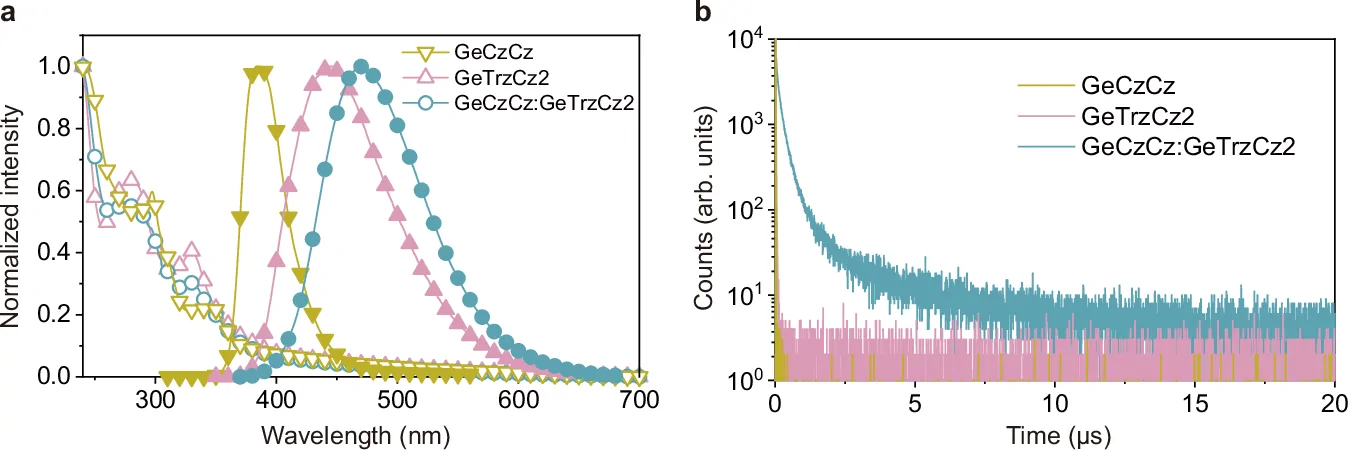
PL properties of germanium-based hosts. **a** UV-visible absorption spectra (open symbols) and PL spectra (filled symbols) of GeCzCz, GeTrzCz2, and GeCzCz:GeTrzCz2 in solid films. **b** Transient PL decay curves of neat GeCzCz, GeTrzCz2, and the GeCzCz:GeTrzCz2 exciplex films.

The steady-state photophysical properties of GeCzCz, GeTrzCz2, and their 1:1 molar blend (GeCzCz:GeTrzCz2) in solid films were further analyzed. As shown in Fig. 2a, both GeCzCz and GeTrzCz2 exhibited absorption bands below 350 nm, with emission maxima at 386 and 446 nm, respectively. The red-shifted and featureless photoluminescence (PL) spectrum of GeCzCz:GeTrzCz2 compared to those of the neat films (GeCzCz and GeTrzCz2) indicates the formation of an exciplex, with a maximum emission wavelength at 470 nm, consistent with the previously reported SiCzCz:SiTrzCz2 system. Phosphorescence spectra at 77 K (Supplementary Fig. 15) revealed T_1_ energies of 2.94 eV for GeCzCz and 2.91 eV for GeTrzCz2, both nearly equivalent to those of the SiCzCz and SiTrzCz2 reference, supporting sufficient T_1_ energy levels to suppress back energy transfer from dopant to host. Theoretical calculations of GeCzCz and GeTrzCz2 (Supplementary Fig. 16) are in good agreement with experimental data, yielding singlet (S_1_) energies of 3.85 eV and 3.41 eV, T_1_ energies of 2.88 eV and 2.80 eV, and oscillator strengths of 0.0525 and 0.0678 for GeCzCz and GeTrzCz2, respectively. The calculated reorganization energies, which characterize the structural relaxation during electronic transitions, are 0.43 eV for GeCzCz and 0.93 eV for GeTrzCz2. As expected, the delayed fluorescence lifetime of GeCzCz:GeTrzCz2 was significantly longer than those of the GeCzCz and GeTrzCz2 neat films, confirming efficient RISC in the exciplex (Fig. 2b and Supplementary Fig. 17). Notably, replacing silicon with germanium reduced the delayed fluorescence lifetime from 1.9 μs (SiCzCz:SiTrzCz2) to 1.6 μs (GeCzCz:GeTrzCz2) and the prompt fluorescence lifetime from 186.8 ns to 173.0 ns (Supplementary Fig. 18 and Supplementary Table 2), demonstrating that the heavy atom effect of germanium accelerates both prompt and delayed fluorescence processes. Key kinetic constants, including singlet radiative decay rate (*k*_r,S_), singlet non-radiative decay rate (*k*_nr,S_) and RISC rate (*k*_RISC_), were determined (Supplementary Note 2 and Supplementary Table 2)^29–31^. Compared to the silicon-based reference (SiCzCz:SiTrzCz2), the germanium-based exciplex exhibited a 1.9-fold enhanced *k*_r,S_ of 2.5 × 10^6^ s^−1^ and a 1.5-fold reduced *k*_nr,S_ of 2.1 × 10^6^ s^−1^, which together led to a 1.8-fold higher PL quantum yield (54% vs. 30%). The reduced *k*_nr,S_ is likely driven by a synergistic combination of closer intermolecular packing and suppressed intramolecular vibrational coupling in GeTrzCz2 compared to SiTrzCz2 (Supplementary Fig. 19 and Supplementary Table 3). Importantly, the *k*_RISC_ of GeCzCz:GeTrzCz2 also increased 1.3-fold to 8.0 × 10^5^ s^−1^ compared to the silicon counterpart (SiCzCz:SiTrzCz2), highlighting the role of germanium in promoting the RISC process of exciplex.

Additionally, density functional theory (DFT) calculations^31^ revealed that the highest occupied molecular orbital (HOMO) of GeCzCz:GeTrzCz2 is localized on the bicarbazole units of GeCzCz, while the lowest unoccupied molecular orbital (LUMO) resides on the triazine moiety of GeTrzCz2 (Supplementary Fig. 20). This indicates that the spatial separation between HOMO and LUMO remains unaffected by the substitution of the heavy-atom germanium. Analysis of spin-orbit coupling matrix elements (SOCMEs) in the optimized T_1_ geometry of the lowest-energy conformer showed an approximately threefold increase in <S_1_|*Ĥ*_SOC_|T_1_> from 0.265 cm^−1^ (SiCzCz:SiTrzCz2) to 0.791 cm^−1^ (GeCzCz:GeTrzCz2), consistent with the expected enhancement in spin-orbit coupling due to the higher atomic number (Supplementary Fig. 21). Similar trends are observed for other conformers (Supplementary Fig. 22) and for the upper excited state (T_2_); for example, <S_1_|*Ĥ*_SOC_|T_2_> reaches 0.174 cm^−1^ for GeCzCz:GeTrzCz2, compared to 0.080 cm^−1^ for SiCzCz:SiTrzCz2. These results confirm accelerated spin-flip processes in the germanium-based exciplex, aligning with the time-resolved photoluminescence data.

**Fig. 3:**
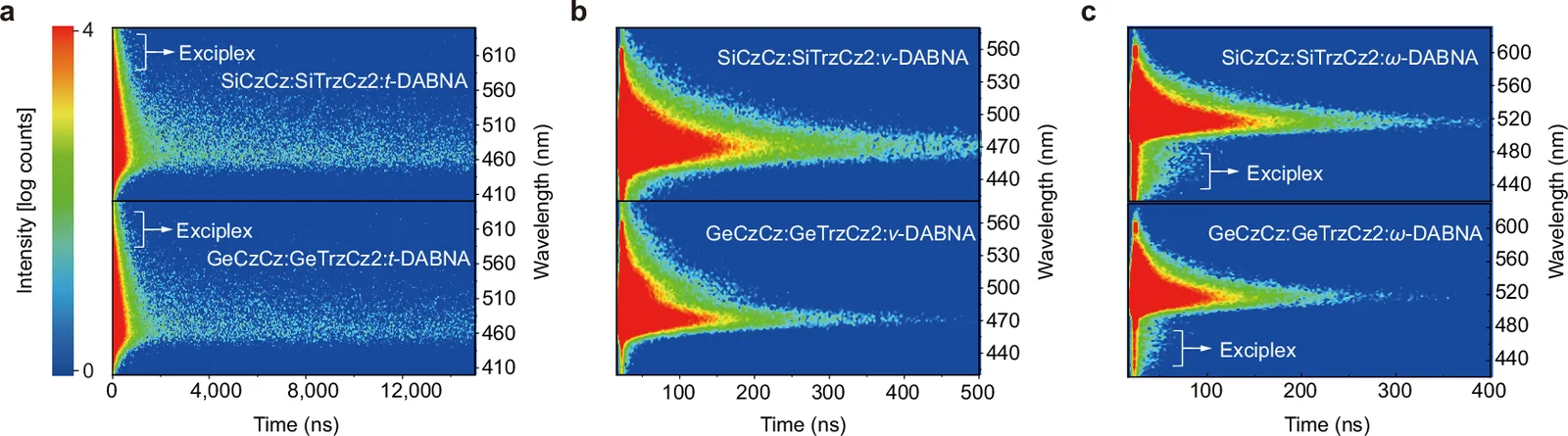
Transient emission properties of MR-TADF guest-doped films. **a** Time-dependent emission contours of SiCzCz:SiTrzCz2:*t*-DABNA and GeCzCz:GeTrzCz2:*t*-DABNA films, measured in the range of 400–650 nm. **b** Time-dependent emission contours of SiCzCz:SiTrzCz2:*υ*-DABNA and GeCzCz:GeTrzCz2:*υ*-DABNA films, measured in the range of 420–580 nm. **c** Time-dependent emission contours of SiCzCz:SiTrzCz2:*ω*-DABNA and GeCzCz:GeTrzCz2:*ω*-DABNA films, measured in the range of 420–630 nm.

The photophysical properties of GeCzCz:GeTrzCz2 as the host were further analyzed and compared to the reference SiCzCz:SiTrzCz2 host. We selected two blue MR-TADF emitters^1,32^ (*t*-DABNA, single-boron; *υ*-DABNA: double-boron) and a green emitter^33^ (*ω*-DABNA, triple-boron), doping all at 1 wt% to minimize interchromophore quenching. Absorption spectra of the emitters overlapped with both hosts (Supplementary Fig. 23), ensuring efficient Förster resonance energy transfer (FRET). Förster radius (*R*_FRET_) for *t*-DABNA, *υ*-DABNA, and *ω*-DABNA were calculated as 4.7 nm, 7.6 nm, and 8.5 nm, respectively, based on spectral overlap between the extinction spectrum of the emitter and PL spectra of the host. Crucially, in the GeCzCz:GeTrzCz2 host, the rate of Dexter energy transfer (*k*_DET_) for *t*-DABNA, *υ*-DABNA and *ω*-DABNA was reduced by factors of 1.33, 1.70 and 3.05, respectively, compared with SiCzCz:SiTrzCz2 host (Supplementary Fig. 24, Supplementary Table 4 and Supplementary Note 3), supporting the fact that the germanium-based exciplex host suppresses Dexter energy transfer. Time-resolved emission spectra (Fig. 3) revealed that the germanium-based exciplex host reduced delayed fluorescence lifetimes of *t*-DABNA, *υ*-DABNA, and *ω*-DABNA by 1.18-fold, 1.67-fold, and 2.14-fold, respectively, compared to the silicon-based exciplex host. Greater lifetime reductions for *υ*-DABNA and *ω*-DABNA correlated with their larger *R*_FRET_, which ensured more complete FRET. Concurrently, the *k*_RISC_ for *t*-DABNA, *υ-DABNA* and *ω*-DABNA increased by 1.69-fold, 2.02-fold and 2.08-fold in the germanium-based exciplex host, with *υ*-DABNA achieving *k*_RISC_ = 9.1 × 10^5^ s^−1^ (Supplementary Table 4). These improvements, driven by enhanced SOCME of germanium-based exciplex host, demonstrate the host’s efficacy in accelerating RISC dynamics.

### OLED devices

**Fig. 4:**
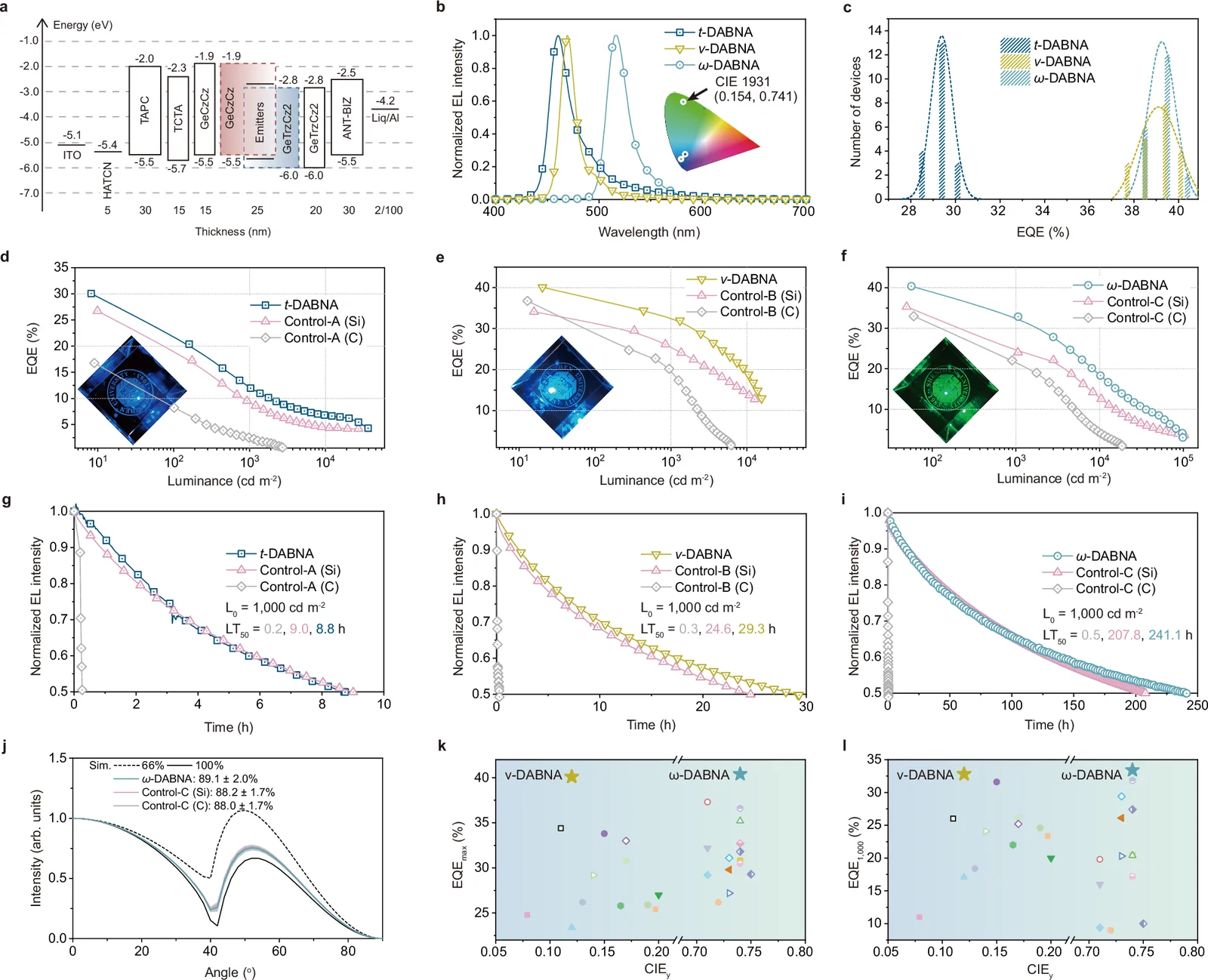
Optimized EL properties of OLEDs. **a** Diagram of the germanium-based device structure. **b** Electroluminescence (EL) spectra recorded at 1000 cd^.^m^−2^. **c** Statistical histogram of external quantum efficiencies (EQEs) for different devices. **d–f** EQE versus luminance characteristics of target devices (*t*-DABNA (**d**), *υ*-DABNA (**e**), *ω*-DABNA (**f**)) and control devices (control-A (**d**), control-B (**e**), control-C (**f**)). **g** Half-lifetime (LT_50_) measurement of *t*-DABNA-based device and control devices (control-A) at an initial luminance (L_0_) of 1000 cd^.^m^−2^. **h** LT_50_ measurement of *υ*-DABNA-based device and control devices (control-B) at an L_0_ of 1000 cd^.^m^-2^. **i** LT_50_ measurement of *ω*-DABNA-based device and control devices (control-C) at an L_0_ of 1000 cd^.^m^-2^. **j** Angle-dependent PL spectra of *ω*-DABNA-doped films. *Θ*_//_, horizontal emitting dipole orientation. **k**, **l** Summary of CIEy coordinates versus maximum EQE (**k**) values and EQE at 1000 cd^.^m^−2^ (**l**) for state-of-the-art blue (CIEy ≤ 0.20) and green (CIEy ≥ 0.71) OLEDs.

Motivated by the shortened delayed fluorescence lifetimes and enhanced *k*_RISC_ of MR-TADF emitters in the germanium-based exciplex host, we evaluated their EL performance in optimized OLEDs. Target devices were fabricated with the structure: indium tin oxide (ITO)/1,4,5,8,9,11-hexaazatriphenylenehexacarbonitrile (HAT-CN, 5 nm)/1,1-bis[(di-4-tolylamino)phenyl]cyclohexane (TAPC, 30 nm)/tris(4-carbazolyl-9-ylphenyl)amine (TCTA, 15 nm)/GeCzCz (15 nm)/GeCzCz:GeTrzCz2:dopant (25 nm, 0.50:0.49:0.01 w/w/w)/ GeTrzCz2 (20 nm)/1-(4-(10-([1,1’-biphenyl]-4-yl)anthracen-9-yl)phenyl)-2-ethyl-1*H*-benzo[*d*]imidazole (ANT-BIZ, 30 nm)/8-hydroxyquinolinato lithium (Liq, 2 nm)/alumina (Al, 100 nm), where the emissive layer incorporated *t*-DABNA, *υ*-DABNA, or *ω*-DABNA as dopant. From single-carrier current-voltage measurements (Supplementary Fig. 25), it is demonstrated that the GeCzCz:GeTrzCz2 (0.50/0.50 w/w) host provides efficient and relatively more balanced charge transport, with a hole mobility of 3.07 × 10^−5^ cm^2.^V^−1.^s^−1^ and an electron mobility of 5.69 × 10^−5^ cm^2.^V^−1.^s^−1^. Control devices (A-C) were fabricated by substituting GeCzCz and GeTrzCz2 with their silicon-based (SiCzCz and SiTrzCz2) or carbon-based counterparts (CCzCz and CTrzCz2, Supplementary Figs. 26–34). The chemical structures of the organic materials, energy-level diagram and EL performance are depicted in Fig. 4 and Supplementary Fig. 35, with detailed parameters provided in Table 1.

**Table 1:** Key data of the devices

Device	CE (cd^.^A^−1^)		PE (lm W^−1^)		EQE (%)		
	**Max (average)**	**1000 cd m**^**−2**^	**Max (average)**	**1000 cd m**^**−2**^	**Max (average)**	**1000 cd m**^**−2**^	**5000 cd m**^**−2**^
*t*-DABNA	38.1 (35.6 ± 1.3)	14.4	42.7 (39.9 ± 1.4)	10.6	30.1 (29.3 ± 0.5)	12.0	7.6
Control-A (Si)	38.7 (37.1 ± 1.9)	10.2	43.5 (41.6 ± 2.2)	6.0	26.9 (26.5 ± 0.5)	7.5	4.5
Control-A (C)	22.6 (20.6 ± 2.3)	3.3	25.4 (23.1 ± 2.5)	1.6	16.8 (15.9 ± 0.7)	2.5	-
*υ*-DABNA	43.6 (37.4 ± 1.6)	30.4	48.9 (38.8 ± 3.3)	27.7	40.1 (39.2 ± 0.9)	32.8	24.4
Control-B (Si)	35.8 (32.3 ± 2.9)	23.1	40.2 (35.2 ± 4.6)	18.5	34.1 (33.1 ± 1.1)	22.4	14.6
Control-B (C)	31.8 (29.3 ± 2.4)	17.1	35.7 (32.8 ± 2.7)	13.3	36.7 (34.4 ± 2.0)	19.7	3.2
*ω*-DABNA	145.5 (142.1 ± 2.3)	118.6	163.2 (159.4 ± 2.6)	117.8	40.4 (39.3 ± 0.7)	33.4	23.9
Control-C (Si)	130.7 (124.5 ± 5.0)	87.3	146.6 (139.7 ± 5.6)	79.4	35.3 (34.3 ± 0.8)	24.7	10.5
Control-C (C)	121.3 (117.1 ± 3.2)	78.8	136.1 (131.4 ± 3.6)	76.6	33.0 (31.7 ± 1.0)	21.7	9.9

The average device parameters in parentheses are based on the measurement of twenty independent devices.

Figure 4b illustrates the EL spectra of the three target OLEDs employing the GeCzCz:GeTrzCz2 host, labeled as *t*-DABNA-based, *υ*-DABNA-based, *ω*-DABNA-based devices. The *t*-DABNA-based device exhibited deep-blue emission peaking at 460 nm, while the *υ*-DABNA-based device displayed pure blue emission at 469 nm. Despite its bluer emission, the *t*-DABNA device achieved CIE coordinates of (0.154, 0.141), less optimal than the *υ*-DABNA device’s (0.125, 0.113), due to incomplete Förster energy transfer from the host to the *t*-DABNA dopant. Notably, the *ω*-DABNA-based device emitted green light with CIE coordinates (0.154, 0.741), closely approaching the Rec. 2020 green standard (0.170, 0.797).

All fabricated devices based on GeCzCz:GeTrzCz2 or SiCzCz:SiTrzCz2 as host exhibited nearly identical turn-on voltages of 2.8 V and high maximum luminance over 30,000 cd m^-2^ (Supplementary Fig. 36), indicating efficient exciton formation in the exciplex host followed by FRET to the guest (Langevin recombination). Target devices using the GeCzCz:GeTrzCz2 as host showed higher efficiencies and reduced efficiency roll-off compared to SiCzCz:SiTrzCz2-based and CCzCz:CTrzCz2-based ones (Control A-C). The *t*-DABNA-based device achieved a maximum EQE of 30.1% (Fig. 4d), surpassing Control-A (silicon, 26.9%; carbon, 16.8%). However, incomplete energy transfer from the GeCzCz:GeTrzCz2 host to the *t*-DABNA guest (Fig. 4b) limited its EQE to 12.0% at 1,000 cd m^-2^. In contrast, devices with complete host-to-guest energy transfer, specifically *υ*-DABNA and *ω*-DABNA-based devices, achieved peak EQEs of 40.1% and 40.4%, respectively. To confirm the origin of the high efficiencies, the angle-dependent PL intensities were characterized to study the emitting dipole orientation of these emitting layer films. As illustrated in Fig. 4j and Supplementary Fig. 37, they showed comparably high horizontal emitting dipole orientation up to 89.1 ± 2.0%. Optical simulations performed on the corresponding devices revealed that the theoretical maximum EQE varied only marginally across the three exciplex hosts (Supplementary Fig. 38). Notably, only the devices based on the GeCzCz:GeTrzCz2 host closely approached their theoretical EQE limits, confirming that their superior performance is driven by highly efficient triplet-to-singlet spin-flip process. Their EQE reproducibility, shown in a histogram (Fig. 4c), averaged 39.2 ± 0.9% (*υ*-DABNA) and 39.3 ± 0.7% (*ω*-DABNA). At 1,000 cd m^-2^, these devices retained EQE values of 32.8% (*υ*-DABNA) and 33.4% (*ω*-DABNA), corresponding to a 18.2% and 17.3% decrease in EQE, respectively. These roll-off characteristics outperformed those of Control-B (34.3% and 46.3% efficiency decreases for silicon-based and carbon-based devices, respectively) and Control-C (30.0% and 34.2%, respectively) at equivalent brightness levels. The suppressed roll-off in germanium-based devices stems from reduced bimolecular annihilation processes (e.g., triplet-triplet and triplet-polaron annihilation), enabled by accelerated spin-flip kinetics from enhanced SOCME via germanium’s heavy-atom effect. To the best of our knowledge, the *υ*-DABNA and *ω*-DABNA-based devices set record EQE values for OLEDs with CIEy ≤ 0.2 (blue) and CIEy ≥ 0.71 (green, NTSC standard), surpassing platinum-based phosphorescent and TADF-sensitized systems (Fig. 4k,l and Supplementary Table 5). Next, we assessed the operational stability of these devices at an initial luminance of 1000 cd^.^m^-2^, as shown in Fig. 4g-i. The CCzCz:CTrzCz2-based devices exhibited the poorest stability, with operational half-lifetimes (LT_50_) of 0.2-0.5 h. The SiCzCz:SiTrzCz2-based devices revealed improved stability, achieving LT_50_ values of 9.0 h, 24.6 h, and 207.8 h for *t*-DABNA, *υ*-DABNA, and *ω*-DABNA, respectively. The GeCzCz:GeTrzCz2-based devices demonstrated the best overall performance (LT_50_ values of 8.8 h, 29.3 h, and 241.1 h for *t*-DABNA, *υ*-DABNA, and *ω*-DABNA, respectively). Using *υ*-DABNA as a representative guest to probe the underlying exciton dynamics, transient electroluminescence measurements (Supplementary Fig. 39) revealed a significantly longer delayed lifetime for the carbon-based device (95.60 μs) than those of the silicon-based device (67.30 μs) and germanium-based device (34.20 μs). This prolonged delayed lifetime indicates a substantial accumulation of long-lived triplet excitons in the carbon-based emitting layer, which promotes destructive bimolecular annihilation to accelerate molecular degradation. This behavior is further supported by photoluminescence stability tests under continuous UV irradiation, where the carbon-based films consistently degraded the most rapidly (Supplementary Fig. 40). Meanwhile, consecutive cyclic voltammetry scans (Supplementary Fig. 41) revealed comparable electrochemical stability among these host materials, confirming that their electrochemical stability does not correlate with the observed device lifetime trend. Notably, when *t*-DABNA was employed as the emitter, the germanium-based and silicon-based devices showed comparable lifetimes, likely because the high emission energy of *t*-DABNA compromises the chemical stability of both exciplex hosts. In contrast, for guests with reduced emission energies (*υ*-DABNA and *ω*-DABNA), the GeCzCz:GeTrzCz2-based devices displayed longer lifetimes than the silicon-based control devices, due to the decreased triplet population associated with the germanium-based exciplex host. These results validate the generality of heavy-atom-modified exciplex hosts for achieving efficient and stable OLEDs without complex guest synthesis.

## Discussion

By integrating HT and ET hosts containing germanium into an exciplex host aligned with the low-energy S_1_ state of MR-TADF emitters, the intrinsically slow *k*_RISC_s of MR-TADF emitters were significantly accelerated. This principle was validated using blue (*υ*-DABNA) and green (*ω*-DABNA) MR-TADF emitters, which exhibited 2.02- and 2.08-fold enhancements in *k*_RISC_, reaching up to 9.1 × 10^5 ^s^-1^ under photoexcitation. Resulting blue and green OLEDs achieved maximum EQEs exceeding 40%, with significantly reduced roll-off and improved operational stability. Our strategy not only advances MR-TADF-based OLED performance but also provides a universal approach to minimize efficiency roll-off without complex emitter synthesis, extendable to applications beyond optoelectronics.

## Methods

### General information

All commercially available reagents were used as received. GeTrzCz2, GeCzCz, CTrzCz2 and CCzCz were synthesized as detailed in the Supplementary Information. High-resolution mass spectrometry was performed on a Thermo Scientific LTQ Orbitrap XL equipped with an electrospray ionization source. NMR spectra were measured on Bruker Advance 400 or 500 MHz spectrometers using tetramethylsilane as an internal standard and CDCl_3_ as the solvent. Organic films for optical characterization were deposited onto clean quartz substrates by thermal evaporation under high vacuum. UV-vis absorption spectra were recorded on a Shimadzu UV-2600 spectrophotometer. Steady-state fluorescence and phosphorescence spectra were collected using a Hitachi F-7100 fluorescence spectrophotometer. Time-resolved fluorescence lifetimes were measured with a PicoQuant FluoTime 300 system employing a 375 nm picosecond pulsed UV laser. Absolute PL quantum yields were determined using a Hamamatsu C13534 UV-NIR spectrometer with an integrating sphere purged with dry argon; samples were excited at 320 nm. The orientation of electric transition dipole in the doped films was determined by angle-resolved and polarization-resolved PL measurements. A doped film with a thickness of 15 nm was deposited onto a fused-silica-based half-cylindrical lens and excited at a fixed angle of 45° using a continuous-wave He:Cd laser (375 nm). The *p*-polarized emission light was detected at the PL peak wavelength of the dopants.

### Theoretical calculations

All DFT calculations were carried out using the Gaussian 16 software package^34^. Geometry optimizations were performed using the B3LYP functional with the DFT-D3(BJ) empirical dispersion correction^35^. The 6-31 G(d) basis set was employed for hydrogen, carbon, nitrogen, and silicon atoms^36^, while the Stuttgart-Dresden (SDD) pseudopotential and its associated valence basis set was applied for germanium (Ge)^37^. Time-dependent DFT (TD-DFT) calculations for excited states were conducted at the CAM-B3LYP functional level with the same basis sets^38^. To obtain the optimal bimolecular conformations of the donor-acceptor pairs in the exciplex systems, a systematic, multi-step conformational search was implemented. Initially, 5,000 donor-acceptor configurations were generated using the Genmer package and pre-optimized at the semiempirical GFN2-xTB levels using the Molclus program^39^. These candidate structures were further optimized at the B3LYP-D3(BJ)/6-31 G(d)/SDD(Ge) level in Gaussian 16. To eliminate structural redundancy, near-duplicate conformations were filtered out by applying a spatial coordinate threshold of 3.0 Å, yielding 16 distinct bimolecular candidates. The geometries of the lowest triplet states (T_1_) were subsequently optimized utilizing the Tamm-Dancoff approximation (TDA) to TD-DFT at the CAM-B3LYP-D3(BJ)/6-31 G(d)/SDD(Ge) level. Subsequently, spin-orbit coupling (SOC) matrix elements were computed using the PySOC package interfaced with Gaussian 16 at the B3LYP functional level^40^. These matrix elements were evaluated from the TD-DFT wavefunctions using the Breit-Pauli spin-orbit Hamiltonian in conjunction with the effective nuclear charge (*Z*_*eff*_) approximation via the MolSOC module. To isolate and quantify the SOC contribution of the specific target atom, we implemented a *Z*_eff_ masking approach. By setting the *Z*_eff_ of all other atoms in the input to zero (*Z*_eff_ = 0), the resulting SOCME reflects only the localized contribution originating from the selected target atom.

### Analyses of Förster radius

The rate of Förster resonance energy transfer (*k*_FRET_) can be expressed as:

1 $${k}_{{{{\rm{FRET}}}}}={k}_{r,{{{\rm{D}}}}}\frac{{k}^{2}}{{R}^{6}}\left(\frac{900({{\mathrm{ln}}}10)}{128{\pi }^{5}{N}_{A}}\right){\int }_{\!\!\!\!\!0}^{\infty }\frac{{F}_{{{{\rm{D}}}}}(\lambda ){\varepsilon }_{{{{\rm{A}}}}}(\lambda ){\lambda }^{4}}{{[n(\lambda )]}^{4}}d\lambda .$$

where *R* is the intermolecular distance of the donor (GeCzCz:GeTrzCz2 exciplex host, SiCzCz:SiTrzCz2 exciplex host and CCzCz:CTrzCz2 exciplex host) and acceptor (*t*-DABNA, *υ*-DABNA, and *ω*-DABNA); *k*_*r*,D_ is the radiative decay rate constant of the donor (GeCzCz:GeTrzCz2 exciplex host, SiCzCz:SiTrzCz2 exciplex host and CCzCz:CTrzCz2 exciplex host) in absence of the acceptor (*t*-DABNA, *υ*-DABNA, and *ω*-DABNA); *κ*^2^ is a configurational factor describing the relative orientation of electric transition dipole moments of the donor (GeCzCz:GeTrzCz2 exciplex host, SiCzCz:SiTrzCz2 exciplex host and CCzCz:CTrzCz2 exciplex host) and acceptor (*t*-DABNA, *υ*-DABNA, and *ω*-DABNA) and is assumed to be 2/3 because, despite the highly aligned dipoles of the guest acceptors, the completely amorphous donor hosts statistically ensure an isotropic relative orientation; *N*_A_ is the Avogadro constant; *n*(*λ*) is the refractive index of the donor (Supplementary Fig. 42); *F*_D_(*λ*) is the normalized emission spectra of the donor (GeCzCz:GeTrzCz2 exciplex host, SiCzCz:SiTrzCz2 exciplex host and CCzCz:CTrzCz2 exciplex host); *ε*_A_(*λ*) is the molar absorption coefficient of the acceptor (*t*-DABNA, *υ*-DABNA, and *ω*-DABNA); and *λ* is the corresponding wavelength. The Förster radius (*R*_FRET_) is defined as an intermolecular distance at which the *k*_FRET_ is equal to the *k*_*r*,D_.

### Device fabrication and characterization

Prior to device fabrication, ITO glass substrates were thoroughly cleaned. The substrates were then transferred to a high-vacuum deposition chamber (pressure ≤ 5.0×10^-5 ^Pa) for sequential layer deposition. Organic layers were thermally evaporated at 1.0 Å^·^s^-1^, followed by 2 nm of Liq and 100 nm aluminum cathodes. All organic materials were purified via vacuum sublimation. The electroluminescence characteristics were measured using a Keithley 2400 DC source meter and a Hamamatsu C9920-12 EQE measurement system. Operational stability of encapsulated devices was evaluated under constant current density (initial luminance: 1000 cd^.^m^-2^) using an FS-MP64 luminance meter (Suzhou FSTAR). Optical simulations of OLED devices were performed with SETFOS 5.1 (Fluxim) program, using the refractive index, extinction coefficient, and thickness of each layer (obtained by ellipsometry) together with the photoluminescence spectrum of the emitting layer as input parameters.

## Supplementary information

Supplementary Information

Description of Additional Supplementary Information

Supplementary Data 1

Supplementary Data 2

Supplementary Data 3

Supplementary Data 4

Transparent Peer Review file

## Source data

Source data

## Data Availability

Crystallographic data for the structures reported in this article have been deposited at the Cambridge Crystallographic Data Center (CCDC), under deposition numbers CCDC 2433937 (SiCzCz), 2433938 (GeCzCz), 2433936 (SiTrzCz2) and 2433939 (GeTrzCz2). Copies of the data can be obtained free of charge via https://www.ccdc.cam.ac.uk/structures/. The full set of data generated in this study has been deposited in the Figshare database under accession code https://doi.org/10.6084/m9.figshare.28741448. Source data are provided with this paper.
