# Supplementary material for: Germanium-integrated exciplex host for high-performance narrowband OLEDs with mitigated efficiency roll-off
Source: Nat Commun. 2026 Aug 20;17:9942. doi: 10.1038/s41467-026-76914-5 (PMC13586258; doi:10.1038/s41467-026-76914-5)
Supplement: Supplementary file 1 [file 41467_2026_76914_MOESM1_ESM.pdf]

Supplementary Information

Germanium-integrated exciplex host for high-performance narrowband OLEDs with mitigated efficiency roll-off

Xu Zhang[1†], Zhanxiang Chen[1†*], Manli Huang[1], Jiahui Liu[1], Siyuan He[1], Cheng Zhong[2], Jingsheng Miao[1], Xue Zhou[3], Yu Gu[3], Chuluo Yang[1*]

[1]Shenzhen Key Laboratory of New Display and Storage Materials, College of Materials Science and Engineering, Shenzhen University, Shenzhen, P. R. China

[2]Hubei Key Lab on Organic and Polymeric Optoelectronic Materials, Department of Chemistry, Wuhan University, Wuhan, P. R. China

[3]Wuhan China Star Optoelectronics Semiconductor Display Technology Co. Ltd., Wuhan, P. R. China

[†]These authors contributed equally: Xu Zhang, Zhanxiang Chen.

*Corresponding authors. Email: Zhanxiang Chen (zxchen@szu.edu.cn); Chuluo Yang (clyang@szu.edu.cn)

Table of Contents

Supplementary Notes

Supplementary Note 1. Materials synthesis and characterization

Supplementary Note 2. Calculation method of $k_{r,S}$, $k_{nr,S}$ and k_{RISC}.

Supplementary Note 3. Calculation method of k_{DET}

Supplementary Figures

Supplementary Fig. 1. Synthesis procedure of GeTrzCz2

Supplementary Fig. 2. Synthesis procedure of GeCzCz

Supplementary Fig. 3. Synthesis procedure of CTrzCz2

Supplementary Fig. 4. Synthesis procedure of CCzCz

Supplementary Fig. 5. ^{1}H NMR spectrum of triphenyl(3-(4,4,5,5-tetramethyl-1,3,2-dioxaborolan-2-yl)phenyl)germane in $CDCl_3$ at room temperature

Supplementary Fig. 6. ^{13}C NMR spectrum of triphenyl(3-(4,4,5,5-tetramethyl-1,3,2-dioxaborolan-2-yl)phenyl)germane in $CDCl_3$ at room temperature

Supplementary Fig. 7. ^{1}H NMR spectrum of GeTrzCz2 in $CDCl_3$ at room temperature

Supplementary Fig. 8. ^{13}C NMR spectrum of GeTrzCz2 in $CDCl_3$ at room temperature

Supplementary Fig. 9. HRMS spectrum of GeTrzCz2

Supplementary Fig. 10. ^{1}H NMR spectrum of GeCzCz in $CDCl_3$ at room temperature

Supplementary Fig. 11. ^{13}C NMR spectrum of GeCzCz in $CDCl_3$ at room temperature

Supplementary Fig. 12. HRMS spectrum of GeCzCz

Supplementary Fig. 13. Single crystal structures of HT hosts

Supplementary Fig. 14. Single crystal structures of ET hosts

Supplementary Fig. 15. Phosphorescence spectra of GeCzCz, and GeTrzCz2 in solid films at 77 K

Supplementary Fig. 16. Theoretical calculations of the hosts

Supplementary Fig. 17. Temperature-dependent transient photoluminescence decay spectra of SiCzCz:SiTrzCz2 and GeCzCz:GeTrzCz2 films

Supplementary Fig. 18. Transient photoluminescence decay curves of exciplex films

Supplementary Fig. 19. Theoretically calculated Huang-Rhys factor (*S*) and non-radiative decay rate (k_{nr}) for the $S_1 \rightarrow S_0$ electronic transitions plotted against the vibrational frequency

Supplementary Fig. 20. Spatial distributions of the highest occupied molecular orbital (HOMO) and lowest unoccupied molecular orbital (LUMO) in the optimized configuration between GeCzCz and GeTrzCz2

Supplementary Fig. 21. Theoretical analysis of excited states

Supplementary Fig. 22. Calculated spin-orbit coupling (SOC) constants of donor-acceptor molecular pairs

Supplementary Fig. 23. Normalized PL spectra of GeCzCz:GeTrzCz2 exciplex film and extinction spectrum of *t*-DABNA, *υ*-DABNA and *ω*-DABNA in toluene (10^{-5} mol·L^{-1})

Supplementary Fig. 24. Transient PL decay curves

Supplementary Fig. 25. Current density-voltage characteristics of the hole-only device (HOD) and electron-only device (EOD)

Supplementary Fig. 26. ^{1}H NMR spectrum of 4,4,5,5-tetramethyl-2-(3-tritylphenyl)-1,3,2-dioxaborolane in $CDCl_3$ at room temperature

Supplementary Fig. 27. ^{13}C NMR spectrum of 4,4,5,5-tetramethyl-2-(3-tritylphenyl)-1,3,2-dioxaborolane in $CDCl_3$ at room temperature

Supplementary Fig. 28. ^{1}H NMR spectrum of CTrzCz2 in $CDCl_3$ at room temperature

Supplementary Fig. 29. ^{13}C NMR spectrum of CTrzCz2 in $CDCl_3$ at room temperature

Supplementary Fig. 30. HRMS spectrum of CTrzCz2

Supplementary Fig. 31. ^{1}H NMR spectrum of CCzCz in $CDCl_3$ at room temperature

Supplementary Fig. 32. ^{13}C NMR spectrum of CCzCz in $CDCl_3$ at room temperature

Supplementary Fig. 33. HRMS spectrum of CCzCz

Supplementary Fig. 34. High-performance liquid chromatograms

Supplementary Fig. 35. Chemical structures of the comprising materials in OLEDs

Supplementary Fig. 36. Current density and luminance versus driving voltage curves

Supplementary Fig. 37. Angle-dependent PL spectra

Supplementary Fig. 38. Theoretical maximum external quantum efficiency as a function of the emitting layer PL quantum yield and the horizontal dipole ratio of the emitter for devices

Supplementary Fig. 39. EL transient decay curves of *υ*-DABNA-based device and control devices (control-B), measured at their respective dominant emission peaks

Supplementary Fig. 40. Photolysis results

Supplementary Fig. 41. Cyclic voltammograms of the hosts

Supplementary Fig. 42. Refractive indices of the charge blocking layers and emitting layers

Supplementary Fig. 43. Normalized PL spectra of CCzCz:CTrzCz2 exciplex film and absorption spectrum of *t*-DABNA, *υ*-DABNA and *ω*-DABNA in toluene (10^{-5} mol·L^{-1})

Supplementary Tables

Supplementary Table 1. Atomic dipole moment corrected Hirshfeld atomic charges for the individual atoms in the Ge- and Si-based hosts

Supplementary Table 2. Physical data and kinetic parameters of GeCzCz:GeTrzCz2 and SiCzCz:SiTrzCz2 exciplex films

Supplementary Table 3. Calculated Huang-Rhys factors (S) for the dominant skeletal vibrational modes (where $S > 0.06$) of SiTrzCz2 and GeTrzCz2

Supplementary Table 4. Physical data and kinetic parameters of MR-TADF emitters *t*-DABNA, *υ*-DABNA and *ω*-DABNA doped in exciplex hosts GeCzCz:GeTrzCz2 and SiCzCz:SiTrzCz2

Supplementary Table 5. Summary of recent advances in green-emitting OLEDs with $CIE_y \geq 0.71$ (NTSC standard) and blue-emitting OLEDs with $CIE_y \leq 0.20$

Supplementary Table 6. Silicon- and germanium-related bond dissociation energies in the lowest triplet state

Supplementary Table 7. Computational results for the CCzCz:CTrzCz2 exciplex
Supplementary Table 8. Computational results for the SiCzCz:SiTrzCz2 exciplex
Supplementary Table 9. Computational results for the GeCzCz:GeTrzCz2 exciplex
Supplementary Table 10. Atomic coordinates of CCzCz:CTrzCz2 (01, E = -4271.39577766 a.u.)
Supplementary Table 11. Atomic coordinates of CCzCz:CTrzCz2 (02, E = -4271.38939897 a.u.)
Supplementary Table 12. Atomic coordinates of CCzCz:CTrzCz2 (03, E = -4271.38860717 a.u.)
Supplementary Table 13. Atomic coordinates of CCzCz:CTrzCz2 (04, E = -4271.38107128 a.u.)
Supplementary Table 14. Atomic coordinates of CCzCz:CTrzCz2 (05, E = -4271.37951293 a.u.)
Supplementary Table 15. Atomic coordinates of CCzCz:CTrzCz2 (06, E = -4271.38098577 a.u.)
Supplementary Table 16. Atomic coordinates of CCzCz:CTrzCz2 (07, E = -4271.38051295 a.u.)
Supplementary Table 17. Atomic coordinates of CCzCz:CTrzCz2 (08, E = -4271.37249410 a.u.)
Supplementary Table 18. Atomic coordinates of CCzCz:CTrzCz2 (09, E = -4271.36082798 a.u.)
Supplementary Table 19. Atomic coordinates of CCzCz:CTrzCz2 (10, E = -4271.36709025 a.u.)
Supplementary Table 20. Atomic coordinates of CCzCz:CTrzCz2 (11, E = -4271.38313321 a.u.)
Supplementary Table 21. Atomic coordinates of CCzCz:CTrzCz2 (12, E = -4271.34182102 a.u.)
Supplementary Table 22. Atomic coordinates of CCzCz:CTrzCz2 (13, E = -4271.35486381 a.u.)
Supplementary Table 23. Atomic coordinates of CCzCz:CTrzCz2 (14, E = -4271.37630985 a.u.)
Supplementary Table 24. Atomic coordinates of CCzCz:CTrzCz2 (15, E = -4271.38693226 a.u.)
Supplementary Table 25. Atomic coordinates of CCzCz:CTrzCz2 (16, E = -4271.38468648 a.u.)
Supplementary Table 26. Atomic coordinates of SiCzCz:SiTrzCz2 (01, E = -4774.27150650 a.u.)
Supplementary Table 27. Atomic coordinates of SiCzCz:SiTrzCz2 (02, E = -4774.26206424 a.u.)
Supplementary Table 28. Atomic coordinates of SiCzCz:SiTrzCz2 (03, E = -4774.26047565 a.u.)
Supplementary Table 29. Atomic coordinates of SiCzCz:SiTrzCz2 (04, E = -4774.25162137 a.u.)
Supplementary Table 30. Atomic coordinates of SiCzCz:SiTrzCz2 (05, E = -4774.24903784 a.u.)
Supplementary Table 31. Atomic coordinates of SiCzCz:SiTrzCz2 (06, E = -4774.25120284 a.u.)
Supplementary Table 32. Atomic coordinates of SiCzCz:SiTrzCz2 (07, E = -4774.25471717 a.u.)
Supplementary Table 33. Atomic coordinates of SiCzCz:SiTrzCz2 (08, E = -4774.24448179 a.u.)
Supplementary Table 34. Atomic coordinates of SiCzCz:SiTrzCz2 (09, E = -4774.23401449 a.u.)
Supplementary Table 35. Atomic coordinates of SiCzCz:SiTrzCz2 (10, E = -4774.23673046 a.u.)
Supplementary Table 36. Atomic coordinates of SiCzCz:SiTrzCz2 (11, E = -4774.24978137 a.u.)
Supplementary Table 37. Atomic coordinates of SiCzCz:SiTrzCz2 (12, E = -4774.21243473 a.u.)

Supplementary Table 38. Atomic coordinates of SiCzCz:SiTrzCz2 (13, E = -4774.21695465 a.u.)

Supplementary Table 39. Atomic coordinates of SiCzCz:SiTrzCz2 (14, E = -4774.24798944 a.u.)

Supplementary Table 40. Atomic coordinates of SiCzCz:SiTrzCz2 (15, E = -4774.25820862 a.u.)

Supplementary Table 41. Atomic coordinates of SiCzCz:SiTrzCz2 (16, E = -4774.26101980 a.u.)

Supplementary Table 42. Atomic coordinates of GeCzCz:GeTrzCz2 (01, E = -4202.82082 a.u.)

Supplementary Table 43. Atomic coordinates of GeCzCz:GeTrzCz2 (02, E = -4202.8125823 a.u.)

Supplementary Table 44. Atomic coordinates of GeCzCz:GeTrzCz2 (03, E = -4202.8109488 a.u.)

Supplementary Table 45. Atomic coordinates of GeCzCz:GeTrzCz2 (04, E = -4202.8027052 a.u.)

Supplementary Table 46. Atomic coordinates of GeCzCz:GeTrzCz2 (05, E = -4202.7988948 a.u.)

Supplementary Table 47. Atomic coordinates of GeCzCz:GeTrzCz2 (06, E = -4202.8023799 a.u.)

Supplementary Table 48. Atomic coordinates of GeCzCz:GeTrzCz2 (07, E = -4202.8057458 a.u.)

Supplementary Table 49. Atomic coordinates of GeCzCz:GeTrzCz2 (08, E = -4202.7949005 a.u.)

Supplementary Table 50. Atomic coordinates of GeCzCz:GeTrzCz2 (09, E = -4202.7859437 a.u.)

Supplementary Table 51. Atomic coordinates of GeCzCz:GeTrzCz2 (10, E = -4202.7880771 a.u.)

Supplementary Table 52. Atomic coordinates of GeCzCz:GeTrzCz2 (11, E = -4202.8009526 a.u.)

Supplementary Table 53. Atomic coordinates of GeCzCz:GeTrzCz2 (12, E = -4202.7626843 a.u.)

Supplementary Table 54. Atomic coordinates of GeCzCz:GeTrzCz2 (13, E = -4202.7671612 a.u.)

Supplementary Table 55. Atomic coordinates of GeCzCz:GeTrzCz2 (14, E = -4202.7980205 a.u.)

Supplementary Table 56. Atomic coordinates of GeCzCz:GeTrzCz2 (15, E = -4202.8092433 a.u.)

Supplementary Table 57. Atomic coordinates of GeCzCz:GeTrzCz2 (16, E = -4202.8127934 a.u.)

Supplementary References

Supplementary Notes

Supplementary Note 1. Materials synthesis and characterization.

Synthesis of triphenyl(3-(4,4,5,5-tetramethyl-1,3,2-dioxaborolan-2-yl)phenyl)germane. Under an argon atmosphere, (3-bromophenyl)triphenylgermane (6.90 g, 15.00 mmol), 4,4,4',4',5,5,5',5'-octamethyl-2,2'-bi(1,3,2-dioxaborolane) (7.60 g, 30.00 mmol), Pd(dppf)Cl_2 (0.22 g, 0.30 mmol), and potassium acetate (4.40 g, 45 mmol) were dissolved in dehydrated DMSO (50 mL) and stirred at 100 °C for 12 h. Upon cooling to room temperature, the black mixture was diluted with water and extracted with ethyl acetate (3 × 30 mL). The organic phases were dried over anhydrous sodium sulfate and the solvent was removed under reduced pressure. The crude product was purified by column chromatography on silica gel using ethyl acetate/petroleum ether (*v*/*v* = 1/30) as eluent to afford a white solid (5.00 g, yield: 65%). ^{1}H NMR ($CDCl_3$, 400 MHz) δ (ppm): 1.31 (s, 12H), 7.34-7.40 (m, 10H), 7.52-7.54 (m, 6H), 7.59 (d, *J* = 8.0 Hz, 1H), 7.86 (d, *J* = 8.0 Hz, 1H), 8.05 (s, 1H). ^{13}C NMR ($CDCl_3$, 100 MHz) δ (ppm): 24.8, 83.7, 127.6, 128.2, 129.0, 135.2, 135.4(2), 135.6, 136.2, 138.3, 141.4.

Synthesis of GeTrzCz2. Under an argon atmosphere, triphenyl(3-(4,4,5,5-tetramethyl-1,3,2-dioxaborolan-2-yl)phenyl)germane (1.80 g, 3.60 mmol), 9,9'-(6-chloro-1,3,5-triazine-2,4-diyl)bis(9*H*-carbazole) (1.30 g, 3.00 mmol), Pd(PPh_3)$_4$ (0.18 g, 0.16 mmol), and potassium carbonate (1.20 g, 9.00 mmol) were dissolved in THF/water (20 mL each) and stirred at 65 °C for 12 h. Upon cooling to room temperature, the black mixture was diluted with water and extracted with dichloromethane (3 × 30 mL). The organic phases were dried over anhydrous sodium sulfate and the solvent was removed under reduced pressure. The crude product was purified by column chromatography on silica gel using dichloromethane/petroleum ether (*v*/*v* = 1/4) as eluent to afford a white solid (1.80 g, yield: 75%). ^{1}H NMR ($CDCl_3$, 500 MHz) δ (ppm): 7.36-7.44 (m, 17H), 7.64 (d, *J* = 7.5 Hz, 6H), 7.69 (t, *J* = 7.5 Hz, 1H), 7.85 (d, *J* = 7.0 Hz, 1H), 8.08 (d, *J* = 7.5 Hz, 4H), 8.78 (d, *J* = 7.5 Hz, 1H), 8.96 (d, *J* = 8.0 Hz, 4H), 9.04 (s, 1H). ^{13}C NMR ($CDCl_3$, 125 MHz) δ (ppm): 117.6, 119.6, 123.3, 126.5, 127.0(2), 128.5(2), 128.9, 129.4(2), 130.1, 135.4(2), 135.7(2), 135.9, 136.1, 137.5, 138.9, 139.9, 164.7(2), 173.2(2). HRMS (ESI): m/z $[M+H]^+$ calcd for $C_{51}H_{36}GeN_5^+$: 792.2177; found: 792.2177.

Synthesis of GeCzCz. Under an argon atmosphere, (3-bromophenyl)triphenylgermane (2.30 g, 5.00 mmol), 9*H*-3,9'-bicarbazole (2.00 g, 6.00 mmol), $Pd_2(dba)_3$ (0.18 g, 0.20 mmol), *tri-tert*-butylphosphine tetrafluoroborate (0.12 g, 0.41 mmol), sodium *tert*-butoxide (0.72 g, 7.5 mmol) were dissolved in toluene (30 mL) and stirred at 100 ^oC for 12 h. Upon cooling to room temperature, the black mixture was diluted with water and extracted with dichloromethane (3 × 30 mL). The organic phases were dried over anhydrous sodium sulfate and the solvent was removed under reduced pressure. The crude product was purified by column chromatography on silica gel using dichloromethane/petroleum ether (v/v = 1/5) as eluent to afford a white solid (3.00 g, yield: 85%). ^1H NMR ($CDCl_3$, 400 MHz) δ (ppm): 7.29 (t, J = 8.0 Hz, 3H), 7.36-7.45 (m, 15H), 7.49 (d, J = 8.0 Hz, 1H), 7.55 (d, J = 8.0 Hz, 1H), 7.60-7.62 (m, 6H), 7.66-7.72 (m, 3H), 7.82 (s, 1H), 8.08 (d, J = 8.0 Hz, 1H), 8.18 (d, J = 8.0 Hz, 2H), 8.25 (s, 1H). ^{13}C NMR ($CDCl_3$, 125 MHz) δ (ppm): 109.8, 110.1, 110.9, 119.4, 119.6, 120.3, 120.4, 120.5, 123.0, 123.1, 124.4, 125.4, 125.8, 126.6, 127.4, 128.5, 129.4, 129.8, 129.9, 133.5, 134.4, 135.4(2), 137.4, 139.2, 139.8, 141.3, 141.9. HRMS (ESI): m/z $[M+H]^+$ calcd for $C_{48}H_{35}GeN_2^+$: 713.2007; found: 713.2003.

Synthesis of 4,4,5,5-tetramethyl-2-(3-tritylphenyl)-1,3,2-dioxaborolane. Under an argon atmosphere, ((3-bromophenyl)methanetriyl)tribenzene (5.97 g, 15.00 mmol), 4,4,4',4',5,5,5',5'-octamethyl-2,2'-bi(1,3,2-dioxaborolane) (7.60 g, 30.00 mmol), Pd(dppf)Cl_2 (0.22 g, 0.30 mmol), and potassium acetate (4.40 g, 45 mmol) were dissolved in dehydrated DMSO (50 mL) and stirred at 100 ^oC for 12 h. Upon cooling to room temperature, the black mixture was diluted with water and extracted with ethyl acetate (3 × 30 mL). The organic phases were dried over anhydrous sodium sulfate and the solvent was removed under reduced pressure. The crude product was purified by column chromatography on silica gel using ethyl acetate/petroleum ether (*v*/*v* = 1/30) as eluent to

afford a white solid (4.02 g, yield: 60%). ^{1}H NMR ($CDCl_3$, 500 MHz) δ (ppm): 1.28 (s, 12H), 7.15-7.19 (m, 3H), 7.22-7.24 (m, 13H), 7.31 (d, *J* = 6.5 Hz, 1H), 7.64 (d, *J* = 6.0 Hz, 1H), 7.72 (s, 1H). ^{13}C NMR ($CDCl_3$, 125 MHz) δ (ppm): 24.8, 65.0, 83.6, 125.8, 126.9, 127.5, 131.2, 132.5, 134.3, 136.8, 145.9, 146.9.

Synthesis of CTrzCz2. Under an argon atmosphere, 4,4,5,5-tetramethyl-2-(3-tritylphenyl)-1,3,2-dioxaborolane (1.61 g, 3.60 mmol), 9,9'-(6-chloro-1,3,5-triazine-2,4-diyl)bis(9*H*-carbazole) (1.30 g, 3.00 mmol), $Pd(PPh_3)_4$ (0.18 g, 0.16 mmol), and potassium carbonate (1.20 g, 9.00 mmol) were dissolved in THF/water (20 mL each) and stirred at 65 ºC for 12 h. Upon cooling to room temperature, the black mixture was diluted with water and extracted with dichloromethane (3 × 30 mL). The organic phases were dried over anhydrous sodium sulfate and the solvent was removed under reduced pressure. The crude product was purified by column chromatography on silica gel using dichloromethane/petroleum ether (*v*/*v* = 1/4) as eluent to afford a white solid (0.96 g, yield: 44%). ^{1}H NMR ($CDCl_3$, 400 MHz) δ (ppm): 7.21-7.23 (m, 4H), 7.30 (t, *J* = 7.6 Hz, 6H), 7.36-7.41 (m, 13H), 7.58 (t, *J* = 7.6 Hz, 1H), 7.63 (d, *J* = 8.0 Hz, 1H), 8.05-8.09 (m, 4H), 8.58 (d, *J* = 7.6 Hz, 1H), 8.74 (s, 1H), 8.88-8.92 (m, 4H). ^{13}C NMR ($CDCl_3$, 125 MHz) δ (ppm): 65.2, 117.6, 119.6, 123.2, 126.2, 126.5, 126.9, 127.0, 127.8, 128.3, 131.1, 132.4, 135.0, 135.5, 138.9, 146.6, 147.8, 164.6, 173.2. HRMS (ESI): m/z $[M+H]^+$ calcd for $C_{52}H_{36}N_5^+$: 730.2965; found: 730.2943.

Synthesis of CCzCz. Under an argon atmosphere, ((3-bromophenyl)methanetriyl)tribenzene (1.99 g, 5.00 mmol), 9*H*-3,9'-bicarbazole (2.00 g, 6.00 mmol), $Pd_2(dba)_3$ (0.18 g, 0.20 mmol), *tri*-*tert*-butylphosphine tetrafluoroborate (0.12 g, 0.41 mmol), sodium *tert*-butoxide (0.72 g, 7.5 mmol) were

dissolved in toluene (30 mL) and stirred at 100 ºC for 12 h. Upon cooling to room temperature, the black mixture was diluted with water and extracted with dichloromethane (3 × 30 mL). The organic phases were dried over anhydrous sodium sulfate and the solvent was removed under reduced pressure. The crude product was purified by column chromatography on silica gel using dichloromethane/petroleum ether (v/v = 1/5) as eluent to afford a white solid (1.76 g, yield: 54%). ^{1}H NMR ($CDCl_3$, 500 MHz) δ (ppm): 7.21 (t, *J* = 7.0 Hz, 3H), 7.24 (s, 1H), 7.26-7.27 (m, 2H), 7.30 (t, *J* = 7.0 Hz, 8H), 7.33 (t, *J* = 7.5 Hz, 6H), 7.34-7.37 (m, 2H), 7.39-7.44 (m, 5H), 7.50 (d, *J* = 8.0 Hz, 2H), 7.53 (t, *J* = 7.5 Hz, 1H), 8.04 (d, *J* = 7.5 Hz, 1H), 8.18 (d, *J* = 8.0 Hz, 2H), 8.21 (d, *J* = 2.5 Hz, 1H). ^{13}C NMR ($CDCl_3$, 125 MHz) δ (ppm): 65.2, 109.8, 110.1, 110.8, 119.4, 119.6, 120.3(2), 120.5, 122.9, 123.1, 124.0, 124.3, 125.3, 125.8, 126.2, 126.5, 127.8, 129.1, 129.2, 129.8, 130.3, 131.0, 136.6, 139.6, 141.1, 141.8, 145.8, 149.3. HRMS (ESI): m/z $[M+H]^+$ calcd for $C_{49}H_{35}N_2^+$: 651.2795; found: 651.2784.

Supplementary Note 2. Calculation method of $k_{r,S}$, $k_{nr,S}$ and k_{RISC}.

The respective rate constants of the prompt and delayed fluorescence components (k_p and k_d) occurring in the exciplex or terminal MR-TADF emitter, can be given by:

$$k_p, k_d = \frac{k_{r,S} + k_{nr,S} + k_{ISC} + k_{r,T} + k_{nr,T} + k_{RISC}}{2} \times \left(1 \pm \sqrt{1 - \frac{4\left(k_{r,S} + k_{nr,S} + k_{ISC}\right)\left(k_{r,T} + k_{nr,T} + k_{RISC}\right) - 4k_{ISC}k_{RISC}}{\left(k_{r,S} + k_{nr,S} + k_{ISC} + k_{r,T} + k_{nr,T} + k_{RISC}\right)^2}}\right). \tag{1}$$

k_p and k_d could be experimentally determined from prompt and delayed fluorescence decay times τ_p, τ_d as follows:

$$k_p = \frac{1}{\tau_p}. \tag{2}$$

$$k_d = \frac{1}{\tau_d}. \tag{3}$$

Besides, the emission quantum yields Φ_p and Φ_d for the prompt and delayed fluorescence components have the following relationship with these rate constants:

$$\Phi_p = \frac{k_{r,S}}{k_p} \frac{k_{r,S} + k_{nr,S} + k_{ISC} - k_d}{k_p - k_d}. \tag{4}$$

$$\Phi_d = \frac{k_{r,S}}{k_d} \frac{k_p - k_{r,S} - k_{nr,S} - k_{ISC}}{k_p - k_d}. \tag{5}$$

From the equations above, one could obtain the following relationship between rate constants and k_p, k_d, Φ_p and Φ_d experimentally determined from typical PLQY and transient PL characteristics:

$$k_{r,S} = \Phi_p k_p + \Phi_d k_d. \tag{6}$$

$$k^S = k_{r,S} + k_{nr,S} + k_{ISC} = \frac{\Phi_p k_p^2 + \Phi_d k_d^2}{k_{r,S}}. \tag{7}$$

$$k^T = k_{r,T} + k_{nr,T} + k_{RISC} = \frac{\left(\Phi_p + \Phi_d\right) k_p k_d}{k_{r,S}}. \tag{8}$$

$$k_{ISC} k_{RISC} = \frac{\Phi_p \Phi_d k_p k_d \left(k_p - k_d\right)^2}{k_{r,S}^2}. \tag{9}$$

Supplementary Note 3. Calculation method of k_{DET}.

In the presence of the MR-TADF emitters, the respective rate constants of the prompt and delayed fluorescence components (k_p and k_d) occurring in the exciplex host, can be given by:

$$k_p, k_d = \frac{k_{FET} + k_{r,S} + k_{nr,S} + k_{ISC} + k_{DET} + k_{r,T} + k_{nr,T} + k_{RISC}}{2} \times \left(1 \pm \sqrt{1 - \frac{4\left(k_{FET} + k_{r,S} + k_{nr,S} + k_{ISC}\right)\left(k_{DET} + k_{r,T} + k_{nr,T} + k_{RISC}\right) - 4k_{ISC}k_{RISC}}{\left(k_{FET} + k_{r,S} + k_{nr,S} + k_{ISC} + k_{DET} + k_{r,T} + k_{nr,T} + k_{RISC}\right)^2}}\right). \quad (10)$$

Using Vieta's formula, the sum of k_p and k_d as well as the product of k_p and k_d can be expressed as:

$$k_p + k_d = k_{FET} + k_{r,S} + k_{nr,S} + k_{ISC} + k_{DET} + k_{r,T} + k_{nr,T} + k_{RISC}. \quad (11)$$

$$k_p k_d = \left(k_{FET} + k_{r,S} + k_{nr,S} + k_{ISC}\right)\left(k_{DET} + k_{r,T} + k_{nr,T} + k_{RISC}\right) - k_{ISC}k_{RISC}. \quad (12)$$

By employing approximations, k_{DET} can be simplified as follows:

$$k_{DET} = \frac{1}{\tau_{d,1}} - \frac{1}{\tau_{d,0}} + k_{ISC}k_{RISC}\left(\tau_{p,1} - \tau_{p,0}\right). \quad (13)$$

$\tau_{p,1}$ and $\tau_{p,0}$ represent prompt fluorescence decay times with or without guests, respectively. Likewise, $\tau_{d,1}$ and $\tau_{d,0}$ represent delayed fluorescence decay times with or without guests, respectively.

Supplementary Figures

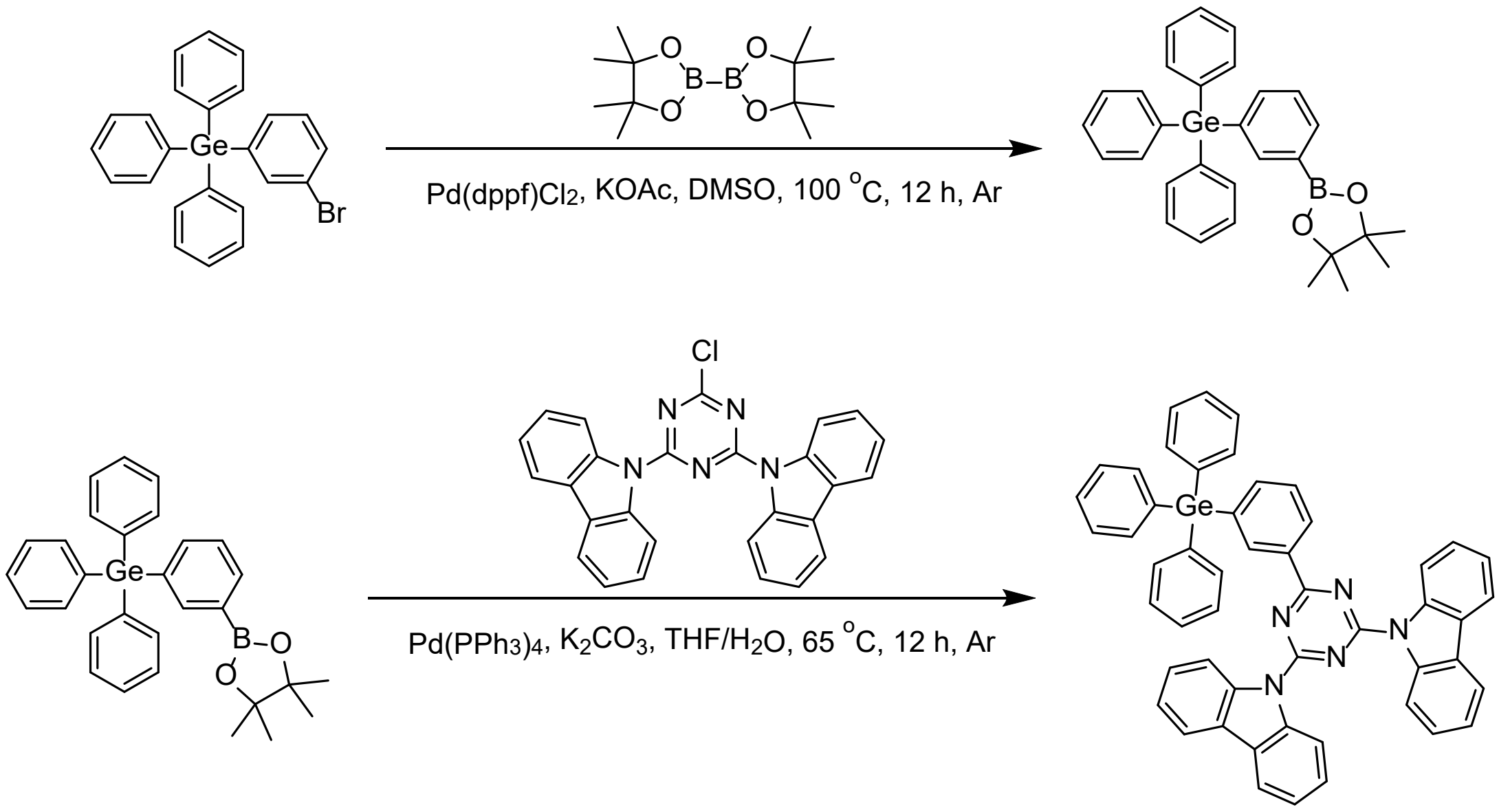

Supplementary Fig. 1. Synthesis procedure of GeTrzCz2.

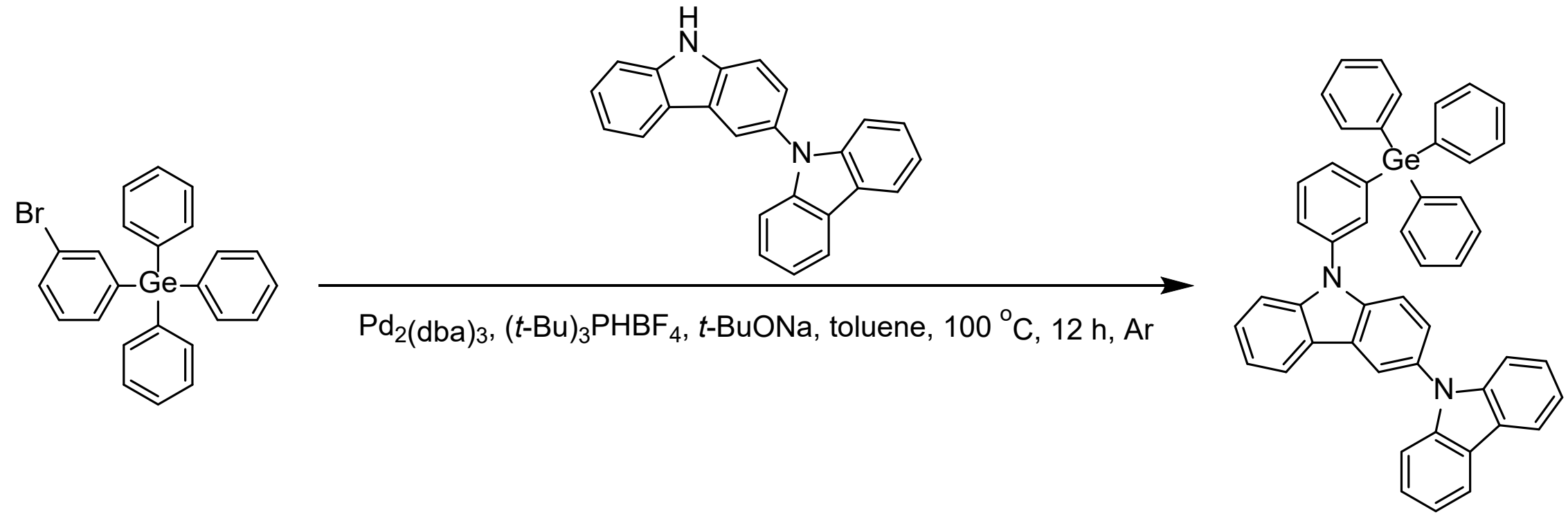

Supplementary Fig. 2. Synthesis procedure of GeCzCz.

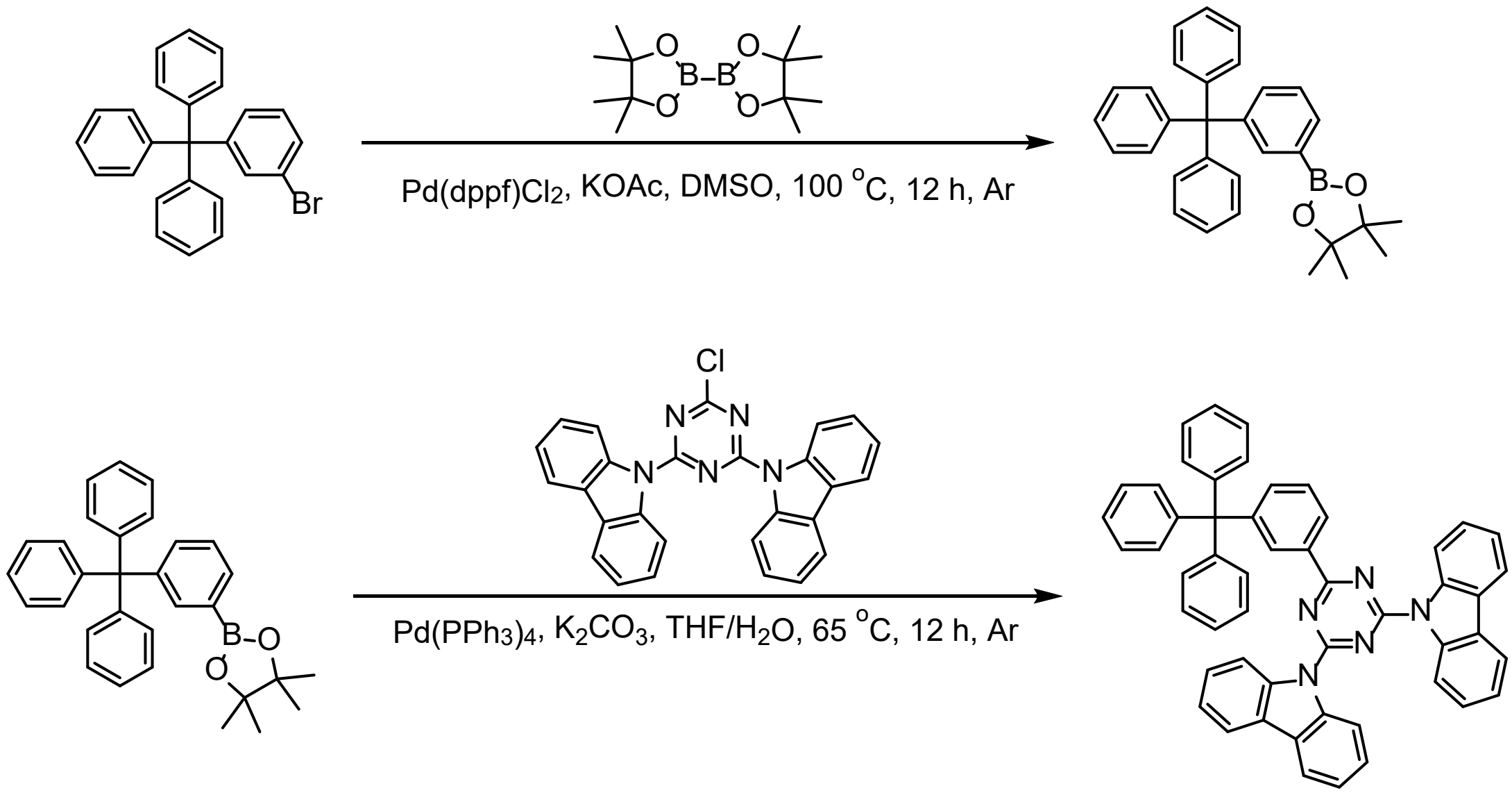

Supplementary Fig. 3. Synthesis procedure of CTrzCz2.

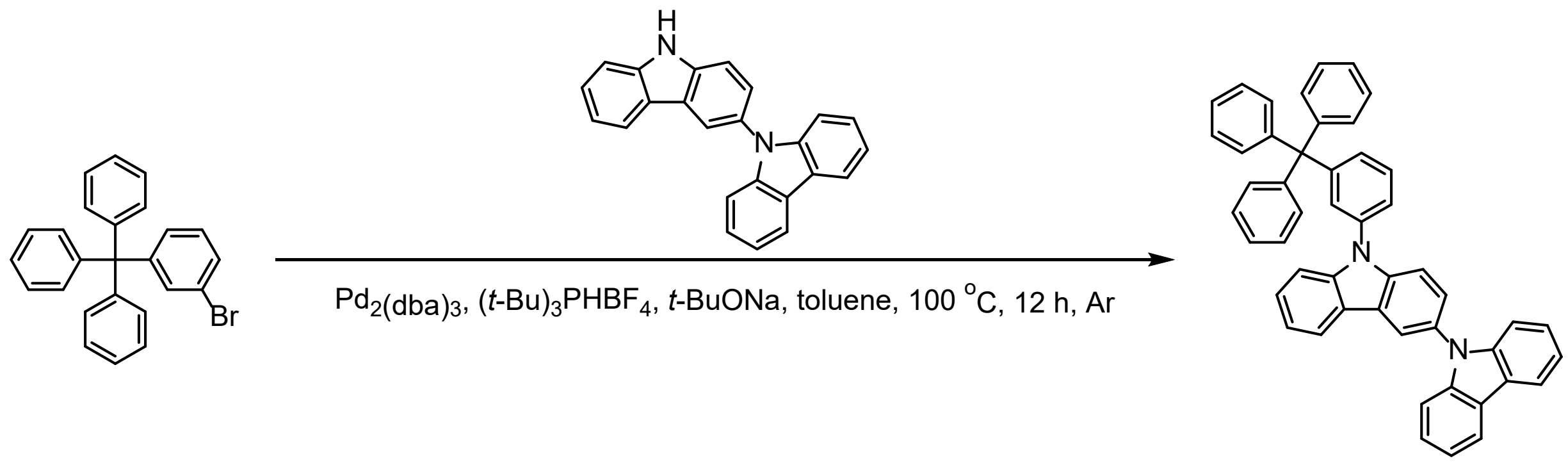

Supplementary Fig. 4. Synthesis procedure of CCzCz.

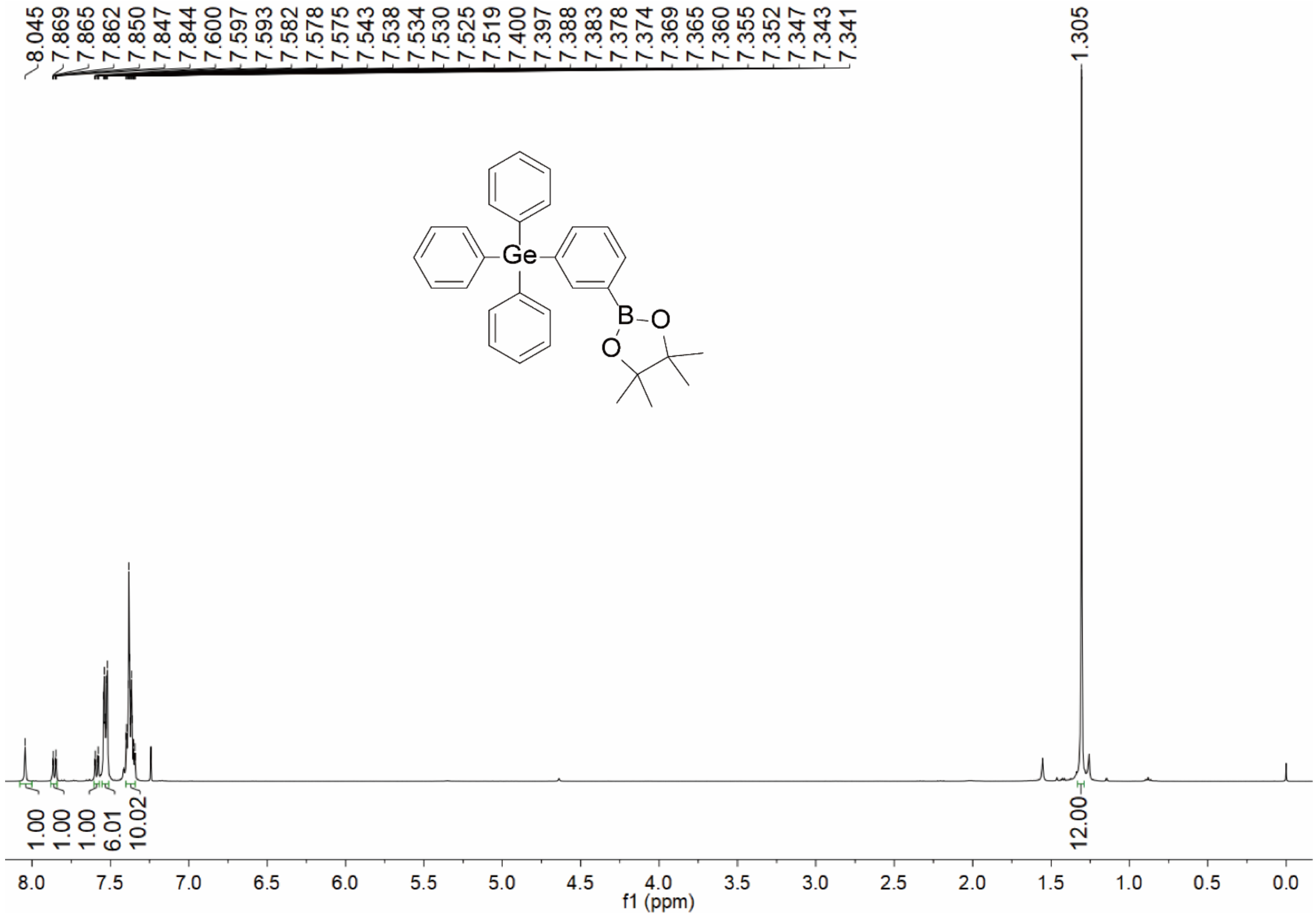

Supplementary Fig. 5. **^{1}H NMR spectrum of triphenyl(3-(4,4,5,5-tetramethyl-1,3,2-dioxaborolan-2-yl)phenyl)germane in $CDCl_3$ at room temperature.**

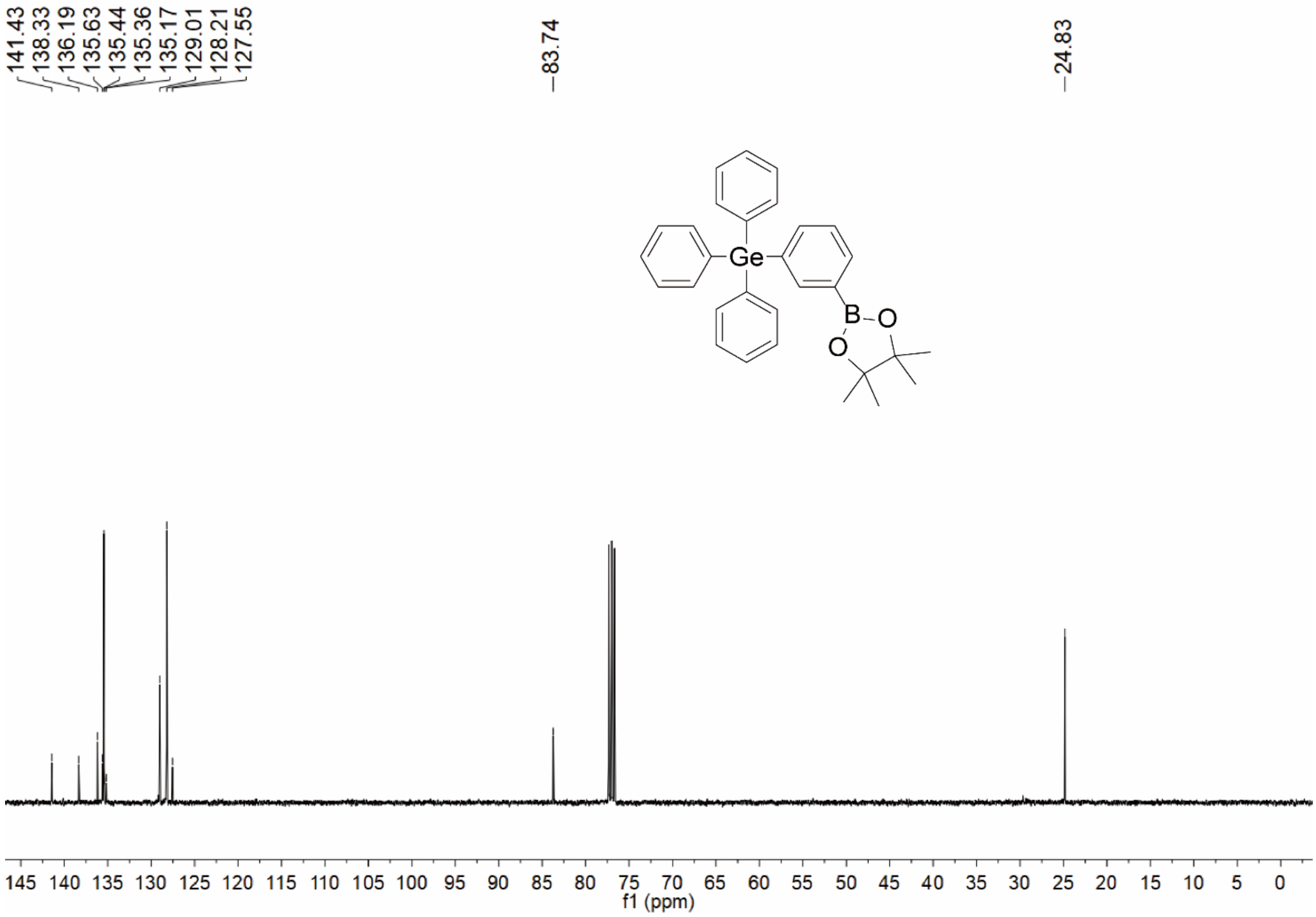

Supplementary Fig. 6. ^{13}C NMR spectrum of triphenyl(3-(4,4,5,5-tetramethyl-1,3,2-dioxaborolan-2-yl)phenyl)germane in $CDCl_3$ at room temperature.

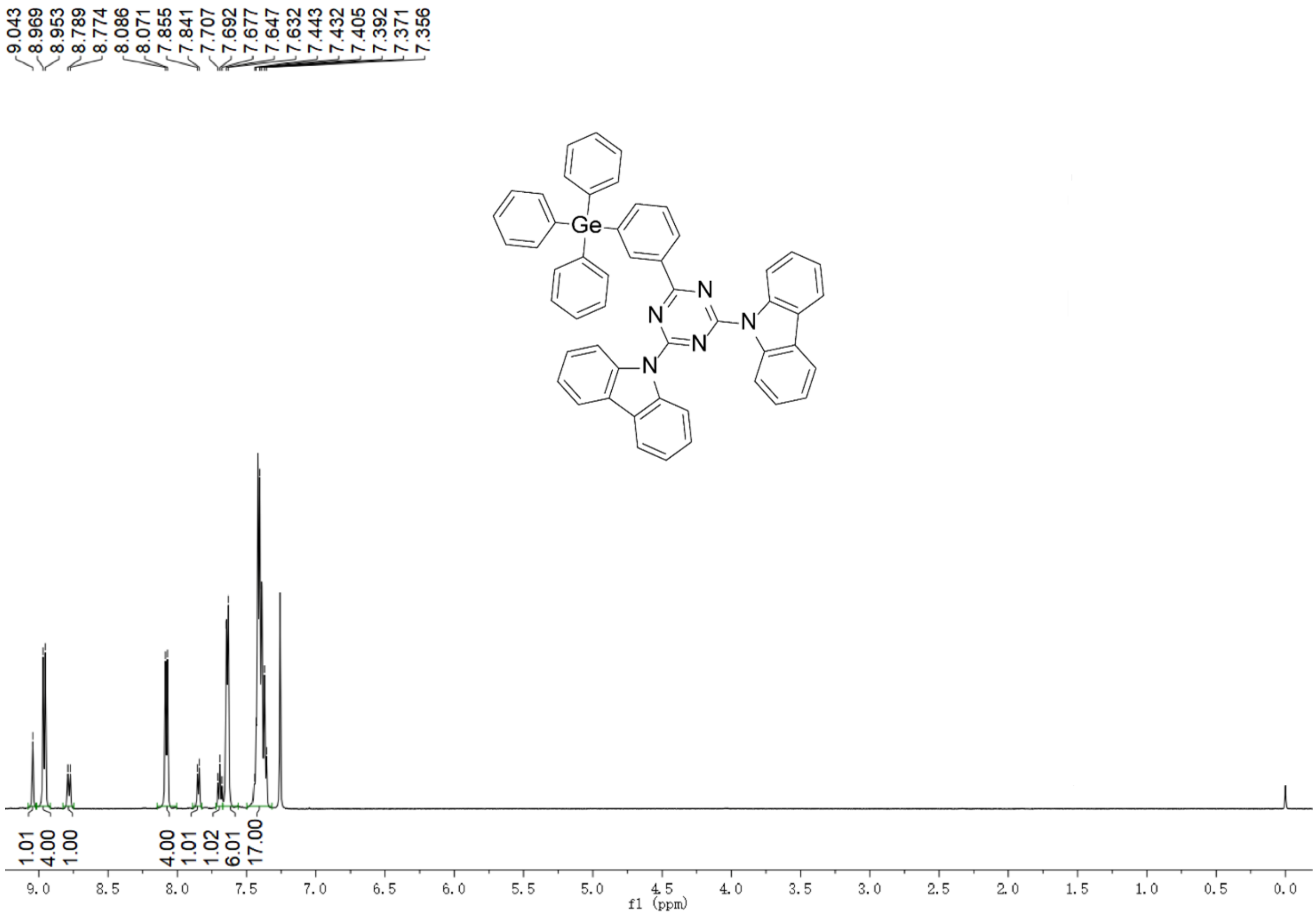

Supplementary Fig. 7. ^{1}H NMR spectrum of GeTrzCz2 in $CDCl_3$ at room temperature.

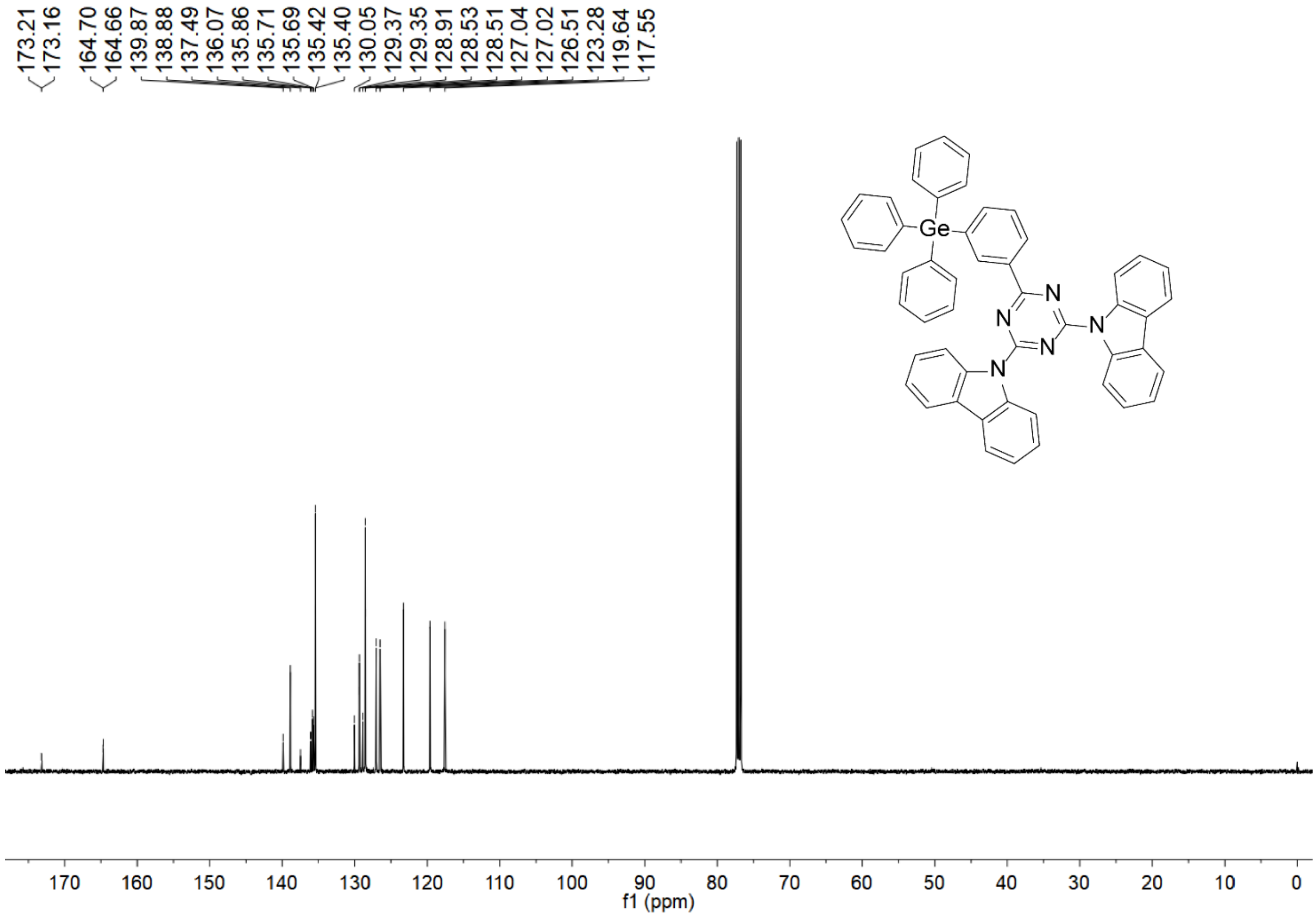

Supplementary Fig. 8. ^{13}C NMR spectrum of GeTrzCz2 in $CDCl_3$ at room temperature.

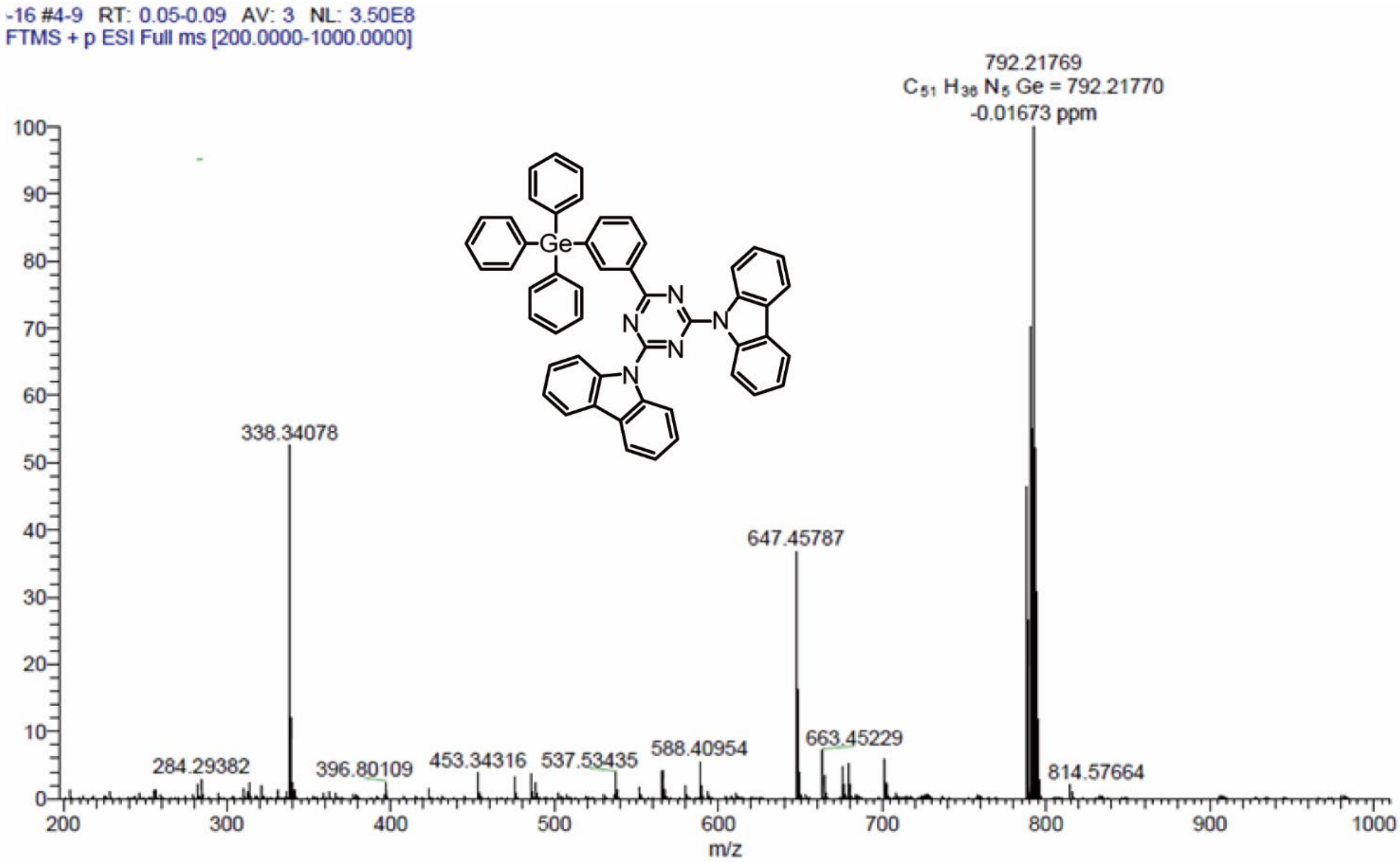

Supplementary Fig. 9. HRMS spectrum of GeTrzCz2.

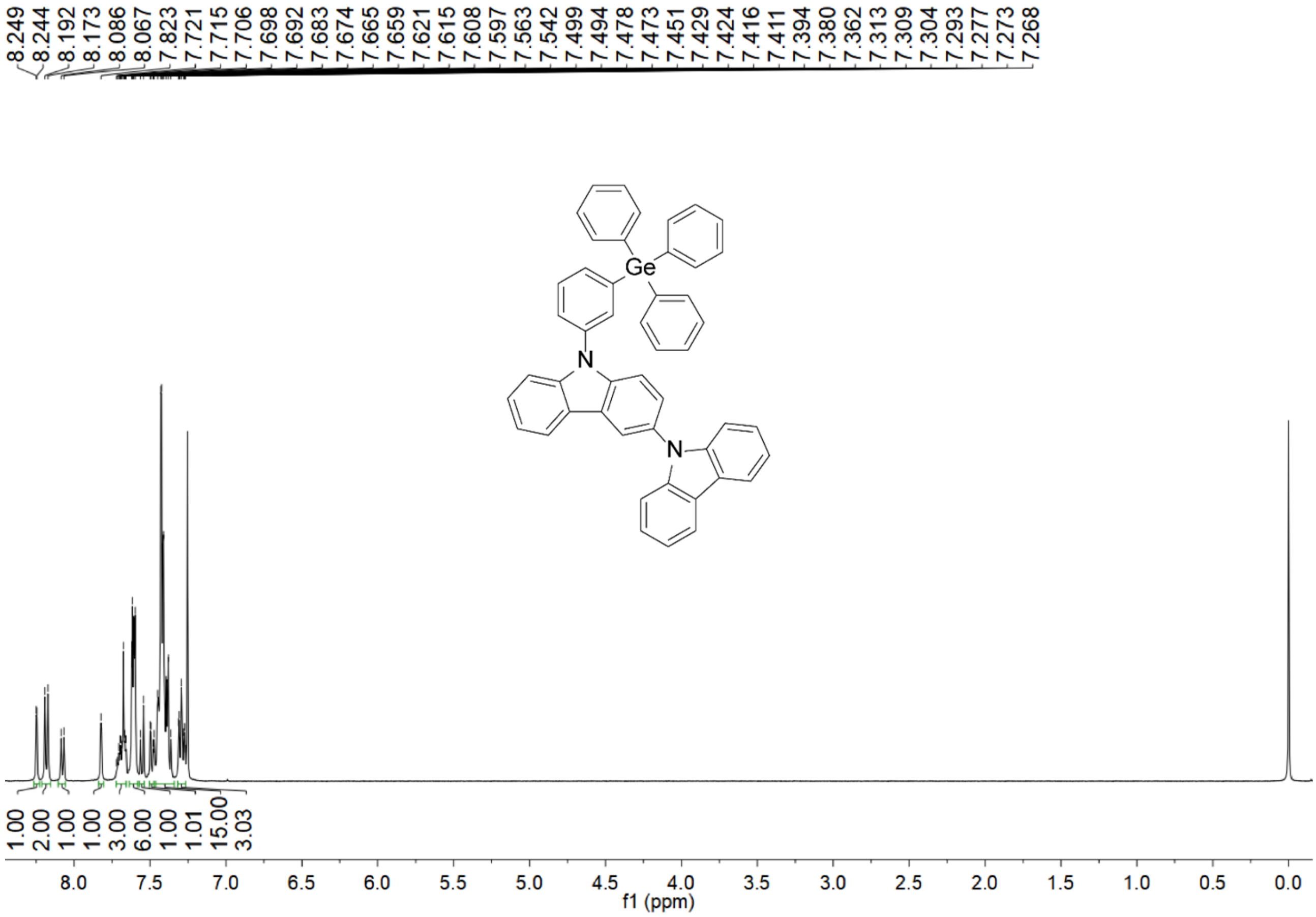

Supplementary Fig. 10. ^{1}H NMR spectrum of GeCzCz in $CDCl_3$ at room temperature.

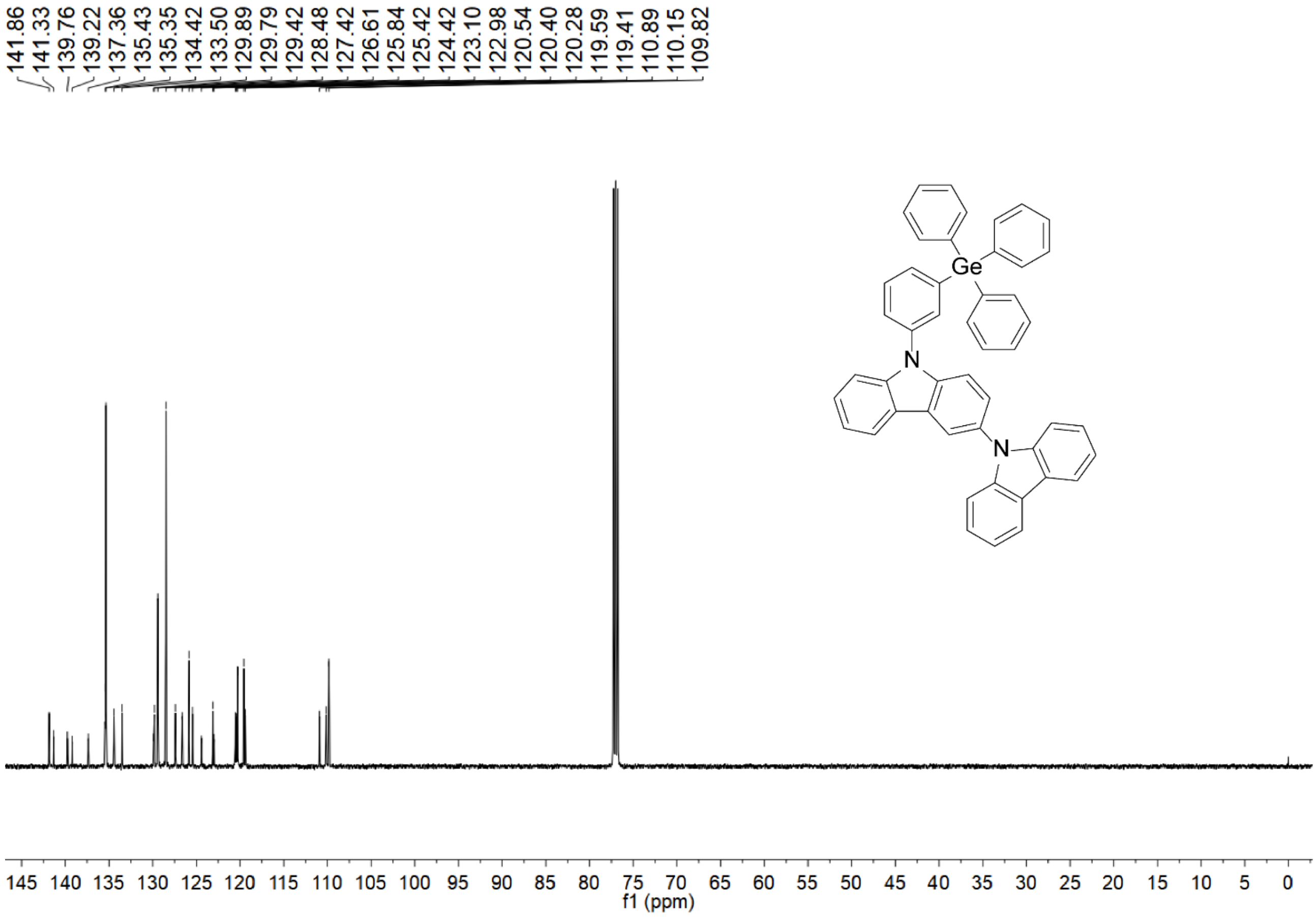

Supplementary Fig. 11. ^{13}C NMR spectrum of GeCzCz in $CDCl_3$ at room temperature.

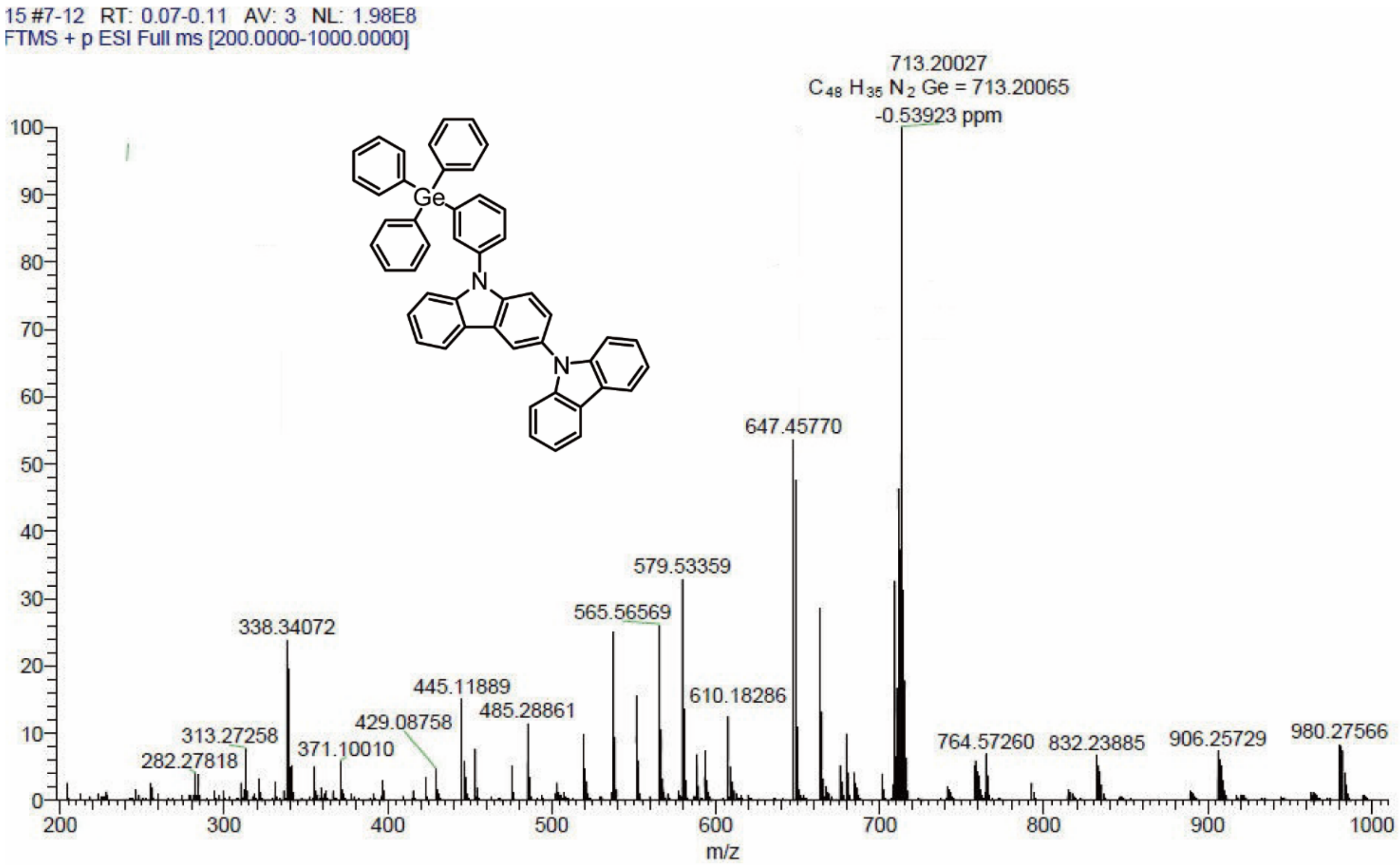

Supplementary Fig. 12. HRMS spectrum of GeCzCz.

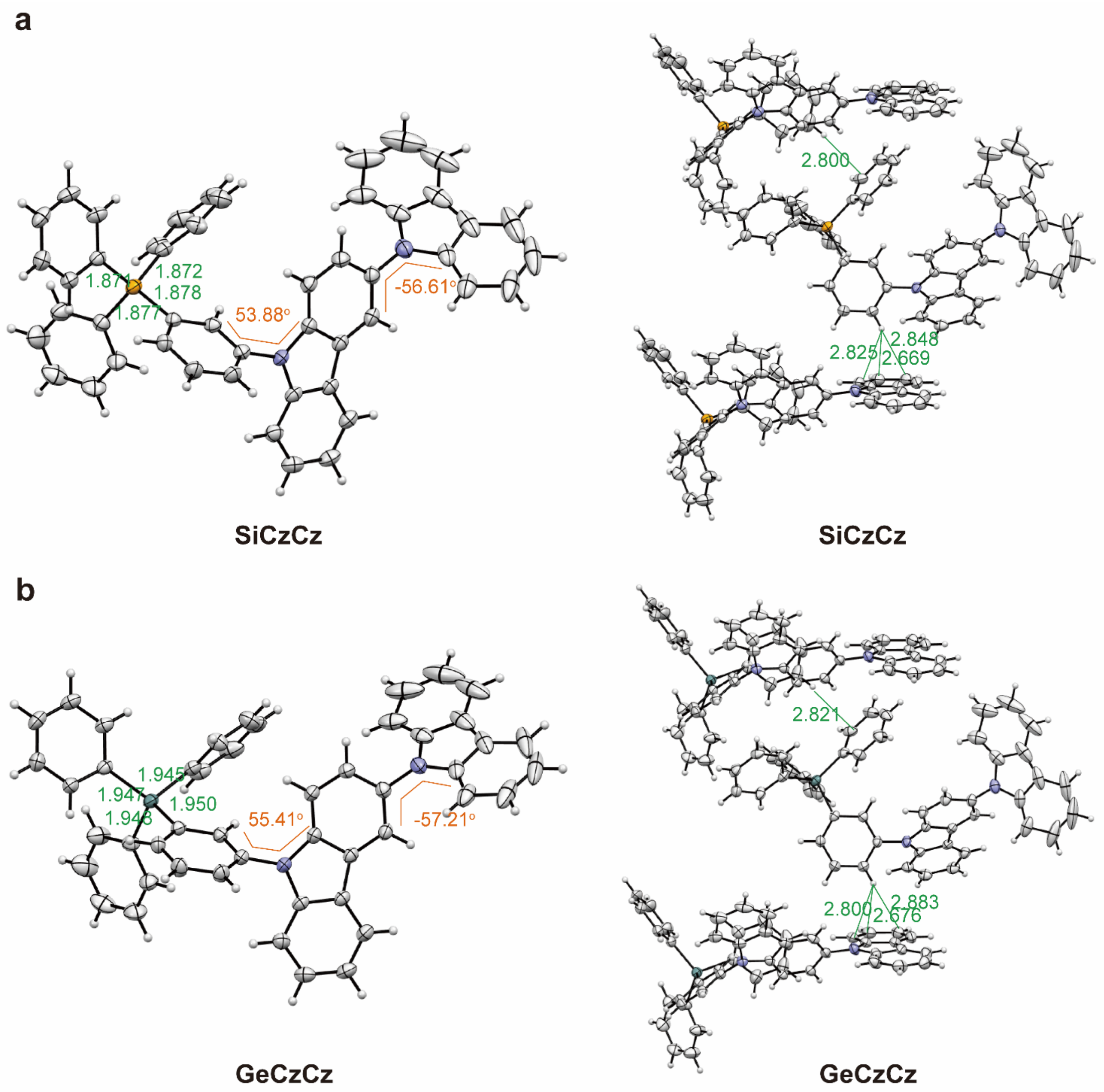

Supplementary Fig. 13. Single crystal structures of HT hosts. (**a**) SiCzCz (CCDC: 2433937, crystal density: 1.237 g·cm^{-3}). (**b**) GeCzCz (CCDC: 2433938, crystal density: 1.309 g·cm^{-3}). The two crystals were grown by slow evaporating a solution in hexane at 25°C. Selected bond length (Å) and angles (°) are annotated.

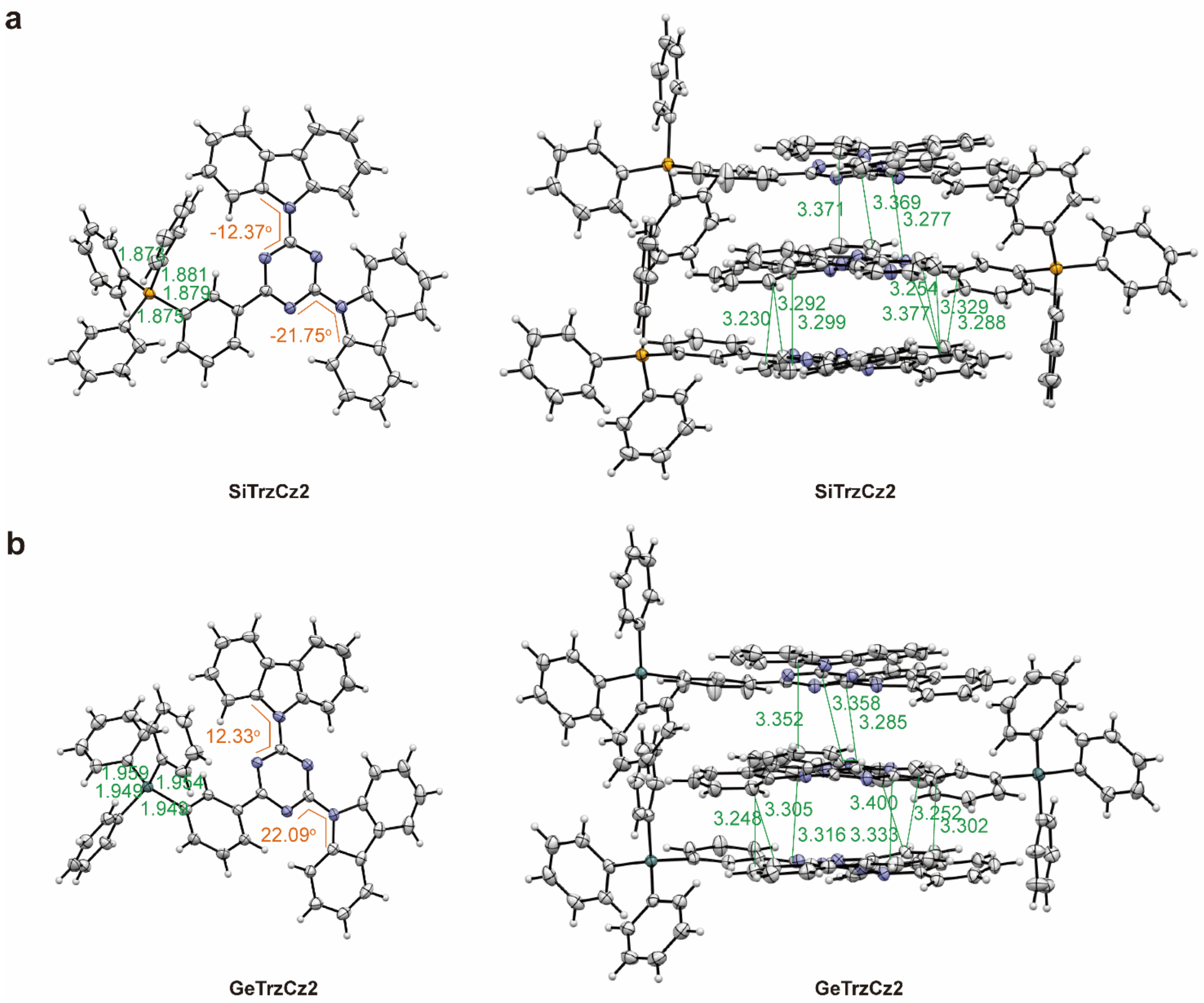

Supplementary Fig. 14. Single crystal structures of ET hosts. (**a**) SiTrzCz2 (CCDC: 2433936, crystal density: 1.324 g·cm^{-3}). (**b**) GeTrzCz2 (CCDC: 2433939, crystal density: 1.390 g·cm^{-3}). The two crystals were grown by slow evaporating a solution in CH_2Cl_2:methanol (5:2) at 25°C. Selected bond length (Å) and angles (°) are annotated.

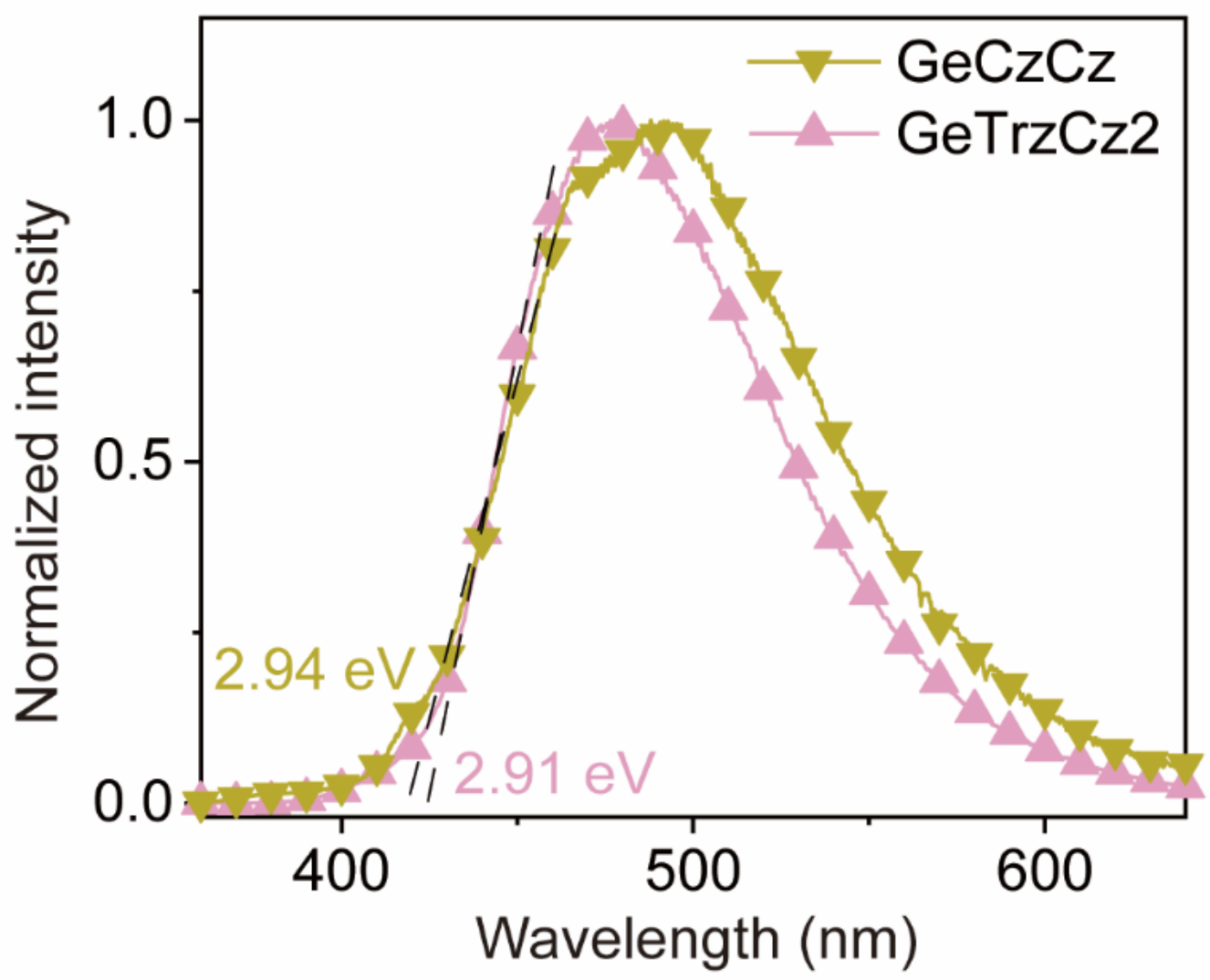

Supplementary Fig. 15. Phosphorescence spectra of GeCzCz, and GeTrzCz2 in solid films at 77 K.

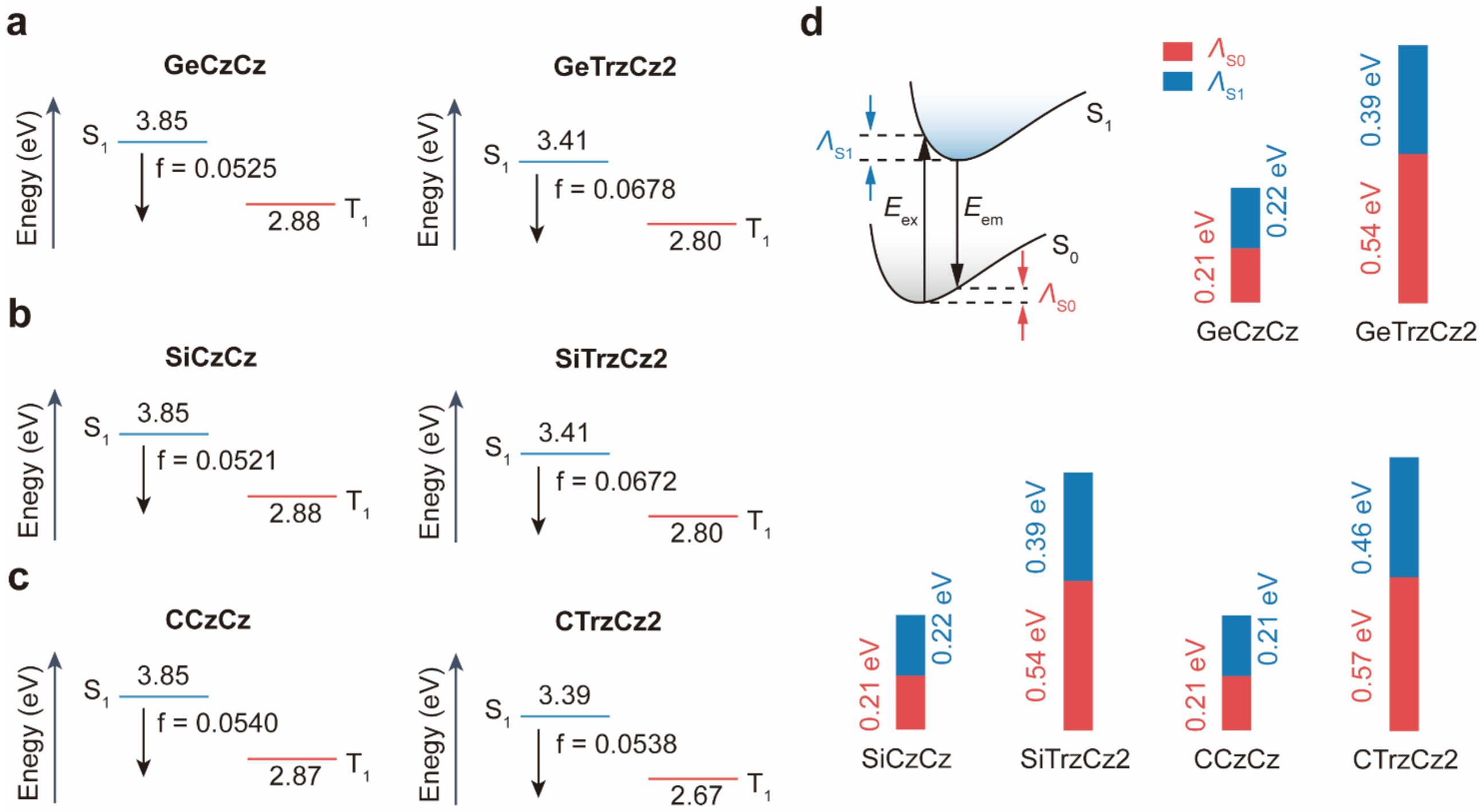

Supplementary Fig. 16. Theoretical calculations of the hosts. (**a**) Transition energies and oscillator strengths of GeCzCz and GeTrzCz2. (**b**) Transition energies and oscillator strengths of SiCzCz and SiTrzCz2. (**c**) Transition energies and oscillator strengths of CCzCz and CTrzCz2. (**d**) Reorganization energy.

The calculated singlet (S_1) and triplet (T_1) energies, as well as the reorganization energies, of SiCzCz and SiTrzCz2 are nearly identical to those of GeCzCz and GeTrzCz2. In contrast, the carbon-based analogues, particularly CTrzCz2, exhibit noticeable differences. Specifically, CTrzCz2 possesses lower calculated S_1 and T_1 energies of 3.39 and 2.67 eV, a lower oscillator strength ($f = 0.0538$), and a significantly higher reorganization energy of 1.03 eV compared to its silicon and germanium counterparts.

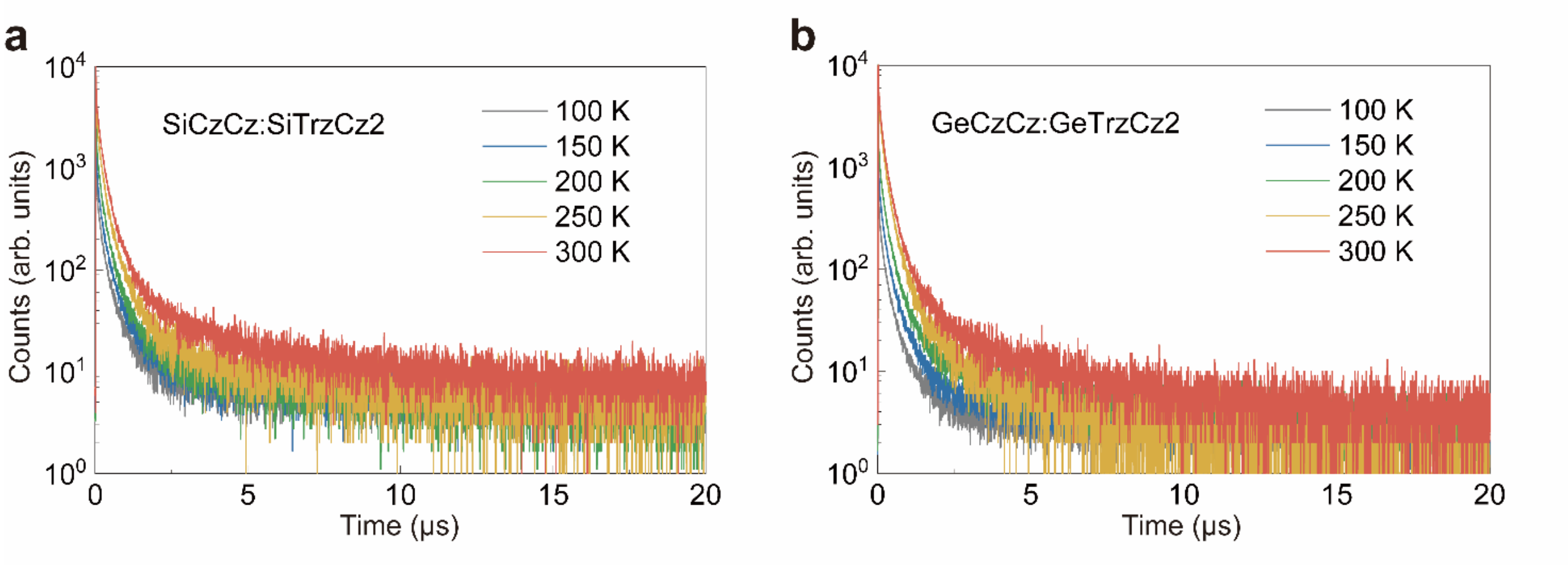

Supplementary Fig. 17. Temperature-dependent transient photoluminescence decay spectra of SiCzCz:SiTrzCz2 and GeCzCz:GeTrzCz2 films.

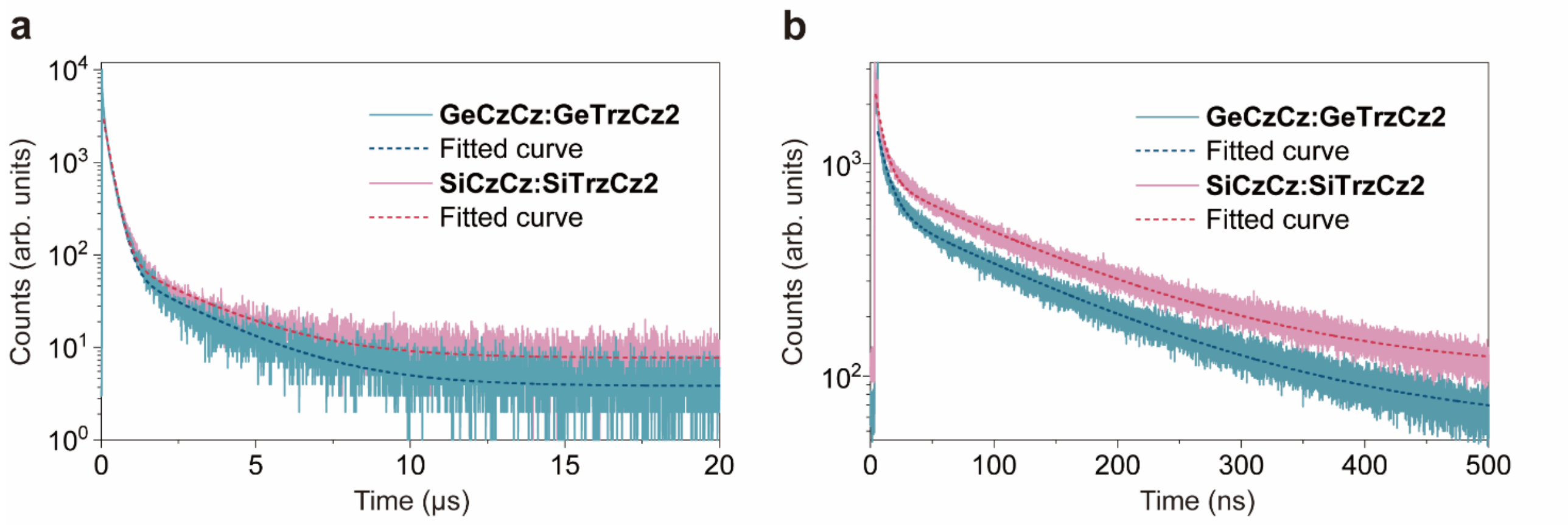

Supplementary Fig. 18. Transient photoluminescence decay curves of exciplex films. GeCzCz:GeTrzCz2 and SiCzCz:SiTrzCz2 films at (**a**) μs-scale and (**b**) ns-scale, theoretical decay curves from bi-exponential fit procedure are shown as dash lines.

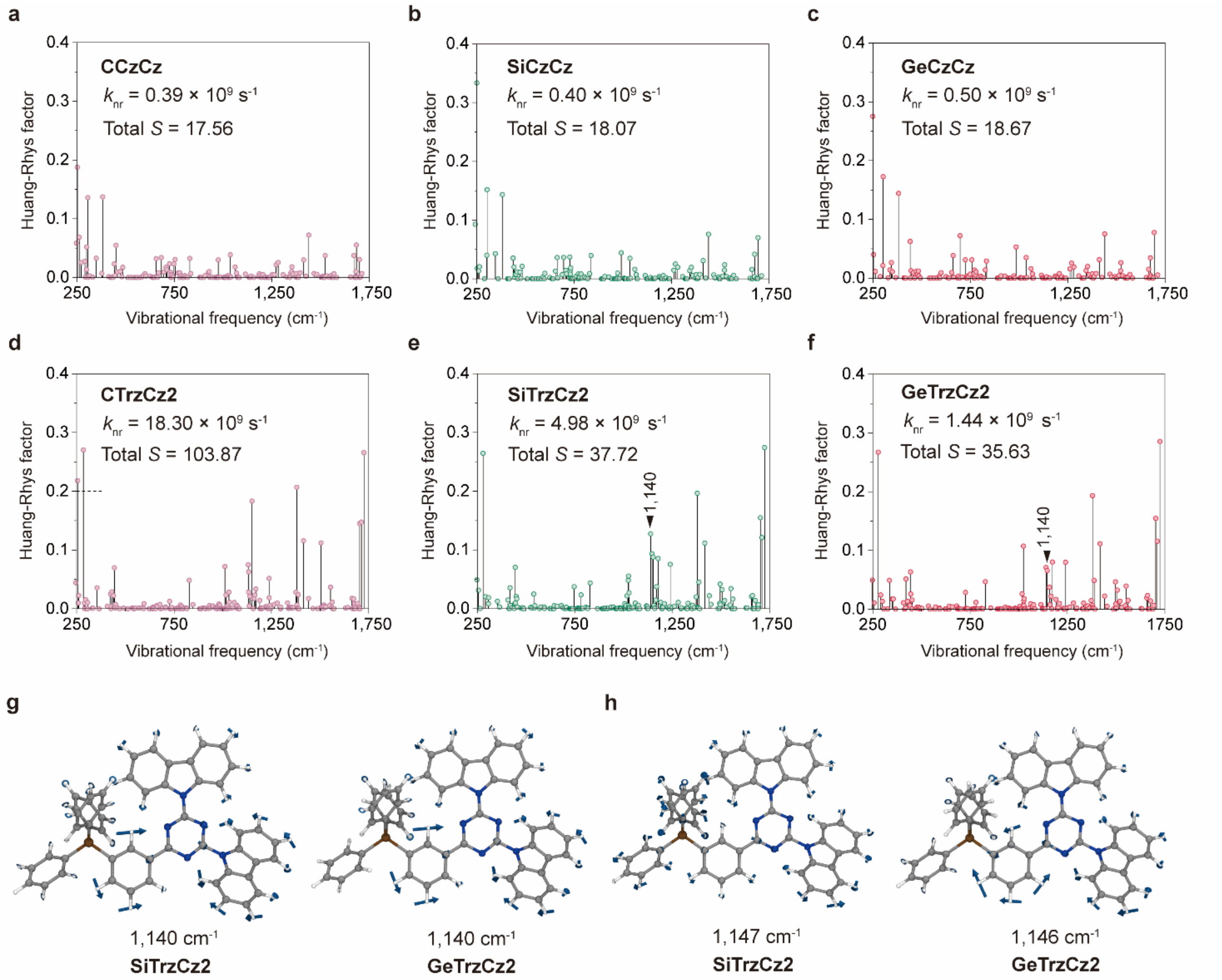

Supplementary Fig. 19. Theoretically calculated Huang-Rhys factor (*S*) and non-radiative decay rate (k_{nr}) for the $S_1 \rightarrow S_0$ electronic transitions plotted against the vibrational frequency. (**a**) CCzCz. (**b**) SiCzCz. (**c**) GeCzCz. (**d**) CTrzCz2. (**e**) SiTrzCz2. (**f**) GeTrzCz2. (**g**) Frequency 1,140 cm^{-1} plotted on the optimized S_1 geometry of SiTrzCz2 and GeTrzCZ2. (**h**) Frequency 1,147 cm^{-1} plotted on the optimized S_1 geometry of SiTrzCz2 and frequency 1,146 cm^{-1} plotted on the optimized S_1 geometry of GeTrzCz2.

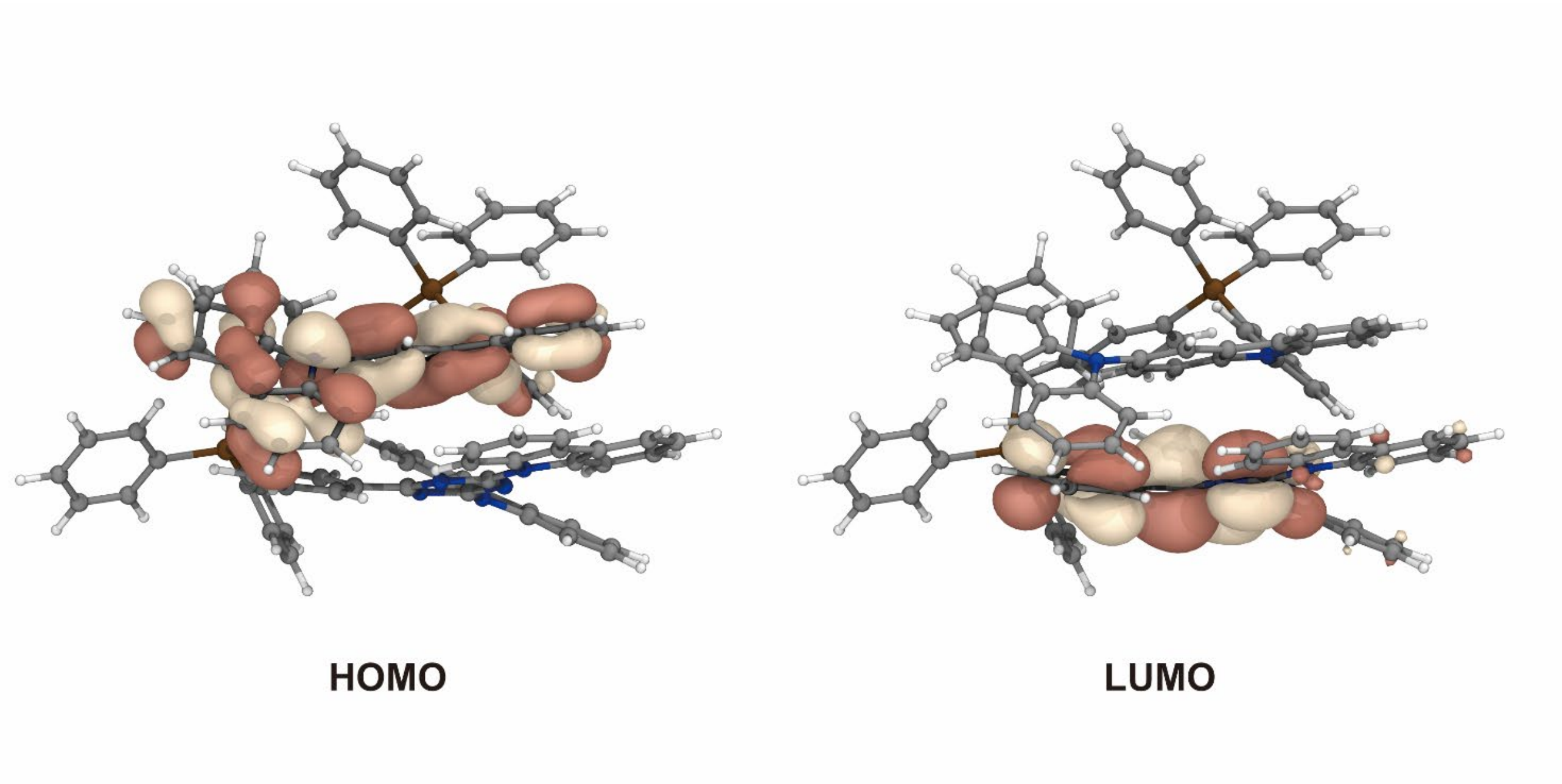

Supplementary Fig. 20. Spatial distributions of the highest occupied molecular orbital (HOMO) and lowest unoccupied molecular orbital (LUMO) in the optimized configuration between GeCzCz and GeTrzCz2.

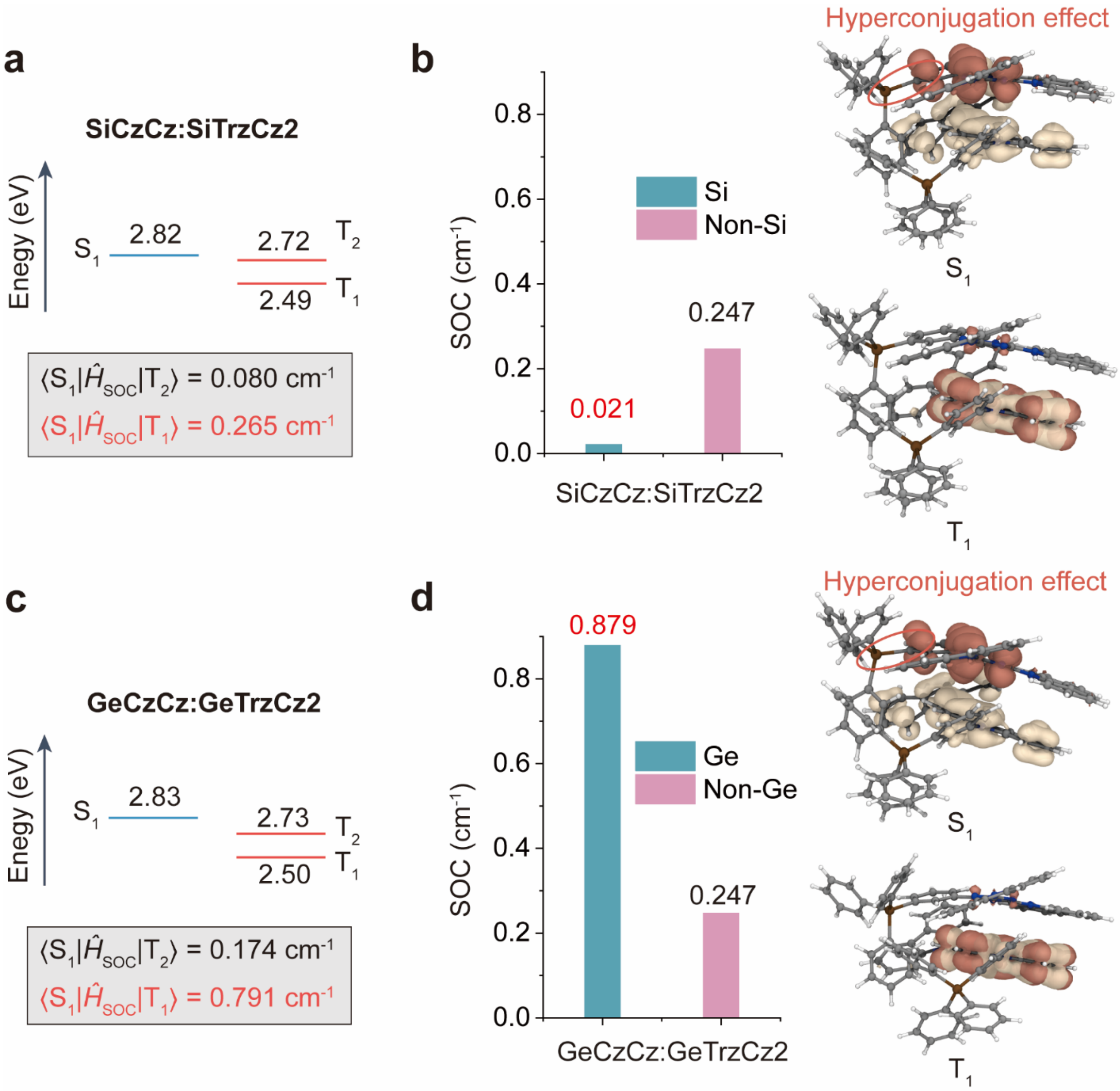

Supplementary Fig 21. Theoretical analysis of excited states. (**a**) Calculated transition energies and spin-orbit coupling (SOC) constants for SiCzCz:SiTrzCz2. (**b**) Atomic and fragment contributions to SOC, together with natural transition orbitals (NTOs) for SiCzCz:SiTrzCz2. (**c**) Calculated transition energies and SOC constants for GeCzCz:GeTrzCz2. (**d**) Atomic and fragment contributions to SOC, together with NTOs for GeCzCz:GeTrzCz2.

Regarding hyperconjugation, natural bond orbital (NBO) analysis shows that the second-order perturbation energy ($E(2)$) for the $\sigma \rightarrow \pi^*$ coupling in the GeCzCz:GeTrzCz2 (1.25 $kcal \cdot mol^{-1}$) is higher than that in the SiCzCz:SiTrzCz2 (0.73 $kcal \cdot mol^{-1}$).

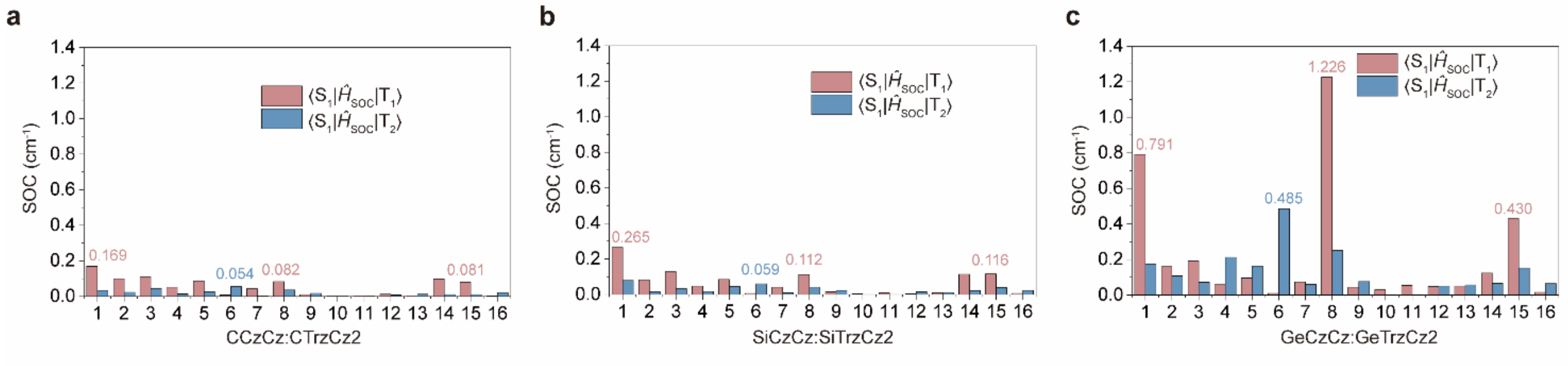

Supplementary Fig. 22. Calculated spin-orbit coupling (SOC) constants of donor-acceptor molecular pairs. (**a**) CCzCz:CTrzCz2. (**b**) SiCzCz:SiTrzCz2. (**c**) GeCzCz:GeTrzCz2.

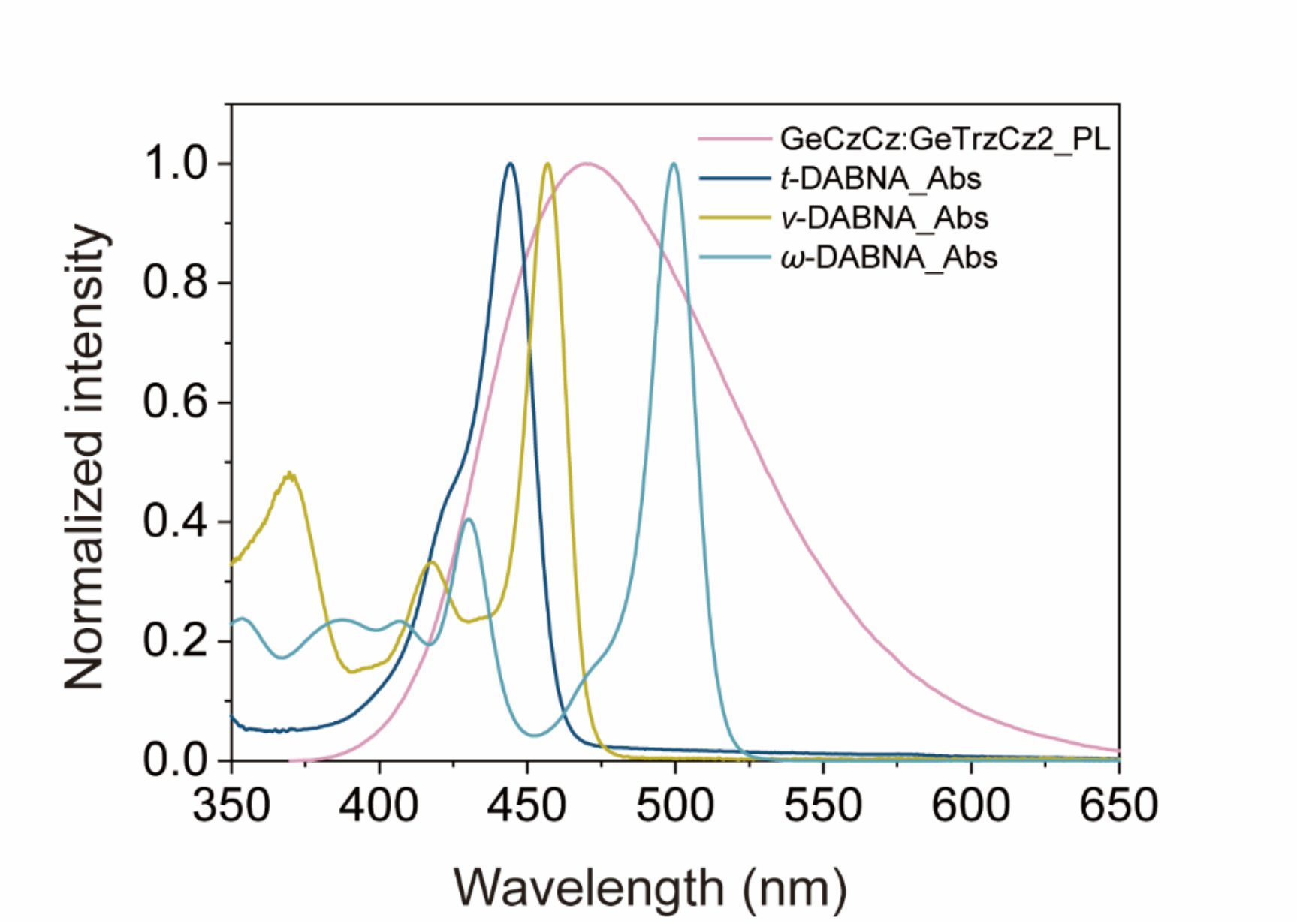

Supplementary Fig. 23. Normalized PL spectra of GeCzCz:GeTrzCz2 exciplex film and extinction spectrum of *t*-DABNA, *υ*-DABNA and *ω*-DABNA in toluene (10^{-5} mol·L^{-1}).

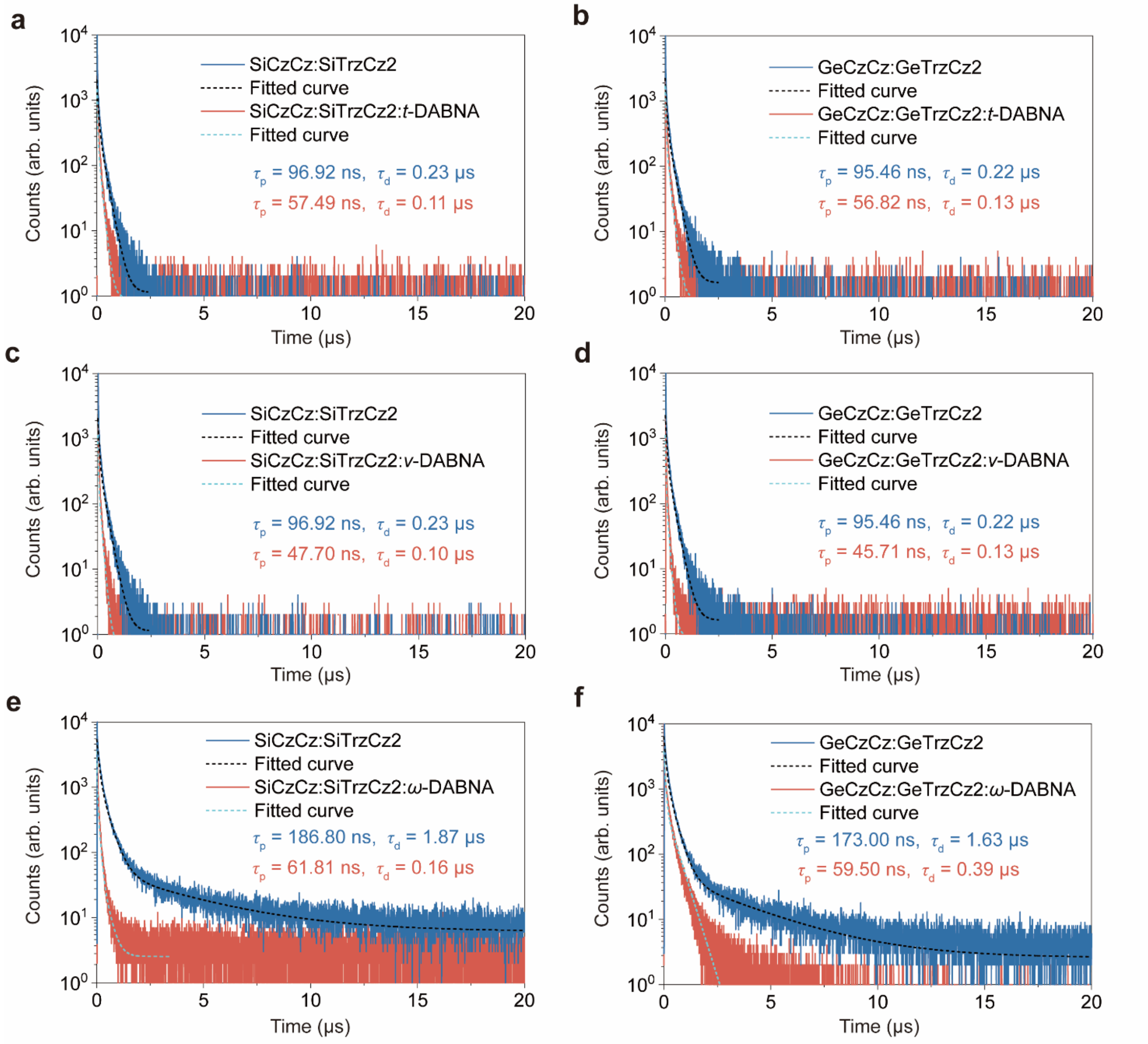

Supplementary Fig. 24. Transient PL decay curves. (**a**) The films with or without *t*-DABNA doping in the SiCzCz:SiTrzCz2 matrix at the emission wavelength of 425 nm. (**b**) The films with or without *t*-DABNA doping in the GeCzCz:GeTrzCz2 matrix at the emission wavelength of 425 nm. (**c**) The films with or without *υ*-DABNA doping in the SiCzCz:SiTrzCz2 matrix at the emission wavelength of 425 nm. (**d**) The films with or without *υ*-DABNA doping in the GeCzCz:GeTrzCz2 matrix at the emission wavelength of 425 nm. (**e**) The films with or without *ω*-DABNA doping in the SiCzCz:SiTrzCz2 matrix at the emission wavelength of 468 nm. (**f**) The films with or without *ω*-DABNA doping in the GeCzCz:GeTrzCz2 matrix at the emission wavelength of 468 nm.

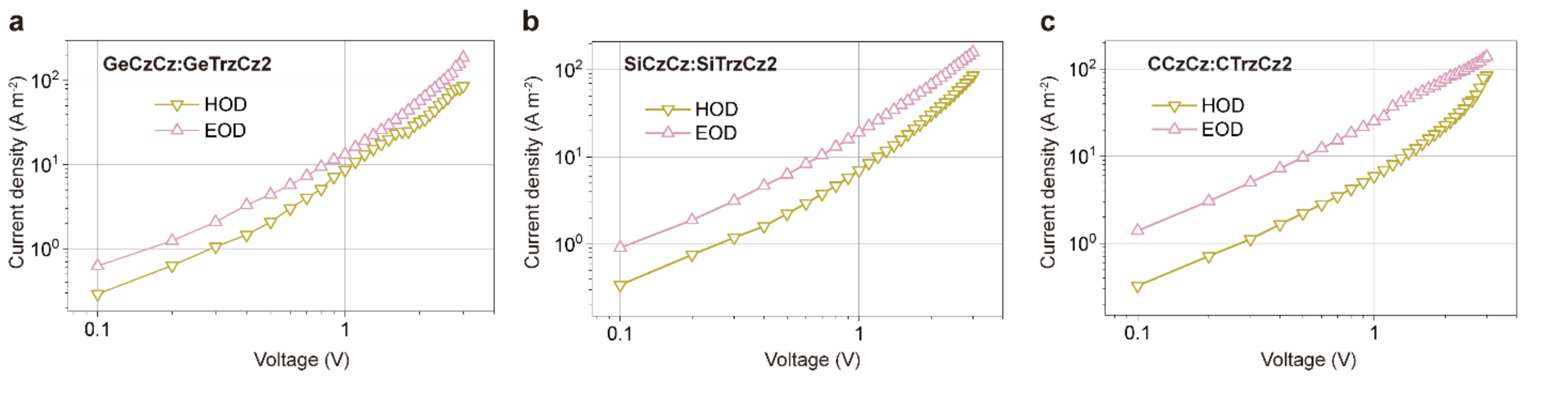

Supplementary Fig. 25. Current density-voltage characteristics of the hole-only device (HOD) and electron-only device (EOD). (**a**) The device structure of the HOD and EOD were constructed as ITO/HATCN (5 nm)/GeCzCz:GeTrzCz2 (100 nm, 0.50/0.50 w/w)/HATCN (20 nm)/Al (100 nm) and ITO/Al_2O_3 (30 nm)/Liq (2 nm)/GeCzCz:GeTrzCz2 (100 nm, 0.50/0.50 w/w)/Liq (2 nm)/Al (100 nm), respectively. (**b**) The device structure of the HOD and EOD were constructed as ITO/HATCN (5 nm)/SiCzCz:SiTrzCz2 (100 nm, 0.50/0.50 w/w)/HATCN (20 nm)/Al (100 nm) and ITO/Al_2O_3 (30 nm)/Liq (2 nm)/SiCzCz:SiTrzCz2 (100 nm, 0.50/0.50 w/w)/Liq (2 nm)/Al (100 nm), respectively. (**c**) The device structure of the HOD and EOD were constructed as ITO/HATCN (5 nm)/CCzCz:CTrzCz2 (100 nm, 0.50/0.50 w/w)/HATCN (20 nm)/Al (100 nm) and ITO/Al_2O_3 (30 nm)/Liq (2 nm)/CCzCz:CTrzCz2 (100 nm, 0.50/0.50 w/w)/Liq (2 nm)/Al (100 nm), respectively.

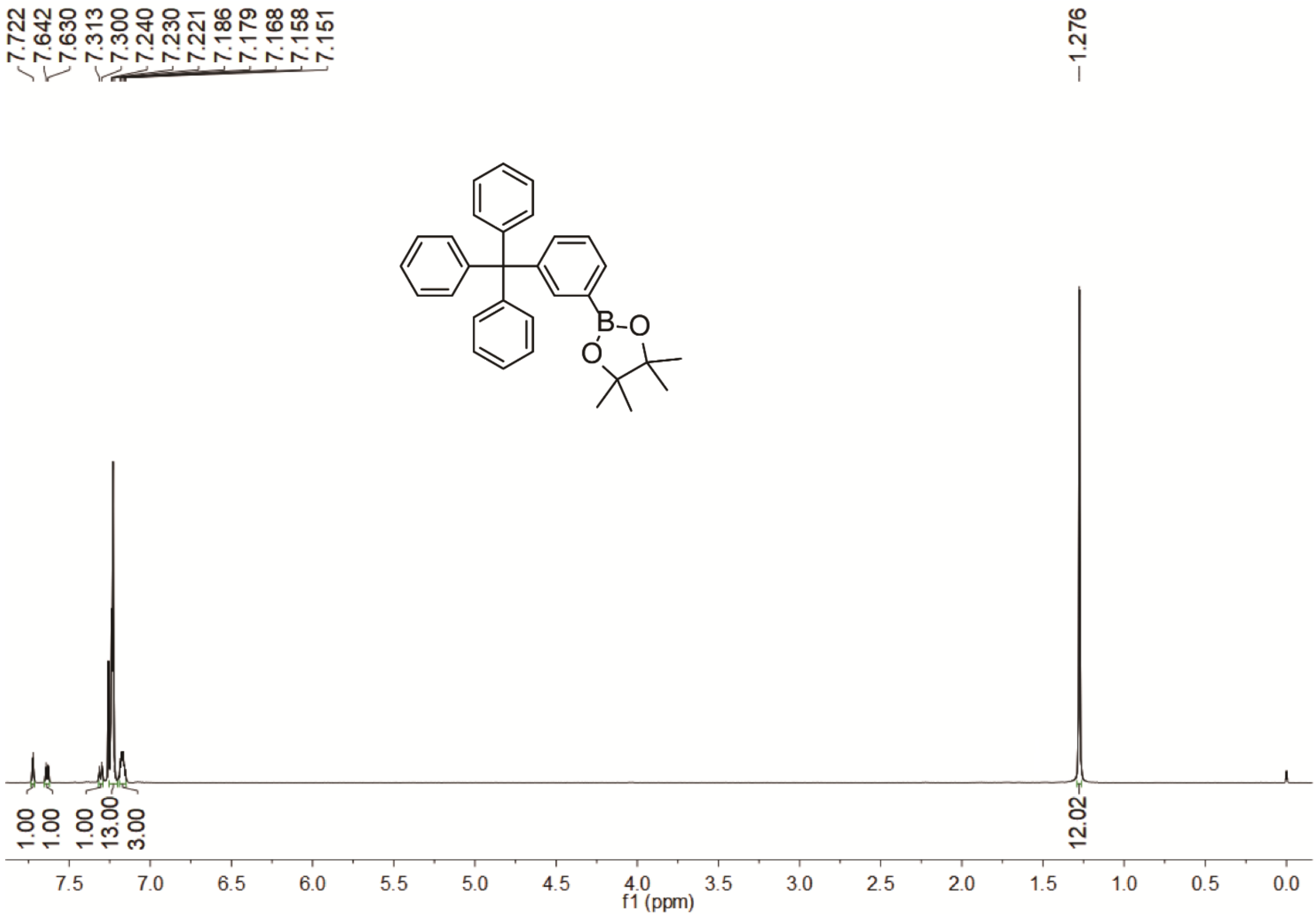

Supplementary Fig. 26. ¹H NMR spectrum of 4,4,5,5-tetramethyl-2-(3-tritylphenyl)-1,3,2-dioxaborolane in $CDCl_3$ at room temperature.

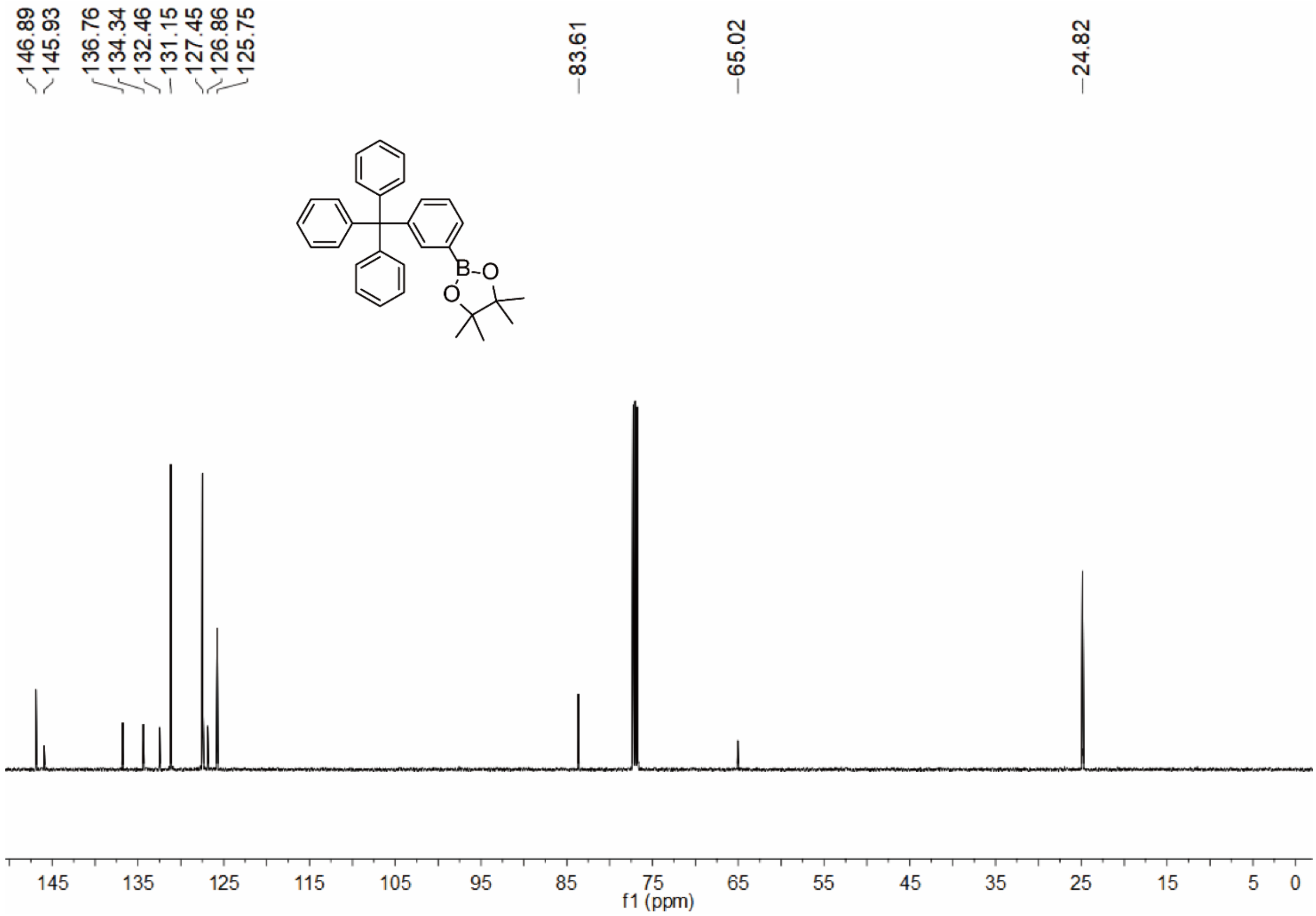

Supplementary Fig. 27. ^{13}C NMR spectrum of 4,4,5,5-tetramethyl-2-(3-tritylphenyl)-1,3,2-dioxaborolane in $CDCl_3$ at room temperature.

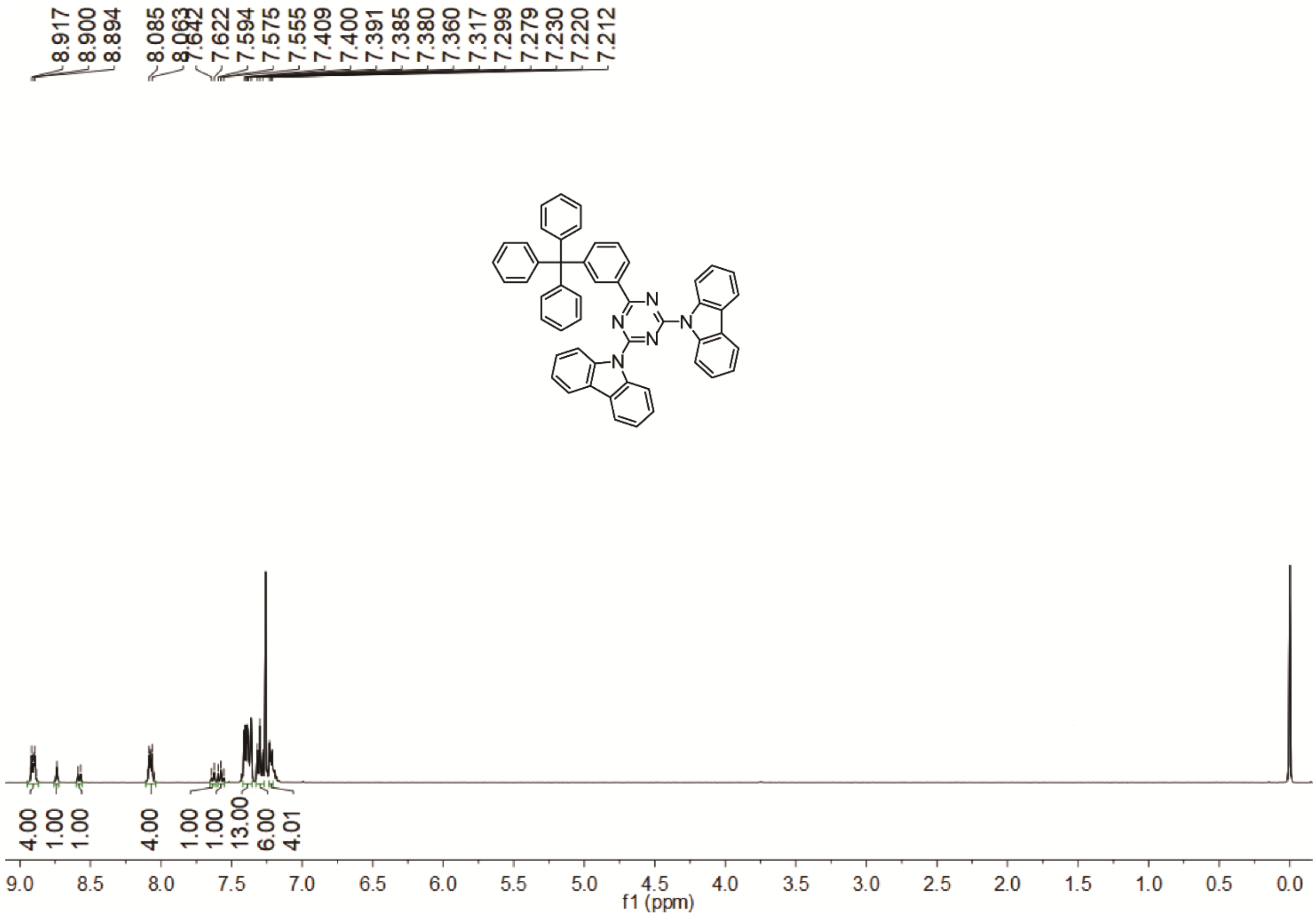

Supplementary Fig. 28. ^{1}H NMR spectrum of CTrzCz2 in $CDCl_3$ at room temperature.

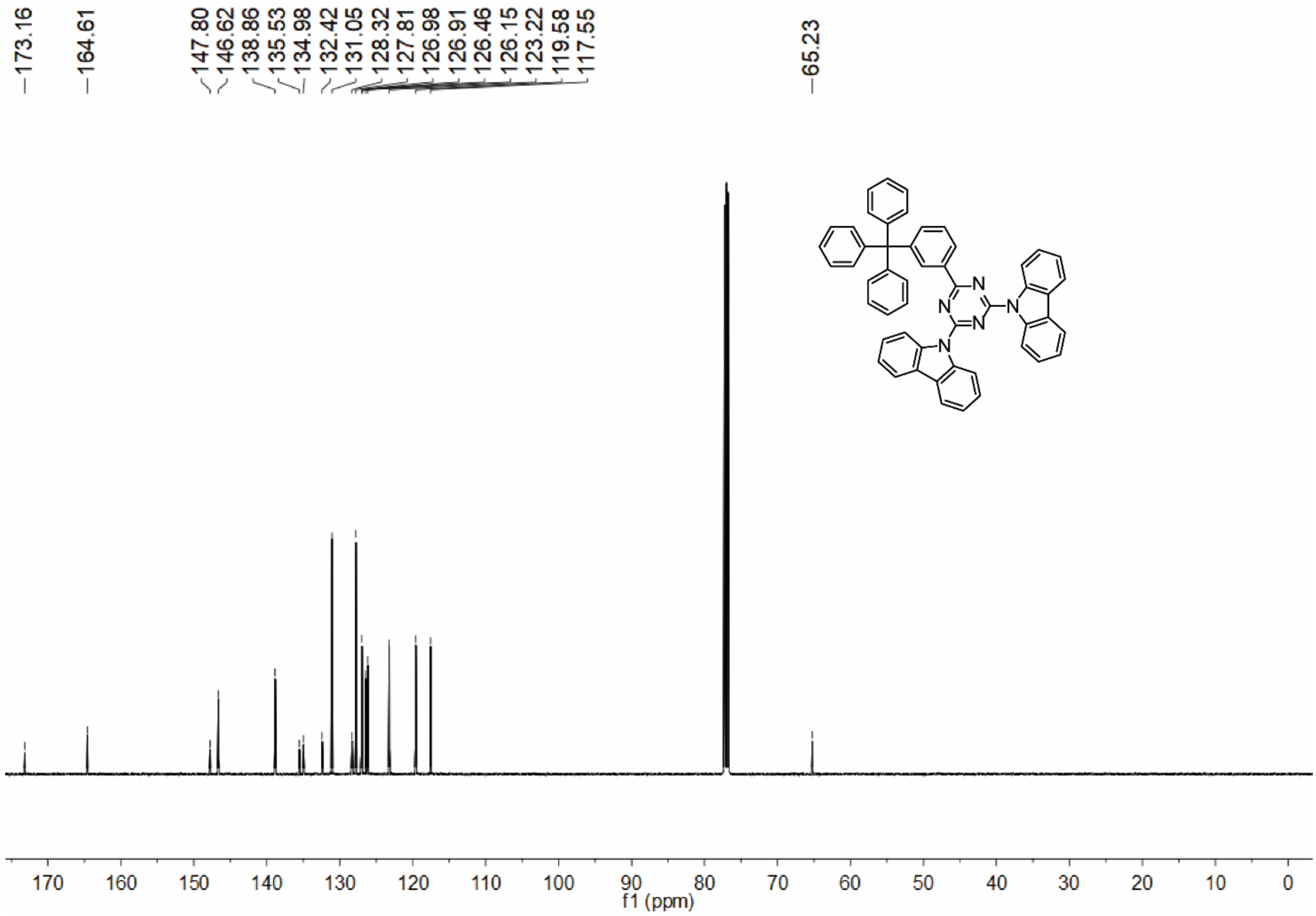

Supplementary Fig. 29. ^{13}C NMR spectrum of CTrzCz2 in $CDCl_3$ at room temperature.

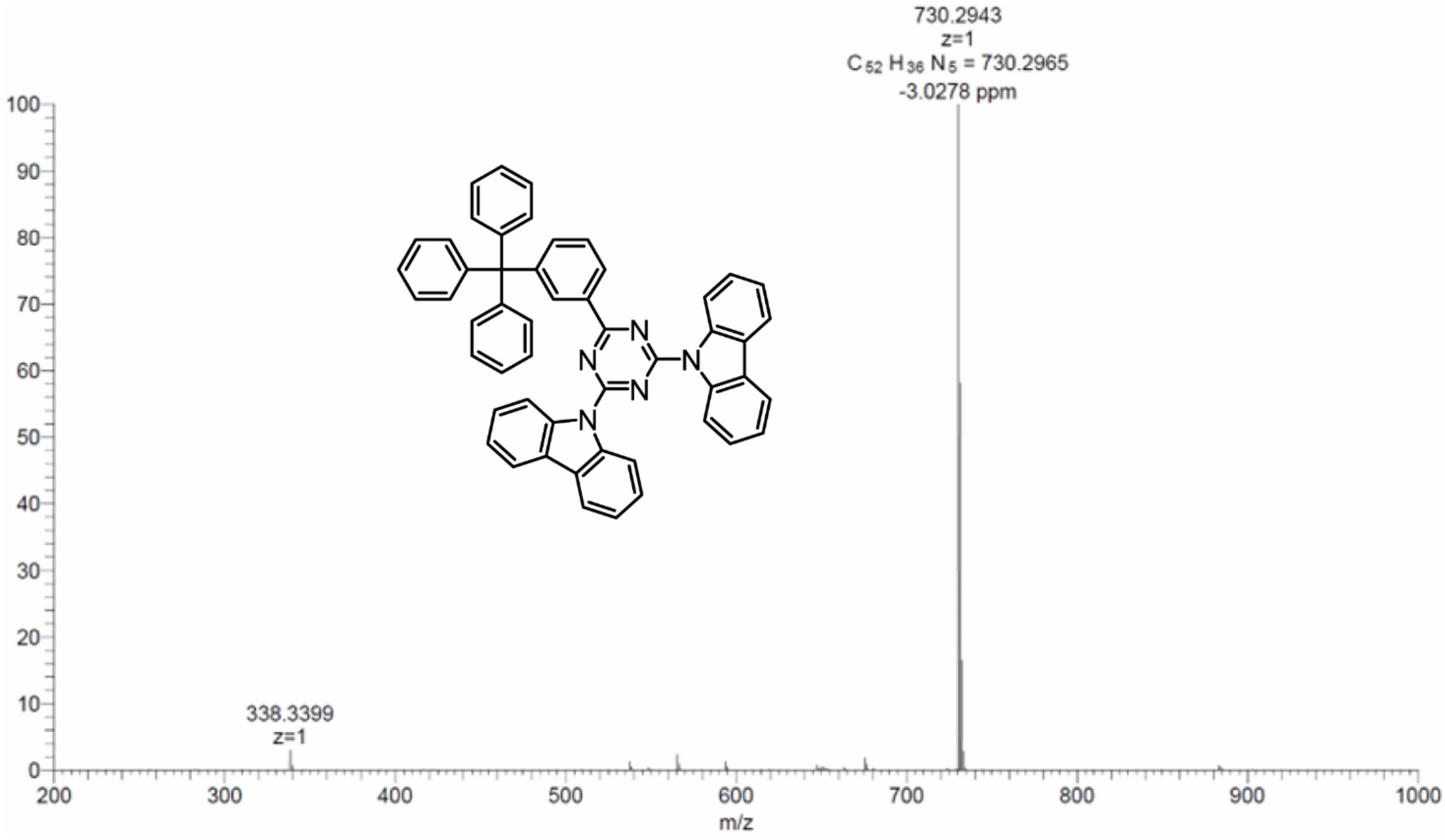

Supplementary Fig. 30. HRMS spectrum of CTrzCz2.

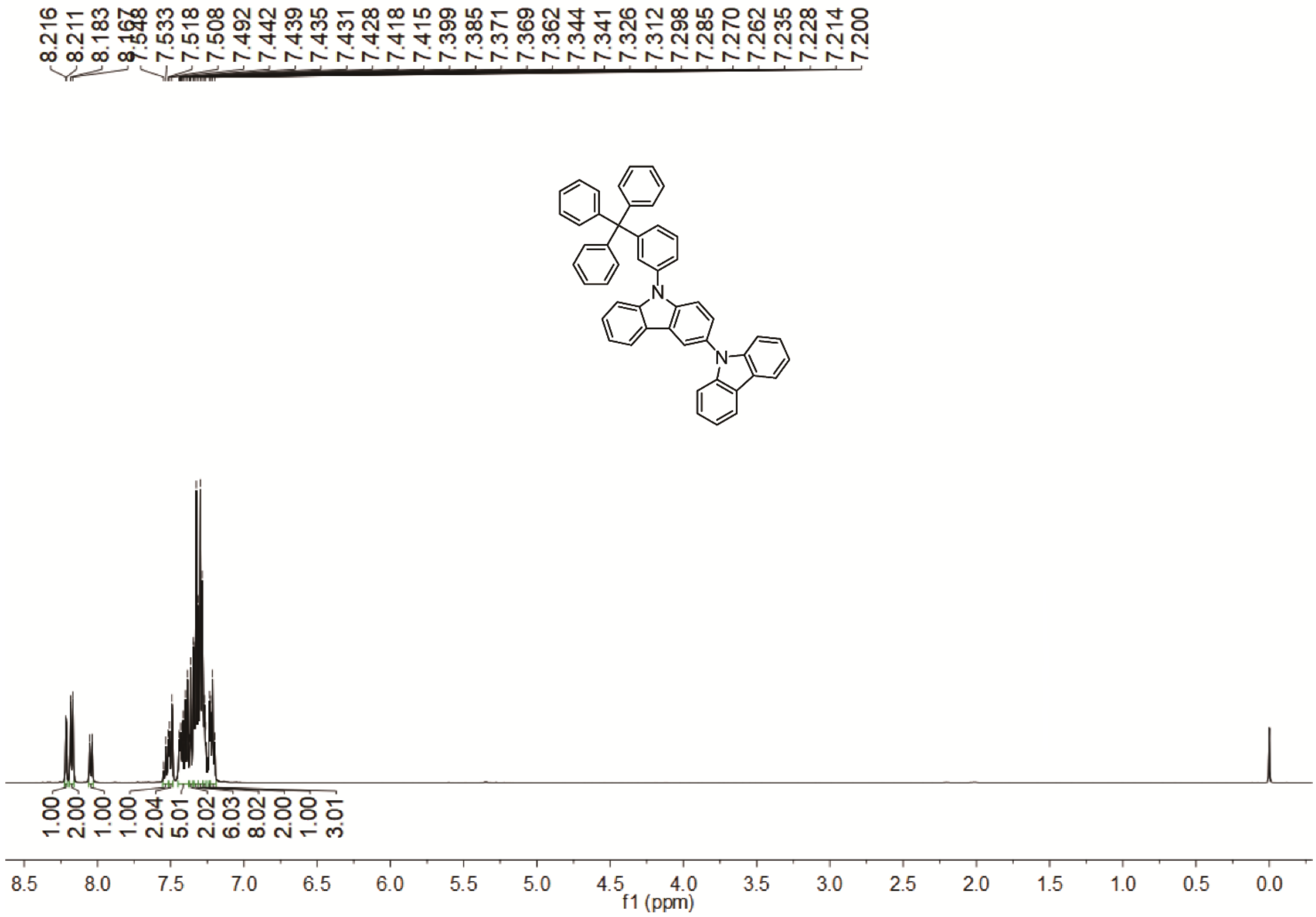

Supplementary Fig. 31. **^{1}H NMR spectrum of CCzCz in $CDCl_3$ at room temperature.**

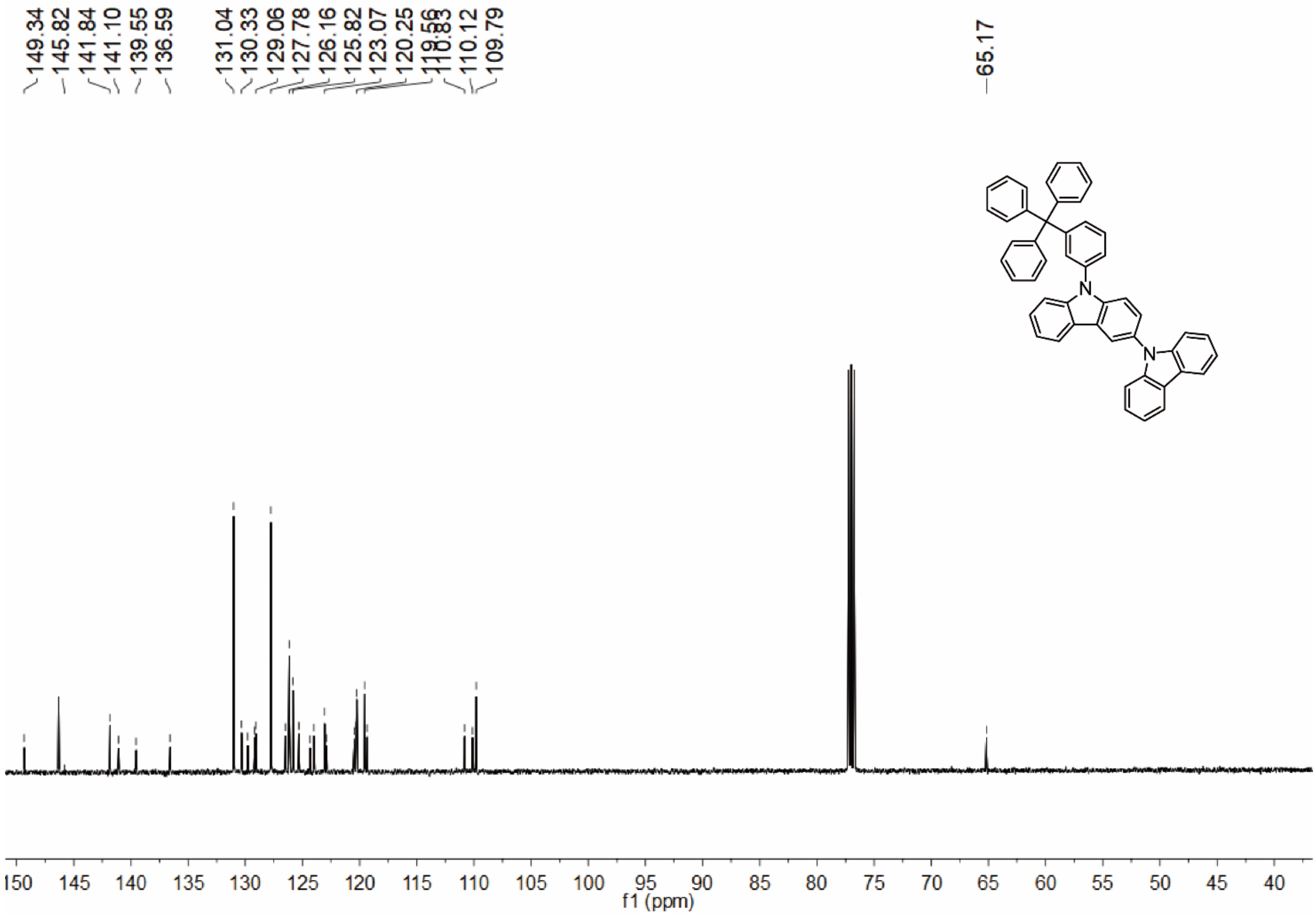

Supplementary Fig. 32. ^{13}C NMR spectrum of CCzCz in $CDCl_3$ at room temperature.

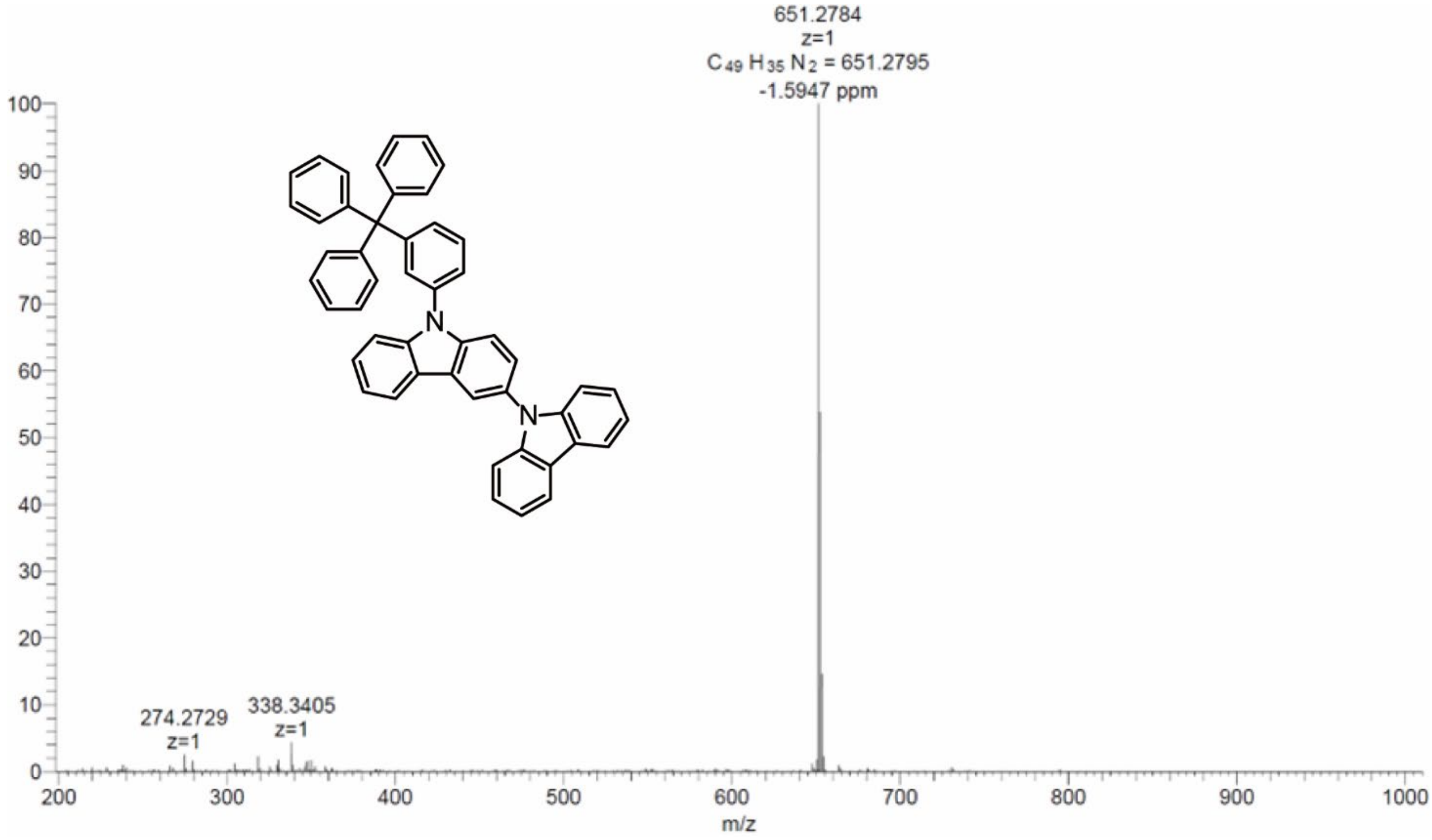

Supplementary Fig. 33. HRMS spectrum of CCzCz.

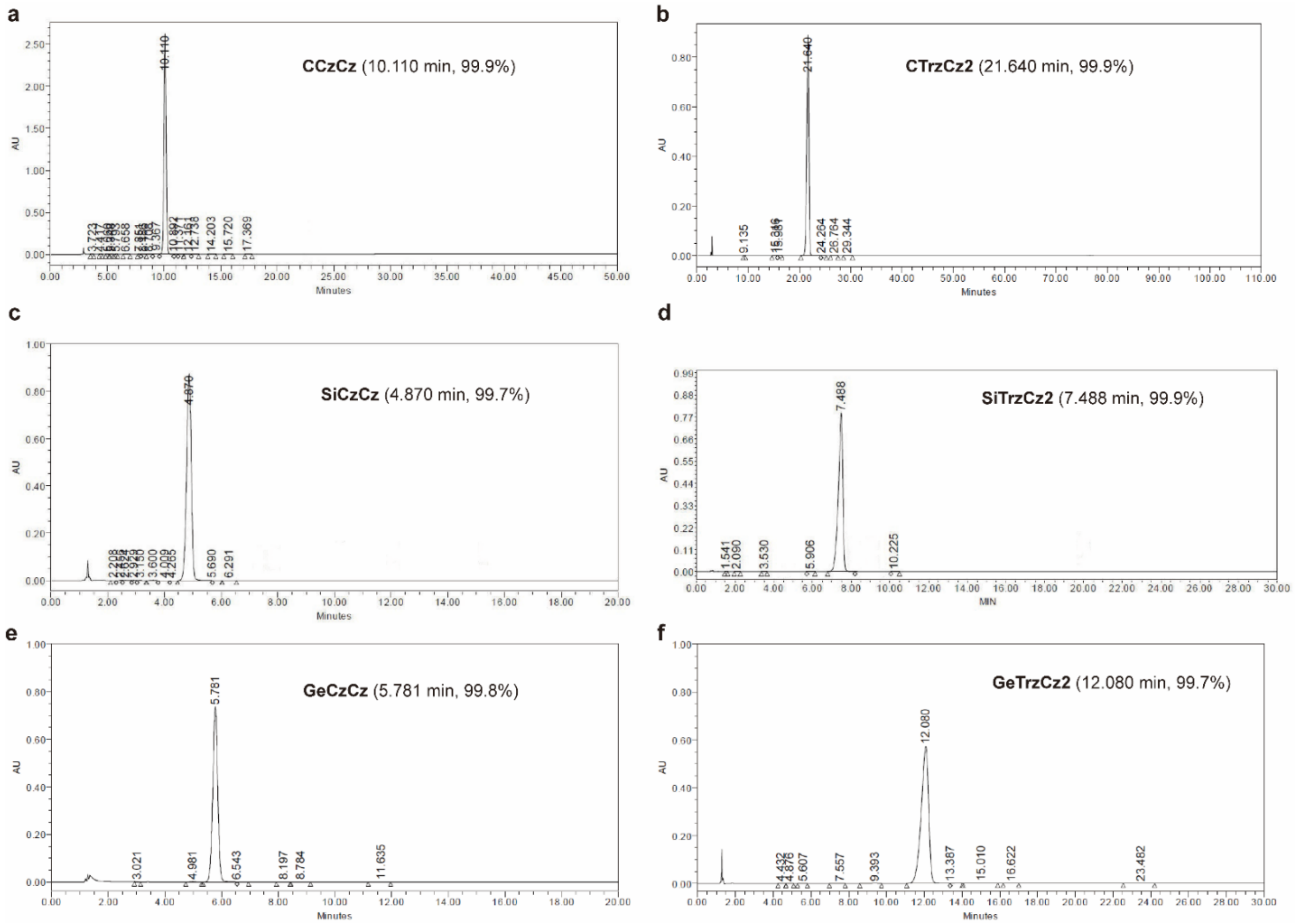

Supplementary Fig. 34. High-performance liquid chromatograms. (**a**) CCzCz. (**b**) CTrzCz2. (**c**) SiCzCz. (**d**) SiTrzCz2. (**e**) GeCzCz. (**f**) GeTrzCz2.

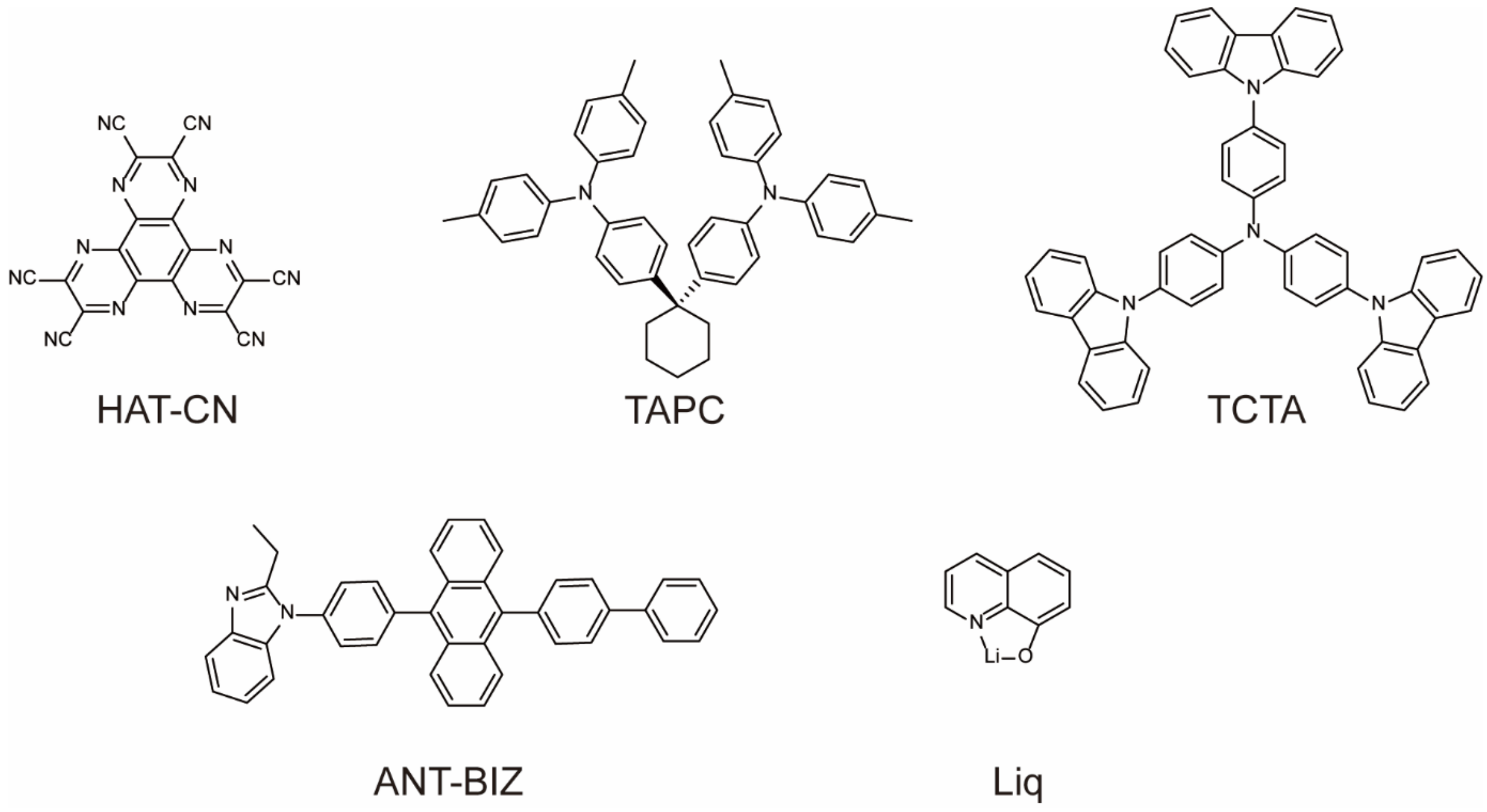

Supplementary Fig. 35. Chemical structures of the comprising materials in OLEDs.

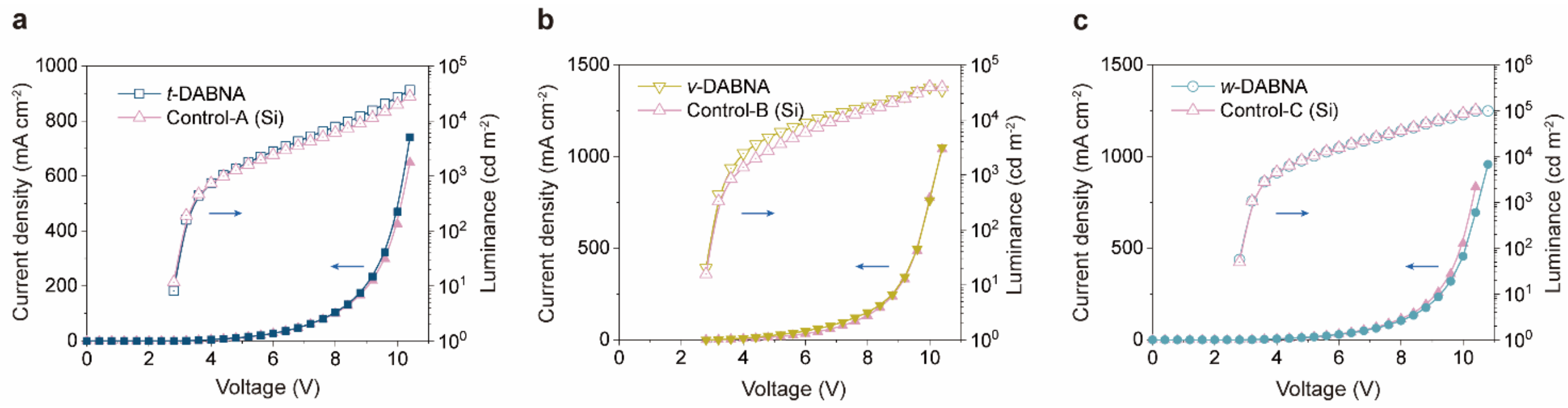

Supplementary Fig. 36. Current density and luminance versus driving voltage curves. (a) *t*-DABNA target device and control-A (Si). (**b**) *υ*-DABNA target device and control-B (Si). (**c**) *ω*-DABNA target device and control-C (Si).

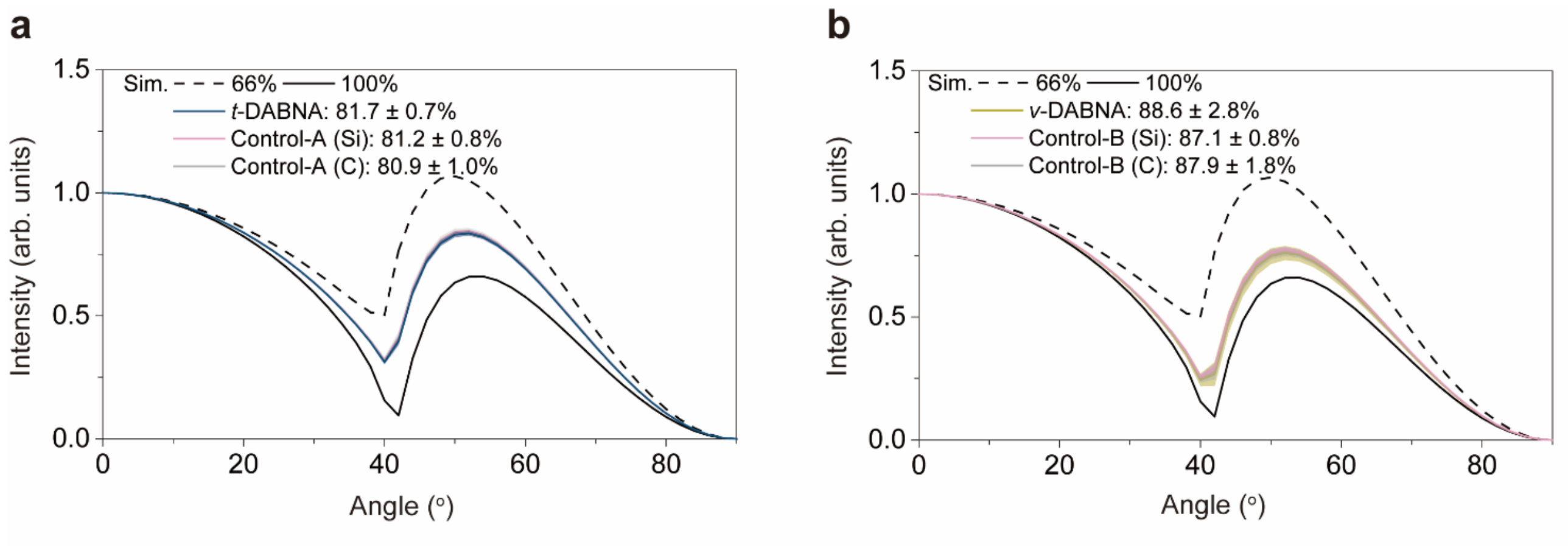

Supplementary Fig. 37. Angle-dependent PL spectra. (**a**) *t*-DABNA-doped films. (**b**) *υ*-DABNA-doped films.

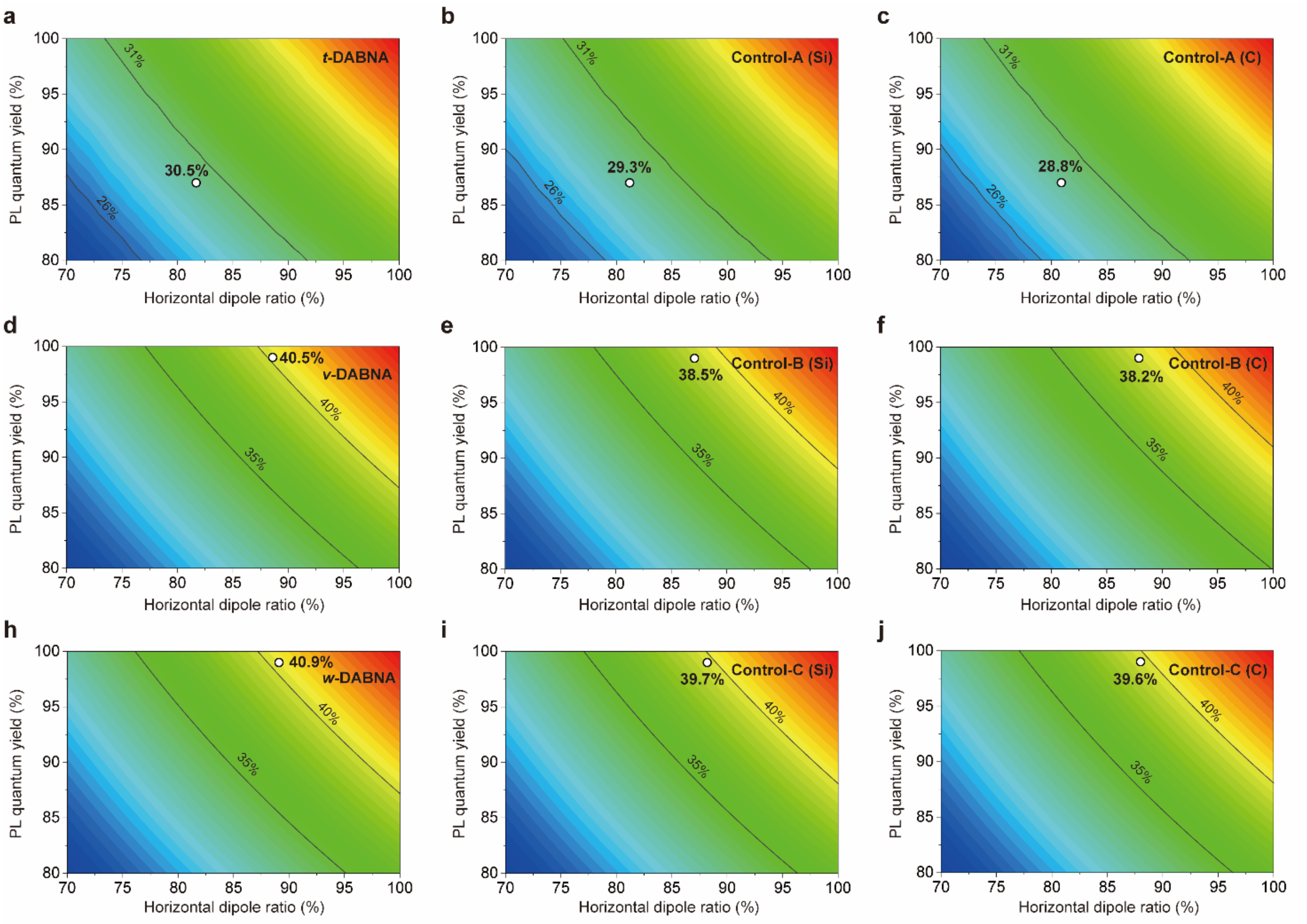

Supplementary Fig. 38. Theoretical maximum external quantum efficiency as a function of the emitting layer PL quantum yield and the horizontal dipole ratio of the emitter for devices. (**a**) *t*-DABNA-based device. (**b**) Control-A (Si) device. (**c**) Control-A (C) device. (**d**) *υ*-DABNA-based device. (**e**) Control-B (Si) device. (**f**) Control-B (C) device. (**h**) *ω*-DABNA-based device. (**i**) Control-C (Si) device. (**j**) Control-C (C) device. The charge balance factor was assumed to be unity.

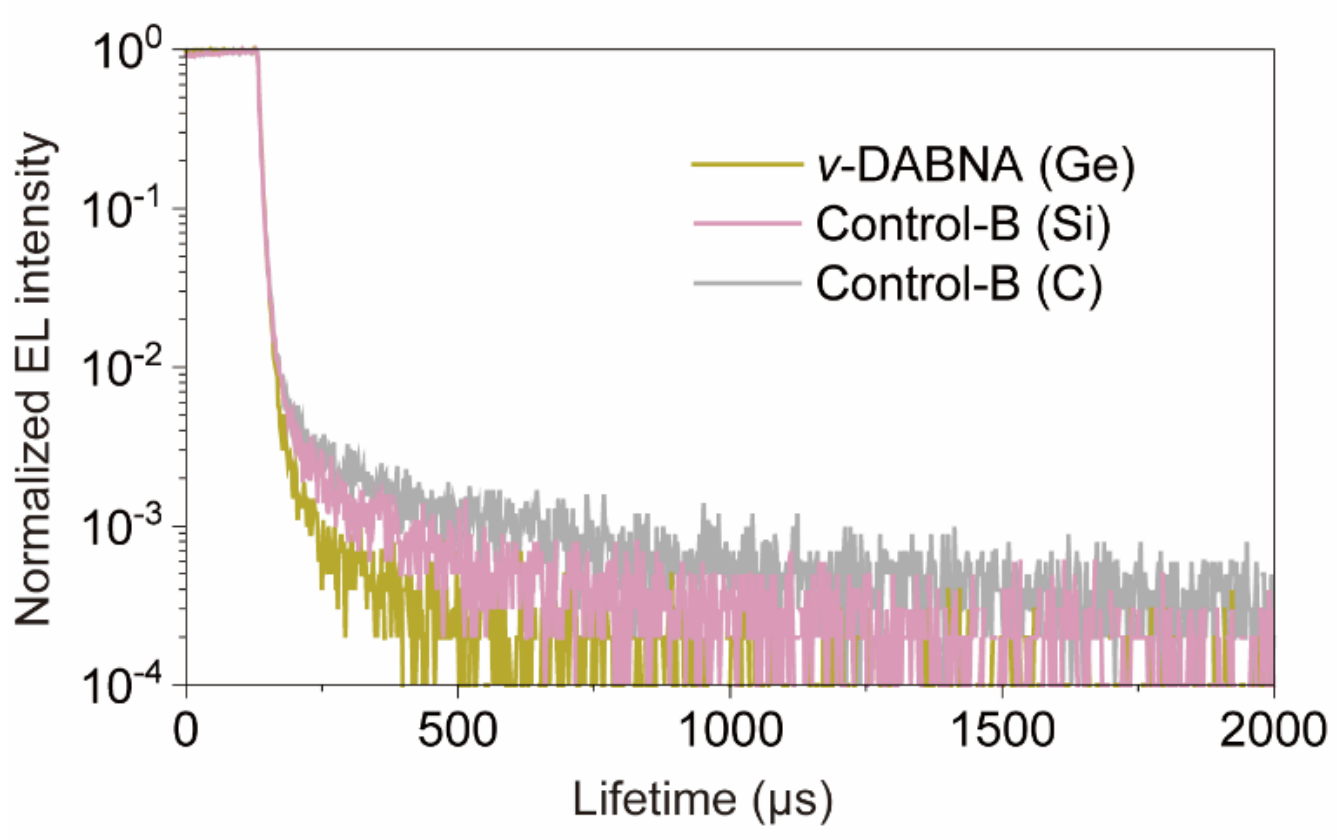

Supplementary Fig. 39. EL transient decay curves of *υ*-DABNA-based device and control devices (control-B), measured at their respective dominant emission peaks.

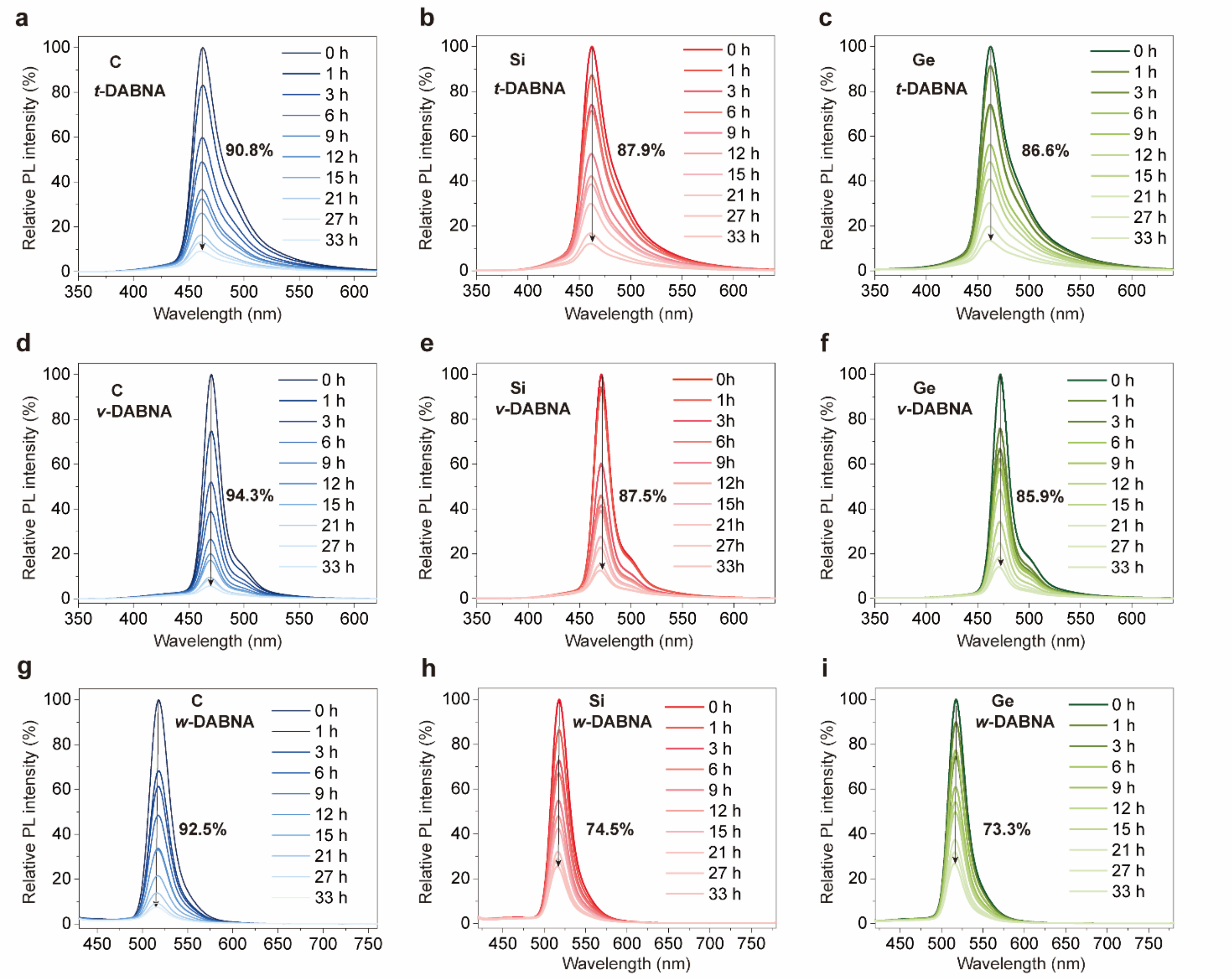

Supplementary Fig. 40. Photolysis results. PL spectra of (**a**) CCzCz:CTrzCz2:*t*-DABNA, (**b**) SiCzCz:SiTrzCz2:*t*-DABNA, (**c**) GeCzCz:GeTrzCz2:*t*-DABNA, (**d**) CCzCz:CTrzCz2:*υ*-DABNA, (**e**) SiCzCz:SiTrzCz2:*υ*-DABNA, (**f**) GeCzCz:GeTrzCz2:*υ*-DABNA, (**g**) CCzCz:CTrzCz2:*ω*-DABNA, (**h**) SiCzCz:SiTrzCz2:*ω*-DABNA, (**i**) GeCzCz:GeTrzCz2:*ω*-DABNA over 33 h under continuous 365 nm LED irradiation (12 W) at room temperature.

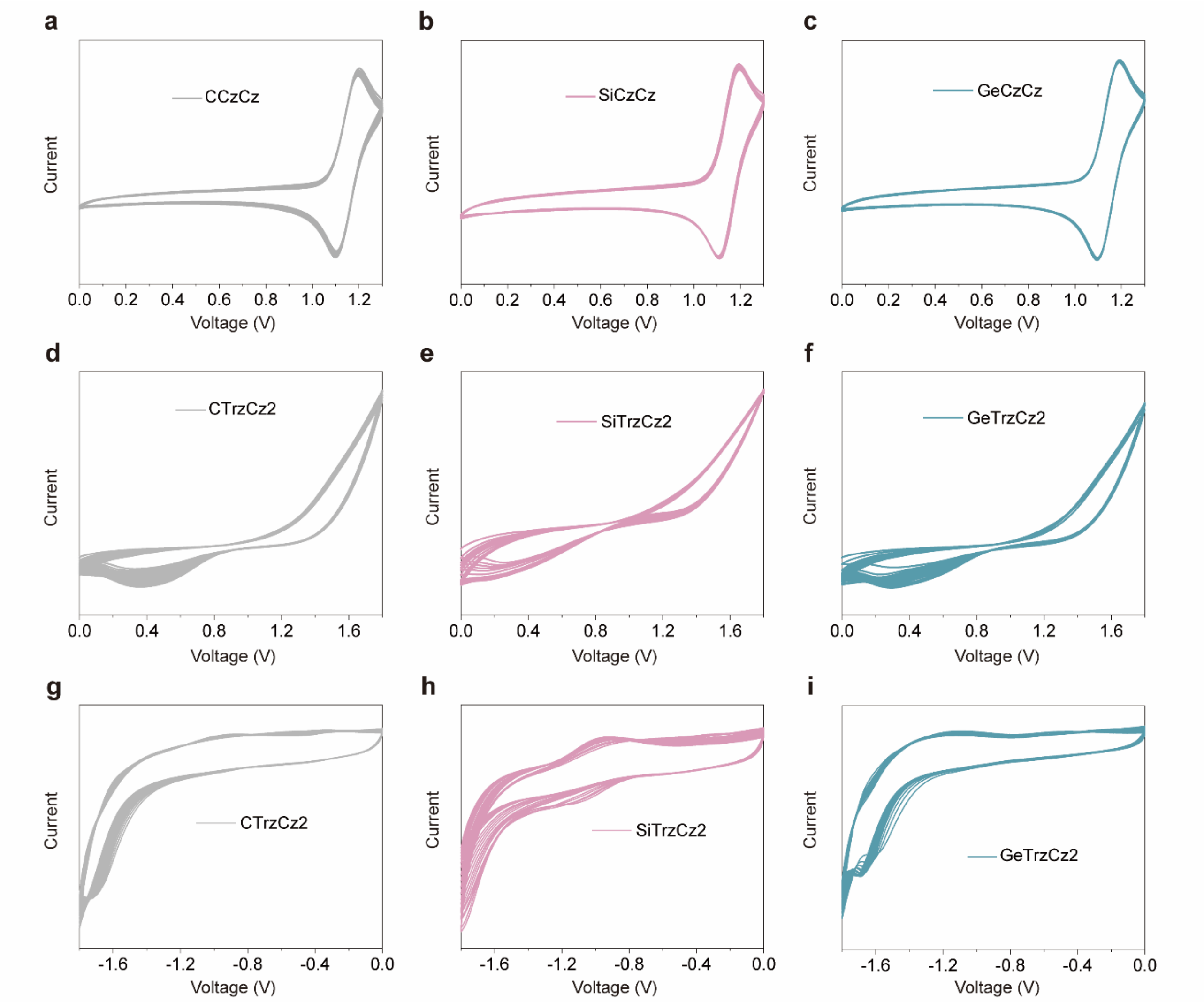

Supplementary Fig. 41. Cyclic voltammograms of the hosts. Oxidation waves of (**a**) CCzCz, (**b**) SiCzCz, (**c**) GeCzCz, (**d**) CTrzCz2, (**e**) SiTrzCz2, (**f**) GeTrzCz2 and reduction waves of (**g**) CTrzCz2, (**h**) SiTrzCz2, (**i**) GeTrzCz2.

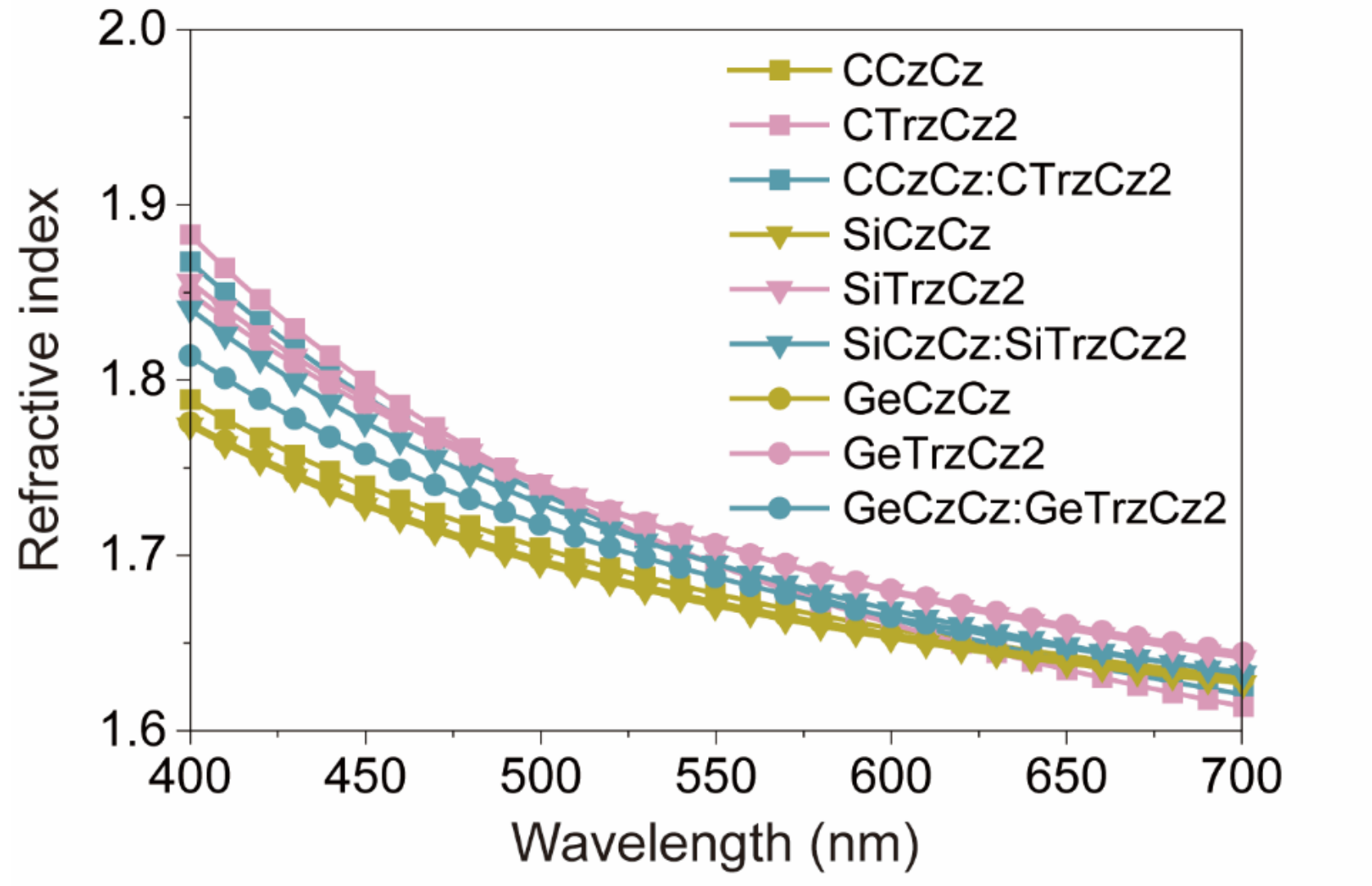

Supplementary Fig. 42. Refractive indices of the charge blocking layers and emitting layers.

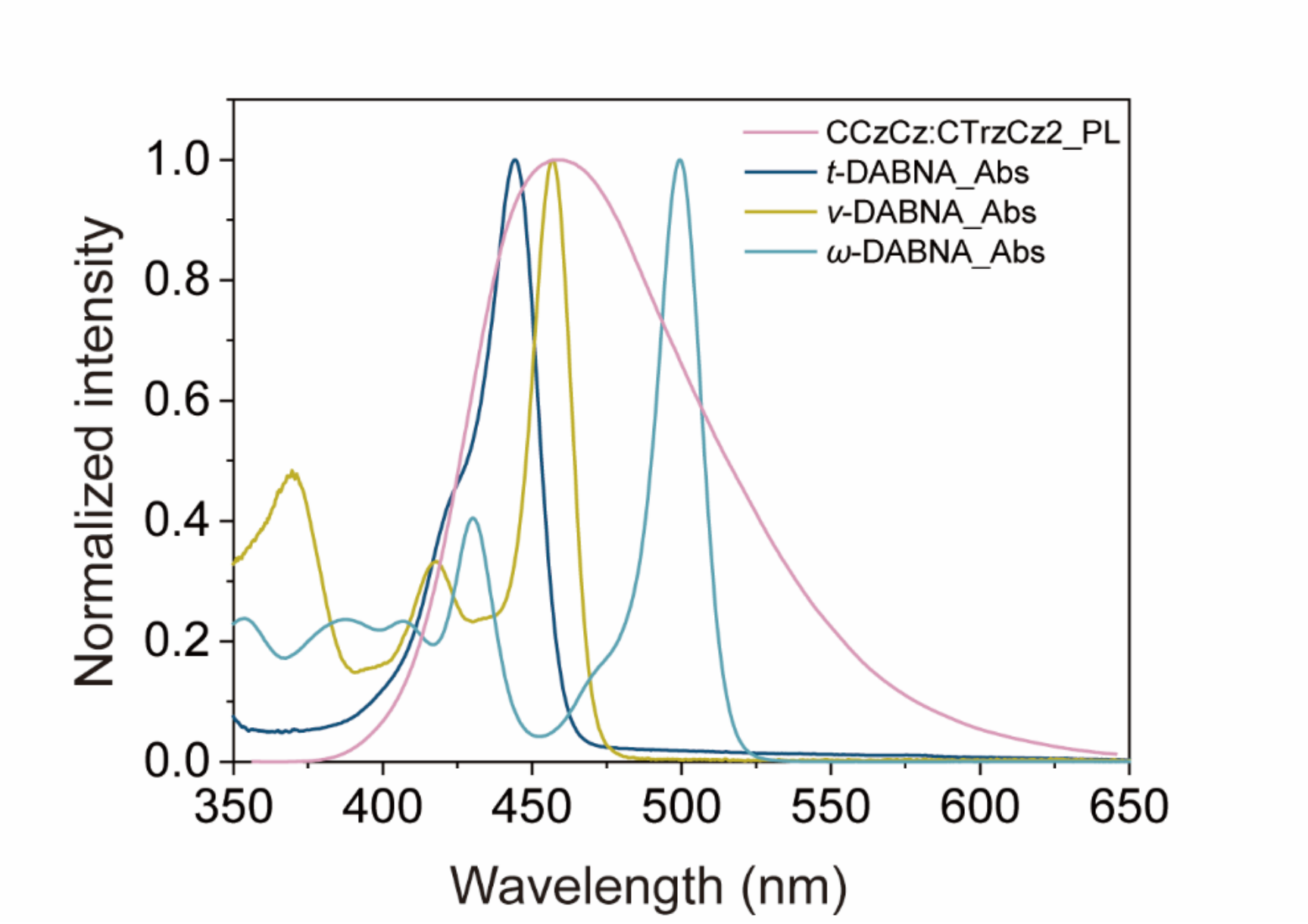

Supplementary Fig. 43. Normalized PL spectra of CCzCz:CTrzCz2 exciplex film and absorption spectrum of *t*-DABNA, *υ*-DABNA and *ω*-DABNA in toluene (10^{-5} mol·L^{-1}).

Based on spectral overlap between absorption spectrum of the emitter and PL spectra of the CCzCz:CTrzCz2 exciplex host, the Förster radius for *t*-DABNA, *υ*-DABNA, and *ω*-DABNA were calculated as 4.7 nm, 7.6 nm, and 8.3 nm, respectively.

Supplementary Tables

Supplementary Table 1. Atomic dipole moment corrected Hirshfeld atomic charges for the individual atoms in the Ge- and Si-based hosts. The atomic numbering corresponds to the labeled positions in the depicted chemical structures.

Atoms	Number	GeTrzCz2	SiTrzCz2	Atoms	Number	GeCzCz	SiCzCz
**Ge/Si**	**-**	**0.43838**	**0.37829**	**Ge/Si**	**-**	**0.66454**	**0.52894**
C	1	-0.29945	-0.19723	C	1	-0.16306	-0.15245
C	2	-0.02483	-0.03618	C	2	-0.10109	-0.11121
C	3	-0.08559	-0.10089	C	3	0.08511	0.08364
C	4	-0.08374	-0.07590	C	4	-0.06485	-0.05969
C	5	-0.08769	-0.09131	C	5	-0.05575	-0.02871
C	6	-0.05763	-0.08075	C	6	-0.16554	-0.16555
C	7	-0.12927	-0.15235	C	7	-0.30057	-0.23181
C	8	-0.11387	-0.03838	C	8	-0.03698	-0.06403
C	9	-0.11978	-0.11455	C	9	-0.10472	-0.10549
C	10	-0.12408	-0.12990	C	10	-0.15041	-0.13040
C	11	-0.11408	-0.11468	C	11	-0.09899	-0.09731
C	12	-0.11153	-0.11322	C	12	-0.00305	-0.05434
C	13	-0.05123	-0.09434	C	13	-0.21336	-0.18135
C	14	-0.14805	-0.07453	C	14	-0.07680	-0.08553
C	15	-0.04061	-0.07206	C	15	-0.11212	-0.10769
C	16	-0.44770	-0.40916	C	16	-0.13736	-0.12418
C	17	-0.22673	-0.22549	C	17	-0.10886	-0.10915
C	18	-0.02867	0.0107	C	18	-0.08563	-0.09028
C	19	-0.15636	-0.14701	C	19	-0.34926	-0.27365
C	20	-0.05963	-0.07486	C	20	0.11063	0.01359
C	21	-0.11924	-0.13317	C	21	-0.09372	-0.07644
C	22	-0.14484	-0.15146	C	22	-0.15539	-0.12017
C	23	-0.11581	-0.01936	C	23	-0.09295	-0.16648
C	24	-0.00677	-0.04181	C	24	-0.07343	-0.07047

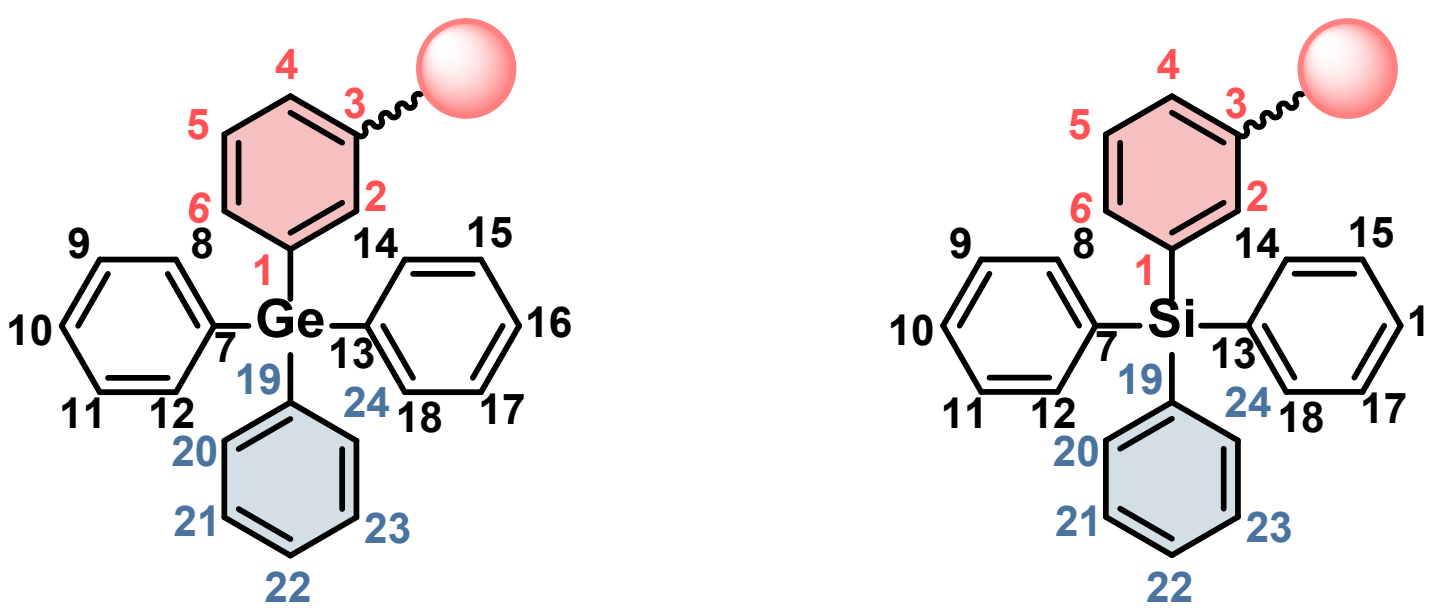

The atomic dipole moment corrected Hirshfeld atomic charge analysis reveals that the Ge atom is more positively charged than the Si atom, which polarizes the surrounding molecular groups around Ge relatively more electron-rich. Since London dispersion forces operate between induced dipoles, these electron-rich groups strengthen the intermolecular London dispersion interactions.

Supplementary Table 2. Physical data and kinetic parameters of GeCzCz:GeTrzCz2 and SiCzCz:SiTrzCz2 exciplex films.

Film	λ_{PL} / nm	PLQY / %	τ_p / ns	τ_d / µs	$k_{r,S}$ / × 10^6 s^{-1}	k_{RISC} / × 10^5 s^{-1}	$k_{nr,S}$ / × 10^6 s^{-1}
**GeCzCz:GeTrzCz2**	470	54	173.0	1.6	2.5	8.0	2.1
**SiCzCz:SiTrzCz2**	470	30	186.8	1.9	1.3	6.3	3.1

Supplementary Table 3. Calculated Huang-Rhys factors (*S*) for the dominant skeletal vibrational modes (where *S* > 0.06) of SiTrzCz2 and GeTrzCz2.

SiTrzCz2		GeTrzCz2	
**Vibrational frequency / cm^{-1}**	**Huang-Rhys factor**	**Vibrational frequency / cm^{-1}**	**Huang-Rhys factor**
278.90	0.26444	278.44	0.26717
444.25	0.07025	444.11	0.06345
1139.58	0.12733	1140.32	0.07084
1146.80	0.09338	1146.23	0.06557
1177.92	0.08535	1173.02	0.08032
1240.36	0.07541	1240.40	0.07988
1379.29	0.19652	1379.17	0.19316
1417.93	0.11157	1417.97	0.11139
1703.76	0.15470	1703.85	0.15454
1711.48	0.12094	1711.68	0.11559
1725.06	0.27407	1725.34	0.28561

Supplementary Table 4. Physical data and kinetic parameters of MR-TADF emitters *t*-DABNA, *υ*-DABNA and *ω*-DABNA doped in exciplex hosts GeCzCz:GeTrzCz2 and SiCzCz:SiTrzCz2.

Film	τ_p / ns	τ_d / µs	k_{RISC} / s^{-1}	k_{DET} / s^{-1}
**GeCzCz:GeTrzCz2:*t*-DABNA**	10.7	112.3	2.2×10^4	3.3×10^6
**SiCzCz:SiTrzCz2:*t*-DABNA**	11.9	133.0	1.3×10^4	4.4×10^6
**GeCzCz:GeTrzCz2:*υ*-DABNA**	5.3	2.4	9.1×10^5	3.3×10^6
**SiCzCz:SiTrzCz2:*υ*-DABNA**	5.5	4.0	4.5×10^5	5.6×10^6
**GeCzCz:GeTrzCz2: *ω*-DABNA**	6.3	3.6	7.5×10^5	1.9×10^6
**SiCzCz:SiTrzCz2:*ω*-DABNA**	6.4	7.7	3.6×10^5	5.8×10^6

Supplementary Table 5. Summary of recent advances in green-emitting OLEDs with $CIE_y \geq$ 0.71 (NTSC standard) and blue-emitting OLEDs with $CIE_y \leq$ 0.20.

Emitting materials	EQE / %		CIE_y	Ref.
	Max	1,000 cd·m^{-2}		
***ω*-DABNA**	**40.4**	**33.4**	**0.74**	**This work**
MCNBN	30.8	20.4	0.74	1
PMCNBN	27.2	20.3	0.73	1
TCz-B	29.2	9.4	0.71	2
TCz-VTCzBN	32.2	16.0	0.71	3
BN-ICz-1	30.5	17.2	0.74	4
BN-ICz-2	29.8	26.1	0.73	4
*t*CzphB-Ph	29.3	~10	0.75	5
*t*CzphB-Fl	26.2	~9	0.72	5
ω-DABNA (single host)	31.1	29.4	0.73	6
ω-DABNA-M	32.7	27.4	0.74	7
ω-DABNA-PH	31.8	27.4	0.74	7
DBNT-2	35.2	20.4	0.74	8
BN-TP-N3	37.3	19.8	0.71	9
QB-PXZ	36.6	31.8	0.74	10
***υ*-DABNA**	**40.1**	**32.8**	**0.12**	**This work**
υ-DABNA(single host)	34.4	26.0	0.11	11
υ-DABNA(TSF)	29.2	24.1	0.14	12
t-BuCz-DABNA(TSF)	30.8	26.2	0.17	13

υ-DABNA(TSF)	27	20	0.20	14
υ-DABNA(TSF)	33.0	25.2	0.17	15
BD-02(phos.)	25.4	23.4	0.197	16
TBE02(PSF)	25.8	~22	0.165	17
PtON-TTBI (phos.)	25.9	24.6	0.19	18
PtON7-dtb (phos.)	24.8	11.0	0.079	19
PtON-CF_3-2 (phos.)	33.8	31.6	0.15	20
f-cf6b (phos.)	23.4	17.1	0.12	21
v-DABNA (PSF)	26.2	18.4	0.13	21

Supplementary Table 6. Silicon- and germanium-related bond dissociation energies (eV) in the lowest triplet state. The bonds involving silicon or germanium atoms are highlighted in number for each molecular structure.

Bond	GeCzCz	SiCzCz	GeTrzCz2	SiTrzCz2
1	3.91	3.93	1.71	1.75
2	4.57	4.69	3.27	3.36
3	4.80	4.81	1.94	2.00
4	4.80	4.83	2.49	2.42

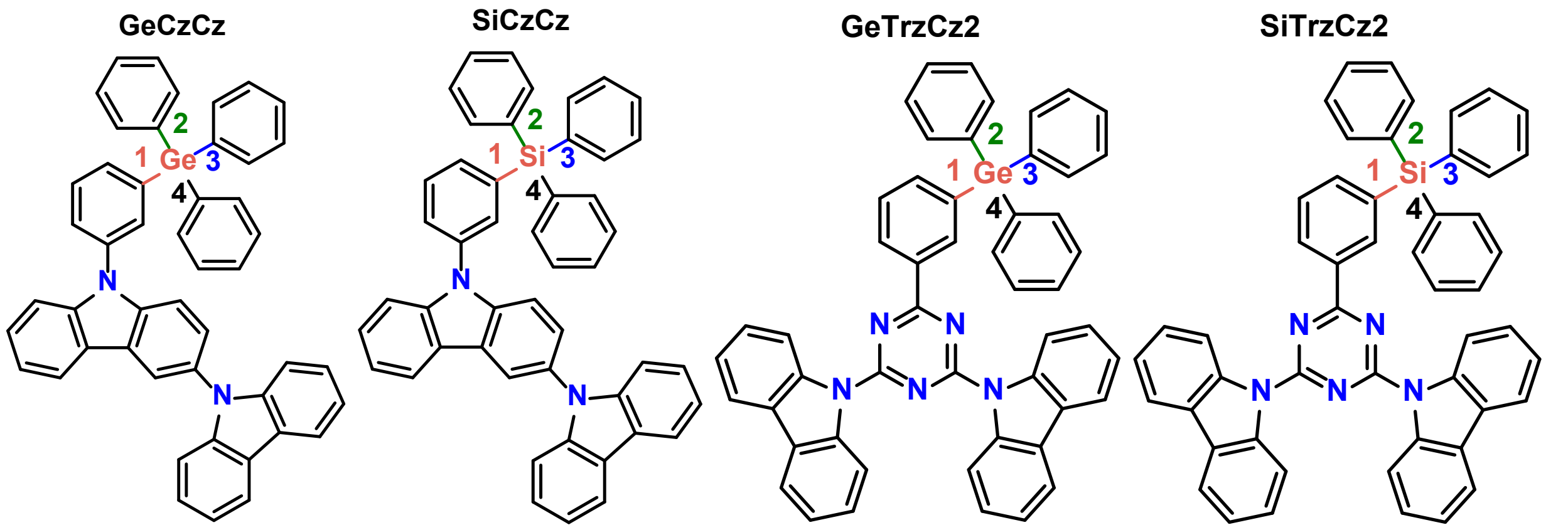

Supplementary Table 7. Computational results for the CCzCz:CTrzCz2 exciplex. Calculated spin-orbit coupling matrix elements between S_1 and T_1, and between S_1 and T_2, for 16 representative conformers. The relative energy (ΔE) and the corresponding Boltzmann factor ($e^{-\Delta E/RT}$), alongside the structural deviation relative to the lowest-energy conformer expressed as root-mean-square deviations (RMSD), are also listed. The mean values, standard deviation (S.D.), and relative standard deviation (RSD) are provided for statistical analysis.

**CCzCz:CTrzCz2**	**$\langle S_1\|\hat{H}_{SOC}\|T_1\rangle$ / cm^{-1}**	**$\langle S_1\|\hat{H}_{SOC}\|T_2\rangle$ / cm^{-1}**	**ΔE / $kcal{\cdot}mol^{-1}$**	**$e^{-\Delta E/RT}$**	***RMSD* / Å**
1	0.16874	0.03390	0.00000	1	0.000
2	0.09916	0.02220	4.00269	0.001158994	4.910
3	0.11068	0.04395	4.49955	0.000500771	5.966
4	0.05297	0.01277	9.22839	1.70254×10^{-7}	4.844
5	0.08648	0.02692	10.20627	3.26464×10^{-8}	5.959
6	0.00642	0.05411	9.28205	1.55503×10^{-7}	6.144
7	0.04313	0.00174	9.57875	9.42137×10^{-8}	6.710
8	0.08245	0.03540	14.61066	1.9199×10^{-11}	6.825
9	0.01040	0.01721	21.93126	8.19815×10^{-17}	6.358
10	0.00067	0.00061	18.00162	6.25244×10^{-14}	7.220
11	0.00362	0.00117	7.93451	1.51405×10^{-6}	5.590
12	0.01197	0.00795	33.85830	1.46347×10^{-25}	6.327
13	0.00493	0.01219	25.67383	1.47431×10^{-19}	6.088
14	0.09815	0.01090	12.21624	1.09535×10^{-9}	5.480
15	0.08060	0.01023	5.55057	8.48651×10^{-5}	5.069
16	0.00234	0.02116	6.95982	7.85352×10^{-6}	7.004
**Mean**	0.05392	0.01953	-	-	-
**S.D.**	0.04965	0.01526	-	-	-
**RSD**	92.08%	78.14%	-	-	-

Supplementary Table 8. Computational results for the SiCzCz:SiTrzCz2 exciplex. Calculated spin-orbit coupling matrix elements between S_1 and T_1, and between S_1 and T_2, for 16 representative conformers. The relative energy (ΔE) and the corresponding Boltzmann factor ($e^{-\Delta E/RT}$), alongside the structural deviation relative to the lowest-energy conformer expressed as root-mean-square deviations (RMSD), are also listed. The mean values, standard deviation (S.D.), and relative standard deviation (RSD) are provided for statistical analysis.

**SiCzCz:SiTrzCz2**	**$\langle S_1\|\hat{H}_{SOC}\|T_1\rangle$ / cm^{-1}**	**$\langle S_1\|\hat{H}_{SOC}\|T_2\rangle$ / cm^{-1}**	**ΔE / kcal·mol^{-1}**	**$e^{-\Delta E/RT}$**	***RMSD* / Å**
1	0.26543	0.08124	0.00000	1	0
2	0.08236	0.01613	5.92511	4.50829×10^{-5}	4.830
3	0.12803	0.03323	6.92196	8.37206×10^{-6}	6.112
4	0.04685	0.01711	12.47811	7.0384×10^{-10}	4.881
5	0.08415	0.04372	14.09930	4.55358×10^{-11}	6.561
6	0.00518	0.05929	12.74074	4.51687×10^{-10}	6.780
7	0.04150	0.00879	10.53546	1.87231×10^{-8}	6.871
8	0.11208	0.04238	16.95826	3.642×10^{-13}	6.807
9	0.01515	0.02341	23.52659	5.54061×10^{-18}	6.885
10	0.00392	0.00032	21.82230	9.85455×10^{-17}	7.752
11	0.00950	0.00061	13.63273	1.00133×10^{-10}	6.333
12	0.00298	0.01499	37.06810	6.47209×10^{-28}	6.704
13	0.00940	0.01114	34.23180	7.78803×10^{-26}	6.747
14	0.11374	0.02210	14.75718	1.49902×10^{-11}	5.592
15	0.11571	0.03940	8.34455	7.57507×10^{-7}	5.182
16	0.00563	0.02327	6.58050	1.49035×10^{-5}	7.518
**Mean**	0.06507	0.02724	-	-	-
**S.D.**	0.06893	0.02086	-	-	-
**RSD**	105.93%	76.58%	-	-	-

Supplementary Table 9. Computational results for the GeCzCz:GeTrzCz2 exciplex. Calculated spin-orbit coupling matrix elements between S_1 and T_1, and between S_1 and T_2, for 16 representative conformers. The relative energy (ΔE) and the corresponding Boltzmann factor ($e^{-\Delta E/RT}$), alongside the structural deviation relative to the lowest-energy conformer expressed as root-mean-square deviations (RMSD), are also listed. The mean values, standard deviation (S.D.), and relative standard deviation (RSD) are provided for statistical analysis.

**GeCzCz:GeTrzCz2**	**$\langle S_1\|\hat{H}_{SOC}\|T_1\rangle$ / cm^{-1}**	**$\langle S_1\|\hat{H}_{SOC}\|T_2\rangle$ / cm^{-1}**	**ΔE / kcal·mol^{-1}**	**$e^{-\Delta E/RT}$**	***RMSD* / Å**
1	0.79143	0.17433	0.00000	1	0.000
2	0.16029	0.10675	5.17048	0.000161259	5.524
3	0.19178	0.07142	6.19551	2.85544×10^{-5}	6.132
4	0.05882	0.21231	11.36845	4.58556×10^{-9}	5.926
5	0.09562	0.16273	13.75949	8.08348×10^{-11}	5.355
6	0.00980	0.48516	11.57260	3.24827×10^{-9}	6.276
7	0.07429	0.05909	9.46046	1.15048×10^{-7}	5.515
8	1.22599	0.25092	16.26596	1.17256×10^{-12}	6.873
9	0.04438	0.07668	21.88645	8.84258×10^{-17}	5.773
10	0.02876	0.00193	20.54772	8.4824×10^{-16}	7.616
11	0.05288	0.00342	12.46821	7.15702×10^{-10}	6.653
12	0.04708	0.05181	36.48192	1.74181×10^{-27}	8.140
13	0.05117	0.05680	33.67265	2.00244×10^{-25}	8.871
14	0.12324	0.06548	14.30816	3.20005×10^{-11}	6.705
15	0.42973	0.15214	7.26573	4.68476×10^{-6}	6.475
16	0.01486	0.06466	5.03800	0.000201695	6.048
**Mean**	0.21248	0.12471	-	-	-
**S.D.**	0.32573	0.11585	-	-	-
**RSD**	153.30%	92.90%	-	-	-

Supplementary Table 10. Atomic coordinates of CCzCz:CTrzCz2 (01, E = -4271.39577766 a.u.).

C	3.936029	-2.30483	1.737608	C	1.641195	-1.797635	2.506736
C	4.679972	-1.648532	0.765721	C	3.289619	-0.44912	3.310088
C	5.575403	-2.345116	-0.051019	C	0.896591	-2.753734	1.857631
C	5.691116	-3.721649	0.128799	C	1.02507	-0.744218	3.31916
C	4.945936	-4.380123	1.104698	C	4.49735	0.116443	3.596118
C	4.071061	-3.680383	1.919007	C	2.056412	0.045832	3.833834
H	4.539588	-0.582279	0.631345	C	-0.499058	-2.654149	1.921532
H	6.358146	-4.295752	-0.501132	H	1.367456	-3.516998	1.249589
H	5.055264	-5.452926	1.226521	C	-0.397814	-0.679833	3.384221
H	3.489789	-4.178736	2.686839	C	4.505549	1.257029	4.480041
C	7.3117	-2.458693	-1.92533	H	5.421853	-0.267181	3.183222
C	8.383974	-2.966408	-1.181754	C	2.092542	1.16917	4.713483
C	7.297747	-2.694157	-3.297111	C	-1.12993	-1.587487	2.661037
C	9.386146	-3.711768	-1.781805	H	-1.118333	-3.308714	1.328926
H	8.434328	-2.763951	-0.116685	H	-0.888807	0.084562	3.971622
C	8.309357	-3.435706	-3.905619	C	3.33124	1.749374	5.015055
H	6.498103	-2.295526	-3.908297	H	5.454214	1.724312	4.718338
C	9.352261	-3.953311	-3.15322	H	1.178016	1.552873	5.150747
H	10.202465	-4.09762	-1.17911	H	3.36517	2.604889	5.682638
H	8.273936	-3.603053	-4.977694	N	-2.523982	-1.463446	2.55483
H	10.137999	-4.532677	-3.627557	C	-3.444933	-2.519595	2.495209
C	7.077922	-0.370228	-0.701517	C	-3.222211	-0.263872	2.327038
C	7.704149	0.450536	-1.648019	C	-3.25696	-3.881654	2.722813
C	7.326522	-0.115482	0.64419	C	-4.723704	-1.999552	2.214874
C	8.513708	1.507475	-1.262233	C	-2.750672	1.041939	2.227977
H	7.56537	0.243704	-2.703943	C	-4.584654	-0.561016	2.125399
C	8.1469	0.940104	1.037151	C	-4.356746	-4.717428	2.595596
H	6.89469	-0.751817	1.405497	H	-2.286163	-4.280287	2.988133
C	8.738338	1.760884	0.089455	C	-5.813938	-2.856789	2.086253
H	8.983647	2.127348	-2.019451	C	-3.670447	2.048592	1.967245
H	8.320838	1.112023	2.094888	H	-1.701332	1.276512	2.323556
H	9.378065	2.582957	0.394662	C	-5.49375	0.465187	1.882137
C	5.103793	-1.180356	-2.139147	C	-5.621495	-4.217648	2.266625
C	4.313415	-2.196687	-2.692318	H	-4.225824	-5.783965	2.743846
C	4.7523	0.137632	-2.415546	H	-6.796661	-2.463025	1.846163
C	3.237735	-1.905334	-3.517029	C	-5.031852	1.771096	1.812051
H	4.546808	-3.232603	-2.468783	H	-3.312965	3.067733	1.874129
C	3.666525	0.434803	-3.2385	H	-6.545089	0.244888	1.731452
H	5.313448	0.951142	-1.977234	H	-6.457106	-4.900214	2.15353
C	2.909363	-0.581509	-3.797963	H	-5.727516	2.578515	1.609511
H	2.64605	-2.714958	-3.932862	C	6.265807	-1.583061	-1.201641
H	3.406983	1.472264	-3.427261	C	-0.716024	0.096895	-0.727551
H	2.059568	-0.34915	-4.431406	C	-0.302306	1.379562	-0.374282
N	2.999199	-1.579918	2.515084	C	1.040407	1.68149	-0.154796

C	1.959273	0.638828	-0.235247
C	1.556652	-0.647365	-0.584825
C	0.226156	-0.923832	-0.844789
H	-1.053899	2.152539	-0.284547
H	3.005862	0.82776	-0.045899
H	2.30005	-1.43086	-0.668673
H	-0.090792	-1.916665	-1.138206
C	2.88916	3.294156	0.553809
C	3.266449	3.683232	1.835347
C	3.90892	3.031469	-0.371789
C	4.611191	3.778234	2.1901
H	2.519167	3.919843	2.579479
C	5.246752	3.107515	-0.018813
H	3.640931	2.765155	-1.388097
C	5.605766	3.481383	1.273489
H	4.869237	4.081591	3.199237
H	6.017995	2.883037	-0.748646
H	6.65265	3.541933	1.550582
C	0.431972	3.707762	1.133389
C	-0.409857	4.793352	0.907431
C	0.335319	3.048881	2.364994
C	-1.293933	5.233588	1.891472
H	-0.39665	5.305836	-0.044881
C	-0.527902	3.496431	3.352994
H	0.947389	2.172763	2.547016
C	-1.348346	4.598355	3.122719
H	-1.944993	6.076117	1.680037
H	-0.574054	2.973248	4.303255
H	-2.03365	4.941834	3.891112
C	1.306387	3.956636	-1.234285
C	1.767211	5.278778	-1.253399
C	0.763376	3.439875	-2.408339
C	1.657751	6.064545	-2.390146
H	2.214759	5.694204	-0.356399
C	0.657053	4.225192	-3.556284
H	0.418596	2.414918	-2.444515
C	1.095397	5.540911	-3.552137
H	2.016757	7.08896	-2.370933
H	0.226574	3.79545	-4.45559
H	1.010063	6.152352	-4.444913
C	-2.149798	-0.134985	-0.990946
C	-3.920594	-1.494489	-1.288595
C	-4.199558	0.735274	-1.325936
N	-4.992891	1.860931	-1.438706
C	-6.390016	1.927178	-1.238769
C	-4.507454	3.183269	-1.61706
C	-7.310385	0.925922	-0.942637
C	-6.787285	3.273766	-1.306212
C	-3.222682	3.661005	-1.876227
C	-5.598499	4.066553	-1.548306
C	-8.63653	1.295386	-0.755082
H	-7.001799	-0.101388	-0.844755
C	-8.121128	3.624274	-1.117468
C	-3.054529	5.03044	-2.035463
H	-2.380072	2.993666	-1.94554
C	-5.409941	5.436937	-1.707026
C	-9.047031	2.627731	-0.84859
H	-9.365012	0.52346	-0.527132
H	-8.426161	4.664752	-1.172065
C	-4.131372	5.916726	-1.945292
H	-2.057398	5.40811	-2.241504
H	-6.255671	6.115219	-1.648374
H	-10.090995	2.883301	-0.699474
H	-3.966609	6.982079	-2.070433
N	-4.440333	-2.77247	-1.380894
C	-3.767681	-3.967625	-1.010283
C	-5.795799	-3.100327	-1.637748
C	-2.425658	-4.21198	-0.733207
C	-4.706295	-5.01205	-0.968123
C	-6.833847	-2.330132	-2.154071
C	-5.988116	-4.463495	-1.35799
C	-2.054967	-5.5059	-0.387443
H	-1.704144	-3.414554	-0.781339
C	-4.317819	-6.301257	-0.620329
C	-8.077858	-2.930714	-2.308689
H	-6.675987	-1.303672	-2.439558
C	-7.240033	-5.04863	-1.516905
C	-2.985685	-6.544563	-0.323166
H	-1.010951	-5.707639	-0.167073
H	-5.049699	-7.102461	-0.586917
C	-8.290937	-4.271227	-1.980141
H	-8.896291	-2.337587	-2.704481
H	-7.384616	-6.100808	-1.291805
H	-2.663303	-7.544508	-0.05141
H	-9.275172	-4.709705	-2.108619
N	-4.764876	-0.468337	-1.422202
N	-2.904007	0.949658	-1.134358
N	-2.608139	-1.384303	-1.066819
C	1.421939	3.158781	0.085094

Supplementary Table 11. Atomic coordinates of CCzCz:CTrzCz2 (02, E = -4271.38939897 a.u.).

C	3.468728	-3.002874	0.841384		C	1.136718	-2.377563	0.395561
C	4.385202	-2.238053	0.140613		C	2.262789	-1.154861	1.940406
C	5.605594	-2.757847	-0.283334		C	0.755489	-3.287638	-0.549569
C	5.883228	-4.088362	0.02228		C	0.388994	-1.154709	0.641509
C	4.956971	-4.865498	0.719007		C	3.214797	-0.655018	2.773877
C	3.744788	-4.332797	1.138259		C	1.068798	-0.425843	1.602979
H	4.137899	-1.202518	-0.058823		C	-0.429367	-3.023981	-1.283326
H	6.828274	-4.529289	-0.267825		H	1.337535	-4.181292	-0.741976
H	5.198546	-5.898366	0.948874		C	-0.806532	-0.917306	-0.113991
H	3.024301	-4.919575	1.697862		C	2.980233	0.646234	3.352122
C	5.996516	-1.386862	-2.392557		H	4.132436	-1.193029	2.979688
C	6.787646	-0.580621	-3.219955		C	0.844916	0.854768	2.2267
C	4.756715	-1.799258	-2.875351		C	-1.196825	-1.851067	-1.039615
C	6.342969	-0.174297	-4.468289		H	-0.747129	-3.701983	-2.065282
H	7.768126	-0.269535	-2.873847		H	-1.381561	-0.020044	0.05083
C	4.310411	-1.400175	-4.135127		C	1.811945	1.356662	3.081991
H	4.119738	-2.43879	-2.27815		H	3.730135	1.064631	4.013902
C	5.094738	-0.581231	-4.932973		H	-0.067914	1.399358	2.033722
H	6.975325	0.45802	-5.083707		H	1.65331	2.323735	3.546384
H	3.340477	-1.739519	-4.48586		N	-2.37943	-1.653417	-1.792279
H	4.744391	-0.267842	-5.911229		C	-3.410588	-2.582334	-1.94267
C	7.936638	-2.416087	-1.270208		C	-2.741709	-0.482239	-2.460305
C	9.120145	-1.949642	-0.704829		C	-3.535053	-3.866588	-1.414948
C	8.016064	-3.486415	-2.170105		C	-4.433493	-2.009515	-2.727852
C	10.342146	-2.548459	-1.006942		C	-2.04296	0.715584	-2.589451
H	9.102839	-1.104369	-0.029107		C	-4.002286	-0.668935	-3.062918
C	9.228322	-4.089925	-2.465971		C	-4.698177	-4.570151	-1.693558
H	7.111048	-3.839836	-2.653941		H	-2.751619	-4.296927	-0.803295
C	10.40265	-3.623274	-1.88059		C	-5.589173	-2.738839	-2.999803
H	11.249865	-2.161571	-0.554088		C	-2.638521	1.730793	-3.322473
H	9.258409	-4.920523	-3.164369		H	-1.071994	0.855075	-2.133422
H	11.354686	-4.089232	-2.114333		C	-4.574747	0.363278	-3.803796
C	6.634416	-0.557325	-0.068279		C	-5.715318	-4.018334	-2.480812
C	6.214886	0.718394	-0.433763		H	-4.824902	-5.565633	-1.280405
C	7.024158	-0.762209	1.262162		H	-6.387493	-2.299636	-3.589577
C	6.18385	1.757592	0.49635		C	-3.89203	1.563498	-3.92411
H	5.877629	0.913182	-1.443572		H	-2.124296	2.681421	-3.419238
C	7.006643	0.27072	2.186035		H	-5.547577	0.229736	-4.265024
H	7.322045	-1.756554	1.57962		H	-6.617513	-4.590654	-2.667046
C	6.578376	1.541263	1.807007		H	-4.329803	2.381144	-4.487783
H	5.828473	2.73505	0.185413		C	6.557303	-1.783153	-1.008871
H	7.313765	0.080297	3.209903		C	-3.140865	2.813275	0.068001
H	6.545917	2.348609	2.531631		C	-1.796165	3.150816	0.18185
N	2.25165	-2.375738	1.232321		C	-1.282989	4.334209	-0.351929

C	-2.160509	5.155631	-1.056106		C	-1.622643	-0.244498	3.87788
C	-3.506562	4.814039	-1.197526		C	-2.395546	-3.244085	1.946919
C	-4.005394	3.651443	-0.637507		C	-0.931314	-2.43149	3.721505
H	-1.132979	2.462746	0.687669		C	-1.706022	1.055687	4.366679
H	-1.802643	6.071356	-1.508782		C	-0.727038	-1.179515	4.423991
H	-4.165522	5.472127	-1.754829		C	-1.742385	-4.466547	2.030687
H	-5.049885	3.381831	-0.737863		H	-3.188826	-3.082905	1.232578
C	0.609819	5.96135	-0.84423		C	-0.287527	-3.664358	3.785504
C	1.467777	6.046678	-1.936683		C	-0.855075	1.406402	5.40494
C	0.100215	7.153864	-0.314526		H	-2.392191	1.771947	3.935892
C	1.793841	7.281972	-2.495431		C	0.125264	-0.803052	5.457896
H	1.898875	5.148644	-2.359599		C	-0.698124	-4.680244	2.935593
C	0.415562	8.382611	-0.872335		H	-2.052693	-5.271765	1.372012
H	-0.549293	7.111601	0.55401		H	0.530787	-3.818858	4.481685
C	1.26757	8.452782	-1.972154		C	0.057777	0.493277	5.943314
H	2.46864	7.318951	-3.34518		H	-0.89497	2.416256	5.800319
H	0.001998	9.289786	-0.442593		H	0.830237	-1.515322	5.874454
H	1.521661	9.413599	-2.408642		H	-0.20638	-5.647057	2.970912
C	0.987316	3.437399	-0.779886		H	0.717399	0.804098	6.746773
C	1.986057	2.732465	-0.114293		N	-6.106784	-0.880468	0.111699
C	0.657677	3.026761	-2.077594		C	-6.98809	-0.373609	-0.882248
C	2.632336	1.654225	-0.716927		C	-6.584172	-2.160354	0.512044
H	2.263558	2.994118	0.895492		C	-6.959357	0.811556	-1.611565
C	1.309501	1.964302	-2.687787		C	-8.004275	-1.319965	-1.094502
H	-0.124723	3.551246	-2.616376		C	-6.096968	-3.070143	1.447087
C	2.303527	1.265233	-2.005657		C	-7.747984	-2.444713	-0.220182
H	3.392349	1.122025	-0.154962		C	-7.966715	1.024647	-2.545867
H	1.027005	1.670919	-3.694188		H	-6.164608	1.524695	-1.475685
H	2.809547	0.427708	-2.475213		C	-9.003266	-1.090505	-2.033989
C	0.577731	4.806324	1.33457		C	-6.78069	-4.269923	1.604796
C	1.868251	5.247679	1.656323		H	-5.21895	-2.85603	2.03194
C	-0.308304	4.583066	2.385719		C	-8.421911	-3.647982	-0.045823
C	2.267768	5.421833	2.971797		C	-8.981435	0.090484	-2.76116
H	2.567821	5.461034	0.855052		H	-7.952301	1.943077	-3.124466
C	0.085894	4.767584	3.710479		H	-9.783095	-1.828415	-2.194787
H	-1.324902	4.27063	2.190151		C	-7.928023	-4.566699	0.867447
C	1.374562	5.179306	4.012724		H	-6.405244	-4.989084	2.325882
H	3.277802	5.757705	3.185374		H	-9.318054	-3.861005	-0.620496
H	-0.632097	4.590082	4.505008		H	-9.752148	0.288599	-3.498996
H	1.680489	5.32132	5.044471		H	-8.436665	-5.513906	1.014945
C	-3.60777	1.534603	0.644513		N	-4.232012	-0.919198	1.475066
C	-4.978153	-0.255903	0.584743		N	-2.859257	0.99898	1.612213
C	-3.201311	-0.242115	1.96208		N	-4.695851	0.967921	0.128378
N	-2.387843	-0.883074	2.88271		C	0.221208	4.631791	-0.162051
C	-1.976189	-2.232148	2.802204					

Supplementary Table 12. Atomic coordinates of CCzCz:CTrzCz2 (03, E = -4271.38860717 a.u.).

C	-5.475877	0.365162	1.593282		C	-3.20711	-0.459159	2.197079
C	-6.341501	-0.087647	0.601533		C	-4.96086	-1.846249	2.633045
C	-7.276487	0.753277	0.010415		C	-2.378293	0.559204	1.793263
C	-7.307822	2.086076	0.436178		C	-2.685781	-1.732416	2.702133
C	-6.422078	2.549224	1.401925		C	-6.208332	-2.379016	2.783978
C	-5.502546	1.693914	1.997029		C	-3.771036	-2.560825	2.970438
H	-6.252872	-1.119503	0.298058		C	-0.993443	0.337964	1.846876
H	-8.030984	2.770528	0.00961		H	-2.769377	1.482911	1.385492
H	-6.462497	3.589775	1.70748		C	-1.272956	-1.908274	2.794673
H	-4.826345	2.041249	2.769944		C	-6.29921	-3.731017	3.288411
C	-8.684458	1.345281	-1.993504		H	-7.105843	-1.822395	2.541811
C	-10.002053	1.653707	-2.324734		C	-3.886061	-3.900066	3.460027
C	-7.674069	2.115127	-2.585712		C	-0.458752	-0.908781	2.334443
C	-10.299595	2.674168	-3.225891		H	-0.316632	1.075096	1.441664
H	-10.818432	1.10592	-1.873945		H	-0.858741	-2.81753	3.208866
C	-7.96408	3.128711	-3.486408		C	-5.162441	-4.451737	3.607357
H	-6.638723	1.928443	-2.330378		H	-7.280712	-4.174909	3.41191
C	-9.285906	3.413767	-3.815349		H	-2.997823	-4.469441	3.709147
H	-11.337904	2.888605	-3.459409		H	-5.261639	-5.467096	3.978598
H	-7.152634	3.69957	-3.927288		N	0.932753	-1.102155	2.262225
H	-9.519309	4.208381	-4.516857		C	1.915348	-0.136628	2.522894
C	-9.555374	-0.280676	-0.256277		C	1.574391	-2.268097	1.813736
C	-10.477081	-1.103703	-0.914738		C	1.790907	1.150439	3.044031
C	-9.823122	0.071882	1.064347		C	3.180169	-0.678228	2.221602
C	-11.62492	-1.552209	-0.278467		C	1.039895	-3.483934	1.389927
H	-10.285349	-1.400919	-1.94042		C	2.9638	-2.038366	1.777741
C	-10.972387	-0.381644	1.708696		C	2.950036	1.899436	3.200785
H	-9.132272	0.703233	1.608656		H	0.827585	1.560598	3.318956
C	-11.878256	-1.193619	1.04273		C	4.327913	0.092902	2.37565
H	-12.321008	-2.189356	-0.81515		C	1.920608	-4.464313	0.953599
H	-11.15176	-0.094887	2.740255		H	-0.027214	-3.655179	1.37402
H	-12.77205	-1.548648	1.545845		C	3.830322	-3.038471	1.344668
C	-7.690736	-0.993871	-1.775182		C	4.20615	1.387215	2.856499
C	-7.131707	-0.890558	-3.048457		H	2.87124	2.909699	3.587867
C	-7.644423	-2.24895	-1.151808		H	5.298091	-0.313951	2.120979
C	-6.526077	-1.984772	-3.662668		C	3.303804	-4.253356	0.937255
H	-7.170469	0.045959	-3.587328		H	1.520611	-5.41696	0.619561
C	-7.036941	-3.34002	-1.757116		H	4.898034	-2.853593	1.309286
H	-8.091757	-2.376816	-0.172573		H	5.089591	2.007533	2.965033
C	-6.468453	-3.212807	-3.020723		H	3.96572	-5.039622	0.589321
H	-6.097286	-1.865788	-4.652613		C	-8.30506	0.212623	-1.014546
H	-7.011272	-4.292109	-1.236403		C	3.953919	-1.062639	-1.429449
H	-5.989953	-4.061964	-3.49777		C	5.267969	-0.750859	-1.095703
N	-4.581488	-0.562771	2.174539		C	6.325384	-1.606157	-1.398483

C	6.017345	-2.838653	-1.972233		C	4.101697	3.631385	0.239791
C	4.697653	-3.176079	-2.275081		C	0.569281	4.05362	1.119381
C	3.66347	-2.289876	-2.025499		C	2.771656	5.009595	1.515468
H	5.457066	0.18825	-0.592727		C	5.266821	3.164503	-0.364944
H	6.805569	-3.547279	-2.194419		C	4.134665	4.737522	1.103898
H	4.485175	-4.143045	-2.719549		C	0.049853	5.006721	1.987616
H	2.6382	-2.532705	-2.277306		H	-0.073898	3.332852	0.642287
C	8.826431	-2.126033	-1.538567		C	2.231728	5.958199	2.378277
C	9.785305	-2.71556	-0.719113		C	6.463752	3.795187	-0.049864
C	8.903949	-2.367408	-2.91592		H	5.248509	2.337613	-1.055043
C	10.773177	-3.541952	-1.25255		C	5.343717	5.354958	1.413263
H	9.777786	-2.530401	0.347313		C	0.86457	5.952862	2.613103
C	9.879003	-3.195372	-3.448816		H	-1.018934	5.009187	2.17708
H	8.191579	-1.88683	-3.578779		H	2.875317	6.68565	2.863296
C	10.821559	-3.792336	-2.615128		C	6.510308	4.872341	0.840023
H	11.508787	-3.986587	-0.58931		H	7.375906	3.441791	-0.52074
H	9.910084	-3.367369	-4.520249		H	5.367137	6.20447	2.088914
H	11.589949	-4.437537	-3.029103		H	0.42659	6.684283	3.284376
C	7.749689	-1.017595	0.506944		H	7.461438	5.341314	1.07072
C	7.898619	0.191395	1.183022		N	-0.58772	0.321658	-1.34072
C	7.520643	-2.168593	1.271448		C	-1.202695	-0.958244	-1.326556
C	7.869921	0.243015	2.575906		C	-1.608826	1.298068	-1.429499
H	8.028568	1.112872	0.631307		C	-0.648761	-2.22752	-1.205244
C	7.492545	-2.122	2.657665		C	-2.594901	-0.781406	-1.394948
H	7.364962	-3.117012	0.766601		C	-1.536305	2.68677	-1.507658
C	7.677831	-0.911027	3.320367		C	-2.849006	0.640906	-1.476537
H	7.986855	1.201188	3.072614		C	-1.52238	-3.307807	-1.143302
H	7.316021	-3.032419	3.221756		H	0.418856	-2.366614	-1.14346
H	7.650038	-0.868966	4.40421		C	-3.453766	-1.873849	-1.336204
C	8.112076	0.199272	-1.71052		C	-2.729145	3.397858	-1.582052
C	9.365955	0.759645	-1.43676		H	-0.587686	3.200393	-1.500912
C	7.299006	0.850674	-2.634981		C	-4.030282	1.367405	-1.561197
C	9.771969	1.946109	-2.027228		C	-2.90811	-3.141887	-1.20615
H	10.034511	0.250592	-0.750239		H	-1.105909	-4.305272	-1.040672
C	7.706079	2.040441	-3.237397		H	-4.527918	-1.734099	-1.386611
H	6.335736	0.436337	-2.902057		C	-3.967953	2.751991	-1.598371
C	8.937651	2.599342	-2.931622		H	-2.68731	4.481463	-1.628758
H	10.747338	2.358027	-1.78738		H	-4.979587	0.847056	-1.580366
H	7.047347	2.526519	-3.95018		H	-3.562556	-4.005537	-1.15006
H	9.251547	3.527952	-3.397527		H	-4.883338	3.333178	-1.638946
C	2.900409	-0.065848	-1.151651		N	1.058428	1.786046	-0.626742
C	0.74702	0.577303	-1.100761		N	3.315395	1.124632	-0.7264
C	2.359603	1.999389	-0.436686		N	1.623708	-0.396958	-1.351525
N	2.754895	3.204902	0.107644		C	7.750527	-1.142861	-1.031855
C	1.942056	4.06381	0.887451					

Supplementary Table 13. Atomic coordinates of CCzCz:CTrzCz2 (04, E = -4271.38107128 a.u.).

C	0.498336	-2.329523	2.118535	C	-1.923112	-2.200891	1.558078
C	1.679209	-1.600014	1.961026	C	-1.022064	-0.398046	2.627425
C	2.919706	-2.179597	2.197531	C	-2.169034	-3.380941	0.902827
C	2.965405	-3.553322	2.462604	C	-2.99538	-1.248941	1.84897
C	1.798585	-4.296448	2.549829	C	-0.201074	0.493413	3.254726
C	0.557249	-3.686372	2.421708	C	-2.433618	-0.164746	2.504814
H	1.607104	-0.557458	1.694071	C	-3.492457	-3.658123	0.511547
H	3.920493	-4.030614	2.651155	H	-1.376952	-4.0686	0.642126
H	1.851773	-5.35667	2.775848	C	-4.341159	-1.576989	1.474083
H	-0.352049	-4.249789	2.584628	C	-0.789421	1.708776	3.767685
C	5.152644	-1.593903	1.094218	H	0.858976	0.33014	3.386308
C	6.242178	-0.735761	0.900269	C	-2.997602	1.035394	3.056115
C	4.996489	-2.659718	0.211384	C	-4.563133	-2.757187	0.819175
C	7.169565	-0.966138	-0.103951	H	-3.705493	-4.537585	-0.081444
H	6.36449	0.122358	1.55269	H	-5.163314	-0.921611	1.734208
C	5.923365	-2.892289	-0.802441	C	-2.149987	1.954722	3.665785
H	4.147633	-3.324619	0.306371	H	-0.137431	2.441399	4.227518
C	7.020802	-2.058601	-0.952358	H	-4.062804	1.220044	2.981308
H	7.99957	-0.280303	-0.238839	H	-2.553484	2.8828	4.053506
H	5.771194	-3.720294	-1.487343	N	-5.862286	-3.117782	0.40111
H	7.738587	-2.23814	-1.745073	C	-6.451693	-4.369171	0.586655
C	4.910332	-1.786926	3.611457	C	-6.756428	-2.312542	-0.307321
C	6.268785	-2.078028	3.704129	C	-5.97236	-5.480868	1.277678
C	4.137478	-1.91215	4.774561	C	-7.726815	-4.371539	-0.01429
C	6.840446	-2.463184	4.915733	C	-6.608622	-1.002027	-0.753998
H	6.898673	-2.018841	2.826902	C	-7.920194	-3.057004	-0.589009
C	4.703797	-2.287861	5.98282	C	-6.779884	-6.607662	1.325874
H	3.070869	-1.719559	4.728665	H	-5.006049	-5.46249	1.767138
C	6.06573	-2.565899	6.060584	C	-8.520773	-5.51498	0.049678
H	7.901955	-2.688108	4.95298	C	-7.651191	-0.446316	-1.480708
H	4.076584	-2.372179	6.864945	H	-5.698901	-0.448335	-0.563057
H	6.512089	-2.867038	7.003089	C	-8.957067	-2.474855	-1.316074
C	3.869593	0.159099	2.343105	C	-8.040171	-6.632033	0.715156
C	3.532894	0.812669	1.152221	H	-6.427055	-7.486795	1.855929
C	3.817498	0.899058	3.523321	H	-9.504212	-5.526447	-0.410498
C	3.133701	2.140742	1.143809	C	-8.819056	-1.168341	-1.757668
H	3.58717	0.266624	0.217685	H	-7.557445	0.571235	-1.848364
C	3.415632	2.234235	3.519783	H	-9.856813	-3.040967	-1.537256
H	4.098021	0.444758	4.46372	H	-8.64683	-7.529788	0.772875
C	3.06454	2.859538	2.333137	H	-9.617355	-0.701235	-2.324919
H	2.875039	2.617571	0.207459	C	4.219839	-1.346763	2.294576
H	3.387027	2.781532	4.457259	C	1.046282	3.098014	-1.629387
H	2.752786	3.899573	2.325228	C	-0.187608	3.482218	-1.09966
N	-0.741399	-1.658684	2.035241	C	-0.43857	4.801926	-0.743247

C	0.587276	5.735598	-0.940674
C	1.805104	5.36888	-1.495915
C	2.041807	4.048613	-1.848057
H	-0.931364	2.713005	-0.958266
H	0.425946	6.769714	-0.65937
H	2.57323	6.119543	-1.651292
H	2.986257	3.741847	-2.280839
C	-1.581167	5.325508	1.452208
C	-2.711083	5.528345	2.254226
C	-0.347646	5.196546	2.084524
C	-2.605142	5.637571	3.632518
H	-3.686032	5.60631	1.784631
C	-0.238551	5.293012	3.470568
H	0.545392	5.011421	1.501741
C	-1.361676	5.522897	4.250814
H	-3.497258	5.807386	4.227679
H	0.736986	5.185138	3.93562
H	-1.275802	5.603564	5.329893
C	-2.926896	4.305893	-0.374197
C	-4.020636	4.649667	-1.167545
C	-2.926252	3.036754	0.219543
C	-5.065774	3.753849	-1.37954
H	-4.082352	5.630974	-1.617097
C	-3.952231	2.13245	-0.012235
H	-2.124008	2.754397	0.892173
C	-5.034093	2.490536	-0.810352
H	-5.910099	4.057957	-1.990721
H	-3.902315	1.147416	0.437019
H	-5.853589	1.797721	-0.966516
C	-2.058572	6.679785	-0.646753
C	-2.065653	6.852401	-2.037881
C	-2.294209	7.79793	0.147068
C	-2.321995	8.087575	-2.611019
H	-1.8601	6.000412	-2.677961
C	-2.546841	9.044263	-0.425713
H	-2.273794	7.713599	1.225543
C	-2.566775	9.195696	-1.803091
H	-2.324338	8.187747	-3.691999
H	-2.723815	9.89889	0.22001
H	-2.762918	10.166255	-2.247707
C	1.324994	1.669585	-1.890857
C	2.81118	0.047863	-2.384977
C	0.671178	-0.481539	-1.948802
N	-0.315626	-1.441165	-1.864495
C	-0.074563	-2.831875	-1.772411

C	-1.672947	-1.253577	-2.237362
C	1.043925	-3.511454	-1.299539
C	-1.244529	-3.515202	-2.13872
C	-2.403144	-0.085678	-2.428705
C	-2.251345	-2.517432	-2.445088
C	0.976383	-4.897682	-1.234796
H	1.931164	-2.982448	-0.982719
C	-1.287648	-4.905325	-2.084103
C	-3.722576	-0.21498	-2.846357
H	-1.964325	0.883802	-2.250983
C	-3.57726	-2.624538	-2.851447
C	-0.170124	-5.5932	-1.632604
H	1.834543	-5.445782	-0.859624
H	-2.189687	-5.437857	-2.369619
C	-4.308437	-1.465191	-3.060516
H	-4.30988	0.685467	-2.991937
H	-4.029443	-3.599675	-3.002877
H	-0.188453	-6.676768	-1.576139
H	-5.3456	-1.527788	-3.369294
N	4.076171	-0.327321	-2.78355
C	5.223631	0.503248	-2.771562
C	4.406672	-1.529314	-3.460668
C	5.451104	1.718464	-2.132457
C	6.249972	-0.147779	-3.473508
C	3.652782	-2.674059	-3.701242
C	5.732096	-1.426814	-3.916024
C	6.719658	2.275382	-2.233467
H	4.673655	2.206832	-1.566819
C	7.511765	0.429958	-3.572326
C	4.260916	-3.714468	-4.394332
H	2.637863	-2.761753	-3.348613
C	6.322454	-2.478757	-4.608116
C	7.741517	1.648752	-2.952066
H	6.916246	3.220609	-1.737577
H	8.304875	-0.07499	-4.115315
C	5.580822	-3.627384	-4.843483
H	3.688355	-4.617156	-4.58248
H	7.348786	-2.399964	-4.953604
H	8.719698	2.114194	-3.016105
H	6.025802	-4.46027	-5.37808
N	1.90219	-0.909393	-2.231465
N	0.326024	0.793319	-1.766068
N	2.579864	1.34916	-2.206473
C	-1.756086	5.273916	-0.08174

Supplementary Table 14. Atomic coordinates of CCzCz:CTrzCz2 (05, E = -4271.37951293 a.u.).

C	4.679629	-1.099273	-0.142034	C	2.54946	0.081653	-0.643785
C	6.026018	-0.755431	-0.282833	C	3.863183	0.990921	0.977942
C	6.998006	-1.730284	-0.463573	C	2.025658	-0.633649	-1.691575
C	6.58402	-3.065509	-0.556466	C	1.894471	1.287153	-0.133651
C	5.246824	-3.406634	-0.428401	C	4.841329	1.235491	1.896569
C	4.282537	-2.428775	-0.206924	C	2.718891	1.839365	0.836455
H	6.290943	0.291827	-0.263991	C	0.801146	-0.204083	-2.235565
H	7.321168	-3.83993	-0.739197	H	2.540844	-1.489647	-2.108853
H	4.948977	-4.448328	-0.495458	C	0.608599	1.649235	-0.646992
H	3.238272	-2.684409	-0.076887	C	4.718415	2.426971	2.705213
C	8.922601	-2.055994	-1.959299	H	5.689952	0.575437	2.022255
C	9.98295	-2.949739	-2.079957	C	2.621882	3.01574	1.646748
C	8.16929	-1.774533	-3.106692	C	0.08363	0.905339	-1.668255
C	10.295974	-3.52795	-3.309432	H	0.40357	-0.683677	-3.119503
H	10.573031	-3.214129	-1.212322	H	0.050139	2.459602	-0.199248
C	8.483164	-2.340438	-4.332225	C	3.637703	3.281279	2.563843
H	7.318122	-1.105561	-3.031134	H	5.494738	2.644213	3.430555
C	9.554578	-3.223524	-4.440461	H	1.771969	3.676599	1.539449
H	11.126306	-4.224609	-3.372314	H	3.57208	4.16776	3.186561
H	7.883402	-2.098712	-5.204248	N	-1.176714	1.223807	-2.214542
H	9.799854	-3.673994	-5.397066	C	-2.16527	0.302244	-2.575899
C	9.324556	-1.941317	0.561378	C	-1.655219	2.511842	-2.494257
C	10.681997	-1.601567	0.630895	C	-2.182467	-1.086408	-2.451039
C	8.791538	-2.705186	1.595753	C	-3.274639	0.999606	-3.096137
C	11.481512	-2.035368	1.676303	C	-1.030161	3.748769	-2.350912
H	11.11113	-0.978184	-0.14682	C	-2.953492	2.4096	-3.034313
C	9.590798	-3.135761	2.654154	C	-3.307613	-1.764055	-2.898206
H	7.741513	-2.96684	1.594991	H	-1.349694	-1.622211	-2.015552
C	10.937359	-2.810347	2.697726	C	-4.392975	0.297061	-3.537584
H	12.531185	-1.759308	1.699508	C	-1.740906	4.88047	-2.72521
H	9.146228	-3.727032	3.448855	H	-0.025473	3.833038	-1.958658
H	11.558521	-3.14668	3.521857	C	-3.651775	3.560172	-3.392326
C	8.731276	0.125602	-0.636479	C	-4.40206	-1.085758	-3.446845
C	9.16422	0.837692	-1.75078	H	-3.331429	-2.846019	-2.812518
C	8.515221	0.836044	0.551769	H	-5.250222	0.828625	-3.938594
C	9.349838	2.218442	-1.689347	C	-3.04327	4.794687	-3.230063
H	9.368092	0.322756	-2.68042	H	-1.277226	5.853518	-2.60251
C	8.684028	2.209773	0.612884	H	-4.660091	3.489291	-3.788359
H	8.212697	0.296019	1.443271	H	-5.27224	-1.64102	-3.779903
C	9.102636	2.911755	-0.514842	H	-3.58334	5.701253	-3.480851
H	9.690581	2.747837	-2.573812	C	8.498082	-1.401621	-0.626325
H	8.491779	2.733667	1.543974	C	-5.174853	0.155534	-0.069719
H	9.24013	3.987474	-0.471517	C	-5.195053	-1.203038	0.255412
N	3.726832	-0.076192	0.060381	C	-6.266673	-2.013444	-0.095479

C	-7.316018	-1.42997	-0.816988		C	-0.446176	-0.688662	1.736649
C	-7.297764	-0.084533	-1.157376		C	-0.291769	2.388503	3.702403
C	-6.229253	0.717803	-0.782263		C	0.916761	0.310078	3.301536
H	-4.349549	-1.598267	0.797955		C	-0.896298	-1.654323	0.843653
H	-8.159306	-2.04301	-1.116902		C	0.73952	-0.866249	2.472298
H	-8.123126	0.338214	-1.721412		C	0.654947	2.698543	4.671114
H	-6.192023	1.765562	-1.053045		H	-1.116086	3.055407	3.494799
C	-7.344776	-3.691923	1.477394		C	1.859076	0.643923	4.268363
C	-7.388505	-4.934286	2.123216		C	-0.127313	-2.801167	0.695327
C	-8.184311	-2.685184	1.94584		H	-1.817521	-1.523164	0.29789
C	-8.252892	-5.166575	3.181662		C	1.492311	-2.025588	2.313596
H	-6.726561	-5.727136	1.790805		C	1.723015	1.842799	4.951309
C	-9.049574	-2.912783	3.014876		H	0.557368	3.630778	5.218481
H	-8.166207	-1.70446	1.488802		H	2.686447	-0.025274	4.480962
C	-9.092165	-4.151945	3.634937		C	1.050668	-2.993744	1.422378
H	-8.265424	-6.140991	3.660231		H	-0.469229	-3.570637	0.011546
H	-9.688437	-2.106484	3.361963		H	2.412208	-2.163181	2.87327
H	-9.765182	-4.327649	4.468243		H	2.452278	2.120601	5.704904
C	-4.994682	-4.046305	0.76493		H	1.6194	-3.909771	1.294478
C	-4.220634	-4.936624	0.025492		N	-3.141883	4.387354	0.517981
C	-4.504257	-3.636539	2.012386		C	-4.213557	5.127681	-0.045361
C	-2.992997	-5.390905	0.506336		C	-2.038985	5.265861	0.682173
H	-4.572961	-5.302052	-0.929913		C	-5.515037	4.748993	-0.363172
C	-3.278078	-4.073909	2.486711		C	-3.78297	6.448634	-0.246251
H	-5.099252	-2.964424	2.621569		C	-0.755948	5.043242	1.177395
C	-2.513397	-4.959928	1.733347		C	-2.410943	6.535085	0.207316
H	-2.417061	-6.092819	-0.089589		C	-6.354246	5.70797	-0.916305
H	-2.917795	-3.723084	3.448339		H	-5.855646	3.742096	-0.189469
H	-1.552687	-5.302948	2.102888		C	-4.636084	7.395609	-0.803697
C	-6.864398	-4.265759	-0.952588		C	0.139197	6.106876	1.170335
C	-7.940082	-5.148773	-0.947614		H	-0.466767	4.079139	1.561124
C	-6.213083	-4.036584	-2.17066		C	-1.502249	7.588104	0.211123
C	-8.340966	-5.794739	-2.116787		C	-5.925043	7.017265	-1.146486
H	-8.486446	-5.336549	-0.032716		H	-7.370262	5.42562	-1.173852
C	-6.603052	-4.681053	-3.33385		H	-4.294698	8.413843	-0.962767
H	-5.394658	-3.32828	-2.197395		C	-0.220263	7.368144	0.691884
C	-7.675101	-5.57	-3.311877		H	1.14347	5.945781	1.549909
H	-9.186488	-6.474998	-2.083586		H	-1.795742	8.564687	-0.16142
H	-6.07292	-4.484094	-4.261018		H	-6.605345	7.740383	-1.584405
H	-7.98949	-6.073984	-4.220365		H	0.503351	8.17658	0.700531
C	-4.042162	0.98957	0.380793		N	-2.067167	2.503864	1.342395
C	-3.118089	3.02101	0.704447		N	-3.006273	0.370569	0.952606
C	-2.05816	1.173975	1.424412		N	-4.140126	2.309463	0.229017
N	-0.976843	0.582584	2.049752		C	-6.366064	-3.50657	0.296343
C	-0.142736	1.188173	3.01422					

Supplementary Table 15. Atomic coordinates of CCzCz:CTrzCz2 (06, E = -4271.38098577 a.u.).

C	-1.919963	4.672065	-0.553613		C	-3.069904	3.045383	-2.025663
C	-1.484063	3.872918	0.499606		C	-1.257814	4.070001	-2.890369
C	-1.352669	4.387307	1.793124		C	-4.114238	2.628263	-1.203417
C	-1.558063	5.758053	1.960201		C	-2.767823	2.37889	-3.230707
C	-1.930999	6.57078	0.892693		C	-0.148125	4.881238	-3.098723
C	-2.14261	6.031593	-0.366803		C	-1.611424	3.047106	-3.793753
H	-1.25432	2.831479	0.304061		C	-4.788189	1.468565	-1.550366
H	-1.423986	6.211207	2.932429		H	-4.36145	3.165492	-0.295345
H	-2.078696	7.632952	1.058863		C	-3.491487	1.245225	-3.59092
H	-2.481117	6.63972	-1.198679		C	0.615143	4.646862	-4.232353
C	-2.074542	2.396779	3.251255		H	0.111354	5.662014	-2.393136
C	-2.079109	1.764808	4.502338		C	-0.835712	2.837797	-4.933623
C	-3.049892	2.00756	2.336505		C	-4.467264	0.767714	-2.725134
C	-3.020986	0.798283	4.824645		H	-5.559487	1.072453	-0.898872
H	-1.332645	2.038812	5.23907		H	-3.272413	0.705391	-4.505663
C	-3.990762	1.029135	2.649837		C	0.275544	3.63894	-5.14468
H	-3.094806	2.460973	1.359063		H	1.501351	5.24809	-4.405762
C	-3.987735	0.423258	3.896809		H	-1.092481	2.052053	-5.637566
H	-2.99455	0.332853	5.805186		H	0.895532	3.481201	-6.020692
H	-4.728602	0.739756	1.907554		N	-5.093166	-0.476107	-2.988109
H	-4.719548	-0.339995	4.135056		C	-6.458163	-0.739543	-2.924685
C	-0.753736	4.247589	4.251829		C	-4.415632	-1.689306	-3.123344
C	0.483005	4.419154	4.86571		C	-7.523756	0.148843	-2.797323
C	-1.874589	4.824889	4.864273		C	-6.665318	-2.130721	-3.036952
C	0.601807	5.156264	6.04387		C	-3.045157	-1.935768	-3.184812
H	1.369439	3.970704	4.437303		C	-5.356273	-2.739073	-3.160418
C	-1.759657	5.563637	6.030824		C	-8.80471	-0.382168	-2.766476
H	-2.853123	4.683599	4.41661		H	-7.35697	1.217884	-2.730178
C	-0.513267	5.734694	6.629246		C	-7.963154	-2.63962	-3.003866
H	1.579438	5.269262	6.502253		C	-2.634467	-3.256177	-3.286178
H	-2.646384	6.000994	6.479262		H	-2.322892	-1.131132	-3.128354
H	-0.419053	6.307633	7.546349		C	-4.915599	-4.059482	-3.254371
C	0.34294	2.712366	2.549972		C	-9.027427	-1.76207	-2.864658
C	0.534629	1.346367	2.73642		H	-9.652252	0.288677	-2.6669
C	1.410053	3.459371	2.036117		H	-8.135502	-3.708398	-3.088306
C	1.759523	0.74482	2.457828		C	-3.554551	-4.312478	-3.315428
H	-0.270006	0.728723	3.104501		H	-1.57347	-3.474887	-3.331213
C	2.636772	2.867816	1.770029		H	-5.630373	-4.876677	-3.265999
H	1.283027	4.523496	1.862432		H	-10.042508	-2.144154	-2.835842
C	2.818875	1.503694	1.983885		H	-3.193192	-5.333704	-3.370062
H	1.870692	-0.323195	2.616499		C	-0.961485	3.437008	2.951548
H	3.448838	3.472661	1.381959		C	0.759714	-0.67044	-0.469964
H	3.777996	1.040043	1.772469		C	0.185223	-1.707386	0.255971
N	-2.158973	4.075709	-1.825907		C	-1.191275	-1.797048	0.462482

C	-1.98034	-0.759222	-0.021159		C	4.328072	-4.060926	0.40134
C	-1.409429	0.295944	-0.732175		C	7.287722	-1.946726	0.089098
C	-0.049506	0.346407	-0.977953		C	6.594045	-4.205206	0.764132
H	0.841063	-2.454501	0.680958		C	3.047245	-4.527301	0.33343
H	-3.049385	-0.756165	0.14252		C	5.412931	-4.907485	0.862384
H	-2.047137	1.077391	-1.114488		C	8.624039	-2.319266	0.429762
H	0.389173	1.154138	-1.552793		H	7.074622	-0.955949	-0.278119
C	-3.240229	-2.937919	1.470268		C	7.956866	-4.55275	1.087067
C	-3.844053	-2.92903	2.72453		C	2.792406	-5.867568	0.755649
C	-4.069742	-2.942672	0.340996		H	2.238823	-3.91502	-0.027595
C	-5.232145	-2.883126	2.850033		C	5.120428	-6.250503	1.301821
H	-3.236297	-2.960037	3.619537		C	8.934235	-3.607261	0.914854
C	-5.449494	-2.874688	0.461774		H	9.412898	-1.585993	0.307449
H	-3.626987	-3.002393	-0.647124		H	8.185295	-5.543593	1.462899
C	-6.039059	-2.837832	1.723297		C	3.825825	-6.697862	1.237514
H	-5.677038	-2.884278	3.841109		H	1.778186	-6.245445	0.685616
H	-6.064829	-2.859887	-0.431269		H	5.921879	-6.883524	1.665614
H	-7.119283	-2.79117	1.82094		H	9.96554	-3.846817	1.154173
C	-0.917323	-2.982869	2.575314		H	3.586852	-7.707267	1.557361
C	0.022248	-3.950373	2.921385		N	4.762938	1.414707	-1.905228
C	-1.043921	-1.862312	3.404314		C	4.275822	2.744551	-1.917472
C	0.810687	-3.807088	4.062112		C	6.068276	1.416746	-2.447808
H	0.167477	-4.819257	2.292704		C	3.071276	3.271074	-1.460717
C	-0.275763	-1.725994	4.550118		C	5.279834	3.578809	-2.440365
H	-1.746669	-1.080781	3.137072		C	6.937722	0.361722	-2.713053
C	0.663437	-2.699787	4.88368		C	6.406031	2.736942	-2.791881
H	1.549134	-4.568187	4.29419		C	2.906684	4.648865	-1.518028
H	-0.400731	-0.846042	5.173466		H	2.29903	2.632963	-1.062731
H	1.277729	-2.587466	5.771497		C	5.083372	4.954823	-2.513732
C	-1.460421	-4.33958	0.501594		C	8.161559	0.662164	-3.297607
C	-1.984377	-5.516985	1.048637		H	6.673381	-0.655869	-2.469507
C	-0.734818	-4.440892	-0.68167		C	7.638422	3.017394	-3.374982
C	-1.770677	-6.748788	0.450047		C	3.89249	5.488103	-2.043739
H	-2.5693	-5.460722	1.96069		H	1.981425	5.072276	-1.143402
C	-0.518786	-5.677057	-1.289681		H	5.859129	5.597895	-2.917754
H	-0.333476	-3.553452	-1.151293		C	8.517948	1.974341	-3.621056
C	-1.030225	-6.836794	-0.726843		H	8.852783	-0.148063	-3.506939
H	-2.187103	-7.643994	0.90146		H	7.900057	4.037667	-3.638054
H	0.054231	-5.723197	-2.210996		H	3.727209	6.560245	-2.078661
H	-0.861572	-7.799187	-1.199782		H	9.483387	2.174931	-4.07395
C	2.220258	-0.668418	-0.667167		N	4.878773	-0.689485	-0.935508
C	4.11891	0.327228	-1.342834		N	2.879907	-1.744821	-0.248842
C	4.20193	-1.713263	-0.405009		N	2.791151	0.39085	-1.243763
N	4.903092	-2.810614	0.029699		C	-1.716326	-3.014358	1.254897
C	6.305378	-2.879783	0.244875					

Supplementary Table 16. Atomic coordinates of CCzCz:CTrzCz2 (07, E = -4271.38051295 a.u.).

C	-2.172506	-0.937548	0.47554		C	-4.63254	-1.179443	0.128046
C	-1.244416	-1.349236	1.425957		C	-3.394212	-3.063374	-0.012056
C	-0.031898	-0.672718	1.595868		C	-5.089666	0.122476	0.326942
C	0.201082	0.446775	0.801359		C	-5.51446	-2.219632	-0.234943
C	-0.749489	0.888589	-0.111261		C	-2.360951	-3.99789	-0.029771
C	-1.936243	0.200999	-0.289462		C	-4.720339	-3.426038	-0.325853
H	-1.477512	-2.211928	2.037168		C	-6.439716	0.370661	0.145421
H	1.130598	0.987735	0.882084		H	-4.415276	0.914516	0.629721
H	-0.544014	1.760918	-0.720362		C	-6.86436	-1.945629	-0.436686
H	-2.650194	0.511885	-1.041888		C	-2.684278	-5.307142	-0.351557
C	0.556237	-0.426261	4.036367		H	-1.341199	-3.720516	0.200504
C	1.498503	-0.308769	5.063847		C	-5.019444	-4.748872	-0.651013
C	-0.734241	0.038038	4.282819		C	-7.321568	-0.648984	-0.245227
C	1.167437	0.263655	6.284375		H	-6.836983	1.366376	0.310025
H	2.507759	-0.670455	4.903812		H	-7.558021	-2.722116	-0.741921
C	-1.071678	0.612626	5.505649		C	-3.998353	-5.685565	-0.65991
H	-1.492861	-0.038843	3.514279		H	-1.894666	-6.051794	-0.358282
C	-0.122824	0.733562	6.511693		H	-6.037461	-5.037612	-0.894594
H	1.922117	0.341343	7.061021		H	-4.215946	-6.720002	-0.904671
H	-2.085415	0.967258	5.66598		N	-8.69302	-0.345042	-0.440446
H	-0.384752	1.182909	7.464501		C	-9.196671	0.575996	-1.353041
C	2.400914	-0.712929	2.357553		C	-9.760685	-0.919868	0.242143
C	3.376639	-1.624148	1.957466		C	-8.512677	1.374091	-2.267257
C	2.778796	0.632222	2.469172		C	-10.604005	0.597508	-1.25715
C	4.677688	-1.208906	1.677556		C	-9.746382	-1.874571	1.256439
H	3.143578	-2.677031	1.876892		C	-10.965587	-0.362278	-0.234722
C	4.067134	1.051574	2.175472		C	-9.264482	2.209746	-3.079594
H	2.060002	1.364973	2.813556		H	-7.4316	1.338262	-2.340996
C	5.031889	0.127887	1.783843		C	-11.33627	1.447256	-2.085087
H	5.417032	-1.946202	1.379009		C	-10.966911	-2.273604	1.780148
H	4.313916	2.104939	2.263433		H	-8.813281	-2.28646	1.623914
H	6.046194	0.445311	1.567891		C	-12.179376	-0.781417	0.308443
C	0.836933	-2.64906	2.877286		C	-10.662464	2.251666	-2.991088
C	0.656739	-3.266584	4.112181		H	-8.757268	2.842986	-3.80097
C	0.907812	-3.466504	1.74264		H	-12.419992	1.475133	-2.021957
C	0.55435	-4.652935	4.213257		C	-12.174318	-1.73746	1.312182
H	0.583843	-2.670965	5.012637		H	-10.984918	-3.01668	2.571336
C	0.809383	-4.846642	1.838433		H	-13.114808	-0.361672	-0.049377
H	1.038267	-3.011719	0.7671		H	-11.220283	2.918206	-3.640694
C	0.629664	-5.448522	3.080676		H	-13.111879	-2.073538	1.742814
H	0.408121	-5.104561	5.189671		C	0.948463	-1.119643	2.710093
H	0.871273	-5.446586	0.938111		C	3.908806	2.52681	-1.3017
H	0.544825	-6.527848	3.159484		C	2.693937	3.058822	-0.885617
N	-3.350279	-1.696687	0.266412		C	2.511596	4.437362	-0.744572

C	3.595809	5.270069	-1.008536
C	4.823158	4.743619	-1.409659
C	4.987311	3.378605	-1.558091
H	1.883747	2.374688	-0.666244
H	3.494554	6.341797	-0.894182
H	5.652711	5.414782	-1.607674
H	5.931917	2.95604	-1.878735
C	1.104529	6.498909	-0.21136
C	0.945536	7.258687	0.943846
C	1.1631	7.17097	-1.438725
C	0.863805	8.649125	0.878951
H	0.87823	6.772737	1.908611
C	1.091436	8.553113	-1.505605
H	1.260168	6.596385	-2.354424
C	0.941266	9.302673	-0.341024
H	0.736317	9.217071	1.795362
H	1.144123	9.047623	-2.470609
H	0.878742	10.385165	-0.390126
C	1.041904	4.393214	1.222444
C	-0.062692	3.700156	1.707218
C	2.118127	4.593497	2.09894
C	-0.106052	3.242385	3.022835
H	-0.902496	3.484925	1.062857
C	2.076336	4.145284	3.410583
H	3.002194	5.112635	1.744077
C	0.956156	3.464626	3.884407
H	-0.973476	2.690029	3.364567
H	2.92614	4.319708	4.063488
H	0.922181	3.089459	4.901915
C	-0.010351	4.522663	-1.128203
C	-1.311785	4.911327	-0.78477
C	0.163441	3.850002	-2.336768
C	-2.400162	4.573482	-1.574453
H	-1.469546	5.499392	0.112881
C	-0.925401	3.531174	-3.147022
H	1.155187	3.575513	-2.670651
C	-2.213569	3.870709	-2.762174
H	-3.397251	4.876322	-1.270461
H	-0.751363	3.022333	-4.090202
H	-3.062211	3.615199	-3.388754
C	4.070562	1.064743	-1.45643
C	5.425738	-0.729581	-1.524736
C	3.201393	-0.995383	-1.704268
N	2.117172	-1.822382	-1.88598
C	2.115902	-3.232094	-1.716116

C	0.850511	-1.432507	-2.404471
C	3.061028	-4.082285	-1.21736
C	0.808277	-3.712618	-2.122671
C	0.388	-0.227278	-2.834082
C	0.045117	-2.634158	-2.514537
C	2.749276	-5.472342	-1.161113
H	4.022737	-3.726828	-0.885311
C	0.528257	-5.125403	-2.06655
C	-0.940451	-0.173068	-3.365974
H	0.984404	0.668099	-2.751903
C	-1.306937	-2.538935	-3.01367
C	1.502973	-5.969828	-1.59886
H	3.497591	-6.155759	-0.776699
H	-0.435181	-5.494477	-2.397227
C	-1.762481	-1.31597	-3.432059
H	-1.329253	0.785915	-3.685372
H	-1.934561	-3.421429	-3.041088
H	1.316609	-7.038615	-1.556577
H	-2.774921	-1.218758	-3.811013
N	6.694056	-1.270056	-1.43559
C	7.827299	-0.628602	-0.873641
C	7.050129	-2.606522	-1.741288
C	8.000274	0.672946	-0.406161
C	8.872957	-1.565217	-0.798747
C	6.325993	-3.613881	-2.373943
C	8.384807	-2.811514	-1.354025
C	9.234833	1.007938	0.137107
H	7.202012	1.39492	-0.472398
C	10.103334	-1.207516	-0.256008
C	6.953679	-4.837679	-2.57025
H	5.310209	-3.453366	-2.698375
C	8.996033	-4.045101	-1.558422
C	10.280321	0.084696	0.213465
H	9.385572	2.018006	0.504794
H	10.908317	-1.933714	-0.200428
C	8.27108	-5.061254	-2.161627
H	6.40003	-5.634543	-3.056683
H	10.026489	-4.202577	-1.255437
H	11.232816	0.381333	0.640125
H	8.730523	-6.030194	-2.327302
N	4.404804	-1.569576	-1.661074
N	2.9756	0.317646	-1.587562
N	5.318597	0.595271	-1.451803
C	1.159046	4.954599	-0.215647

Supplementary Table 17. Atomic coordinates of CCzCz:CTrzCz2 (08, E = -4271.37249410 a.u.).

C	-2.355925	-1.215759	2.562996	C	-4.307089	-0.796	1.06515
C	-0.983266	-0.975537	2.662248	C	-3.201735	1.018142	1.840136
C	-0.195677	-1.706557	3.546252	C	-4.69883	-2.078106	0.683732
C	-0.7874	-2.762936	4.246353	C	-5.077809	0.329111	0.716971
C	-2.142068	-3.023677	4.125507	C	-2.316185	1.897367	2.458535
C	-2.941207	-2.234213	3.308195	C	-4.365789	1.490577	1.201294
H	-0.542282	-0.211139	2.03796	C	-5.885447	-2.216141	-0.017336
H	-0.176527	-3.37998	4.89589	H	-4.082395	-2.941503	0.901161
H	-2.5887	-3.834443	4.692197	C	-6.287318	0.171776	0.045839
H	-4.011043	-2.396931	3.25874	C	-2.606085	3.252242	2.405517
C	1.973593	-2.746306	3.194181	H	-1.429812	1.547467	2.967143
C	2.61897	-3.677878	4.004367	C	-4.631925	2.856468	1.150093
C	1.798302	-3.061904	1.843849	C	-6.692908	-1.105461	-0.315329
C	3.085902	-4.880482	3.477575	H	-6.206164	-3.197876	-0.347623
H	2.751651	-3.480131	5.060549	H	-6.911879	1.027287	-0.185894
C	2.269741	-4.252096	1.314937	C	-3.745676	3.735248	1.75122
H	1.260239	-2.370028	1.207735	H	-1.926224	3.948851	2.885
C	2.918479	-5.172027	2.132402	H	-5.519775	3.222844	0.645201
H	3.580523	-5.591224	4.132849	H	-3.936558	4.802852	1.716172
H	2.127292	-4.461896	0.260763	N	-7.922953	-1.302491	-0.991821
H	3.290196	-6.104714	1.720739	C	-8.912787	-2.211622	-0.621958
C	1.679523	-1.197048	5.211113	C	-8.333895	-0.655697	-2.155481
C	3.031924	-1.047521	5.541526	C	-8.971606	-3.052926	0.487567
C	0.737036	-0.992898	6.215182	C	-9.962427	-2.158271	-1.562209
C	3.428542	-0.742827	6.833643	C	-7.686368	0.327833	-2.900038
H	3.781988	-1.162817	4.76583	C	-9.59091	-1.163252	-2.545513
C	1.131749	-0.677217	7.514372	C	-10.093414	-3.856045	0.628724
H	-0.319684	-1.071168	5.993653	H	-8.172416	-3.073236	1.219169
C	2.475622	-0.557986	7.832508	C	-11.078923	-2.976837	-1.398819
H	4.485081	-0.638347	7.060348	C	-8.316306	0.794239	-4.044256
H	0.374375	-0.521771	8.276577	H	-6.729083	0.72423	-2.589125
H	2.781878	-0.313743	8.844757	C	-10.205303	-0.676608	-3.698188
C	1.797214	-0.182481	3.015502	C	-11.137601	-3.825929	-0.304976
C	2.869378	-0.162925	2.128902	H	-10.162965	-4.519765	1.484912
C	1.222686	1.042214	3.378176	H	-11.892503	-2.944226	-2.11727
C	3.357037	1.042333	1.626551	C	-9.564571	0.300589	-4.44412
H	3.344783	-1.089711	1.836171	H	-7.828487	1.5597	-4.639907
C	1.682479	2.24019	2.853631	H	-11.172957	-1.060753	-4.006764
H	0.421489	1.053817	4.110187	H	-12.000197	-4.469154	-0.16533
C	2.765886	2.246529	1.978881	H	-10.031885	0.68816	-5.343347
H	4.208365	1.034568	0.954154	C	1.315105	-1.460238	3.733206
H	1.209738	3.174252	3.14189	C	4.141357	1.229969	-1.826864
H	3.150722	3.181044	1.584663	C	4.145471	-0.160188	-1.921139
N	-3.164756	-0.380234	1.751913	C	5.29147	-0.865209	-2.295249

C	6.437148	-0.1265	-2.589784		C	-2.147499	3.21199	-1.554312
C	6.447931	1.26108	-2.480691		C	-2.691458	0.833396	-1.811264
C	5.310279	1.945439	-2.090866		C	0.166034	-1.126231	-0.88957
H	3.225417	-0.686372	-1.704014		C	-2.05477	-0.373239	-1.621155
H	7.340031	-0.628725	-2.909223		C	-3.4714	3.501334	-2.017784
H	7.358234	1.806737	-2.707484		H	-1.476225	4.013695	-1.292489
H	5.310286	3.023069	-1.989584		C	-4.007651	1.159233	-2.301646
C	6.396389	-2.954493	-3.218865		C	-0.310198	-2.462411	-1.016122
C	6.150906	-3.41341	-4.51124		H	1.179863	-0.92944	-0.575741
C	7.721965	-2.969643	-2.765196		C	-2.496901	-1.735839	-1.76652
C	7.188899	-3.859998	-5.325683		C	-4.365536	2.479132	-2.394961
H	5.140151	-3.438447	-4.896283		H	-3.778727	4.538302	-2.088193
C	8.759167	-3.409055	-3.574987		H	-4.674677	0.360295	-2.599593
H	7.943026	-2.639121	-1.756286		C	-1.620917	-2.743136	-1.455497
C	8.497202	-3.858222	-4.865498		H	0.361259	-3.278826	-0.787428
H	6.962826	-4.215367	-6.326323		H	-3.509076	-1.942124	-2.092586
H	9.775087	-3.406136	-3.192127		H	-5.351409	2.748292	-2.762968
H	9.305086	-4.208358	-5.500241		H	-1.934047	-3.77865	-1.546897
C	3.900446	-2.90807	-2.793882		N	1.902946	5.195495	-0.563734
C	3.261421	-4.021847	-2.252884		C	2.937307	6.094689	-0.918711
C	3.272913	-2.259754	-3.864437		C	0.950932	5.899453	0.213058
C	2.043344	-4.470664	-2.756301		C	4.066089	5.910986	-1.713293
H	3.707423	-4.549235	-1.421163		C	2.63832	7.353677	-0.371588
C	2.053628	-2.697296	-4.363091		C	-0.21753	5.462805	0.834332
H	3.741774	-1.389328	-4.309675		C	1.388643	7.228632	0.350067
C	1.428085	-3.808333	-3.808691		C	4.895401	7.004837	-1.924597
H	1.572716	-5.342614	-2.310899		H	4.284795	4.949829	-2.149855
H	1.589095	-2.161878	-5.184941		C	3.48444	8.436927	-0.590846
H	0.469418	-4.146081	-4.188519		C	-0.942792	6.39421	1.568332
C	5.547491	-2.921404	-0.856941		H	-0.555029	4.441534	0.74629
C	5.94038	-4.250674	-0.654048		C	0.647206	8.143392	1.091518
C	5.418558	-2.108777	0.267181		C	4.618366	8.255917	-1.366863
C	6.184728	-4.745609	0.618625		H	5.780786	6.878108	-2.539548
H	6.071734	-4.903343	-1.510396		H	3.25193	9.408129	-0.165195
C	5.666552	-2.60069	1.546594		C	-0.525424	7.721035	1.697447
H	5.136974	-1.070308	0.154214		H	-1.862642	6.077677	2.048215
C	6.049669	-3.919685	1.730046		H	0.98792	9.168986	1.193864
H	6.488273	-5.780929	0.740701		H	5.290156	9.088414	-1.548429
H	5.557065	-1.939519	2.400538		H	-1.120065	8.42005	2.276338
H	6.236376	-4.303599	2.727264		N	0.662451	3.255472	-0.826855
C	2.898736	1.944616	-1.457227		N	1.7767	1.238242	-1.370463
C	1.847725	3.853603	-0.893245		N	2.988966	3.259971	-1.240957
C	0.678315	1.947749	-1.095464		C	5.277879	-2.413209	-2.294458
N	-0.530051	1.29584	-1.134515		C	-2.147499	3.21199	-1.554312
C	-1.77498	1.905553	-1.469029					

Supplementary Table 18. Atomic coordinates of CCzCz:CTrzCz2 (09, E = -4271.36082798 a.u.).

C	3.933538	0.882614	0.174835	C	4.585006	-1.343936	-0.731167
C	3.950548	2.20654	-0.255465	C	2.952792	-0.242205	-1.845963
C	4.01575	3.264976	0.648007	C	5.614962	-1.720169	0.129048
C	4.141609	2.951819	2.004726	C	4.150942	-2.197282	-1.765502
C	4.127087	1.631587	2.437108	C	2.000807	0.674537	-2.291313
C	3.99081	0.586819	1.533938	C	3.112021	-1.491353	-2.481738
H	3.912774	2.392168	-1.317617	C	6.16931	-2.978269	-0.032117
H	4.23445	3.746316	2.734213	H	5.979768	-1.052446	0.89923
H	4.201601	1.414244	3.498174	C	4.699921	-3.469509	-1.896763
H	3.909389	-0.438209	1.873264	C	1.218921	0.318466	-3.38073
C	5.489768	5.163359	-0.130425	H	1.872243	1.637795	-1.816105
C	6.587433	4.481514	0.389232	C	2.31179	-1.82877	-3.572127
C	5.7331	6.295741	-0.916509	C	5.703723	-3.859014	-1.020362
C	7.886486	4.913799	0.133713	H	6.978917	-3.302064	0.612744
H	6.438292	3.598267	0.997472	H	4.350893	-4.158797	-2.658526
C	7.025577	6.731222	-1.170505	C	1.366516	-0.9196	-4.018063
H	4.894956	6.841424	-1.33659	H	0.472071	1.016886	-3.742771
C	8.11287	6.038762	-0.645782	H	2.429601	-2.790852	-4.06161
H	8.723033	4.359478	0.54779	H	0.735863	-1.164371	-4.866024
H	7.183331	7.612975	-1.783838	N	6.26109	-5.158729	-1.110999
H	9.125505	6.373509	-0.84725	C	6.214823	-6.123643	-0.109372
C	3.438361	5.639479	1.276486	C	6.874384	-5.712129	-2.230782
C	4.017285	6.84372	1.67059	C	5.671229	-6.039575	1.170512
C	2.252675	5.250368	1.913449	C	6.81212	-7.309631	-0.585251
C	3.425346	7.638613	2.650493	C	7.152499	-5.124904	-3.462766
H	4.944873	7.174724	1.223104	C	7.235855	-7.04534	-1.944956
C	1.657611	6.040014	2.885318	C	5.74476	-7.166644	1.975575
H	1.786678	4.308756	1.653051	H	5.209487	-5.124483	1.524382
C	2.242802	7.246012	3.259595	C	6.87549	-8.42762	0.245095
H	3.903362	8.570364	2.936916	C	7.793711	-5.903344	-4.414776
H	0.736046	5.703032	3.350083	H	6.880213	-4.095293	-3.666025
H	1.784217	7.866796	4.022904	C	7.87969	-7.806349	-2.919654
C	3.201649	4.863391	-1.134446	C	6.342555	-8.350187	1.522755
C	3.75241	4.444885	-2.353867	H	5.328306	-7.129046	2.977354
C	1.888584	5.332214	-1.153427	H	7.332802	-9.347432	-0.10699
C	3.0077	4.440842	-3.52484	C	8.15351	-7.231913	-4.151254
H	4.785901	4.118576	-2.388626	H	8.022667	-5.471446	-5.384024
C	1.138345	5.3377	-2.327678	H	8.16384	-8.834163	-2.714679
H	1.43253	5.704434	-0.246514	H	6.385059	-9.213395	2.178777
C	1.68803	4.881693	-3.517115	H	8.652853	-7.813501	-4.919219
H	3.464252	4.096048	-4.447129	C	4.036219	4.741056	0.167989
H	0.116391	5.70372	-2.302812	C	-3.877777	-1.531877	1.769431
H	1.104908	4.885794	-4.432923	C	-2.74259	-0.728648	1.906026
N	3.850768	-0.16159	-0.777275	C	-1.707828	-1.077592	2.761854

C	-1.821025	-2.283994	3.464646		C	-4.106757	1.73911	-2.076713
C	-2.950898	-3.08083	3.347693		C	-7.729498	1.870318	-2.500616
C	-3.990734	-2.70812	2.503661		C	-5.652631	2.864132	-3.353947
H	-2.697081	0.179208	1.32349		C	-2.85853	1.408992	-1.64058
H	-1.004178	-2.606939	4.101707		C	-4.304038	2.751183	-3.098716
H	-3.013978	-4.008338	3.907574		C	-8.439517	2.737501	-3.384328
H	-4.872113	-3.327853	2.387981		H	-8.266356	1.194154	-1.855544
C	-0.081884	0.175317	4.310425		C	-6.399567	3.717606	-4.245171
C	1.091672	0.90911	4.524663		C	-1.743856	2.09194	-2.21843
C	-0.926476	-0.040067	5.394665		H	-2.701583	0.66041	-0.882093
C	1.424301	1.380778	5.783644		C	-3.15772	3.434474	-3.649602
H	1.74203	1.122269	3.683106		C	-7.76757	3.635553	-4.240466
C	-0.597351	0.438597	6.662754		H	-9.522512	2.693218	-3.393392
H	-1.857801	-0.57595	5.261534		H	-5.874719	4.408346	-4.895329
C	0.578922	1.143204	6.865694		C	-1.908634	3.088213	-3.199769
H	2.340689	1.947363	5.920484		H	-0.753755	1.832137	-1.871616
H	-1.275115	0.257996	7.491454		H	-3.299427	4.199224	-4.404864
H	0.833641	1.51513	7.853094		H	-8.349256	4.270126	-4.901659
C	-0.508247	1.069947	2.090123		H	-1.027994	3.584441	-3.595522
C	0.374551	1.448054	1.083317		N	-8.261965	-1.953615	0.043079
C	-1.495898	1.984576	2.475918		C	-8.797928	-2.691836	1.127599
C	0.250028	2.685958	0.457274		C	-9.223088	-1.933589	-0.99645
H	1.174897	0.786585	0.781905		C	-8.266901	-2.977258	2.383327
C	-1.630971	3.211226	1.844619		C	-10.092758	-3.112652	0.778697
H	-2.169755	1.725939	3.286252		C	-9.154468	-1.407527	-2.284083
C	-0.757442	3.566545	0.821307		C	-10.35784	-2.641548	-0.565665
H	0.953509	2.96624	-0.318125		C	-9.054779	-3.701497	3.269258
H	-2.418924	3.890978	2.153134		H	-7.274857	-2.650962	2.653152
H	-0.857488	4.522017	0.316569		C	-10.864337	-3.840265	1.679689
C	0.672604	-1.207948	2.299021		C	-10.257715	-1.573973	-3.112049
C	1.664494	-1.788817	3.086048		H	-8.276305	-0.885221	-2.629454
C	0.579192	-1.609477	0.96095		C	-11.453681	-2.795996	-1.409574
C	2.547706	-2.726039	2.549706		C	-10.338287	-4.135414	2.92763
H	1.752655	-1.520072	4.131454		H	-8.655535	-3.935475	4.251137
C	1.462142	-2.531526	0.422189		H	-11.863415	-4.165524	1.406702
H	-0.205524	-1.199224	0.333817		C	-11.401645	-2.252421	-2.683892
C	2.456959	-3.096608	1.216186		H	-10.22129	-1.164823	-4.11673
H	3.308172	-3.166875	3.187872		H	-12.330428	-3.341024	-1.073765
H	1.372873	-2.810487	-0.621817		H	-10.924495	-4.702725	3.643139
H	3.147078	-3.819208	0.792969		H	-12.247374	-2.361696	-3.354886
C	-4.940939	-1.117367	0.829406		N	-6.857296	-0.343703	-0.859607
C	-7.03223	-1.32429	0.023332		N	-4.653585	-0.137636	-0.024583
C	-5.643978	0.215911	-0.846378		N	-6.114137	-1.748958	0.893211
N	-5.393751	1.230513	-1.737727		C	-0.403698	-0.258677	2.8668
C	-6.366327	1.926058	-2.505975					

Supplementary Table 19. Atomic coordinates of CCzCz:CTrzCz2 (10, E = -4271.36709025 a.u.).

C	-4.557594	0.033069	-1.574303		C	-6.953972	0.517288	-1.061372
C	-3.552707	-0.602235	-0.861441		C	-6.406539	-1.646646	-1.41898
C	-2.205856	-0.28108	-1.058527		C	-6.938256	1.8959	-0.858585
C	-1.899097	0.738543	-1.95314		C	-8.141013	-0.232363	-0.922058
C	-2.910813	1.394678	-2.654674		C	-5.744437	-2.841223	-1.696301
C	-4.238303	1.03871	-2.488657		C	-7.791045	-1.616953	-1.154785
H	-3.82484	-1.357509	-0.133358		C	-8.130634	2.515077	-0.522121
H	-0.870098	1.031798	-2.112658		H	-6.02002	2.465628	-0.937594
H	-2.649646	2.184502	-3.35183		C	-9.334684	0.411291	-0.60932
H	-5.025452	1.521754	-3.056907		C	-6.494614	-4.007082	-1.695076
C	-1.641384	-0.960782	1.224323		H	-4.680604	-2.866794	-1.896058
C	-1.863421	0.32133	1.745417		C	-8.52401	-2.802979	-1.160982
C	-1.967805	-2.050251	2.027198		C	-9.323262	1.784524	-0.406767
C	-2.358178	0.506661	3.026422		H	-8.154594	3.582688	-0.332586
H	-1.660062	1.187784	1.124749		H	-10.265239	-0.139171	-0.518324
C	-2.481566	-1.867369	3.309469		C	-7.870654	-3.994706	-1.43015
H	-1.84267	-3.059485	1.660232		H	-5.999239	-4.950452	-1.902384
C	-2.672251	-0.593519	3.819281		H	-9.590687	-2.789914	-0.958477
H	-2.51516	1.513812	3.400274		H	-8.426181	-4.926626	-1.43573
H	-2.726184	-2.737883	3.90827		N	-10.523734	2.457788	-0.064433
H	-3.070107	-0.454441	4.819652		C	-11.12297	3.477154	-0.79699
C	0.257155	-0.486988	-0.437556		C	-11.306251	2.200756	1.057096
C	0.873693	-0.632532	-1.686263		C	-10.718472	4.05248	-1.999447
C	0.94874	0.212162	0.550596		C	-12.303478	3.883737	-0.140068
C	2.098081	-0.039165	-1.959109		C	-11.098495	1.282961	2.084186
H	0.384427	-1.216736	-2.458674		C	-12.421036	3.065065	1.048664
C	2.17983	0.805703	0.28023		C	-11.51363	5.057023	-2.530472
H	0.53224	0.304052	1.544773		H	-9.816437	3.721809	-2.501888
C	2.754118	0.698434	-0.978352		C	-13.084678	4.896465	-0.694887
H	2.546274	-0.161512	-2.940179		C	-12.037968	1.235704	3.103162
H	2.691556	1.356326	1.063223		H	-10.231006	0.632692	2.086112
H	3.709161	1.164958	-1.189622		C	-13.351323	2.996763	2.084993
C	-1.110891	-2.537604	-0.723331		C	-12.6845	5.480148	-1.886958
C	-0.267595	-3.442187	-0.069131		H	-11.222071	5.522974	-3.466647
C	-1.845436	-3.004581	-1.812179		H	-13.995729	5.220105	-0.200388
C	-0.229113	-4.781232	-0.431902		C	-13.156464	2.080232	3.106552
H	0.356718	-3.092095	0.745413		H	-11.90105	0.529308	3.916038
C	-1.792454	-4.342796	-2.19694		H	-14.214943	3.655083	2.091293
H	-2.466219	-2.323697	-2.379824		H	-13.282946	6.26985	-2.329321
C	-1.002304	-5.240997	-1.495245		H	-13.873555	2.01579	3.918402
H	0.407638	-5.466757	0.113457		C	-1.157993	-1.073413	-0.241371
H	-2.379779	-4.675701	-3.047582		C	5.324693	0.494217	2.111447
H	-0.975608	-6.289423	-1.776274		C	5.938062	1.391967	1.248221
N	-5.906993	-0.344897	-1.369944		C	6.450068	2.610805	1.685349

C	6.308898	2.924473	3.034262		C	5.597191	-1.416549	-2.436121
C	5.685095	2.032334	3.911174		C	2.719302	-3.657766	-2.303258
C	5.189671	0.818498	3.463165		C	4.179947	-2.487459	-3.894457
H	5.986749	1.134617	0.199382		C	6.662944	-0.642458	-2.078396
H	6.669208	3.871984	3.415313		C	5.250945	-1.622672	-3.829726
H	5.584145	2.302792	4.957438		C	1.970747	-4.125726	-3.425588
H	4.69894	0.123098	4.134736		H	2.406577	-3.907558	-1.30325
C	7.517272	4.879959	1.189934		C	3.421299	-3.003621	-5.008271
C	7.013047	6.115815	0.794455		C	7.409714	-0.000765	-3.112859
C	8.56199	4.864307	2.122342		H	6.942142	-0.501987	-1.04747
C	7.516825	7.298992	1.332557		C	6.013926	-0.947977	-4.851801
H	6.225853	6.169085	0.053483		C	2.335595	-3.798657	-4.749046
C	9.059131	6.038285	2.666107		H	1.092456	-4.731138	-3.239082
H	8.997308	3.913676	2.41383		H	3.709517	-2.746985	-6.021397
C	8.534041	7.267215	2.274072		C	7.067318	-0.155725	-4.472418
H	7.106632	8.249049	1.004199		H	8.260138	0.610655	-2.831981
H	9.866517	5.995132	3.390544		H	5.748582	-1.077529	-5.894977
H	8.924175	8.189118	2.693588		H	1.741169	-4.185764	-5.570717
C	5.904914	3.701976	-0.427131		H	7.655501	0.357597	-5.226757
C	5.986405	3.248182	-1.741294		N	2.525828	-3.331805	2.290727
C	4.684049	4.235011	0.006325		C	1.720671	-2.950941	3.38633
C	4.895695	3.346847	-2.60415		C	2.305585	-4.699648	2.028283
H	6.895539	2.789927	-2.106246		C	1.555329	-1.707852	3.992039
C	3.600755	4.344862	-0.851642		C	0.984427	-4.072289	3.805962
H	4.581475	4.554156	1.038695		C	2.902946	-5.545382	1.096981
C	3.702839	3.902708	-2.168652		C	1.357936	-5.181141	2.949562
H	4.9881	2.966903	-3.616959		C	0.659281	-1.622797	5.048045
H	2.667057	4.761112	-0.486787		H	2.118814	-0.84923	3.658552
H	2.850663	3.971462	-2.836994		C	0.09769	-3.966043	4.874843
C	8.296456	2.909084	-0.052881		C	2.510072	-6.877903	1.089838
C	8.967567	3.649873	-1.033458		H	3.631665	-5.17423	0.391628
C	8.8142	1.662599	0.289295		C	0.970811	-6.518182	2.917672
C	10.087506	3.146762	-1.676601		C	-0.055157	-2.73794	5.497434
H	8.599767	4.636932	-1.29435		H	0.51062	-0.663577	5.53292
C	9.945621	1.155671	-0.349379		H	-0.464626	-4.832559	5.209102
H	8.344522	1.070169	1.063549		C	1.547512	-7.363136	1.981105
C	10.582162	1.888413	-1.339916		H	2.959093	-7.551932	0.367427
H	10.581191	3.741727	-2.438709		H	0.233554	-6.891859	3.621316
H	10.32567	0.180377	-0.061572		H	-0.742522	-2.635165	6.330755
H	11.46121	1.492378	-1.838429		H	1.256271	-8.407628	1.942891
C	4.767941	-0.742868	1.528031		N	3.603992	-2.81014	0.311418
C	3.382656	-2.503403	1.586131		N	5.136659	-1.018289	0.283042
C	4.474083	-2.00944	-0.31042		N	3.899562	-1.462509	2.240425
N	4.677716	-2.177019	-1.656334		C	7.046359	3.535739	0.603634
C	3.796034	-2.85766	-2.542872					

Supplementary Table 20. Atomic coordinates of CCzCz:CTrzCz2 (11, E = -4271.38313321 a.u.).

C	-1.38468	3.707164	2.74729		C	-3.506333	2.533266	2.234199
C	-0.522407	3.538085	1.663343		C	-3.568562	4.783646	2.150632
C	0.854341	3.518474	1.843239		C	-3.105312	1.209427	2.371477
C	1.342662	3.641731	3.151517		C	-4.781937	2.878451	1.74215
C	0.48818	3.820919	4.22756		C	-3.247965	6.137115	2.193584
C	-0.890999	3.857534	4.032735		C	-4.824213	4.326913	1.693824
H	-0.958135	3.414312	0.680657		C	-3.989769	0.225458	1.970112
H	2.413114	3.590827	3.320934		H	-2.11949	0.953773	2.741472
H	0.895979	3.920374	5.22823		C	-5.675946	1.871197	1.384466
H	-1.578783	3.983228	4.861813		C	-4.217637	7.03759	1.778596
C	2.676771	2.064384	1.074647		H	-2.274193	6.468479	2.537578
C	4.065403	2.035142	1.16574		C	-5.782731	5.253953	1.285935
C	1.974217	0.893779	1.391232		C	-5.261962	0.54831	1.477153
C	4.731523	0.877903	1.567183		H	-3.689636	-0.812278	2.004708
H	4.64675	2.917399	0.934118		H	-6.668651	2.105279	1.015731
C	2.632262	-0.260178	1.785847		C	-5.474058	6.604807	1.331772
H	0.890059	0.900319	1.336467		H	-3.997562	8.100319	1.800221
C	4.021574	-0.268105	1.888841		H	-6.754147	4.920948	0.932962
H	5.815589	0.881836	1.628807		H	-6.210049	7.336882	1.016164
H	2.063206	-1.156229	2.015957		N	-6.107993	-0.49863	1.032512
H	4.541604	-1.160699	2.214044		C	-6.376272	-1.679058	1.719648
C	2.75312	4.562857	0.504525		C	-6.71085	-0.571982	-0.220942
C	3.68134	4.556812	-0.54349		C	-5.986469	-2.068263	3.000258
C	2.646645	5.718194	1.274225		C	-7.176774	-2.512365	0.90988
C	4.499459	5.647912	-0.786365		C	-6.663747	0.332184	-1.28055
H	3.763684	3.681591	-1.177734		C	-7.403391	-1.796911	-0.327307
C	3.458746	6.823519	1.023305		C	-6.389471	-3.318246	3.445448
H	1.921224	5.776745	2.075243		H	-5.388801	-1.41677	3.626402
C	4.393135	6.792425	-0.00014		C	-7.569981	-3.76419	1.380662
H	5.220529	5.601243	-1.595968		C	-7.354981	0.004676	-2.437135
H	3.351025	7.712129	1.637862		H	-6.080645	1.242286	-1.220785
H	5.028203	7.651771	-0.191509		C	-8.104947	-2.094398	-1.494996
C	1.134425	3.126522	-0.658013		C	-7.168956	-4.16497	2.64536
C	1.215668	1.96218	-1.415715		H	-6.095828	-3.644444	4.438449
C	0.383909	4.192595	-1.170404		H	-8.181155	-4.415167	0.762704
C	0.536725	1.85068	-2.627338		C	-8.082315	-1.188567	-2.544718
H	1.817886	1.128775	-1.077382		H	-7.320171	0.688853	-3.278754
C	-0.307295	4.077359	-2.366442		H	-8.653491	-3.02787	-1.581794
H	0.3405	5.125215	-0.616809		H	-7.464203	-5.138243	3.023498
C	-0.238842	2.896648	-3.100529		H	-8.621026	-1.408124	-3.460783
H	0.618343	0.934739	-3.194928		C	1.859953	3.318024	0.689812
H	-0.898993	4.913151	-2.727022		C	1.449146	-1.770787	-2.731839
H	-0.790237	2.792579	-4.029324		C	0.207446	-1.887943	-2.106852
N	-2.787989	3.689094	2.50609		C	-0.967993	-1.494083	-2.731342

C	-0.870372	-1.109911	-4.074913		C	2.35315	-4.489296	1.375581
C	0.352767	-1.062727	-4.73216		C	5.786899	-3.477938	2.110813
C	1.53188	-1.349274	-4.053592		C	3.945983	-4.83316	2.998702
H	0.205284	-2.252119	-1.092849		C	1.12949	-4.628722	0.788442
H	-1.760206	-0.829787	-4.621773		C	2.650726	-5.132379	2.642334
H	0.386398	-0.761175	-5.774148		C	6.539447	-3.845683	3.266649
H	2.500467	-1.249732	-4.530325		H	6.211617	-2.814411	1.377956
C	-3.003144	-0.124002	-2.23137		C	4.735821	-5.194575	4.150282
C	-2.901411	0.903985	-1.293331		C	0.141199	-5.40984	1.463795
C	-3.660614	0.172251	-3.432217		H	0.90011	-4.168486	-0.156692
C	-3.39912	2.177666	-1.550967		C	1.631619	-5.914194	3.299504
H	-2.443899	0.729207	-0.332695		C	6.007567	-4.696053	4.258435
C	-4.155893	1.442287	-3.697213		H	7.54414	-3.453088	3.374008
H	-3.81403	-0.606983	-4.169029		H	4.315009	-5.845945	4.907755
C	-4.021114	2.459549	-2.758616		C	0.40442	-6.032243	2.700165
H	-3.29944	2.948157	-0.793658		H	-0.835342	-5.507505	1.005334
H	-4.657586	1.630934	-4.641669		H	1.849014	-6.390229	4.248891
H	-4.406512	3.45393	-2.961547		H	6.618144	-4.952886	5.11827
C	-2.185325	-1.837125	-0.507215		H	-0.378348	-6.612864	3.177999
C	-2.943082	-2.827717	0.121043		N	5.885101	-0.701376	-1.583674
C	-1.239348	-1.163359	0.280973		C	5.844198	0.608721	-2.125016
C	-2.744362	-3.15932	1.459822		C	7.168707	-0.913659	-1.040717
H	-3.701879	-3.363722	-0.432108		C	4.817035	1.294275	-2.769509
C	-1.022988	-1.50384	1.609508		C	7.08743	1.222244	-1.887682
H	-0.631961	-0.379994	-0.158197		C	7.732554	-2.068232	-0.507009
C	-1.76941	-2.513897	2.207456		C	7.92723	0.257201	-1.204455
H	-3.3642	-3.927168	1.911627		C	5.070778	2.595223	-3.186642
H	-0.266401	-0.975208	2.180798		H	3.86309	0.825786	-2.952719
H	-1.594191	-2.78831	3.242565		C	7.319653	2.527781	-2.310688
C	-3.193898	-2.637503	-2.660558		C	9.052791	-2.006923	-0.079471
C	-4.589968	-2.597138	-2.637502		H	7.166261	-2.985203	-0.437636
C	-2.577553	-3.775905	-3.182367		C	9.249304	0.299833	-0.769821
C	-5.342058	-3.652316	-3.140454		C	6.307055	3.210231	-2.96739
H	-5.100946	-1.739518	-2.217323		H	4.282718	3.139854	-3.697337
C	-3.327788	-4.833614	-3.683167		H	8.281163	2.998651	-2.131298
H	-1.496442	-3.844555	-3.196512		C	9.804334	-0.833639	-0.195108
C	-4.715373	-4.77457	-3.66906		H	9.508565	-2.896018	0.344663
H	-6.423559	-3.586123	-3.110931		H	9.837227	1.20436	-0.890701
H	-2.820675	-5.705356	-4.085314		H	6.475998	4.222827	-3.319519
H	-5.3041	-5.597055	-4.063511		H	10.832702	-0.81691	0.150484
C	2.65381	-2.010068	-1.911531		N	4.799382	-2.358945	-0.362546
C	4.777609	-1.50583	-1.384159		N	2.541758	-2.84021	-0.881782
C	3.637784	-2.980081	-0.131289		N	3.752292	-1.319603	-2.216013
N	3.531099	-3.783681	0.976938		C	-2.350294	-1.518461	-2.016084
C	4.522648	-3.974702	1.979895					

Supplementary Table 21. Atomic coordinates of CCzCz:CTrzCz2 (12, E = -4271.34182102 a.u.).

C	8.615744	0.294402	0.801728		C	6.361477	-0.253399	-0.096255
C	9.892408	0.237392	0.239797		C	7.415557	1.543501	-1.010938
C	11.014408	0.042528	1.034227		C	6.005389	-1.378503	0.609378
C	10.822491	-0.151954	2.408138		C	5.501695	0.312552	-1.137103
C	9.554879	-0.102631	2.966319		C	8.293471	2.546276	-1.29875
C	8.439299	0.13654	2.169976		C	6.158613	1.407619	-1.681195
H	9.983925	0.334697	-0.832644		C	4.763982	-1.972002	0.319222
H	11.680479	-0.349178	3.041786		H	6.66425	-1.814711	1.349847
H	9.431587	-0.244559	4.035148		C	4.23112	-0.297661	-1.389716
H	7.446595	0.203395	2.600279		C	7.923976	3.480933	-2.341796
C	13.01941	-1.377094	0.946817		H	9.233419	2.656564	-0.773478
C	14.214627	-1.502861	1.649268		C	5.813726	2.348229	-2.708881
C	12.272818	-2.538878	0.710652		C	3.888807	-1.411188	-0.669388
C	14.662734	-2.749885	2.082593		H	4.468833	-2.891887	0.807207
H	14.807117	-0.626438	1.876456		H	3.554412	0.121171	-2.123947
C	12.719358	-3.781226	1.132608		C	6.718425	3.367284	-3.012641
H	11.322406	-2.462491	0.192249		H	8.614171	4.279114	-2.590727
C	13.923791	-3.893562	1.822686		H	4.868058	2.263125	-3.231741
H	15.597002	-2.816738	2.631536		H	6.471114	4.083032	-3.790138
H	12.120725	-4.663889	0.929973		N	2.661882	-2.065602	-0.901782
H	14.274116	-4.863792	2.160366		C	1.732806	-2.408246	0.082865
C	13.303185	1.162488	0.949084		C	2.134257	-2.417186	-2.146357
C	14.585153	1.319296	0.406033		C	1.761629	-2.151618	1.452218
C	12.859779	2.119985	1.856931		C	0.621494	-3.019472	-0.529504
C	15.402605	2.373906	0.779964		C	2.683737	-2.270217	-3.417709
H	14.939013	0.604056	-0.329333		C	0.874628	-3.020031	-1.954352
C	13.676064	3.187694	2.228607		C	0.667903	-2.550864	2.206716
H	11.86599	2.052957	2.279881		H	2.599875	-1.638194	1.908578
C	14.950304	3.317511	1.699197		C	-0.451451	-3.439032	0.253013
H	16.39293	2.46576	0.344846		C	1.933866	-2.712	-4.4987
H	13.301299	3.921303	2.935753		H	3.662654	-1.828032	-3.557735
H	15.585072	4.148694	1.989525		C	0.139038	-3.452132	-3.055158
C	12.432104	0.071689	-1.078682		C	-0.423635	-3.201959	1.619063
C	12.782543	-0.974405	-1.926606		H	0.650048	-2.338335	3.270315
C	12.065758	1.2926	-1.66067		H	-1.303949	-3.931393	-0.206131
C	12.74326	-0.816386	-3.311645		C	0.670451	-3.290715	-4.325406
H	13.097636	-1.925113	-1.516982		H	2.338602	-2.604312	-5.500153
C	12.011873	1.449249	-3.036152		H	-0.838331	-3.904627	-2.91818
H	11.823922	2.133568	-1.018508		H	-1.263186	-3.501814	2.236709
C	12.350325	0.388473	-3.872952		H	0.106672	-3.61747	-5.19294
H	13.024673	-1.649149	-3.948742		C	12.446815	-0.027094	0.463008
H	11.710081	2.403427	-3.456645		C	-6.206894	-0.928641	0.079119
H	12.314087	0.507576	-4.951102		C	-5.260226	0.045574	0.416347
N	7.50476	0.515171	-0.0461		C	-3.9107	-0.264798	0.495776

C	-3.520232	-1.57593	0.192777		C	-8.772175	3.428021	-0.45136
C	-4.451387	-2.547898	-0.13874		C	-12.042945	2.22531	0.686456
C	-5.803821	-2.232913	-0.19097		C	-10.879212	4.247657	-0.006547
H	-5.615683	1.045757	0.618071		C	-7.437811	3.450834	-0.848597
H	-2.466589	-1.827624	0.217937		C	-9.541734	4.604208	-0.436142
H	-4.119881	-3.556507	-0.366312		C	-13.173207	3.004289	0.902662
H	-6.545502	-2.976088	-0.458594		H	-12.058232	1.163711	0.87593
C	-2.239218	0.433493	2.277323		C	-12.021918	5.01022	0.214656
C	-1.363955	1.355104	2.866557		C	-6.897578	4.673517	-1.227576
C	-2.565168	-0.706454	3.007129		H	-6.83897	2.554118	-0.854233
C	-0.803721	1.125148	4.113303		C	-8.978532	5.8203	-0.809674
H	-1.124965	2.270918	2.336377		C	-13.172553	4.380812	0.664758
C	-2.015753	-0.935151	4.267792		H	-14.075419	2.522832	1.266273
H	-3.261923	-1.430129	2.60563		H	-12.006306	6.081937	0.042399
C	-1.125853	-0.028835	4.823302		C	-7.650512	5.849547	-1.207022
H	-0.122212	1.855911	4.537734		H	-5.857711	4.705594	-1.532076
H	-2.29608	-1.830592	4.814605		H	-9.575377	6.727048	-0.795981
H	-0.697758	-0.207605	5.804771		H	-14.073492	4.959442	0.840733
C	-3.38157	2.186279	0.958474		H	-7.194107	6.788022	-1.504952
C	-3.036567	3.198406	0.066766		N	-10.771068	-2.143905	-0.114588
C	-4.259643	2.503984	2.002656		C	-10.586936	-3.501864	0.25068
C	-3.559535	4.483154	0.206567		C	-12.124826	-1.968252	-0.497333
H	-2.343753	2.997869	-0.740075		C	-9.461482	-4.171734	0.725001
C	-4.796929	3.775119	2.132321		C	-11.817258	-4.166827	0.113407
H	-4.519562	1.739352	2.727547		C	-12.791059	-0.84952	-0.99167
C	-4.446341	4.776429	1.231436		C	-12.785572	-3.20128	-0.365871
H	-3.26425	5.255963	-0.497129		C	-9.594918	-5.518168	1.041117
H	-5.487248	3.986756	2.942638		H	-8.518294	-3.661344	0.838365
H	-4.865765	5.772451	1.32888		C	-11.928949	-5.516678	0.432093
C	-1.725441	0.652579	-0.21565		C	-14.134763	-0.986922	-1.317721
C	-0.363012	0.572852	0.054452		H	-12.286215	0.095173	-1.114574
C	-2.126839	0.645171	-1.558365		C	-14.13211	-3.318125	-0.69676
C	0.569958	0.511851	-0.978223		C	-10.810051	-6.191609	0.894748
H	-0.007503	0.532261	1.074561		H	-8.726966	-6.055748	1.409713
C	-1.20178	0.566285	-2.588024		H	-12.880495	-6.027742	0.323198
H	-3.18441	0.693059	-1.796511		C	-14.806735	-2.202435	-1.168137
C	0.159013	0.501087	-2.30154		H	-14.668423	-0.121818	-1.698235
H	1.624517	0.453465	-0.733328		H	-14.640671	-4.271463	-0.591847
H	-1.544733	0.548729	-3.617867		H	-10.877637	-7.2447	1.147459
H	0.884958	0.420543	-3.104541		H	-15.857503	-2.274298	-1.429216
C	-7.636112	-0.560483	0.01467		N	-10.216699	0.108147	-0.080189
C	-9.805654	-1.159064	-0.072061		N	-7.940794	0.737079	0.008576
C	-9.242428	1.01698	-0.045112		N	-8.531384	-1.547892	-0.029087
N	-9.606577	2.346559	-0.065566		C	-2.814269	0.75171	0.877831
C	-10.897777	2.85997	0.212114					

Supplementary Table 22. Atomic coordinates of CCzCz:CTrzCz2 (13, E = -4271.35486381 a.u.).

C	-7.130988	-0.699843	-1.098392	C	-5.026186	-0.844883	0.228889
C	-8.350984	-0.043378	-0.933788	C	-5.597521	1.21136	-0.566993
C	-9.509362	-0.51446	-1.537328	C	-4.968488	-2.165035	0.596683
C	-9.427996	-1.698401	-2.281109	C	-4.006697	0.111572	0.644855
C	-8.222528	-2.36896	-2.422407	C	-6.201834	2.27	-1.176608
C	-7.058864	-1.866508	-1.850065	C	-4.358185	1.35278	0.149114
H	-8.368431	0.852271	-0.331344	C	-3.868941	-2.585911	1.382286
H	-10.31616	-2.084959	-2.768485	H	-5.743134	-2.868614	0.319661
H	-8.180514	-3.280046	-3.01067	C	-2.890291	-0.348053	1.421869
H	-6.105024	-2.358888	-1.999566	C	-5.556081	3.561611	-1.072963
C	-11.82034	-0.501254	-0.467326	H	-7.132313	2.175541	-1.721205
C	-12.991351	0.153392	-0.06413	C	-3.728132	2.647371	0.226149
C	-11.580068	-1.775986	0.037586	C	-2.844785	-1.674404	1.764893
C	-13.896597	-0.45188	0.793632	H	-3.807159	-3.610094	1.729094
H	-13.188249	1.157	-0.42652	H	-2.097502	0.332941	1.706809
C	-12.484627	-2.385091	0.905853	C	-4.354062	3.721334	-0.389499
H	-10.676519	-2.306898	-0.232482	H	-6.033467	4.41451	-1.541837
C	-13.646702	-1.730731	1.284715	H	-2.786495	2.762151	0.748706
H	-14.796801	0.079788	1.086047	H	-3.897984	4.705193	-0.33866
H	-12.26804	-3.377932	1.287919	N	-1.755427	-2.166994	2.525832
H	-14.349792	-2.205613	1.961667	C	-0.85023	-3.13903	2.101904
C	-11.449038	0.272515	-2.863733	C	-1.287179	-1.619984	3.718946
C	-12.759883	-0.083861	-3.168692	C	-0.874153	-3.927601	0.953288
C	-10.619303	0.671723	-3.920438	C	0.209723	-3.215657	3.028949
C	-13.233685	-0.025539	-4.478382	C	-1.870718	-0.651035	4.531747
H	-13.426739	-0.423925	-2.387647	C	-0.070652	-2.24331	4.065353
C	-11.08865	0.737659	-5.22313	C	0.184415	-4.800325	0.748588
H	-9.584348	0.925899	-3.716631	H	-1.690805	-3.858102	0.243509
C	-12.405732	0.389139	-5.510002	C	1.255378	-4.109884	2.805914
H	-14.259748	-0.314214	-4.684555	C	-1.20007	-0.299281	5.693778
H	-10.420785	1.054015	-6.018398	H	-2.811611	-0.187788	4.258609
H	-12.775734	0.433258	-6.529447	C	0.591197	-1.862039	5.231591
C	-10.642826	1.652901	-0.875272	C	1.24109	-4.895282	1.663841
C	-10.377969	1.806354	0.492138	H	0.188966	-5.4249	-0.1396
C	-10.662339	2.800454	-1.664081	H	2.073155	-4.180502	3.514837
C	-10.108364	3.050711	1.040927	C	0.02535	-0.88681	6.037714
H	-10.379034	0.931951	1.13426	H	-1.633526	0.450883	6.348431
C	-10.399501	4.054249	-1.114312	H	1.539648	-2.31495	5.496617
H	-10.892988	2.732613	-2.718667	H	2.056068	-5.585265	1.472816
C	-10.113558	4.186273	0.23584	H	0.532777	-0.57677	6.945141
H	-9.896118	3.133661	2.102204	C	-10.862703	0.226699	-1.433575
H	-10.424824	4.930286	-1.755185	C	5.675379	2.164114	0.354125
H	-9.904675	5.162332	0.662007	C	4.479838	1.544517	0.688581
N	-5.972764	-0.148001	-0.50575	C	3.424677	2.233719	1.27995

C	3.623346	3.575606	1.59046	C	5.775505	-2.567726	-1.278871
C	4.827819	4.208845	1.271366	C	9.400428	-2.967299	-1.676986
C	5.852029	3.519783	0.642921	C	7.326353	-4.239305	-1.603474
H	4.384081	0.487319	0.490094	C	4.514004	-1.990706	-1.141021
H	2.843196	4.143348	2.083127	C	5.916206	-3.957863	-1.419933
H	4.955682	5.258227	1.517302	C	10.077066	-4.16926	-1.848824
H	6.779273	4.011366	0.372325	H	9.935885	-2.032844	-1.628579
C	0.987635	2.259199	2.114641	C	8.022301	-5.431137	-1.777841
C	0.222449	1.9108	3.225975	C	3.410337	-2.834201	-1.097141
C	0.568887	3.349086	1.34291	H	4.387934	-0.923193	-1.072933
C	-0.903289	2.652685	3.577048	C	4.799422	-4.788411	-1.375728
H	0.488294	1.049274	3.825788	C	9.402607	-5.390722	-1.905619
C	-0.543501	4.09761	1.698178	H	11.158387	-4.148594	-1.940329
H	1.114019	3.605014	0.440586	H	7.489171	-6.376357	-1.807924
C	-1.285803	3.754804	2.825695	C	3.546725	-4.220952	-1.201809
H	-1.479863	2.353925	4.4458	H	2.42352	-2.397517	-0.982678
H	-0.839156	4.943731	1.084986	H	4.913799	-5.863147	-1.479161
H	-2.16159	4.333086	3.103079	H	9.961497	-6.310444	-2.044344
C	2.695114	0.413368	2.677481	H	2.664211	-4.849906	-1.155575
C	3.033835	-0.900065	2.36066	N	10.004782	1.622052	-1.509556
C	3.039806	0.894715	3.944961	C	10.592683	2.77992	-0.941121
C	3.710434	-1.703328	3.274357	C	10.913764	1.081404	-2.452062
H	2.798012	-1.308435	1.386375	C	10.126481	3.643621	0.04689
C	3.690961	0.089263	4.866286	C	11.865945	2.953654	-1.509666
H	2.813275	1.923969	4.203397	C	10.785717	-0.001644	-3.318388
C	4.039835	-1.216559	4.530734	C	12.065132	1.886695	-2.469724
H	3.98632	-2.711506	2.983344	C	10.955291	4.688158	0.436948
H	3.94524	0.489021	5.843144	H	9.152616	3.505841	0.489221
H	4.572086	-1.841669	5.241312	C	12.679277	4.008929	-1.108031
C	1.607934	0.701502	0.366622	C	11.84674	-0.278239	-4.171871
C	0.617553	-0.272493	0.522264	H	9.894754	-0.609459	-3.32811
C	1.957112	1.080231	-0.929036	C	13.117678	1.592175	-3.330961
C	0.008673	-0.85697	-0.578916	C	12.216767	4.878528	-0.132697
H	0.31886	-0.57498	1.520047	H	10.606614	5.37244	1.203991
C	1.351528	0.491601	-2.036263	H	13.661787	4.141698	-1.550207
H	2.707086	1.844907	-1.088337	C	13.006385	0.500765	-4.178612
C	0.375723	-0.479741	-1.867184	H	11.764007	-1.122654	-4.848687
H	-0.757613	-1.606696	-0.426947	H	14.007252	2.214295	-3.340521
H	1.646759	0.803398	-3.033301	H	12.836437	5.707623	0.192924
H	-0.100084	-0.937463	-2.728632	H	13.817919	0.254848	-4.855594
C	6.720091	1.347724	-0.298083	N	8.559107	-0.18997	-1.47166
C	8.77582	1.091379	-1.172464	N	6.399115	0.084414	-0.569863
C	7.353245	-0.648637	-1.133928	N	7.900255	1.902283	-0.573127
N	7.066107	-1.977442	-1.36425	C	2.167976	1.405869	1.621136
C	8.013091	-3.013315	-1.567722				

Supplementary Table 23. Atomic coordinates of CCzCz:CTrzCz2 (14, E = -4271.37630985 a.u.).

C	5.471668	0.524936	0.031908	C	3.056282	0.754775	0.583877
C	6.472308	-0.37939	0.39475	C	3.667788	-1.10997	-0.569514
C	7.809045	-0.003702	0.398327	C	3.001032	1.910353	1.324065
C	8.121681	1.325879	0.087406	C	1.862863	-0.047696	0.33034
C	7.128413	2.227243	-0.262038	C	4.349296	-2.06306	-1.26589
C	5.795281	1.832648	-0.30822	C	2.253317	-1.18778	-0.354558
H	6.17875	-1.377176	0.687031	C	1.743806	2.335468	1.792796
H	9.155264	1.652665	0.12604	H	3.893106	2.476412	1.560555
H	7.393881	3.250081	-0.510335	C	0.590807	0.445094	0.759497
H	5.014183	2.518557	-0.612247	C	3.600084	-3.199972	-1.755764
C	9.711381	-0.272497	1.933166	H	5.411712	-1.987488	-1.457216
C	11.079656	-0.017179	1.914452	C	1.532229	-2.327291	-0.847645
C	8.976607	0.174909	3.039109	C	0.546689	1.620619	1.46007
C	11.699768	0.642637	2.974622	H	1.671416	3.197512	2.442191
H	11.67843	-0.321511	1.066078	H	-0.311307	-0.085319	0.49089
C	9.591037	0.823658	4.098486	C	2.23661	-3.311509	-1.536208
H	7.903243	0.016391	3.061731	H	4.128565	-3.970561	-2.305776
C	10.963236	1.060258	4.072181	H	0.462545	-2.398766	-0.6917
H	12.768081	0.831067	2.930524	H	1.704127	-4.174823	-1.921949
H	8.995205	1.154134	4.943667	N	-0.686735	2.154039	1.887736
H	11.447441	1.573457	4.897021	C	-1.069037	3.499138	1.80431
C	9.875499	-1.253495	-0.421519	C	-1.76199	1.424777	2.408298
C	10.882856	-2.215348	-0.269938	C	-0.379051	4.598008	1.293793
C	9.73367	-0.651542	-1.668612	C	-2.383217	3.632426	2.296029
C	11.735135	-2.537672	-1.313977	C	-1.870388	0.063255	2.687778
H	10.989581	-2.724003	0.682708	C	-2.823065	2.310445	2.684707
C	10.583243	-0.980204	-2.724288	C	-1.019913	5.829028	1.304564
H	8.9502	0.075822	-1.836304	H	0.620017	4.49619	0.891241
C	11.590108	-1.917079	-2.552437	C	-3.004393	4.878616	2.302933
H	12.509347	-3.283759	-1.163853	C	-3.066844	-0.393201	3.222248
H	10.445867	-0.496795	-3.686699	H	-1.049409	-0.617722	2.505493
H	12.251456	-2.171636	-3.374617	C	-4.009243	1.830609	3.232544
C	8.393041	-2.343554	1.212225	C	-2.315914	5.975642	1.809182
C	8.468401	-2.842605	2.50892	H	-0.504642	6.69391	0.899346
C	7.792138	-3.14774	0.234676	H	-4.021897	4.979646	2.666565
C	7.936512	-4.092001	2.826492	C	-4.13162	0.474944	3.490511
H	8.948625	-2.264313	3.287335	H	-3.169471	-1.451939	3.439811
C	7.249128	-4.382851	0.549131	H	-4.830429	2.509154	3.436916
H	7.755524	-2.795209	-0.79116	H	-2.792027	6.949749	1.789767
C	7.317257	-4.862561	1.854881	H	-5.057612	0.082587	3.894889
H	8.011538	-4.456989	3.846197	C	8.951695	-0.96853	0.783006
H	6.774883	-4.974616	-0.227586	C	-3.543238	-1.772774	-0.879232
H	6.896914	-5.831179	2.105952	C	-4.851241	-1.857288	-0.415342
N	4.127478	0.091596	0.016585	C	-5.504059	-3.083817	-0.273314

C	-4.792221	-4.237122	-0.594794		C	-4.984834	3.029097	-0.102313
C	-3.470675	-4.164904	-1.034438		C	-2.351726	4.725072	-2.015656
C	-2.84148	-2.942039	-1.184567		C	-4.415965	5.147047	-0.807282
H	-5.378828	-0.937857	-0.2036		C	-5.731113	2.04688	0.541688
H	-5.265336	-5.207408	-0.52289		C	-5.398088	4.372025	-0.0787
H	-2.940448	-5.080092	-1.277929		C	-2.287409	6.086482	-2.287522
H	-1.824152	-2.872916	-1.550765		H	-1.597847	4.052897	-2.388338
C	-7.661753	-4.435516	-0.082367		C	-4.337684	6.505457	-1.092365
C	-8.642988	-4.611424	-1.054704		C	-6.889925	2.437079	1.202009
C	-7.306569	-5.548149	0.692357		H	-5.409318	1.020515	0.550821
C	-9.230591	-5.857704	-1.265307		C	-6.561594	4.74463	0.586265
H	-8.973157	-3.772947	-1.652993		C	-3.261277	6.975316	-1.829027
C	-7.882348	-6.791309	0.480067		H	-1.454781	6.458852	-2.875966
H	-6.57456	-5.431341	1.484642		H	-5.11011	7.18309	-0.741787
C	-8.850589	-6.954111	-0.506879		C	-7.309064	3.768319	1.228897
H	-9.996002	-5.960778	-2.028383		H	-7.472919	1.677436	1.712258
H	-7.581514	-7.633508	1.095581		H	-6.873065	5.784528	0.602698
H	-9.30912	-7.923984	-0.672024		H	-3.179936	8.032894	-2.058443
C	-7.670386	-1.920885	-0.538225		H	-8.218989	4.039461	1.754684
C	-8.362926	-0.896876	0.101004		N	0.036421	0.848247	-2.479811
C	-7.545559	-1.8533	-1.93256		C	0.635005	-0.173717	-3.252601
C	-8.922931	0.153481	-0.625331		C	0.972215	1.895376	-2.327855
H	-8.459231	-0.896538	1.178975		C	0.098846	-1.357594	-3.753509
C	-8.107041	-0.815109	-2.658399		C	1.947761	0.216943	-3.565204
H	-6.980679	-2.62064	-2.451948		C	0.870269	3.100224	-1.64225
C	-8.801434	0.199553	-2.004638		C	2.164932	1.52097	-2.971494
H	-9.442279	0.947108	-0.098006		C	0.915824	-2.15098	-4.547015
H	-7.990146	-0.790156	-3.737374		H	-0.914664	-1.651189	-3.525375
H	-9.230155	1.022545	-2.567244		C	2.756848	-0.602096	-4.346542
C	-7.045826	-2.882433	1.737867		C	1.978197	3.937514	-1.639835
C	-8.283891	-3.033704	2.376497		H	-0.035098	3.374618	-1.125405
C	-5.934201	-2.6029	2.525762		C	3.266677	2.370758	-2.951321
C	-8.407997	-2.894351	3.749476		C	2.235536	-1.790741	-4.832817
H	-9.159467	-3.275216	1.78261		H	0.517408	-3.079215	-4.944471
C	-6.052496	-2.482395	3.91047		H	3.776165	-0.307429	-4.574846
H	-4.959905	-2.483322	2.069862		C	3.164377	3.586355	-2.289875
C	-7.285684	-2.620574	4.527947		H	1.91312	4.888227	-1.119742
H	-9.381164	-3.012891	4.215764		H	4.190428	2.082074	-3.442921
H	-5.166795	-2.282692	4.505766		H	2.851596	-2.442354	-5.443669
H	-7.374677	-2.525101	5.605457		H	4.009059	4.268249	-2.27665
C	-2.928808	-0.447976	-1.115206		N	-1.83062	1.931914	-1.616711
C	-1.217264	0.786697	-1.900996		N	-3.625916	0.636661	-0.787389
C	-3.04393	1.803794	-1.071955		N	-1.71906	-0.428298	-1.677595
N	-3.759416	2.952083	-0.818766		C	-6.975152	-3.08039	0.209114
C	-3.419688	4.262484	-1.251858					

Supplementary Table 24. Atomic coordinates of CCzCz:CTrzCz2 (15, E = -4271.38693226 a.u.).

C	-3.938035	-0.181138	1.751469		C	-1.704237	0.818035	1.410069
C	-5.035434	-0.49955	0.964061		C	-1.916269	-1.381391	0.880356
C	-6.324062	-0.555107	1.513667		C	-1.907285	2.131872	1.741657
C	-6.481086	-0.22185	2.855703		C	-0.368121	0.270789	1.189591
C	-5.383459	0.132943	3.637868		C	-2.38421	-2.623446	0.58497
C	-4.110378	0.140222	3.099519		C	-0.511548	-1.072951	0.882348
H	-4.874752	-0.71051	-0.085823		C	-0.77532	2.960565	1.863612
H	-7.465514	-0.228945	3.304693		H	-2.903883	2.529174	1.889226
H	-5.531023	0.38941	4.682058		C	0.766548	1.141639	1.310786
H	-3.245438	0.390277	3.703578		C	-1.413521	-3.650174	0.275612
C	-7.607561	0.294657	-0.410469		H	-3.442065	-2.843749	0.544539
C	-7.716569	0.142067	-1.791239		C	0.434463	-2.122925	0.609956
C	-7.595973	1.600437	0.097691		C	0.542923	2.452282	1.639908
C	-7.836485	1.247924	-2.632361		H	-0.886708	3.99488	2.162109
H	-7.707897	-0.844979	-2.233226		H	1.770307	0.775705	1.130515
C	-7.726304	2.70292	-0.733658		C	-0.0491	-3.38913	0.303726
H	-7.487979	1.752718	1.165881		H	-1.772452	-4.640531	0.018959
C	-7.852241	2.532216	-2.1099		H	1.498369	-1.92694	0.661999
H	-7.912061	1.093183	-3.704017		H	0.654397	-4.182239	0.071501
H	-7.723039	3.700134	-0.304485		N	1.625249	3.345416	1.805754
H	-7.945941	3.392191	-2.764984		C	2.713585	3.163738	2.660649
C	-8.834888	-0.993317	1.407101		C	1.700303	4.629085	1.265781
C	-8.951171	-2.035875	2.335116		C	3.035704	2.074613	3.464798
C	-9.928183	-0.150784	1.22969		C	3.49661	4.336758	2.660234
C	-10.107919	-2.212764	3.076559		C	0.855425	5.250691	0.34812
H	-8.119419	-2.719616	2.470311		C	2.848346	5.273493	1.767151
C	-11.097664	-0.332209	1.967544		C	4.167176	2.174874	4.261532
H	-9.883366	0.655383	0.509004		H	2.422157	1.182248	3.468294
C	-11.192405	-1.356928	2.895745		C	4.637156	4.406193	3.456361
H	-10.167566	-3.026577	3.792563		C	1.16377	6.546226	-0.035142
H	-11.936589	0.337789	1.806403		H	-0.006876	4.734177	-0.055371
H	-12.102493	-1.496072	3.470624		C	3.141257	6.574295	1.361233
C	-7.372571	-2.252277	-0.095527		C	4.969579	3.322556	4.254477
C	-8.401852	-2.70012	-0.934518		H	4.431832	1.34504	4.910243
C	-6.317159	-3.12624	0.150246		H	5.251702	5.301304	3.45568
C	-8.340241	-3.93727	-1.556945		C	2.291964	7.207349	0.467551
H	-9.274165	-2.073279	-1.086194		H	0.528738	7.048086	-0.757461
C	-6.255361	-4.37599	-0.462982		H	4.024079	7.081048	1.739632
H	-5.535929	-2.848313	0.843226		H	5.852235	3.364569	4.884393
C	-7.256405	-4.78255	-1.331472		H	2.508162	8.219209	0.140497
H	-9.150052	-4.249845	-2.208789		C	-7.52292	-0.87877	0.59541
H	-5.414528	-5.029385	-0.251233		C	2.795443	-2.203594	-1.924317
H	-7.208125	-5.754148	-1.812826		C	3.959507	-2.297544	-1.176533
N	-2.621307	-0.201564	1.214291		C	4.59	-3.520613	-0.942303

C	4.039287	-4.656964	-1.525628		C	4.089124	2.604079	-0.818997
C	2.886308	-4.567907	-2.309916		C	1.64992	4.127679	-3.079996
C	2.249911	-3.354114	-2.499215		C	3.691784	4.625761	-1.844039
H	4.351872	-1.394032	-0.729185		C	4.70464	1.680471	0.021638
H	4.487017	-5.628212	-1.357804		C	4.586449	3.910628	-0.954939
H	2.47485	-5.469116	-2.753385		C	1.690812	5.423329	-3.580541
H	1.335826	-3.284393	-3.075773		H	0.86309	3.444972	-3.364343
C	6.247291	-4.95561	0.358463		C	3.713683	5.917666	-2.357867
C	6.141916	-5.555307	1.610031		C	5.843123	2.088052	0.702344
C	6.866537	-5.680275	-0.667726		H	4.316917	0.680549	0.144004
C	6.620202	-6.846803	1.826812		C	5.73227	4.297623	-0.26583
H	5.694072	-5.015677	2.434514		C	2.70733	6.313809	-3.225861
C	7.335221	-6.96764	-0.458176		H	0.912383	5.743858	-4.265844
H	6.990528	-5.216962	-1.641481		H	4.507459	6.603138	-2.079098
C	7.211691	-7.560699	0.796073		C	6.36053	3.37852	0.559292
H	6.528877	-7.288623	2.814344		H	6.334947	1.383732	1.364193
H	7.807922	-7.506754	-1.273362		H	6.113927	5.309105	-0.364244
H	7.582963	-8.566425	0.966014		H	2.708691	7.319739	-3.633082
C	5.201149	-2.814196	1.305585		H	7.250032	3.664195	1.110922
C	5.75011	-1.654611	1.845259		N	-1.134356	0.363907	-2.352592
C	4.005411	-3.291846	1.859033		C	-1.989975	-0.752638	-2.557619
C	5.128526	-0.992479	2.903162		C	-1.944392	1.488346	-2.029518
H	6.659061	-1.239088	1.429563		C	-1.705867	-2.075572	-2.882379
C	3.388181	-2.641862	2.916458		C	-3.317514	-0.328384	-2.382731
H	3.537849	-4.173945	1.433157		C	-1.606282	2.802361	-1.714998
C	3.94893	-1.480818	3.443613		C	-3.287653	1.076542	-2.0328
H	5.563352	-0.075935	3.286553		C	-2.771363	-2.958252	-3.014283
H	2.455476	-3.030032	3.313444		H	-0.689109	-2.408342	-2.997617
H	3.462833	-0.961578	4.263495		C	-4.372014	-1.223399	-2.533175
C	6.994937	-2.767267	-0.500912		C	-2.636631	3.678838	-1.394146
C	8.183572	-2.803919	0.23894		H	-0.579877	3.126898	-1.699606
C	6.993231	-2.058232	-1.698579		C	-4.303849	1.963467	-1.697472
C	9.318786	-2.133958	-0.189009		C	-4.093905	-2.546322	-2.842058
H	8.213971	-3.370174	1.164352		H	-2.559985	-3.995524	-3.254747
C	8.134994	-1.387908	-2.134848		H	-5.394043	-0.885535	-2.408152
H	6.101914	-2.019765	-2.310935		C	-3.971203	3.270452	-1.375678
C	9.299009	-1.41695	-1.382817		H	-2.385109	4.704838	-1.142516
H	10.224239	-2.176078	0.408545		H	-5.334638	1.634639	-1.684324
H	8.103026	-0.840347	-3.071521		H	-4.905757	-3.25831	-2.944938
H	10.186278	-0.892134	-1.722544		H	-4.754112	3.974099	-1.111635
C	2.114303	-0.893989	-2.001492		N	0.883726	1.464693	-2.059112
C	0.236358	0.315783	-2.256112		N	2.832548	0.184377	-1.683409
C	2.178451	1.341005	-1.783199		N	0.825566	-0.881017	-2.328105
N	2.903949	2.497698	-1.582086		C	5.769553	-3.523525	0.053386
C	2.656876	3.743311	-2.199977					

Supplementary Table 25. Atomic coordinates of CCzCz:CTrzCz2 (16, E = -4271.38468648 a.u.).

C	-0.398972	4.168349	0.329472	C	-2.674053	3.400522	-0.300468
C	0.720916	3.428991	0.687453	C	-1.007779	2.926703	-1.753548
C	1.638575	3.907019	1.626708	C	-3.415631	3.806383	0.807283
C	1.394209	5.153889	2.19854	C	-3.226046	2.563117	-1.293585
C	0.276854	5.901421	1.83281	C	0.212231	2.846184	-2.420525
C	-0.619452	5.424999	0.89107	C	-2.15934	2.266745	-2.229627
H	0.889988	2.471934	0.208751	C	-4.719073	3.354214	0.912314
H	2.087241	5.569066	2.917131	H	-2.982689	4.431691	1.578097
H	0.12124	6.879249	2.27717	C	-4.537771	2.112542	-1.165602
H	-1.472149	6.015836	0.575585	C	0.260644	2.079708	-3.574334
C	3.864294	3.866886	2.823734	H	1.096864	3.353652	-2.053581
C	5.04546	4.445788	2.369744	C	-2.082975	1.495583	-3.389126
C	3.53173	4.024083	4.175262	C	-5.273749	2.505402	-0.055474
C	5.867552	5.166482	3.234991	H	-5.328454	3.637754	1.763418
H	5.341829	4.333437	1.334814	H	-4.971768	1.42434	-1.88351
C	4.343097	4.745847	5.036636	C	-0.870637	1.407286	-4.055041
H	2.623244	3.566246	4.552873	H	1.20246	2.000594	-4.105715
C	5.521177	5.322978	4.568234	H	-2.956099	0.966137	-3.757219
H	6.786262	5.602804	2.855027	H	-0.794083	0.805972	-4.954441
H	4.058949	4.852671	6.079023	N	-6.585538	2.003356	0.147951
H	6.162505	5.883506	5.241164	C	-7.70607	2.27591	-0.626472
C	3.576384	2.800292	0.540896	C	-6.952696	1.13355	1.171314
C	4.224754	1.605251	0.238297	C	-7.819735	3.08738	-1.752479
C	3.590509	3.812611	-0.427853	C	-8.81254	1.577504	-0.095123
C	4.878385	1.433883	-0.979217	C	-6.152734	0.549321	2.15046
H	4.232116	0.790854	0.950856	C	-8.327481	0.842635	1.056164
C	4.222137	3.63486	-1.651453	C	-9.067645	3.184645	-2.349031
H	3.102696	4.758309	-0.218589	H	-6.960552	3.620184	-2.144794
C	4.874732	2.439314	-1.935219	C	-10.056016	1.6919	-0.716596
H	5.394423	0.501654	-1.173142	C	-6.760524	-0.337292	3.025206
H	4.209519	4.437674	-2.382483	H	-5.091628	0.760363	2.212581
H	5.37105	2.29325	-2.888801	C	-8.914884	-0.048391	1.954582
C	2.578357	1.740818	2.632112	C	-10.176974	2.49457	-1.840407
C	3.620469	1.056525	3.272576	H	-9.186414	3.809126	-3.22897
C	1.310357	1.16314	2.672809	H	-10.916925	1.159617	-0.323292
C	3.406141	-0.15049	3.920241	C	-8.128174	-0.632472	2.935167
H	4.61835	1.480814	3.269728	H	-6.155942	-0.817662	3.788076
C	1.092924	-0.056477	3.310714	H	-9.971311	-0.288099	1.879259
H	0.462452	1.655713	2.217694	H	-11.138831	2.59144	-2.333198
C	2.135167	-0.715914	3.942794	H	-8.572264	-1.331606	3.636287
H	4.238955	-0.651654	4.402522	C	2.906279	3.071919	1.910572
H	0.096446	-0.484682	3.307006	C	0.109777	-2.08579	-2.136869
H	1.961484	-1.662072	4.444045	C	-0.878347	-2.282507	-1.177837
N	-1.3307	3.627956	-0.590747	C	-2.219011	-2.442079	-1.526071

C	-2.547447	-2.352635	-2.878406		C	2.562798	-3.473251	2.028674
C	-1.56664	-2.13622	-3.844747		C	5.751738	-1.807389	1.430986
C	-0.23663	-2.006361	-3.486107		C	4.585608	-3.196289	3.086044
H	-0.582315	-2.301771	-0.137127		C	1.27643	-3.907735	1.898283
H	-3.577437	-2.448531	-3.194665		C	3.337492	-3.761266	3.222027
H	-1.855237	-2.073738	-4.889378		C	6.844453	-1.732881	2.348022
H	0.538757	-1.856722	-4.227978		H	5.805473	-1.300618	0.481503
C	-4.673577	-2.553011	-0.975797		C	5.714354	-3.118522	3.982053
C	-5.428566	-1.403592	-0.765537		C	0.700731	-4.644305	2.977247
C	-5.215257	-3.556122	-1.789671		H	0.695967	-3.698256	1.016415
C	-6.680567	-1.25047	-1.358324		C	2.72268	-4.494101	4.301898
H	-5.053289	-0.618555	-0.124235		C	6.809134	-2.387433	3.596753
C	-6.460552	-3.40727	-2.382421		H	7.718803	-1.158152	2.065308
H	-4.649177	-4.465472	-1.961735		H	5.678865	-3.625729	4.939531
C	-7.199886	-2.247268	-2.170575		C	1.425397	-4.916132	4.155901
H	-7.254309	-0.350269	-1.174173		H	-0.319902	-4.994506	2.872447
H	-6.854155	-4.201286	-3.009764		H	3.289029	-4.701222	5.202881
H	-8.176289	-2.125333	-2.628811		H	7.666008	-2.308652	4.258367
C	-2.984008	-1.748092	0.760404		H	0.944692	-5.470279	4.955957
C	-3.115601	-2.117568	2.099368		N	4.662643	-1.174221	-3.036719
C	-2.59838	-0.430849	0.488226		C	4.458223	-0.319769	-4.14645
C	-2.874441	-1.210458	3.127548		C	6.054175	-1.377086	-2.885321
H	-3.396149	-3.128554	2.360366		C	3.284177	0.199108	-4.685967
C	-2.344084	0.473251	1.51045		C	5.713598	0.031842	-4.672181
H	-2.476972	-0.105142	-0.537774		C	6.758792	-2.17647	-1.988193
C	-2.484661	0.090623	2.840232		C	6.722408	-0.645775	-3.882441
H	-2.981472	-1.534887	4.158256		C	3.397526	1.074921	-5.75825
H	-2.039861	1.48259	1.264399		H	2.323462	-0.072039	-4.277752
H	-2.283684	0.797795	3.639138		C	5.805048	0.909409	-5.749496
C	-3.09882	-4.183943	0.101807		C	8.143771	-2.200702	-2.093106
C	-4.150909	-4.760161	0.825805		H	6.248776	-2.751859	-1.231381
C	-1.942457	-4.94288	-0.068301		C	8.111073	-0.682771	-3.969925
C	-4.039726	-6.026794	1.381041		C	4.640126	1.431333	-6.290117
H	-5.07177	-4.202385	0.957216		H	2.492684	1.487718	-6.194073
C	-1.826152	-6.215746	0.487127		H	6.775313	1.181868	-6.153028
H	-1.114544	-4.557001	-0.647477		C	8.819631	-1.45936	-3.066236
C	-2.869646	-6.762728	1.219584		H	8.708423	-2.814137	-1.398271
H	-4.873262	-6.440527	1.940252		H	8.626754	-0.1158	-4.738692
H	-0.909554	-6.777752	0.336429		H	4.691212	2.116418	-7.129913
H	-2.779507	-7.754648	1.651052		H	9.902727	-1.498239	-3.117937
C	1.519823	-2.009538	-1.70982		N	4.069954	-1.93289	-0.926346
C	3.682772	-1.585852	-2.151224		N	1.796781	-2.388783	-0.465189
C	3.082888	-2.335783	-0.119461		N	2.427865	-1.602705	-2.598533
N	3.409796	-2.739879	1.150312		C	-3.249544	-2.730454	-0.410883
C	4.655799	-2.531576	1.797638					

Supplementary Table 26. Atomic coordinates of SiCzCz:SiTrzCz2 (01, E = -4774.27150650 a.u.).

C	-3.214333	1.98715	1.756561		C	-0.925732	1.178988	2.285776
C	-4.273191	1.470158	1.010568		C	-2.644431	0.072683	3.286752
C	-5.174532	2.320805	0.354942		C	-0.11973	2.047464	1.593563
C	-4.96962	3.70197	0.454151		C	-0.370127	0.076792	3.074545
C	-3.912775	4.218816	1.195929		C	-3.863942	-0.277211	3.782911
C	-3.041079	3.367012	1.857954		C	-1.433911	-0.575939	3.691737
H	-4.37684	0.391039	0.941065		C	1.265523	1.820108	1.608511
H	-5.646252	4.382968	-0.051897		H	-0.531698	2.850544	0.997637
H	-3.774434	5.292941	1.269617		C	1.045219	-0.117105	3.091426
H	-2.228154	3.758305	2.459336		C	-3.910902	-1.355935	4.740642
C	-7.520718	3.070597	-1.4757		H	-4.771396	0.23378	3.488825
C	-8.464257	3.834769	-0.776155		C	-1.509778	-1.658967	4.624605
C	-7.243375	3.422202	-2.801766		C	1.831749	0.727542	2.350806
C	-9.100216	4.915788	-1.375057		H	1.912228	2.410632	0.979447
H	-8.712511	3.57574	0.249728		H	1.48568	-0.905393	3.687819
C	-7.876965	4.502324	-3.406481		C	-2.758757	-2.015324	5.135278
H	-6.527678	2.838459	-3.373725		H	-4.873153	-1.64977	5.144205
C	-8.805655	5.251522	-2.692675		H	-0.607285	-2.166339	4.947182
H	-9.829148	5.494296	-0.815656		H	-2.827357	-2.825706	5.854413
H	-7.648981	4.75659	-4.437147		N	3.219447	0.522083	2.254988
H	-9.303362	6.093807	-3.16368		C	4.211362	1.514578	2.278295
C	-7.8419	0.786815	0.511583		C	3.853967	-0.724922	2.144343
C	-9.108046	0.409101	0.041559		C	4.094467	2.897194	2.406027
C	-7.514944	0.463657	1.83239		C	5.476355	0.897545	2.18401
C	-9.99873	-0.28815	0.849091		C	3.299215	-1.990707	1.975573
H	-9.40843	0.670882	-0.970018		C	5.248398	-0.531784	2.127519
C	-8.403112	-0.228495	2.648582		C	5.257709	3.653929	2.361494
H	-6.549789	0.755881	2.231356		H	3.132594	3.377425	2.527346
C	-9.644442	-0.61281	2.155137		C	6.629854	1.676559	2.141332
H	-10.971768	-0.574326	0.461772		C	4.16542	-3.06668	1.854293
H	-8.123688	-0.467861	3.670272		H	2.230517	-2.135508	1.911857
H	-10.338848	-1.156474	2.78839		C	6.10143	-1.630104	2.032136
C	-5.993884	0.453378	-1.936515		C	6.513956	3.056908	2.214323
C	-4.76564	0.692581	-2.564861		H	5.180968	4.733756	2.429161
C	-6.699368	-0.698373	-2.307455		H	7.603321	1.206013	2.04496
C	-4.241501	-0.196343	-3.497848		C	5.553225	-2.896746	1.907022
H	-4.191296	1.576303	-2.299826		H	3.74893	-4.055564	1.696345
C	-6.18184	-1.591405	-3.238837		H	7.177266	-1.491695	2.023421
H	-7.655653	-0.918435	-1.842225		H	7.401085	3.678681	2.157879
C	-4.944425	-1.349389	-3.827444		H	6.204449	-3.758688	1.810842
H	-3.269584	-0.006171	-3.941654		Si	-6.631899	1.648957	-0.635027
H	-6.734412	-2.491525	-3.489898		C	0.929518	0.336685	-1.242123
H	-4.519259	-2.064741	-4.52321		C	0.388371	-0.881044	-0.822375
N	-2.299739	1.119471	2.40115		C	-0.992692	-1.060455	-0.717824

C	-1.818903	0.028593	-1.015186		C	4.860331	-2.823474	-1.515847
C	-1.291907	1.240345	-1.445114		C	7.546707	-0.457118	-0.739103
C	0.080189	1.392442	-1.57218		C	7.140351	-2.81111	-1.189088
H	1.06861	-1.686977	-0.569463		C	3.597217	-3.36028	-1.753382
H	-2.892621	-0.072632	-0.926441		C	5.993002	-3.653074	-1.459359
H	-1.953762	2.070901	-1.671826		C	8.883029	-0.77336	-0.526366
H	0.509214	2.327755	-1.912922		H	7.189577	0.549205	-0.60273
C	-3.328782	-2.772351	0.520948		C	8.481888	-3.109723	-0.971874
C	-3.432992	-3.396936	1.767955		C	3.496105	-4.736322	-1.914458
C	-4.494115	-2.219067	-0.032088		H	2.723977	-2.731216	-1.795668
C	-4.648596	-3.464436	2.441441		C	5.871213	-5.029528	-1.62063
H	-2.557873	-3.84546	2.224695		C	9.355706	-2.082501	-0.647412
C	-5.706087	-2.269442	0.644988		H	9.568043	0.023743	-0.253609
H	-4.467643	-1.766542	-1.017612		H	8.832944	-4.134484	-1.044674
C	-5.785214	-2.892318	1.886553		C	4.614436	-5.570013	-1.845822
H	-4.700057	-3.958465	3.405777		H	2.515069	-5.165399	-2.087389
H	-6.592516	-1.833965	0.199804		H	6.749258	-5.66605	-1.568759
H	-6.735327	-2.931553	2.41066		H	10.405405	-2.29719	-0.475246
C	-0.472722	-3.877751	0.372111		H	4.49872	-6.641866	-1.970677
C	0.01548	-5.033951	-0.245204		N	4.738616	3.133748	-1.32424
C	-0.026862	-3.58533	1.668994		C	4.073011	4.292745	-0.848503
C	0.914122	-5.872729	0.4072		C	6.112641	3.446797	-1.462846
H	-0.314541	-5.285272	-1.249267		C	2.727655	4.524638	-0.574555
C	0.857369	-4.428657	2.331007		C	5.031967	5.297829	-0.633104
H	-0.380873	-2.690065	2.172691		C	7.155996	2.699042	-1.998712
C	1.331726	-5.574341	1.698562		C	6.320017	4.765081	-1.027535
H	1.285622	-6.76264	-0.091633		C	2.373027	5.762128	-0.050309
H	1.187362	-4.186626	3.336887		H	1.986555	3.767385	-0.770634
H	2.02629	-6.23181	2.212748		C	4.656942	6.533169	-0.115211
C	-2.133952	-3.411579	-2.186358		C	8.421308	3.272547	-2.02161
C	-3.144818	-4.360815	-2.382238		H	6.991632	1.704787	-2.382502
C	-1.43773	-2.955713	-3.312561		C	7.593962	5.324126	-1.056473
C	-3.446489	-4.84163	-3.651479		C	3.321769	6.759563	0.184846
H	-3.716213	-4.719089	-1.530779		H	1.328001	5.954028	0.172211
C	-1.734266	-3.431578	-4.585355		H	5.402128	7.304613	0.053079
H	-0.656642	-2.209864	-3.195738		C	8.648038	4.565756	-1.543298
C	-2.740475	-4.377155	-4.756809		H	9.247386	2.697254	-2.427575
H	-4.235636	-5.576658	-3.779005		H	7.754489	6.341129	-0.712083
H	-1.18068	-3.063417	-5.443978		H	3.011407	7.715995	0.592611
H	-2.97407	-4.750592	-5.749506		H	9.649007	4.983793	-1.571192
C	2.394915	0.513173	-1.302383		N	5.04266	0.828882	-1.350643
C	4.199523	1.860094	-1.349788		N	3.148776	-0.581143	-1.27976
C	4.466044	-0.372578	-1.313206		N	2.870952	1.75984	-1.354414
N	5.28103	-1.482604	-1.3107		Si	-1.731181	-2.769171	-0.463104
C	6.68081	-1.487867	-1.091583					

Supplementary Table 27. Atomic coordinates of SiCzCz:SiTrzCz2 (02, E = -4774.26206424 a.u.).

C	3.255088	-2.968664	1.028284		C	0.957471	-2.27801	0.478237
C	4.24975	-2.268541	0.356114		C	2.06298	-1.056442	2.038705
C	5.458819	-2.869734	0.010575		C	0.587984	-3.191452	-0.467777
C	5.64128	-4.214095	0.357135		C	0.216596	-1.046458	0.700286
C	4.644041	-4.922694	1.019936		C	2.99063	-0.573162	2.909039
C	3.445123	-4.302326	1.364379		C	0.888852	-0.312281	1.663028
H	4.058114	-1.228274	0.115275		C	-0.577979	-2.921784	-1.22974
H	6.574761	-4.711571	0.109798		H	1.164983	-4.092083	-0.641215
H	4.80372	-5.96398	1.281889		C	-0.966611	-0.809515	-0.075389
H	2.661882	-4.833984	1.894929		C	2.759742	0.733683	3.474127
C	6.180109	-1.811973	-2.759438		H	3.878905	-1.139279	3.164056
C	7.09895	-1.470862	-3.76034		C	0.667359	0.973548	2.276988
C	4.85613	-2.062702	-3.142338		C	-1.344337	-1.744798	-1.005729
C	6.708398	-1.36723	-5.090914		H	-0.879522	-3.59875	-2.018908
H	8.13828	-1.289874	-3.498319		H	-1.544705	0.08742	0.081119
C	4.459711	-1.959082	-4.471078		C	1.614154	1.463376	3.160178
H	4.122011	-2.348258	-2.394134		H	3.487169	1.139805	4.168364
C	5.385735	-1.607775	-5.447347		H	-0.233174	1.530459	2.063385
H	7.437013	-1.100459	-5.850444		H	1.456492	2.433387	3.617887
H	3.427671	-2.156845	-4.74445		N	-2.50712	-1.539168	-1.787527
H	5.078229	-1.526736	-6.485457		C	-3.537612	-2.461595	-1.978524
C	8.419231	-2.539676	-0.79348		C	-2.848143	-0.358131	-2.449082
C	9.336256	-2.01653	0.125321		C	-3.676888	-3.757074	-1.482173
C	8.813524	-3.643294	-1.562066		C	-4.541137	-1.870454	-2.775357
C	10.59876	-2.578598	0.278852		C	-2.134762	0.833043	-2.553183
H	9.061856	-1.15333	0.725182		C	-4.097737	-0.526313	-3.078363
C	10.073621	-4.210411	-1.411137		C	-4.835393	-4.450842	-1.800743
H	8.130488	-4.058774	-2.298587		H	-2.908366	-4.20353	-0.863699
C	10.968038	-3.678091	-0.488163		C	-5.693093	-2.590069	-3.087305
H	11.296607	-2.155939	0.995283		C	-2.704211	1.863536	-3.284739
H	10.360213	-5.064768	-2.016843		H	-1.172191	0.951058	-2.075111
H	11.954041	-4.117348	-0.370605		C	-4.647084	0.522245	-3.814703
C	6.595392	-0.089662	-0.263278		C	-5.834314	-3.879217	-2.59756
C	6.669314	1.029593	-1.10076		H	-4.973633	-5.454485	-1.411661
C	6.411235	0.131816	1.10914		H	-6.477433	-2.135938	-3.684554
C	6.566421	2.319296	-0.590613		C	-3.949986	1.716758	-3.90814
H	6.788622	0.892709	-2.171511		H	-2.180188	2.811397	-3.358698
C	6.30204	1.41836	1.624724		H	-5.612236	0.404021	-4.296031
H	6.325839	-0.714849	1.784763		H	-6.733969	-4.444532	-2.815014
C	6.379864	2.51564	0.772863		H	-4.369961	2.546195	-4.468026
H	6.613386	3.171491	-1.261245		Si	6.692105	-1.835428	-0.954452
H	6.142137	1.564121	2.688588		C	-3.466375	2.744858	0.103697
H	6.283155	3.521343	1.170265		C	-2.116734	3.070482	0.221493
N	2.050968	-2.280362	1.341325		C	-1.578654	4.223997	-0.3517

C	-2.449583	5.060911	-1.057548	C	-1.870091	-0.332054	3.836843
C	-3.800976	4.744727	-1.187973	C	-2.630028	-3.32546	1.890248
C	-4.311469	3.586891	-0.617561	C	-1.141602	-2.503754	3.639845
H	-1.476096	2.381142	0.756064	C	-1.97098	0.958131	4.348649
H	-2.068772	5.969741	-1.514718	C	-0.947429	-1.256993	4.354236
H	-4.458319	5.405072	-1.744847	C	-1.956294	-4.538329	1.950517
H	-5.358588	3.327449	-0.724199	H	-3.443842	-3.171968	1.197876
C	0.72847	6.119357	-1.06012	C	-0.477759	-3.726893	3.681059
C	1.386059	6.05688	-2.293784	C	-1.114496	1.306989	5.383189
C	0.39599	7.383939	-0.555512	H	-2.678017	1.667372	3.9409
C	1.692849	7.212933	-3.003771	C	-0.087904	-0.881371	5.382364
H	1.669124	5.091191	-2.70244	C	-0.890022	-4.742698	2.831565
C	0.69828	8.542326	-1.261185	H	-2.269383	-5.343898	1.293427
H	-0.099071	7.465588	0.408915	H	0.355616	-3.87426	4.360793
C	1.34728	8.457286	-2.489093	C	-0.176013	0.404045	5.892844
H	2.205386	7.142027	-3.958422	H	-1.169536	2.308474	5.797163
H	0.432175	9.512277	-0.851973	H	0.637426	-1.586187	5.77592
H	1.587133	9.360921	-3.0414	H	-0.384634	-5.703015	2.850774
C	1.246819	3.102006	-0.728196	H	0.488346	0.714267	6.692549
C	2.336137	2.586881	-0.016888	N	-6.369597	-0.999669	0.08105
C	0.912283	2.485611	-1.942038	C	-7.233503	-0.511715	-0.936877
C	3.061532	1.499501	-0.492636	C	-6.83282	-2.286717	0.472309
H	2.619005	3.023887	0.933969	C	-7.199818	0.666634	-1.677039
C	1.629527	1.395392	-2.421888	C	-8.226803	-1.476722	-1.172042
H	0.071992	2.858189	-2.522396	C	-6.351983	-3.184569	1.422089
C	2.708243	0.900143	-1.695304	C	-7.973174	-2.593821	-0.287169
H	3.898799	1.123567	0.084337	C	-8.178547	0.852898	-2.646913
H	1.342548	0.929626	-3.359775	H	-6.423592	1.396047	-1.520511
H	3.274609	0.054332	-2.072912	C	-9.199199	-1.27235	-2.144534
C	0.590543	4.856324	1.715168	C	-7.016809	-4.39667	1.566234
C	1.844361	5.334473	2.12173	H	-5.490834	-2.953237	2.025636
C	-0.357587	4.589049	2.708909	C	-8.62756	-3.8099	-0.127122
C	2.148129	5.515813	3.465531	C	-9.171739	-0.099196	-2.884087
H	2.597119	5.574616	1.374961	H	-8.160233	1.765658	-3.234379
C	-0.063482	4.778191	4.055643	H	-9.96099	-2.024822	-2.32331
H	-1.343415	4.228628	2.432939	C	-8.139179	-4.717094	0.800624
C	1.192247	5.235931	4.437513	H	-6.646058	-5.107242	2.298181
H	3.128331	5.882066	3.755463	H	-9.504691	-4.041514	-0.723543
H	-0.818897	4.569578	4.806909	H	-9.920989	0.078968	-3.648627
H	1.424239	5.380909	5.488292	H	-8.632769	-5.673814	0.937304
C	-3.930456	1.460925	0.670143	N	-4.493409	-1.025414	1.443696
C	-5.256743	-0.358879	0.570641	N	-3.173399	0.923794	1.629566
C	-3.479875	-0.333822	1.947852	N	-5.005438	0.881613	0.141348
N	-2.64394	-0.973318	2.848674	Si	0.255004	4.571126	-0.112917
C	-2.207925	-2.31313	2.743437				

Supplementary Table 28. Atomic coordinates of SiCzCz:SiTrzCz2 (03, E = -4774.26047565 a.u.).

C	-5.348951	0.255162	1.82041		C	-3.048806	-0.547524	2.291026
C	-6.285985	-0.144705	0.872965		C	-4.748025	-2.001048	2.727318
C	-7.203894	0.757765	0.334337		C	-2.260865	0.508079	1.900802
C	-7.154927	2.087791	0.767782		C	-2.474785	-1.8253	2.721057
C	-6.218551	2.49225	1.713863		C	-5.972656	-2.575994	2.904635
C	-5.317997	1.580239	2.24927		C	-3.526568	-2.696432	2.985205
H	-6.264838	-1.174091	0.534525		C	-0.86916	0.326557	1.905487
H	-7.842885	2.818009	0.352214		H	-2.687291	1.435032	1.539241
H	-6.189532	3.526923	2.040695		C	-1.054838	-1.962007	2.7669
H	-4.593157	1.885077	2.995786		C	-6.006783	-3.950417	3.351231
C	-8.905613	1.581029	-2.064917		H	-6.894237	-2.03522	2.727073
C	-10.243409	1.854271	-2.369854		C	-3.585621	-4.058037	3.420192
C	-7.922788	2.40879	-2.62759		C	-0.283058	-0.918536	2.331606
C	-10.58895	2.909288	-3.208373		H	-0.228212	1.099367	1.508748
H	-11.02796	1.236264	-1.943145		H	-0.603028	-2.874661	3.1318
C	-8.261579	3.461972	-3.467914		C	-4.839073	-4.6521	3.59268
H	-6.873374	2.232101	-2.408938		H	-6.970284	-4.426769	3.4939
C	-9.598668	3.713975	-3.759362		H	-2.672755	-4.611712	3.608192
H	-11.633885	3.103956	-3.429899		H	-4.895983	-5.684896	3.922365
H	-7.482433	4.08694	-3.89367		N	1.112673	-1.057941	2.228385
H	-9.867153	4.537768	-4.413653		C	2.066432	-0.074297	2.529506
C	-9.932194	-0.558835	-0.088903		C	1.789725	-2.188757	1.745138
C	-10.808061	-1.422054	-0.761126		C	1.903044	1.194273	3.084101
C	-10.22443	-0.229131	1.239523		C	3.348288	-0.572115	2.2239
C	-11.939337	-1.92945	-0.133154		C	1.291775	-3.399651	1.26492
H	-10.597447	-1.711439	-1.787567		C	3.17299	-1.923939	1.739212
C	-11.353446	-0.736211	1.873839		C	3.039523	1.972046	3.264868
H	-9.557218	0.429812	1.788035		H	0.927838	1.570212	3.365488
C	-12.213212	-1.585962	1.187086		C	4.473401	0.226377	2.405104
H	-12.605065	-2.597218	-0.671436		C	2.203521	-4.341619	0.808705
H	-11.560579	-0.470065	2.905877		H	0.229145	-3.596445	1.228921
H	-13.094306	-1.984229	1.681008		C	4.071657	-2.889661	1.292788
C	-7.592275	-1.216799	-1.937667		C	4.311463	1.504975	2.915366
C	-6.938422	-0.961013	-3.149292		H	2.929222	2.969973	3.675526
C	-7.531552	-2.520576	-1.424608		H	5.457134	-0.146865	2.149477
C	-6.239471	-1.961388	-3.816112		C	3.582075	-4.100237	0.831209
H	-6.976629	0.033641	-3.583318		H	1.832712	-5.289142	0.429719
C	-6.834237	-3.524827	-2.08716		H	5.135233	-2.680715	1.291826
H	-8.040783	-2.759278	-0.494551		H	5.176174	2.14775	3.042205
C	-6.183502	-3.244689	-3.284286		H	4.268507	-4.858741	0.469953
H	-5.734044	-1.737079	-4.750132		Si	-8.429189	0.148371	-0.954439
H	-6.798805	-4.525697	-1.667764		C	3.756771	-1.043598	-1.561712
H	-5.63129	-4.024013	-3.799921		C	5.078414	-0.776629	-1.211286
N	-4.420964	-0.687287	2.315941		C	6.115189	-1.655223	-1.526833

C	5.782721	-2.846499	-2.180934
C	4.460679	-3.136396	-2.511299
C	3.446208	-2.237212	-2.211205
H	5.28053	0.142302	-0.672788
H	6.563263	-3.556718	-2.438019
H	4.221681	-4.069035	-3.012573
H	2.415824	-2.445215	-2.475531
C	9.11456	-2.388246	-1.575874
C	10.005923	-2.984021	-0.677662
C	9.206285	-2.75016	-2.927784
C	10.945921	-3.915427	-1.108132
H	9.969858	-2.716196	0.373743
C	10.140264	-3.681461	-3.363143
H	8.539937	-2.292528	-3.655167
C	11.012094	-4.267987	-2.450548
H	11.628264	-4.365011	-0.393083
H	10.192103	-3.9475	-4.414515
H	11.744611	-4.99508	-2.787686
C	7.901188	-0.827884	0.800897
C	8.042535	0.44863	1.357265
C	7.737929	-1.908005	1.68125
C	8.034736	0.639828	2.735917
H	8.1506	1.31138	0.708786
C	7.726115	-1.723666	3.058743
H	7.616388	-2.913717	1.285182
C	7.877526	-0.445717	3.589066
H	8.140525	1.641642	3.140931
H	7.591859	-2.575051	3.71864
H	7.860768	-0.297338	4.66415
C	8.217545	0.520195	-1.922541
C	9.44216	1.160856	-1.685178
C	7.319168	1.136661	-2.800009
C	9.749145	2.376819	-2.283386
H	10.168662	0.704438	-1.017119
C	7.620809	2.353418	-3.404653
H	6.365409	0.66459	-3.013287
C	8.834592	2.977899	-3.143831
H	10.702128	2.855863	-2.080235
H	6.903638	2.81539	-4.076056
H	9.069529	3.929646	-3.61038
C	2.724786	-0.039615	-1.236614
C	0.583518	0.632528	-1.116472
C	2.231996	2.017964	-0.463864
N	2.657058	3.204457	0.098291
C	1.869923	4.063578	0.904345
C	4.012331	3.609474	0.213002
C	0.501055	4.070669	1.159795
C	2.724267	4.988266	1.530565
C	5.16054	3.133364	-0.416023
C	4.075822	4.702548	1.091797
C	0.011538	5.018356	2.051116
H	-0.162172	3.368102	0.682191
C	2.213836	5.932026	2.416156
C	6.371807	3.74122	-0.111091
H	5.118545	2.315544	-1.115925
C	5.298837	5.295992	1.392205
C	0.851165	5.942995	2.675681
H	-1.053677	5.034202	2.259096
H	2.876592	6.643227	2.89952
C	6.449021	4.80373	0.79394
H	7.271024	3.381667	-0.601348
H	5.345763	6.134733	2.080026
H	0.436114	6.670906	3.365108
H	7.410928	5.254598	1.015984
N	-0.761067	0.389757	-1.308076
C	-1.38336	-0.884967	-1.26341
C	-1.776085	1.372942	-1.370121
C	-0.832189	-2.154665	-1.130358
C	-2.775619	-0.699385	-1.297021
C	-1.691567	2.760589	-1.454507
C	-3.022727	0.725095	-1.383144
C	-1.708883	-3.22824	-1.021405
H	0.236449	-2.298013	-1.097079
C	-3.636981	-1.787395	-1.198582
C	-2.879024	3.482215	-1.504573
H	-0.737452	3.264419	-1.469743
C	-4.1992	1.46353	-1.445215
C	-3.09484	-3.055178	-1.05447
H	-1.296688	-4.226438	-0.907943
H	-4.710639	-1.64374	-1.229017
C	-4.123602	2.847213	-1.490572
H	-2.82917	4.56534	-1.554719
H	-5.154945	0.954985	-1.44246
H	-3.752635	-3.913117	-0.964848
H	-5.03349	3.438646	-1.510447
N	0.923969	1.829899	-0.633549
N	3.166981	1.132039	-0.786574
N	1.439163	-0.346265	-1.41598
Si	7.847054	-1.107616	-1.057064

Supplementary Table 29. Atomic coordinates of SiCzCz:SiTrzCz2 (04, E = -4774.25162137 a.u.).

C	-0.764897	-2.129426	2.49976	C	-3.108944	-1.803688	1.711369
C	0.478116	-1.525151	2.333266	C	-2.111122	-0.026933	2.737158
C	1.65923	-2.184779	2.679015	C	-3.441546	-3.01334	1.150894
C	1.570263	-3.518083	3.095671	C	-4.057047	-0.690871	1.747627
C	0.336123	-4.150446	3.193071	C	-1.261597	0.808434	3.404199
C	-0.835719	-3.453152	2.932473	C	-3.443037	0.37266	2.389892
H	0.51051	-0.506908	1.968297	C	-4.717093	-3.146686	0.573317
H	2.466868	-4.058199	3.385405	H	-2.732838	-3.827533	1.088829
H	0.279161	-5.183341	3.521684	C	-5.349643	-0.856811	1.155754
H	-1.799664	-3.918774	3.09665	C	-1.740531	2.133771	3.736359
C	4.389682	-1.396874	1.327004	H	-0.261067	0.522434	3.697465
C	5.102951	-0.318603	0.790834	C	-3.899778	1.688553	2.740985
C	4.689064	-2.683475	0.856864	C	-5.645123	-2.056378	0.569956
C	6.097805	-0.520834	-0.160938	H	-4.98771	-4.059366	0.05836
H	4.888013	0.690704	1.129124	H	-6.075012	-0.052662	1.170494
C	5.685321	-2.892122	-0.087995	C	-3.024506	2.539611	3.410535
H	4.149927	-3.540922	1.253516	H	-1.059825	2.81281	4.234871
C	6.400961	-1.807914	-0.587519	H	-4.90339	2.003921	2.480631
H	6.63775	0.325981	-0.572658	H	-3.354559	3.538654	3.676625
H	5.901407	-3.895179	-0.441428	N	-6.866508	-2.224253	-0.118076
H	7.180174	-1.965021	-1.325221	C	-7.747533	-3.295056	0.02086
C	4.18504	-1.823703	4.311999	C	-7.354186	-1.379285	-1.116248
C	5.581902	-1.913968	4.309932	C	-7.700261	-4.36744	0.909296
C	3.508586	-2.171161	5.489676	C	-8.811628	-3.136833	-0.890706
C	6.278479	-2.330518	5.439343	C	-6.798772	-0.21283	-1.638205
H	6.134521	-1.659808	3.410303	C	-8.557742	-1.911796	-1.620935
C	4.199245	-2.586233	6.622247	C	-8.727825	-5.29736	0.849778
H	2.423307	-2.125636	5.521154	H	-6.89224	-4.46616	1.624767
C	5.587856	-2.666724	6.597991	C	-9.833721	-4.083286	-0.928744
H	7.362088	-2.39508	5.413503	C	-7.477824	0.421155	-2.667978
H	3.653763	-2.851103	7.523026	H	-5.857807	0.169155	-1.26268
H	6.12979	-2.993804	7.480282	C	-9.22612	-1.251644	-2.651096
C	2.767081	0.595679	3.061877	C	-9.784063	-5.163199	-0.060601
C	2.320912	1.398053	2.002029	H	-8.713847	-6.143819	1.529188
C	2.73296	1.140325	4.351637	H	-10.658729	-3.97281	-1.626008
C	1.856735	2.68918	2.221304	C	-8.684621	-0.085103	-3.168503
H	2.322597	1.010011	0.98809	H	-7.061598	1.328389	-3.096015
C	2.260328	2.429547	4.577028	H	-10.154545	-1.651924	-3.04704
H	3.08009	0.549454	5.193778	H	-10.572532	-5.908344	-0.079725
C	1.820705	3.206945	3.511741	H	-9.194022	0.437984	-3.971036
H	1.52646	3.294163	1.386533	Si	3.256667	-1.196237	2.802875
H	2.240637	2.828468	5.58702	C	2.347816	2.279953	-1.575487
H	1.452602	4.213158	3.678463	C	1.178167	2.975048	-1.263264
N	-1.935875	-1.364703	2.296332	C	1.186528	4.356087	-1.056277

C	2.41097	5.027503	-1.182265	C	-1.150827	-1.570686	-1.719257
C	3.584654	4.343339	-1.476281	C	1.182302	-4.054182	-0.425099
C	3.556602	2.970444	-1.670818	C	-1.094366	-3.828831	-1.272688
H	0.256064	2.412282	-1.180224	C	-1.667285	-0.345516	-2.122228
H	2.449205	6.10643	-1.058698	C	-1.919774	-2.746478	-1.775997
H	4.523367	4.882493	-1.554282	C	0.91535	-5.3946	-0.180733
H	4.463724	2.419172	-1.887343	H	2.143214	-3.620419	-0.183725
C	-0.263033	5.731121	1.289413	C	-1.344344	-5.175628	-1.018841
C	-1.313358	5.449944	2.168994	C	-2.968442	-0.328322	-2.612029
C	0.850681	6.414419	1.798858	H	-1.080836	0.559297	-2.053914
C	-1.257959	5.839522	3.504434	C	-3.219329	-2.705661	-2.270328
H	-2.186824	4.91674	1.809426	C	-0.332437	-5.95448	-0.47539
C	0.909983	6.811162	3.128709	H	1.690529	-6.013977	0.259215
H	1.688884	6.645911	1.147523	H	-2.317555	-5.604598	-1.237932
C	-0.149062	6.524011	3.98623	C	-3.737933	-1.491767	-2.694708
H	-2.085881	5.611621	4.169297	H	-3.391233	0.618068	-2.93098
H	1.782077	7.342168	3.497911	H	-3.814585	-3.610925	-2.327188
H	-0.107454	6.833112	5.026266	H	-0.511197	-7.005058	-0.270586
C	-1.90708	4.316176	-0.892357	H	-4.749443	-1.440971	-3.081491
C	-2.855658	4.803874	-1.800356	N	4.393063	-1.700898	-3.096411
C	-2.172124	3.092943	-0.257739	C	5.537649	-1.060717	-3.639591
C	-4.029105	4.104985	-2.061363	C	4.584316	-3.105272	-3.196837
H	-2.67799	5.747774	-2.306208	C	5.834085	0.28874	-3.817431
C	-3.337436	2.383858	-0.526641	C	6.447073	-2.051824	-4.046801
H	-1.473286	2.684787	0.468795	C	3.761315	-4.167232	-2.830847
C	-4.27106	2.894842	-1.422772	C	5.849735	-3.339609	-3.759294
H	-4.754892	4.507806	-2.761355	C	7.061375	0.617121	-4.381582
H	-3.51104	1.432709	-0.038482	H	5.130475	1.05182	-3.530173
H	-5.191426	2.35181	-1.616723	C	7.671847	-1.703833	-4.607201
C	-0.38255	6.912997	-1.508568	C	4.245627	-5.456576	-3.013873
C	0.014473	6.949054	-2.85191	H	2.781009	-3.999791	-2.419483
C	-0.841324	8.101143	-0.927773	C	6.317954	-4.638003	-3.935056
C	-0.048994	8.125252	-3.589691	C	7.979066	-0.361405	-4.76865
H	0.385991	6.045417	-3.328068	H	7.304836	1.665162	-4.524852
C	-0.906248	9.281283	-1.660494	H	8.370794	-2.474925	-4.916007
H	-1.146854	8.105728	0.114558	C	5.511004	-5.699111	-3.553292
C	-0.510492	9.29438	-2.99333	H	3.612785	-6.291023	-2.72818
H	0.26453	8.131085	-4.62921	H	7.299519	-4.812449	-4.364751
H	-1.263626	10.192184	-1.189883	H	8.92944	-0.070135	-5.203695
H	-0.558745	10.215223	-3.56668	H	5.859054	-6.718963	-3.68083
C	2.314584	0.817034	-1.791425	N	2.319401	-1.83102	-2.066738
C	3.338909	-1.074912	-2.470709	N	1.218237	0.155074	-1.405378
C	1.278984	-1.164312	-1.576987	N	3.3856	0.253581	-2.343011
N	0.149156	-1.906544	-1.262249	Si	-0.356043	5.305701	-0.541577
C	0.167507	-3.287083	-0.983613				

Supplementary Table 30. Atomic coordinates of SiCzCz:SiTrzCz2 (05, E = -4774.24903784 a.u.).

C	4.651698	-1.274952	-0.275369	C	2.514711	-0.125763	-0.826237
C	5.995271	-0.967034	-0.459954	C	3.859215	0.886369	0.708425
C	6.967814	-1.968777	-0.508494	C	1.971879	-0.908589	-1.814516
C	6.548997	-3.300304	-0.419866	C	1.857576	1.099262	-0.365653
C	5.199632	-3.613704	-0.279382	C	4.858004	1.187018	1.588718
C	4.248029	-2.607127	-0.190133	C	2.704281	1.714903	0.548156
H	6.275468	0.076019	-0.559804	C	0.718694	-0.536369	-2.332035
H	7.281286	-4.100773	-0.459515	H	2.492457	-1.775008	-2.2035
H	4.888383	-4.651896	-0.214625	C	0.544819	1.403788	-0.845348
H	3.200074	-2.834991	-0.034889	C	4.740771	2.417096	2.336588
C	9.838805	-2.904729	-1.053163	H	5.715388	0.541327	1.735679
C	11.002628	-3.247258	-0.355938	C	2.616523	2.931124	1.293862
C	9.503306	-3.663868	-2.183393	C	-0.008117	0.583002	-1.790833
C	11.806741	-4.302509	-0.773369	H	0.301615	-1.066499	-3.177068
H	11.282927	-2.683507	0.529036	H	-0.012366	2.224483	-0.414669
C	10.303393	-4.71906	-2.605681	C	3.649476	3.253092	2.174168
H	8.597501	-3.433588	-2.738966	H	5.531007	2.677679	3.031846
C	11.458811	-5.039047	-1.899812	H	1.764328	3.583011	1.161489
H	12.704976	-4.551671	-0.216602	H	3.588239	4.170788	2.750503
H	10.024453	-5.293811	-3.483617	N	-1.308514	0.812546	-2.280742
H	12.084839	-5.863898	-2.226295	C	-2.266455	-0.177247	-2.539791
C	9.204866	-0.908152	1.222973	C	-1.884813	2.060387	-2.556217
C	10.325357	-0.10324	1.468462	C	-2.20557	-1.557128	-2.341559
C	8.419302	-1.288607	2.317952	C	-3.444129	0.435902	-3.010497
C	10.654154	0.299606	2.757715	C	-1.336739	3.339732	-2.473302
H	10.943793	0.226914	0.637766	C	-3.205334	1.863603	-3.009632
C	8.740099	-0.884178	3.609492	C	-3.323682	-2.311428	-2.663397
H	7.540125	-1.906387	2.15904	H	-1.321273	-2.027737	-1.934208
C	9.859704	-0.090145	3.831092	C	-4.552146	-0.342019	-3.340369
H	11.526507	0.924199	2.924505	C	-2.14658	4.415043	-2.811326
H	8.11452	-1.188185	4.443316	H	-0.314779	3.499385	-2.156141
H	10.111203	0.228182	4.838243	C	-4.001385	2.959131	-3.336373
C	9.019779	0.005434	-1.670554	C	-4.483545	-1.715116	-3.171553
C	9.513325	-0.154654	-2.970344	H	-3.298966	-3.383359	-2.501181
C	8.663361	1.29768	-1.259283	H	-5.461971	0.125677	-3.701536
C	9.635274	0.929807	-3.832181	C	-3.471215	4.234896	-3.225225
H	9.814587	-1.139891	-3.314048	H	-1.742669	5.418926	-2.733965
C	8.778637	2.38443	-2.117806	H	-5.024454	2.811297	-3.668703
H	8.29909	1.462822	-0.248558	H	-5.336264	-2.335604	-3.425359
C	9.264223	2.200786	-3.408171	H	-4.084769	5.100964	-3.449401
H	10.022553	0.782483	-4.835824	Si	8.775756	-1.456823	-0.520527
H	8.492398	3.375201	-1.778517	C	-5.227775	0.638972	-0.075252
H	9.357431	3.048234	-4.080491	C	-5.37592	-0.716133	0.209523
N	3.710019	-0.230967	-0.147255	C	-6.516959	-1.419909	-0.188175

C	-7.501924	-0.725951	-0.899625		C	-0.520511	-0.502278	1.739879
C	-7.351331	0.622795	-1.210622		C	-0.153931	2.613401	3.618016
C	-6.220353	1.306717	-0.797319		C	0.944006	0.474991	3.224358
H	-4.581589	-1.211193	0.757		C	-1.038934	-1.465859	0.88339
H	-8.398469	-1.246061	-1.223534		C	0.685757	-0.711077	2.432413
H	-8.121974	1.139668	-1.774298		C	0.846715	2.906359	4.536504
H	-6.07932	2.352627	-1.039756		H	-0.955322	3.312051	3.426891
C	-7.369043	-3.242667	2.099674		C	1.941464	0.791049	4.1404
C	-7.212177	-4.38612	2.894413		C	-0.313514	-2.639916	0.723543
C	-8.025668	-2.14027	2.659179		H	-1.970003	-1.30812	0.360911
C	-7.700962	-4.430903	4.195014		C	1.391817	-1.898453	2.266454
H	-6.689936	-5.251777	2.495644		C	1.888431	2.012042	4.794491
C	-8.514667	-2.178312	3.960255		H	0.813104	3.855989	5.061132
H	-8.150408	-1.235084	2.071729		H	2.747536	0.091283	4.33573
C	-8.354027	-3.325213	4.72971		C	0.884204	-2.863208	1.408064
H	-7.56719	-5.326622	4.794035		H	-0.702167	-3.402732	0.057917
H	-9.017706	-1.310306	4.375566		H	2.328255	-2.057855	2.791928
H	-8.732765	-3.356095	5.74688		H	2.661425	2.276702	5.50821
C	-5.150812	-4.167735	0.234282		H	1.416136	-3.799835	1.271411
C	-4.890717	-5.072494	-0.801362		N	-2.925811	4.714652	0.487304
C	-4.166812	-3.996457	1.217149		C	-3.961721	5.503279	-0.075469
C	-3.689975	-5.772484	-0.861683		C	-1.743981	5.498934	0.516346
H	-5.639978	-5.240013	-1.569426		C	-5.311804	5.224334	-0.270403
C	-2.966113	-4.693855	1.164547		C	-3.430867	6.756686	-0.419271
H	-4.343005	-3.3197	2.049028		C	-0.463015	5.201171	0.973893
C	-2.724634	-5.581986	0.121156		C	-2.03003	6.753298	-0.049826
H	-3.511814	-6.473331	-1.671935		C	-6.101078	6.20853	-0.851576
H	-2.217427	-4.538846	1.934098		H	-5.727102	4.271349	0.013055
H	-1.788796	-6.131753	0.080124		C	-4.235321	7.729493	-1.005085
C	-8.030898	-4.008852	-0.803492		C	0.519492	6.175327	0.837761
C	-9.043979	-4.825939	-0.289402		H	-0.242819	4.249062	1.427216
C	-7.969598	-3.839943	-2.193894		C	-1.033958	7.716357	-0.175013
C	-9.956672	-5.455824	-1.129133		C	-5.574013	7.446703	-1.228205
H	-9.123345	-4.969582	0.784146		H	-7.154578	6.004137	-1.014615
C	-8.877752	-4.46689	-3.038625		H	-3.818311	8.694285	-1.27678
H	-7.206094	-3.197202	-2.623537		C	0.246302	7.420457	0.268462
C	-9.874079	-5.278419	-2.505319		H	1.52398	5.955006	1.185613
H	-10.735596	-6.08388	-0.70749		H	-1.259549	8.682415	-0.615899
H	-8.810503	-4.320319	-4.112413		H	-6.217386	8.19151	-1.685116
H	-10.58652	-5.768485	-3.161957		H	1.036601	8.158263	0.177413
C	-4.042506	1.383173	0.397731		N	-1.956083	2.769937	1.309724
C	-2.98661	3.351908	0.697102		N	-3.039549	0.696756	0.953008
C	-2.0361	1.442622	1.411385		N	-4.065734	2.707565	0.260742
N	-0.986172	0.798685	2.039281		Si	-6.761881	-3.209526	0.324827
C	-0.087951	1.390515	2.95656					

Supplementary Table 31. Atomic coordinates of SiCzCz:SiTrzCz2 (06, E = -4774.25120284 a.u.).

C	-2.180977	4.37503	-0.446057	C	-3.570972	2.736509	-1.712565
C	-1.307077	3.750299	0.440796	C	-1.962371	3.809025	-2.872977
C	-0.891549	4.401515	1.60771	C	-4.39987	2.252176	-0.70369
C	-1.379498	5.692653	1.848117	C	-3.606391	2.216464	-3.024649
C	-2.255356	6.313959	0.96611	C	-0.884696	4.607658	-3.247134
C	-2.659618	5.654903	-0.187416	C	-2.577978	2.912205	-3.772965
H	-0.94306	2.756162	0.194087	C	-5.233995	1.194088	-1.013467
H	-1.057674	6.228513	2.735579	H	-4.358561	2.656009	0.301667
H	-2.618206	7.315509	1.173712	C	-4.466134	1.158772	-3.319364
H	-3.338774	6.119077	-0.894886	C	-0.4285	4.493473	-4.551626
C	-0.624336	2.565342	4.085992	H	-0.42161	5.287158	-2.540014
C	-0.022168	2.274199	5.318796	C	-2.096836	2.816267	-5.079416
C	-1.918468	2.082436	3.860604	C	-5.256711	0.640373	-2.304368
C	-0.680339	1.522356	6.284711	H	-5.852138	0.747857	-0.245055
H	0.971923	2.656243	5.535102	H	-4.500977	0.714926	-4.308817
C	-2.585224	1.33837	4.829072	C	-1.025079	3.607648	-5.461127
H	-2.417737	2.297724	2.919351	H	0.407309	5.10576	-4.876454
C	-1.965358	1.052107	6.039377	H	-2.551346	2.125874	-5.783319
H	-0.192049	1.307473	7.230439	H	-0.638621	3.540515	-6.472473
H	-3.591004	0.976864	4.642248	N	-6.036158	-0.525638	-2.535845
H	-2.484465	0.464476	6.789887	C	-7.397303	-0.649076	-2.283212
C	1.194313	4.964596	3.76013	C	-5.494555	-1.80582	-2.650111
C	2.45727	5.416265	3.360392	C	-8.339047	0.349351	-2.049771
C	0.592134	5.592668	4.859295	C	-7.748597	-2.015995	-2.287352
C	3.091554	6.4609	4.02404	C	-4.165415	-2.18217	-2.824884
H	2.957563	4.939861	2.522532	C	-6.524143	-2.758491	-2.51069
C	1.219876	6.639115	5.524959	C	-9.648964	-0.043705	-1.817785
H	-0.378234	5.250926	5.209855	H	-8.05312	1.395389	-2.046558
C	2.472392	7.075986	5.106176	C	-9.073553	-2.384709	-2.057734
H	4.072888	6.79206	3.698121	C	-3.878923	-3.538759	-2.839481
H	0.734489	7.110228	6.37439	H	-3.378823	-1.4435	-2.920815
H	2.966934	7.890639	5.62653	C	-6.210156	-4.118017	-2.534833
C	1.572887	2.589285	1.891912	C	-10.017352	-1.395857	-1.822066
C	2.140693	1.443944	2.462589	H	-10.403988	0.713635	-1.631155
C	2.111015	3.057088	0.685135	H	-9.360697	-3.432065	-2.059264
C	3.230547	0.810925	1.876481	C	-4.886863	-4.501551	-2.69172
H	1.736311	1.049294	3.389728	H	-2.849834	-3.861101	-2.949114
C	3.206525	2.434561	0.097347	H	-6.99041	-4.863809	-2.414413
H	1.677395	3.929305	0.202617	H	-11.051332	-1.669455	-1.639146
C	3.777861	1.321207	0.704463	H	-4.620907	-5.552958	-2.687781
H	3.652504	-0.078133	2.334799	Si	0.302047	3.599735	2.823835
H	3.617202	2.817435	-0.831713	C	0.964645	-0.81585	-1.063503
H	4.64673	0.846835	0.259488	C	0.188439	-1.565697	-0.181546
N	-2.584134	3.705715	-1.635129	C	-1.189295	-1.363686	-0.066628

C	-1.77209	-0.360615	-0.84831
C	-1.005901	0.387624	-1.738399
C	0.354779	0.157097	-1.854592
H	0.682876	-2.322431	0.416771
H	-2.835292	-0.16231	-0.765494
H	-1.469956	1.143714	-2.355233
H	0.951621	0.724797	-2.556796
C	-3.911729	-1.730647	1.339565
C	-4.083202	-0.652686	2.217867
C	-5.046282	-2.240193	0.696788
C	-5.339995	-0.11102	2.458968
H	-3.220317	-0.243944	2.733987
C	-6.310329	-1.712544	0.945807
H	-4.950366	-3.063875	-0.002609
C	-6.459717	-0.650572	1.830969
H	-5.451418	0.725471	3.14373
H	-7.176192	-2.124105	0.438531
H	-7.446267	-0.23921	2.023132
C	-1.295237	-2.464289	2.749581
C	-1.481447	-3.521495	3.649456
C	-0.473857	-1.40193	3.150209
C	-0.890305	-3.511513	4.907912
H	-2.09472	-4.370972	3.361879
C	0.126837	-1.390724	4.40431
H	-0.292463	-0.570246	2.475813
C	-0.086537	-2.4414	5.28878
H	-1.053788	-4.340795	5.589837
H	0.75006	-0.553659	4.697971
H	0.379237	-2.430261	6.269638
C	-2.317199	-4.189114	0.416665
C	-3.292798	-5.076999	0.889202
C	-1.430334	-4.65738	-0.559714
C	-3.383777	-6.376516	0.403982
H	-4.008771	-4.74517	1.636221
C	-1.512911	-5.956852	-1.047296
H	-0.66806	-3.994398	-0.957495
C	-2.492909	-6.81921	-0.567778
H	-4.153711	-7.042547	0.78155
H	-0.812918	-6.295508	-1.805239
H	-2.561515	-7.833464	-0.949497
C	2.420938	-1.032402	-1.144256
C	4.397411	-0.630014	-2.13782
C	4.311053	-1.92781	-0.304319
N	4.935604	-2.640897	0.685898
C	6.327951	-2.923767	0.769501

C	4.282751	-3.305383	1.768065
C	7.39409	-2.421707	0.082721
C	6.508799	-3.877437	1.850353
C	2.999975	-3.246606	2.228903
C	5.288771	-4.102812	2.447637
C	8.693806	-2.909491	0.411573
H	7.271341	-1.675512	-0.683866
C	7.834771	-4.359106	2.150353
C	2.65342	-4.038944	3.36482
H	2.258719	-2.621919	1.761895
C	4.904498	-4.904239	3.583926
C	8.890655	-3.870027	1.427248
H	9.544734	-2.526452	-0.140084
H	7.976747	-5.087596	2.940565
C	3.60381	-4.85542	4.012885
H	1.631631	-4.006148	3.72203
H	5.644336	-5.521385	4.081095
H	9.897319	-4.216771	1.638484
H	3.29394	-5.448197	4.867845
N	5.121873	0.003702	-3.133593
C	4.75396	1.212564	-3.77053
C	6.398006	-0.373045	-3.603466
C	3.609974	1.996725	-3.638259
C	5.80768	1.60747	-4.613548
C	7.14453	-1.520329	-3.351219
C	6.844368	0.598735	-4.515278
C	3.539558	3.173001	-4.373537
H	2.802006	1.692874	-2.992905
C	5.716601	2.789554	-5.34354
C	8.369227	-1.649618	-3.993599
H	6.784759	-2.285756	-2.680476
C	8.075456	0.450987	-5.148464
C	4.577015	3.570027	-5.221774
H	2.645833	3.783688	-4.289381
H	6.529672	3.093696	-5.995476
C	8.840119	-0.67311	-4.876267
H	8.967733	-2.535251	-3.804921
H	8.42377	1.201802	-5.850955
H	4.489306	4.492003	-5.787272
H	9.802711	-0.803167	-5.359862
N	5.069133	-1.371266	-1.258745
N	2.983274	-1.810214	-0.225978
N	3.084473	-0.416524	-2.125318
Si	-2.190844	-2.442076	1.093702

Supplementary Table 32. Atomic coordinates of SiCzCz:SiTrzCz2 (07, E = -4774.25471717 a.u.).

C	-1.753599	-1.342227	0.70313		C	-4.191192	-1.595647	0.288476
C	-0.722044	-1.802743	1.519129		C	-2.940278	-3.470947	0.118947
C	0.462338	-1.07114	1.667114		C	-4.67057	-0.309996	0.542973
C	0.559569	0.169157	1.028464		C	-5.038954	-2.603978	-0.213794
C	-0.487519	0.645459	0.255016		C	-1.915384	-4.415561	0.116043
C	-1.629464	-0.111232	0.065914		C	-4.239288	-3.805052	-0.318591
H	-0.842261	-2.747763	2.036064		C	-5.995267	-0.042244	0.24905
H	1.454429	0.773014	1.128514		H	-4.036185	0.46191	0.959428
H	-0.422675	1.603915	-0.238746		C	-6.365607	-2.311018	-0.521683
H	-2.402863	0.241215	-0.603362		C	-2.218769	-5.697298	-0.317776
C	1.73587	-0.982898	4.450383		H	-0.91027	-4.170981	0.429139
C	2.869407	-0.63476	5.192305		C	-4.51853	-5.098371	-0.757545
C	0.480679	-0.809112	5.047413		C	-6.834774	-1.025006	-0.296988
C	2.756112	-0.12904	6.484133		H	-6.404112	0.944262	0.439336
H	3.855474	-0.751103	4.752162		H	-7.026927	-3.061068	-0.943012
C	0.359666	-0.308763	6.338145		C	-3.50471	-6.043119	-0.753185
H	-0.418017	-1.059654	4.489628		H	-1.434116	-6.447445	-0.312927
C	1.50032	0.034855	7.058993		H	-5.516315	-5.358374	-1.0979
H	3.648781	0.138052	7.041743		H	-3.70688	-7.056385	-1.08423
H	-0.623248	-0.182033	6.782093		N	-8.170401	-0.681117	-0.627742
H	1.409305	0.429931	8.066262		C	-8.538663	0.294176	-1.54639
C	3.53627	-1.296302	1.993452		C	-9.322833	-1.202512	-0.049417
C	4.404325	-2.296991	1.541212		C	-7.730802	1.066154	-2.378792
C	3.97177	0.035578	1.905177		C	-9.944662	0.405602	-1.563129
C	5.660188	-1.986486	1.028499		C	-9.448168	-2.182681	0.931804
H	4.097406	-3.336714	1.594954		C	-10.447985	-0.556796	-0.604325
C	5.221525	0.351692	1.385015		C	-8.356216	1.974291	-3.220044
H	3.33782	0.840587	2.267753		H	-6.652874	0.946986	-2.374349
C	6.073031	-0.662167	0.955323		C	-10.548475	1.32606	-2.418874
H	6.318803	-2.778382	0.68427		C	-10.729383	-2.517857	1.343249
H	5.538738	1.388157	1.319509		H	-8.57375	-2.661728	1.357997
H	7.056102	-0.418209	0.567696		C	-11.725243	-0.91368	-0.173679
C	1.661475	-3.587449	2.871425		C	-9.751183	2.109287	-3.239707
C	1.617526	-4.224117	4.11644		H	-7.751575	2.589528	-3.879264
C	1.526876	-4.381569	1.723341		H	-11.629543	1.425572	-2.443317
C	1.441999	-5.600906	4.21565		C	-11.859595	-1.894341	0.796911
H	1.714887	-3.635043	5.023318		H	-10.857606	-3.279192	2.106383
C	1.351767	-5.756963	1.816841		H	-12.601537	-0.427343	-0.591772
H	1.55964	-3.923116	0.739383		H	-10.209471	2.830337	-3.90854
C	1.306372	-6.36899	3.065547		H	-12.847835	-2.182178	1.140291
H	1.407356	-6.072893	5.192906		Si	1.864597	-1.727262	2.73139
H	1.250372	-6.346024	0.911608		C	2.866887	3.119865	-1.903482
H	1.164583	-7.442931	3.140917		C	1.707738	3.356441	-1.173363
N	-2.917085	-2.122217	0.487298		C	1.249579	4.649265	-0.910824

C	1.977966	5.715997	-1.446145		C	0.539733	-1.335417	-2.543839
C	3.13595	5.492042	-2.190125		C	3.283827	-3.50838	-1.521956
C	3.59589	4.199393	-2.403565		C	0.915521	-3.5722	-2.17462
H	1.194049	2.495397	-0.767425		C	-0.172148	-0.244451	-2.942176
H	1.651564	6.735588	-1.261884		C	-0.057334	-2.658641	-2.518047
H	3.691129	6.334175	-2.59117		C	3.223917	-4.926284	-1.374152
H	4.5098	4.014024	-2.957672		H	4.194769	-2.975456	-1.306138
C	-0.432357	6.658535	0.700857		C	0.893581	-5.007988	-2.037148
C	0.176602	7.253907	1.811853		C	-1.543197	-0.434963	-3.297621
C	-1.212025	7.467632	-0.135951		H	0.270988	0.738753	-2.964118
C	0.019974	8.610264	2.074718		C	-1.454855	-2.811294	-2.848878
H	0.775597	6.647233	2.485143		C	2.043588	-5.648805	-1.652926
C	-1.370414	8.824371	0.121105		H	4.113051	-5.452043	-1.04568
H	-1.711418	7.029296	-0.996132		H	-0.02059	-5.551095	-2.243252
C	-0.752764	9.397521	1.228095		C	-2.161918	-1.700535	-3.228244
H	0.498002	9.052445	2.943566		H	-2.120201	0.424889	-3.615395
H	-1.979812	9.434144	-0.538991		H	-1.922002	-3.786105	-2.780413
H	-0.877905	10.456462	1.43302		H	2.051795	-6.730133	-1.554716
C	0.355036	3.899145	1.835309		H	-3.215325	-1.790332	-3.473548
C	-0.543101	3.178877	2.631024		N	6.330426	-0.073881	-2.221787
C	1.705889	3.898253	2.212493		C	7.365235	0.804685	-1.810725
C	-0.114464	2.492455	3.760698		C	6.900156	-1.349408	-2.450349
H	-1.591013	3.129102	2.352554		C	7.329237	2.148902	-1.444416
C	2.138753	3.217574	3.345236		C	8.567538	0.076729	-1.75834
H	2.435208	4.427946	1.605694		C	6.326157	-2.524093	-2.929912
C	1.227229	2.511429	4.123521		C	8.274272	-1.282256	-2.167056
H	-0.823719	1.925026	4.35413		C	8.517685	2.740133	-1.032864
H	3.189779	3.232882	3.619168		H	6.408364	2.708178	-1.485502
H	1.560927	1.969175	5.000802		C	9.747527	0.68949	-1.347083
C	-1.770109	4.138481	-0.391759		C	7.147122	-3.635397	-3.073847
C	-2.957418	4.257011	0.345985		H	5.278289	-2.573232	-3.178125
C	-1.829793	3.460298	-1.614628		C	9.08117	-2.406655	-2.317107
C	-4.147241	3.6947	-0.10112		C	9.717826	2.02695	-0.984144
H	-2.957225	4.806235	1.284181		H	8.506494	3.786997	-0.746245
C	-3.021987	2.90685	-2.074066		H	10.672954	0.123354	-1.307881
H	-0.932881	3.362194	-2.219958		C	8.509008	-3.587788	-2.763463
C	-4.181356	3.013882	-1.314231		H	6.712236	-4.559946	-3.440261
H	-5.05209	3.797507	0.490378		H	10.141989	-2.353313	-2.092926
H	-3.048326	2.400018	-3.033351		H	10.627701	2.521697	-0.660619
H	-5.11264	2.583347	-1.668581		H	9.121552	-4.475276	-2.883968
C	3.320692	1.722563	-2.062918		N	4.12915	-0.811974	-2.174633
C	4.979009	0.210753	-2.185582		N	2.382766	0.778925	-2.057696
C	2.838011	-0.475199	-2.131736		N	4.630344	1.499311	-2.158534
N	1.906457	-1.483607	-2.174752		Si	-0.173394	4.847121	0.300525
C	2.158722	-2.860349	-1.940833					

Supplementary Table 33. Atomic coordinates of SiCzCz:SiTrzCz2 (08, E = -4774.24448179 a.u.).

C	-2.540071	-1.226615	2.604787		C	-4.505639	-0.903363	1.102399
C	-1.16685	-0.986221	2.62611		C	-3.481051	0.96206	1.860874
C	-0.329459	-1.700373	3.486374		C	-4.8418	-2.208849	0.748226
C	-0.895682	-2.710917	4.274677		C	-5.306518	0.186911	0.711315
C	-2.257765	-2.974954	4.232381		C	-2.638446	1.878142	2.485381
C	-3.087021	-2.218647	3.4162		C	-4.645581	1.38237	1.186846
H	-0.759382	-0.237352	1.957508		C	-6.004797	-2.406493	0.022765
H	-0.264673	-3.294662	4.939018		H	-4.201435	-3.043011	1.007978
H	-2.682871	-3.754549	4.856683		C	-6.490773	-0.03161	0.012472
H	-4.158838	-2.38141	3.414929		C	-2.966705	3.222401	2.397841
C	2.400931	-2.927018	2.950028		H	-1.763282	1.555899	3.033268
C	2.798624	-3.979075	3.784001		C	-4.95168	2.738302	1.103905
C	2.547358	-3.099722	1.568515		C	-6.83983	-1.331467	-0.326058
C	3.314303	-5.159088	3.258311		H	-6.286518	-3.406879	-0.286913
H	2.70656	-3.876046	4.861223		H	-7.138181	0.793598	-0.263281
C	3.049551	-4.27904	1.03612		C	-4.105468	3.654935	1.706461
H	2.271001	-2.295832	0.89346		H	-2.322342	3.951122	2.878945
C	3.43427	-5.312523	1.882236		H	-5.838895	3.068678	0.573696
H	3.619826	-5.959936	3.925165		H	-4.329081	4.714832	1.645238
H	3.149772	-4.381827	-0.036986		N	-8.042282	-1.588077	-1.03223
H	3.837999	-6.23112	1.46792		C	-9.035568	-2.482765	-0.640005
C	1.950935	-1.236637	5.456402		C	-8.418304	-1.018651	-2.246096
C	3.289974	-1.321683	5.861804		C	-9.119343	-3.258975	0.514125
C	0.993074	-0.934882	6.430125		C	-10.055627	-2.495222	-1.613863
C	3.657636	-1.124689	7.186679		C	-7.748323	-0.081478	-3.029074
H	4.059918	-1.551138	5.129241		C	-9.659488	-1.558139	-2.643795
C	1.354194	-0.734129	7.758711		C	-10.237955	-4.064021	0.668742
H	-0.053508	-0.85967	6.150769		H	-8.340476	-3.229236	1.267247
C	2.687114	-0.830794	8.139592		C	-11.169595	-3.314225	-1.435481
H	4.701227	-1.199161	7.477205		C	-8.342151	0.310202	-4.219517
H	0.592424	-0.502579	8.497074		H	-6.799491	0.33187	-2.713639
H	2.970789	-0.676062	9.176195		C	-10.237526	-1.14647	-3.843685
C	2.089031	0.146485	2.770629		C	-11.254041	-4.098164	-0.295529
C	3.354912	0.208269	2.175252		H	-10.327467	-4.678405	1.55922
C	1.361174	1.33887	2.877478		H	-11.961206	-3.332753	-2.178559
C	3.881471	1.412593	1.722876		C	-9.57608	-0.21316	-4.626951
H	3.942325	-0.698773	2.074656		H	-7.837038	1.038219	-4.846843
C	1.872776	2.544729	2.411647		H	-11.192721	-1.554699	-4.160079
H	0.387278	1.334569	3.357597		H	-12.115079	-4.740796	-0.143996
C	3.143148	2.584975	1.845816		H	-10.015252	0.115715	-5.563004
H	4.867911	1.438041	1.270443		Si	1.524347	-1.419038	3.634037
H	1.286365	3.454645	2.50109		C	3.963223	1.670679	-1.655285
H	3.551492	3.524351	1.48871		C	4.031106	0.288721	-1.831634
N	-3.39587	-0.432227	1.801448		C	5.212825	-0.33923	-2.22749

C	6.344259	0.462816	-2.428255		C	-0.733435	-0.04545	-1.007465
C	6.297361	1.836979	-2.221108		C	-2.445615	3.128903	-1.545091
C	5.10938	2.444081	-1.836578		C	-2.786255	0.704199	-1.710883
H	3.126722	-0.28411	-1.668237		C	0.199043	-0.969589	-0.635857
H	7.275689	0.006949	-2.752376		C	-2.056366	-0.436408	-1.454473
H	7.188011	2.439135	-2.370547		C	-3.773785	3.289187	-2.055727
H	5.057532	3.513907	-1.674045		H	-1.848765	3.99279	-1.300818
C	6.578284	-2.654559	-3.716457		C	-4.111354	0.900712	-2.244267
C	6.185573	-2.855919	-5.045135		C	-0.159017	-2.34656	-0.712807
C	7.942591	-2.750123	-3.412712		H	1.181047	-0.670861	-0.30021
C	7.119663	-3.132931	-6.036631		C	-2.380307	-1.837286	-1.544237
H	5.132449	-2.802036	-5.307165		C	-4.571027	2.182428	-2.407191
C	8.88162	-3.025801	-4.400521		H	-4.161721	4.293519	-2.180462
H	8.276235	-2.619216	-2.386731		H	-4.70749	0.037832	-2.513896
C	8.470323	-3.21648	-5.715278		C	-1.432389	-2.752547	-1.167734
H	6.793402	-3.287588	-7.060617		H	0.56728	-3.095594	-0.424798
H	9.934223	-3.09653	-4.143451		H	-3.364176	-2.141632	-1.880573
H	9.201243	-3.434792	-6.487995		H	-5.565599	2.354767	-2.808257
C	3.638064	-2.901892	-2.881386		H	-1.656212	-3.813649	-1.216876
C	3.327459	-4.237694	-2.592702		N	1.405179	5.481352	-0.521684
C	2.671204	-2.143496	-3.554416		C	2.381791	6.446144	-0.869685
C	2.098466	-4.787961	-2.937593		C	0.388632	6.12818	0.222715
H	4.057625	-4.860756	-2.083309		C	3.540953	6.330723	-1.633518
C	1.435963	-2.684474	-3.894789		C	1.981926	7.689075	-0.350891
H	2.873554	-1.107504	-3.806212		C	-0.760571	5.620487	0.825912
C	1.145144	-4.006848	-3.582433		C	0.728564	7.486969	0.346422
H	1.88226	-5.824302	-2.69612		C	4.295991	7.477621	-1.843017
H	0.6948	-2.067096	-4.392168		H	3.838437	5.380964	-2.048177
H	0.17666	-4.425507	-3.837443		C	2.754424	8.826185	-0.568322
C	5.883691	-2.905399	-0.754079		C	-1.562855	6.508649	1.532972
C	6.392928	-4.206367	-0.641223		H	-1.026567	4.57796	0.743818
C	5.790047	-2.139106	0.41309		C	-0.09039	8.358068	1.058017
C	6.772503	-4.726133	0.590731		C	3.917409	8.714331	-1.31385
H	6.510462	-4.819986	-1.531088		H	5.203197	7.404661	-2.434469
C	6.169372	-2.652102	1.648244		H	2.443584	9.784635	-0.164332
H	5.413122	-1.122815	0.353803		C	-1.242001	7.862986	1.649337
C	6.656299	-3.949792	1.739638		H	-2.46623	6.1344	2.00256
H	7.162628	-5.737519	0.655021		H	0.175171	9.40661	1.149879
H	6.079238	-2.039644	2.540457		H	4.533074	9.589547	-1.493495
H	6.945066	-4.356626	2.703613		H	-1.894951	8.526651	2.206569
C	2.672457	2.299595	-1.303808		N	0.328632	3.438581	-0.734717
C	1.46177	4.131171	-0.813191		N	1.612996	1.505187	-1.194693
C	0.456295	2.128515	-0.959946		N	2.649751	3.624749	-1.14065
N	-0.691245	1.374161	-0.999488		Si	5.318202	-2.206459	-2.40226
C	-1.971566	1.862002	-1.393098					

Supplementary Table 34. Atomic coordinates of SiCzCz:SiTrzCz2 (09, E = -4774.23401449 a.u.).

C	3.954009	0.862739	0.069722	C	4.976736	-1.267216	-0.723883
C	3.848415	2.19942	-0.299646	C	3.437394	-0.313537	-2.076361
C	3.517924	3.184458	0.637162	C	5.893922	-1.552973	0.28576
C	3.361892	2.797115	1.972229	C	4.742233	-2.175304	-1.775764
C	3.503865	1.466957	2.352209	C	2.487374	0.519483	-2.665431
C	3.776224	0.494753	1.402791	C	3.761866	-1.563055	-2.644768
H	4.007269	2.461014	-1.338376	C	6.537375	-2.777762	0.250312
H	3.097066	3.533078	2.723787	H	6.103576	-0.841864	1.075154
H	3.352992	1.179439	3.387279	C	5.381734	-3.411641	-1.785335
H	3.810957	-0.549878	1.680787	C	1.877367	0.081004	-3.831859
C	4.900665	5.861099	0.036407	H	2.233464	1.48134	-2.241491
C	5.983654	5.449719	0.82268	C	3.132644	-1.983837	-3.815403
C	5.07459	6.975521	-0.793945	C	6.270241	-3.712131	-0.762102
C	7.195244	6.130295	0.788118	H	7.262238	-3.034153	1.01514
H	5.879883	4.580054	1.465878	H	5.18479	-4.143157	-2.562205
C	6.283638	7.66071	-0.831685	C	2.190055	-1.157571	-4.405749
H	4.256182	7.30749	-1.427272	H	1.142653	0.718426	-4.31225
C	7.345754	7.238244	-0.039142	H	3.378054	-2.946228	-4.254211
H	8.024128	5.793909	1.403477	H	1.691556	-1.467559	-5.317914
H	6.399158	8.521841	-1.482856	N	6.90374	-4.979113	-0.720221
H	8.292093	7.769757	-0.06964	C	6.752292	-5.915305	0.297719
C	2.147489	5.757234	1.429044	C	7.714817	-5.526882	-1.708907
C	2.502048	6.936707	2.091526	C	6.008284	-5.826055	1.472179
C	0.941951	5.135856	1.789825	C	7.481703	-7.076488	-0.032953
C	1.685719	7.477625	3.08104	C	8.148826	-4.961042	-2.905227
H	3.432475	7.43645	1.837537	C	8.0996	-6.826922	-1.319233
C	0.121352	5.67387	2.773325	C	6.015099	-6.922268	2.321894
H	0.639528	4.215639	1.30066	H	5.447	-4.929652	1.712141
C	0.495473	6.846639	3.424108	C	7.474057	-8.163326	0.839966
H	1.98169	8.39205	3.586185	C	8.972764	-5.727438	-3.71592
H	-0.808096	5.174106	3.029318	H	7.854705	-3.956437	-3.187885
H	-0.141394	7.267955	4.196147	C	8.929177	-7.576164	-2.152399
C	2.427581	5.035969	-1.560523	C	6.741745	-8.080229	2.014487
C	3.090917	4.546639	-2.695579	H	5.443385	-6.880648	3.243778
C	1.114397	5.490992	-1.72229	H	8.031257	-9.06393	0.600009
C	2.452277	4.460542	-3.927274	C	9.360129	-7.023197	-3.348368
H	4.128689	4.231642	-2.623189	H	9.325818	-5.31174	-4.654522
C	0.474329	5.425614	-2.956243	H	9.233881	-8.578403	-1.866352
H	0.575632	5.885784	-0.866912	H	6.727135	-8.919402	2.702134
C	1.135945	4.893979	-4.057293	H	10.004792	-7.596042	-4.006849
H	2.983114	4.061125	-4.785859	Si	3.251793	4.978201	0.129057
H	-0.545889	5.784189	-3.056526	C	-4.112531	-1.546963	2.055422
H	0.633865	4.831754	-5.018157	C	-2.877933	-0.912551	1.928938
N	4.186731	-0.135844	-0.909303	C	-1.933758	-0.919509	2.95654

C	-2.262989	-1.610516	4.128785		C	-3.993704	0.567088	-2.5189
C	-3.479794	-2.274411	4.257451		C	-7.590942	0.962784	-2.993924
C	-4.409256	-2.243831	3.226298		C	-5.433397	1.731574	-3.880791
H	-2.685951	-0.372997	1.010911		C	-2.783082	0.098235	-2.101952
H	-1.568686	-1.627772	4.963295		C	-4.099849	1.523902	-3.605307
H	-3.708748	-2.809639	5.173619		C	-8.221265	1.810307	-3.955128
H	-5.36314	-2.750209	3.318033		H	-8.185683	0.378128	-2.311015
C	0.392505	0.750125	4.20761		C	-6.101748	2.568188	-4.846664
C	0.626593	2.131809	4.20811		C	-1.611182	0.601498	-2.744179
C	0.709965	0.041442	5.375016		H	-2.700463	-0.627165	-1.308234
C	1.161923	2.779608	5.315462		C	-2.894626	2.057748	-4.193347
H	0.398383	2.714681	3.323373		C	-7.472696	2.588749	-4.86157
C	1.247696	0.681097	6.486603		H	-9.304275	1.846194	-3.981875
H	0.544174	-1.031301	5.42568		H	-5.517562	3.162916	-5.539752
C	1.477572	2.052947	6.457969		C	-1.686454	1.585483	-3.753411
H	1.330395	3.852035	5.278584		H	-0.643867	0.228246	-2.432394
H	1.487633	0.107471	7.376715		H	-2.962296	2.809937	-4.971251
H	1.898866	2.552448	7.325069		H	-7.995853	3.211947	-5.58002
C	-0.618544	1.288477	1.379328		H	-0.767677	1.980713	-4.176407
C	0.246313	1.555432	0.312028		N	-8.463848	-2.178155	0.289535
C	-1.78211	2.065453	1.484926		C	-9.085006	-2.566454	1.502396
C	-0.061513	2.526612	-0.63459		C	-9.387983	-2.379167	-0.76489
H	1.167896	0.993478	0.209522		C	-8.619813	-2.526317	2.814513
C	-2.092838	3.038733	0.54278		C	-10.391521	-2.995417	1.213065
H	-2.47487	1.889103	2.303024		C	-9.246743	-2.210168	-2.140329
C	-1.23597	3.261271	-0.528223		C	-10.58005	-2.885244	-0.219285
H	0.613025	2.715979	-1.461191		C	-9.483298	-2.940963	3.820647
H	-3.010424	3.611099	0.636878		H	-7.620987	-2.186594	3.037723
H	-1.480136	4.000554	-1.283015		C	-11.239669	-3.411186	2.235052
C	0.91737	-1.330563	1.962886		C	-10.332445	-2.527383	-2.94735
C	1.870174	-1.971968	2.763137		H	-8.3271	-1.842565	-2.567667
C	0.848844	-1.703306	0.613381		C	-11.656806	-3.195274	-1.044493
C	2.724259	-2.937501	2.238765		C	-10.778344	-3.385238	3.541879
H	1.969068	-1.700113	3.809223		H	-9.135791	-2.918702	4.848729
C	1.710685	-2.653456	0.078348		H	-12.247743	-3.744179	2.008279
H	0.119504	-1.233476	-0.040494		C	-11.530096	-3.008236	-2.412313
C	2.653683	-3.273692	0.89139		H	-10.239424	-2.396352	-4.02076
H	3.456608	-3.417908	2.881469		H	-12.577353	-3.583616	-0.619919
H	1.653355	-2.901543	-0.976364		H	-11.424898	-3.706406	4.351954
H	3.334281	-4.008724	0.473517		H	-12.359779	-3.243057	-3.071014
C	-5.102777	-1.403347	0.967016		N	-6.916182	-0.971564	-0.943289
C	-7.188262	-1.66371	0.161631		N	-4.709521	-0.750384	-0.12413
C	-5.65867	-0.531438	-1.034624		N	-6.330255	-1.891382	1.157756
N	-5.319138	0.227905	-2.128453		Si	-0.285055	-0.056524	2.650031
C	-6.227556	0.92403	-2.972268					

Supplementary Table 35. Atomic coordinates of SiCzCz:SiTrzCz2 (10, E = -4774.23673046 a.u.).

C	-4.74359	0.339116	-1.491832	C	-7.158638	0.842616	-1.077535
C	-3.822801	-0.457849	-0.822725	C	-6.651883	-1.245406	-1.778757
C	-2.451059	-0.195029	-0.891515	C	-7.114467	2.162178	-0.632347
C	-2.03164	0.929221	-1.607015	C	-8.376575	0.135628	-1.167215
C	-2.953774	1.75028	-2.251243	C	-6.004089	-2.397816	-2.220637
C	-4.308376	1.450236	-2.213634	C	-8.051894	-1.200502	-1.619324
H	-4.191228	-1.300443	-0.24499	C	-8.31004	2.767976	-0.28373
H	-0.97408	1.16629	-1.666101	H	-6.174213	2.692723	-0.538791
H	-2.612346	2.618974	-2.805664	C	-9.570707	0.770321	-0.837541
H	-5.030433	2.059299	-2.747079	C	-6.786452	-3.510239	-2.489173
C	-1.988467	-1.711989	1.650392	H	-4.928311	-2.431426	-2.345289
C	-2.107092	-0.631849	2.537742	C	-8.816636	-2.332974	-1.897267
C	-2.568004	-2.928289	2.029543	C	-9.531248	2.084877	-0.39291
C	-2.764378	-0.761896	3.753756	H	-8.315493	3.785905	0.090558
H	-1.697609	0.337652	2.267144	H	-10.523379	0.257512	-0.918803
C	-3.237198	-3.061771	3.242162	C	-8.178766	-3.484545	-2.329461
H	-2.514147	-3.783703	1.365301	H	-6.304137	-4.421472	-2.828905
C	-3.336993	-1.979197	4.10659	H	-9.895596	-2.309877	-1.777545
H	-2.839153	0.090096	4.422299	H	-8.759706	-4.374718	-2.546895
H	-3.688525	-4.013849	3.506057	N	-10.733254	2.744026	-0.028879
H	-3.862348	-2.080913	5.051554	C	-11.228086	3.910372	-0.602999
C	0.433227	-0.661808	-0.068307	C	-11.611718	2.33613	0.970316
C	1.113129	-0.526331	-1.288112	C	-10.704534	4.669055	-1.647489
C	1.010375	-0.086464	1.069496	C	-12.438755	4.25867	0.032091
C	2.303315	0.18623	-1.374859	C	-11.524159	1.241563	1.827472
H	0.709239	-0.986665	-2.186199	C	-12.684789	3.249099	1.040677
C	2.202489	0.627247	0.986878	C	-11.41047	5.796693	-2.039237
H	0.530639	-0.196653	2.036629	H	-9.780264	4.383554	-2.137023
C	2.846246	0.775186	-0.236791	C	-13.128896	5.397434	-0.380857
H	2.815785	0.278508	-2.327545	C	-12.541395	1.067986	2.753926
H	2.633351	1.066216	1.88153	H	-10.687682	0.55403	1.772945
H	3.770912	1.337315	-0.305679	C	-13.695292	3.051077	1.980644
C	-1.384098	-3.004048	-1.076495	C	-12.609799	6.162948	-1.413506
C	-0.904914	-4.222698	-0.576949	H	-11.024913	6.40642	-2.850488
C	-2.014841	-3.016579	-2.327288	H	-14.061628	5.678155	0.09923
C	-1.085711	-5.409669	-1.278609	C	-13.619931	1.959556	2.831811
H	-0.396072	-4.256463	0.380876	H	-12.49825	0.222929	3.434027
C	-2.188749	-4.199041	-3.039977	H	-14.527843	3.745249	2.045706
H	-2.387805	-2.088723	-2.749904	H	-13.1362	7.052374	-1.744054
C	-1.736053	-5.401753	-2.509035	H	-14.400278	1.793115	3.567217
H	-0.716822	-6.338836	-0.858513	Si	-1.278673	-1.420674	-0.065501
H	-2.685054	-4.180516	-4.005756	C	5.746734	0.154954	2.283105
H	-1.88315	-6.329278	-3.054236	C	6.329138	1.046139	1.387114
N	-6.118117	0.001562	-1.453891	C	6.955335	2.219611	1.80581

C	6.993514	2.479219	3.17843		C	5.537618	-1.632285	-2.293489
C	6.418979	1.591816	4.089039		C	2.433887	-3.544069	-2.096889
C	5.786636	0.435188	3.649156		C	3.911321	-2.427119	-3.708689
H	6.248075	0.815243	0.331559		C	6.727135	-1.045143	-1.972998
H	7.46073	3.388651	3.545374		C	5.08304	-1.703864	-3.668922
H	6.456114	1.812427	5.151283		C	1.584226	-3.874139	-3.196346
H	5.322449	-0.251374	4.349006		H	2.154145	-3.823486	-1.094574
C	8.159489	4.972144	1.010852		C	3.038465	-2.787392	-4.799366
C	7.641131	6.174432	0.517953		C	7.498696	-0.461704	-3.024447
C	9.171341	5.041596	1.979369		H	7.084244	-1.002812	-0.957085
C	8.105375	7.401115	0.982339		C	5.868628	-1.080094	-4.706214
H	6.865282	6.154806	-0.241303		C	1.898652	-3.493634	-4.517908
C	9.636328	6.26323	2.449257		H	0.67268	-4.421001	-2.998006
H	9.61072	4.126043	2.368639		H	3.290894	-2.491682	-5.811409
C	9.100634	7.447115	1.951137		C	7.051927	-0.477194	-4.362194
H	7.689217	8.322142	0.585559		H	8.446138	0.00215	-2.774459
H	10.41957	6.293413	3.200521		H	5.522893	-1.10729	-5.73346
H	9.462766	8.403696	2.315357		H	1.220087	-3.769775	-5.318961
C	6.146499	3.433471	-0.80403		H	7.663341	-0.009843	-5.127873
C	6.134775	2.697291	-1.994288		N	2.37852	-3.156572	2.521956
C	4.988933	4.151796	-0.469508		C	1.697528	-2.612631	3.637729
C	5.014822	2.673974	-2.820323		C	1.754155	-4.370317	2.170121
H	7.004591	2.116195	-2.279874		C	1.908653	-1.428425	4.33977
C	3.873284	4.146615	-1.297518		C	0.634478	-3.470669	3.967586
H	4.95239	4.716905	0.45863		C	2.10641	-5.316746	1.211429
C	3.883827	3.403883	-2.475299		C	0.67427	-4.587177	3.04461
H	5.030676	2.075295	-3.726267		C	1.037433	-1.131718	5.38034
H	2.987729	4.708592	-1.017266		H	2.728517	-0.774324	4.087129
H	3.005855	3.385603	-3.113699		C	-0.217572	-3.161049	5.022509
C	8.992098	2.398617	-0.455335		C	1.33435	-6.46859	1.125873
C	9.610362	2.983163	-1.569061		H	2.949358	-5.159742	0.554878
C	9.433796	1.134338	-0.050946		C	-0.099343	-5.738784	2.929159
C	10.620261	2.326175	-2.260889		C	-0.012268	-1.986028	5.72702
H	9.294258	3.967482	-1.90605		H	1.185888	-0.213346	5.939742
C	10.448542	0.472117	-0.735951		H	-1.039785	-3.821896	5.273311
H	8.977526	0.655041	0.809574		C	0.234141	-6.677662	1.963784
C	11.040896	1.065844	-1.844759		H	1.59486	-7.218782	0.385926
H	11.081998	2.796751	-3.123559		H	-0.941307	-5.900793	3.594655
H	10.774186	-0.509112	-0.404294		H	-0.673356	-1.724111	6.546438
H	11.830412	0.550026	-2.382826		H	-0.354555	-7.583804	1.86419
C	5.017795	-0.997247	1.714799		N	3.492299	-2.828099	0.513147
C	3.373735	-2.519362	1.803241		N	5.263921	-1.270311	0.44046
C	4.443851	-2.149665	-0.133793		N	4.104485	-1.617382	2.460988
N	4.56722	-2.305719	-1.49265		Si	7.582751	3.299128	0.402038
C	3.566823	-2.833578	-2.3568					

Supplementary Table 36. Atomic coordinates of SiCzCz:SiTrzCz2 (11, E = -4774.24978137 a.u.).

C	-3.251291	4.823157	-0.109761		C	-4.627738	2.752078	-0.066408
C	-1.980344	4.311445	-0.359409		C	-5.454906	4.485539	-1.259575
C	-0.831416	4.998018	0.035715		C	-3.860542	1.921205	0.746193
C	-0.997883	6.226302	0.691638		C	-5.868777	2.330902	-0.587272
C	-2.263263	6.755128	0.915742		C	-5.643064	5.7012	-1.912782
C	-3.397262	6.053657	0.523043		C	-6.395753	3.438899	-1.357037
H	-1.902638	3.354589	-0.86244		C	-4.3443	0.653059	1.015405
H	-0.129768	6.77478	1.045691		H	-2.905599	2.237309	1.148088
H	-2.371384	7.708832	1.422605		C	-6.354418	1.063379	-0.278944
H	-4.390932	6.439248	0.724632		C	-6.803352	5.857471	-2.656365
C	1.547456	3.809799	1.482424		H	-4.906092	6.493537	-1.847026
C	2.919845	3.756016	1.753877		C	-7.554167	3.62119	-2.11077
C	0.663195	3.409751	2.493361		C	-5.582752	0.226345	0.51581
C	3.394285	3.305286	2.981272		H	-3.754415	-0.030597	1.610304
H	3.631957	4.069863	0.99587		H	-7.320534	0.729463	-0.641396
C	1.130234	2.959222	3.722791		C	-7.753591	4.832162	-2.755039
H	-0.408715	3.452273	2.318266		H	-6.975739	6.795174	-3.175208
C	2.497975	2.905855	3.966191		H	-8.286073	2.823406	-2.193572
H	4.461787	3.256754	3.170036		H	-8.650785	4.988943	-3.34456
H	0.428326	2.647828	4.490828		N	-6.048278	-1.073566	0.835366
H	2.86871	2.55145	4.921539		C	-6.185053	-1.59523	2.118999
C	2.033554	5.407028	-1.129485		C	-6.540312	-2.005518	-0.073589
C	3.230095	4.887794	-1.645368		C	-5.859211	-1.023048	3.347165
C	1.7745	6.767199	-1.328965		C	-6.766227	-2.878347	2.035153
C	4.14272	5.69844	-2.308757		C	-6.570885	-1.948211	-1.465485
H	3.446641	3.827633	-1.54155		C	-7.009272	-3.134081	0.631561
C	2.681063	7.582721	-1.998325		C	-6.096295	-1.771775	4.490242
H	0.846142	7.199786	-0.970702		H	-5.433169	-0.028739	3.40647
C	3.869883	7.051149	-2.483773		C	-6.990031	-3.612995	3.198757
H	5.062893	5.272917	-2.697362		C	-7.113061	-3.031041	-2.140654
H	2.456282	8.635081	-2.14355		H	-6.160623	-1.102125	-2.00281
H	4.578629	7.687008	-3.005535		C	-7.565892	-4.202362	-0.070667
C	0.761818	2.656934	-1.203328		C	-6.648769	-3.058162	4.422098
C	1.501122	1.539811	-0.80018		H	-5.849696	-1.348142	5.459062
C	0.015323	2.552084	-2.386383		H	-7.427593	-4.605336	3.145009
C	1.497252	0.368333	-1.547342		C	-7.619471	-4.144247	-1.455174
H	2.088331	1.577886	0.112712		H	-7.136013	-3.014004	-3.225553
C	-0.023252	1.368514	-3.114728		H	-7.944603	-5.070101	0.461502
H	-0.55273	3.406442	-2.745158		H	-6.815504	-3.619278	5.335725
C	0.722233	0.270175	-2.696099		H	-8.047007	-4.96977	-2.014931
H	2.082467	-0.474764	-1.210973		Si	0.874366	4.247315	-0.214915
H	-0.635919	1.300627	-4.008086		C	2.242958	-3.088854	-1.414719
H	0.696551	-0.659445	-3.25595		C	0.974066	-2.899788	-0.860268
N	-4.385466	4.061017	-0.475266		C	-0.18581	-3.214409	-1.563677

C	-0.038545	-3.769201	-2.843308
C	1.217996	-4.001039	-3.391021
C	2.362183	-3.653038	-2.683763
H	0.910337	-2.448908	0.121522
H	-0.920132	-4.018992	-3.427504
H	1.307059	-4.439675	-4.379877
H	3.348641	-3.806457	-3.105459
C	-2.904776	-1.921071	-2.102756
C	-3.02996	-0.530885	-1.99273
C	-3.500639	-2.544883	-3.207017
C	-3.720703	0.21076	-2.94526
H	-2.594377	-0.012345	-1.146947
C	-4.183624	-1.809243	-4.168891
H	-3.448907	-3.62559	-3.309846
C	-4.295854	-0.427969	-4.038772
H	-3.815929	1.285625	-2.825521
H	-4.636399	-2.314879	-5.016738
H	-4.835554	0.148075	-4.784604
C	-1.767223	-1.956734	0.758714
C	-2.457599	-2.379548	1.901697
C	-0.996117	-0.788336	0.853514
C	-2.382858	-1.662776	3.093372
H	-3.072232	-3.273875	1.861007
C	-0.906074	-0.074907	2.042992
H	-0.44574	-0.427026	-0.009167
C	-1.604127	-0.511532	3.165518
H	-2.944627	-1.999997	3.958507
H	-0.279977	0.81023	2.096573
H	-1.532737	0.041748	4.09716
C	-2.674547	-4.589467	-0.551248
C	-4.056136	-4.807243	-0.608751
C	-1.85043	-5.670138	-0.207198
C	-4.593492	-6.060012	-0.33051
H	-4.725013	-3.996979	-0.878612
C	-2.383648	-6.921198	0.075902
H	-0.773459	-5.532873	-0.165621
C	-3.759465	-7.117185	0.012914
H	-5.666804	-6.202614	-0.386542
H	-1.726501	-7.744251	0.340339
H	-4.180242	-8.094896	0.22875
C	3.431262	-2.577188	-0.698088
C	5.599702	-1.971288	-0.695743
C	4.309679	-1.518619	1.089008
N	4.153541	-0.97355	2.339266
C	5.056639	-0.081498	2.977193

C	3.078214	-1.240266	3.234065
C	6.171159	0.556192	2.515258
C	4.5465	0.161551	4.313499
C	1.973445	-2.027849	3.111185
C	3.361042	-0.522604	4.46344
C	6.84903	1.446351	3.40157
H	6.541355	0.397513	1.515245
C	5.272102	1.042477	5.195256
C	1.087925	-2.120931	4.229728
H	1.759638	-2.566439	2.203238
C	2.432014	-0.613733	5.563917
C	6.398023	1.665778	4.720228
H	7.739912	1.951808	3.046885
H	4.922353	1.199061	6.209598
C	1.324534	-1.409973	5.423361
H	0.20484	-2.740313	4.136093
H	2.625299	-0.065124	6.478964
H	6.956631	2.337102	5.364844
H	0.615035	-1.499352	6.240053
N	6.827028	-1.92456	-1.326018
C	7.048937	-2.050143	-2.71991
C	8.077412	-1.71078	-0.695877
C	6.150356	-2.221197	-3.770035
C	8.425634	-1.908336	-2.963059
C	8.403263	-1.581831	0.652025
C	9.076149	-1.703596	-1.684387
C	6.663999	-2.266777	-5.059932
H	5.09206	-2.315929	-3.585092
C	8.920714	-1.961803	-4.262601
C	9.742955	-1.417098	0.981598
H	7.642769	-1.602656	1.416687
C	10.412336	-1.535411	-1.333687
C	8.033167	-2.145538	-5.311332
H	5.97817	-2.401546	-5.890363
H	9.984956	-1.854087	-4.44767
C	10.74151	-1.386564	0.004826
H	10.012554	-1.310217	2.027525
H	11.182174	-1.528029	-2.099045
H	8.40049	-2.190355	-6.331348
H	11.778053	-1.252881	0.29658
N	5.509543	-1.417777	0.511261
N	3.243672	-2.096138	0.530202
N	4.595188	-2.558425	-1.349312
Si	-1.906734	-2.907998	-0.853814

Supplementary Table 37. Atomic coordinates of SiCzCz:SiTrzCz2 (12, E = -4774.21243473 a.u.).

C	8.338581	0.303161	0.793277	C	6.217846	-0.441652	-0.27547
C	9.678966	0.167318	0.447416	C	7.401958	1.171838	-1.359609
C	10.682523	0.20918	1.417794	C	5.760372	-1.422503	0.573531
C	10.299771	0.340039	2.757429	C	5.498755	-0.075091	-1.49717
C	8.957179	0.440755	3.109843	C	8.321266	2.119503	-1.703613
C	7.972392	0.440165	2.131007	C	6.236588	0.908496	-2.144957
H	9.931009	0.030459	-0.598906	C	4.553795	-2.064613	0.247438
H	11.057246	0.363169	3.535035	H	6.319904	-1.71508	1.453584
H	8.675761	0.542755	4.153247	C	4.259671	-0.727692	-1.787626
H	6.926231	0.558585	2.391059	C	8.093122	2.856354	-2.929581
C	13.566245	-0.359068	2.277841	H	9.189294	2.332962	-1.091739
C	14.75198	0.302414	2.616426	C	6.032396	1.652548	-3.353228
C	13.225911	-1.509978	3.00269	C	3.812887	-1.694983	-0.926447
C	15.572974	-0.170052	3.634664	H	4.191457	-2.887592	0.849279
H	15.035932	1.202827	2.07958	H	3.681456	-0.450359	-2.659757
C	14.043104	-1.987348	4.02061	C	6.980272	2.614073	-3.714581
H	12.303038	-2.037965	2.775356	H	8.817273	3.607223	-3.224695
C	15.220261	-1.316693	4.337081	H	5.157394	1.469936	-3.966484
H	16.487984	0.359477	3.882009	H	6.83828	3.182187	-4.628472
H	13.760486	-2.880554	4.569402	N	2.616609	-2.389231	-1.189487
H	15.859582	-1.686181	5.133073	C	1.585686	-2.60571	-0.270926
C	12.899724	2.015068	0.415089	C	2.234294	-2.937315	-2.41714
C	14.009874	2.301989	-0.390926	C	1.4673	-2.17062	1.047784
C	12.123586	3.08851	0.868136	C	0.549485	-3.316294	-0.906191
C	14.338786	3.610981	-0.723229	C	2.925056	-2.981904	-3.625899
H	14.620264	1.489425	-0.776597	C	0.961659	-3.526207	-2.277096
C	12.444976	4.400019	0.534765	C	0.307456	-2.496016	1.733928
H	11.252405	2.897829	1.488307	H	2.245393	-1.579405	1.515631
C	13.554752	4.662879	-0.260379	C	-0.601514	-3.643937	-0.193713
H	15.203181	3.810581	-1.349226	C	2.303645	-3.599791	-4.702198
H	11.827222	5.21715	0.894732	H	3.914612	-2.552763	-3.723327
H	13.80665	5.68587	-0.522958	C	0.357011	-4.138947	-3.37162
C	12.705626	-0.827434	-0.648219	C	-0.713668	-3.238253	1.12693
C	13.216213	-2.127658	-0.563618	H	0.179467	-2.154208	2.755367
C	12.318755	-0.355076	-1.91042	H	-1.399171	-4.204781	-0.67257
C	13.326174	-2.931564	-1.692949	C	1.029033	-4.167362	-4.584161
H	13.539892	-2.516951	0.397301	H	2.821066	-3.642835	-5.655459
C	12.42145	-1.15587	-3.041631	H	-0.627692	-4.585993	-3.274416
H	11.940287	0.658518	-2.015792	H	-1.605524	-3.478263	1.695203
C	12.925246	-2.447843	-2.933236	H	0.568788	-4.637086	-5.447243
H	13.727421	-3.936623	-1.604864	Si	12.482186	0.244265	0.87439
H	12.112106	-0.77029	-4.008222	C	-6.707934	-1.129127	-0.056454
H	13.009443	-3.07495	-3.815553	C	-5.622943	-0.268263	0.121536
N	7.358166	0.327873	-0.225856	C	-4.310259	-0.737355	0.073297

C	-4.11007	-2.101489	-0.184118		C	-8.806648	3.461285	-0.581402
C	-5.183826	-2.965986	-0.367234		C	-12.037885	2.727097	0.986266
C	-6.48402	-2.484962	-0.296313		C	-10.731735	4.55701	0.052317
H	-5.825776	0.779689	0.311049		C	-7.538583	3.297161	-1.132739
H	-3.097578	-2.489456	-0.244274		C	-9.422085	4.724234	-0.546049
H	-5.006694	-4.01806	-0.568238		C	-13.033878	3.649593	1.283178
H	-7.332874	-3.143485	-0.440398		H	-12.156314	1.684737	1.236844
C	-2.238012	0.055253	2.167049		C	-11.741317	5.464806	0.356836
C	-1.288824	0.899218	2.758916		C	-6.904264	4.423474	-1.641038
C	-2.700999	-1.030523	2.917868		H	-7.058779	2.33189	-1.157587
C	-0.79348	0.64888	4.032978		C	-8.765273	5.841996	-1.05151
H	-0.937575	1.776818	2.222517		C	-12.897213	5.003742	0.968613
C	-2.218891	-1.281829	4.198364		H	-13.937599	3.300769	1.772662
H	-3.451892	-1.692575	2.497567		H	-11.619074	6.517833	0.12299
C	-1.256172	-0.446962	4.75519		C	-7.501402	5.685472	-1.59969
H	-0.053373	1.31416	4.467318		H	-5.912411	4.311482	-2.063675
H	-2.596312	-2.129615	4.762405		H	-9.24218	6.816856	-1.022046
H	-0.876304	-0.641818	5.753603		H	-13.695562	5.697408	1.211382
C	-3.369837	2.194248	0.348047		H	-6.9741	6.545118	-2.000784
C	-2.951248	3.035861	-0.688679		N	-11.372181	-1.806245	0.323368
C	-4.176426	2.743897	1.354689		C	-11.301101	-3.15793	0.747657
C	-3.320314	4.377239	-0.718152		C	-12.732562	-1.490482	0.077558
H	-2.317455	2.64019	-1.476762		C	-10.211561	-3.93303	1.138088
C	-4.562495	4.077803	1.321048		C	-12.605705	-3.678824	0.782409
H	-4.502393	2.121453	2.184364		C	-13.321338	-0.32568	-0.408637
C	-4.12934	4.898612	0.284893		C	-13.50776	-2.629615	0.352231
H	-2.975233	5.015689	-1.526275		C	-10.458551	-5.238001	1.546433
H	-5.198659	4.479367	2.103481		H	-9.210256	-3.533062	1.119308
H	-4.429502	5.941508	0.257368		C	-12.831403	-4.989525	1.191201
C	-1.518661	0.095472	-0.865321		C	-14.700103	-0.319904	-0.581389
C	-0.187734	0.456747	-0.62755		H	-12.731511	0.5465	-0.640592
C	-1.843905	-0.399081	-2.135351		C	-14.887499	-2.603295	0.17405
C	0.777621	0.34985	-1.621212		C	-11.750216	-5.769385	1.571177
H	0.108259	0.818285	0.352238		H	-9.620264	-5.856402	1.851454
C	-0.87983	-0.523542	-3.128952		H	-13.840436	-5.389193	1.215121
H	-2.865756	-0.697484	-2.351343		C	-15.482301	-1.439715	-0.289016
C	0.432438	-0.141639	-2.874811		H	-15.173446	0.582883	-0.9541
H	1.804389	0.630295	-1.409092		H	-15.483895	-3.484421	0.389377
H	-1.149188	-0.926011	-4.100437		H	-11.906535	-6.794101	1.892001
H	1.185873	-0.246943	-3.649264		H	-16.557148	-1.400009	-0.432338
C	-8.086189	-0.600831	0.013345		N	-10.568764	0.362898	0.15506
C	-10.304218	-0.94213	0.199636		N	-8.241579	0.72109	-0.041482
C	-9.50119	1.150575	0.027186		N	-9.084385	-1.476767	0.129324
N	-9.71142	2.510939	-0.040289		Si	-2.849344	0.392195	0.421071
C	-10.889861	3.193146	0.350936					

Supplementary Table 38. Atomic coordinates of SiCzCz:SiTrzCz2 (13, E = -4774.21695465 a.u.).

C	-8.950319	-0.277482	-0.912898		C	-6.52226	-0.390478	-0.378952
C	-10.073608	0.203632	-0.24933		C	-7.440678	1.688841	-0.538955
C	-11.351471	-0.280073	-0.534413		C	-6.272419	-1.740858	-0.30421
C	-11.471156	-1.30899	-1.475303		C	-5.480658	0.602791	-0.112784
C	-10.346659	-1.815766	-2.120136		C	-8.284575	2.745295	-0.722844
C	-9.0866	-1.291854	-1.859578		C	-6.056519	1.861777	-0.220118
H	-9.939046	0.986511	0.487156		C	-4.964025	-2.153571	0.003601
H	-12.451471	-1.704101	-1.724722		H	-7.057325	-2.472088	-0.451494
H	-10.453937	-2.606883	-2.855689		C	-4.153416	0.151834	0.174362
H	-8.21508	-1.647713	-2.39748		C	-7.737781	4.075904	-0.559774
C	-13.190226	-0.344364	1.940745		H	-9.329854	2.622738	-0.975113
C	-13.911181	0.296835	2.957217		C	-5.537497	3.190639	-0.069038
C	-12.786135	-1.667017	2.155167		C	-3.917178	-1.195672	0.223051
C	-14.225285	-0.361895	4.140323		H	-4.738702	-3.203888	0.134656
H	-14.22331	1.330053	2.827686		H	-3.351432	0.861191	0.329365
C	-13.096407	-2.33042	3.33707		C	-6.404911	4.271005	-0.242119
H	-12.214797	-2.184266	1.389416		H	-8.404061	4.92206	-0.681833
C	-13.818189	-1.678449	4.330698		H	-4.493067	3.343358	0.178094
H	-14.783823	0.152971	4.91607		H	-6.026054	5.281249	-0.12328
H	-12.771255	-3.355742	3.484551		N	-2.63337	-1.689712	0.527701
H	-14.059627	-2.194014	5.255223		C	-1.957376	-2.677701	-0.190083
C	-14.304643	0.46326	-0.816293		C	-1.821058	-1.284551	1.591016
C	-15.590713	0.22644	-0.317291		C	-2.329015	-3.331209	-1.362864
C	-14.166991	0.691644	-2.19273		C	-0.716659	-2.927136	0.429799
C	-16.699317	0.226278	-1.15671		C	-2.057937	-0.332499	2.579157
H	-15.728032	0.03314	0.74265		C	-0.628697	-2.035216	1.566152
C	-15.271695	0.693949	-3.036343		C	-1.453862	-4.275697	-1.879803
H	-13.180249	0.860043	-2.616307		H	-3.265514	-3.104324	-1.858264
C	-16.541414	0.462082	-2.517737		C	0.145076	-3.881259	-0.10448
H	-17.687521	0.03831	-0.748104		C	-1.063177	-0.125749	3.525471
H	-15.141662	0.872663	-4.099389		H	-2.985438	0.226276	2.609721
H	-17.405782	0.460828	-3.174807		C	0.354173	-1.814365	2.526739
C	-12.305866	2.301283	0.660156		C	-0.233894	-4.56017	-1.254128
C	-11.571892	2.633116	1.806876		H	-1.722757	-4.802504	-2.790008
C	-12.504187	3.299687	-0.301271		H	1.099879	-4.080243	0.37141
C	-11.036697	3.905149	1.977257		C	0.134321	-0.850912	3.499429
H	-11.410031	1.884533	2.577954		H	-1.222062	0.615714	4.302434
C	-11.975113	4.575153	-0.134677		H	1.278788	-2.379973	2.509013
H	-13.081368	3.079129	-1.194836		H	0.42288	-5.31406	-1.6773
C	-11.234479	4.877929	1.003266		H	0.897802	-0.659894	4.246279
H	-10.462631	4.136495	2.869051		Si	-12.819264	0.525445	0.325261
H	-12.142414	5.333967	-0.893163		C	6.547051	1.557939	-0.993352
H	-10.816772	5.871594	1.133924		C	5.619966	0.530922	-0.827537
N	-7.689258	0.297376	-0.636724		C	4.311706	0.637338	-1.299332

C	3.937996	1.832793	-1.92197		C	9.124047	-2.555682	0.25866
C	4.846408	2.879853	-2.065403		C	12.028467	-0.975762	1.846977
C	6.15157	2.745834	-1.609744		C	10.999705	-3.147531	1.458963
H	5.942421	-0.365224	-0.310451		C	7.945751	-2.739279	-0.462828
H	2.924518	1.949116	-2.295755		C	9.806667	-3.653264	0.808981
H	4.536655	3.802077	-2.546877		C	13.029318	-1.62818	2.557193
H	6.874771	3.543296	-1.739901		H	12.036592	0.096828	1.737499
C	1.420088	-0.381861	-1.415035		C	12.011811	-3.78176	2.172345
C	0.742158	0.378408	-0.453508		C	7.457316	-4.032938	-0.597308
C	0.741399	-0.701813	-2.596793		H	7.422651	-1.906541	-0.902073
C	-0.559445	0.811275	-0.668378		C	9.301762	-4.942276	0.665193
H	1.22668	0.62424	0.48708		C	13.031736	-3.015693	2.716439
C	-0.569034	-0.287604	-2.807166		H	13.825499	-1.036015	2.997056
H	1.238083	-1.291144	-3.361125		H	11.995954	-4.859257	2.303622
C	-1.22083	0.473688	-1.84439		C	8.120308	-5.127133	-0.03653
H	-1.060754	1.396089	0.096265		H	6.53966	-4.183201	-1.15703
H	-1.082598	-0.561941	-3.72339		H	9.830991	-5.787387	1.09456
H	-2.246775	0.790697	-2.002558		H	13.830333	-3.493814	3.274224
C	3.498245	-1.531776	0.608397		H	7.711617	-6.125307	-0.156204
C	3.670574	-2.898851	0.855586		N	10.930884	3.106132	-0.060953
C	3.597862	-0.65581	1.697042		C	10.618993	4.487696	0.010652
C	3.915745	-3.375865	2.139305		C	12.34045	2.967974	-0.016571
H	3.62979	-3.602808	0.029935		C	9.389845	5.142358	0.031655
C	3.844457	-1.124066	2.98225		C	11.821422	5.206637	0.120215
H	3.501046	0.414894	1.538952		C	13.144764	1.836217	-0.124921
C	4.000905	-2.487986	3.20663		C	12.908126	4.248379	0.094308
H	4.054176	-4.440042	2.304011		C	9.396095	6.526616	0.151365
H	3.921546	-0.423589	3.808293		H	8.466113	4.591439	-0.048241
H	4.199798	-2.856889	4.208117		C	11.80605	6.593251	0.235443
C	3.753411	-2.184774	-2.350814		C	14.522255	2.013335	-0.081918
C	2.950141	-3.307159	-2.597695		H	12.716778	0.852576	-0.23568
C	5.009984	-2.133764	-2.965878		C	14.290155	4.404891	0.137162
C	3.391061	-4.343502	-3.412829		C	10.586301	7.251675	0.248427
H	1.963119	-3.373779	-2.15043		H	8.446973	7.05301	0.16689
C	5.457932	-3.169684	-3.779518		H	12.736769	7.14579	0.318563
H	5.652133	-1.27241	-2.81017		C	15.095978	3.279657	0.055601
C	4.649915	-4.279419	-4.001445		H	15.161985	1.139957	-0.158943
H	2.749751	-5.202221	-3.589222		H	14.726177	5.395062	0.225996
H	6.439379	-3.109123	-4.239767		H	10.5542	8.332674	0.336834
H	4.99814	-5.089134	-4.635511		H	16.175659	3.382324	0.089353
C	7.935216	1.338348	-0.540046		N	10.440275	0.860082	0.244763
C	10.016232	2.073978	-0.108358		N	8.257228	0.090683	-0.205514
C	9.515215	-0.098526	0.182077		N	8.77661	2.370878	-0.506596
N	9.871156	-1.379797	0.542601		Si	3.203227	-0.869213	-1.128597
C	11.019642	-1.753293	1.284537					

Supplementary Table 39. Atomic coordinates of SiCzCz:SiTrzCz2 (14, E = -4774.24798944 a.u.).

C	5.513112	0.708119	0.155891		C	3.060972	0.727177	0.59193
C	6.534789	-0.188119	0.455206		C	3.841822	-0.878647	-0.820363
C	7.859232	0.238056	0.573253		C	2.900468	1.735757	1.508316
C	8.129085	1.604094	0.4308		C	1.933402	-0.085357	0.141851
C	7.104328	2.5077	0.166584		C	4.616851	-1.662879	-1.622548
C	5.796909	2.06549	0.013303		C	2.425808	-1.067472	-0.70305
H	6.28001	-1.232703	0.597344		C	1.598345	2.000959	1.977143
H	9.147571	1.967272	0.531012		H	3.743276	2.30434	1.881198
H	7.327625	3.564916	0.059939		C	0.613966	0.252493	0.580639
H	4.996671	2.75272	-0.234322		C	3.965141	-2.739873	-2.334538
C	10.696581	-0.249927	1.614857		H	5.681472	-1.498901	-1.736906
C	12.001564	-0.497269	1.174282		C	1.803836	-2.145309	-1.418134
C	10.526095	0.588492	2.725544		C	0.465334	1.281695	1.470858
C	13.097288	0.063294	1.821754		H	1.44353	2.732369	2.758833
H	12.163904	-1.133387	0.308938		H	-0.240198	-0.269034	0.172576
C	11.617494	1.150473	3.377408		C	2.600869	-2.959932	-2.216535
H	9.524226	0.814215	3.082275		H	4.56696	-3.378271	-2.971582
C	12.906594	0.886661	2.925613		H	0.734785	-2.30195	-1.337989
H	14.101276	-0.140557	1.462101		H	2.143168	-3.773689	-2.769515
H	11.462689	1.797111	4.235783		N	-0.816359	1.652445	1.929254
H	13.760932	1.326065	3.431547		C	-1.284978	2.963865	2.077185
C	9.676667	-1.676969	-0.926045		C	-1.850476	0.77597	2.279048
C	10.329354	-2.907207	-1.081968		C	-0.659489	4.177336	1.790462
C	9.390252	-0.935606	-2.078493		C	-2.614472	2.924827	2.543638
C	10.692978	-3.374479	-2.339623		C	-1.870997	-0.617668	2.31253
H	10.54523	-3.516389	-0.208066		C	-2.975093	1.52955	2.671967
C	9.74827	-1.399668	-3.339659		C	-1.380002	5.344297	2.001229
H	8.875364	0.016736	-1.989242		H	0.35154	4.211295	1.406163
C	10.402236	-2.619623	-3.471423		C	-3.316902	4.1093	2.75407
H	11.197723	-4.330799	-2.437837		C	-3.042957	-1.237704	2.720877
H	9.514371	-0.809637	-4.220676		H	-1.001817	-1.201476	2.03956
H	10.68118	-2.984593	-4.455292		C	-4.138943	0.884101	3.083215
C	8.579235	-2.484218	1.79524		C	-2.692417	5.316995	2.485021
C	8.865861	-2.603575	3.159789		H	-0.917463	6.297942	1.768401
C	7.752049	-3.45409	1.211255		H	-4.346719	4.082093	3.095678
C	8.338728	-3.64398	3.917004		C	-4.172359	-0.501274	3.098383
H	9.515623	-1.87637	3.638062		H	-3.074835	-2.322571	2.749377
C	7.218362	-4.493225	1.964077		H	-5.007738	1.462609	3.378418
H	7.525732	-3.403745	0.149193		H	-3.231897	6.247452	2.624354
C	7.510953	-4.588485	3.320615		H	-5.07337	-1.019013	3.40803
H	8.575816	-3.718259	4.973991		Si	9.218474	-1.042402	0.779658
H	6.576094	-5.229983	1.491478		C	-3.608711	-1.414518	-1.141593
H	7.096999	-5.400331	3.910896		C	-4.912645	-1.532989	-0.658885
N	4.196134	0.228429	-0.017079		C	-5.538252	-2.775273	-0.547656

C	-4.809345	-3.911513	-0.924886		C	-4.990967	3.333041	0.011087
C	-3.501248	-3.80852	-1.383512		C	-2.283633	5.142606	-1.680703
C	-2.900382	-2.562475	-1.497235		C	-4.34964	5.495474	-0.451809
H	-5.44145	-0.628435	-0.388324		C	-5.754823	2.310235	0.563899
H	-5.273502	-4.892439	-0.867142		C	-5.363628	4.677029	0.179166
H	-2.9519	-4.701454	-1.664705		C	-2.163005	6.523574	-1.782518
H	-1.886462	-2.462758	-1.866879		H	-1.547493	4.491441	-2.120846
C	-8.214117	-4.242678	-0.871322		C	-4.213322	6.87446	-0.564877
C	-8.999266	-3.894103	-1.976548		C	-6.892556	2.660784	1.280788
C	-8.102924	-5.601979	-0.549632		H	-5.462307	1.28027	0.461695
C	-9.639732	-4.864098	-2.739117		C	-6.510143	5.010363	0.892224
H	-9.121127	-2.847757	-2.241236		C	-3.108172	7.387781	-1.226735
C	-8.739065	-6.576926	-1.309772		H	-1.307704	6.931905	-2.311616
H	-7.521739	-5.904251	0.317597		H	-4.962969	7.534693	-0.139575
C	-9.508086	-6.208391	-2.408238		C	-7.275823	3.993451	1.4441
H	-10.246043	-4.570161	-3.590586		H	-7.495233	1.868798	1.710917
H	-8.639417	-7.624342	-1.041261		H	-6.790786	6.051183	1.021426
H	-10.008969	-6.967224	-3.001786		H	-2.981727	8.461432	-1.321405
C	-8.174924	-1.298633	0.003542		H	-8.173152	4.232724	2.005859
C	-9.008347	-0.847477	1.034235		N	0.023943	1.287085	-2.478196
C	-8.082938	-0.510597	-1.152948		C	0.61729	0.356309	-3.359831
C	-9.735496	0.331828	0.908987		C	0.977152	2.283315	-2.175676
H	-9.092435	-1.425891	1.94938		C	0.066866	-0.746205	-4.007961
C	-8.797984	0.67457	-1.278837		C	1.942649	0.755985	-3.60202
H	-7.432566	-0.819336	-1.96722		C	0.883629	3.391171	-1.341081
C	-9.631436	1.094972	-0.248534		C	2.175834	1.966238	-2.838767
H	-10.379515	0.658521	1.720137		C	0.879237	-1.4478	-4.888661
H	-8.69928	1.273671	-2.178469		H	-0.95453	-1.046933	-3.826987
H	-10.187804	2.022251	-0.342991		C	2.745195	0.028141	-4.474534
C	-7.199385	-3.533524	1.902682		C	2.010494	4.190456	-1.200722
C	-8.341498	-3.998388	2.569421		H	-0.031341	3.622465	-0.819435
C	-5.993764	-3.507766	2.611084		C	3.295498	2.777483	-2.681356
C	-8.284607	-4.399485	3.899013		C	2.207307	-1.076721	-5.115829
H	-9.289081	-4.057791	2.03976		H	0.469904	-2.310843	-5.404398
C	-5.928987	-3.908781	3.941646		H	3.77359	0.32664	-4.650909
H	-5.091974	-3.168973	2.112164		C	3.204195	3.896818	-1.865889
C	-7.076907	-4.351146	4.588861		H	1.9541	5.064654	-0.559389
H	-9.181804	-4.754212	4.397145		H	4.22486	2.533221	-3.186334
H	-4.981844	-3.875274	4.47194		H	2.819185	-1.656455	-5.798931
H	-7.030572	-4.664216	5.627457		H	4.063608	4.54906	-1.744855
C	-2.989067	-0.080871	-1.296912		N	-1.84437	2.314438	-1.561466
C	-1.245353	1.189912	-1.939115		N	-3.682297	0.983808	-0.901081
C	-3.07314	2.160796	-1.061		N	-1.769588	-0.031387	-1.837786
N	-3.768014	3.295444	-0.711191		Si	-7.285958	-2.947137	0.123179
C	-3.379705	4.633599	-0.989617					

Supplementary Table 40. Atomic coordinates of SiCzCz:SiTrzCz2 (15, E = -4774.25820862 a.u.).

C	-3.559603	0.105822	1.931832		C	-1.333596	1.134501	1.603096
C	-4.603615	-0.351213	1.135115		C	-1.480381	-1.10048	1.21864
C	-5.91348	-0.402416	1.636286		C	-1.568486	2.466599	1.849919
C	-6.144408	0.054089	2.937464		C	0.018934	0.607483	1.429599
C	-5.104922	0.544001	3.722326		C	-1.921483	-2.370348	1.002119
C	-3.811255	0.557617	3.22833		C	-0.089945	-0.761878	1.217441
H	-4.383587	-0.668087	0.120455		C	-0.462853	3.326401	1.91743
H	-7.149298	0.023933	3.346641		H	-2.57431	2.846683	1.980396
H	-5.301441	0.893505	4.731008		C	1.124891	1.512241	1.472546
H	-2.984699	0.905391	3.838231		C	-0.924052	-3.398565	0.778008
C	-7.751482	0.314408	-0.676681		H	-2.974325	-2.616457	0.975757
C	-8.169728	0.050216	-1.985684		C	0.879228	-1.803032	1.034485
C	-7.697557	1.654667	-0.269532		C	0.871968	2.838726	1.70445
C	-8.523772	1.078747	-2.853036		H	-0.599649	4.368572	2.171785
H	-8.21357	-0.973558	-2.343033		H	2.132193	1.16182	1.289286
C	-8.06832	2.686917	-1.124051		C	0.426456	-3.105804	0.808466
H	-7.351208	1.89948	0.730927		H	-1.259621	-4.411716	0.586831
C	-8.480587	2.400423	-2.42097		H	1.93701	-1.578069	1.079319
H	-8.836495	0.84753	-3.866645		H	1.150928	-3.895656	0.635906
H	-8.022423	3.716083	-0.78112		N	1.924433	3.772365	1.771437
H	-8.760948	3.203772	-3.095123		C	3.122954	3.621799	2.479436
C	-8.855922	-1.228282	1.705756		C	1.903282	5.054369	1.212506
C	-9.009708	-2.409687	2.443763		C	3.586432	2.547599	3.235179
C	-9.825203	-0.230113	1.857963		C	3.863925	4.816921	2.379161
C	-10.083221	-2.584105	3.308327		C	0.951142	5.648406	0.385649
H	-8.282038	-3.209466	2.3356		C	3.086231	5.729913	1.569289
C	-10.902638	-0.399278	2.721214		C	4.811228	2.687343	3.871516
H	-9.743466	0.691635	1.2894		H	3.016785	1.631655	3.32526
C	-11.032319	-1.576625	3.448972		C	5.094431	4.930209	3.020562
H	-10.182524	-3.508015	3.869995		C	1.183083	6.948168	-0.035948
H	-11.644057	0.387625	2.821983		H	0.069883	5.109001	0.06271
H	-11.873999	-1.7114	4.121623		C	3.303691	7.031922	1.123324
C	-6.964206	-2.694607	-0.141755		C	5.566581	3.860405	3.764343
C	-7.859249	-3.321567	-1.020725		H	5.189387	1.863913	4.468277
C	-5.823156	-3.408924	0.23835		H	5.670564	5.847228	2.941415
C	-7.605347	-4.590399	-1.527345		C	2.343012	7.640984	0.331258
H	-8.783334	-2.823813	-1.30175		H	0.458066	7.426414	-0.685868
C	-5.566243	-4.68272	-0.256635		H	4.213177	7.559465	1.395259
H	-5.124218	-2.96697	0.939889		H	6.523487	3.932258	4.271106
C	-6.452902	-5.273071	-1.14839		H	2.495939	8.655873	-0.021305
H	-8.31275	-5.052744	-2.209032		Si	-7.355021	-1.013689	0.594396
H	-4.669837	-5.211419	0.052868		C	2.382814	-2.329103	-1.877133
H	-6.254192	-6.266007	-1.54011		C	3.603092	-2.384385	-1.213283
N	-2.217407	0.084324	1.464692		C	4.319004	-3.572231	-1.070239

C	3.776503	-4.727281	-1.643031		C	3.747744	2.465949	-1.032212
C	2.560916	-4.684401	-2.324215		C	1.027623	4.002504	-2.937492
C	1.855454	-3.49281	-2.434537		C	3.223365	4.488586	-1.997061
H	3.968204	-1.469605	-0.765079		C	4.476087	1.542584	-0.286553
H	4.298926	-5.674648	-1.546133		C	4.22643	3.771238	-1.234303
H	2.155536	-5.591817	-2.760946		C	1.012386	5.295484	-3.448108
H	0.900583	-3.453991	-2.946459		H	0.20132	3.328564	-3.104657
C	6.476498	-5.220171	0.368881		C	3.188099	5.776632	-2.518299
C	6.417898	-5.717933	1.675049		C	5.698277	1.946517	0.234912
C	6.960863	-6.069022	-0.637027		H	4.106872	0.543757	-0.114511
C	6.82376	-7.015744	1.968562		C	5.454543	4.156439	-0.705555
H	6.050785	-5.081435	2.474802		C	2.076629	6.176715	-3.244947
C	7.366857	-7.366607	-0.349773		H	0.148999	5.619694	-4.020679
H	7.030455	-5.708997	-1.660848		H	4.016393	6.457112	-2.350712
C	7.297723	-7.841926	0.956302		C	6.190442	3.236997	0.026
H	6.771278	-7.381492	2.989527		H	6.270108	1.23764	0.823339
H	7.739704	-8.00691	-1.14343		H	5.817094	5.169233	-0.850629
H	7.615569	-8.854921	1.183802		H	2.031504	7.180322	-3.655403
C	5.42655	-2.52672	1.500261		H	7.143529	3.524889	0.456424
C	6.246947	-1.510896	2.004322		N	-1.610315	0.197153	-2.078981
C	4.209914	-2.768287	2.152271		C	-2.470145	-0.895846	-2.371354
C	5.865737	-0.76056	3.112027		C	-2.409762	1.364465	-1.944622
H	7.191178	-1.292256	1.514434		C	-2.209122	-2.25858	-2.497433
C	3.822696	-2.023855	3.260778		C	-3.778848	-0.401634	-2.502877
H	3.539803	-3.537372	1.77679		C	-2.079055	2.658058	-1.547923
C	4.65185	-1.014933	3.740642		C	-3.741622	1.020722	-2.228789
H	6.512349	0.031786	3.475756		C	-3.279156	-3.099719	-2.77994
H	2.867376	-2.221105	3.737176		H	-1.214075	-2.644346	-2.352476
H	4.348672	-0.42718	4.602082		C	-4.834243	-1.259014	-2.798443
C	7.24513	-2.532756	-0.927621		C	-3.103295	3.595553	-1.476814
C	8.572164	-2.977176	-0.928924		H	-1.067886	2.921765	-1.286863
C	6.969764	-1.29092	-1.519283		C	-4.751733	1.973527	-2.163249
C	9.586613	-2.214275	-1.499602		C	-4.577886	-2.613515	-2.940177
H	8.820089	-3.932739	-0.476415		H	-3.094645	-4.165931	-2.867609
C	7.979388	-0.521068	-2.081566		H	-5.839673	-0.867425	-2.898952
H	5.955438	-0.904812	-1.542135		C	-4.424217	3.267508	-1.788056
C	9.291968	-0.983831	-2.074241		H	-2.859361	4.606285	-1.163997
H	10.608762	-2.580713	-1.490531		H	-5.775408	1.703262	-2.391666
H	7.739009	0.442197	-2.520621		H	-5.392392	-3.299529	-3.144259
H	10.083103	-0.384442	-2.514651		H	-5.201962	4.021634	-1.724303
C	1.664088	-1.038782	-1.904113		N	0.412928	1.311556	-1.895699
C	-0.236238	0.153402	-2.031016		N	2.397637	0.053128	-1.682357
C	1.728401	1.202488	-1.736358		N	0.351575	-1.043125	-2.114703
N	2.467513	2.363206	-1.624621		Si	5.892644	-3.490311	-0.046195
C	2.144283	3.610971	-2.205753					

Supplementary Table 41. Atomic coordinates of SiCzCz:SiTrzCz2 (16, E = -4774.26101980 a.u.).

C	-0.277323	-2.582502	-2.578061	C	2.11091	-1.9503	-2.301298
C	-1.348836	-2.440224	-1.702016	C	0.685868	-0.280937	-2.836124
C	-2.384051	-3.38299	-1.672243	C	2.664219	-3.167105	-1.907927
C	-2.30319	-4.469989	-2.550794	C	2.888068	-0.777436	-2.414419
C	-1.236415	-4.61022	-3.4318	C	-0.439475	0.473515	-3.158136
C	-0.221756	-3.665261	-3.452646	C	1.972731	0.293061	-2.761832
H	-1.348084	-1.587785	-1.027952	C	4.018532	-3.193575	-1.626798
H	-3.084219	-5.222613	-2.544334	H	2.054664	-4.058641	-1.816205
H	-1.197743	-5.458686	-4.107593	C	4.249379	-0.823806	-2.117631
H	0.615267	-3.75318	-4.137192	C	-0.256722	1.826792	-3.396586
C	-4.85737	-4.822313	-0.634668	H	-1.419722	0.016243	-3.234291
C	-5.817745	-4.953545	-1.647016	C	2.12857	1.657982	-3.005655
C	-4.632262	-5.925155	0.198347	C	4.801793	-2.034042	-1.722816
C	-6.52621	-6.137004	-1.820411	H	4.494878	-4.116266	-1.313673
H	-6.021932	-4.114672	-2.30652	H	4.867836	0.06554	-2.142385
C	-5.338221	-7.111435	0.030606	C	1.01266	2.416618	-3.320204
H	-3.899449	-5.853559	0.996372	H	-1.117528	2.436387	-3.648851
C	-6.287267	-7.219125	-0.97972	H	3.111438	2.111929	-2.951858
H	-7.267408	-6.213859	-2.610198	H	1.119889	3.480672	-3.498564
H	-5.148489	-7.952076	0.691165	N	6.174376	-2.10949	-1.36706
H	-6.841085	-8.14383	-1.11123	C	7.255461	-2.006281	-2.229763
C	-4.919634	-1.801156	-0.943418	C	6.650982	-2.309462	-0.077902
C	-6.277334	-1.801501	-0.59554	C	7.264226	-1.772108	-3.602174
C	-4.433676	-0.703282	-1.663398	C	8.44934	-2.164761	-1.492659
C	-7.114587	-0.751983	-0.954883	C	5.935225	-2.399617	1.113236
H	-6.69299	-2.641764	-0.046689	C	8.06053	-2.360685	-0.11061
C	-5.265851	0.349696	-2.026347	C	8.497508	-1.684129	-4.23
H	-3.389411	-0.670605	-1.958745	H	6.33738	-1.652582	-4.152122
C	-6.610532	0.327479	-1.672202	C	9.676281	-2.070594	-2.148464
H	-8.164334	-0.775407	-0.677188	C	6.662444	-2.548312	2.284124
H	-4.867717	1.187482	-2.590458	H	4.853898	-2.332668	1.125981
H	-7.260513	1.150184	-1.949393	C	8.766985	-2.515608	1.082534
C	-3.189424	-3.173322	1.290969	C	9.694574	-1.828676	-3.514031
C	-4.097628	-3.341069	2.34671	H	8.535603	-1.498192	-5.298917
C	-1.835138	-3.036134	1.614667	H	10.60461	-2.182794	-1.596029
C	-3.666706	-3.407289	3.664184	C	8.063643	-2.609544	2.274103
H	-5.159744	-3.436763	2.13778	H	6.129311	-2.609005	3.227604
C	-1.400144	-3.072321	2.935672	H	9.852439	-2.552912	1.078325
H	-1.098786	-2.911731	0.827971	H	10.64286	-1.752039	-4.035942
C	-2.313896	-3.276981	3.960794	H	8.601186	-2.726394	3.209531
H	-4.390163	-3.543559	4.461469	Si	-3.830018	-3.256562	-0.472216
H	-0.347914	-2.93406	3.162144	C	0.208955	3.417807	-0.032546
H	-1.974763	-3.310813	4.990591	C	1.272669	2.685688	0.491027
N	0.782669	-1.643246	-2.566357	C	2.585425	3.145387	0.418487

C	2.80091	4.395709	-0.181201		C	-1.98304	0.456631	3.367025
C	1.75134	5.125387	-0.727724		C	-5.343077	0.235055	1.999983
C	0.452831	4.636105	-0.662038		C	-4.093036	-0.111466	4.087626
H	1.048313	1.739016	0.962248		C	-0.623157	0.513475	3.431255
H	3.803372	4.813887	-0.214828		C	-2.774857	-0.041754	4.480032
H	1.943863	6.088053	-1.190967		C	-6.491035	-0.283919	2.667112
H	-0.38036	5.200849	-1.064614		H	-5.401052	0.524601	0.96332
C	5.39734	1.946193	-0.034651		C	-5.278971	-0.605983	4.742068
C	5.410892	2.546119	-1.299591		C	0.00885	0.109956	4.644636
C	6.492878	1.146825	0.319054		H	-0.031709	0.850627	2.597279
C	6.465998	2.346267	-2.184782		C	-2.106899	-0.419921	5.700634
H	4.576484	3.168056	-1.604393		C	-6.442884	-0.681393	4.021619
C	7.560697	0.964042	-0.550734		H	-7.417448	-0.365622	2.110836
H	6.511323	0.642803	1.281768		H	-5.232472	-0.920025	5.778876
C	7.544087	1.551594	-1.811184		C	-0.739685	-0.337465	5.754633
H	6.443717	2.80944	-3.166789		H	1.089641	0.155377	4.701248
H	8.394211	0.338957	-0.254659		H	-2.688537	-0.768171	6.546967
H	8.363839	1.375507	-2.50045		H	-7.345413	-1.058235	4.492953
C	3.372281	0.500199	1.787202		H	-0.216965	-0.623445	6.662242
C	3.720819	0.008594	3.051305		N	-4.402222	3.561923	-1.076284
C	2.58037	-0.316103	0.967343		C	-4.274788	4.096993	-2.380252
C	3.295286	-1.245573	3.480496		C	-5.778275	3.481208	-0.764824
H	4.3325	0.614366	3.712914		C	-3.141254	4.380644	-3.139641
C	2.142731	-1.565084	1.392872		C	-5.564269	4.308614	-2.900721
H	2.295469	0.026158	-0.020804		C	-6.414684	3.096436	0.411686
C	2.502667	-2.03392	2.652756		C	-6.514617	3.929058	-1.874513
H	3.580678	-1.605994	4.464564		C	-3.329076	4.864337	-4.428458
H	1.53212	-2.174287	0.734124		H	-2.15444	4.24165	-2.727272
H	2.170932	-3.0129	2.985792		C	-5.729762	4.799139	-4.193112
C	4.614088	3.131852	2.684965		C	-7.80231	3.12665	0.436219
C	5.949929	3.512113	2.845523		H	-5.851079	2.765297	1.269364
C	3.703087	3.489788	3.689348		C	-7.906747	3.951707	-1.831185
C	6.36536	4.218566	3.970645		C	-4.605998	5.07309	-4.956897
H	6.677985	3.253919	2.082537		H	-2.456602	5.091592	-5.033017
C	4.11185	4.1928	4.814796		H	-6.725827	4.960837	-4.593382
H	2.655007	3.216088	3.590379		C	-8.547951	3.538748	-0.672881
C	5.447702	4.558203	4.956991		H	-8.313775	2.818338	1.342561
H	7.407853	4.503778	4.075375		H	-8.476461	4.293774	-2.689676
H	3.389444	4.459013	5.580461		H	-4.716337	5.45516	-5.966384
H	5.770451	5.109218	5.835251		H	-9.631846	3.547022	-0.621599
C	-1.170078	2.921196	0.144812		N	-3.678918	2.146222	0.613037
C	-3.366965	3.037241	-0.319841		N	-1.361832	1.982806	1.068196
C	-2.637121	1.655367	1.29403		N	-2.136767	3.478903	-0.586872
N	-2.893987	0.784184	2.319967		Si	3.996517	2.17473	1.19224
C	-4.188766	0.344407	2.713584					

Supplementary Table 42. Atomic coordinates of GeCzCz:GeTrzCz2 (01, E = -4202.82082 a.u.).

C	-1.32432	-0.67568	0		C	-7.22418	0.672065	2.445306
C	-1.35736	-1.98616	-0.48049		H	-5.36028	-2.10123	2.93479
C	-0.44028	-2.93579	-0.03464		H	-9.57519	-1.75191	2.187015
C	0.516274	-2.55086	0.910293		H	-9.34397	0.701642	2.127899
C	0.563813	-1.2428	1.375458		H	-7.11801	1.748271	2.391201
C	-0.35523	-0.30227	0.927775		C	-9.45873	-4.63325	2.717677
H	-2.10415	-2.24591	-1.22192		C	-9.8944	-5.28743	3.871346
H	1.235385	-3.27271	1.28591		C	-10.391	-4.34672	1.71322
H	1.3042	-0.95379	2.113667		C	-11.2307	-5.64523	4.023418
H	-0.34558	0.708735	1.31416		H	-9.18412	-5.52157	4.659916
C	-1.08904	-6.08706	0.566443		C	-11.727	-4.70114	1.864598
C	-0.2037	-6.82478	1.355452		H	-10.0798	-3.84705	0.799114
C	-2.46095	-6.28972	0.74003		C	-12.1481	-5.35093	3.020698
C	-0.6755	-7.74391	2.288277		H	-11.556	-6.15418	4.925732
H	0.867753	-6.68985	1.239402		H	-12.4383	-4.46958	1.07737
C	-2.93758	-7.21024	1.66602		H	-13.191	-5.62879	3.139518
H	-3.17806	-5.73236	0.147495		C	-6.39785	-4.80387	3.884322
C	-2.04379	-7.93802	2.444876		C	-5.86356	-6.08741	3.736905
H	0.02728	-8.31172	2.891021		C	-6.01652	-4.04741	4.995635
H	-4.00727	-7.34994	1.77969		C	-4.97308	-6.60207	4.672686
H	-2.41201	-8.65804	3.169733		H	-6.13793	-6.6895	2.874239
C	1.423468	-5.23985	-1.21263		C	-5.11591	-4.55358	5.927449
C	1.762597	-6.57333	-1.46347		H	-6.40671	-3.04242	5.127323
C	2.408181	-4.26263	-1.37374		C	-4.59262	-5.83121	5.766294
C	3.050595	-6.92255	-1.85294		H	-4.56468	-7.59969	4.542439
H	1.015289	-7.35434	-1.34862		H	-4.81403	-3.94468	6.77374
C	3.698663	-4.60738	-1.76434		H	-3.88187	-6.2226	6.487431
H	2.172133	-3.21889	-1.1886		C	-6.93682	-4.75388	0.767503
C	4.021975	-5.93802	-2.00269		C	-7.65809	-5.68349	0.016758
H	3.29655	-7.96339	-2.04072		C	-5.72311	-4.28742	0.256176
H	4.452308	-3.83456	-1.88245		C	-7.18737	-6.12105	-1.21884
H	5.028685	-6.20822	-2.3068		H	-8.60434	-6.0637	0.390105
C	-1.54722	-4.88099	-2.36059		C	-5.26347	-4.69286	-0.99174
C	-2.29292	-6.02958	-2.64033		H	-5.1297	-3.58652	0.835878
C	-1.54954	-3.84594	-3.30178		C	-5.99841	-5.61509	-1.73157
C	-3.03132	-6.13506	-3.81525		H	-7.75885	-6.84917	-1.78735
H	-2.30658	-6.85305	-1.9328		H	-4.33624	-4.29487	-1.39269
C	-2.29758	-3.94059	-4.47039		H	-5.63438	-5.93568	-2.70179
H	-0.96255	-2.94902	-3.12588		C	-4.7507	0.469829	2.767038
C	-3.04284	-5.08617	-4.72797		C	-3.31087	2.168091	2.406585
H	-3.59852	-7.03917	-4.01673		C	-2.59903	0.274527	3.3927
H	-2.29501	-3.12071	-5.18192		N	-1.59061	-0.4722	3.971181
H	-3.62482	-5.16364	-5.6412		C	-0.39853	0.034537	4.530617
N	-2.27269	0.267571	-0.46349		C	-1.52046	-1.88736	3.983554

C	-3.62993	0.034911	-0.59377
C	-1.98172	1.559054	-0.95941
C	-4.38453	-1.04742	-0.23016
C	-4.23246	1.227978	-1.18601
C	-0.78709	2.202307	-1.07892
C	-3.21808	2.150929	-1.392
C	-5.77641	-0.98541	-0.44612
H	-3.93903	-1.9106	0.2463
C	-5.64725	1.241004	-1.44322
C	-0.7957	3.53232	-1.63788
H	0.145025	1.748547	-0.76832
C	-3.19898	3.478762	-1.93517
C	-6.38622	0.154439	-1.05716
H	-6.41061	-1.77611	-0.07926
H	-6.10306	2.083476	-1.94651
C	-1.97858	4.135367	-2.04408
H	0.142158	4.07042	-1.70831
H	-4.12507	3.96119	-2.22532
H	-1.95283	5.146908	-2.43648
N	-7.7942	0.133782	-1.18959
C	-8.66233	1.144	-0.76074
C	-8.55857	-1.00528	-1.4663
C	-8.38	2.434297	-0.31824
C	-9.98185	0.647653	-0.75965
C	-8.15931	-2.24916	-1.95447
C	-9.91609	-0.72438	-1.21274
C	-9.43779	3.21341	0.12833
H	-7.36789	2.81333	-0.30763
C	-11.0302	1.451184	-0.31683
C	-9.13786	-3.21319	-2.14675
H	-7.12169	-2.46732	-2.17354
C	-10.8829	-1.70767	-1.41299
C	-10.7536	2.734419	0.129499
H	-9.24021	4.21894	0.487503
H	-12.0472	1.070626	-0.31018
C	-10.4874	-2.95414	-1.87445
H	-8.84191	-4.18975	-2.51206
H	-11.9295	-1.49592	-1.21564
H	-11.558	3.372028	0.480885
H	-11.2269	-3.73171	-2.03764
C	-6.10014	-0.12053	2.666023
C	-6.24468	-1.50685	2.74168
C	-7.48015	-2.11885	2.551025
C	-8.59664	-1.30489	2.333009
C	-8.4714	0.0802	2.297096
C	-0.02601	1.343477	4.821513
C	0.429112	-1.04899	4.875297
C	-2.41976	-2.84332	3.521167
C	-0.27973	-2.26379	4.524734
C	1.217592	1.550326	5.406051
H	-0.68106	2.173297	4.604598
C	1.671818	-0.82105	5.459835
C	-2.06164	-4.17945	3.631205
H	-3.36951	-2.55434	3.103416
C	0.064946	-3.60933	4.622178
C	2.067463	0.484323	5.713091
H	1.526942	2.565956	5.631379
H	2.314643	-1.65589	5.721481
C	-0.83397	-4.56558	4.177088
H	-2.75531	-4.93804	3.290061
H	1.02453	-3.89974	5.039423
H	3.034174	0.678873	6.165889
H	-0.5891	-5.61939	4.241554
N	-3.03887	3.411685	1.875104
C	-3.98793	4.296465	1.29477
C	-1.75975	4.02644	1.789898
C	-5.35604	4.157172	1.078335
C	-3.31717	5.462248	0.890722
C	-0.49294	3.564405	2.138456
C	-1.91516	5.292952	1.203559
C	-6.02508	5.203056	0.4527
H	-5.8698	3.258024	1.371779
C	-4.00299	6.498954	0.267657
C	0.595661	4.395812	1.904246
H	-0.35321	2.591977	2.575766
C	-0.81569	6.11464	0.980106
C	-5.36489	6.364778	0.04772
H	-7.09094	5.107355	0.273656
H	-3.47384	7.393111	-0.04687
C	0.445215	5.662166	1.336174
H	1.585359	4.04105	2.174599
H	-0.94792	7.090978	0.52428
H	-5.91911	7.160249	-0.43959
H	1.315546	6.288317	1.16879
N	-2.29861	1.500655	2.964689
N	-3.79617	-0.29498	3.300037
N	-4.5622	1.709975	2.316122
Ge	-7.59308	-4.08271	2.502142
Ge	-0.43028	-4.77424	-0.74103

Supplementary Table 43. Atomic coordinates of GeCzCz:GeTrzCz2 (02, E = -4202.8125823 a.u.).

C	3.115426	-2.963732	1.079912	C	0.817193	-2.298399	0.497162
C	4.122586	-2.258893	0.429995	C	1.889721	-1.0569	2.065437
C	5.334544	-2.862488	0.111659	C	0.471278	-3.218832	-0.451019
C	5.518554	-4.205017	0.453774	C	0.054772	-1.07859	0.711174
C	4.50797	-4.916684	1.093944	C	2.801134	-0.55854	2.944388
C	3.301446	-4.298357	1.415183	C	0.706678	-0.332209	1.678534
H	3.935516	-1.218317	0.186246	C	-0.691437	-2.96865	-1.223934
H	6.458071	-4.699378	0.222298	H	1.065124	-4.109388	-0.619216
H	4.662585	-5.958878	1.355496	C	-1.127219	-0.863741	-0.07257
H	2.507672	-4.833081	1.926654	C	2.542949	0.744202	3.507424
C	6.155831	-1.73723	-2.75558	H	3.695722	-1.110202	3.208702
C	7.089135	-1.375621	-3.73095	C	0.459059	0.950716	2.289035
C	4.844755	-2.01372	-3.154086	C	-1.479642	-1.804971	-1.006957
C	6.71922	-1.277953	-5.068778	H	-0.974527	-3.650967	-2.015262
H	8.119317	-1.17377	-3.448087	H	-1.723054	0.022317	0.080062
C	4.47087	-1.915193	-4.490246	C	1.388745	1.455219	3.182072
H	4.104773	-2.314359	-2.416926	H	3.255038	1.160703	4.211361
C	5.408106	-1.544091	-5.448679	H	-0.450351	1.491615	2.071012
H	7.455265	-0.994965	-5.815217	H	1.210198	2.421491	3.639991
H	3.448004	-2.131678	-4.783334	N	-2.638742	-1.618702	-1.79903
H	5.117724	-1.466491	-6.491943	C	-3.650617	-2.55887	-2.002069
C	8.46161	-2.483472	-0.647963	C	-2.99522	-0.441632	-2.459559
C	9.334636	-1.95305	0.304801	C	-3.770342	-3.858987	-1.512358
C	8.894597	-3.557227	-1.431688	C	-4.658337	-1.982608	-2.804468
C	10.607406	-2.48683	0.477235	C	-2.300955	0.761566	-2.55597
H	9.021109	-1.109997	0.914826	C	-4.2373	-0.629261	-3.09808
C	10.166194	-4.094359	-1.260703	C	-4.9131	-4.572433	-1.843678
H	8.239032	-3.973284	-2.192637	H	-2.999498	-4.294271	-0.889005
C	11.023097	-3.559528	-0.304444	C	-5.794363	-2.721891	-3.129074
H	11.276753	-2.063385	1.219945	C	-2.882612	1.784271	-3.288685
H	10.490165	-4.927837	-1.876616	H	-1.343335	0.8942	-2.071477
H	12.0168	-3.976339	-0.171361	C	-4.800174	0.412745	-3.833719
C	6.486685	0.047607	-0.122971	C	-5.915705	-4.015677	-2.646518
C	6.557256	1.164733	-0.958851	H	-5.036088	-5.580135	-1.459979
C	6.239309	0.248719	1.239093	H	-6.582553	-2.279635	-3.730212
C	6.389344	2.448361	-0.449532	C	-4.122502	1.618926	-3.919343
H	6.724513	1.033059	-2.024262	H	-2.373435	2.74055	-3.357151
C	6.064827	1.530215	1.751921	H	-5.760269	0.279492	-4.321359
H	6.155134	-0.604377	1.906951	H	-6.80305	-4.596331	-2.874013
C	6.140267	2.632132	0.906089	H	-4.553111	2.443171	-4.478793
H	6.434617	3.30483	-1.11487	Ge	6.650012	-1.771206	-0.855858
H	5.855898	1.667333	2.808444	C	-3.757883	2.578452	0.106068
H	5.993161	3.632448	1.301603	C	-2.418879	2.944819	0.235309
N	1.902863	-2.281232	1.369357	C	-1.918126	4.108937	-0.341672

C	-2.801206	4.921823	-1.055682		C	-2.063673	-0.458813	3.824783
C	-4.14213	4.565383	-1.19325		C	-2.74357	-3.470114	1.875955
C	-4.621644	3.393518	-0.623442		C	-1.276662	-2.60996	3.625363
H	-1.761683	2.277412	0.777575		C	-2.19829	0.827915	4.337633
H	-2.441893	5.839757	-1.513013		C	-1.116297	-1.359152	4.341169
H	-4.815611	5.204241	-1.755939		C	-2.036816	-4.664168	1.933988
H	-5.6596	3.102595	-0.73769		H	-3.562594	-3.337658	1.18539
C	0.430533	6.168373	-1.083256		C	-0.579884	-3.814712	3.664805
C	1.082526	6.114766	-2.317335		C	-1.351953	1.197954	5.373273
C	0.070787	7.417835	-0.569162		H	-2.923149	1.518955	3.930019
C	1.360726	7.279318	-3.025953		C	-0.267912	-0.962432	5.37058
H	1.383976	5.154778	-2.727558		C	-0.964732	-4.840395	2.814202
C	0.346124	8.583998	-1.274924		H	-2.328089	-5.477059	1.27586
H	-0.422295	7.485081	0.39751		H	0.257406	-3.940318	4.344105
C	0.990645	8.51477	-2.505987		C	-0.390574	0.319527	5.882758
H	1.869509	7.222319	-3.98357		H	-1.432512	2.197741	5.787048
H	0.061312	9.547551	-0.863108		H	0.475748	-1.648182	5.763577
H	1.207844	9.424418	-3.057728		H	-0.433612	-5.786788	2.832341
C	1.076964	3.027848	-0.747707		H	0.26489	0.646351	6.683177
C	2.161998	2.545998	-0.01298		N	-6.544069	-1.253581	0.06829
C	0.772306	2.410716	-1.965082		C	-7.414604	-0.793898	-0.956931
C	2.920683	1.475548	-0.476074		C	-6.974716	-2.550359	0.463552
H	2.416071	2.99195	0.942324		C	-7.404281	0.378865	-1.706681
C	1.523623	1.335754	-2.429808		C	-8.380541	-1.786121	-1.192632
H	-0.065347	2.765296	-2.560476		C	-6.474298	-3.430544	1.419764
C	2.600708	0.867075	-1.683874		C	-8.102806	-2.891261	-0.299914
H	3.758258	1.121004	0.113642		C	-8.378388	0.531669	-2.686991
H	1.264814	0.862522	-3.372164		H	-6.648789	1.12963	-1.549358
H	3.193369	0.034809	-2.050935		C	-9.349432	-1.614512	-2.174906
C	0.325329	4.818522	1.81854		C	-7.107521	-4.659138	1.566996
C	1.571402	5.288307	2.246379		H	-5.621484	-3.173852	2.025081
C	-0.647072	4.543741	2.782813		C	-8.724993	-4.123781	-0.137129
C	1.846142	5.458316	3.598953		C	-9.345163	-0.447331	-2.92435
H	2.34079	5.528291	1.516645		H	-8.378215	1.439704	-3.282049
C	-0.379933	4.721121	4.137085		H	-10.089853	-2.38789	-2.354297
H	-1.626475	4.185475	2.480245		C	-8.217313	-5.013016	0.797689
C	0.869202	5.173079	4.547916		H	-6.721333	-5.356228	2.303914
H	2.821101	5.818657	3.913118		H	-9.592506	-4.381894	-0.736714
H	-1.150449	4.507768	4.871612		H	-10.091869	-0.294797	-3.696883
H	1.079891	5.308242	5.604438		H	-8.685818	-5.981931	0.936686
C	-4.184426	1.280023	0.668586		N	-4.669309	-1.225483	1.433005
C	-5.452437	-0.580005	0.561746		N	-3.411071	0.76323	1.625971
C	-3.677544	-0.504331	1.939433		N	-5.240449	0.669338	0.136982
N	-2.820864	-1.120406	2.836912		Ge	-0.010087	4.529261	-0.097846
C	-2.348146	-2.447372	2.729601					

Supplementary Table 44. Atomic coordinates of GeCzCz:GeTrzCz2 (03, E = -4202.8109488 a.u.).

C	-5.320549	0.225177	1.923947		C	-3.006249	-0.551621	2.364123
C	-6.267104	-0.174665	0.984989		C	-4.680521	-2.034451	2.798738
C	-7.189341	0.730818	0.466671		C	-2.236396	0.515697	1.969817
C	-7.148697	2.057309	0.901567		C	-2.410245	-1.823354	2.781551
C	-6.205587	2.459061	1.843076		C	-5.894417	-2.632031	2.974726
C	-5.295081	1.547615	2.36239		C	-3.446654	-2.713548	3.041364
H	-6.245104	-1.200672	0.635445		C	-0.842134	0.353824	1.962934
H	-7.843147	2.786116	0.493511		H	-2.678814	1.436819	1.61261
H	-6.178156	3.491281	2.177825		C	-0.988018	-1.938437	2.820603
H	-4.564391	1.851319	3.103628		C	-5.903713	-4.012468	3.403652
C	-8.923629	1.615386	-2.06758		H	-6.82565	-2.103947	2.810594
C	-10.259861	1.902268	-2.354497		C	-3.481068	-4.08088	3.460981
C	-7.930402	2.427206	-2.626496		C	-0.234141	-0.881738	2.385889
C	-10.59691	2.96983	-3.181023		H	-0.215986	1.13622	1.561531
H	-11.047843	1.288593	-1.926681		H	-0.52093	-2.844492	3.18261
C	-8.263396	3.493009	-3.454926		C	-4.723528	-4.698049	3.630541
H	-6.881923	2.232142	-2.419109		H	-6.858551	-4.506723	3.543662
C	-9.59915	3.765642	-3.732384		H	-2.558152	-4.621495	3.637526
H	-11.64055	3.180987	-3.393551		H	-4.76194	-5.735847	3.946918
H	-7.479821	4.11117	-3.88256		N	1.163449	-0.998967	2.277739
H	-9.861576	4.598709	-4.377289		C	2.10265	-0.000876	2.577817
C	-10.037555	-0.643628	-0.041627		C	1.857437	-2.120057	1.795485
C	-10.892986	-1.502389	-0.738581		C	1.920746	1.265786	3.130917
C	-10.340351	-0.327253	1.284858		C	3.391827	-0.480075	2.27292
C	-12.028485	-2.024091	-0.128189		C	1.377774	-3.338614	1.315627
H	-10.667426	-1.777384	-1.765936		C	3.23672	-1.835068	1.790526
C	-11.474195	-0.848855	1.89934		C	3.045505	2.060825	3.309848
H	-9.681601	0.329216	1.847217		H	0.94023	1.627295	3.412695
C	-12.31994	-1.696357	1.192083		C	4.50505	0.335388	2.452314
H	-12.683957	-2.690058	-0.681156		C	2.303705	-4.267699	0.861772
H	-11.69628	-0.595894	2.931628		H	0.318268	-3.551417	1.279089
H	-13.204564	-2.10519	1.670782		C	4.149962	-2.78881	1.347598
C	-7.540508	-1.314504	-1.918235		C	4.32407	1.612407	2.960185
C	-6.8704	-1.043351	-3.11418		H	2.920387	3.05761	3.719054
C	-7.484731	-2.613487	-1.401885		H	5.494354	-0.023352	2.1968
C	-6.152116	-2.037772	-3.770177		C	3.678633	-4.006797	0.886798
H	-6.907509	-0.046035	-3.543364		H	1.947251	-5.220787	0.482995
C	-6.767918	-3.610882	-2.055764		H	5.210667	-2.565579	1.349358
H	-8.009044	-2.855673	-0.480871		H	5.17913	2.268316	3.085099
C	-6.097528	-3.321972	-3.239756		H	4.376411	-4.755769	0.527534
H	-5.629774	-1.808147	-4.693564		Ge	-8.449961	0.106635	-0.909105
H	-6.732001	-4.612922	-1.639108		C	3.738919	-1.007061	-1.539966
H	-5.529515	-4.095722	-3.746485		C	5.059713	-0.729368	-1.191695
N	-4.376435	-0.711223	2.399867		C	6.09335	-1.606619	-1.504978

C	5.776061	-2.800345	-2.156818		C	3.976573	3.652226	0.213687
C	4.455229	-3.097631	-2.487078		C	0.46767	4.10212	1.174685
C	3.435528	-2.203821	-2.186384		C	2.688332	5.031629	1.53055
H	5.257617	0.19173	-0.655415		C	5.1242	3.178729	-0.418435
H	6.562086	-3.506293	-2.41069		C	4.039066	4.74982	1.086902
H	4.220889	-4.031916	-2.987582		C	-0.022094	5.051764	2.063798
H	2.406113	-2.418616	-2.449134		H	-0.194264	3.393905	0.703692
C	9.229613	-2.39151	-1.531244		C	2.177706	5.977338	2.413904
C	10.102466	-2.960155	-0.601384		C	6.334064	3.793461	-0.121664
C	9.322724	-2.793895	-2.867925		H	5.08302	2.35782	-1.114831
C	11.037811	-3.913461	-0.992984		C	5.260648	5.350081	1.379331
H	10.056705	-2.656804	0.44047		C	0.816231	5.983185	2.679964
C	10.253799	-3.747673	-3.262511		H	-1.086381	5.063771	2.276729
H	8.664548	-2.356645	-3.614954		H	2.839354	6.694019	2.890662
C	11.112312	-4.310134	-2.323042		C	6.410479	4.860196	0.778396
H	11.709751	-4.345933	-0.257779		H	7.232689	3.435991	-0.614371
H	10.312532	-4.05003	-4.303683		H	5.306712	6.192207	2.063075
H	11.841127	-5.054377	-2.629245		H	0.401107	6.712504	3.367866
C	7.957539	-0.707913	0.923314		H	7.371334	5.316431	0.993999
C	8.074399	0.58831	1.431324		N	-0.787394	0.391857	-1.253468
C	7.814548	-1.765409	1.828812		C	-1.397202	-0.887998	-1.189753
C	8.058704	0.822438	2.803458		C	-1.811843	1.364813	-1.315213
H	8.167647	1.430698	0.753731		C	-0.832593	-2.150756	-1.046499
C	7.794748	-1.535935	3.200585		C	-2.791391	-0.715884	-1.212825
H	7.71346	-2.784856	1.463521		C	-1.740196	2.752303	-1.412712
C	7.918807	-0.239197	3.689794		C	-3.052673	0.705485	-1.310631
H	8.144157	1.838206	3.177434		C	-1.697713	-3.23115	-0.915789
H	7.675224	-2.367994	3.887463		H	0.237635	-2.283567	-1.02182
H	7.895127	-0.057155	4.75955		C	-3.64099	-1.811309	-1.094795
C	8.272041	0.666883	-1.932017		C	-2.934209	3.463242	-1.456837
C	9.468783	1.348688	-1.68775		H	-0.790575	3.264008	-1.442004
C	7.350535	1.237422	-2.812923		C	-4.236055	1.4335	-1.367094
C	9.730438	2.572947	-2.29334		C	-3.085456	-3.071821	-0.939234
H	10.206986	0.925485	-1.010829		H	-1.275122	-4.223931	-0.793234
C	7.608543	2.461641	-3.42327		H	-4.716137	-1.678744	-1.119112
H	6.415259	0.727522	-3.023047		C	-4.172796	2.817415	-1.424426
C	8.797356	3.132748	-3.161143		H	-2.894461	4.546288	-1.516678
H	10.662772	3.090577	-2.088801		H	-5.187801	0.918034	-1.348466
H	6.877078	2.893465	-4.099357		H	-3.734186	-3.934723	-0.833268
H	8.997536	4.090373	-3.631797		H	-5.088207	3.400174	-1.439099
C	2.701767	-0.009341	-1.212695		N	0.892511	1.851002	-0.605398
C	0.556956	0.648104	-1.078297		N	3.138896	1.166753	-0.769574
C	2.200476	2.04862	-0.445791		N	1.417127	-0.325491	-1.381404
N	2.622427	3.24029	0.107572		Ge	7.908057	-1.037583	-1.01577
C	1.835336	4.09989	0.913167					

Supplementary Table 45. Atomic coordinates of GeCzCz:GeTrzCz2 (04, E = -4202.8027052 a.u.).

C	-0.849969	-2.326988	2.404457		C	-3.207242	-2.039934	1.642771
C	0.377219	-1.694464	2.224373		C	-2.240249	-0.260231	2.691492
C	1.570601	-2.342106	2.536715		C	-3.51751	-3.247105	1.063521
C	1.523448	-3.677374	2.941865		C	-4.177289	-0.94728	1.701353
C	0.302769	-4.334292	3.055293		C	-1.403039	0.581046	3.366324
C	-0.886343	-3.657247	2.821532		C	-3.582812	0.116909	2.360451
H	0.385429	-0.669954	1.874316		C	-4.793013	-3.397314	0.490593
H	2.437484	-4.202404	3.203913		H	-2.791786	-4.045327	0.986704
H	0.271257	-5.371751	3.372792		C	-5.466852	-1.12819	1.107673
H	-1.838282	-4.143767	2.995256		C	-1.907324	1.890973	3.725306
C	4.429972	-1.374669	1.135335		H	-0.394078	0.311401	3.64582
C	5.04419	-0.227577	0.627275		C	-4.065091	1.416165	2.739878
C	4.834194	-2.622956	0.649963		C	-5.740181	-2.324164	0.503894
C	6.054997	-0.327439	-0.324402		H	-5.050449	-4.308939	-0.033155
H	4.739779	0.7528	0.983221		H	-6.205574	-0.336457	1.130832
C	5.845515	-2.72661	-0.297761		C	-3.20282	2.27388	3.418349
H	4.370627	-3.530369	1.030623		H	-1.236679	2.574976	4.230487
C	6.466614	-1.576086	-0.7744		H	-5.078366	1.713241	2.495951
H	6.522519	0.569712	-0.717847		H	-3.553454	3.260071	3.705858
H	6.147432	-3.700759	-0.66883		N	-6.956941	-2.500843	-0.190715
H	7.257733	-1.652038	-1.512368		C	-7.826403	-3.58216	-0.065056
C	4.205571	-1.944406	4.229717		C	-7.445932	-1.655534	-1.187639
C	5.600801	-2.010216	4.212796		C	-7.771976	-4.660235	0.815876
C	3.532979	-2.337879	5.391062		C	-8.886119	-3.429643	-0.982693
C	6.307087	-2.453268	5.326521		C	-6.898078	-0.480936	-1.699545
H	6.144292	-1.717952	3.3187		C	-8.640417	-2.19753	-1.703723
C	4.235404	-2.779255	6.507396		C	-8.788441	-5.601304	0.74342
H	2.447041	-2.309455	5.425086		H	-6.967092	-4.754761	1.535493
C	5.624826	-2.837238	6.47558		C	-9.897058	-4.387382	-1.033605
H	7.391533	-2.500221	5.296427		C	-7.576034	0.151897	-2.7307
H	3.698051	-3.081546	7.401292		H	-5.964676	-0.090584	-1.3136
H	6.174905	-3.183768	7.345281		C	-9.307979	-1.537981	-2.734779
C	2.630749	0.573925	3.004389		C	-9.840493	-5.47259	-0.172563
C	2.104586	1.371006	1.981967		H	-8.768991	-6.45249	1.416749
C	2.637054	1.079814	4.307104		H	-10.718755	-4.281717	-1.735503
C	1.598496	2.63656	2.255615		C	-8.774344	-0.363444	-3.242072
H	2.071999	1.003814	0.960394		H	-7.165399	1.065115	-3.151246
C	2.124858	2.344234	4.583786		H	-10.229651	-1.944962	-3.139574
H	3.045028	0.481395	5.116778		H	-10.620253	-6.226505	-0.201817
C	1.604876	3.125122	3.557685		H	-9.28321	0.158795	-4.045484
H	1.2016	3.245485	1.453783		Ge	3.217335	-1.268074	2.669122
H	2.135776	2.720745	5.602468		C	2.286168	2.25504	-1.605159
H	1.205847	4.111987	3.764308		C	1.104274	2.913222	-1.259625
N	-2.040498	-1.5862	2.227714		C	1.09236	4.284867	-1.014309

C	2.293436	4.992435	-1.12783		C	-1.103357	-1.689817	-1.740721
C	3.477975	4.343355	-1.459333		C	1.332343	-4.134464	-0.565493
C	3.477017	2.977012	-1.696973		C	-0.976214	-3.952494	-1.332741
H	0.194038	2.329517	-1.180876		C	-1.66214	-0.473135	-2.112302
H	2.307874	6.066813	-0.96396		C	-1.842898	-2.884441	-1.795669
H	4.404118	4.90475	-1.530986		C	1.108338	-5.486258	-0.340962
H	4.392719	2.452972	-1.943414		H	2.289806	-3.680481	-0.348296
C	-0.475524	5.651513	1.463872		C	-1.183443	-5.309723	-1.097137
C	-1.499432	5.263045	2.329403		C	-2.974163	-0.483908	-2.572389
C	0.585712	6.402404	1.980048		H	-1.098387	0.446082	-2.045157
C	-1.467786	5.619155	3.67496		C	-3.153222	-2.871622	-2.262246
H	-2.329086	4.672583	1.954445		C	-0.134162	-6.07242	-0.604174
C	0.619177	6.764497	3.321923		H	1.914014	-6.094121	0.058466
H	1.398908	6.715567	1.330487		H	-2.15186	-5.759924	-1.293705
C	-0.411366	6.373189	4.172384		C	-3.71386	-1.666437	-2.656154
H	-2.271995	5.310059	4.336264		H	-3.429189	0.455102	-2.867641
H	1.449233	7.350166	3.705109		H	-3.724975	-3.79171	-2.319868
H	-0.388912	6.65536	5.220595		H	-0.278929	-7.131167	-0.415312
C	-2.143682	4.154619	-0.84359		H	-4.734385	-1.637862	-3.021154
C	-3.10173	4.651887	-1.731993		N	4.409378	-1.629223	-3.256896
C	-2.370448	2.913712	-0.237263		C	5.519993	-0.941346	-3.812354
C	-4.26227	3.935015	-2.002886		C	4.656416	-3.024143	-3.364566
H	-2.943928	5.612662	-2.213499		C	5.757403	0.419525	-3.991806
C	-3.525432	2.189545	-0.51606		C	6.464452	-1.89332	-4.23258
H	-1.656484	2.502053	0.472304		C	3.880561	-4.119364	-2.994377
C	-4.475003	2.704316	-1.392525		C	5.923906	-3.205141	-3.941717
H	-5.001273	4.339315	-2.688069		C	6.963076	0.799214	-4.570076
H	-3.677123	1.223499	-0.0502		H	5.026677	1.152544	-3.69372
H	-5.385799	2.147951	-1.593811		C	7.666898	-1.494072	-4.806936
C	-0.613351	6.918397	-1.445117		C	4.4145	-5.387505	-3.18716
C	-0.201902	6.980966	-2.779811		H	2.898093	-3.992534	-2.573804
C	-1.099392	8.080271	-0.840379		C	6.442112	-4.482939	-4.127385
C	-0.27958	8.171609	-3.494192		C	7.916061	-0.139827	-4.969496
H	0.191349	6.093021	-3.268235		H	7.161009	1.856599	-4.71479
C	-1.177843	9.273474	-1.551166		H	8.393507	-2.235044	-5.125441
H	-1.413903	8.057181	0.199428		C	5.682811	-5.576892	-3.740608
C	-0.768872	9.319508	-2.879468		H	3.818768	-6.24791	-2.898676
H	0.044158	8.204543	-4.530102		H	7.424991	-4.616266	-4.568614
H	-1.556549	10.168631	-1.067133		H	8.848207	0.19134	-5.415301
H	-0.828682	10.250399	-3.435166		H	6.070174	-6.581476	-3.875744
C	2.286862	0.797736	-1.856029		N	2.363322	-1.843352	-2.184811
C	3.348233	-1.048391	-2.600467		N	1.222919	0.095021	-1.451469
C	1.317026	-1.218252	-1.654548		N	3.355621	0.278552	-2.452403
N	0.217472	-1.999056	-1.327131		Ge	-0.548614	5.223946	-0.457784
C	0.280199	-3.383161	-1.073932					

Supplementary Table 46. Atomic coordinates of GeCzCz:GeTrzCz2 (05, E = -4202.7988948 a.u.).

C	4.578472	-1.19948	-0.207597	C	2.458732	-0.029677	-0.774978
C	5.927831	-0.916307	-0.395279	C	3.809158	0.981233	0.755445
C	6.874262	-1.939269	-0.433375	C	1.914386	-0.812712	-1.76208
C	6.440453	-3.261744	-0.329763	C	1.80895	1.201585	-0.320715
C	5.085345	-3.548836	-0.183407	C	4.814543	1.284903	1.627133
C	4.152623	-2.523778	-0.105837	C	2.657902	1.814602	0.593012
H	6.229469	0.120131	-0.506726	C	0.666447	-0.432885	-2.286755
H	7.160359	-4.074206	-0.361795	H	2.430359	-1.684626	-2.144788
H	4.754899	-4.580176	-0.105307	C	0.500735	1.514529	-0.807801
H	3.100811	-2.730915	0.052618	C	4.704965	2.518931	2.36888
C	9.855024	-3.014247	-0.949847	H	5.672174	0.638944	1.770956
C	10.986968	-3.35014	-0.203814	C	2.57573	3.032589	1.335991
C	9.51628	-3.812189	-2.047808	C	-0.053286	0.695491	-1.754046
C	11.765303	-4.450701	-0.547879	H	0.248972	-0.96255	-3.131833
H	11.26323	-2.748691	0.657952	H	-0.052007	2.341631	-0.383603
C	10.292233	-4.913028	-2.39501	C	3.613735	3.355738	2.209982
H	8.632666	-3.579909	-2.637466	H	5.500066	2.781519	3.0578
C	11.419098	-5.232325	-1.644555	H	1.721126	3.682642	1.210704
H	12.642084	-4.69967	0.042216	H	3.556632	4.275084	2.784104
H	10.016358	-5.523049	-3.249833	N	-1.349349	0.932748	-2.251526
H	12.025476	-6.091909	-1.913329	C	-2.312486	-0.051101	-2.514257
C	9.223917	-0.770373	1.306866	C	-1.91298	2.183242	-2.540346
C	10.33636	0.052127	1.506575	C	-2.262983	-1.430507	-2.310079
C	8.422253	-1.088482	2.406709	C	-3.481308	0.568432	-2.998382
C	10.645343	0.537392	2.773203	C	-1.354628	3.458373	-2.461417
H	10.963032	0.330978	0.663275	C	-3.230857	1.994163	-3.004692
C	8.725195	-0.601404	3.674154	C	-3.383352	-2.178466	-2.63882
H	7.547552	-1.719306	2.273277	H	-1.385404	-1.905531	-1.89369
C	9.838772	0.211098	3.858403	C	-4.59214	-0.203094	-3.333896
H	11.512404	1.176053	2.91217	C	-2.151413	4.537825	-2.816489
H	8.091004	-0.855301	4.518217	H	-0.334526	3.611441	-2.135145
H	10.076037	0.593286	4.846602	C	-4.013802	3.093998	-3.348306
C	9.060302	0.007682	-1.770985	C	-4.53478	-1.575958	-3.158576
C	9.564439	-0.245901	-3.048441	H	-3.366693	-3.250091	-2.473525
C	8.723909	1.322342	-1.43286	H	-5.495505	0.269523	-3.704741
C	9.718865	0.784605	-3.969956	C	-3.473295	4.365846	-3.242574
H	9.847385	-1.257012	-3.328046	H	-1.739374	5.538713	-2.743739
C	8.873493	2.354471	-2.352795	H	-5.034678	2.952377	-3.689881
H	8.350271	1.550823	-0.437797	H	-5.389485	-2.191672	-3.417748
C	9.370164	2.085469	-3.623764	H	-4.076508	5.23547	-3.480559
H	10.113385	0.572009	-4.958997	Ge	8.777491	-1.446692	-0.48272
H	8.604541	3.369371	-2.076319	C	-5.293812	0.849387	-0.086996
H	9.488769	2.890272	-4.342826	C	-5.453654	-0.504915	0.198991
N	3.652389	-0.14001	-0.0938	C	-6.592406	-1.194449	-0.217367

C	-7.566135	-0.504101	-0.941894
C	-7.403578	0.844387	-1.250088
C	-6.27347	1.521632	-0.822123
H	-4.670853	-1.005922	0.758079
H	-8.459053	-1.022945	-1.279229
H	-8.163208	1.366178	-1.824121
H	-6.123108	2.566504	-1.063481
C	-7.515688	-3.139185	2.132539
C	-7.361306	-4.293363	2.905987
C	-8.168629	-2.039568	2.694628
C	-7.855447	-4.351067	4.204965
H	-6.839518	-5.154611	2.496677
C	-8.662274	-2.092551	3.993931
H	-8.287224	-1.127758	2.115413
C	-8.507397	-3.249721	4.74962
H	-7.727021	-5.253954	4.794376
H	-9.164923	-1.228744	4.418458
H	-8.890531	-3.291819	5.764717
C	-5.175792	-4.087099	0.202598
C	-4.928786	-4.985161	-0.83834
C	-4.206672	-3.934284	1.198103
C	-3.739003	-5.704893	-0.89146
H	-5.674831	-5.130975	-1.614731
C	-3.017927	-4.654389	1.151846
H	-4.380216	-3.257511	2.030692
C	-2.781454	-5.538364	0.103901
H	-3.56297	-6.401851	-1.705514
H	-2.274518	-4.519319	1.930427
H	-1.854763	-6.103638	0.068065
C	-8.166812	-3.887641	-0.929192
C	-9.179846	-4.711551	-0.433304
C	-8.090481	-3.682485	-2.310482
C	-10.088722	-5.319586	-1.293458
H	-9.263677	-4.878444	0.637095
C	-8.996492	-4.288453	-3.17432
H	-7.321129	-3.032572	-2.718721
C	-9.997073	-5.10964	-2.664975
H	-10.871339	-5.956331	-0.892094
H	-8.923199	-4.118069	-4.244206
H	-10.706488	-5.583129	-3.336841
C	-4.106151	1.583582	0.395129
C	-3.025838	3.539178	0.690143
C	-2.108002	1.621889	1.425833
N	-1.073491	0.966841	2.067373
C	-0.170677	1.55275	2.983868
C	-0.62334	-0.342112	1.777551
C	-0.223395	2.780508	3.637407
C	0.848385	0.625744	3.261138
C	-1.150208	-1.303992	0.924279
C	0.577607	-0.561876	2.475723
C	0.778505	3.067136	4.556585
H	-1.015824	3.487617	3.439904
C	1.847267	0.935341	4.17787
C	-0.438552	-2.48759	0.77333
H	-2.076187	-1.137012	0.395648
C	1.269942	-1.758585	2.318722
C	1.80817	2.161454	4.823298
H	0.75538	4.020483	5.074997
H	2.643805	0.226724	4.380293
C	0.754011	-2.721541	1.463357
H	-0.833631	-3.249056	0.110084
H	2.20265	-1.926161	2.848279
H	2.582674	2.421213	5.537163
H	1.275523	-3.664894	1.332771
N	-2.94217	4.898775	0.468219
C	-3.959519	5.696932	-0.114779
C	-1.746975	5.662973	0.495327
C	-5.312748	5.438619	-0.31609
C	-3.403834	6.935253	-0.473626
C	-0.475784	5.350158	0.969666
C	-2.00606	6.912866	-0.093001
C	-6.080032	6.427283	-0.918745
H	-5.746881	4.497559	-0.021094
C	-4.186372	7.912555	-1.081224
C	0.525365	6.303935	0.826043
H	-0.278494	4.402367	1.442035
C	-0.99168	7.855729	-0.225225
C	-5.528111	7.649823	-1.31056
H	-7.135704	6.238958	-1.086973
H	-3.750308	8.865456	-1.364707
C	0.279382	7.54403	0.233667
H	1.522762	6.071335	1.186071
H	-1.196176	8.818467	-0.683323
H	-6.154598	8.398511	-1.784283
H	1.083708	8.265792	0.137033
N	-2.008656	2.947133	1.315478
N	-3.117243	0.887097	0.962619
N	-4.111518	2.907339	0.251263
Ge	-6.855772	-3.074303	0.284708

Supplementary Table 47. Atomic coordinates of GeCzCz:GeTrzCz2 (06, E = -4202.8023799 a.u.).

C	-1.732641	4.377525	-0.497353		C	-3.34056	2.885035	-1.686791
C	-0.943351	3.644216	0.38696		C	-1.752113	3.8721	-2.947511
C	-0.419168	4.252731	1.530292		C	-4.129332	2.437754	-0.629784
C	-0.696551	5.604696	1.755011		C	-3.537373	2.433267	-3.009884
C	-1.481278	6.335194	0.869894		C	-0.64736	4.596294	-3.38906
C	-2.004391	5.721165	-0.25989		C	-2.521752	3.07426	-3.8215
H	-0.735157	2.602981	0.153048		C	-5.090743	1.483259	-0.906517
H	-0.282825	6.099601	2.628364		H	-3.966771	2.789794	0.382671
H	-1.684191	7.384363	1.059681		C	-4.520423	1.478951	-3.269454
H	-2.622479	6.267902	-0.964363		C	-0.323134	4.50899	-4.734382
C	-0.300814	2.244318	4.093382		H	-0.064558	5.199652	-2.701703
C	0.290597	1.885968	5.309961		C	-2.171897	3.003714	-5.170647
C	-1.63226	1.887314	3.869159		C	-5.275892	0.995991	-2.211107
C	-0.422981	1.177347	6.270361		H	-5.689619	1.068102	-0.105952
H	1.316132	2.178029	5.520558		H	-4.684105	1.090117	-4.269101
C	-2.35169	1.187058	4.833304		C	-1.075069	3.722073	-5.619233
H	-2.118817	2.164845	2.937176		H	0.531315	5.062955	-5.110993
C	-1.746323	0.825747	6.030948		H	-2.747131	2.389049	-5.856253
H	0.053067	0.903777	7.207143		H	-0.789997	3.673878	-6.664741
H	-3.386834	0.91709	4.652616		N	-6.199727	-0.063801	-2.422141
H	-2.307545	0.27236	6.777123		C	-7.550138	-0.031142	-2.095768
C	1.736227	4.633673	3.834989		C	-5.827936	-1.393149	-2.616972
C	3.02993	5.010679	3.467324		C	-8.345798	1.063543	-1.769225
C	1.148819	5.255029	4.941573		C	-8.068274	-1.343292	-2.130305
C	3.716528	5.988769	4.17967		C	-4.567392	-1.921438	-2.884453
H	3.512546	4.531579	2.620058		C	-6.959286	-2.21854	-2.452888
C	1.830836	6.235338	5.65483		C	-9.67963	0.823317	-1.473979
H	0.151034	4.964512	5.260566		H	-7.931658	2.065388	-1.743021
C	3.116484	6.604001	5.272823		C	-9.414089	-1.557575	-1.835078
H	4.722857	6.268362	3.882743		C	-4.453009	-3.301024	-2.966277
H	1.360731	6.708123	6.511953		H	-3.70153	-1.280895	-3.002056
H	3.651794	7.366922	5.829932		C	-6.818711	-3.603652	-2.546524
C	2.054534	2.205701	1.827868		C	-10.213052	-0.472382	-1.506395
C	2.57852	1.042139	2.396457		H	-10.3232	1.65802	-1.214075
C	2.630939	2.694938	0.651607		H	-9.828708	-2.561059	-1.858143
C	3.68	0.407553	1.832892		C	-5.563907	-4.138586	-2.795758
H	2.136396	0.633848	3.300946		H	-3.479918	-3.742641	-3.148304
C	3.738719	2.068887	0.088938		H	-7.678335	-4.253051	-2.409067
H	2.224702	3.584328	0.176174		H	-11.261361	-0.625574	-1.27183
C	4.276625	0.93748	0.693561		H	-5.431291	-5.213637	-2.848784
H	4.075455	-0.496052	2.286578		Ge	0.740837	3.28754	2.797518
H	4.184841	2.466562	-0.817368		C	0.934826	-0.862792	-1.287246
H	5.155006	0.461862	0.269903		C	0.124086	-1.576301	-0.404486
N	-2.264312	3.757333	-1.661321		C	-1.243429	-1.321599	-0.314606

C	-1.790399	-0.320403	-1.120274		C	4.146148	-3.591179	1.474654
C	-0.990868	0.382921	-2.017697		C	7.300041	-2.684926	-0.118044
C	0.364783	0.111718	-2.105778		C	6.363507	-4.191096	1.578976
H	0.583561	-2.340928	0.210751		C	2.854734	-3.537674	1.910259
H	-2.847526	-0.087511	-1.05198		C	5.129436	-4.425157	2.142918
H	-1.424225	1.137429	-2.659001		C	8.588153	-3.195336	0.223152
H	0.989473	0.645994	-2.810195		H	7.201841	-1.915044	-0.864348
C	-4.076635	-1.528129	1.255617		C	7.678127	-4.696785	1.889975
C	-4.121536	-0.471442	2.16937		C	2.475608	-4.371238	3.0052
C	-5.269633	-1.954475	0.667266		H	2.13241	-2.884479	1.453787
C	-5.326169	0.141301	2.495903		C	4.713956	-5.264432	3.240116
H	-3.205555	-0.135714	2.644799		C	8.754304	-4.19183	1.209036
C	-6.480677	-1.352738	1.001418		H	9.454184	-2.802044	-0.296844
H	-5.26529	-2.76427	-0.055071		H	7.79568	-5.454785	2.656061
C	-6.511007	-0.306741	1.917203		C	3.404201	-5.22095	3.64161
H	-5.344794	0.96364	3.206175		H	1.446727	-4.341983	3.341547
H	-7.398823	-1.693306	0.53563		H	5.437514	-5.906548	3.72957
H	-7.456247	0.163098	2.171761		H	9.753028	-4.554861	1.430365
C	-1.365228	-2.562972	2.592907		H	3.070524	-5.843028	4.466246
C	-1.601146	-3.647185	3.44296		N	5.145822	-0.072692	-3.245652
C	-0.461028	-1.578556	3.00293		C	4.821579	1.179013	-3.82025
C	-0.96817	-3.73757	4.678633		C	6.411505	-0.467053	-3.728369
H	-2.28524	-4.434861	3.138093		C	3.705937	1.996063	-3.646929
C	0.180281	-1.670028	4.233956		C	5.89125	1.579864	-4.640232
H	-0.245296	-0.730276	2.359506		C	7.120854	-1.647819	-3.528022
C	-0.078172	-2.744764	5.076651		C	6.893813	0.533788	-4.589198
H	-1.16728	-4.584189	5.329106		C	3.678894	3.20969	-4.321896
H	0.870285	-0.892834	4.542096		H	2.88787	1.690942	-3.014484
H	0.419457	-2.812301	6.039378		C	5.843347	2.799994	-5.30921
C	-2.650603	-4.172584	0.111712		C	8.344953	-1.783817	-4.170238
C	-3.687065	-4.996246	0.559688		H	6.733341	-2.432711	-2.896296
C	-1.825892	-4.639977	-0.914459		C	8.123519	0.378343	-5.223187
C	-3.89931	-6.249056	-0.006485		C	4.731077	3.612258	-5.149187
H	-4.352708	-4.653988	1.347567		H	2.808033	3.847655	-4.206034
C	-2.031546	-5.89334	-1.481346		H	6.668507	3.108618	-5.943613
H	-1.018664	-4.015228	-1.286502		C	8.8511	-0.781124	-5.00261
C	-3.07205	-6.698753	-1.029839		H	8.914943	-2.695473	-4.021495
H	-4.71405	-6.872621	0.349043		H	8.499618	1.151308	-5.886224
H	-1.380815	-6.240587	-2.278338		H	4.676924	4.564258	-5.667152
H	-3.236444	-7.676047	-1.473619		H	9.812172	-0.917653	-5.487442
C	2.385806	-1.121687	-1.347762		N	5.01938	-1.5559	-1.458316
C	4.387093	-0.738879	-2.297657		N	2.908936	-1.960824	-0.461004
C	4.232831	-2.122174	-0.533931		N	3.083814	-0.478383	-2.286249
N	4.826836	-2.893689	0.431539		Ge	-2.350764	-2.39755	0.894643
C	6.214275	-3.197175	0.529847					

Supplementary Table 48. Atomic coordinates of GeCzCz:GeTrzCz2 (07, E = -4202.8057458 a.u.).

C	-1.816562	-1.352797	0.694922	C	-4.244784	-1.669339	0.257895
C	-0.763756	-1.811493	1.486097	C	-2.943615	-3.512532	0.09796
C	0.395644	-1.049483	1.642792	C	-4.765193	-0.400537	0.517564
C	0.459038	0.214893	1.057013	C	-5.061082	-2.699165	-0.25282
C	-0.610685	0.688364	0.312566	C	-1.898979	-4.435791	0.106524
C	-1.731544	-0.09517	0.102939	C	-4.230383	-3.879043	-0.349751
H	-0.849564	-2.773903	1.976624	C	-6.094573	-0.169641	0.213314
H	1.336106	0.841217	1.177731	H	-4.15951	0.386719	0.947005
H	-0.577692	1.669744	-0.137722	C	-6.392772	-2.44341	-0.571171
H	-2.519042	0.261029	-0.547108	C	-2.170718	-5.72589	-0.323327
C	1.85527	-0.999314	4.492989	H	-0.900179	-4.171377	0.422028
C	3.026032	-0.627873	5.156382	C	-4.477455	-5.179086	-0.787579
C	0.632629	-0.837701	5.150743	C	-6.900425	-1.172269	-0.34639
C	2.978388	-0.105327	6.445988	H	-6.533793	0.803278	0.405981
H	3.986099	-0.736265	4.659463	H	-7.028877	-3.210959	-0.999754
C	0.579436	-0.321145	6.440653	C	-3.443957	-6.102026	-0.769726
H	-0.292219	-1.107114	4.646778	H	-1.369239	-6.457615	-0.30681
C	1.754666	0.048179	7.089459	H	-5.466446	-5.461286	-1.13585
H	3.896877	0.182755	6.94855	H	-3.621069	-7.120922	-1.097873
H	-0.377812	-0.201811	6.939424	N	-8.241467	-0.863744	-0.690349
H	1.715748	0.455726	8.095099	C	-8.625059	0.095545	-1.619293
C	3.627157	-1.322544	1.845827	C	-9.385806	-1.408801	-0.118113
C	4.484485	-2.341342	1.422287	C	-7.828361	0.880515	-2.450251
C	4.046834	0.005497	1.700667	C	-10.033138	0.173071	-1.648755
C	5.731265	-2.046314	0.878561	C	-9.49552	-2.385421	0.868419
H	4.178283	-3.378701	1.5197	C	-10.521402	-0.794362	-0.687133
C	5.290149	0.303667	1.152396	C	-8.467811	1.767402	-3.303559
H	3.411724	0.821053	2.036628	H	-6.748029	0.786567	-2.435483
C	6.138191	-0.723953	0.750546	C	-10.651076	1.072681	-2.516508
H	6.387412	-2.84808	0.553003	C	-10.771702	-2.748825	1.271491
H	5.603525	1.337301	1.040435	H	-8.613228	-2.840586	1.304524
H	7.11418	-0.492067	0.338561	C	-11.793238	-1.179212	-0.264565
C	1.663999	-3.704073	2.806603	C	-9.865394	1.868803	-3.336181
C	1.556893	-4.333538	4.048505	H	-7.872376	2.392034	-3.962336
C	1.557426	-4.484947	1.650344	H	-11.73394	1.146244	-2.551145
C	1.345424	-5.706405	4.136603	C	-11.911967	-2.15632	0.711658
H	1.633609	-3.746291	4.959167	H	-10.888013	-3.508164	2.038509
C	1.348104	-5.857262	1.734948	H	-12.67752	-0.717221	-0.693319
H	1.639875	-4.025394	0.669919	H	-10.334856	2.573794	-4.014321
C	1.239262	-6.469283	2.979458	H	-12.895824	-2.4657	1.048834
H	1.261245	-6.179779	5.110223	Ge	1.904929	-1.759711	2.68118
H	1.270998	-6.442943	0.825018	C	2.80368	3.047575	-1.988666
H	1.071407	-7.539967	3.046732	C	1.633363	3.284101	-1.273816
N	-2.957016	-2.161233	0.462511	C	1.190364	4.57858	-1.0082

C	1.930251	5.647724	-1.516394		C	0.474854	-1.418423	-2.532627
C	3.0945	5.421444	-2.249851		C	3.263209	-3.581783	-1.613751
C	3.545388	4.12666	-2.470384		C	0.88638	-3.660214	-2.235432
H	1.101381	2.422363	-0.891575		C	-0.264178	-0.324099	-2.864091
H	1.609991	6.66781	-1.322332		C	-0.10383	-2.749855	-2.536149
H	3.661595	6.262451	-2.636438		C	3.221726	-5.00197	-1.492776
H	4.463985	3.939023	-3.015638		H	4.170383	-3.041986	-1.398448
C	-0.562942	6.704324	0.662249		C	0.882511	-5.097895	-2.123606
C	0.147925	7.328316	1.690692		C	-1.644245	-0.521096	-3.186215
C	-1.450923	7.467721	-0.100336		H	0.163629	0.66576	-2.860516
C	-0.017348	8.685443	1.945875		C	-1.506424	-2.91171	-2.84291
H	0.831042	6.748286	2.305772		C	2.04484	-5.732427	-1.766483
C	-1.618861	8.825054	0.152557		H	4.121307	-5.523704	-1.187037
H	-2.025975	6.998314	-0.894415		H	-0.02898	-5.64774	-2.323612
C	-0.900396	9.434908	1.175538		C	-2.240691	-1.79777	-3.156785
H	0.539556	9.157708	2.749496		H	-2.24456	0.344428	-3.438506
H	-2.313233	9.406417	-0.446469		H	-1.956341	-3.896236	-2.805751
H	-1.032021	10.493993	1.375396		H	2.06577	-6.815117	-1.686529
C	0.32246	3.842967	1.874142		H	-3.298844	-1.894673	-3.377752
C	-0.555981	3.174243	2.729817		N	6.267945	-0.133635	-2.414068
C	1.690315	3.807044	2.165555		C	7.313853	0.751086	-2.047348
C	-0.0842	2.493334	3.846377		C	6.834481	-1.406727	-2.662887
H	-1.619785	3.157625	2.51249		C	7.285979	2.095439	-1.680547
C	2.164819	3.131085	3.285753		C	8.521435	0.029953	-2.044065
H	2.398988	4.300511	1.506001		C	6.247414	-2.586065	-3.114381
C	1.27702	2.471727	4.129072		C	8.218936	-1.331388	-2.437702
H	-0.77588	1.962617	4.492659		C	8.487015	2.69346	-1.318255
H	3.229803	3.114434	3.499046		H	6.361288	2.649813	-1.683104
H	1.643515	1.934931	4.996384		C	9.714003	0.649499	-1.682112
C	-1.961916	4.037989	-0.424692		C	7.067719	-3.693329	-3.289322
C	-3.128324	4.086083	0.347077		H	5.190332	-2.641741	-3.317914
C	-2.022056	3.423625	-1.6782		C	9.025079	-2.451839	-2.618316
C	-4.312563	3.515367	-0.107231		C	9.692086	1.986975	-1.318749
H	-3.121559	4.584063	1.313351		H	8.481752	3.740312	-1.031388
C	-3.209132	2.861999	-2.141868		H	10.643317	0.088401	-1.680904
H	-1.133588	3.381346	-2.302717		C	8.441203	-3.637431	-3.036738
C	-4.354715	2.899494	-1.354134		H	6.622702	-4.621401	-3.63405
H	-5.207215	3.561322	0.506621		H	10.094092	-2.392058	-2.439176
H	-3.243337	2.406204	-3.126647		H	10.611724	2.486866	-1.032941
H	-5.282274	2.464298	-1.712264		H	9.053032	-4.522007	-3.180209
C	3.257567	1.650679	-2.152922		N	4.073209	-0.880721	-2.283395
C	4.917454	0.145343	-2.327657		N	2.324909	0.702989	-2.112562
C	2.782671	-0.549177	-2.197119		N	4.564295	1.43245	-2.290346
N	1.854335	-1.561102	-2.206994		Ge	-0.289539	4.804605	0.268185
C	2.124995	-2.938954	-2.006371					

Supplementary Table 49. Atomic coordinates of GeCzCz:GeTrzCz2 (08, E = -4202.7949005 a.u.).

C	-2.769395	-1.464577	2.382489	C	-4.794955	-1.101707	0.968032
C	-1.424982	-1.096508	2.436712	C	-3.827332	0.74215	1.850912
C	-0.506878	-1.867779	3.146317	C	-5.089358	-2.388566	0.516939
C	-0.937451	-3.060683	3.734155	C	-5.61764	-0.008771	0.630864
C	-2.272757	-3.438579	3.667855	C	-3.019294	1.646165	2.53837
C	-3.195242	-2.629052	3.020728	C	-4.988931	1.172467	1.178089
H	-1.101366	-0.208901	1.907482	C	-6.236787	-2.56765	-0.237663
H	-0.229269	-3.696682	4.258515	H	-4.430659	-3.222178	0.724801
H	-2.605179	-4.355512	4.144458	C	-6.783398	-0.210816	-0.103037
H	-4.247146	-2.888771	3.018633	C	-3.36693	2.988055	2.496379
C	2.540576	-2.79732	3.014329	H	-2.157348	1.31819	3.103606
C	3.059427	-3.617644	4.019077	C	-5.316859	2.525388	1.144689
C	2.815657	-3.119803	1.682116	C	-7.093771	-1.494102	-0.52893
C	3.819708	-4.739566	3.701513	H	-6.490544	-3.55238	-0.615023
H	2.8699	-3.379559	5.062069	H	-7.444647	0.614309	-0.345309
C	3.569858	-4.240802	1.361397	C	-4.496391	3.431135	1.796925
H	2.450156	-2.486022	0.879141	H	-2.745124	3.7063	3.021457
C	4.070435	-5.054356	2.371571	H	-6.20082	2.861895	0.612722
H	4.216026	-5.366736	4.494632	H	-4.735483	4.489033	1.772054
H	3.776845	-4.467591	0.323168	N	-8.281315	-1.735324	-1.266396
H	4.666744	-5.925679	2.119422	C	-9.3178	-2.577569	-0.87393
C	1.524895	-0.964133	5.413114	C	-8.611509	-1.173439	-2.495304
C	2.698291	-0.366415	5.884917	C	-9.444412	-3.334516	0.288423
C	0.526867	-1.287382	6.333846	C	-10.325169	-2.559472	-1.861062
C	2.875369	-0.110714	7.239408	C	-7.879931	-0.291025	-3.285781
H	3.483267	-0.087416	5.186126	C	-9.872251	-1.659103	-2.900998
C	0.698455	-1.030401	7.691253	C	-10.598359	-4.088458	0.441222
H	-0.399045	-1.741345	5.993614	H	-8.669355	-3.329467	1.046455
C	1.873632	-0.444183	8.145854	C	-11.475384	-3.327131	-1.683491
H	3.792831	0.353688	7.588325	C	-8.436254	0.109414	-4.491248
H	-0.08913	-1.286397	8.393743	H	-6.908781	0.066396	-2.968755
H	2.00817	-0.242841	9.204342	C	-10.411206	-1.239833	-4.116539
C	1.895185	0.353963	2.599236	C	-11.60532	-4.090189	-0.533475
C	3.184821	0.462356	2.072131	H	-10.723452	-4.688028	1.337496
C	1.111384	1.509394	2.681052	H	-12.259061	-3.323558	-2.435169
C	3.686865	1.692692	1.659986	C	-9.691547	-0.35537	-4.905386
H	3.810549	-0.42206	1.99747	H	-7.884199	0.795594	-5.126058
C	1.603781	2.739843	2.259595	H	-11.38106	-1.604723	-4.441205
H	0.111705	1.462976	3.10328	H	-12.495025	-4.692743	-0.382797
C	2.899999	2.834723	1.762155	H	-10.100267	-0.021585	-5.853435
H	4.692839	1.761676	1.257481	Ge	1.34825	-1.309472	3.478794
H	0.980594	3.626478	2.331758	C	3.767572	1.983476	-1.717848
H	3.290423	3.794274	1.44032	C	3.939076	0.605818	-1.859687
N	-3.71189	-0.6453	1.717737	C	5.169884	0.06759	-2.224512

C	6.241318	0.939559	-2.443072
C	6.086592	2.312125	-2.279021
C	4.853048	2.836106	-1.915223
H	3.080606	-0.03179	-1.686704
H	7.207862	0.544496	-2.744789
H	6.929209	2.976986	-2.440904
H	4.719498	3.902409	-1.778073
C	6.742557	-2.301246	-3.734536
C	6.327321	-2.60382	-5.033713
C	8.11237	-2.275678	-3.457501
C	7.256715	-2.8648	-6.034786
H	5.266394	-2.642802	-5.266457
C	9.045107	-2.534855	-4.456748
H	8.457895	-2.06404	-2.448799
C	8.617133	-2.828277	-5.747043
H	6.919427	-3.099778	-7.039778
H	10.1062	-2.512068	-4.227186
H	9.343691	-3.032867	-6.527555
C	3.71521	-2.752553	-2.754635
C	3.501241	-4.076382	-2.359891
C	2.697586	-2.097344	-3.454586
C	2.3029	-4.724198	-2.640547
H	4.282152	-4.61357	-1.828292
C	1.494517	-2.738478	-3.732396
H	2.832235	-1.069534	-3.777573
C	1.294829	-4.051761	-3.323406
H	2.154885	-5.751758	-2.322289
H	0.706792	-2.206373	-4.25612
H	0.351909	-4.547422	-3.531904
C	6.119934	-2.482944	-0.60795
C	6.766757	-3.7143	-0.467858
C	5.961691	-1.681078	0.524629
C	7.22831	-4.137485	0.774515
H	6.927074	-4.346678	-1.337746
C	6.41891	-2.100641	1.768793
H	5.475442	-0.714283	0.431959
C	7.050371	-3.33235	1.894875
H	7.729156	-5.096533	0.868376
H	6.275617	-1.469629	2.640697
H	7.401634	-3.666082	2.866163
C	2.436305	2.519206	-1.3653
C	1.104616	4.255639	-0.846909
C	0.24553	2.186043	-0.986585
N	-0.846915	1.353264	-1.002945
C	-2.161637	1.750357	-1.383858
C	-0.795388	-0.064772	-0.983825
C	-2.71653	2.982633	-1.549344
C	-2.904994	0.537589	-1.666944
C	0.201192	-0.917288	-0.607441
C	-2.099485	-0.548768	-1.397702
C	-4.057742	3.050654	-2.044878
H	-2.171491	3.886169	-1.32811
C	-4.249313	0.64202	-2.177518
C	-0.07253	-2.315312	-0.63952
H	1.166791	-0.550707	-0.291735
C	-2.333421	-1.970168	-1.449761
C	-4.788456	1.890673	-2.360576
H	-4.508538	4.026311	-2.184901
H	-4.803476	-0.260669	-2.405025
C	-1.325305	-2.812754	-1.062613
H	0.703897	-3.01124	-0.349323
H	-3.297786	-2.344801	-1.770901
H	-5.799723	1.993752	-2.742334
H	-1.481415	-3.886852	-1.080663
N	0.954027	5.592894	-0.530992
C	1.853573	6.631562	-0.87139
C	-0.086152	6.145969	0.256081
C	2.998983	6.616105	-1.663765
C	1.38304	7.828764	-0.306784
C	-1.183579	5.544148	0.870564
C	0.164163	7.520971	0.412972
C	3.669808	7.817125	-1.855584
H	3.349623	5.700149	-2.111986
C	2.071677	9.021497	-0.507441
C	-2.024131	6.355056	1.624437
H	-1.384001	4.48926	0.76132
C	-0.692706	8.31329	1.170364
C	3.221232	9.009791	-1.28161
H	4.565536	7.823078	-2.468607
H	1.707087	9.944911	-0.068314
C	-1.792107	7.724027	1.774633
H	-2.887165	5.90605	2.103862
H	-0.496185	9.374449	1.287567
H	3.771984	9.929582	-1.448884
H	-2.473048	8.325274	2.368034
N	0.027049	3.482174	-0.754181
N	1.439351	1.648685	-1.242971
N	2.319538	3.839504	-1.201077
Ge	5.425701	-1.876133	-2.342739

Supplementary Table 50. Atomic coordinates of GeCzCz:GeTrzCz2 (09, E = -4202.7859437 a.u.).

C	3.892628	-0.735173	-0.018488	C	5.017541	1.333205	0.798606
C	3.747522	-2.07361	0.333634	C	3.43856	0.435216	2.143047
C	3.366169	-3.023753	-0.614971	C	5.940666	1.589329	-0.213262
C	3.192964	-2.61695	-1.939053	C	4.831371	2.236026	1.864598
C	3.375559	-1.286072	-2.30076	C	2.446367	-0.357194	2.718689
C	3.703929	-0.340039	-1.341951	C	3.825687	1.658619	2.729169
H	3.915587	-2.357684	1.365433	C	6.641526	2.781655	-0.164942
H	2.883752	-3.33243	-2.694031	H	6.109822	0.880514	-1.014476
H	3.2139	-0.975602	-3.327641	C	5.527497	3.441318	1.886018
H	3.774784	0.706965	-1.604501	C	1.859036	0.093997	3.89195
C	4.676188	-5.900645	-0.00844	H	2.142476	-1.297303	2.278308
C	5.759793	-5.556893	-0.820951	C	3.219058	2.091955	3.907242
C	4.803748	-6.991054	0.855827	C	6.423592	3.713761	0.861394
C	6.940666	-6.290029	-0.777839	H	7.373166	3.014537	-0.930816
H	5.685223	-4.702934	-1.489392	H	5.368168	4.171318	2.672834
C	5.983401	-7.726724	0.901603	C	2.235759	1.305352	4.485388
H	3.977891	-7.265224	1.50715	H	1.09054	-0.510918	4.361491
C	7.052237	-7.376828	0.083321	H	3.512379	3.033924	4.360718
H	7.77589	-6.011176	-1.413129	H	1.753713	1.626223	5.402624
H	6.070059	-8.571259	1.578543	N	7.112832	4.951577	0.830641
H	7.974398	-7.948868	0.119694	C	6.999908	5.903993	-0.177405
C	1.819238	-5.617565	-1.494818	C	7.942716	5.456821	1.826431
C	2.159211	-6.768857	-2.20742	C	6.255279	5.856867	-1.353973
C	0.636731	-4.946582	-1.827912	C	7.773405	7.0322	0.166938
C	1.340565	-7.239166	-3.230859	C	8.352276	4.861699	3.017209
H	3.075727	-7.301758	-1.968888	C	8.378778	6.744878	1.451273
C	-0.184917	-5.415977	-2.846611	C	6.30597	6.961552	-2.191202
H	0.351221	-4.04588	-1.29443	H	5.660936	4.985422	-1.605779
C	0.168997	-6.562561	-3.552403	C	7.809532	8.128222	-0.693736
H	1.619647	-8.13395	-3.779051	C	9.204254	5.586531	3.837095
H	-1.09955	-4.882104	-3.086647	H	8.018813	3.866373	3.288727
H	-0.46938	-6.929029	-4.350704	C	9.235658	7.452149	2.293449
C	2.111468	-4.928976	1.65127	C	7.076336	8.086964	-1.869885
C	2.739143	-4.395914	2.78274	H	5.734843	6.95236	-3.114347
C	0.80884	-5.414348	1.784996	H	8.401005	9.003664	-0.442934
C	2.068686	-4.301983	3.998081	C	9.642583	6.870002	3.483962
H	3.768035	-4.050748	2.726106	H	9.539271	5.147302	4.771577
C	0.139459	-5.340471	3.003022	H	9.579888	8.444664	2.018557
H	0.299772	-5.834945	0.922895	H	7.095434	8.933757	-2.548015
C	0.76215	-4.768847	4.10717	H	10.308063	7.410108	4.149363
H	2.567896	-3.868977	4.859468	Ge	2.99884	-4.891519	-0.104631
H	-0.874185	-5.721049	3.087793	C	-4.215587	1.821567	-1.929732
H	0.235802	-4.699023	5.054394	C	-2.980386	1.18136	-1.837884
N	4.176176	0.238192	0.97219	C	-2.041789	1.25396	-2.86334

C	-2.359233	2.013315	-3.992488		C	-4.059661	-0.439942	2.579495
C	-3.57787	2.681842	-4.083303		C	-7.647286	-0.962283	3.003613
C	-4.510429	2.585622	-3.058511		C	-5.476655	-1.664279	3.913024
H	-2.786724	0.588781	-0.95335		C	-2.858767	0.062016	2.173347
H	-1.658556	2.084177	-4.819704		C	-4.147534	-1.412138	3.653947
H	-3.805418	3.273303	-4.964629		C	-8.260392	-1.846549	3.94231
H	-5.465197	3.094745	-3.122325		H	-8.252757	-0.38993	2.319623
C	0.319061	-0.540544	-4.258272		C	-6.128264	-2.536072	4.858885
C	0.512525	-1.925503	-4.262707		C	-1.678439	-0.414784	2.821508
C	0.622571	0.173059	-5.421932		H	-2.789297	0.793461	1.384091
C	1.002624	-2.57998	-5.387262		C	-2.932927	-1.916666	4.248628
H	0.287816	-2.50459	-3.373529		C	-7.497529	-2.608442	4.850918
C	1.115317	-0.476107	-6.550143		H	-9.341525	-1.924075	3.95017
H	0.480767	1.250085	-5.459852		H	-5.533091	-3.117882	5.55359
C	1.30857	-1.853549	-6.533287		C	-1.734745	-1.406374	3.82428
H	1.142765	-3.656831	-5.36215		H	-0.71875	-0.016713	2.516412
H	1.34836	0.094931	-7.443806		H	-2.986135	-2.677672	5.019072
H	1.694356	-2.358906	-7.413402		H	-8.008205	-3.260255	5.552765
C	-0.702508	-1.106663	-1.292944		H	-0.809382	-1.780398	4.251994
C	0.158054	-1.391809	-0.230536		N	-8.585288	2.283805	-0.158549
C	-1.889385	-1.841016	-1.401991		C	-9.207924	2.711873	-1.357458
C	-0.177636	-2.354691	0.716013		C	-9.521197	2.413916	0.896888
H	1.096089	-0.856206	-0.132816		C	-8.733413	2.743042	-2.666546
C	-2.228968	-2.801824	-0.455829		C	-10.526351	3.095574	-1.058768
H	-2.576178	-1.642928	-2.220355		C	-9.385566	2.18477	2.264179
C	-1.376051	-3.051058	0.612098		C	-10.721559	2.91554	0.365573
H	0.493434	-2.567727	1.540151		C	-9.60043	3.182169	-3.659203
H	-3.164507	-3.345048	-0.546181		H	-7.725141	2.438162	-2.897453
H	-1.641468	-3.7814	1.367874		C	-11.377863	3.537007	-2.067079
C	0.984533	1.596515	-1.903258		C	-10.48424	2.4384	3.076146
C	1.918087	2.218738	-2.735602		H	-8.460584	1.819214	2.681517
C	0.977703	1.93355	-0.546411		C	-11.811226	3.161363	1.195441
C	2.825569	3.143846	-2.226256		C	-10.907727	3.581767	-3.37025
H	1.960319	1.965578	-3.790998		H	-9.245941	3.215208	-4.684607
C	1.891544	2.845884	-0.030261		H	-12.395164	3.834994	-1.832947
H	0.262323	1.469011	0.126693		C	-11.689427	2.914752	2.554225
C	2.819868	3.4524	-0.869995		H	-10.395523	2.259951	4.143067
H	3.546506	3.615506	-2.888066		H	-12.738055	3.546461	0.781712
H	1.886161	3.074288	1.030305		H	-11.556787	3.92381	-4.169714
H	3.539563	4.156998	-0.465328		H	-12.529125	3.099043	3.216309
C	-5.205056	1.614285	-0.851072		N	-7.013808	1.066499	1.033199
C	-7.298345	1.795491	-0.044484		N	-4.800321	0.926803	0.214761
C	-5.746267	0.653656	1.113779		N	-6.441818	2.083529	-1.026322
N	-5.391513	-0.132643	2.183608		Ge	-0.326913	0.307563	-2.606041
C	-6.286022	-0.871629	3.005234					

Supplementary Table 51. Atomic coordinates of GeCzCz:GeTrzCz2 (10, E = -4202.7880771 a.u.).

C	-4.932285	0.48242	-1.440435	C	-7.350264	0.986973	-1.041875
C	-4.020024	-0.337966	-0.785868	C	-6.842923	-1.088259	-1.780098
C	-2.650971	-0.075814	-0.84352	C	-7.306833	2.296906	-0.569021
C	-2.2143	1.06014	-1.524315	C	-8.56954	0.286497	-1.160104
C	-3.12864	1.903011	-2.152044	C	-6.194614	-2.233787	-2.238754
C	-4.485369	1.609214	-2.129852	C	-8.244416	-1.041315	-1.63577
H	-4.395201	-1.194541	-0.233303	C	-8.50435	2.899782	-0.221938
H	-1.153812	1.289078	-1.571545	H	-6.365924	2.821966	-0.453755
H	-2.780006	2.783639	-2.682753	C	-9.765237	0.91856	-0.831253
H	-5.200134	2.23532	-2.653218	C	-6.977703	-3.337552	-2.538768
C	-2.149083	-1.696894	1.789976	H	-5.117849	-2.269574	-2.352231
C	-2.187602	-0.649368	2.71673	C	-9.01006	-2.164891	-1.945605
C	-2.787382	-2.895148	2.11903	C	-9.726485	2.223575	-0.359312
C	-2.837821	-0.795508	3.935971	H	-8.510727	3.909912	0.172903
H	-1.721099	0.30352	2.481876	H	-10.718622	0.41093	-0.933933
C	-3.44629	-3.042677	3.335996	C	-8.371608	-3.309888	-2.394143
H	-2.786512	-3.722286	1.416967	H	-6.494097	-4.24326	-2.891286
C	-3.474034	-1.992627	4.245417	H	-10.090218	-2.140008	-1.837612
H	-2.854913	0.028992	4.641977	H	-8.953348	-4.193167	-2.636183
H	-3.946172	-3.978614	3.56836	N	-10.93048	2.879609	0.003902
H	-3.992491	-2.104692	5.193002	C	-11.416207	4.057247	-0.554601
C	0.371009	-0.604592	-0.049102	C	-11.821259	2.455611	0.985249
C	1.016855	-0.436762	-1.279303	C	-10.879048	4.833389	-1.57914
C	0.976418	-0.089064	1.098958	C	-12.63325	4.396929	0.072949
C	2.221073	0.253568	-1.363295	C	-11.746117	1.345025	1.822719
H	0.581787	-0.851994	-2.184754	C	-12.893035	3.369744	1.060083
C	2.182557	0.602095	1.016563	C	-11.577982	5.969601	-1.958449
H	0.51276	-0.228842	2.070679	H	-9.949825	4.554541	-2.063126
C	2.802512	0.782899	-0.215006	C	-13.316049	5.544672	-0.32712
H	2.714285	0.375384	-2.322654	C	-12.774422	1.156714	2.733925
H	2.643154	0.995905	1.917456	H	-10.910603	0.656599	1.764793
H	3.738095	1.326844	-0.283289	C	-13.914796	3.156732	1.984468
C	-1.626655	-3.018895	-1.09181	C	-12.783531	6.327407	-1.339831
C	-1.22258	-4.266972	-0.609209	H	-11.181866	6.592955	-2.754107
C	-2.260226	-2.96243	-2.337039	H	-14.253533	5.819021	0.147359
C	-1.47181	-5.427354	-1.335863	C	-13.851753	2.049379	2.815952
H	-0.717998	-4.34759	0.347732	H	-12.741111	0.298998	3.398554
C	-2.504368	-4.119422	-3.071301	H	-14.746558	3.85153	2.052842
H	-2.578168	-2.004876	-2.738789	H	-13.304166	7.223932	-1.66018
C	-2.119333	-5.356675	-2.565455	H	-14.640971	1.871201	3.539035
H	-1.157557	-6.384942	-0.934969	Ge	-1.425182	-1.384411	-0.010957
H	-3.000209	-4.05392	-4.035359	C	5.676898	-0.117138	2.242128
H	-2.318572	-6.262829	-3.129602	C	6.277371	0.781732	1.364585
N	-6.308311	0.149869	-1.423275	C	6.968054	1.900773	1.818521

C	7.058883	2.107131	3.195393		C	5.258306	-1.72372	-2.394086
C	6.465877	1.210494	4.08542		C	2.111357	-3.559154	-2.160114
C	5.767197	0.104335	3.616358		C	3.566841	-2.42731	-3.781485
H	6.159013	0.599691	0.302884		C	6.474471	-1.182775	-2.092986
H	7.580823	2.977615	3.583363		C	4.761397	-1.741658	-3.756901
H	6.54166	1.383689	5.15442		C	1.225612	-3.843063	-3.243627
H	5.289605	-0.588685	4.300606		H	1.854613	-3.86299	-1.158976
C	8.331099	4.767838	1.083543		C	2.653716	-2.73451	-4.855375
C	7.843137	5.995375	0.630275		C	7.234523	-0.595889	-3.151037
C	9.346869	4.765577	2.04538		H	6.862213	-1.181838	-1.087483
C	8.349569	7.190941	1.131067		C	5.536468	-1.113626	-4.799726
H	7.060655	6.022618	-0.122581		C	1.508764	-3.427669	-4.561674
C	9.853049	5.957861	2.549967		H	0.314421	-4.387098	-3.036117
H	9.755948	3.823822	2.403611		H	2.88204	-2.412448	-5.865101
C	9.352553	7.173097	2.093308		C	6.748711	-0.559663	-4.47496
H	7.960316	8.137461	0.768384		H	8.204671	-0.171931	-2.917984
H	10.640732	5.939702	3.297005		H	5.160897	-1.101459	-5.816734
H	9.74739	8.105366	2.485389		H	0.802088	-3.666791	-5.350237
C	6.111661	3.316196	-0.823851		H	7.353265	-0.091526	-5.245614
C	6.041483	2.591378	-2.016055		N	2.178318	-3.292667	2.457114
C	5.010667	4.097459	-0.455438		C	1.542656	-2.757253	3.602858
C	4.905932	2.640033	-2.819686		C	1.506796	-4.476167	2.089723
H	6.873279	1.966682	-2.323483		C	1.810685	-1.599904	4.329622
C	3.879844	4.161547	-1.262291		C	0.45956	-3.588442	3.936414
H	5.026526	4.657291	0.476428		C	1.807626	-5.409932	1.101493
C	3.825145	3.42979	-2.445146		C	0.440893	-4.681002	2.984311
H	4.870193	2.050848	-3.731387		C	0.976445	-1.303522	5.400187
H	3.033362	4.770806	-0.960727		H	2.646161	-0.967058	4.073427
H	2.935226	3.467442	-3.065861		C	-0.355228	-3.279619	5.020517
C	9.026216	2.077075	-0.594893		C	0.998996	-6.535758	1.007681
C	9.621469	2.640918	-1.727466		H	2.640447	-5.263552	0.429669
C	9.397213	0.783414	-0.221606		C	-0.370371	-5.805497	2.860195
C	10.5545	1.928821	-2.47305		C	-0.09233	-2.132121	5.750843
H	9.350431	3.646387	-2.040118		H	1.169717	-0.406422	5.980057
C	10.333677	0.067486	-0.96234		H	-1.193436	-3.918799	5.274492
H	8.947904	0.322616	0.6531		C	-0.087723	-6.731226	1.866179
C	10.911263	0.638733	-2.091027		H	1.219097	-7.275353	0.244377
H	11.004594	2.380083	-3.352091		H	-1.201638	-5.95776	3.541228
H	10.609313	-0.938048	-0.659304		H	-0.723479	-1.871374	6.593882
H	11.639084	0.080672	-2.672105		H	-0.705976	-7.616717	1.760017
C	4.88239	-1.21343	1.651852		N	3.254128	-2.940367	0.432422
C	3.180608	-2.670924	1.734205		N	5.083809	-1.450662	0.362518
C	4.213641	-2.276346	-0.217997		N	3.9624	-1.82028	2.401148
N	4.29118	-2.390096	-1.583919		Ge	7.642345	3.072702	0.389776
C	3.251143	-2.864117	-2.432148					

Supplementary Table 52. Atomic coordinates of GeCzCz:GeTrzCz2 (11, E = -4202.8009526 a.u.).

C	-3.22706	4.87003	-0.102044	C	-4.614286	2.806575	-0.022625
C	-1.962916	4.333823	-0.336356	C	-5.441536	4.531221	-1.229345
C	-0.810152	5.024599	0.030983	C	-3.845731	1.979271	0.792387
C	-0.951163	6.27696	0.638715	C	-5.857289	2.383365	-0.536466
C	-2.211016	6.826535	0.849765	C	-5.628099	5.739304	-1.897018
C	-3.354431	6.124218	0.488228	C	-6.384532	3.485495	-1.314321
H	-1.895905	3.358804	-0.805686	C	-4.328392	0.712115	1.068209
H	-0.074293	6.828706	0.964906	H	-2.889869	2.296322	1.190908
H	-2.306554	7.799569	1.321248	C	-6.343174	1.118188	-0.219543
H	-4.342261	6.528592	0.681032	C	-6.789271	5.889413	-2.640562
C	1.648094	3.717604	1.546354	H	-4.889188	6.530604	-1.843173
C	3.017832	3.584415	1.787707	C	-7.543951	3.66148	-2.067956
C	0.754655	3.408656	2.575987	C	-5.568712	0.283271	0.574781
C	3.484451	3.138186	3.020096	H	-3.734784	0.031241	1.662474
H	3.733153	3.832619	1.007879	H	-7.310803	0.7838	-0.577261
C	1.216263	2.962835	3.810312	C	-7.742168	4.865452	-2.725509
H	-0.315051	3.519017	2.41579	H	-6.959997	6.82124	-3.170407
C	2.582034	2.825496	4.030782	H	-8.277214	2.863981	-2.140795
H	4.54981	3.028392	3.193293	H	-8.640058	5.017426	-3.315217
H	0.510619	2.720804	4.599673	N	-6.033929	-1.015257	0.899986
H	2.946924	2.472851	4.989189	C	-6.164707	-1.532687	2.186522
C	2.207992	5.374395	-1.181608	C	-6.55404	-1.939998	-0.001312
C	3.380102	4.820538	-1.707397	C	-5.814697	-0.964892	3.410116
C	1.981177	6.740773	-1.357398	C	-6.769018	-2.805522	2.111642
C	4.310844	5.613884	-2.368499	C	-6.601267	-1.884971	-1.393021
H	3.565457	3.753167	-1.615782	C	-7.034505	-3.05862	0.711644
C	2.907258	7.537218	-2.023877	C	-6.051182	-1.708174	4.556941
H	1.067958	7.19399	-0.984635	H	-5.371454	0.022118	3.463717
C	4.076043	6.97585	-2.525046	C	-6.991626	-3.535003	3.278663
H	5.2159	5.167239	-2.768813	C	-7.173836	-2.958041	-2.058764
H	2.713736	8.597756	-2.153992	H	-6.182562	-1.048059	-1.938184
H	4.799558	7.596989	-3.044181	C	-7.621347	-4.116964	0.01918
C	0.801657	2.531965	-1.259305	C	-6.626369	-2.984874	4.49716
C	1.598482	1.442288	-0.90039	H	-5.785972	-1.287553	5.522162
C	-0.022771	2.409986	-2.38314	H	-7.446813	-4.519686	3.231205
C	1.578241	0.270284	-1.64691	C	-7.693237	-4.059806	-1.364498
H	2.241306	1.499845	-0.025906	H	-7.211099	-2.941912	-3.143289
C	-0.077395	1.221938	-3.10564	H	-8.009111	-4.976345	0.558237
H	-0.636716	3.247573	-2.702979	H	-6.791734	-3.54206	5.41343
C	0.728375	0.148316	-2.740131	H	-8.144953	-4.877383	-1.916921
H	2.211833	-0.555541	-1.357731	Ge	0.953641	4.198466	-0.229426
H	-0.749298	1.133439	-3.953302	C	2.264262	-3.187709	-1.383361
H	0.690858	-0.782356	-3.297428	C	0.993729	-2.953353	-0.851149
N	-4.371561	4.112597	-0.441598	C	-0.157719	-3.291281	-1.552854

C	-0.015328	-3.907934	-2.800501		C	3.118127	-1.300203	3.248417
C	1.244477	-4.176708	-3.326547		C	6.234297	0.44995	2.521462
C	2.38623	-3.809698	-2.62539		C	4.629855	0.048425	4.336288
H	0.924662	-2.452277	0.106309		C	1.98741	-2.048921	3.119544
H	-0.896574	-4.17965	-3.375936		C	3.429232	-0.608789	4.486286
H	1.337395	-4.662672	-4.292699		C	6.936004	1.317778	3.411152
H	3.374397	-3.995324	-3.029929		H	6.58629	0.30425	1.512867
C	-2.979577	-1.859561	-2.131494		C	5.380413	0.904353	5.22169
C	-3.101137	-0.474145	-1.999479		C	1.10596	-2.13129	4.241948
C	-3.580286	-2.480215	-3.230483		H	1.751206	-2.565088	2.204277
C	-3.80388	0.274761	-2.938293		C	2.506089	-0.691098	5.592298
H	-2.656072	0.033808	-1.151507		C	6.507245	1.520882	4.740021
C	-4.276083	-1.73539	-4.177161		H	7.826083	1.820417	3.050507
H	-3.524164	-3.559931	-3.343628		H	5.046433	1.050691	6.242826
C	-4.389885	-0.35576	-4.031259		C	1.372488	-1.44821	5.445624
H	-3.900359	1.348376	-2.808556		H	0.202681	-2.719962	4.143801
H	-4.736435	-2.232745	-5.025844		H	2.722277	-0.163919	6.514774
H	-4.938628	0.226324	-4.765643		H	7.08324	2.175359	5.386653
C	-1.753032	-1.870224	0.839554		H	0.665415	-1.528456	6.265354
C	-2.424306	-2.266976	1.99983		N	6.85648	-2.060914	-1.307897
C	-0.965348	-0.713908	0.884291		C	7.077033	-2.210981	-2.699713
C	-2.315464	-1.525395	3.173854		C	8.109078	-1.849961	-0.68108
H	-3.050423	-3.154834	1.988224		C	6.177001	-2.391735	-3.747096
C	-0.842271	0.022294	2.058076		C	8.455013	-2.086017	-2.944967
H	-0.430408	-0.378082	0.001059		C	8.436485	-1.703722	0.664671
C	-1.521792	-0.381902	3.203532		C	9.107619	-1.867468	-1.669668
H	-2.861326	-1.837635	4.058483		C	6.690385	-2.463395	-5.035925
H	-0.204407	0.899975	2.081478		H	5.117957	-2.475125	-3.560851
H	-1.42285	0.189987	4.121265		C	8.949663	-2.165444	-4.243348
C	-2.787309	-4.634764	-0.465597		C	9.777787	-1.546932	0.991561
C	-4.166637	-4.828968	-0.563871		H	7.676129	-1.705825	1.429627
C	-1.982203	-5.71155	-0.078998		C	10.445444	-1.706771	-1.321693
C	-4.726786	-6.071079	-0.27953		C	8.060569	-2.358433	-5.289081
H	-4.815093	-4.014338	-0.868785		H	6.003444	-2.606125	-5.864105
C	-2.540458	-6.950786	0.210692		H	10.0149	-2.07085	-4.429877
H	-0.904936	-5.584774	-0.009988		C	10.776375	-1.540761	0.014367
C	-3.916365	-7.130895	0.10925		H	10.04855	-1.426854	2.035763
H	-5.799601	-6.202843	-0.365515		H	11.215143	-1.718518	-2.08714
H	-1.902505	-7.776832	0.510536		H	8.427538	-2.423486	-6.308139
H	-4.355641	-8.099125	0.330742		H	11.814245	-1.412752	0.303954
C	3.45424	-2.671135	-0.673442		N	5.543617	-1.519552	0.523394
C	5.628882	-2.087613	-0.676979		N	3.269909	-2.1727	0.548198
C	4.342553	-1.602555	1.102206		N	4.618626	-2.67219	-1.324023
N	4.194359	-1.049576	2.349696		Ge	-1.948996	-2.899331	-0.820182
C	5.119773	-0.18278	2.990412					

Supplementary Table 53. Atomic coordinates of GeCzCz:GeTrzCz2 (12, E = -4202.7626843 a.u.).

C	8.076978	0.221103	0.739752		C	5.967983	-0.575342	-0.317334
C	9.423442	0.110473	0.40875		C	7.148964	1.010611	-1.446432
C	10.411832	0.220505	1.385876		C	5.510342	-1.530652	0.559936
C	10.024679	0.388672	2.715944		C	5.251338	-0.248749	-1.551596
C	8.675384	0.455255	3.055306		C	8.066422	1.94882	-1.820295
C	7.698477	0.391303	2.071033		C	5.984772	0.720515	-2.224754
H	9.688811	-0.05034	-0.631235		C	4.303096	-2.18162	0.25313
H	10.776872	0.475143	3.494393		H	6.068856	-1.796626	1.449007
H	8.382461	0.586848	4.092265		C	4.012406	-0.91015	-1.823121
H	6.647953	0.491627	2.320615		C	7.836291	2.64796	-3.067609
C	13.480353	0.175158	2.346135		H	8.937074	2.183265	-1.220452
C	14.507839	1.103205	2.529858		C	5.778682	1.427776	-3.45515
C	13.33318	-0.854518	3.281787		C	3.562492	-1.8473	-0.930987
C	15.371169	1.003483	3.616138		H	3.939246	-2.984313	0.880961
H	14.634482	1.915017	1.818863		H	3.43496	-0.660445	-2.703976
C	14.194444	-0.957215	4.369104		C	6.723384	2.380574	-3.844915
H	12.534632	-1.58395	3.168621		H	8.560881	3.389532	-3.384495
C	15.215618	-0.027479	4.536094		H	4.904512	1.223759	-4.062815
H	16.165071	1.732719	3.745708		H	6.580261	2.920391	-4.775665
H	14.067691	-1.761796	5.087071		N	2.36107	-2.542525	-1.168632
H	15.88823	-0.105731	5.384741		C	1.330026	-2.719109	-0.242042
C	12.482784	2.032978	-0.120419		C	1.974643	-3.133127	-2.374802
C	13.510797	2.242295	-1.044085		C	1.217651	-2.238952	1.061636
C	11.579076	3.072257	0.119251		C	0.288488	-3.445715	-0.850073
C	13.636488	3.459065	-1.706911		C	2.663794	-3.225503	-3.581694
H	14.214852	1.443481	-1.262431		C	0.698479	-3.70871	-2.212535
C	11.698259	4.288837	-0.545112		C	0.058653	-2.535445	1.761929
H	10.767745	2.931005	0.828246		H	2.000781	-1.637753	1.507819
C	12.728674	4.483157	-1.458898		C	-0.863622	-3.739973	-0.124616
H	14.439745	3.605456	-2.422659		C	2.038112	-3.878713	-4.634389
H	10.986176	5.084909	-0.349541		H	3.655566	-2.805272	-3.695076
H	12.822597	5.431221	-1.97965		C	0.089879	-4.358256	-3.283392
C	12.659734	-1.116142	-0.501621		C	-0.969411	-3.289756	1.181989
C	13.331772	-2.28178	-0.127358		H	-0.062567	-2.162113	2.772992
C	12.22637	-0.989138	-1.825283		H	-1.66753	-4.308991	-0.582858
C	13.555843	-3.300653	-1.04759		C	0.760639	-4.434919	-4.494628
H	13.693174	-2.395891	0.890885		H	2.554413	-3.959268	-5.585827
C	12.445642	-2.00702	-2.747023		H	-0.897142	-4.796149	-3.169214
H	11.723618	-0.081445	-2.149669		H	-1.860646	-3.504977	1.761236
C	13.10978	-3.165402	-2.357657		H	0.297164	-4.933801	-5.339435
H	14.081456	-4.200262	-0.741942		Ge	12.288366	0.307016	0.798935
H	12.101287	-1.894184	-3.770494		C	-6.996581	-1.188396	0.00803
H	13.283586	-3.960356	-3.076427		C	-5.887885	-0.354524	0.169368
N	7.106488	0.198974	-0.288268		C	-4.593923	-0.86811	0.145827

C	-4.425483	-2.241474	-0.064462
C	-5.524791	-3.078892	-0.229268
C	-6.81047	-2.557377	-0.186279
H	-6.05921	0.705011	0.324296
H	-3.4243	-2.660617	-0.104217
H	-5.378021	-4.141804	-0.394494
H	-7.677284	-3.194775	-0.317902
C	-2.380494	-0.034577	2.285889
C	-1.375699	0.76985	2.830657
C	-2.893587	-1.07436	3.063477
C	-0.873034	0.522223	4.10337
H	-0.982235	1.609054	2.262947
C	-2.400642	-1.322439	4.340936
H	-3.686524	-1.703098	2.668682
C	-1.382704	-0.528843	4.859599
H	-0.087837	1.153458	4.508481
H	-2.811556	-2.135461	4.932085
H	-0.994557	-0.722087	5.855087
C	-3.568409	2.191084	0.37541
C	-3.092821	3.020541	-0.642864
C	-4.394472	2.746304	1.358327
C	-3.42894	4.371018	-0.675737
H	-2.44258	2.612233	-1.411512
C	-4.747051	4.090309	1.318548
H	-4.764767	2.126549	2.171317
C	-4.259249	4.90571	0.302934
H	-3.042756	5.005633	-1.468177
H	-5.400692	4.502949	2.080602
H	-4.533619	5.955554	0.27148
C	-1.685714	-0.033669	-0.911161
C	-0.364612	0.380625	-0.729559
C	-2.039032	-0.617389	-2.131077
C	0.57531	0.233947	-1.743391
H	-0.054225	0.816103	0.215646
C	-1.098931	-0.779295	-3.14279
H	-3.058504	-0.955757	-2.294177
C	0.208453	-0.346747	-2.951942
H	1.599115	0.554623	-1.580355
H	-1.382389	-1.251216	-4.07829
H	0.942325	-0.481419	-3.740493
C	-8.357935	-0.614524	0.042404
C	-10.589042	-0.873895	0.197604
C	-9.711113	1.184303	-0.019585
N	-9.870923	2.54862	-0.127644
C	-11.029653	3.283905	0.224531
C	-8.918687	3.451713	-0.668458
C	-12.208364	2.875772	0.843194
C	-10.811531	4.634272	-0.097103
C	-7.647514	3.227016	-1.190694
C	-9.484031	4.738166	-0.670227
C	-13.173069	3.842417	1.100643
H	-12.373089	1.844062	1.11111
C	-11.790563	5.586774	0.167897
C	-6.957884	4.317182	-1.70526
H	-7.206679	2.24316	-1.189259
C	-8.772652	5.819169	-1.182114
C	-12.976307	5.183616	0.763348
H	-14.100041	3.539129	1.576804
H	-11.621932	6.629242	-0.083703
C	-7.504724	5.602478	-1.699393
H	-5.962154	4.158386	-2.103086
H	-9.21085	6.812483	-1.180986
H	-13.751527	5.912692	0.975393
H	-6.934621	6.432837	-2.103578
N	-11.688668	-1.696586	0.32613
C	-11.673416	-3.035129	0.79523
C	-13.032042	-1.342224	0.042464
C	-10.619572	-3.833695	1.233367
C	-12.995961	-3.509222	0.820043
C	-13.569828	-0.174456	-0.493152
C	-13.852007	-2.44431	0.337537
C	-10.920292	-5.114839	1.67937
H	-9.604679	-3.469219	1.222091
C	-13.275618	-4.796842	1.266978
C	-14.94391	-0.126688	-0.694445
H	-12.945192	0.668616	-0.741091
C	-15.226226	-2.376217	0.130062
C	-12.230114	-5.600242	1.694841
H	-10.110442	-5.751193	2.021968
H	-14.298389	-5.160318	1.283238
C	-15.770554	-1.208634	-0.382402
H	-15.37783	0.779112	-1.105816
H	-15.857409	-3.228636	0.36122
H	-12.428559	-6.607574	2.04599
H	-16.840329	-1.136605	-0.54915
N	-10.80751	0.43754	0.109678
N	-8.466476	0.709562	-0.052393
N	-9.387373	-1.452295	0.165982
Ge	-3.044043	0.298636	0.462729

Supplementary Table 54. Atomic coordinates of GeCzCz:GeTrzCz2 (13, E = -4202.7671612 a.u.).

C	-8.736722	-0.325025	-0.906138	C	-6.304281	-0.404157	-0.386639
C	-9.864601	0.143317	-0.239803	C	-7.25058	1.662742	-0.549678
C	-11.130738	-0.36371	-0.523551	C	-6.035327	-1.750997	-0.30989
C	-11.247123	-1.393311	-1.459525	C	-5.273006	0.603712	-0.135638
C	-10.116453	-1.886011	-2.106057	C	-8.109838	2.707452	-0.729284
C	-8.863168	-1.344478	-1.849156	C	-5.865772	1.854831	-0.24479
H	-9.741484	0.931534	0.493786	C	-4.719648	-2.145207	-0.010447
H	-12.223793	-1.800632	-1.704639	H	-6.81201	-2.492901	-0.446978
H	-10.214078	-2.679966	-2.839963	C	-3.938011	0.171455	0.142049
H	-7.988412	-1.690182	-2.388354	C	-7.577798	4.045937	-0.581323
C	-13.080106	-0.387639	2.058797	H	-9.156142	2.571061	-0.969782
C	-13.793899	0.284198	3.055827	C	-5.361463	3.190837	-0.110109
C	-12.678906	-1.704858	2.293718	C	-3.683379	-1.172556	0.195661
C	-14.10742	-0.348349	4.254357	H	-4.479652	-3.191974	0.122615
H	-14.101308	1.315711	2.902719	H	-3.143918	0.891709	0.286433
C	-12.989989	-2.340169	3.49125	C	-6.243634	4.259494	-0.282297
H	-12.111544	-2.239882	1.536745	H	-8.256143	4.882533	-0.701999
C	-13.706277	-1.662303	4.471838	H	-4.315692	3.358299	0.121469
H	-14.661617	0.185502	5.02037	H	-5.87533	5.275063	-0.176323
H	-12.670102	-3.363737	3.660958	N	-2.391998	-1.647331	0.497687
H	-13.948351	-2.156464	5.407773	C	-1.709679	-2.63887	-0.209046
C	-14.216356	0.41231	-0.854559	C	-1.578857	-1.219797	1.552071
C	-15.501691	0.187805	-0.355886	C	-2.079552	-3.311588	-1.37154
C	-14.056792	0.609518	-2.229906	C	-0.464019	-2.86789	0.408677
C	-16.601799	0.168898	-1.206874	C	-1.821103	-0.258444	2.52985
H	-15.647094	0.020149	0.707869	C	-0.380549	-1.961249	1.533673
C	-15.154807	0.5925	-3.083592	C	-1.196415	-4.252698	-1.880774
H	-13.064807	0.768008	-2.645376	H	-3.020343	-3.100916	-1.865891
C	-16.429265	0.373203	-2.571575	C	0.406614	-3.817915	-0.118485
H	-17.594635	-0.008242	-0.804625	C	-0.827364	-0.035229	3.473485
H	-15.015262	0.746813	-4.149286	H	-2.752574	0.293718	2.556233
H	-17.2872	0.35779	-3.236792	C	0.600195	-1.725643	2.49294
C	-12.109744	2.343308	0.669239	C	0.02987	-4.515208	-1.257806
C	-11.384437	2.68203	1.815388	H	-1.463897	-4.794425	-2.782613
C	-12.291123	3.315885	-0.317506	H	1.365279	-4.000661	0.356336
C	-10.837666	3.95277	1.963833	C	0.374203	-0.753614	3.455646
H	-11.238463	1.946005	2.601963	H	-0.990998	0.713374	4.242592
C	-11.749938	4.589381	-0.171085	H	1.526599	-2.288395	2.483715
H	-12.862186	3.080317	-1.21177	H	0.692056	-5.268107	-1.674098
C	-11.017293	4.907007	0.968237	H	1.135779	-0.551514	4.20149
H	-10.268693	4.197189	2.855443	Ge	-12.675195	0.486643	0.354391
H	-11.900813	5.334565	-0.946403	C	6.835999	1.616999	-0.99257
H	-10.59003	5.898623	1.082381	C	5.89821	0.597436	-0.834865
N	-7.482138	0.267235	-0.637803	C	4.603689	0.715878	-1.331722

C	4.24399	1.904624	-1.969679	C	9.332258	-2.506695	0.376327
C	5.163826	2.943529	-2.104611	C	12.222682	-0.937159	1.999904
C	6.460288	2.802291	-1.625646	C	11.167015	-3.100539	1.637239
H	6.203437	-0.297574	-0.305345	C	8.170831	-2.6882	-0.372573
H	3.237852	2.024498	-2.362584	C	9.982168	-3.601971	0.96916
H	4.870596	3.864449	-2.598868	C	13.1946	-1.588771	2.749928
H	7.192098	3.592605	-1.751024	H	12.251714	0.132249	1.865423
C	1.570053	-0.354111	-1.484327	C	12.150473	-3.734014	2.390292
C	0.899549	0.468331	-0.574946	C	7.664394	-3.976567	-0.4903
C	0.895426	-0.773792	-2.632865	H	7.67407	-1.857779	-0.845659
C	-0.40858	0.870422	-0.814	C	9.45937	-4.885633	0.84197
H	1.389871	0.788722	0.340287	C	13.1695	-2.971954	2.941655
C	-0.420215	-0.386894	-2.864826	H	13.989781	-0.999504	3.195453
H	1.395034	-1.417043	-3.350917	H	12.113257	-4.807692	2.546232
C	-1.072449	0.440088	-1.957816	C	8.293669	-5.067691	0.1137
H	-0.912106	1.508029	-0.094078	H	6.760148	-4.125636	-1.071679
H	-0.93769	-0.734854	-3.753393	H	9.963014	-5.728949	1.304387
H	-2.102127	0.735708	-2.132162	H	13.945946	-3.44965	3.530229
C	3.747636	-1.520036	0.67144	H	7.871501	-6.061636	0.006207
C	3.939026	-2.881258	0.919424	N	11.227188	3.121228	-0.017609
C	3.837162	-0.629608	1.744553	C	10.935732	4.508683	0.01722
C	4.192099	-3.344771	2.207049	C	12.633731	2.962815	0.052208
H	3.905852	-3.589783	0.096624	C	9.716736	5.182294	0.004349
C	4.093868	-1.086236	3.032881	C	12.147363	5.211712	0.128484
H	3.720474	0.437678	1.576586	C	13.422219	1.816852	-0.016146
C	4.266686	-2.446606	3.266796	C	13.219329	4.23681	0.141908
H	4.344201	-4.405623	2.380902	C	9.742685	6.568728	0.09184
H	4.163602	-0.379404	3.854242	H	8.785852	4.643764	-0.076618
H	4.470595	-2.805225	4.270971	C	12.151825	6.60073	0.211056
C	4.04858	-2.244	-2.399615	C	14.801446	1.974296	0.044511
C	3.279933	-3.389713	-2.630225	H	12.980968	0.837367	-0.110136
C	5.308689	-2.15711	-2.997483	C	14.602759	4.373562	0.202975
C	3.763025	-4.424447	-3.425053	C	10.942418	7.277735	0.190023
H	2.291683	-3.479115	-2.189322	H	8.801722	7.109681	0.080663
C	5.796752	-3.191773	-3.789871	H	13.089671	7.140881	0.295524
H	5.921241	-1.273191	-2.845487	C	15.392395	3.234774	0.160873
C	5.025141	-4.329338	-4.001901	H	15.428765	1.089794	-0.001424
H	3.152207	-5.306638	-3.593729	H	15.052481	5.358935	0.275356
H	6.781131	-3.109427	-4.240424	H	10.925792	8.36088	0.252664
H	5.405379	-5.138097	-4.618484	H	16.472876	3.322009	0.209301
C	8.214054	1.387964	-0.513599	N	10.698303	0.889119	0.322962
C	10.297834	2.102115	-0.059863	N	8.513079	0.142058	-0.152212
C	9.760906	-0.057433	0.26149	N	9.069078	2.409313	-0.483609
N	10.091276	-1.336671	0.652614	Ge	3.432288	-0.853255	-1.155834
C	11.214846	-1.711273	1.431042				

Supplementary Table 55. Atomic coordinates of GeCzCz:GeTrzCz2 (14, E = -4202.7980205 a.u.).

C	-5.462611	0.783349	-0.272193		C	-3.00506	0.787059	-0.684887
C	-6.499029	-0.11319	-0.513236		C	-3.806154	-0.788024	0.752496
C	-7.819842	0.324354	-0.602327		C	-2.83023	1.772035	-1.624124
C	-8.083651	1.690749	-0.502577		C	-1.882295	-0.004695	-0.188367
C	-7.041636	2.595302	-0.311653		C	-4.591129	-1.562205	1.555175
C	-5.734199	2.148628	-0.178191		C	-2.386163	-0.967826	0.670944
H	-6.258123	-1.166052	-0.615378		C	-1.519282	2.035263	-2.069419
H	-9.104817	2.054368	-0.566922		H	-3.668173	2.322245	-2.033594
H	-7.25325	3.657717	-0.236979		C	-0.555472	0.329714	-0.607538
H	-4.924391	2.83875	0.025643		C	-3.94598	-2.615791	2.306368
C	-10.971909	-0.183475	-0.993803		H	-5.660265	-1.415099	1.642374
C	-12.059286	-0.47334	-0.166485		C	-1.770246	-2.020703	1.427441
C	-11.155007	0.720017	-2.046357		C	-0.393388	1.337819	-1.51902
C	-13.29911	0.118297	-0.386303		H	-1.351572	2.746407	-2.86705
H	-11.937309	-1.166511	0.661337		H	0.292513	-0.176637	-0.168681
C	-12.392985	1.313636	-2.268964		C	-2.576971	-2.824237	2.226827
H	-10.323038	0.971653	-2.699794		H	-4.559593	-3.246399	2.939985
C	-13.467162	1.011729	-1.438312		H	-0.69785	-2.166205	1.37692
H	-14.134644	-0.117066	0.265941		H	-2.124232	-3.619737	2.809632
H	-12.518997	2.013051	-3.08986		N	0.896005	1.704028	-1.95949
H	-14.43406	1.474712	-1.610169		C	1.361095	3.013932	-2.127712
C	-9.180739	-1.978591	1.033945		C	1.940313	0.825159	-2.270359
C	-9.689931	-3.273809	1.163131		C	0.723336	4.229642	-1.880305
C	-8.600685	-1.375154	2.153683		C	2.699512	2.971484	-2.567696
C	-9.625521	-3.94733	2.378868		C	1.967215	-0.568769	-2.272519
H	-10.128072	-3.772204	0.302295		C	3.068988	1.575369	-2.658222
C	-8.529079	-2.047134	3.369747		C	1.441536	5.395698	-2.10332
H	-8.190948	-0.371807	2.07537		H	-0.295319	4.265726	-1.516808
C	-9.04266	-3.334787	3.483115		C	3.399566	4.155219	-2.790535
H	-10.023614	-4.954091	2.462168		C	3.149071	-1.192253	-2.645167
H	-8.071272	-1.565723	4.228718		H	1.095584	-1.150132	-2.002067
H	-8.986026	-3.861867	4.430685		C	4.243189	0.926083	-3.032812
C	-8.845354	-2.346222	-2.10341		C	2.76331	5.364923	-2.560938
C	-9.500936	-2.299097	-3.335611		H	0.969594	6.351755	-1.901197
C	-7.875968	-3.333417	-1.899077		H	4.43604	4.126801	-3.111262
C	-9.18994	-3.206756	-4.342889		C	4.282473	-0.459235	-3.017072
H	-10.269593	-1.551881	-3.512172		H	3.186344	-2.277217	-2.648678
C	-7.560466	-4.240626	-2.90452		H	5.115042	1.501855	-3.324386
H	-7.367019	-3.40862	-0.941415		H	3.300589	6.295096	-2.710441
C	-8.216831	-4.17665	-4.129141		H	5.190691	-0.980036	-3.299595
H	-9.709792	-3.157984	-5.294957		Ge	-9.235756	-1.037799	-0.691564
H	-6.803867	-4.999707	-2.730739		C	3.630976	-1.256218	1.18572
H	-7.972687	-4.885167	-4.914787		C	4.937212	-1.375067	0.708446
N	-4.150783	0.295371	-0.089057		C	5.552917	-2.619057	0.59902

C	4.829545	-3.756842	0.970542		C	5.014083	3.480139	-0.001204
C	3.520038	-3.651137	1.427369		C	2.268089	5.303144	1.611717
C	2.920918	-2.404095	1.538936		C	4.352947	5.646509	0.411978
H	5.469819	-0.472289	0.438228		C	5.792182	2.4522	-0.523404
H	5.29359	-4.737959	0.909985		C	5.382119	4.822918	-0.187095
H	2.96775	-4.54301	1.706269		C	2.137761	6.684987	1.686583
H	1.90526	-2.303267	1.903354		H	1.528145	4.655363	2.050543
C	8.36593	-4.123975	0.97588		C	4.206554	7.026431	0.4977
C	9.135531	-3.745914	2.078609		C	6.939551	2.795847	-1.227884
C	8.279593	-5.48158	0.654469		H	5.50318	1.422857	-0.407989
C	9.7934	-4.699561	2.848108		C	6.538269	5.149824	-0.887541
H	9.232414	-2.694854	2.336937		C	3.087315	7.544764	1.131449
C	8.934162	-6.438719	1.423045		H	1.271126	7.097585	2.193451
H	7.707002	-5.798247	-0.213641		H	4.95939	7.683561	0.073227
C	9.690786	-6.04785	2.522412		C	7.31833	4.127555	-1.408624
H	10.389758	-4.389867	3.701072		H	7.553557	1.999583	-1.633481
H	8.85795	-7.489651	1.160472		H	6.815388	6.189732	-1.030936
H	10.204649	-6.793112	3.121989		H	2.953021	8.61915	1.204465
C	8.304443	-1.056479	0.032675		H	8.223696	4.361687	-1.959544
C	9.14848	-0.623322	-0.993517		N	-0.030425	1.447621	2.438541
C	8.184689	-0.259884	1.176451		C	-0.632751	0.530111	3.327624
C	9.868014	0.561636	-0.872776		C	-0.985832	2.429347	2.097786
H	9.248399	-1.216501	-1.898172		C	-0.085239	-0.554159	4.008071
C	8.893462	0.930888	1.295756		C	-1.965686	0.923197	3.536803
H	7.522972	-0.562919	1.983825		C	-0.885053	3.520881	1.242857
C	9.741694	1.339604	0.273068		C	-2.194423	2.115481	2.74441
H	10.524073	0.880565	-1.677348		C	-0.907856	-1.244595	4.888073
H	8.778245	1.54278	2.184772		H	0.941676	-0.849919	3.851315
H	10.293473	2.269954	0.364002		C	-2.778044	0.206495	4.409497
C	7.318721	-3.433385	-1.938613		C	-2.015697	4.307249	1.064287
C	8.478509	-3.843546	-2.603899		H	0.038346	3.750331	0.735325
C	6.102387	-3.49486	-2.621073		C	-3.31752	2.913471	2.548555
C	8.425406	-4.280347	-3.923369		C	-2.242892	-0.880221	5.083226
H	9.4351	-3.832759	-2.087304		H	-0.501116	-2.09344	5.428812
C	6.043519	-3.932168	-3.940783		H	-3.811978	0.499663	4.56071
H	5.189734	-3.198369	-2.113781		C	-3.219578	4.016561	1.712049
C	7.207328	-4.320856	-4.594666		H	-1.954075	5.168785	0.406519
H	9.334786	-4.592982	-4.427655		H	-4.254867	2.67138	3.039569
H	5.089092	-3.96825	-4.457524		H	-2.862366	-1.451081	5.766973
H	7.165325	-4.66092	-5.624885		H	-4.082409	4.657724	1.560376
C	3.007201	0.077119	1.325668		N	1.846867	2.470092	1.535482
C	1.24838	1.34861	1.922614		N	3.70182	1.139353	0.925807
C	3.084713	2.315557	1.057969		N	1.780691	0.128616	1.849992
N	3.779347	3.447996	0.701024		Ge	7.388578	-2.78806	-0.086356
C	3.378899	4.788613	0.948892					

Supplementary Table 56. Atomic coordinates of GeCzCz:GeTrzCz2 (15, E = -4202.8092433 a.u.).

C	-3.534268	0.182641	1.949472		C	-1.320642	1.244836	1.638131
C	-4.552655	-0.312814	1.140383		C	-1.426924	-0.995082	1.267394
C	-5.867157	-0.366937	1.617684		C	-1.580039	2.574017	1.877786
C	-6.142176	0.115333	2.898295		C	0.040941	0.741828	1.463764
C	-5.127587	0.639395	3.693808		C	-1.84541	-2.275207	1.065126
C	-3.822965	0.661431	3.227986		C	-0.043272	-0.630368	1.258071
H	-4.306472	-0.651629	0.138982		C	-0.490998	3.454967	1.934411
H	-7.156843	0.077675	3.283383		H	-2.592193	2.935731	2.010894
H	-5.351172	1.010361	4.689143		C	1.1291	1.668111	1.495576
H	-3.016232	1.037244	3.847651		C	-0.82941	-3.286902	0.847838
C	-7.801317	0.307907	-0.817504		H	-2.893693	-2.541879	1.050007
C	-8.196429	0.005112	-2.122432		C	0.944546	-1.654313	1.077106
C	-7.779142	1.649843	-0.42502		C	0.851398	2.991381	1.717111
C	-8.557799	1.012461	-3.012235		H	-0.646226	4.496032	2.182942
H	-8.214825	-1.026899	-2.459504		H	2.142106	1.337706	1.307326
C	-8.157438	2.6594	-1.304217		C	0.515204	-2.968211	0.868601
H	-7.449215	1.916302	0.575777		H	-1.146391	-4.308293	0.66924
C	-8.54391	2.341804	-2.601967		H	1.998076	-1.407974	1.108449
H	-8.852669	0.758968	-4.025892		H	1.253346	-3.746186	0.700939
H	-8.137379	3.695485	-0.980019		N	1.885387	3.946548	1.768701
H	-8.829154	3.127994	-3.294002		C	3.089423	3.830204	2.473612
C	-8.91304	-1.308357	1.686136		C	1.836558	5.21913	1.190645
C	-9.060461	-2.49737	2.406227		C	3.579463	2.774878	3.239165
C	-9.879853	-0.311415	1.836806		C	3.805171	5.038917	2.353925
C	-10.140255	-2.682516	3.262327		C	0.87069	5.780195	0.356782
H	-8.328469	-3.292876	2.29312		C	3.00673	5.92377	1.533021
C	-10.961531	-0.492513	2.693114		C	4.805263	2.947057	3.865603
H	-9.795979	0.615391	1.275757		H	3.029384	1.848238	3.342132
C	-11.091797	-1.678199	3.407697		C	5.036267	5.185601	2.987357
H	-10.241327	-3.61205	3.814414		C	1.076293	7.076655	-0.088073
H	-11.705798	0.29113	2.798695		H	-0.000722	5.218103	0.045836
H	-11.93655	-1.821501	4.074654		C	3.197268	7.222101	1.064529
C	-6.886749	-2.818289	-0.218596		C	5.53479	4.134641	3.740725
C	-7.736047	-3.468624	-1.11996		H	5.205637	2.138372	4.467937
C	-5.738582	-3.487973	0.208772		H	5.592974	6.113247	2.893974
C	-7.429763	-4.736412	-1.602878		C	2.22319	7.798282	0.264248
H	-8.659512	-2.994349	-1.44124		H	0.340634	7.529243	-0.744394
C	-5.430759	-4.758861	-0.264796		H	4.096854	7.772085	1.324696
H	-5.074048	-3.014807	0.924492		H	6.492905	4.232371	4.240864
C	-6.272649	-5.382509	-1.17784		H	2.355539	8.809636	-0.10633
H	-8.099542	-5.225001	-2.304061		Ge	-7.346437	-1.061315	0.51996
H	-4.530149	-5.259499	0.077508		C	2.428709	-2.208511	-1.848464
H	-6.034027	-6.373427	-1.55189		C	3.689391	-2.221733	-1.260147
N	-2.183932	0.177171	1.509341		C	4.402499	-3.403141	-1.081009

C	3.838268	-4.592003	-1.546331		C	3.705496	2.613515	-1.013948
C	2.590963	-4.586893	-2.170938		C	0.974177	4.090089	-2.948618
C	1.878226	-3.403267	-2.311312		C	3.163201	4.615056	-2.012142
H	4.084746	-1.278883	-0.904738		C	4.435184	1.712473	-0.242385
H	4.365486	-5.532671	-1.413201		C	4.171415	3.920458	-1.235021
H	2.165126	-5.517447	-2.533349		C	0.944438	5.375661	-3.47678
H	0.896014	-3.394797	-2.769279		H	0.155669	3.404153	-3.106076
C	6.741155	-5.062744	0.380395		C	3.113014	5.89564	-2.5505
C	6.812305	-5.494524	1.70674		C	5.647394	2.140045	0.284411
C	7.138983	-5.942984	-0.631954		H	4.073486	0.713394	-0.051798
C	7.269636	-6.77157	2.016209		C	5.391774	4.327617	-0.704558
H	6.506724	-4.826807	2.507572		C	1.997957	6.272203	-3.284118
C	7.596781	-7.220227	-0.326355		H	0.077931	5.682222	-4.054341
H	7.095922	-5.631412	-1.673036		H	3.932375	6.588651	-2.390554
C	7.662296	-7.635276	0.999861		C	6.13004	3.430307	0.051642
H	7.319886	-7.092517	3.052274		H	6.217116	1.452269	0.899482
H	7.902952	-7.891623	-1.122864		H	5.745266	5.341213	-0.865406
H	8.019594	-8.631948	1.240286		H	1.94122	7.269641	-3.707926
C	5.567853	-2.268645	1.564595		H	7.075043	3.736785	0.486875
C	6.399826	-1.279358	2.095144		N	-1.609242	0.249017	-2.05104
C	4.325995	-2.48709	2.168416		C	-2.449733	-0.858272	-2.344901
C	6.000418	-0.52627	3.19532		C	-2.427296	1.405578	-1.936069
H	7.363866	-1.081312	1.635047		C	-2.167707	-2.218236	-2.4526
C	3.921263	-1.737465	3.268343		C	-3.763328	-0.38403	-2.499658
H	3.652186	-3.239367	1.766905		C	-2.120581	2.706325	-1.543293
C	4.75994	-0.753911	3.781834		C	-3.749909	1.041117	-2.237564
H	6.653907	0.246967	3.587401		C	-3.220858	-3.077326	-2.743677
H	2.946619	-1.913878	3.712879		H	-1.169748	-2.587796	-2.287796
H	4.444238	-0.164898	4.637761		C	-4.801774	-1.259418	-2.802608
C	7.437759	-2.216087	-0.992812		C	-3.158795	3.629813	-1.494655
C	8.700624	-2.738867	-1.277646		H	-1.117072	2.985817	-1.269063
C	7.168056	-0.889375	-1.347084		C	-4.773806	1.980622	-2.196246
C	9.668207	-1.959161	-1.90526		C	-4.523459	-2.611137	-2.929116
H	8.937983	-3.762974	-1.00362		H	-3.020639	-4.141708	-2.817249
C	8.133642	-0.105455	-1.966404		H	-5.8114	-0.883666	-2.921166
H	6.20123	-0.444187	-1.133535		C	-4.469898	3.281571	-1.825316
C	9.386207	-0.641963	-2.24869		H	-2.934048	4.645919	-1.184958
H	10.645603	-2.380467	-2.120767		H	-5.789914	1.694784	-2.439298
H	7.904782	0.92473	-2.221718		H	-5.324343	-3.311236	-3.138866
H	10.142891	-0.032181	-2.733121		H	-5.258459	4.025712	-1.779638
C	1.688424	-0.930149	-1.881861		N	0.394284	1.398555	-1.873194
C	-0.234297	0.228325	-2.002824		N	2.402415	0.176606	-1.669522
C	1.712048	1.312585	-1.720588		N	0.374225	-0.957797	-2.082618
N	2.430779	2.487031	-1.611362		Ge	6.075883	-3.273241	-0.051857
C	2.094805	3.722141	-2.210845					

Supplementary Table 57. Atomic coordinates of GeCzCz:GeTrzCz2 (16, E = -4202.8127934 a.u.).

C	-0.372082	-2.405946	-2.699994		C	2.035031	-1.827602	-2.467724
C	-1.43416	-2.240012	-1.814255		C	0.634508	-0.122816	-2.955223
C	-2.48842	-3.157102	-1.790116		C	2.57389	-3.059186	-2.102951
C	-2.445608	-4.243607	-2.668042		C	2.833018	-0.669721	-2.583456
C	-1.385641	-4.408834	-3.553133		C	-0.479867	0.658263	-3.251391
C	-0.348934	-3.487681	-3.577109		C	1.934425	0.423535	-2.899902
H	-1.410025	-1.388841	-1.138738		C	3.93165	-3.114003	-1.844036
H	-3.248985	-4.973227	-2.659636		H	1.950782	-3.941346	-2.012226
H	-1.370556	-5.255544	-4.232112		C	4.198603	-0.74583	-2.316024
H	0.480311	-3.592799	-4.268599		C	-0.271785	2.008726	-3.484416
C	-5.151918	-4.582062	-0.815689		H	-1.470989	0.222852	-3.31335
C	-6.152056	-4.575808	-1.792769		C	2.115024	1.787739	-3.129626
C	-4.941824	-5.756465	-0.087201		C	4.735503	-1.968073	-1.937182
C	-6.922669	-5.708549	-2.033963		H	4.394348	-4.049668	-1.549936
H	-6.339301	-3.674211	-2.369407		H	4.836502	0.128239	-2.367557
C	-5.710259	-6.891435	-0.324743		C	1.010726	2.571478	-3.422377
H	-4.174933	-5.785372	0.68152		H	-1.12298	2.638629	-3.719292
C	-6.702471	-6.868415	-1.298886		H	3.107092	2.224456	-3.082529
H	-7.696806	-5.684928	-2.795059		H	1.137431	3.634095	-3.595885
H	-5.535087	-7.794346	0.252491		N	6.108346	-2.071318	-1.594939
H	-7.30386	-7.753206	-1.484686		C	7.190883	-1.87224	-2.440256
C	-5.073656	-1.418895	-0.98919		C	6.585633	-2.395418	-0.330056
C	-6.422717	-1.367275	-0.625471		C	7.202869	-1.501615	-3.782136
C	-4.534469	-0.343922	-1.699259		C	8.383984	-2.096073	-1.719575
C	-7.209414	-0.270622	-0.959476		C	5.870824	-2.60672	0.84632
H	-6.869866	-2.193959	-0.080164		C	7.994924	-2.432816	-0.365865
C	-5.318342	0.754718	-2.036805		C	8.437359	-1.338236	-4.392399
H	-3.491948	-0.361375	-2.0036		H	6.277895	-1.337167	-4.323461
C	-6.657872	0.793452	-1.664935		C	9.612068	-1.92584	-2.356808
H	-8.255537	-0.24335	-0.668881		C	6.599393	-2.860595	1.998251
H	-4.885786	1.581582	-2.59178		H	4.788853	-2.55185	0.866019
H	-7.267769	1.653803	-1.917715		C	8.70298	-2.696295	0.806646
C	-3.342658	-3.081879	1.30966		C	9.633389	-1.543946	-3.69019
C	-4.218095	-3.454676	2.334479		H	8.476773	-1.044494	-5.436799
C	-1.998983	-2.870542	1.628459		H	10.539376	-2.087905	-1.814979
C	-3.75735	-3.646416	3.631593		C	8.000898	-2.908511	1.983819
H	-5.270622	-3.616808	2.11933		H	6.066716	-3.017089	2.930868
C	-1.537337	-3.042562	2.929079		H	9.788786	-2.724936	0.799013
H	-1.29232	-2.577648	0.858691		H	10.582706	-1.406911	-4.197745
C	-2.41378	-3.448678	3.928028		H	8.539418	-3.110612	2.904064
H	-4.451074	-3.943735	4.411842		Ge	-4.005898	-3.005762	-0.540272
H	-0.496103	-2.846317	3.162267		C	0.360895	3.338407	-0.022232
H	-2.051375	-3.587777	4.941184		C	1.38542	2.536498	0.476505
N	0.707514	-1.48949	-2.698999		C	2.718035	2.923208	0.395272

C	3.009049	4.157827	-0.196947		C	-1.883629	0.395667	3.342915
C	1.996415	4.956027	-0.72039		C	-5.284543	0.36382	2.050173
C	0.671001	4.548933	-0.638038		C	-3.988544	-0.193647	4.061868
H	1.111765	1.597754	0.938017		C	-0.522742	0.450623	3.397922
H	4.035295	4.511603	-0.24454		C	-2.662023	-0.174916	4.430282
H	2.241057	5.909563	-1.17808		C	-6.429793	-0.173528	2.708104
H	-0.131258	5.171231	-1.018123		H	-5.363698	0.742453	1.044351
C	5.676629	1.672105	0.079373		C	-5.170759	-0.705229	4.70925
C	5.712837	2.255598	-1.189596		C	0.123999	-0.037916	4.571983
C	6.802034	0.968185	0.518671		H	0.058678	0.849275	2.585108
C	6.834947	2.129296	-2.003861		C	-1.979061	-0.639066	5.613057
H	4.854939	2.807656	-1.560984		C	-6.356987	-0.683924	4.021805
C	7.931344	0.857449	-0.284282		H	-7.373899	-0.17789	2.176223
H	6.801106	0.483673	1.491483		H	-5.104247	-1.107961	5.7136
C	7.946019	1.426567	-1.552526		C	-0.611444	-0.561913	5.656902
H	6.838396	2.575138	-2.994129		H	1.206004	-0.002407	4.616136
H	8.789717	0.299436	0.071143		H	-2.551561	-1.044527	6.439755
H	8.813772	1.302842	-2.1923		H	-7.257621	-1.071187	4.488215
C	3.336927	0.056362	1.706109		H	-0.078488	-0.91187	6.535658
C	3.668304	-0.551394	2.921302		N	-4.266095	3.803992	-0.89471
C	2.474301	-0.622445	0.840421		C	-4.145304	4.401632	-2.172196
C	3.155051	-1.800066	3.260385		C	-5.633858	3.79546	-0.538764
H	4.331398	-0.045077	3.616738		C	-3.021339	4.64853	-2.95845
C	1.951707	-1.866991	1.178027		C	-5.433537	4.729351	-2.631825
H	2.201696	-0.181405	-0.111307		C	-6.258138	3.381276	0.634213
C	2.295111	-2.460452	2.388395		C	-6.373365	4.3552	-1.594257
H	3.42505	-2.256874	4.208052		C	-3.218452	5.216291	-4.211092
H	1.286016	-2.375974	0.488311		H	-2.033344	4.417463	-2.593241
H	1.894331	-3.43513	2.649992		C	-5.608093	5.302546	-3.888514
C	4.622027	2.663012	2.94037		C	-7.639608	3.497309	0.707505
C	5.909239	3.15413	3.161983		H	-5.691683	2.964332	1.451626
C	3.66864	2.802719	3.954717		C	-7.758827	4.464602	-1.501779
C	6.240166	3.76479	4.368715		C	-4.494623	5.542355	-4.678348
H	6.665591	3.057608	2.388397		H	-2.353218	5.416625	-4.835311
C	3.994701	3.410968	5.160791		H	-6.603443	5.553961	-4.241477
H	2.656945	2.431421	3.806309		C	-8.390218	4.023183	-0.348673
C	5.283958	3.892605	5.369052		H	-8.142251	3.167105	1.611052
H	7.246774	4.140258	4.526861		H	-8.330567	4.893818	-2.318791
H	3.24323	3.510996	5.938226		H	-4.612242	5.987957	-5.660612
H	5.541152	4.368344	6.310642		H	-9.469163	4.097152	-0.259665
C	-1.040497	2.922003	0.180657		N	-3.575203	2.279	0.712622
C	-3.241108	3.18582	-0.198117		N	-1.258902	1.962883	1.076373
C	-2.541915	1.701477	1.336471		N	-1.995625	3.562668	-0.494691
N	-2.808981	0.804184	2.337431		Ge	4.103357	1.813361	1.242547
C	-4.106119	0.373297	2.732173					

Supplementary References

1. Xue, Z., Hu, Y., Xiao, S., Liu, J., Miao, J. & Yang, C. Cyano-Modified Multi-Resonance Thermally Activated Delayed Fluorescent Emitters Towards Pure-Green OLEDs with a CIE_y Value of 0.74. *Angew. Chem. Int. Ed.* **64**, e202500108 (2025).
2. Yang, M. et al. Wide-Range Color Tuning of Narrowband Emission in Multi-resonance Organoboron Delayed Fluorescence Materials through Rational Imine/Amine Functionalization. *Angew. Chem. Int. Ed.* **60**, 23142-23147 (2021).
3. Luo, X.-F. et al. Multiple-Resonance-Induced Thermally Activated Delayed Fluorescence Materials Based on Indolo[3,2,1-*jk*]carbazole with an Efficient Narrowband Pure-Green Electroluminescence. *Angew. Chem. Int. Ed.* **61**, e202209984 (2022).
4. Zhang, Y. et al. Fusion of Multi-Resonance Fragment with Conventional Polycyclic Aromatic Hydrocarbon for Nearly BT.2020 Green Emission. *Angew. Chem. Int. Ed.* **61**, e202202380 (2022).
5. Liu, J. et al. Toward a BT.2020 green emitter through a combined multiple resonance effect and multi-lock strategy. *Nat. Commun.* **13**, 4876 (2022).
6. Uemura, S. et al. Sequential Multiple Borylation Toward an Ultrapure Green Thermally Activated Delayed Fluorescence Material. *J. Am. Chem. Soc.* **145**, 1505-1511 (2023).
7. Lee, Y.-T. et al. Bright, efficient, and stable pure-green hyperfluorescent organic light-emitting diodes by judicious molecular design. *Nat. Commun.* **15**, 3174 (2024).
8. Fan, X.-C. et al. Ultrapure green organic light-emitting diodes based on highly distorted fused π-conjugated molecular design. *Nat. Photon.* **17**, 280-285 (2023).
9. Wang, Q. et al. Precise Regulation of Emission Maxima and Construction of Highly Efficient Electroluminescent Materials with High Color Purity. *Angew. Chem. Int. Ed.* **62**, e202301930 (2023).
10. Xiao, S. et al. Synergistic π-Extension and Peripheral-Locking of B/N-Based Multi-Resonance Framework Enables High-Performance Pure-Green Organic Light-Emitting Diodes. *Angew. Chem. Int. Ed.* **64**, e202418348 (2025).
11. Kondo, Y. et al. Narrowband deep-blue organic light-emitting diode featuring an organoboron-based emitter. *Nat. Photon.* **13**, 678-682 (2019).
12. Huang, T. et al. Enhancing the efficiency and stability of blue thermally activated delayed fluorescence emitters by perdeuteration. *Nat. Photon.* **18**, 516-523 (2024).
13. Huang, T. et al. Delocalizing electron distribution in thermally activated delayed fluorophors for high-efficiency and long-lifetime blue electroluminescence. *Nat. Mater.* **23**, 1523-1530 (2024).
14. Chan, C.-Y. et al. Stable pure-blue hyperfluorescence organic light-emitting diodes with high-efficiency and narrow emission. *Nat. Photon.* **15**, 203-207 (2021).
15. Jeon, S. O. et al. High-efficiency, long-lifetime deep-blue organic light-emitting diodes. *Nat. Photon.* **15**, 208-215 (2021).
16. Sun, J. et al. Exceptionally stable blue phosphorescent organic light-emitting diodes. *Nat. Photon.* **16**, 212-218 (2022).
17. Kim, E. et al. Highly efficient and stable deep-blue organic light-emitting diode using phosphor-sensitized thermally activated delayed fluorescence. *Sci. Adv.* **8**, eabq1641 (2022).
18. Jung, Y. H. et al. Modified t-butyl in tetradentate platinum (II) complexes enables exceptional lifetime for blue-phosphorescent organic light-emitting diodes. *Nat. Commun.* **15**, 2977 (2024).

19. Fleetham, T., Li, G., Wen, L. & Li, J. Efficient "Pure" Blue OLEDs Employing Tetradentate Pt Complexes with a Narrow Spectral Bandwidth. *Adv. Mater.* **26**, 7116-7121 (2014).
20. Wu, Y. et al. Iridium(III) Blue Phosphors with Heteroleptic Carbene Cyclometalates: Isomerization, Emission Tuning, and OLED Fabrications. *Angew. Chem. Int. Ed.* **64**, e202421664 (2025).
21. Yan, J. et al. Blue Electrophosphorescence from Iridium(III) Phosphors Bearing Asymmetric Di-N-aryl 6-(trifluoromethyl)-2H-imidazo[4,5-b]pyridin-2-ylidene Chelates. *Adv. Mater.* **36**, 2305273 (2024).
