# Supplementary material for: Germanium-integrated exciplex host for high-performance narrowband OLEDs with mitigated efficiency roll-off
Source: Nat Commun. 2026 Aug 20;17:9942. doi: 10.1038/s41467-026-76914-5 (PMC13586258; doi:10.1038/s41467-026-76914-5)
Supplement: Supplementary file 2 [file 41467_2026_76914_MOESM2_ESM.pdf]

**Description of Additional Supplementary Files**

File Name: Supplementary Data 1
Description: CheckCIF report of SiTrzCz2.

File Name: Supplementary Data 2
Description: CheckCIF report of SiCzCz.

File Name: Supplementary Data 3
Description: CheckCIF report of GeCzCz.

File Name: Supplementary Data 4
Description: CheckCIF report of GeTrzCz2.
