# Supplementary material for: Germanium-integrated exciplex host for high-performance narrowband OLEDs with mitigated efficiency roll-off
Source: Nat Commun. 2026 Aug 20;17:9942. doi: 10.1038/s41467-026-76914-5 (PMC13586258; doi:10.1038/s41467-026-76914-5)

# checkCIF/PLATON report

You have not supplied any structure factors. As a result the full set of tests cannot be run.

THIS REPORT IS FOR GUIDANCE ONLY. IF USED AS PART OF A REVIEW PROCEDURE FOR PUBLICATION, IT SHOULD NOT REPLACE THE EXPERTISE OF AN EXPERIENCED CRYSTALLOGRAPHIC REFEREE.

No syntax errors found. CIF dictionary Interpreting this report

## Datablock: 241028_ge_15

---

```
Bond precision:   C-C = 0.0029 A          Wavelength=1.34139

Cell:           a=12.3966(5)      b=14.1769(6)      c=23.0035(9)
                alpha=93.094(1)   beta=101.477(1)   gamma=112.927(1)
Temperature:    170 K

                 Calculated                    Reported
Volume           3610.0(3)                     3610.0(3)
Space group      P -1                          P -1
Hall group       -P 1                          -P 1
Moiety formula   C48 H34 Ge N2                 C48 H34 Ge N2
Sum formula      C48 H34 Ge N2                 C48 H34 Ge N2
Mr               711.38                        711.36
Dx,g cm-3        1.309                         1.309
Z                4                             4
Mu (mm-1)        0.934                         0.934
F000             1472.0                        1472.0
F000'            1468.46
h,k,lmax         16,18,29                      16,18,29
Nref             16587                         16482
Tmin,Tmax        0.914,0.954                   0.680,0.752
Tmin'            0.911

Correction method= # Reported T Limits: Tmin=0.680 Tmax=0.752
AbsCorr = MULTI-SCAN

Data completeness= 0.994           Theta(max)= 60.667

                                                   wR2(reflections)=
R(reflections)= 0.0342( 14084)                     0.0908( 16482)

S = 1.030                    Npar= 919
```

The following ALERTS were generated. Each ALERT has the format
**test-name_ALERT_alert-type_alert-level.**
Click on the hyperlinks for more details of the test.

### Alert level C

```
PLAT213_ALERT_2_C Atom C3              has ADP max/min Ratio .....        3.1 prolat
PLAT220_ALERT_2_C NonSolvent   Resd 1  C   Ueq(max)/Ueq(min) Range        4.1 Ratio
PLAT230_ALERT_2_C Hirshfeld Test Diff for    C94       --C95      .       6.3 s.u.
PLAT241_ALERT_2_C High   'MainMol' Ueq as Compared to Neighbors of         C3 Check
PLAT241_ALERT_2_C High   'MainMol' Ueq as Compared to Neighbors of        C10 Check
PLAT601_ALERT_2_C Unit Cell Contains Solvent Accessible VOIDS of .         36 Ang**3
```

### Alert level G

```
ABSMU01_ALERT_1_G  Calculation of _exptl_absorpt_correction_mu
                not performed for this radiation type.
PLAT003_ALERT_2_G Number of Uiso or U(i,j) Restrained non-H Atoms          12 Report
PLAT012_ALERT_1_G No       _shelx_res_checksum Found in CIF ......     Please Check
PLAT154_ALERT_1_G The s.u.'s on the Cell Angles are Equal ..(Note)      0.001 Degree
PLAT177_ALERT_4_G The CIF-Embedded .res File Contains DELU Records          1 Report
PLAT192_ALERT_3_G A Non-default DELU Restraint Value for First Par     0.0010 Report
PLAT192_ALERT_3_G A Non-default DELU Restraint Value for SecondPar     0.0020 Report
PLAT790_ALERT_4_G Centre of Gravity not Within Unit Cell: Resd.  #          2 Note
              C48 H34 Ge N2
PLAT860_ALERT_3_G Number of Least-Squares Restraints ............          29 Note
PLAT933_ALERT_2_G Number of HKL-OMIT Records in Embedded .res File         31 Note
                0  0  1,   1  0  3,   0 -1  3,   0  1  0,  -6  1  4,  -5  5  2,
               -3  1  8,  -2  1  8,   5  2  3,   2  4  3,  -2  3  6,   2 -1 13,
               -2  6  5,  -3  4  0,  -2 -2 12,   0 -3 11,   2 -7  9,  -3 -5  4,
                4 -1  6,  -6  4  4,   3  0 10,  -1  8  4,  -2  7  3,  -5  1  7,
               -2 -4  5,   5 -5  4,  -4  1 14,  -4  3  7,   1  1 16,   2  0 10,
               -5  2  8,
```

0 **ALERT level A** = Most likely a serious problem - resolve or explain
0 **ALERT level B** = A potentially serious problem, consider carefully
6 **ALERT level C** = Check. Ensure it is not caused by an omission or oversight
10 **ALERT level G** = General information/check it is not something unexpected

3 ALERT type 1 CIF construction/syntax error, inconsistent or missing data
8 ALERT type 2 Indicator that the structure model may be wrong or deficient
3 ALERT type 3 Indicator that the structure quality may be low
2 ALERT type 4 Improvement, methodology, query or suggestion
0 ALERT type 5 Informative message, check

It is advisable to attempt to resolve as many as possible of the alerts in all categories. Often the minor alerts point to easily fixed oversights, errors and omissions in your CIF or refinement strategy, so attention to these fine details can be worthwhile. In order to resolve some of the more serious problems it may be necessary to carry out additional measurements or structure refinements. However, the purpose of your study may justify the reported deviations and the more serious of these should normally be commented upon in the discussion or experimental section of a paper or in the "special_details" fields of the CIF. checkCIF was carefully designed to identify outliers and unusual parameters, but every test has its limitations and alerts that are not important in a particular case may appear. Conversely, the absence of alerts does not guarantee there are no aspects of the results needing attention. It is up to the individual to critically assess their own results and, if necessary, seek expert advice.

**Publication of your CIF in IUCr journals**

A basic structural check has been run on your CIF. These basic checks will be run on all CIFs submitted for publication in IUCr journals (*Acta Crystallographica*, *Journal of Applied Crystallography*, *Journal of Synchrotron Radiation*); however, if you intend to submit to *Acta Crystallographica Section C* or *E* or *IUCrData*, you should make sure that full publication checks are run on the final version of your CIF prior to submission.

**Publication of your CIF in other journals**

Please refer to the *Notes for Authors* of the relevant journal for any special instructions relating to CIF submission.

---

**PLATON version of 22/08/2024; check.def file version of 21/08/2024**

**Datablock 241028_ge_15** - ellipsoid plot

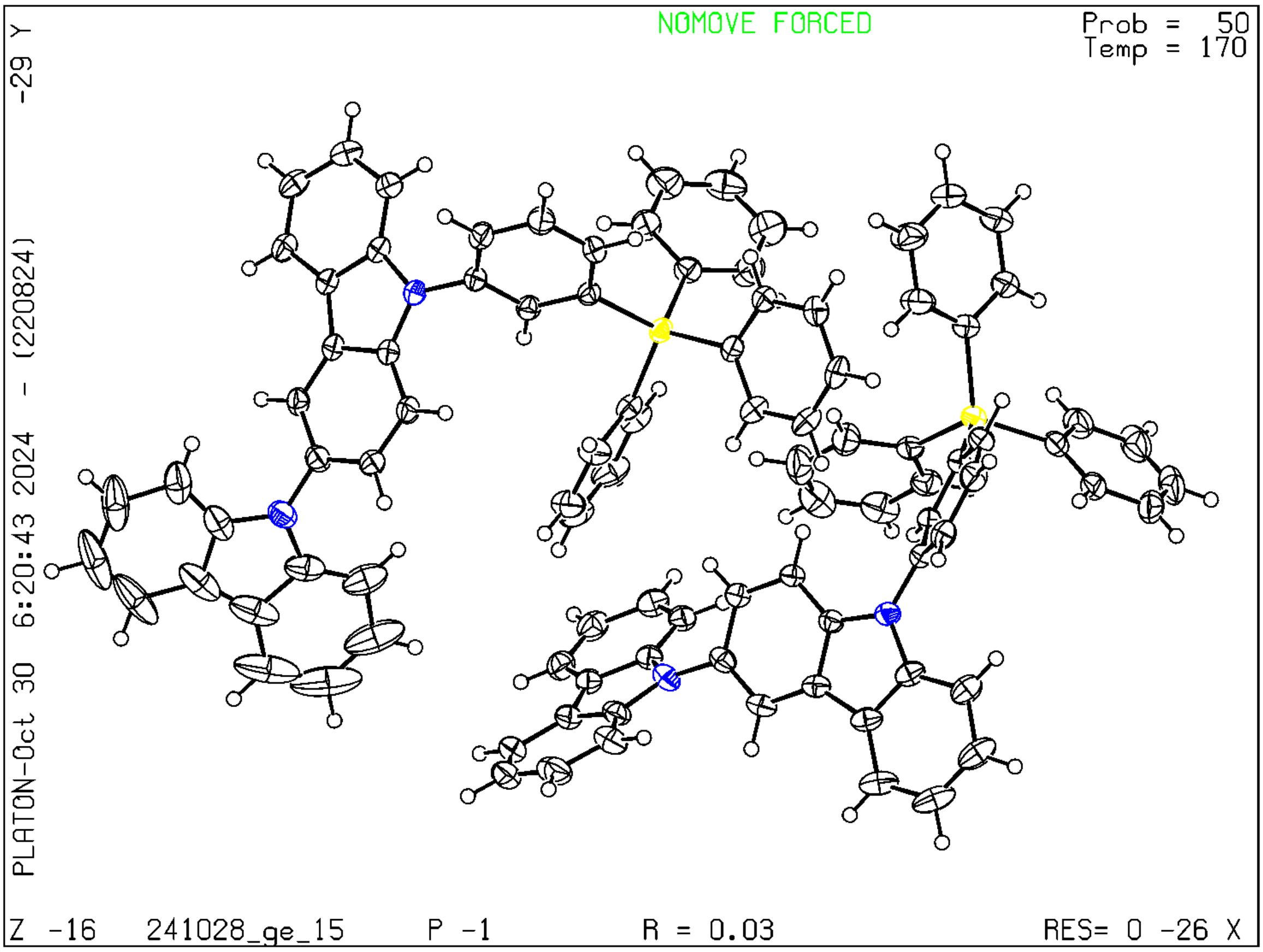

Supplement: Supplementary file 5 [file 41467_2026_76914_MOESM5_ESM.pdf]
