# Supplementary material for: Germanium-integrated exciplex host for high-performance narrowband OLEDs with mitigated efficiency roll-off
Source: Nat Commun. 2026 Aug 20;17:9942. doi: 10.1038/s41467-026-76914-5 (PMC13586258; doi:10.1038/s41467-026-76914-5)

checkCIF/PLATON report

You have not supplied any structure factors. As a result the full set of tests cannot be run.

THIS REPORT IS FOR GUIDANCE ONLY. IF USED AS PART OF A REVIEW PROCEDURE FOR PUBLICATION, IT SHOULD NOT REPLACE THE EXPERTISE OF AN EXPERIENCED CRYSTALLOGRAPHIC REFEREE.

No syntax errors found.   CIF dictionary   Interpreting this report

Datablock: 241028_zx_16

Bond precision: C-C = 0.0030 A   Wavelength=1.34139

Cell:	a=13.5854(8)	b=18.5266(10)	c=24.3405(13)
	alpha=74.668(2)	beta=83.951(2)	gamma=73.566(2)
Temperature:	170 K		

	Calculated	Reported
Volume	5663.8(5)	5663.8(5)
Space group	P -1	P -1
Hall group	-P 1	-P 1
Moiety formula	C51 H35 Ge N5	C51 H35 Ge N5
Sum formula	C51 H35 Ge N5	C51 H35 Ge N5
Mr	790.45	790.43
Dx,g cm-3	1.390	1.390
Z	6	6
Mu (mm-1)	0.946	0.946
F000	2448.0	2448.0
F000'	2443.26	
h,k,lmax	17,24,31	17,24,31
Nref	26014	25843
Tmin,Tmax	0.873,0.954	0.678,0.752
Tmin'	0.835	

Correction method= # Reported T Limits: Tmin=0.678 Tmax=0.752
AbsCorr = MULTI-SCAN

Data completeness= 0.993   Theta(max)= 60.619

R(reflections)= 0.0390(20597)   wR2(reflections)= 0.1087(25843)

S = 1.024   Npar= 1540

The following ALERTS were generated. Each ALERT has the format
test-name_ALERT_alert-type_alert-level.
Click on the hyperlinks for more details of the test.

Alert level C

PLAT601_ALERT_2_C Unit Cell Contains Solvent Accessible VOIDS of . 46 Ang**3

Alert level G

ABSMU01_ALERT_1_G Calculation of _exptl_absorpt_correction_mu
not performed for this radiation type.
PLAT012_ALERT_1_G No _shelx_res_checksum Found in CIF ...... Please Check
PLAT154_ALERT_1_G The s.u.'s on the Cell Angles are Equal ..(Note) 0.002 Degree
PLAT933_ALERT_2_G Number of HKL-OMIT Records in Embedded .res File 51 Note

```
 -2  2  6,  -3 -2  8,   1  3  7,   5 -3  3,  -5  0  7,  -5  4  5,
  0 -6  5,   0 -2  1,   2  3  7,   1 10  5,   1  0  4,  -2 -2  7,
  1  6  7,   0  1  0,   2  2  7,  -4-10  1,   0 -5  5,   2  3  8,
 -1 -9  4,  -5 -2  6,  -4  6  2,  -1 -3  8,   1 -5 11,  -2 -3  1,
  1  3  2,   0 -7  6,   2  3  3,   0 -8  2,   4  7  3,  -1  0 11,
  0 -2  4,   0  0  1,   5  6 12,   5 -1  1,  -4  6  8,   4 -3  1,
  0 -3 12,   4 -4  3,   0  1  7,   1  0 13,   4  1 11,   1 -3 11,
 -3  4  1,   1 -2  8,   5  0  6,  -7  0  3,  -3  3 10,   1  6  5,
 -1 -5  6,  -1 -8  3,
```

0 **ALERT level A** = Most likely a serious problem - resolve or explain
0 **ALERT level B** = A potentially serious problem, consider carefully
1 **ALERT level C** = Check. Ensure it is not caused by an omission or oversight
4 **ALERT level G** = General information/check it is not something unexpected

3 ALERT type 1 CIF construction/syntax error, inconsistent or missing data
2 ALERT type 2 Indicator that the structure model may be wrong or deficient
0 ALERT type 3 Indicator that the structure quality may be low
0 ALERT type 4 Improvement, methodology, query or suggestion
0 ALERT type 5 Informative message, check

It is advisable to attempt to resolve as many as possible of the alerts in all categories. Often the minor alerts point to easily fixed oversights, errors and omissions in your CIF or refinement strategy, so attention to these fine details can be worthwhile. In order to resolve some of the more serious problems it may be necessary to carry out additional measurements or structure refinements. However, the purpose of your study may justify the reported deviations and the more serious of these should normally be commented upon in the discussion or experimental section of a paper or in the "special_details" fields of the CIF. checkCIF was carefully designed to identify outliers and unusual parameters, but every test has its limitations and alerts that are not important in a particular case may appear. Conversely, the absence of alerts does not guarantee there are no aspects of the results needing attention. It is up to the individual to critically assess their own results and, if necessary, seek expert advice.

Publication of your CIF in IUCr journals

A basic structural check has been run on your CIF. These basic checks will be run on all CIFs submitted for publication in IUCr journals (*Acta Crystallographica*, *Journal of Applied Crystallography*, *Journal of Synchrotron Radiation*); however, if you intend to submit to *Acta Crystallographica Section C* or *E* or *IUCrData*, you should make sure that full publication checks are run on the final version of your CIF prior to submission.

Publication of your CIF in other journals

Please refer to the *Notes for Authors* of the relevant journal for any special instructions relating to CIF submission.

PLATON version of 22/08/2024; check.def file version of 21/08/2024

Datablock 241028_zx_16 - ellipsoid plot

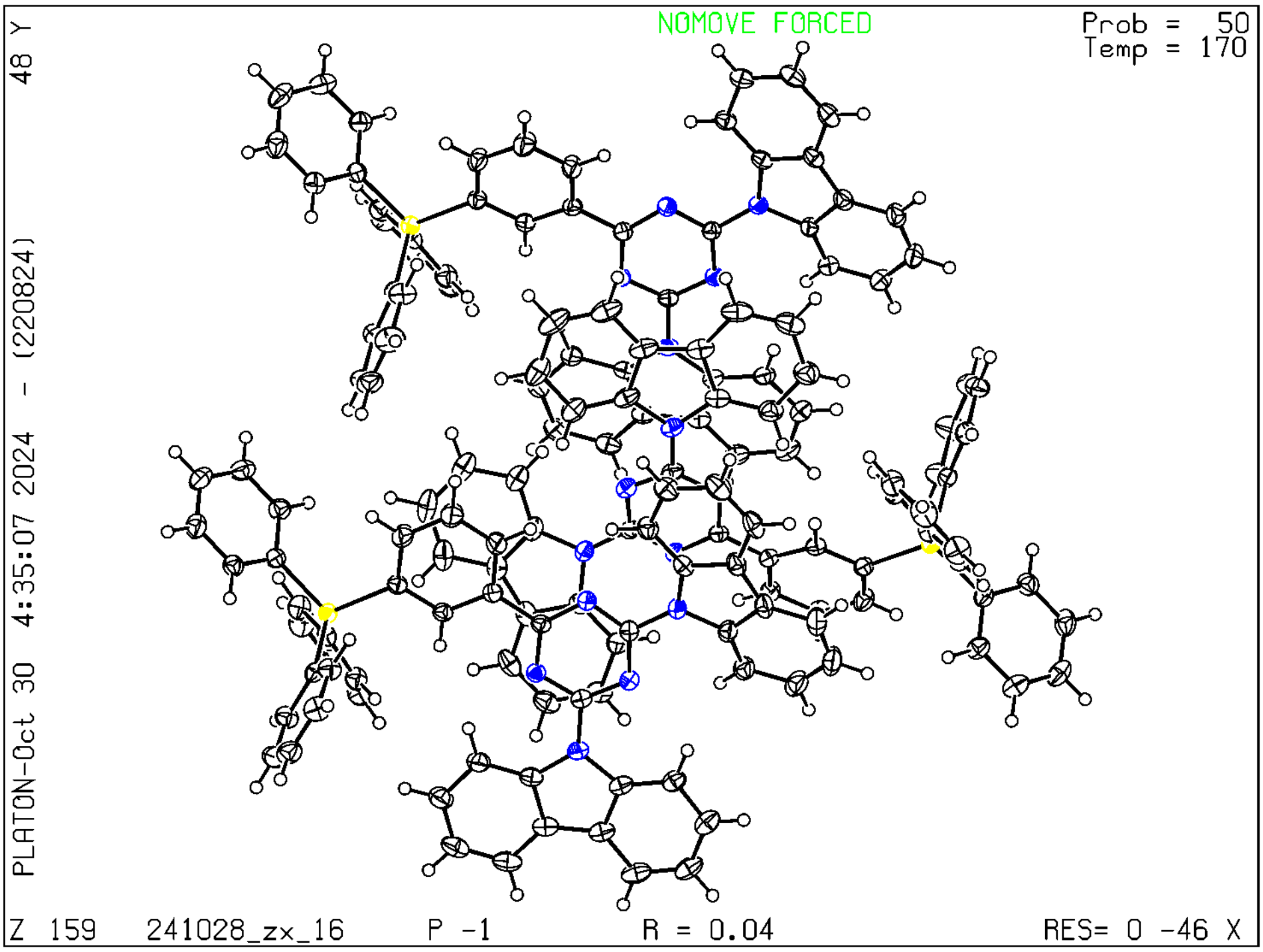

Supplement: Supplementary file 6 [file 41467_2026_76914_MOESM6_ESM.pdf]
