# Supplementary material for: Germanium-integrated exciplex host for high-performance narrowband OLEDs with mitigated efficiency roll-off
Source: Nat Commun. 2026 Aug 20;17:9942. doi: 10.1038/s41467-026-76914-5 (PMC13586258; doi:10.1038/s41467-026-76914-5)
Supplement: Supplementary file 7 [file 41467_2026_76914_MOESM7_ESM.pdf]

nature portfolio

Peer Review File

Germanium-integrated exciplex host for high-performance narrowband OLEDs with mitigated efficiency roll-off

Corresponding Author: Professor Chuluo Yang

This file contains all reviewer reports in order by version, followed by all author rebuttals in order by version.

Version 0:

Reviewer comments:

Reviewer #1

(Remarks to the Author)
The paper by Yang and coworkers reported on a germanium-integrated exciplex host for blue and green OLEDs. The introduction heavy-atom germanium has been demonstrated to accelerate both radiative and RISC processes, but not induce any discernible effect on the ground and excited states. The emitting devices exhibit exceptionally high EQEs over 40%, surpassing the Si-based counterparts. It is a good step forward, but I do feel that the novelty and significance of this work are not clearly presented and some results do not fully support the authors' argument. Therefore, I think the paper might be suitable for Nature Communications after addressing the following issues:
1. The author mentioned that both GeCzCz and GeTrzCz2 exhibit higher triplet energies than their Si-based counterparts. In fact, the value of 2.91 eV for GeTrzCz2 is nearly equivalent to the value of 2.89 eV for SiTrzCz2, but the value of 3.12 eV for GeCzCz is much higher than the value of 2.92 eV for SiCzCz. What accounts for the apparent differences?
2. The author should provide the method by which the FRET radius was calculated in the Methods section.
3. The author exclusively provides lifetime curve for green devices, while neglecting to offer such data for blue devices. Nevertheless, at present, blue device stability represents the most significant challenge confronting the industry. It is imperative that the author provides a detailed comparison of the introduction of germanium with that of silicon, alongside a thorough analysis of the reasons for any observed discrepancies in stability. This will allow for the effective highlighting of the features of the manuscript.
4. Whether the introduction of germanium affects the horizontal dipole orientation?
5. For the host material, it is fundamental that the authors provide a comprehensive analysis of the transport properties, encompassing either a complete single-carrier device analysis or a mobility analysis.
6. The author's statement for the comparison of efficiency roll-off appears to be questionable. A more appropriate approach would be to compare the percentage of decay in efficiency at the corresponding luminance, as opposed to the efficiency value at the corresponding luminance, as the latter does not accurately reflect the advantages of the introduction of germanium.
7. In order to facilitate a more substantial scientific study, it is recommended that the author synthesizes selenium-based comparison molecules. This would enable a more effective comparison of the effects of heavy atom effects, such as carbon, silicon, germanium and selenium, on the exciplex host materials.
8. The comparisons in Supplementary Table 4 appear to lack rational coherence. I would recommend dropping the relevant argument and that table.

Reviewer #2

(Remarks to the Author)
The comparative analysis and logical discussion of replacing silicon exciplexes with germanium exciplexes, which form the core of this paper, are critically insufficient. Therefore, this paper is deemed unsuitable for publication in Nature Communications in its current form. The difference in photophysical properties observed between germanium and silicon exciplex systems is negligible and not significant enough to warrant special consideration. The manuscript lacks compelling evidence to support the proposed contribution of heavy atom effect. Furthermore, the lack of direct comparison of OLED performance in germanium and silicon exciplex systems weakens the authors' claims. Therefore, it cannot be concluded that the germanium exciplex system described in this study represents a significant advance over the previously reported silicon

exciplex system. Therefore, the manuscript does not meet the criteria for originality and novelty for publication in Nature Communications. If this is to be published elsewhere, the following points should be further considered to provide sufficient supporting evidence.

Major issues:
1. To claim that energy transfer primarily occurs in the manner shown in Figure 1a, further discussion on whether the exciplex system effectively suppresses DET transfer is necessary. The DET rate constant should be analyzed.
2. The absence of an explanation for the threefold increase in SOCME raises some concerns. It would be valuable to provide data on the T1 NTO transition (hole to electron) for both the Si and Ge versions. Given that Si and Ge are positioned in sp3 hybridized sites, a more detailed NTO analysis would help to substantiate the presence of the heavy atom effect. Additionally, it would be appreciated clarification on whether the upper excited state levels and the corresponding SOCME have been analyzed.
3. According to the SI Table 1 fitting results, there appear to be no significant differences in the key rate constants related to the heavy atom effect, so additional explanation on this point is necessary. Also, how can the approximately 1.8-fold increase in PLQY be explained? Specifically, an explanation is needed for why such a difference occurs despite no significant differences in the rates of other radiative and non-radiative transitions.
4. In the device results shown in Figure 4 and Table 1, the EQE is particularly high at the driving voltage, but then drops sharply. An explanation for this behavior is needed. It is also necessary to provide an explanation for why the roll-off is so massive.
5. All the device lifetime data mentioned in the manuscript should be provided. In particular, comparison with the Si exciplex devices is necessary to demonstrate the differences in the external heavy atom effect originating from the host. Additionally, the differences in roll-off efficiency should be addressed.
6. It would be beneficial to examine the CV reproducibility difference when substituting Si with Ge. Analyzing the increased stability of holes and electrons within the exciplex system would provide a crucial basis for explaining the improved device stability presented in Figure 4g.
7. It would be interesting to know whether there is a difference in PL stability arising from the heavy atom effect in the thin doped films. PL stability should be measured for the emitter in both Si and Ge hosts.

Minor points:
1. The substitution of Si with Ge should be further analyzed by comparing the Transient-PL data to examine the differences. It would be better to present the comparison of the prepared data in a single figure for more effective analysis. Furthermore, the manuscript or SI should provide Transient-PL data related to prompt fluorescence.
2. The advantages of this type of Ge-exciplex device, aside from the driving voltage, compared to the currently widely reported HF systems should be clarified. Additionally, the PE and CE values at 1000 cd for the measurement of device stability should be provided.
3. The purity of the material should be clarified. It would also be helpful to provide the HPLC and/or HRMS data across the entire m/z range, not just for the main peak.
4. It would be interesting to know whether the bonding dissociation energy (BDE) of both p-type and n-type host materials upon substituting Si with Ge has been calculated and compared, as this could provide evidence for whether the effect of substituting Si with Ge is solely due to the heavy atom effect or if it also contributes to device stability.
5. The basic quantum chemical calculation results for the Ge host materials, such as oscillator strength, S1, T1, and reorganization energy, should be provided.
6. The angular PL orientation of all films should be provided. Explanations of how it relates to the EQEs would be necessary to explain high maximum EQEs.

Reviewer #3

(Remarks to the Author)
The authors report a strategy to improve device efficiency roll-off in OLEDs using an exciplex host formed between hole (GeCzCz) and electron (GeTrzCz2) transport moieties containing germanium. The exciplex formation between GeCzCz and GeTrzGCz2 is demonstrated by the red-shifted emission with longer lifetime observed in the blended film, compared to neat films of GeCCz and GeTrzCz2. The heavy-atom effect caused by the presence of Ge (Z=32) accelerates the spin-flip dynamics and enhances RISC rates in exciplex host, compared with the silicon SiCCz:SiTrzCz2 exciplex. Spin-orbit coupling matrix elements (SOCMEs) calculated for (SiCzCz:SiTrzCz2) and (GeCzCz:GeTrzCz2) show an increase from 0.265 cm-1 to 0.791 cm-1, consistent with the expected enhancement in spin-orbit coupling.

TADF Blue and Green OLEDs were fabricated with the germanium exciplex host and its silicon counterpart host. The OLEDs with the Ge exciplex host show impressive performances that consistently outperform the performances of devices with the silicon host, which demonstrates the strategy is effective on enhancing spin-flip dynamics and effectively harvesting triplet states.

I think the work is very interesting and well done. We could ask for further confirmation of TADF in the host exciplex which has not been fully tested, but the results show that TADF is most likely reason for the observation of delayed fluorescence. Thus, I don’t think it is necessary to request further confirmation. The manuscript is written in a concise manner and well organized. Therefore, I have no further questions, and recommend the manuscript to be accepted for publication in the current form.

Version 1:

Reviewer comments:

Reviewer #1

(Remarks to the Author)
It is acceptable as is.

Reviewer #2

(Remarks to the Author)
The current version has been significantly improved through the authors' revisions. However, it still contains ambiguities and contradictions in fundamental aspects.

Major points:
1. The reported enhancement in SOCME upon substitution of Si with Ge constitutes a central mechanistic argument of the manuscript. Basically, to improve transparency and reproducibility, the authors are also encouraged to describe the computational protocol used for SOC analysis in sufficient detail (e.g., functional and basis set, relativistic treatment, SOC evaluation approach, and the method used for atomic/fragment decomposition). While the authors attribute this enhancement partly to heavy-atom effects and hyperconjugative interactions at the sp3-bridged position, the theoretical basis for invoking hyperconjugation remains insufficiently clarified. In Supplementary Fig. 17, SOCMEs and atomic/fragment contributions are presented for the Si- and Ge-based systems. However, it is not clearly established how hyperconjugation is defined and quantified in this context. It would be helpful if the authors could clarify whether direct computational evidence (e.g., NBO analysis, orbital interaction analysis, or second-order perturbation energies) demonstrates σ–π mixing involving the sp3-bridged atom, and how such mixing mechanistically translates into increased SOC matrix elements. Furthermore, since the manuscript aims to establish a systematic C → Si → Ge trend, it would be important to include SOC calculations for the carbon-based exciplex as well. At present, the discussion focuses on the Si and Ge systems, which makes it difficult to assess whether the SOC enhancement follows a consistent progression across the entire series.

2. Spin–orbit coupling matrix elements can be sensitive to molecular geometry, particularly in exciplex systems involving through-space donor–acceptor interactions and conformational flexibility. It would therefore be helpful if the authors could clarify how the molecular geometry used for SOC calculations was selected.

Specifically:
1) Was a conformational search performed prior to geometry optimization?
2) Were multiple low-energy conformers examined?
3)How sensitive are the SOCME values to conformational variation?

If the reported SOC enhancement remains consistent across energetically accessible conformations, this would strongly reinforce the robustness of the conclusion. Finally, given the central importance of the computational results to the manuscript's mechanistic claims, I kindly request that the Cartesian coordinates of all optimized structures used for SOC calculations (carbon-, silicon-, and germanium-based systems) be provided in the Supporting Information to enhance reproducibility and transparency.

3. The quantitative estimation of kDET provides useful insight into exciton dynamics. However, based on Supplementary Table 2, the absolute kDET values remain on the order of 106 s-1, whereas kRISC lies in the 104–105 s−1 regime. This suggests that Dexter energy transfer is kinetically faster than RISC in several cases In addition, Figure 1a schematically presents the proposed energy-transfer pathway. Given that DET appears to be kinetically competitive—or even faster—than RISC in several systems, it would be helpful if the authors could clarify whether the schematic represents the dominant kinetic pathway or serves as a conceptual illustration. A clearer reconciliation between the measured rate constants and the pathway illustrated in Figure 1a would improve internal consistency.

4. The systematic increase in horizontal dipole ratio from the carbon- to silicon- to germanium-based hosts is an interesting and potentially important observation. However, the current discussion remains largely descriptive, and the mechanistic origin of the orientation trend is not fully clarified. It would be helpful if the authors could address the following points:

1) What structural or electronic features of the Ge-based host are responsible for promoting improved horizontal alignment? For example, could differences in steric bulk, molecular polarizability, intermolecular interactions, or free volume contribute to the observed trend?
2) Is there any change in the intrinsic transition dipole moment (TDM) direction or magnitude of the emitters when embedded in different host environments? Providing computational analysis of TDM vectors or host–guest configurations would help clarify whether the improvement arises from host-induced alignment effects or intrinsic electronic changes.
3) Given that some reported differences in Θ// appear relatively small, could the authors provide uncertainty estimates, fitting sensitivity analysis, or reproducibility data to confirm that the observed C → Si → Ge trend is statistically meaningful?
If possible, optical simulations incorporating the measured dipole orientation would help quantify how much of the EQE enhancement can be attributed to improved outcoupling versus intrinsic photophysical improvements.

A more quantitative discussion linking molecular orientation, TDM behavior, and device efficiency would substantially

strengthen the interpretation of the orientation-dependent performance enhancement.

5. While the increase in kr,S and decrease in knr,S quantitatively explain the enhancement in PLQY, the mechanistic origin of these changes remains insufficiently clarified.

1) In the rebuttal, the reduction in knr,S is attributed to increased molecular packing inferred from higher crystal density. However, could the authors clarify how crystal density translates into suppressed non-radiative decay in amorphous exciplex films?
2) If tighter molecular packing is proposed to suppress non-radiative decay, how does this interpretation reconcile with the intramolecular reorganization energies reported in Supplementary Fig. 13? A more quantitative discussion linking reorganization energy, vibrational coupling, and non-radiative decay rates would significantly strengthen the mechanistic explanation for the enhanced PLQY.
3) Have mode-specific contributions (e.g., dominant vibrational modes or Huang–Rhys factors) been analyzed to determine whether vibrational coupling is indeed reduced in the Ge-based system?

6. Supplementary Fig. 20 includes hole-only and electron-only device (HOD/EOD) measurements for the Ge-based system. However, a direct comparison with the corresponding C- and Si-based analogues is not clearly presented. If feasible, it would be beneficial to fabricate and characterize HOD/EOD devices for the C- and Si-based host systems under identical conditions. Such comparative data would allow a more quantitative assessment of whether the improved performance in the Ge-based system indeed arises from enhanced charge transport or balance, rather than from purely photophysical effects.

7. Given that the carbon-based device exhibits the shortest operational lifetime, a clearer explanation of why the C system is more susceptible to degradation from an exciton-dynamics perspective would strengthen the argument. It would also be helpful to clarify whether differences in thin-film properties among the C-, Si-, and Ge-based systems contribute to the stability trend, enabling a more systematic comparison across the series.

8. Given that the carbon-based device exhibits the shortest operational lifetime, it would be helpful to clarify whether its electrochemical stability differs systematically from the Si- and Ge-based systems. If cyclic voltammetry (CV) data are available for the C analogue, a brief comparison of oxidation/reduction behavior may provide additional insight into its relative device stability. Even a short discussion on whether electrochemical robustness correlates with the observed lifetime trend would further strengthen the stability analysis.

Minor points:
1. Given the very high in-plane dipole orientation reported (up to ~99%), the authors state in the rebuttal that κ2 = 2/3 was assumed in the RFRET calculation. It would be helpful if the authors could clarify whether this isotropic assumption remains appropriate under such strongly anisotropic dipole distributions, and whether the use of κ2 = 2/3 introduces any significant deviation in the estimated RFRET values. A brief discussion on the potential impact of dipole alignment on the FRET analysis would improve internal consistency.
2. It would also be helpful to clarify whether the same FRET analysis was performed for the carbon-based analogue, to consistently establish the C → Si → Ge progression.
3. It is stated that a refractive index of n = 1.7 was assumed in the RFRET calculation. Since the Förster radius depends on n−4, the estimated RFRET values can be sensitive to the choice of refractive index. It would be helpful if the authors could clarify whether the value n = 1.7 was experimentally measured for the present host films or adopted as a general assumption. If it is an assumed value, a brief discussion on the sensitivity of RFRET to variations in n (e.g., within a realistic range of organic thin films) would strengthen the quantitative robustness of the FRET analysis.
4. Supplementary Fig. 14 presents calculated bond dissociation energies (BDEs) for the host systems. It would be helpful if the authors could clarify in which electronic state the BDE calculations were performed.
5. Supplementary Fig. 13 presents the calculated parameters for the Ge-based system. For completeness and consistency, it would be helpful if the corresponding calculated data for the C- and Si-based analogues could also be provided in the same format. A direct comparison across the C → Si → Ge series would allow a more systematic evaluation of the trends and further strengthen the comparative discussion.

Version 2:

Reviewer comments:

Reviewer #2

(Remarks to the Author)
The authors have made a careful and substantial revision of the manuscript. The revised version has been significantly improved, and my previous concerns have been satisfactorily addressed. In particular, the clarified SOC calculation protocol, additional C/Si/Ge comparative and conformational analyses, corrected dipole-orientation measurements, optical simulations, and additional device/stability data have strengthened the mechanistic interpretation and internal consistency of the work.
The claims are now appropriately moderated and sufficiently supported by the revised data. I have no further major concerns and recommend publication in Nature Communications.

Open Access This Peer Review File is licensed under a Creative Commons Attribution 4.0 International License, which permits use, sharing, adaptation, distribution and reproduction in any medium or format, as long as you give appropriate credit to the original author(s) and the source, provide a link to the Creative Commons license, and indicate if changes were made.
In cases where reviewers are anonymous, credit should be given to 'Anonymous Referee' and the source.
The images or other third party material in this Peer Review File are included in the article's Creative Commons license, unless indicated otherwise in a credit line to the material. If material is not included in the article's Creative Commons license and your intended use is not permitted by statutory regulation or exceeds the permitted use, you will need to obtain permission directly from the copyright holder.
To view a copy of this license, visit https://creativecommons.org/licenses/by/4.0/

Dear Reviewers,

Enclosed please find our revised manuscript entitled "**Germanium-integrated exciplex host for high-performance narrowband OLEDs with mitigated efficiency roll-off**" (Manuscript ID: **NCOMMS-25-25950A**) for publication as a research article in ***Nature Communications***. We sincerely thank the reviewers for their insightful and professional comments, which helped us substantially improve the quality of our manuscript.

We revised the manuscript thoroughly in response to the reviewers' comments and the point-by-point responses are provided. The major improvements upon revision include:

1. Carbon-based reference molecules are synthesized and used as controls to more clearly confirm the heavy-atom effect of germanium.
2. Stability tests are conducted for all devices and discussed in the manuscript. Additionally, the reproducibility of cyclic voltammetry and the photoluminescence stability of the emitting layers are evaluated.
3. The photophysical data is deeply analyzed. The calculation formulas and energy transfer process are also added in both the manuscript and supplementary information.
4. Single-carrier devices are supplemented for comprehensive analysis of charge-transport properties.
5. The figures and tables in the manuscript and supporting information are optimized for enhanced clarity.

For your reference, annotated files of the revised manuscript and supporting information with all changes highlighted in yellow are also attached. We hope that the revised paper could now be satisfied for your requirements.

Yours Sincerely,

Chuluo Yang on behalf of the authors.

Corresponding author: **Professor Chuluo Yang**
at College of Materials Sciences and Engineering, Shenzhen University, Shenzhen 518071, People's Republic of China. Tel: +86-0755-86713985, e-mail: clyang@szu.edu.cn.

We greatly appreciate the reviewers for taking the time to review and critique our manuscript.

Accordingly, we have fully addressed the concerns raised by the reviewers.

Reviewer #1:

General comment: *The paper by Yang and coworkers reported on a germanium-integrated exciplex host for blue and green OLEDs. The introduction heavy-atom germanium has been demonstrated to accelerate both radiative and RISC processes, but not induce any discernible effect on the ground and excited states. The emitting devices exhibit exceptionally high EQEs over 40%, surpassing the Si-based counterparts. It is a good step forward, but I do feel that the novelty and significance of this work are not clearly presented and some results do not fully support the authors' argument. Therefore, I think the paper might be suitable for Nature Communications after addressing the following issues:*

Response: We sincerely appreciate the reviewer's positive and professional comments.

Comment 1: *The author mentioned that both GeCzCz and GeTrzCz2 exhibit higher triplet energies than their Si-based counterparts. In fact, the value of 2.91 eV for GeTrzCz2 is nearly equivalent to the value of 2.89 eV for SiTrzCz2, but the value of 3.12 eV for GeCzCz is much higher than the value of 2.92 eV for SiCzCz. What accounts for the apparent differences?*

Response: We thank the reviewer for pointing out this discrepancy and apologize for the oversight in our previous submission. The reported triplet energy of 3.12 eV for the GeCzCz neat film was anomalously high and likely arose from an artifact in our film fabrication or measurement setup. To address this, we have prepared a new neat film of GeCzCz and re-measured its phosphorescence spectrum at 77 K. The onset of this spectrum yields a corrected T_1 energy of 2.94 eV for GeCzCz, which is nearly equivalent to the 2.92 eV value for its SiCzCz counterpart (Supplementary Fig. 12). We have updated the relevant descriptions in the manuscript accordingly.

Modified sentences (Manuscript, page 6): "…(Supplementary Fig. 12) revealed T_1 energies of 2.94 eV for GeCzCz and 2.91 eV for GeTrzCz2, both nearly equivalent to those of the SiCzCz and SiTrzCz2 reference…"

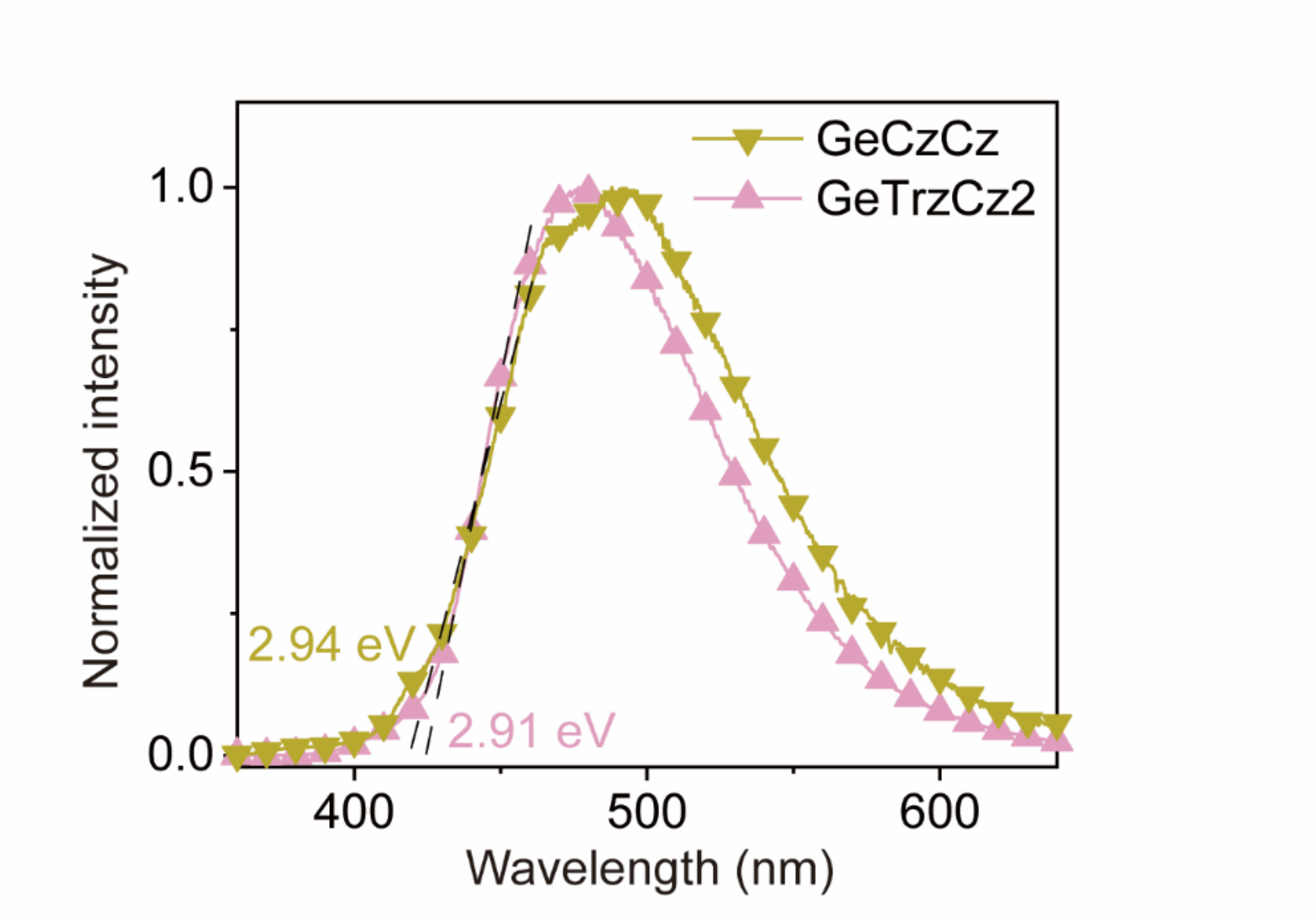

Supplementary Fig. 12. Phosphorescence spectra of GeCzCz, and GeTrzCz2 in solid films at 77 K.

Comment 2: *The author should provide the method by which the FRET radius was calculated in the Methods section.*

Response: We appreciate the reviewer for the constructive suggestion. The method used to calculate the FRET radius (R_{FRET}) has been added to the Methods section.

Additional section (Methods):

"**R_{FRET} calculation**

The rate of Förster resonance energy transfer (k_{FRET}) can be expressed as:

$$k_{\mathrm{FRET}} = k_{r,\mathrm{D}} \frac{\kappa^2}{R^6} \left(\frac{9000(\ln 10)}{128\pi^5 N_{\mathrm{A}} n^4} \right) \int_0^\infty F_{\mathrm{D}}(\lambda) \varepsilon_{\mathrm{A}}(\lambda) \lambda^4 d\lambda. \tag{1}$$

where R is the intermolecular distance of the donor (GeCzCz:GeTrzCz2 exciplex host) and acceptor (*t*-DABNA, *v*-DABNA, and *ω*-DABNA); $k_{r,\mathrm{D}}$ is the radiative decay rate constant of the donor (GeCzCz:GeTrzCz2 exciplex host) in absence of the acceptor (*t*-DABNA, *v*-DABNA, and *ω*-DABNA); κ^2 is a configurational factor describing the relative orientation of electric transition dipole moments of the donor (GeCzCz:GeTrzCz2 exciplex host) and acceptor (*t*-DABNA, *v*-DABNA, and *ω*-DABNA) and is assumed to be 2/3 for a random distribution of the donor-acceptor pairs in our study; N_{A} is the Avogadro constant; n is the refractive index of the medium, for most of the organic materials is about 1.7; $F_{\mathrm{D}}(\lambda)$ is the normalized emission spectra of the donor (GeCzCz:GeTrzCz2 exciplex host); $\varepsilon_{\mathrm{A}}(\lambda)$ is the molar absorption coefficient of the acceptor (*t*-DABNA, *v*-DABNA, and *ω*-DABNA); and λ is the corresponding wavelength. The R_{FRET} is defined as an intermolecular distance at which the k_{FRET} is equal to the $k_{r,\mathrm{D}}$."

Comment 3: *The author exclusively provides lifetime curve for green devices, while neglecting to offer such data for blue devices. Nevertheless, at present, blue device stability represents the most significant challenge confronting the industry. It is imperative that the author provides a detailed comparison of the introduction of germanium with that of silicon, alongside a thorough analysis of the reasons for any observed discrepancies in stability. This will allow for the effective highlighting of the features of the manuscript.*

Response: We appreciate the reviewer's constructive suggestion. In the revised manuscript, we have included operational lifetime curves for both blue and green devices (Fig. 4g-i) and provided a direct comparison between germanium- and silicon-based systems.

Specifically, the deep-blue device based on GeCzCz:GeTrzCz2:*t*-DABNA and the blue device based on GeCzCz:GeTrzCz2:*v*-DABNA exhibit operational half-lifetimes (LT_{50}) of 8.6 h and 29.3 h at an initial brightness (L_0) of 1,000 cd·m^{-2}, respectively. As expected, these lifetimes are shorter than those of the green device based on GeCzCz:GeTrzCz2:*ω*-DABNA, primarily because higher-energy blue light accelerates material degradation.

Moreover, we compared these devices with control devices based on SiCzCz:SiTrzCz2 exciplex host. When *t*-DABNA was employed as the emitter, the germanium-based and silicon-based devices show comparable operational lifetimes, likely because the high emission energy of *t*-DABNA compromises the chemical stability of both exciplex hosts. In contrast, the blue device based on GeCzCz:GeTrzCz2:*v*-DABNA and the green device based on GeCzCz:GeTrzCz2:*ω*-DABNA display longer operational lifetimes than their silicon counterparts. We attribute these improvements to the use of germanium in the exciplex host for *v*-DABNA and *ω*-DABNA guests, which shortens the emission lifetime and reduces the population of long-lived triplet states. This, in turn, mitigates degradation pathways such as triplet-triplet and triplet-polaron annihilation, thereby enhancing operational stability. These data and the detailed discussion have been incorporated into the revised manuscript.

Additional sentence, (manuscript, page 11): "Next, we assessed the operational stability of these devices at an initial luminance of 1,000 cd·m^{-2}, as shown in Fig. 4g-i. The CCzCz:CTrzCz2-based devices exhibited the poorest stability, with operational half-lifetimes (LT_{50}) of 0.2-0.5 h. The SiCzCz:SiTrzCz2-based devices revealed improved stability, achieving LT_{50} values of 9.0 h, 24.6 h, and 207.8 h for *t*-DABNA, *υ*-DABNA, and *ω*-DABNA, respectively. The GeCzCz:GeTrzCz2-based devices demonstrated the best overall performance (LT_{50} values of 8.8 h, 29.3 h, and 241.1 h for *t*-DABNA, *υ*-DABNA, and *ω*-DABNA, respectively). Notably, when *t*-DABNA was employed as the emitter, the germanium-based and silicon-based

devices showed comparable lifetimes, likely because the high emission energy of *t*-DABNA compromises the chemical stability of both exciplex hosts. In contrast, for guests with reduced emission energies (*v*-DABNA and *ω*-DABNA), the GeCzCz:GeTrzCz2-based devices displayed longer lifetimes than the silicon-based control devices, due to the decreased triplet population associated with the germanium-based exciplex host."

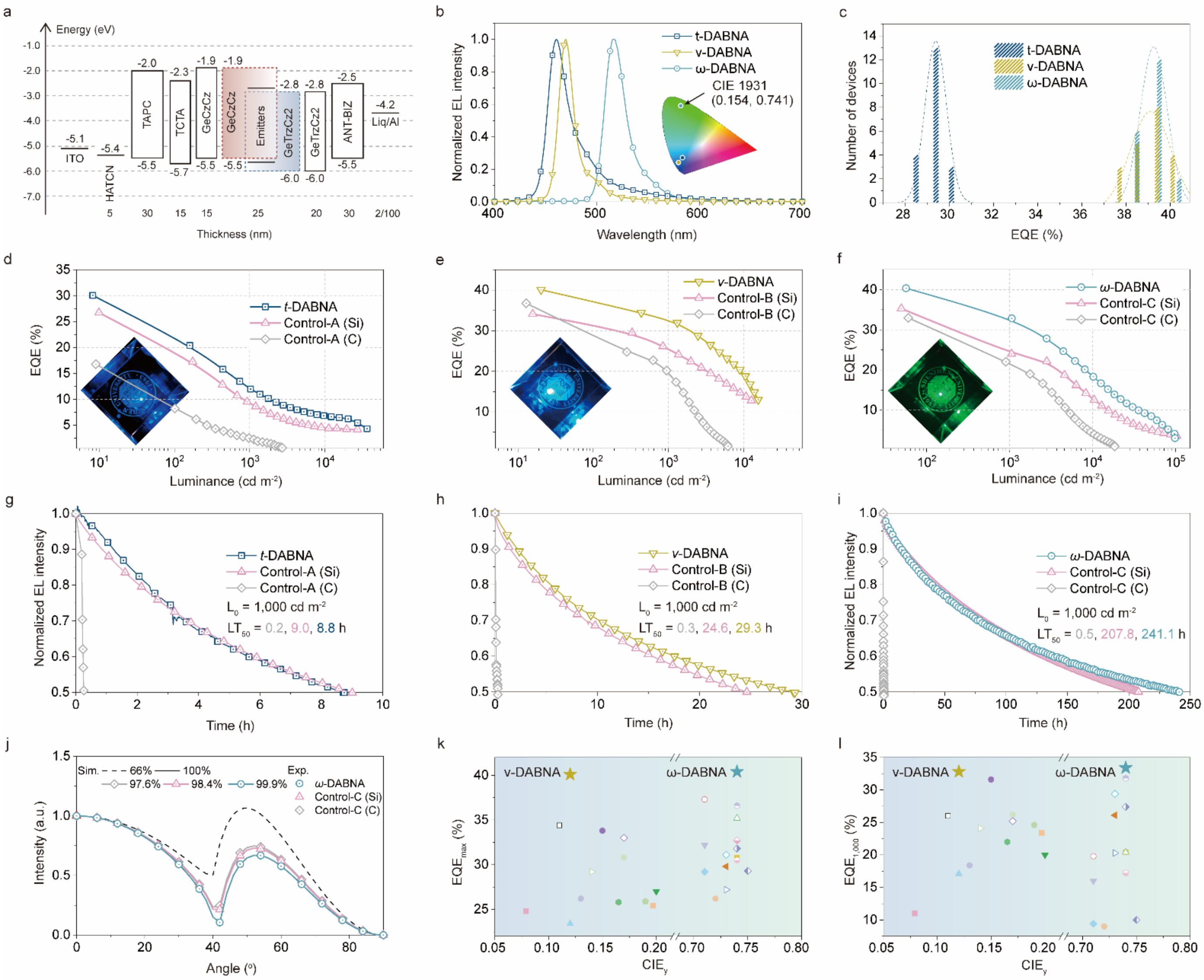

Fig. 4 | Optimized EL properties of OLEDs. a, Diagram of the germanium-based device structure. **b**, EL specrtra recorded at 1,000 cd·m^{-2}. c, Statistical histogram of EQEs for different devices. **d-f**, EQE versus luminance characteristics of target devices (*t*-DABNA (**d**), *υ*-DABNA (**e**), *ω*-DABNA (**f**)) and control devices (control-A (**d**), control-B (**e**), control-C (**f**)). **g**, Half-lifetime (LT_{50}) measurement of *t*-DABNA-based device and control devices (control-A) at an initial luminance of 1,000 cd·m^{-2}. **h**, LT_{50} measurement of *υ*-DABNA-based device and control devices (control-B) at an initial luminance of 1,000 cd·m^{-2}. **i**, LT_{50} measurement of *ω*-DABNA-based device and control devices (control-C) at an initial luminance of 1,000 cd·m^{-2}. **j**, Angle-dependent PL spectra of *ω*-DABNA-doped films. $\Theta_{//}$, horizontal emitting dipole orientation. **k,l**, Summary of

CIEy coordinates versus maximum EQE (**k**) values and EQE at 1,000 cd·m^{-2} (**l**) for state-of-the-art blue (CIEy ≤ 0.20) and green (CIEy ⩾ 0.71) OLEDs.

Comment 4: *Whether the introduction of germanium affects the horizontal dipole orientation?*

Response: We thank the reviewer for this insightful question. In response, we measured the angle-dependent *p*-polarized emission intensities (at the PL peak wavelength) for all emitting layer films used in the devices. For the *t*-DABNA-doped films, the horizontal dipole ratios ($\Theta_{//}$) are estimated to be 88.9%, 89.5% and 91.7% in the CCzCz:CTrzCz2, SiCzCz:SiTrzCz2 and GeCzCz:GeTrzCz2 hosts, respectively (Supplementary Fig. 32a). For the *υ*-DABNA-doped films, the estimated $\Theta_{//}$ values are 96.4%, 96.4% and 98.5% in the carbon, silicon, and germanium-based exciplex hosts, respectively (Supplementary Fig. 32b). For the *ω*-DABNA-doped films, the estimated $\Theta_{//}$ values are 97.6%, 98.4% and 99.9% in the carbon, silicon, and germanium-based exciplex hosts, respectively (Fig. 4j). As shown above, these $\Theta_{//}$ values' slight variations fall within the range of measurement error, and the introduction of germanium does not affect the horizontal dipole orientation.

Additional sentence, (manuscript, page 11): "To confirm the origin of the high efficiencies, the angle-dependent PL intensities were characterized to study the emitting dipole orientation of these emitting layer films. As illustrated in Fig. 4j and Supplementary Fig. 32, they showed very high horizontal emitting dipole orientation up to 99%, leading to high outcoupling efficiencies and consequently high EQEs, particularly when triplet excitons are efficiently utilized."

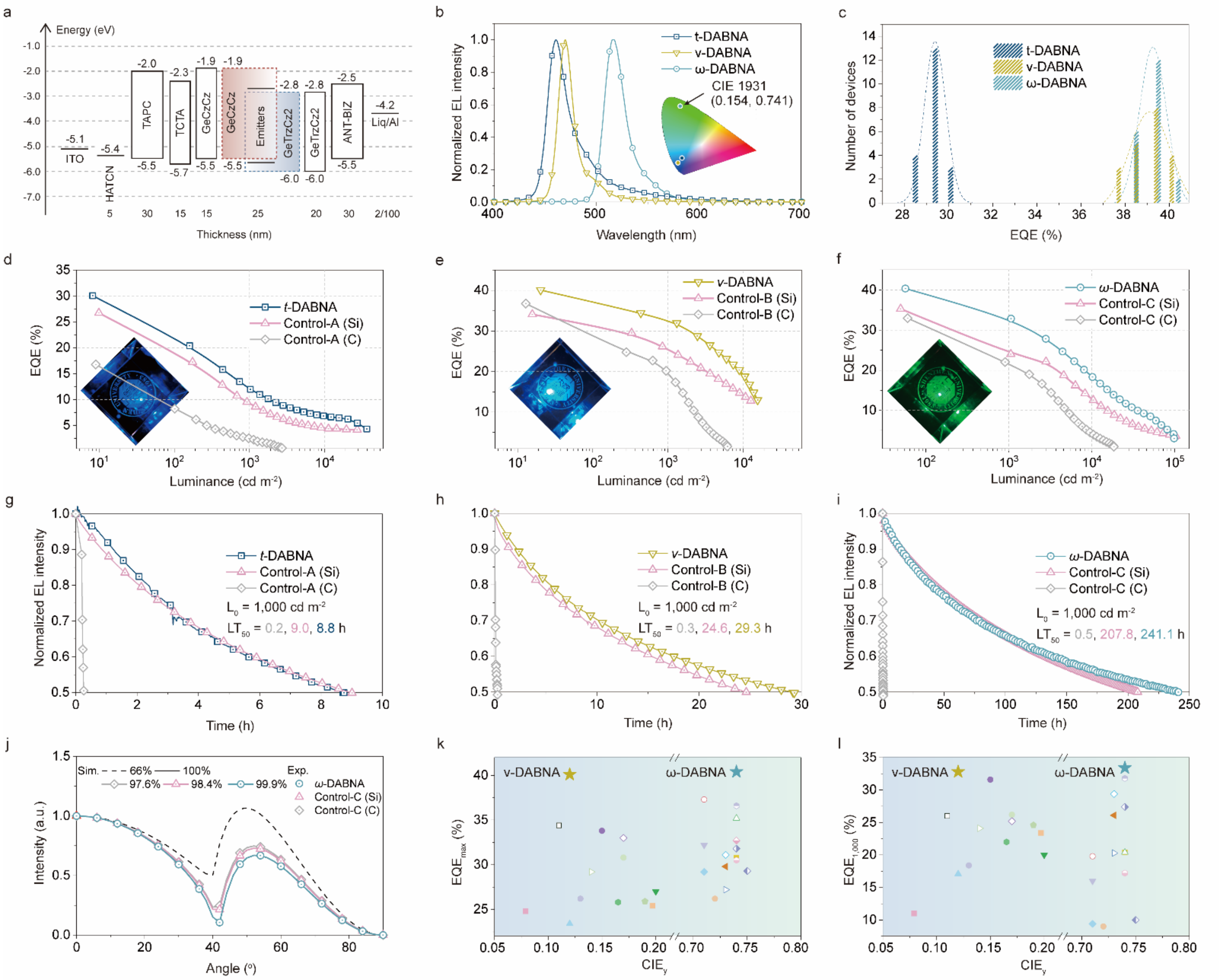

Fig. 4 | Optimized EL properties of OLEDs. a, Diagram of the germanium-based device structure. **b**, EL specrtra recorded at 1,000 cd·m^{-2}. c, Statistical histogram of EQEs for different devices. **d-f**, EQE versus luminance characteristics of target devices (*t*-DABNA (**d**), *υ*-DABNA (**e**), *ω*-DABNA (**f**)) and control devices (control-A (**d**), control-B (**e**), control-C (**f**)). **g**, Half-lifetime (LT_{50}) measurement of *t*-DABNA-based device and control devices (control-A) at an initial luminance of 1,000 cd·m^{-2}. **h**, LT_{50} measurement of *υ*-DABNA-based device and control devices (control-B) at an initial luminance of 1,000 cd·m^{-2}. **i**, LT_{50} measurement of *ω*-DABNA-based device and control devices (control-C) at an initial luminance of 1,000 cd·m^{-2}. **j**, Angle-dependent PL spectra of *ω*-DABNA-doped films. $\Theta_{//}$, horizontal emitting dipole orientation. **k,l**, Summary of CIEy coordinates versus maximum EQE (**k**) values and EQE at 1,000 cd·m^{-2} (**l**) for state-of-the-art blue (CIEy ≤ 0.20) and green (CIEy ≥ 0.71) OLEDs.

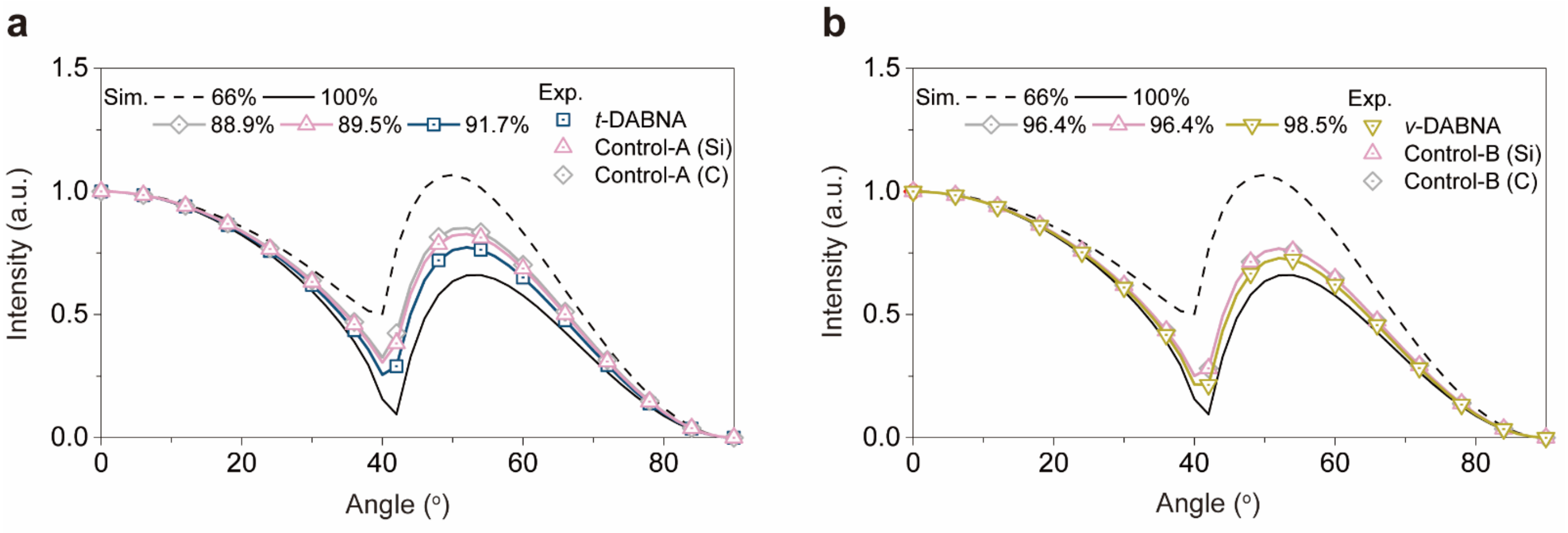

Supplementary Fig. 32. Angle-dependent PL spectra. (**a**) *t*-DABNA-doped films. (**b**) *υ*-DABNA-doped films.

Comment 5: *For the host material, it is fundamental that the authors provide a comprehensive analysis of the transport properties, encompassing either a complete single-carrier device analysis or a mobility analysis.*

Response: We thank the reviewer for the insightful comment. To address it, we have performed a single-carrier transport analysis by fabricating both hole-only (HOD) and electron-only devices (EOD) based on the GeCzCz:GeTrzCz2 host (Supplementary Fig. 20). The HOD structure was ITO/HATCN (5 nm)/GeCzCz:GeTrzCz2 (100 nm, 0.50/0.50 w/w)/HATCN (20 nm)/Al (100 nm), and the EOD structure was ITO/Al_2O_3 (30 nm)/Liq (2 nm)/GeCzCz:GeTrzCz2 (100 nm, 0.50/0.50 w/w)/Liq (2 nm)/Al (100 nm). In the space-charge-limited current (SCLC) region, the current density-voltage characteristics can be expressed by the equation:

$$J = \frac{9}{8}\varepsilon\varepsilon_0\mu\frac{V^2}{L^3} \qquad (2)$$

where J is the current density, V is the applied voltage, ε is the relative dielectric constant of the host, ε_0 is the free-space permittivity, μ is the carrier mobility, and L is the thickness of the host material. By fitting the experimental J-V curves to this equation, we determined the hole and electron mobilities (μ_h and μ_e) of the GeCzCz:GeTrzCz2 host. The estimated μ_h and μ_e are around 3.07×10^{-5} and 5.69×10^{-5} $cm^2 \cdot V^{-1} \cdot s^{-1}$, indicating balanced and efficient charge transport for the GeCzCz:GeTrzCz2 host.

Additional sentence, (manuscript, page 9): "From single-carrier current-voltage measurements (Supplementary Fig. 20) it is demonstrated that the GeCzCz:GeTrzCz2 (0.50/0.50 w/w) host provides balanced and efficient charge transport, with a hole mobility of 3.07×10^{-5} $cm^2 \cdot V^{-1} \cdot s^{-1}$ and an electron mobility

of 5.69×10^{-5} $cm^2 \cdot V^{-1} \cdot s^{-1}$."

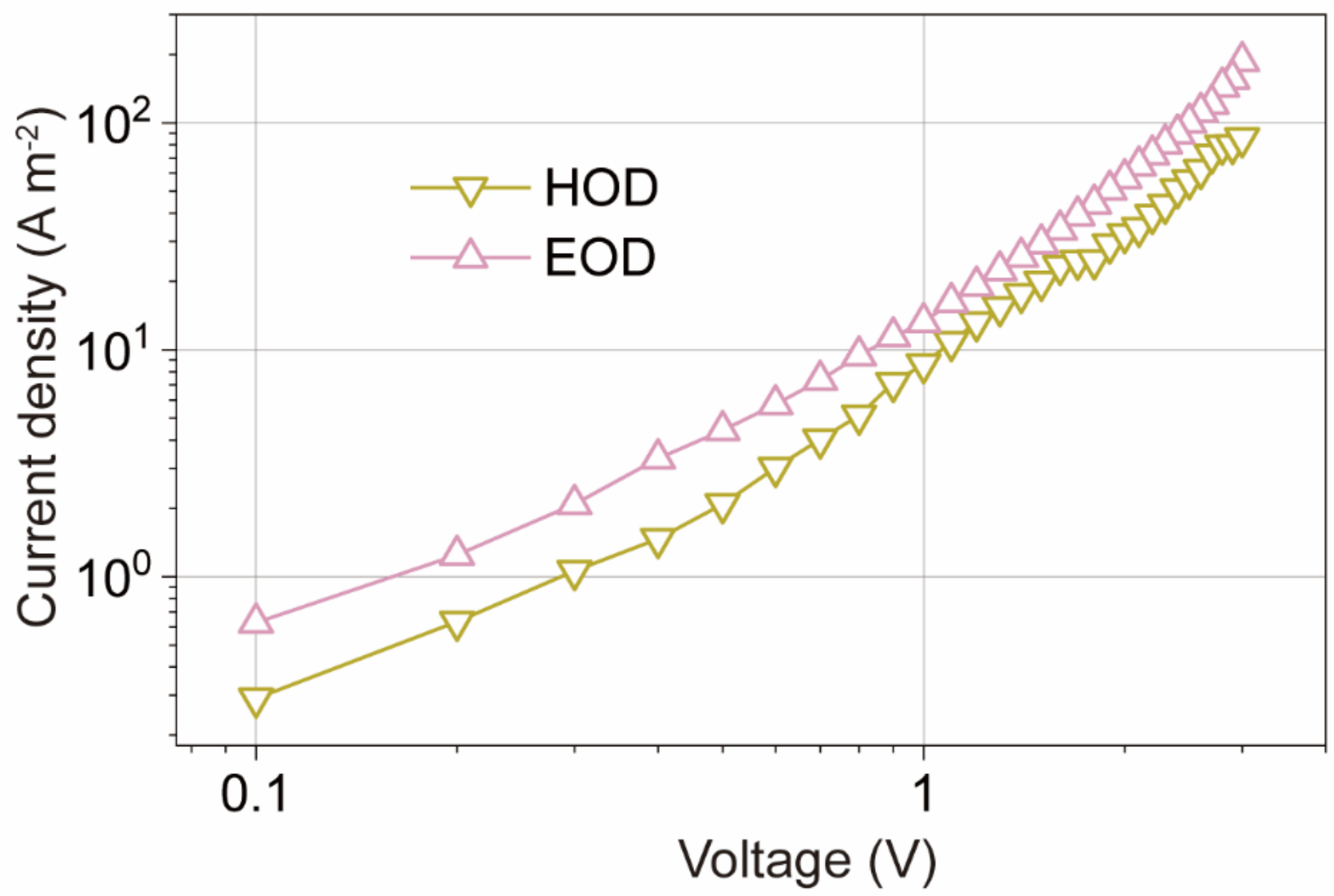

Supplementary Fig. 20. Current density-voltage characteristics of the hole-only device (HOD) and electron-only device (EOD). The device structure of the HOD and EOD were constructed as ITO/HATCN (5 nm)/GeCzCz:GeTrzCz2 (100 nm, 0.50/0.50 w/w)/HATCN (20 nm)/Al (100 nm) and ITO/Al_2O_3 (30 nm)/Liq (2 nm)/GeCzCz:GeTrzCz2 (100 nm, 0.50/0.50 w/w)/Liq (2 nm)/Al (100 nm), respectively.

Comment 6: *The author 's statement for the comparison of efficiency roll-off appears to be questionable. A more appropriate approach would be to compare the percentage of decay in efficiency at the corresponding luminance, as opposed to the efficiency value at the corresponding luminance, as the latter does not accurately reflect the advantages of the introduction of germanium.*

Response: We appreciate the reviewer's constructive suggestion. In response, we have revised the manuscript to compare the percentage decrease in efficiency at 1,000 $cd \cdot m^{-2}$. With the introduction of germanium, the efficiency roll-off percentages at 1,000 $cd \cdot m^{-2}$ are 18.2% for the blue device (*υ*-DABNA) and 17.3% for the green device (*ω*-DABNA), both of which are lower than those of the silicon-based and carbon-based control devices.

Modified sentence, (manuscript, page 11): "At 1,000 cd m^{-2}, these devices retained EQE values of 32.8% (*υ*-DABNA) and 33.4% (*ω*-DABNA), corresponding to a 18.2% and 17.3% decrease in EQE, respectively. These roll-off characteristics outperformed those of Control-B (34.3% and 46.3% efficiency decreases for silicon-based and carbon-based devices, respectively) and Control-C (30.0% and 34.2%, respectively) at equivalent brightness levels."

Comment 7: *In order to facilitate a more substantial scientific study, it is recommended that the author synthesizes selenium-based comparison molecules. This would enable a more effective comparison of the effects of heavy atom effects, such as carbon, silicon, germanium and selenium, on the exciplex host materials.*

Response: We thank the reviewer for this valuable suggestion. Since selenium (Group 16) does not belong to the same group as carbon/silicon/germanium (Group 14) in the periodic table, we instead synthesized CTrzCz2 and CCzCz to construct carbon-based exciplex hosts, enabling a more appropriate and effective comparison of heavy atom effects.

Modified sentences (Manuscript, page 9): "…and carbon-based counterparts (CCzCz and CTrzCz2, Supplementary Figs. 21-29)…"

Modified sentences (Manuscript, page 11): "…compared to SiCzCz:SiTrzCz2-based and CCzCz:CTrzCz2-based ones (Control A-C). The *t*-DABNA-based device achieved a maximum EQE of 30.1% (Fig. 4d), surpassing Control-A (silicon, 26.9%; carbon, 16.8%)."

Modified sentence, (manuscript, page 11): "At 1,000 cd m^{-2}, these devices retained EQE values of 32.8% (*υ*-DABNA) and 33.4% (*ω*-DABNA), corresponding to a 18.2% and 17.3% decrease in EQE, respectively. These roll-off characteristics outperformed those of Control-B (34.3% and 46.3% efficiency decreases for silicon-based and carbon-based devices, respectively) and Control-C (30.0% and 34.2%, respectively) at equivalent brightness levels."

Additional sentence, (manuscript, page 11): "Next, we assessed the operational stability of these devices at an initial luminance of 1,000 cd·m^{-2}, as shown in Fig. 4g-i. The CCzCz:CTrzCz2-based devices exhibited the poorest stability, with operational half-lifetimes (LT_{50}) of 0.2-0.5 h. The SiCzCz:SiTrzCz2-based devices revealed improved stability, achieving LT_{50} values of 9.0 h, 24.6 h, and 207.8 h for *t*-DABNA, *υ*-DABNA, and *ω*-DABNA, respectively. The GeCzCz:GeTrzCz2-based devices demonstrated the best overall performance (LT_{50} values of 8.8 h, 29.3 h, and 241.1 h for *t*-DABNA, *υ*-DABNA, and *ω*-DABNA, respectively). Notably, when *t*-DABNA was employed as the emitter, the germanium-based and silicon-based devices showed comparable lifetimes, likely because the high emission energy of *t*-DABNA compromises the chemical stability of both exciplex hosts. In contrast, for guests with reduced emission energies (*ν*-DABNA and *ω*-DABNA), the GeCzCz:GeTrzCz2-based devices displayed longer lifetimes than the silicon-based

control devices, due to the decreased triplet population associated with the germanium-based exciplex host.”

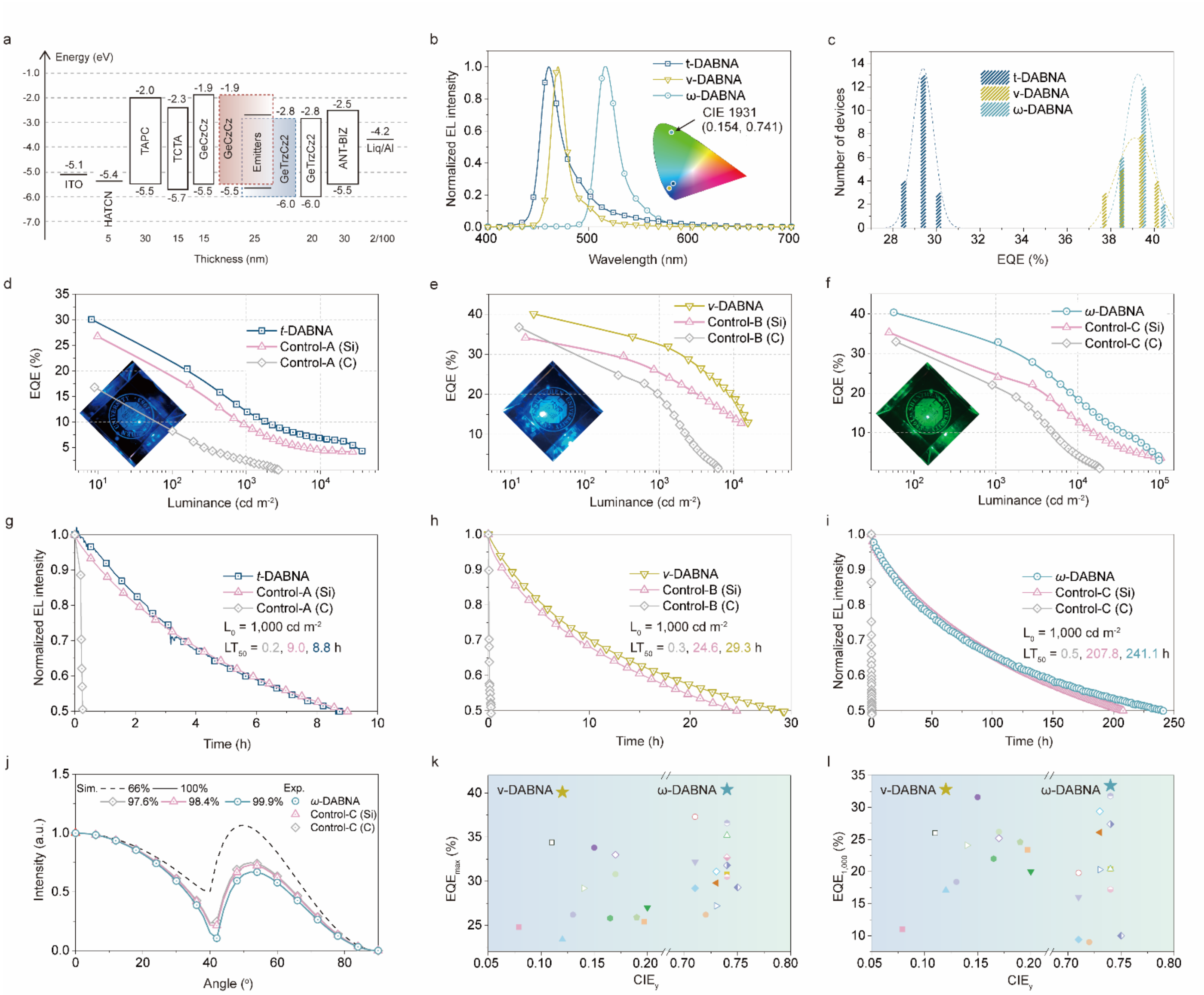

Fig. 4 | Optimized EL properties of OLEDs. a, Diagram of the germanium-based device structure. **b**, EL specrtra recorded at 1,000 cd·m^{-2}. c, Statistical histogram of EQEs for different devices. **d-f**, EQE versus luminance characteristics of target devices (*t*-DABNA (**d**), *υ*-DABNA (**e**), *ω*-DABNA (**f**)) and control devices (control-A (**d**), control-B (**e**), control-C (**f**)). **g**, Half-lifetime (LT_{50}) measurement of *t*-DABNA-based device and control devices (control-A) at an initial luminance of 1,000 cd·m^{-2}. **h**, LT_{50} measurement of *υ*-DABNA-based device and control devices (control-B) at an initial luminance of 1,000 cd·m^{-2}. **i**, LT_{50} measurement of *ω*-DABNA-based device and control devices (control-C) at an initial luminance of 1,000 cd·m^{-2}. **j**, Angle-dependent PL spectra of *ω*-DABNA-doped films. $\Theta_{//}$, horizontal emitting dipole orientation. **k**,**l**, Summary of CIEy coordinates versus maximum EQE (**k**) values and EQE at 1,000 cd·m^{-2} (**l**) for state-of-the-art blue (CIEy ≤ 0.20) and green (CIEy ≥ 0.71) OLEDs.

Table 1 | Key data of the devices

Device	CE (cd·A^{-1})		PE (lm W^{-1})		EQE (%)		
	**Max (average)**	**1,000 cd m^{-2}**	**Max (average)**	**1,000 cd m^{-2}**	**Max (average)**	**1,000 cd m^{-2}**	**5,000 cd m^{-2}**
t-DABNA	38.1 (35.6±1.3)	14.4	42.7 (39.9±1.4)	10.6	30.1 (29.3±0.5)	12.0	7.6
Control-A (Si)	38.7 (37.1±1.9)	10.2	43.5 (41.6±2.2)	6.0	26.9 (26.5±0.5)	7.5	4.5
Control-A (C)	22.6 (20.6±2.3)	3.3	25.4 (23.1±2.5)	1.6	16.8 (15.9±0.7)	2.5	-
υ-DABNA	43.6 (37.4±1.6)	30.4	48.9 (38.8±3.3)	27.7	40.1 (39.2±0.9)	32.8	24.4
Control-B (Si)	35.8 (32.3±2.9)	23.1	40.2 (35.2±4.6)	18.5	34.1 (33.1±1.1)	22.4	14.6
Control-B (C)	31.8 (29.3±2.4)	17.1	35.7 (32.8±2.7)	13.3	36.7 (34.4±2.0)	19.7	3.2
ω-DABNA	145.5 (142.1±2.3)	118.6	163.2 (159.4±2.6)	117.8	40.4 (39.3±0.7)	33.4	23.9
Control-C (Si)	130.7 (124.5±5.0)	87.3	146.6 (139.7±5.6)	79.4	35.3 (34.3±0.8)	24.7	10.5
Control-C (C)	121.3 (117.1±3.2)	78.8	136.1 (131.4±3.6)	76.6	33.0 (31.7±1.0)	21.7	9.9

The average device parameters in parentheses are based on the measurement of twenty independent devices.

Comment 8: *The comparisons in Supplementary Table 4 appear to lack rational coherence. I would recommend dropping the relevant argument and that table.*

Response: We thank the reviewer for this comment. In response, we have removed original Supplementary Table 4 and the associated discussion in the manuscript.

Reviewer #2:

General comment: *The comparative analysis and logical discussion of replacing silicon exciplexes with germanium exciplexes, which form the core of this paper, are critically insufficient. Therefore, this paper is deemed unsuitable for publication in Nature Communications in its current form. The difference in photophysical properties observed between germanium and silicon exciplex systems is negligible and not significant enough to warrant special consideration. The manuscript lacks compelling evidence to support the proposed contribution of heavy atom effect. Furthermore, the lack of direct comparison of OLED performance in germanium and silicon exciplex systems weakens the authors' claims. Therefore, it cannot be concluded that the germanium exciplex system described in this study represents a significant advance over the previously reported silicon exciplex system. Therefore, the manuscript does not meet the criteria for originality and novelty for publication in Nature Communications. If this is to be published elsewhere, the following points should be further considered to provide sufficient supporting evidence.*

Response: We appreciate the reviewer's critical comments and constructive suggestions. We respectfully disagree with the assessment that the differences between germanium and silicon exciplex systems are negligible. In fact, the germanium-based exciplex exhibits a 1.9-fold enhanced radiative decay rate, a 1.5-fold reduced non-radiative decay rate, and thus a 1.8-fold higher photoluminescence quantum yield compared to the silicon analogue. Furthermore, when employed as the host, the germanium exciplex accelerates the triplet spin-flip process of the MR-TADF emitter by more than twofold.

Importantly, these improvements translate into significant performance enhancements in electroluminescent devices. At a practical brightness of 1,000 cd·m^{-2}, the germanium-based devices exhibit clearly improved efficiency and markedly reduced efficiency roll-off compared to the silicon-based devices. Moreover, devices employing the germanium exciplex host achieve comparable or improved operational stability compared to those based on the silicon exciplex host. To further substantiate the proposed contribution of heavy atom effect, we have included a three-way comparison with a carbon-based exciplex system in the updated manuscript, which progressively shows enhanced performance from carbon to silicon, and further to germanium.

Taken together, the improvements in efficiency, efficiency roll-off, and stability support our conclusion that the germanium exciplex system represents a substantive advance over the previously reported silicon exciplex host. We hope the additional experimental evidence have thoroughly addressed the reviewer's concerns and demonstrated the manuscript's suitability for publication in *Nature Communications*.

Comment 1: *To claim that energy transfer primarily occurs in the manner shown in Figure 1a, further discussion on whether the exciplex system effectively suppresses DET transfer is necessary. The DET rate constant should be analyzed.*

Response: We thank the reviewer for this constructive comment. To determine whether the exciplex system suppresses Dexter energy transfer (DET), we calculated the rate of DET (k_{DET}) by comparing the transient PL decay curves of the host emission measured with and without the guest emitters (see Supplementary Note 3 and Supplementary Fig. 19). Our analysis shows that the germanium-based exciplex host is more effectively than the silicon-based reference host to suppresses DET. Specifically, in GeCzCz:GeTrzCz2 host, the k_{DET} values for *t*-DABNA, *υ*-DABNA, and *ω*-DABNA were reduced by factors of 1.33, 1.70, and 3.05, respectively, compared with SiCzCz:SiTrzCz2 host (Supplementary Table 2). These results support our claim that energy transfer in our germanium exciplex system occurs predominantly via the pathway shown in Fig. 1a.

Additional sentence, (manuscript, page 10): "Crucially, in the GeCzCz:GeTrzCz2 host, the rate of Dexter energy transfer (k_{DET}) for *t*-DABNA, *υ*-DABNA, and *ω*-DABNA were reduced by factors of 1.33, 1.70, and 3.05, respectively, compared with SiCzCz:SiTrzCz2 host (Supplementary Fig. 19, Supplementary Table 2 and Supplementary Note 3), supporting the fact that the germanium-based exciplex host suppress Dexter energy transfer."

Additional section, (Supplementary Information, Supplementary Note 3):

"**Supplementary Note 3. Calculation method of k_{DET}.**

In the presence of the MR-TADF emitters, the respective rate constants of the prompt and delayed fluorescence components (k_p and k_d) occurring in the exciplex host, can be given by:

$$k_p, k_d = \frac{k_{FET} + k_{r,S} + k_{nr,S} + k_{ISC} + k_{DET} + k_{r,T} + k_{nr,T} + k_{RISC}}{2} \times \left(1 \pm \sqrt{1 - \frac{4(k_{FET} + k_{r,S} + k_{nr,S} + k_{ISC})(k_{DET} + k_{r,T} + k_{nr,T} + k_{RISC}) - 4k_{ISC}k_{RISC}}{(k_{FET} + k_{r,S} + k_{nr,S} + k_{ISC} + k_{DET} + k_{r,T} + k_{nr,T} + k_{RISC})^2}}\right). \quad (10)$$

Using Vieta's formula, the sum of k_p and k_d as well as the product of k_p and k_d can be expressed as:

$$k_p + k_d = k_{FET} + k_{r,S} + k_{nr,S} + k_{ISC} + k_{DET} + k_{r,T} + k_{nr,T} + k_{RISC}. \quad (11)$$

$$k_p k_d = (k_{FET} + k_{r,S} + k_{nr,S} + k_{ISC})(k_{DET} + k_{r,T} + k_{nr,T} + k_{RISC}) - k_{ISC}k_{RISC}. \quad (12)$$

By employing approximations, k_{DET} can be simplified as follows:

$$k_{DET} = \frac{1}{\tau_{d,1}} - \frac{1}{\tau_{d,0}} + k_{ISC}k_{RISC}(\tau_{p,1} - \tau_{p,0}). \quad (13)$$

$\tau_{p,1}$ and $\tau_{p,0}$ represent prompt fluorescence decay times with or without guests, respectively. Likewise, $\tau_{d,1}$ and $\tau_{d,0}$ represent delayed fluorescence decay times with or without guests, respectively."

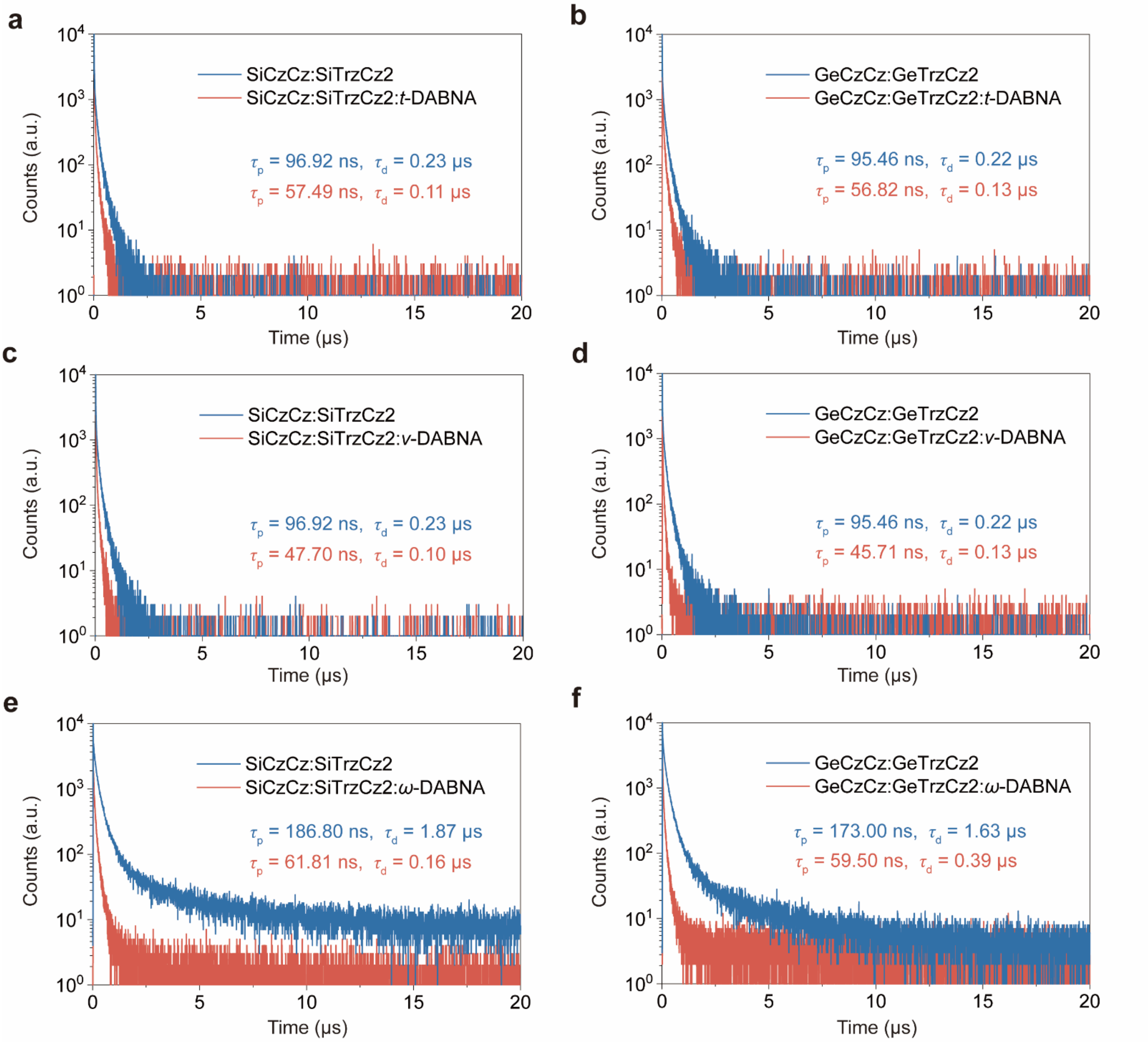

Supplementary Fig. 19. Transient PL decay curves. (a) The films with or without *t*-DABNA doping in the SiCzCz:SiTrzCz2 matrix at the emission wavelength of 468 nm. (b) The films with or without *t*-DABNA doping in the GeCzCz:GeTrzCz2 matrix at the emission wavelength of 468 nm. (c) The films with or without *v*-DABNA doping in the SiCzCz:SiTrzCz2 matrix at the emission wavelength of 425 nm. (d) The films with or without *v*-DABNA doping in the GeCzCz:GeTrzCz2 matrix at the emission wavelength of 425 nm. (e) The films with or without *ω*-DABNA doping in the SiCzCz:SiTrzCz2 matrix at the emission wavelength of 425 nm. (f) The films with or without *ω*-DABNA doping in the GeCzCz:GeTrzCz2 matrix at the emission wavelength of 425 nm.

Supplementary Table 2. Physical data and kinetic parameters of MR-TADF emitters *t*-DABNA, *υ*-DABNA and *ω*-DABNA doped in exciplex hosts GeCzCz:GeTrzCz2 and SiCzCz:SiTrzCz2.

Film	τ_p / ns	τ_d / µs	k_{RISC} / s^{-1}	k_{DET} / s^{-1}
**GeCzCz:GeTrzCz2:*t*-DABNA**	10.7	112.3	2.2×10^4	3.3×10^6
**SiCzCz:SiTrzCz2:*t*-DABNA**	11.9	133.0	1.3×10^4	4.4×10^6
**GeCzCz:GeTrzCz2:*υ*-DABNA**	5.3	2.4	9.1×10^5	3.3×10^6
**SiCzCz:SiTrzCz2:*υ*-DABNA**	5.5	4.0	4.5×10^5	5.6×10^6
**GeCzCz:GeTrzCz2: *ω*-DABNA**	6.3	3.6	7.5×10^5	1.9×10^6
**SiCzCz:SiTrzCz2:*ω*-DABNA**	6.4	7.7	3.6×10^5	5.8×10^6

Comment 2: *The absence of an explanation for the threefold increase in SOCME raises some concerns. It would be valuable to provide data on the T_1 NTO transition (hole to electron) for both the Si and Ge versions. Given that Si and Ge are positioned in sp^3 hybridized sites, a more detailed NTO analysis would help to substantiate the presence of the heavy atom effect. Additionally, it would be appreciated clarification on whether the upper excited state levels and the corresponding SOCME have been analyzed.*

Response: We appreciate the reviewer for the insightful comments. To address these concerns, we calculated the excited-state energies and corresponding spin-orbit coupling matrix elements (SOCMEs) for both SiCzCz:SiTrzCz2 and GeCzCz:GeTrzCz2. The excited state energies are nearly identical between the two systems, consistent with the experimental trend. In contrast, the SOCMEs between S_1 and T_1 (or T_2) are significantly larger for the germanium system (Supplementary Fig. 17). Specifically, the values of $\langle S_1|\hat{H}_{SOC}|T_1\rangle$ and $\langle S_1|\hat{H}_{SOC}|T_2\rangle$ for GeCzCz:GeTrzCz2 are 0.791 and 0.174 cm^{-1}, representing approximately threefold and over twofold enhancements compared to SiCzCz:SiTrzCz2 ($\langle S_1|\hat{H}_{SOC}|T_1\rangle$ = 0.265 cm^{-1} and $\langle S_1|\hat{H}_{SOC}|T_2\rangle$ = 0.080 cm^{-1}), respectively.

To elucidate the mechanism underlying this enhancement, we analyzed the natural transition orbitals (NTO) for the spin-flip process between S_1 and T_1 in both systems. The NTO analysis reveals that although Si and Ge occupy sp^3 hybridized sites, they exhibit hyperconjugation effect with the π-electron system of the aromatic backbone. This hyperconjugation effect provides a pathway for spin-orbit coupling. Atomic and fragment contribution analysis of the SOCME confirms that the germanium atom contributes predominantly to the enhanced spin-orbit coupling in GeCzCz:GeTrzCz2, directly substantiating the heavy atom effect as the origin of the SOCME enhancement.

Additional sentence, (manuscript, page 6): "Analysis of spin-orbit coupling matrix elements (SOCMEs) in the optimized T_1 geometry showed an approximately threefold increase in $<S_1|\hat{H}_{SOC}|T_1>$ from 0.265 cm^{-1} (SiCzCz:SiTrzCz2) to 0.791 cm^{-1} (GeCzCz:GeTrzCz2), consistent with the expected enhancement in spin-orbit coupling due to the higher atomic number (Supplementary Fig. 17). Similar trends are observed for the upper excited state (T_2): $<S_1|\hat{H}_{SOC}|T_2>$ reaches 0.174 cm^{-1} for GeCzCz:GeTrzCz2, compared to 0.080 cm^{-1} for SiCzCz:SiTrzCz2."

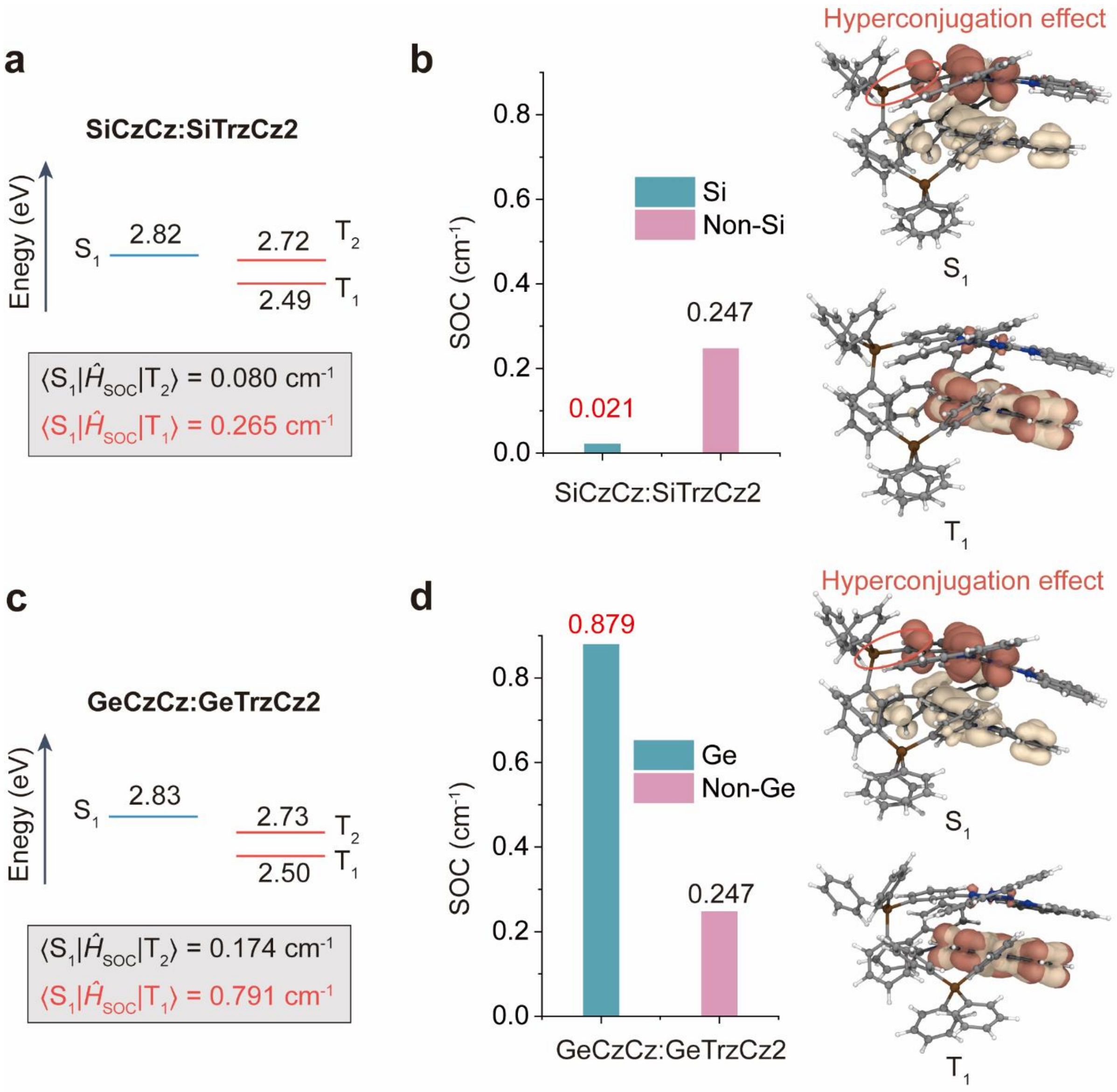

Supplementary Fig 17. Theoretical analysis of excited states. (a) Calculated transition energies and spin-orbit coupling (SOC) constants for SiCzCz:SiTrzCz2. (b) Atomic and fragment contributions to SOC, together with natural transition orbitals (NTOs) for SiCzCz:SiTrzCz2. (c) Calculated transition energies and SOC constants for GeCzCz:GeTrzCz2. (d) Atomic and fragment contributions to SOC, together with NTOs for GeCzCz:GeTrzCz2.

Comment 3: *According to the SI Table 1 fitting results, there appear to be no significant differences in the key rate constants related to the heavy atom effect, so additional explanation on this point is necessary. Also, how can the approximately 1.8-fold increase in PLQY be explained? Specifically, an explanation is needed for why such a difference occurs despite no significant differences in the rates of other radiative and non-radiative transitions.*

Response: We appreciate the reviewer for the professional comments. In fact, there are significant differences in the key rate constants related to the heavy atom effect. Specifically, compared to the silicon-based exciplex system (SiCzCz:SiTrzCz2), the germanium-based exciplex (GeCzCz:GeTrzCz2) exhibits a 1.9-fold increase in the singlet radiative decay rate ($k_{r,S}$) and a 1.5-fold decrease in the singlet non-radiative decay rate ($k_{nr,S}$). According to the relationship ($PLQY = \frac{k_{r,S}}{k_{r,S} + k_{nr,S}}$), both the increase in $k_{r,S}$ and the decrease in $k_{nr,S}$ contribute positively to the PLQY. The combined effect of these two changes results in the observed 1.8-fold enhancement in PLQY for the germanium exciplex system.

The reduction in non-radiative decay is primarily attributed to increased molecular packing density in the germanium-based exciplex system. This interpretation is also supported by recent literature (*Nat. Commun.* **16**, 4446 (2025)), and is consistent with the higher crystal densities (Supplementary Figs. 9 and 10) of GeCzCz and GeTrzCz2 compared with SiCzCz and SiTrzCz2, respectively.

Modified sentences (Manuscript, page 5): "…and that GeCzCz and GeTrzCz2 exhibit increased crystal densities, suggesting tighter molecular packing suppress non-radiative decay."

Modified sentences (Manuscript, page 6): "…singlet non-radiative decay rate ($k_{nr,S}$) and RISC rate (k_{RISC}), were determined (Supplementary Note 2 and Supplementary Table 1)[29-31]. Compared to the silicon-based reference (SiCzCz:SiTrzCz2), the germanium-based exciplex exhibited a 1.9-fold enhanced $k_{r,S}$ of 2.5×10^6 s^{-1} and a 1.5-fold reduced $k_{nr,S}$ of 2.1×10^6 s^{-1}, which together led to a 1.8-fold higher PL quantum yield (54% vs. 30%)."

Comment 4: *In the device results shown in Figure 4 and Table 1, the EQE is particularly high at the driving voltage, but then drops sharply. An explanation for this behavior is needed. It is also necessary to provide an explanation for why the roll-off is so massive.*

Response: We appreciate the reviewer's professional comments regarding the device efficiency roll-off. The sharp EQE drop at higher voltages is an intrinsic characteristic of sensitizer-free OLEDs. As widely reported

in the existing literature (e.g., *Angew. Chem. Int. Ed.* **64**, e202504002 (2025); *Adv. Mater.* **36**, 2412761 (2024); *Angew. Chem. Int. Ed.* **62**, e202306413 (2023); *Adv. Funct. Mater.* **31**, 2102017 (2021)), such devices without sensitizer typically exhibit substantial efficiency decreases, often ranging from 47% to 69% at a luminance of 1000 cd·m^{-2}. While this behavior is common across the sensitizer-free OLEDs, it is important to note that our germanium-based devices exhibit significantly suppressed efficiency roll-off at 1000 cd·m^{-2} (18.2% for *v*-DABNA and 17.3% for *ω*-DABNA) compared to both silicon-based (34.3% and 30.0%, respectively) and carbon-based control devices (46.3% and 34.2%, respectively), validating the effectiveness of our design strategy.

Comment 5: *All the device lifetime data mentioned in the manuscript should be provided. In particular, comparison with the Si exciplex devices is necessary to demonstrate the differences in the external heavy atom effect originating from the host. Additionally, the differences in roll-off efficiency should be addressed.*

Response: We appreciate the reviewer for this constructive suggestion. In accordance with this suggestion, we have incorporated all device operational lifetime data mentioned in the manuscript (including the carbon-based control device suggested by Reviewer #1) as Fig. 4g-i.

Specifically, the *t*-DABNA-, *v*-DABNA- and *ω*-DABNA-based devices using GeCzCz:GeTrzCz2 as the host demonstrate operational half-lifetime (LT_{50}) of 8.6 h, 29.3 h, 241.1 h at an initial brightness (L_0) of 1,000 cd·m^{-2}, which are significantly superior to those of control devices incorporating CCzCz:CTrzCz2 as host (LT_{50} values of 0.2-0.5 h). This comparison supports that host-originated external heavy-atom effect effectively improves device operational stability.

In comparison with the silicon control devices, the *t*-DABNA-based germanium exciplex device exhibits comparable stability, likely because the high emission energy of the guest (*t*-DABNA) compromises the chemical stability of both exciplex hosts. In contrast, for guests with reduced emission energies (*v*-DABNA and *ω*-DABNA), the GeCzCz:GeTrzCz2-based devices show longer lifetimes than the silicon-based control devices (LT_{50} value of 24.6 h and 207.8 h, respectively), due to the decreased triplet population associated with the germanium-based exciplex host.

Furthermore, the germanium exciplex-based devices exhibit significantly suppressed efficiency roll-off at 1,000 cd·m^{-2} (18.2% for *v*-DABNA and 17.3% for *ω*-DABNA) compared to both silicon-based (34.3% and 30.0%, respectively) and carbon-based control devices (46.3% and 34.2%, respectively). We attribute this improvement to the synergistic effect of external heavy atom effect and complete host-to-guest energy transfer, which effectively reduces the triplet accumulation and thereby suppresses efficiency roll-off.

Additional sentence, (manuscript, page 11): "Next, we assessed the operational stability of these devices at an initial luminance of 1,000 cd·m^{-2}, as shown in Fig. 4g-i. The CCzCz:CTrzCz2-based devices exhibited the poorest stability, with operational half-lifetimes (LT_{50}) of 0.2-0.5 h. The SiCzCz:SiTrzCz2-based devices revealed improved stability, achieving LT_{50} values of 9.0 h, 24.6 h, and 207.8 h for *t*-DABNA, *υ*-DABNA, and *ω*-DABNA, respectively. The GeCzCz:GeTrzCz2-based devices demonstrated the best overall performance (LT_{50} values of 8.8 h, 29.3 h, and 241.1 h for *t*-DABNA, *υ*-DABNA, and *ω*-DABNA, respectively). Notably, when *t*-DABNA was employed as the emitter, the germanium-based and silicon-based devices showed comparable lifetimes, likely because the high emission energy of *t*-DABNA compromises the chemical stability of both exciplex hosts. In contrast, for guests with reduced emission energies (*ν*-DABNA and *ω*-DABNA), the GeCzCz:GeTrzCz2-based devices displayed longer lifetimes than the silicon-based control devices, due to the decreased triplet population associated with the germanium-based exciplex host."

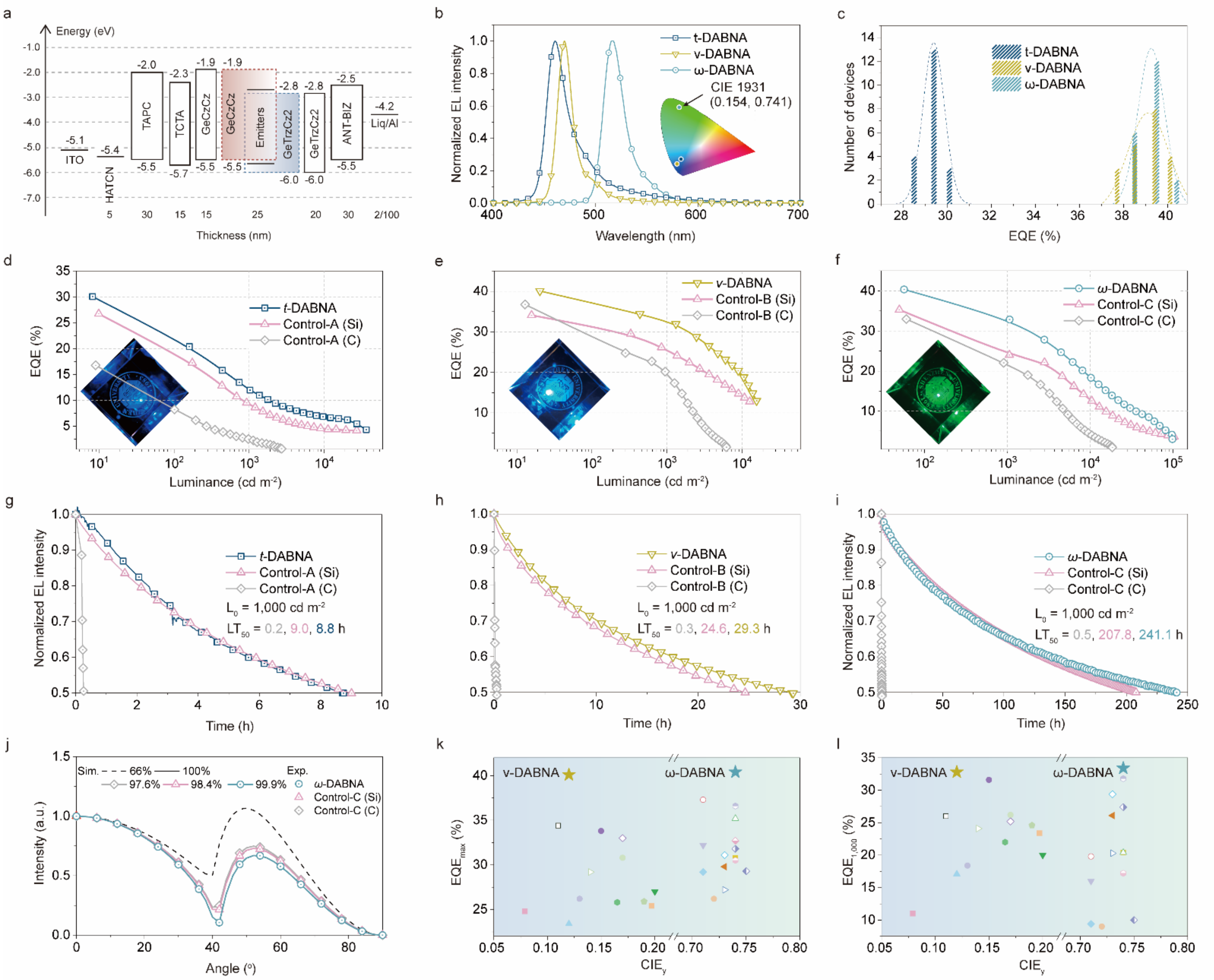

Fig. 4 | Optimized EL properties of OLEDs. a, Diagram of the germanium-based device structure. **b**, EL specrtra recorded at 1,000 cd·m^{-2}. c, Statistical histogram of EQEs for different devices. **d-f**, EQE versus luminance characteristics of target devices (*t*-DABNA (**d**), *υ*-DABNA (**e**), *ω*-DABNA (**f**)) and control devices (control-A (**d**), control-B (**e**), control-C (**f**)). **g**, Half-lifetime (LT_{50}) measurement of *t*-DABNA-based device and control devices (control-A) at an initial luminance of 1,000 cd·m^{-2}. **h**, LT_{50} measurement of *υ*-DABNA-based device and control devices (control-B) at an initial luminance of 1,000 cd·m^{-2}. **i**, LT_{50} measurement of *ω*-DABNA-based device and control devices (control-C) at an initial luminance of 1,000 cd·m^{-2}. **j**, Angle-dependent PL spectra of *ω*-DABNA-doped films. $\Theta_{//}$, horizontal emitting dipole orientation. **k**,**l**, Summary of CIEy coordinates versus maximum EQE (**k**) values and EQE at 1,000 cd·m^{-2} (**l**) for state-of-the-art blue (CIEy ≤ 0.20) and green (CIEy ≥ 0.71) OLEDs.

Comment 6: *It would be beneficial to examine the CV reproducibility difference when substituting Si with Ge. Analyzing the increased stability of holes and electrons within the exciplex system would provide a crucial basis for explaining the improved device stability presented in Figure 4g.*

Response: We appreciate the reviewer's insightful suggestion. We have repeated the cyclic voltammetry (CV) measurements for the HT hosts (SiCzCz and GeCzCz) and the ET hosts (SiTrzCz2 and GeTrzCz2) for 20 consecutive cycles to evaluate CV reproducibility (Supplementary Fig. 11). After multiple scans, both the HT and ET hosts exhibit reversible oxidation/reduction behavior, and their oxidation potentials remain essentially unchanged, demonstrating good CV reproducibility and indicating stable hole-related redox processes. We have also examined the reduction potentials of the ET hosts and find that the reduction processes remain similarly reproducible for SiTrzCz2 and GeTrzCz2.

Additional sentence, (manuscript, page 5): "Repeated cyclic voltammetry scans (Supplementary Fig. 11) further indicated that substituting silicon with germanium results in comparable electrochemical stability for both the ET hosts (SiTrzCz2 versus GeTrzCz2) and the HT hosts (SiCzCz versus GeCzCz)."

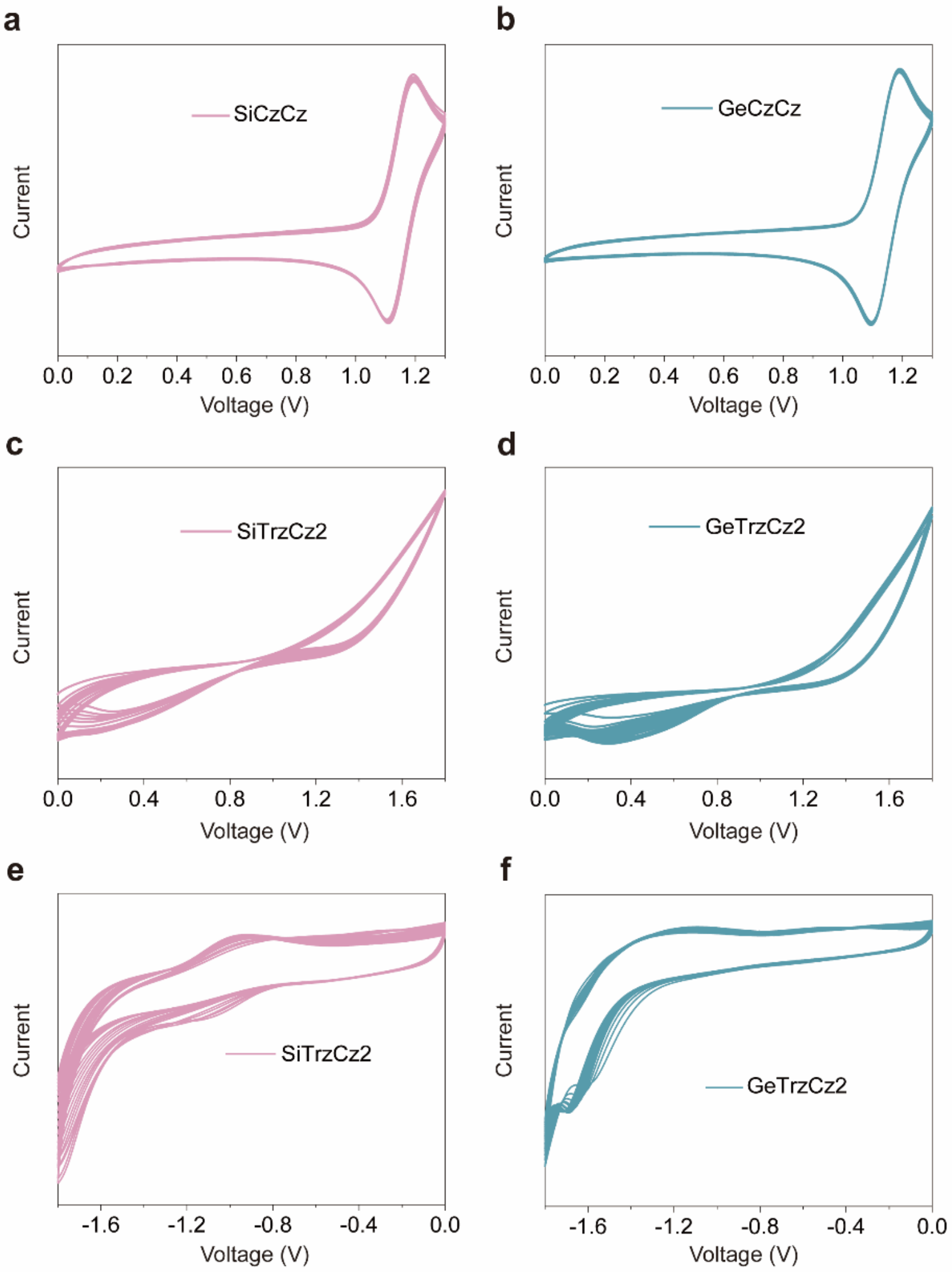

Supplementary Fig 11. Cyclic voltammograms of the hosts. Oxidation waves of (**a**) SiCzCz, (**b**) GeCzCz, (**c**) SiTrzCz2, (**d**) GeTrzCz2 and reduction waves of (**e**) SiTrzCz2, (**f**) GeTrzCz2.

Comment 7: *It would be interesting to know whether there is a difference in PL stability arising from the heavy atom effect in the thin doped films. PL stability should be measured for the emitter in both Si and Ge hosts.*

Response: We appreciate the reviewer for the constructive suggestion. To examine whether the heavy-atom effect influences photostability, we have measured the photoluminescence stability of *t*-DABNA, *υ*-DABNA, and *ω*-DABNA-doped thin films in both Si and Ge hosts under continuous 365 nm LED irradiation (12 W) at room temperature (Supplementary Fig. 33). For both hosts, the emission spectral shapes remain unchanged during irradiation, and the percentage of the charge-transfer emission band at the PL peak is basically the same, indicating comparable PL stability of the doped films in Si and Ge hosts under these conditions.

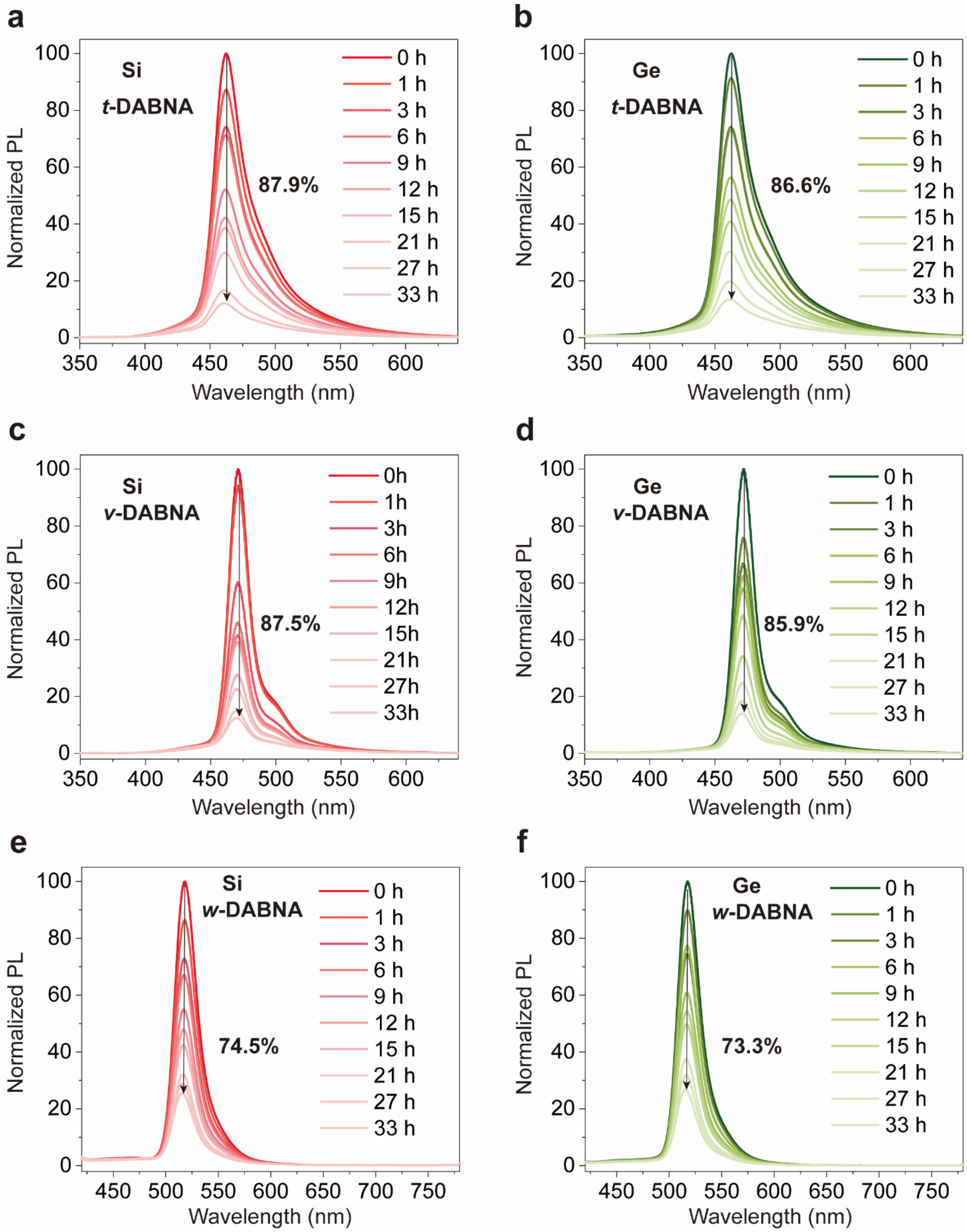

Supplementary Fig. 33. Photolysis results. PL spectra of (**a**) SiCzCz:SiTrzCz2:*t*-DABNA, (**b**) GeCzCz:GeTrzCz2:*t*-DABNA, (**c**) SiCzCz:SiTrzCz2:*υ*-DABNA, (**d**) GeCzCz:GeTrzCz2:*υ*-DABNA, (**e**) SiCzCz:SiTrzCz2:*ω*-DABNA, (**f**) GeCzCz:GeTrzCz2:*ω*-DABNA over 33 h under continuous 365 nm LED irradiation (12 W) at room temperature.

Comment 8: *The substitution of Si with Ge should be further analyzed by comparing the Transient-PL data to examine the differences. It would be better to present the comparison of the prepared data in a single figure for more effective analysis. Furthermore, the manuscript or SI should provide Transient-PL data related to prompt fluorescence.*

Response: We are grateful to the reviewer for this insightful comment. Following this advice, we have now presented the comparison of Transient-PL data for GeCzCz:GeTrzCz2 and SiCzCz:SiTrzCz2 (Supplementary Fig. 15), including the corresponding bi-exponential fitting analyses. For GeCzCz:GeTrzCz2, the prompt component exhibits a lifetime of 173.0 ns (accounting for 75.6% of the total intensity), while the delayed component shows a lifetime of 1.6 μs (24.4% intensity ratio). In comparison, SiCzCz:SiTrzCz2 displays a prompt lifetime of 186.8 ns (81.5% intensity ratio) and a delayed lifetime of 1.9 μs (18.5% intensity ratio). Additionally, we have included the transient-PL data related to prompt fluorescence for clarity.

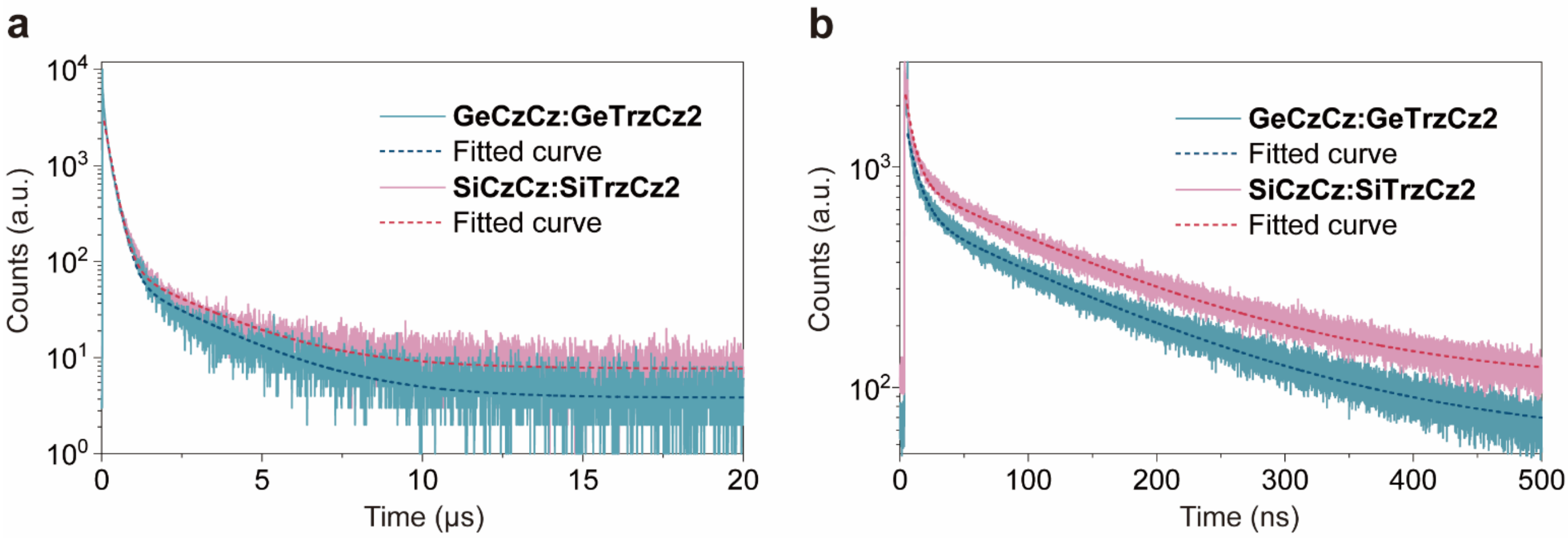

Supplementary Fig. 15. Transient photoluminescence decay curves of exciplex films. GeCzCz:GeTrzCz2 and SiCzCz:SiTrzCz2 films at (**a**) μs-scale and (**b**) ns-scale, theoretical decay curves from bi-exponential fit procedure are shown as dash lines.

Comment 9: *The advantages of this type of Ge-exciplex device, aside from the driving voltage, compared to the currently widely reported HF systems should be clarified. Additionally, the PE and CE values at 1000 cd for the measurement of device stability should be provided.*

Response: We thank the reviewer for this insightful comment. Currently, widely reported hyperfluorescent (HF) devices typically rely on a ternary or even quaternary emissive system, in which a TADF or phosphorescent sensitizer is needed to dope into a single host or an exciplex host and paired with a narrowband fluorescent emitter. Such systems require not only selecting an appropriate sensitizer with a short delayed

lifetime and fast radiative decay, but also carefully optimizing energy transfer from the host to the sensitizer and from the sensitizer to the terminal emitter, as well as matching the sensitizer's HOMO/LUMO and T_1 energy levels and precisely controlling the sensitizer concentration. As a result, fabrication and optimization are relatively complex.

In contrast, our Ge-exciplex devices allow the charge-transport balance and the recombination-zone width to be tuned by adjusting the ratio of the hole-transport and electron-transport host materials. Once charge balance is achieved, the main consideration is the energy transfer between the exciplex host and the emitting dopant, eliminating the additional intermediate transfer step required in HF systems. Therefore, this type of Ge-exciplex device avoids complicated fabrication and stringent control of doping and energy-transfer pathways, and it also reduces potential negative effects caused by energy-level mismatch of sensitizer.

Additionally, as requested, we have also provided the PE and CE values at 1000 cd·m^{-2} in the Table 1.

Modified sentences (Manuscript, page 3): "…an exciplex host with accelerated RISC that maintains balanced exciton transport and enables direct energy transfer to the terminal emitter without intermediate sensitization. This not only reduce the triplet population of MR-TADF emitters without requiring TADF sensitizers, but also deliver exceptional electroluminescent (EL) performance."

Table 1 | Key data of the devices

Device	CE (cd·A^{-1})		PE (lm W^{-1})		EQE (%)		
	Max (average)	1,000 cd m^{-2}	Max (average)	1,000 cd m^{-2}	Max (average)	1,000 cd m^{-2}	5,000 cd m^{-2}
t-DABNA	38.1 (35.6±1.3)	14.4	42.7 (39.9±1.4)	10.6	30.1 (29.3±0.5)	12.0	7.6
Control-A (Si)	38.7 (37.1±1.9)	10.2	43.5 (41.6±2.2)	6.0	26.9 (26.5±0.5)	7.5	4.5
Control-A (C)	22.6 (20.6±2.3)	3.3	25.4 (23.1±2.5)	1.6	16.8 (15.9±0.7)	2.5	-
υ-DABNA	43.6 (37.4±1.6)	30.4	48.9 (38.8±3.3)	27.7	40.1 (39.2±0.9)	32.8	24.4
Control-B (Si)	35.8 (32.3±2.9)	23.1	40.2 (35.2±4.6)	18.5	34.1 (33.1±1.1)	22.4	14.6
Control-B (C)	31.8 (29.3±2.4)	17.1	35.7 (32.8±2.7)	13.3	36.7 (34.4±2.0)	19.7	3.2

ω-DABNA	145.5 (142.1±2.3)	118.6	163.2 (159.4±2.6)	117.8	40.4 (39.3±0.7)	33.4	23.9
Control-C (Si)	130.7 (124.5±5.0)	87.3	146.6 (139.7±5.6)	79.4	35.3 (34.3±0.8)	24.7	10.5
Control-C (C)	121.3 (117.1±3.2)	78.8	136.1 (131.4±3.6)	76.6	33.0 (31.7±1.0)	21.7	9.9

The average device parameters in parentheses are based on the measurement of twenty independent devices.

Comment 10: *The purity of the material should be clarified. It would also be helpful to provide the HPLC and/or HRMS data across the entire m/z range, not just for the main peak.*

Response: We appreciate the reviewer's professional advice. To clarify the purity of the materials, we have now provided the full HRMS across the entire m/z range and HPLC data for the donors and acceptors, as shown in Supplementary Figs. 5, 8, 25, 28, and 29. Based on the HPLC results, the purities of the relevant materials are in the range of 99.7%-99.9%.

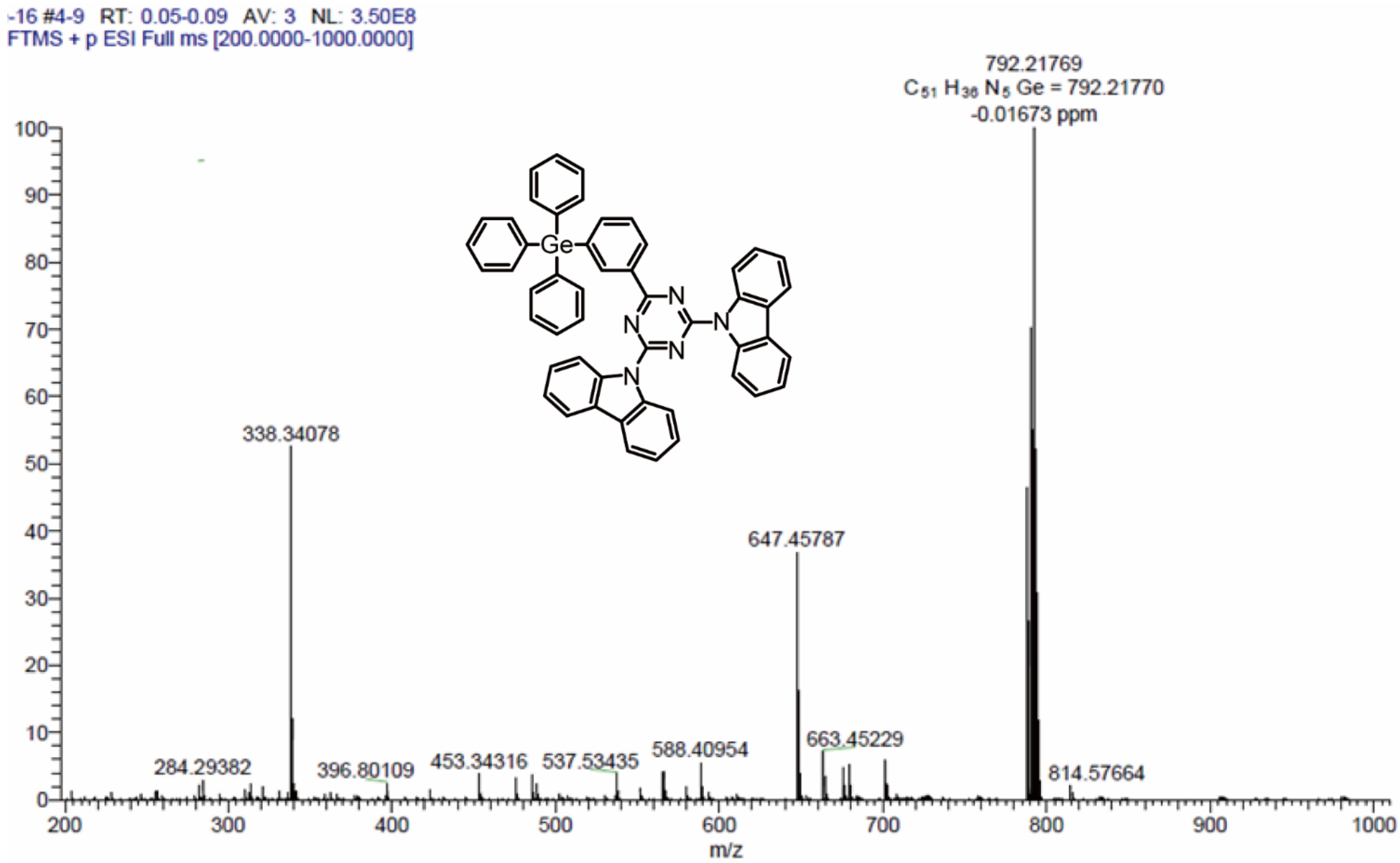

Supplementary Fig. 5. HRMS spectrum of GeTrzCz2.

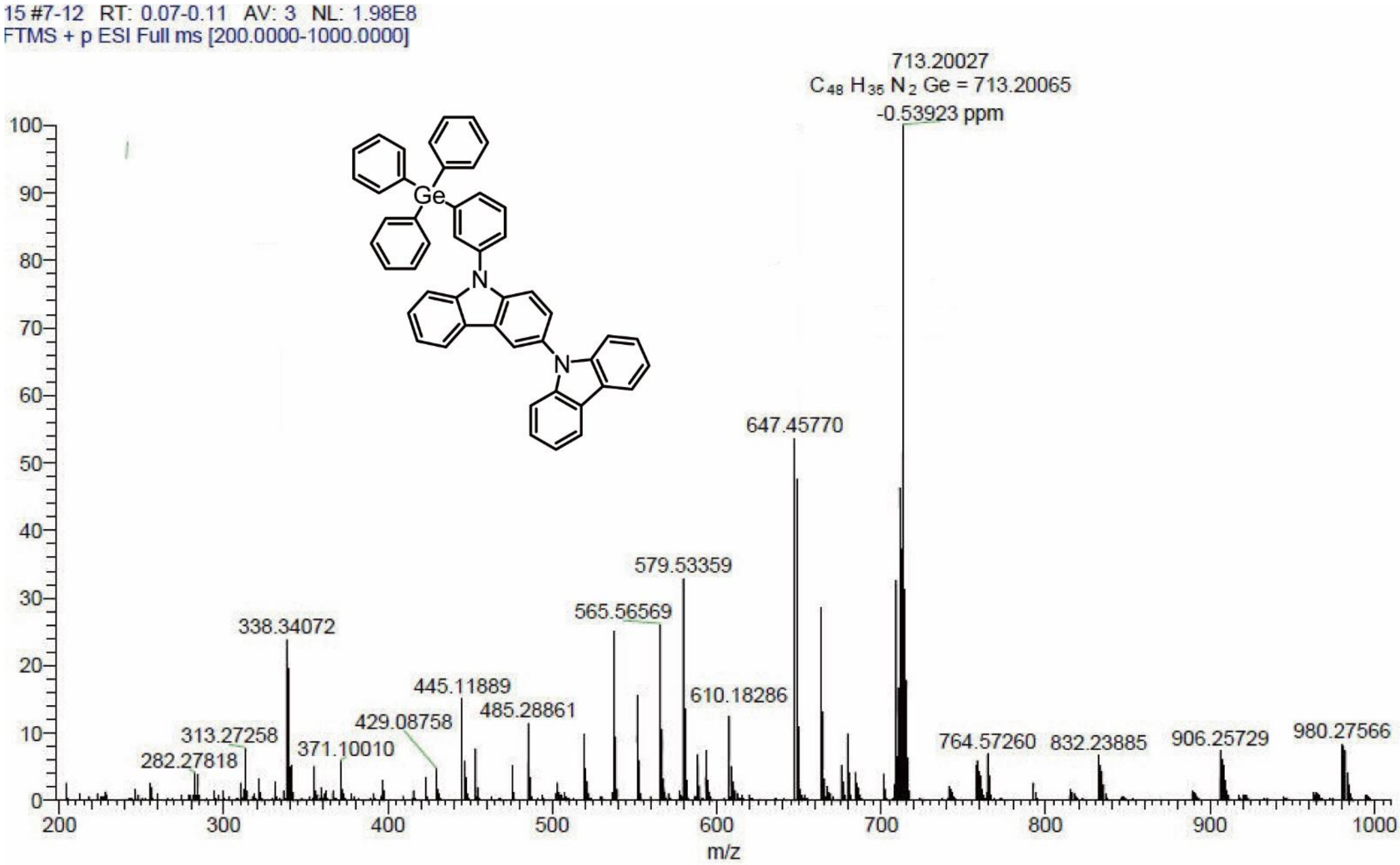

Supplementary Fig. 8. HRMS spectrum of GeCzCz.

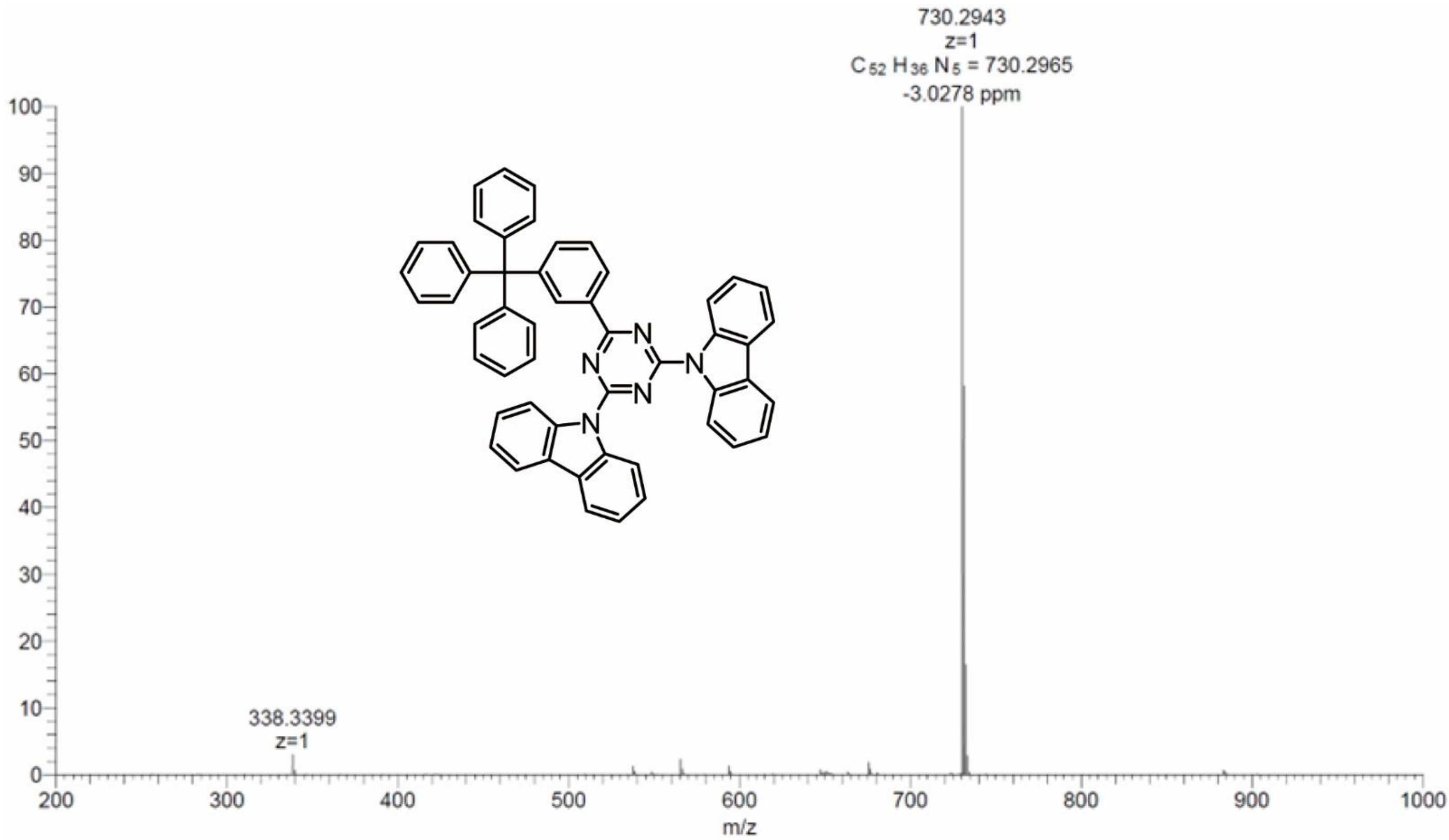

Supplementary Fig. 25. HRMS spectrum of CTrzCz2.

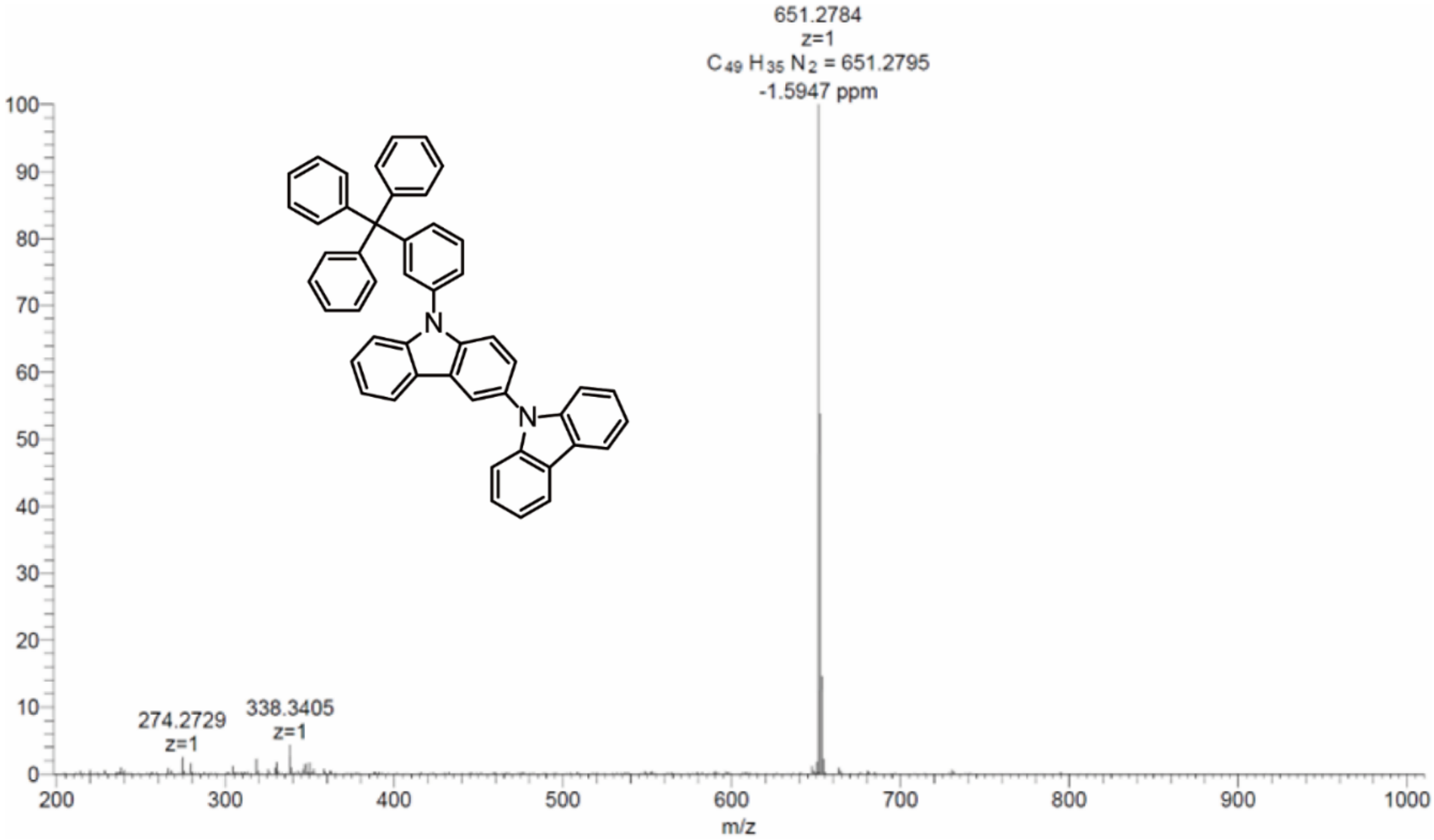

Supplementary Fig. 28. HRMS spectrum of CCzCz.

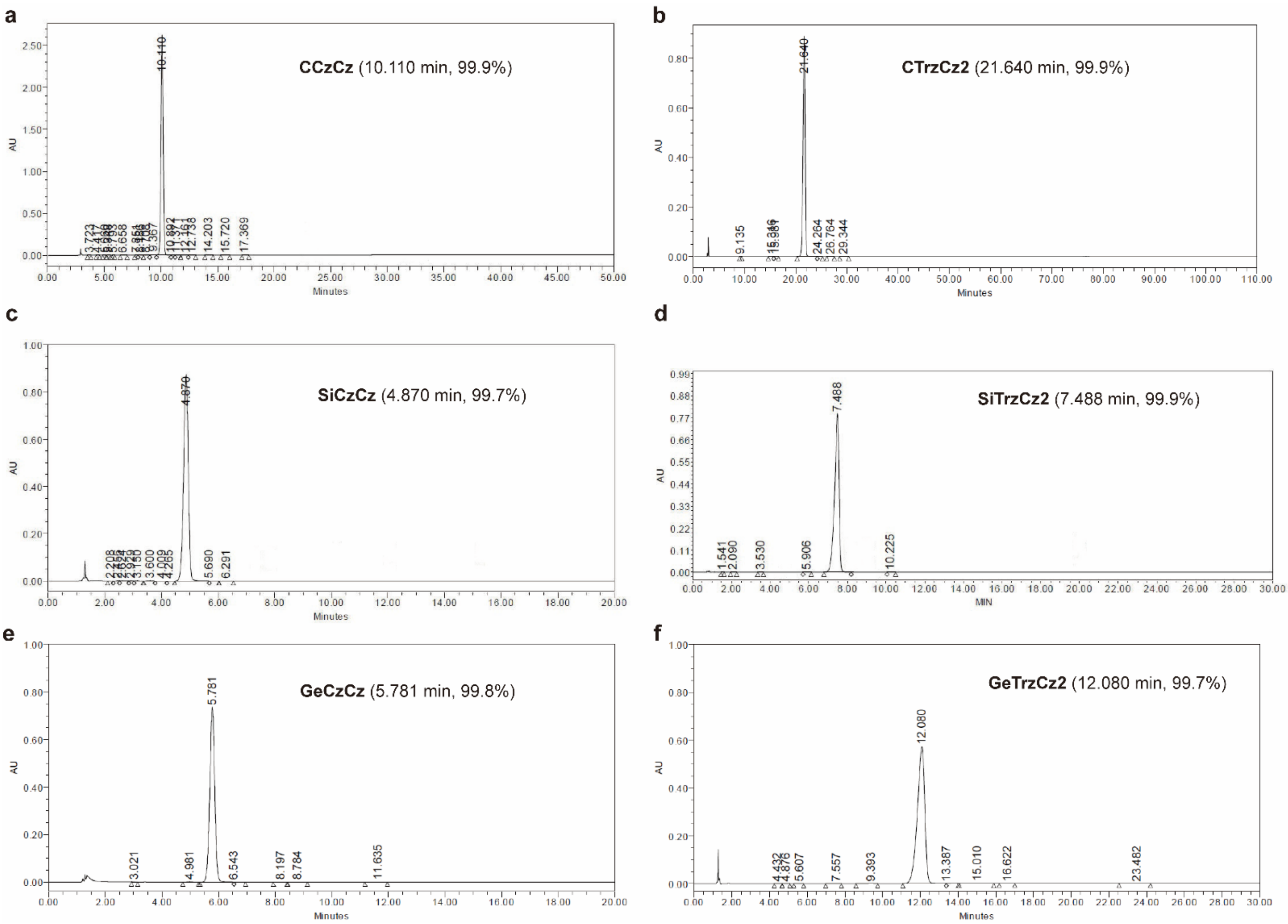

Supplementary Fig. 29. High-performance liquid chromatograms. (**a**) CCzCz. (**b**) CTrzCz2. (**c**) SiCzCz. (**d**) SiTrzCz2. (**e**) GeCzCz. (**f**) GeTrzCz2.

Comment 11: *It would be interesting to know whether the bonding dissociation energy (BDE) of both p-type and n-type host materials upon substituting Si with Ge has been calculated and compared, as this could provide evidence for whether the effect of substituting Si with Ge is solely due to the heavy atom effect or if it also contributes to device stability.*

Response: We sincerely thank the reviewer for this constructive suggestion. In response, we calculated the bond dissociation energies (BDEs) for the bonds involving the Si or Ge atom in both the p-type (GeCzCz and SiCzCz) and n-type host materials (GeTrzCz2 and SiTrzCz2) (Supplementary Table 4). The BDEs for GeCzCz and GeTrzCz2 show values essentially comparable to their silicon analogues. This similarity demonstrates that the chemical bond strengths remain basically unchanged when silicon is replaced by germanium, ruling out chemical bond stability as a contributing factor to device stability.

Supplementary Table 4. Silicon- and germanium-related bond dissociation energies. The bonds involving silicon or germanium atoms are highlighted in number for each molecular structure.

Bond	GeCzCz	SiCzCz	GeTrzCz2	SiTrzCz2
1	3.91	3.93	1.71	1.75
2	4.57	4.69	3.27	3.36
3	4.80	4.81	1.94	2.00
4	4.80	4.83	2.49	2.42

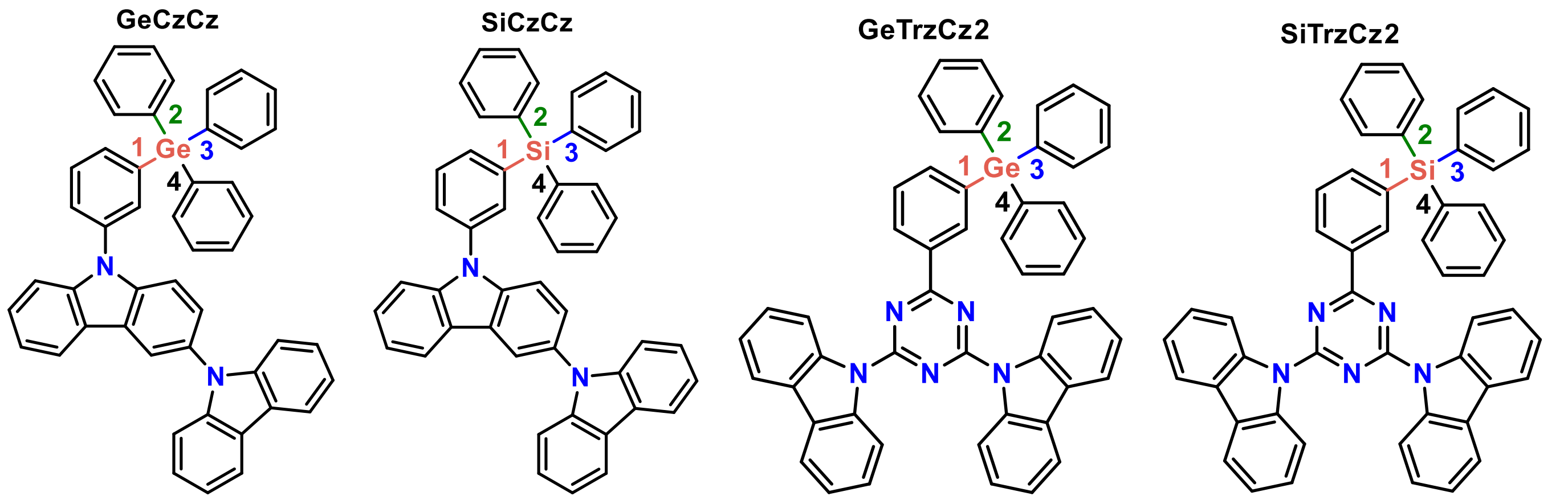

Comment 12: *The basic quantum chemical calculation results for the Ge host materials, such as oscillator strength, S_1, T_1, and reorganization energy, should be provided.*

Response: We thank the reviewer for this constructive suggestion. We performed density functional theory (DFT) and time-dependent DFT (TDDFT) calculations on the Ge-based host materials (GeCzCz and GeTrzCz2), as shown in Supplementary Fig. 13. The oscillator strengths are 0.0525 for GeCzCz and 0.0678

for GeTrzCz2. The S_1 energy levels are 3.85 eV for GeCzCz and 3.41 eV for GeTrzCz2, while the T_1 energy levels are 2.88 eV and 2.80 eV, respectively, in good agreement with experimental values. We also calculated the reorganization energies using Nelsen's four-point approach, obtaining values of 0.43 eV for GeCzCz and 0.93 eV for GeTrzCz2.

Additional sentence, (manuscript, page 6): "Theoretical calculations of GeCzCz and GeTrzCz2 (Supplementary Fig. 13) are in good agreement with experimental data, yielding singlet (S_1) energies of 3.85 eV and 3.41 eV, T_1 energies of 2.88 eV and 2.80 eV, and oscillator strengths of 0.0525 and 0.0678 for GeCzCz and GeTrzCz2, respectively. The calculated reorganization energies, which characterize the structural relaxation during electronic transitions, are 0.43 eV for GeCzCz and 0.93 eV for GeTrzCz2."

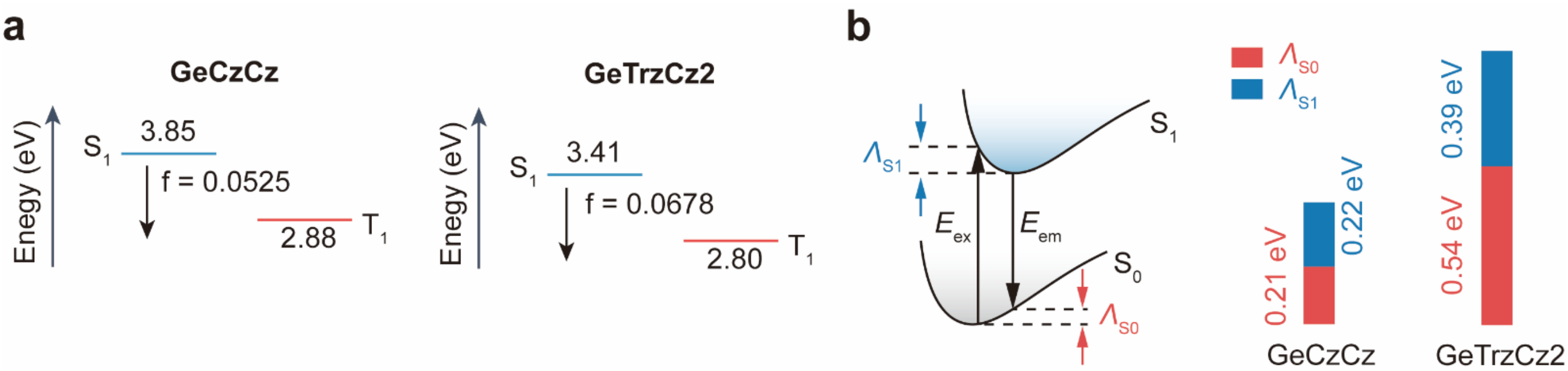

Supplementary Fig. 13. Theoretical calculations of the Ge hosts. (**a**) Transition energies and oscillator strengths. (**b**) Reorganization energy.

Comment 13: *The angular PL orientation of all films should be provided. Explanations of how it relates to the EQEs would be necessary to explain high maximum EQEs.*

Response: We appreciate the reviewer's professional suggestion. In response, we measured the angle-dependent *p*-polarized emission intensities (at the PL peak wavelength) for all emitting layer films used in the devices. For the *t*-DABNA-doped films, the horizontal dipole ratios ($\Theta_{//}$) are estimated to be 88.9%, 89.5% and 91.7% in the CCzCz:CTrzCz2, SiCzCz:SiTrzCz2 and GeCzCz:GeTrzCz2 hosts, respectively (Supplementary Fig. 32a). For the *υ*-DABNA-doped films, the estimated $\Theta_{//}$ values are 96.4%, 96.4% and 98.5% in the carbon, silicon, and germanium-based exciplex hosts, respectively (Supplementary Fig. 32b). For the *ω*-DABNA-doped films, the estimated $\Theta_{//}$ values are 97.6%, 98.4% and 99.9% in the carbon, silicon, and germanium-based exciplex hosts, respectively (Fig. 4j). These high $\Theta_{//}$ values indicate that the electric transition dipole moments of the emitters are predominantly oriented horizontally, which is known to increase the optical outcoupling of light from thin-film devices. As a result, the enhanced outcoupling contributes to

higher measured EQEs, particularly when triplet excitons are efficiently utilized.

Additional sentence, (manuscript, page 11): "To confirm the origin of the high efficiencies, the angle-dependent PL intensities were characterized to study the emitting dipole orientation of these emitting layer films. As illustrated in Fig. 4j and Supplementary Fig. 32, they showed very high horizontal emitting dipole orientation up to 99%, leading to high outcoupling efficiencies and consequently high EQEs, particularly when triplet excitons are efficiently utilized."

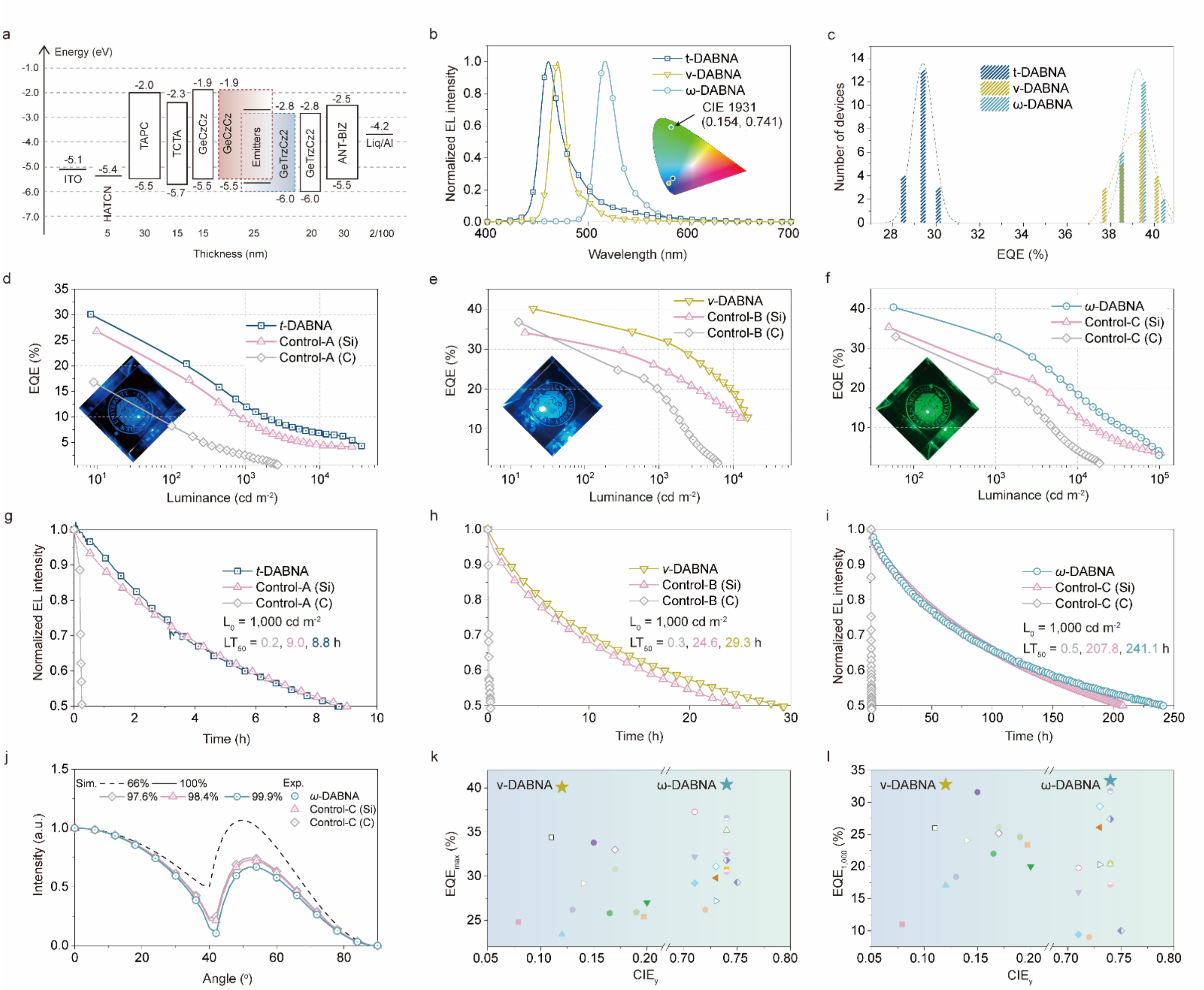

Fig. 4 | Optimized EL properties of OLEDs. a, Diagram of the germanium-based device structure. **b**, EL specrtra recorded at 1,000 cd·m^{-2}. c, Statistical histogram of EQEs for different devices. **d-f**, EQE versus luminance characteristics of target devices (*t*-DABNA (**d**), *υ*-DABNA (**e**), *ω*-DABNA (**f**)) and control devices (control-A (**d**), control-B (**e**), control-C (**f**)). **g**, Half-lifetime (LT_{50}) measurement of *t*-DABNA-based device and control devices (control-A) at an initial luminance of 1,000 cd·m^{-2}. **h**, LT_{50} measurement of *υ*-DABNA-

based device and control devices (control-B) at an initial luminance of 1,000 cd·m^{-2}. **i**, LT_{50} measurement of *ω*-DABNA-based device and control devices (control-C) at an initial luminance of 1,000 cd·m^{-2}. **j**, Angle-dependent PL spectra of *ω*-DABNA-doped films. $\Theta_{//}$, horizontal emitting dipole orientation. **k**,**l**, Summary of CIEy coordinates versus maximum EQE (**k**) values and EQE at 1,000 cd·m^{-2} (**l**) for state-of-the-art blue (CIEy ≤ 0.20) and green (CIEy ≥ 0.71) OLEDs.

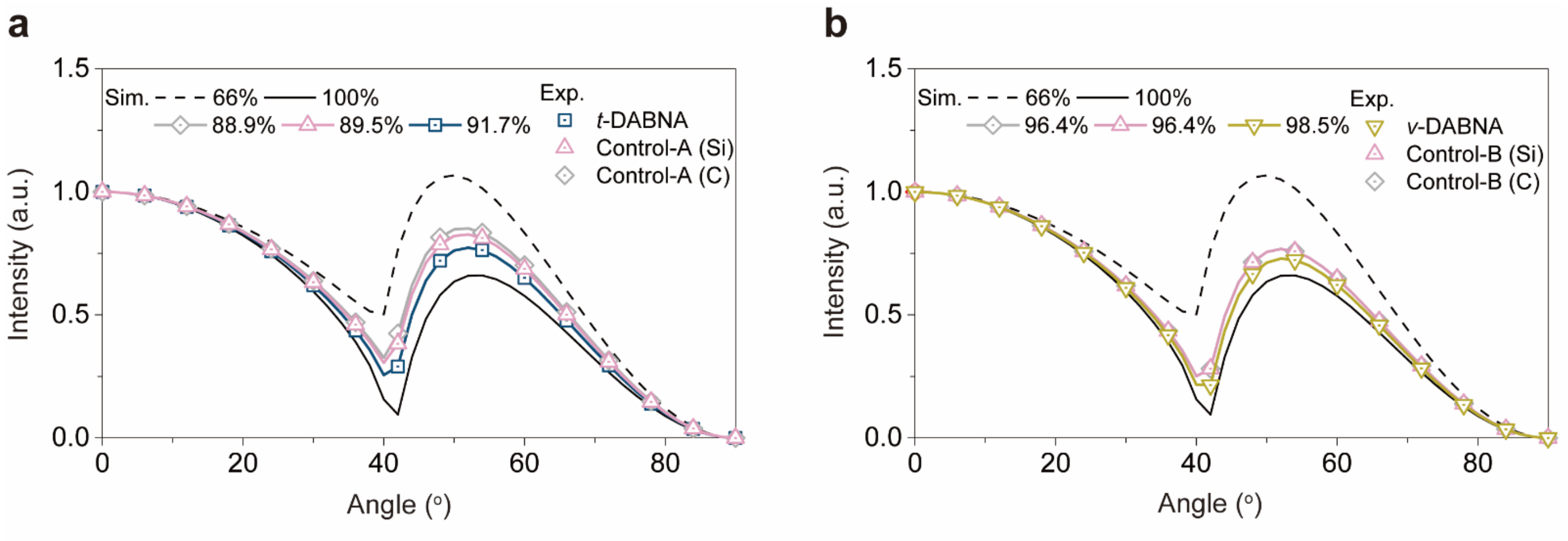

Supplementary Fig. 32. Angle-dependent PL spectra. (**a**) *t*-DABNA-doped films. (**b**) *υ*-DABNA-doped films.

Reviewer #3:

General comment: *The authors report a strategy to improve device efficiency roll-off in OLEDs using an exciplex host formed between hole (GeCzCz) and electron (GeTrzCz2) transport moieties containing germanium. The exciplex formation between GeCzCz and GeTrzGCz2 is demonstrated by the red-shifted emission with longer lifetime observed in the blended film, compared to neat films of GeCzCz and GeTrzCz2. The heavy-atom effect caused by the presence of Ge (Z=32) accelerates the spin-flip dynamics and enhances RISC rates in exciplex host, compared with the silicon SiCCz:SiTrzCz2 exciplex. Spin-orbit coupling matrix elements (SOCMEs) calculated for (SiCzCz:SiTrzCz2) and (GeCzCz:GeTrzCz2) show an increase from 0.265 cm^{-1} to 0.791 cm^{-1}, consistent with the expected enhancement in spin-orbit coupling.*

TADF Blue and Green OLEDs were fabricated with the germanium exciplex host and its silicon counterpart host. The OLEDs with the Ge exciplex host show impressive performances that consistently outperform the performances of devices with the silicon host, which demonstrates the strategy is effective on enhancing spin-flip dynamics and effectively harvesting triplet states.

I think the work is very interesting and well done. We could ask for further confirmation of TADF in the host exciplex which has not been fully tested, but the results show that TADF is most likely reason for the observation of delayed fluorescence. Thus, I don't think it is necessary to request further confirmation. The manuscript is written in a concise manner and well organized. Therefore, I have no further questions, and recommend the manuscript to be accepted for publication in the current form.

Response: We sincerely appreciate the reviewer's positive comments. To unequivocally confirm that the delayed component of the host exciplex originates from TADF, we have carried out temperature-dependent transient PL studies from 100 K to 300 K for the SiCzCz:SiTrzCz2 and GeCzCz:GeTrzCz2 films. These results are now presented in Supplementary Fig. 14. The transient PL data reveal a noticeable increase in the delayed fluorescence components with rising temperature, which is characteristic of an endothermic RISC process and confirms the TADF behavior in the host exciplex.

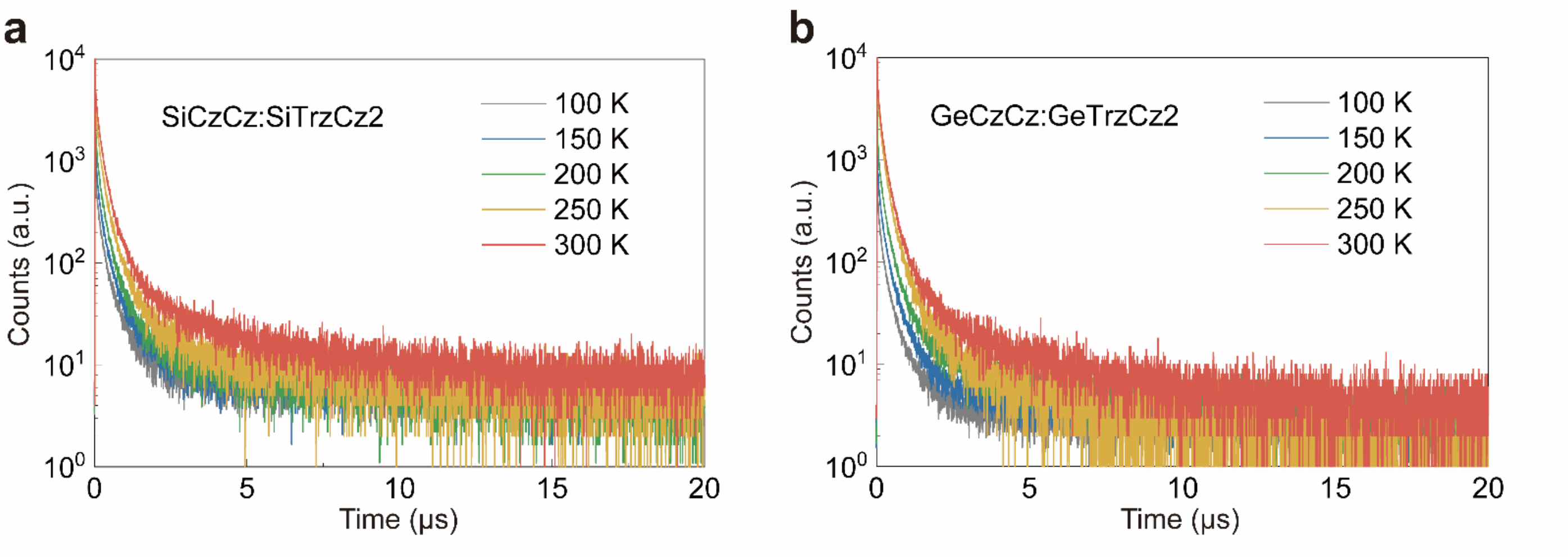

Supplementary Fig. 14. Temperature-dependent transient photoluminescence decay spectra of SiCzCz:SiTrzCz2 and GeCzCz:GeTrzCz2 films.

Dear Reviewers,

Enclosed please find our revised manuscript entitled "**Germanium-integrated exciplex host for high-performance narrowband OLEDs with mitigated efficiency roll-off**" (Manuscript ID: **NCOMMS-25-25950B**) for publication as a research article in ***Nature Communications***. We sincerely thank the reviewers for their insightful and professional comments, which helped us substantially improve the quality of our manuscript.

We have meticulously revised the manuscript in response to Reviewer #2's comments and the point-by-point responses are provided. The major improvements upon revision include:

1. The hyperconjugation effect is quantified and the robustness of the spin-orbit coupling enhancement is validated through systematic conformational studies.
2. Theoretical calculations and vibrational coupling analyses are performed for the entire C → Si → Ge series.
3. The horizontal dipole orientations for the carbon, silicon and germanium-based systems are re-evaluated with statistical reproducibility and corresponding optical simulations are conducted.
4. Single-carrier devices are supplemented for comprehensive analysis of charge-transport properties.

Additionally, we have added Ms. Siyuan He from Shenzhen University as a co-author for her contribution to the fabrication of hole-only and electron-only devices. We have also added Dr. Cheng Zhong from Wuhan University for his contribution to the theoretical calculations, and Ms. Xue Zhou and Dr. Yu Gu from Wuhan China Star Optoelectronics Semiconductor Display Technology Co., Ltd. for their contributions to the optical simulations of the devices. Furthermore, Dr. Zhanxiang Chen (Shenzhen University) has been designated as a co-corresponding author. All authors have formally approved these changes and the new author list, and the completed Author List Change Form is enclosed with this submission.

For your reference, annotated files of the revised manuscript and supporting information with all changes highlighted in yellow are also attached. We believe these revisions have greatly strengthened the manuscript, and we hope it now meets the high standards required for publication in *Nature Communications*.

We sincerely thank the reviewers for your time, constructive feedback, and consideration of our work.

Yours Sincerely,
Chuluo Yang on behalf of the authors.
Corresponding author: **Professor Chuluo Yang**

Point-by-Point Response to Reviewers' Comments

Reviewer #1:

General comment: *It is acceptable as is.*

Response: We sincerely appreciate the reviewer for the positive comment.

Reviewer #2:

General comment: *The current version has been significantly improved through the authors' revisions. However, it still contains ambiguities and contradictions in fundamental aspects.*

Response: We sincerely appreciate the reviewer's critical and professional comments.

Comment 1: *Major points: The reported enhancement in SOCME upon substitution of Si with Ge constitutes a central mechanistic argument of the manuscript. Basically, to improve transparency and reproducibility, the authors are also encouraged to describe the computational protocol used for SOC analysis in sufficient detail (e.g., functional and basis set, relativistic treatment, SOC evaluation approach, and the method used for atomic/fragment decomposition). While the authors attribute this enhancement partly to heavy-atom effects and hyperconjugative interactions at the sp^3-bridged position, the theoretical basis for invoking hyperconjugation remains insufficiently clarified. In Supplementary Fig. 17, SOCMEs and atomic/fragment contributions are presented for the Si- and Ge-based systems. However, it is not clearly established how hyperconjugation is defined and quantified in this context. It would be helpful if the authors could clarify whether direct computational evidence (e.g., NBO analysis, orbital interaction analysis, or second-order perturbation energies) demonstrates σ-π mixing involving the sp^3-bridged atom, and how such mixing mechanistically translates into increased SOC matrix elements. Furthermore, since the manuscript aims to establish a systematic C → Si → Ge trend, it would be important to include SOC calculations for the carbon-based exciplex as well. At present, the discussion focuses on the Si and Ge systems, which makes it difficult to assess whether the SOC enhancement follows a consistent progression across the entire series.*

Response: We thank the reviewer for pointing these crucial points. To ensure that the calculated S_1 energies were in good agreement with experimental values, spin-orbit coupling (SOC) matrix elements were computed using the PySOC package interfaced with Gaussian 16 at the B3LYP functional level. The 6-31G(d) basis set was employed for all hydrogen, carbon, nitrogen, and silicon atoms. To properly account for relativistic effects, the heavy germanium atom was treated using the Stuttgart-Dresden (SDD) pseudopotential and its associated

valence basis set. The SOC matrix elements were evaluated from time-dependent density functional theory wavefunctions, employing the Breit-Pauli spin-orbit Hamiltonian and an effective nuclear charge (Z_{eff}) approximation via the MolSOC module. To isolate and quantify the SOC contribution of the specific target atom, we implemented a Z_{eff} masking approach. By setting the Z_{eff} of all other atoms in the input to zero (Z_{eff} = 0), the resulting SOCME reflects only the localized contribution originating from the selected target atom.

Regarding hyperconjugation, we have performed natural bond orbital (NBO) analysis to confirm the presence of σ-π mixing. The second-order perturbation energy (E(2)) for the $\sigma \rightarrow \pi^*$ coupling in the GeCzCz:GeTrzCz2 (1.25 kcal·mol^{-1}) is found to be higher than that in the SiCzCz:SiTrzCz2 (0.73 kcal·mol^{-1}). To quantify how σ-π mixing mechanistically enhances the SOC matrix elements, we have performed fuzzy atomic space analysis on the natural transition orbitals (NTOs) of the S_1 state. This analysis shows that the lowest unoccupied NTO exhibits spatial distributions of 0.26% on the Ge atoms in GeCzCz:GeTrzCz2, 0.28% on the Si atoms in SiCzCz:SiTrzCz2, and 0.12% on the sp^3-bridged C atoms in CCzCz:CTrzCz2. Although these percentage values appear modest, this σ-π mixing enables the sp^3-bridged atoms to participate directly in the triplet-to-singlet electronic transitions. Given that SOC scales with the fourth power of the effective nuclear charge ($SOC \propto Z_{\mathrm{eff}}^4$), the participation of the heavy Ge atom (Z_{eff} = 6.78) introduces a heavy atom effect that is significantly more potent than that of Si (Z_{eff} = 4.29) or C (Z_{eff} = 3.14). This synergy between σ-π mixing and the large Z_{eff} of Ge ultimately drives the increased SOC matrix elements in GeCzCz:GeTrzCz2.

Finally, as requested, we have calculated the SOC matrix elements for the carbon-based exciplex. As detailed in Supplementary Fig. 18 and Supplementary Tables 7-9, the SOC matrix elements ($\langle S_1|\hat{H}_{SOC}|T_1\rangle$) for the lowest-energy conformers exhibit **a clear monotonic increase**, progressing from 0.169 cm^{-1} for the carbon-based system (CCzCz:CTrzCz2) to 0.265 cm^{-1} for the silicon-based system (SiCzCz:SiTrzCz2), and further to 0.791 cm^{-1} for the germanium-based system (GeCzCz:GeTrzCz2). This consistent progression is also observed across all other sampled conformers, confirming that the SOC enhancement follows a systematic trend across the entire C → Si → Ge series.

Modified sentences (manuscript, page 7):

"Analysis of spin-orbit coupling matrix elements (SOCMEs) in the optimized T_1 geometry of the lowest-energy conformer showed an approximately threefold increase in $\langle S_1|\hat{H}_{SOC}|T_1\rangle$ from 0.265 cm^{-1} (SiCzCz:SiTrzCz2) to 0.791 cm^{-1} (GeCzCz:GeTrzCz2), consistent with the expected enhancement in spin-orbit coupling due to the higher atomic number (Supplementary Fig. 17). Similar trends are observed for other conformers (Supplementary Fig. 18) and for the upper excited state (T_2)…."

Additional sentences (manuscript, page 14):

"Subsequently, spin-orbit coupling (SOC) matrix elements were computed using the PySOC package interfaced with Gaussian 16 at the B3LYP functional level[40]. These matrix elements were evaluated from the TD-DFT wavefunctions using the Breit-Pauli spin-orbit Hamiltonian in conjunction with the effective nuclear charge (Z_{eff}) approximation via the MolSOC module. To isolate and quantify the SOC contribution of the specific target atom, we implemented a Z_{eff} masking approach. By setting the Z_{eff} of all other atoms in the input to zero ($Z_{eff} = 0$), the resulting SOCME reflects only the localized contribution originating from the selected target atom."

Additional sentence (Supplementary Information, Page S31): "Regarding hyperconjugation, natural bond orbital (NBO) analysis shows that the second-order perturbation energy ($E(2)$) for the $\sigma \rightarrow \pi^*$ coupling in the GeCzCz:GeTrzCz2 (1.25 kcal·mol^{-1}) is higher than that in the SiCzCz:SiTrzCz2 (0.73 kcal·mol^{-1})."

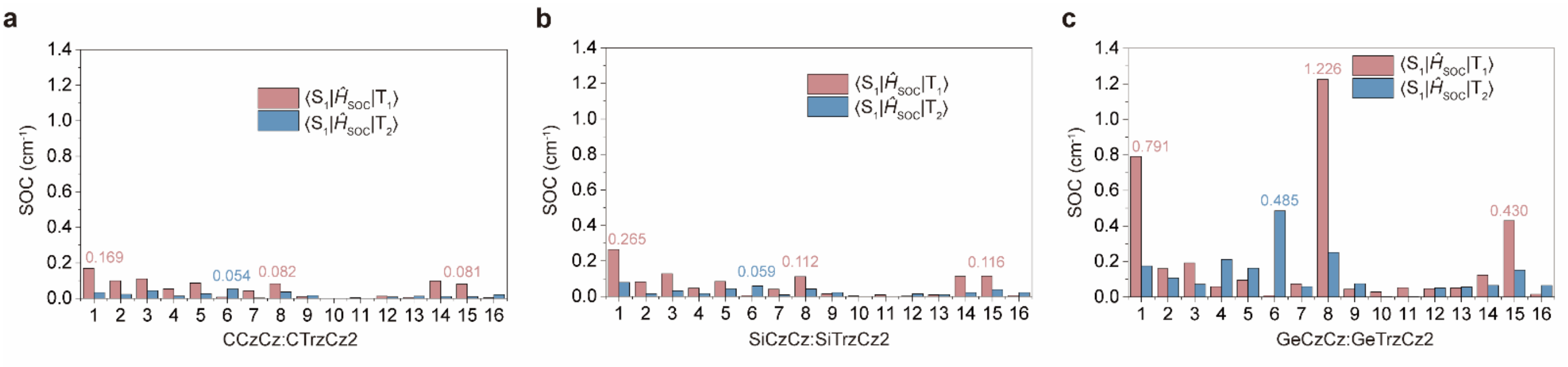

Supplementary Fig. 18. Calculated spin-orbit coupling (SOC) constants of donor-acceptor molecular pairs. (**a**) CCzCz:CTrzCz2. (**b**) SiCzCz:SiTrzCz2. (**c**) GeCzCz:GeTrzCz2.

Comment 2: *Spin-orbit coupling matrix elements can be sensitive to molecular geometry, particularly in exciplex systems involving through-space donor–acceptor interactions and conformational flexibility. It would therefore be helpful if the authors could clarify how the molecular geometry used for SOC calculations was selected. Specifically: 1) Was a conformational search performed prior to geometry optimization? 2) Were multiple low-energy conformers examined? 3) How sensitive are the SOCME values to conformational variation? If the reported SOC enhancement remains consistent across energetically accessible conformations, this would strongly reinforce the robustness of the conclusion. Finally, given the central importance of the computational results to the manuscript's mechanistic claims, I kindly request that the Cartesian coordinates of all optimized structures used for SOC calculations (carbon-, silicon-, and germanium-based systems) be provided in the Supporting Information to enhance reproducibility and transparency.*

Response: We fully agree with this suggestion and thank the reviewer for bringing it to our attention.

1) Although a conformational search was not performed before geometry optimization in our initial calculations, we recognize that this step is indeed necessary for exciplex systems with conformational flexibility. Accordingly, we have performed a conformational search for the exciplex systems based on the optimized T_1 state using the Molclus program in combination with xtb and Gaussian, as detailed in the updated Methods section. By applying a spatial coordinate threshold of 3.0 Å, this search yielded 16 unique conformers of each exciplex system, which are presented in Supplementary Fig. 18 and Supplementary Tables 7-9.

2) We have examined multiple low-energy conformers. To evaluate their thermodynamic relevance at room temperature (T = 298.15 K), we have calculated their Boltzmann population distribution based on their relative single-point energies (Supplementary Tables 7-9). The Boltzmann analysis reveals that the population is overwhelmingly dominated by a single, lowest-energy conformer, which accounts for 99.82%, 99.99%, and 99.96% of the total population for CCzCz:CTrzCz2, SiCzCz:SiTrzCz2, and GeCzCz:GeTrzCz2, respectively. While the contributions of the remaining 15 higher-energy conformers are thermodynamically negligible at room temperature, we have nevertheless calculated the spin-orbit coupling matrix elements (SOCMEs) for all 16 conformers to ensure scientific rigor and rule out any potential conformational bias.

3) To quantitatively assess the sensitivity of the SOCMEs to conformational variations, we have systematically analyzed these 16 optimized conformers for the carbon-, silicon-, and germanium-based systems. We used the root-mean-square deviation (RMSD) of the remaining 15 conformers relative to the lowest-energy one to represent the degree of structural variation. These conformers exhibit substantial structural variation, with RMSD values ranging from 4.830 Å to 8.871 Å. We also calculated the statistical mean, standard deviation (S.D.) and relative standard deviation (RSD = S.D./Mean × 100%) for the SOCMEs (Supplementary Tables 7-9). Our statistical analysis shows that **the SOCMEs are indeed highly sensitive to conformational variation**; specifically, the RSD values for the key $\langle S_1|\hat{H}_{SOC}|T_1\rangle$ are 92.08%, 105.93%, and 153.30% for the carbon-, silicon-, and germanium-based systems, respectively.

Crucially, despite the high sensitivity of individual conformations, **the SOC enhancement remains consistent across energetically accessible conformations**. By calculating the ensemble-averaged SOCME across all 16 conformers for each system, we confirmed that the germanium-based system exhibits a consistent and substantial enhancement over its lighter congeners. Specifically, for the $\langle S_1|\hat{H}_{SOC}|T_1\rangle$, the ensemble-averaged SOCME of the germanium-based system (0.212 cm^{-1}) is significantly higher than those of the silicon-based (0.065 cm^{-1}) and carbon-based (0.054 cm^{-1}) systems. An even more pronounced enhancement is observed for the $\langle S_1|\hat{H}_{SOC}|T_2\rangle$, where the average SOC of the germanium-based system (0.125 cm^{-1})

outperforms the silicon (0.027 cm^{-1}) and carbon (0.020 cm^{-1}) systems by several folds.

Finally, to ensure the transparency and reproducibility of our results, the Cartesian coordinates for all optimized structures of the carbon, silicon, and germanium-based exciplex systems have been provided in the Supporting Information (Supplementary Tables 10-57).

Taken together, these results confirm that germanium effectively increases the SOCME values of the exciplex system.

Modified section (Methods, Page 13):

"**Theoretical calculations**

All DFT calculations were carried out using the Gaussian 16 software package[34]. Geometry optimizations were performed using the B3LYP functional with the DFT-D3(BJ) empirical dispersion correction[35]. The 6-31G(d) basis set was employed for hydrogen, carbon, nitrogen, and silicon atoms[36], while the Stuttgart-Dresden (SDD) effective core potential was applied for germanium (Ge)[37]. Time-dependent DFT calculations for excited states were conducted at the CAM-B3LYP functional level with the same basis sets[38]. To obtain the optimal bimolecular conformations of the donor-acceptor pairs in the exciplex systems, a systematic, multi-step conformational search was implemented. Initially, 5,000 donor-acceptor configurations were generated using the Genmer package and pre-optimized at the semiempirical GFN2-xTB levels using the Molclus program[39]. These candidate structures were further optimized at the B3LYP-D3(BJ)/6-31G(d)/SDD(Ge) level in Gaussian 16. To eliminate structural redundancy, near-duplicate conformations were filtered out by applying a spatial coordinate threshold of 3.0 Å, yielding 16 distinct bimolecular candidates. The geometries of the lowest triplet states (T_1) were subsequently optimized utilizing the Tamm-Dancoff approximation (TDA) to TD-DFT at the CAM-B3LYP-D3(BJ)/6-31G(d)/SDD(Ge) level. Subsequently, spin-orbit coupling (SOC) matrix elements were computed using the PySOC package interfaced with Gaussian 16 at the B3LYP functional level[40]. These matrix elements were evaluated from the TD-DFT wavefunctions using the Breit-Pauli spin-orbit Hamiltonian in conjunction with the effective nuclear charge (Z_{eff}) approximation via the MolSOC module. To isolate and quantify the SOC contribution of the specific target atom, we implemented a Z_{eff} masking approach. By setting the Z_{eff} of all other atoms in the input to zero ($Z_{eff} = 0$), the resulting SOCME reflects only the localized contribution originating from the selected target atom."

Additional reference:

"39. Lu, T. Molclus program, Version 1.12. http://www.keinsci.com/research/molclus.html."

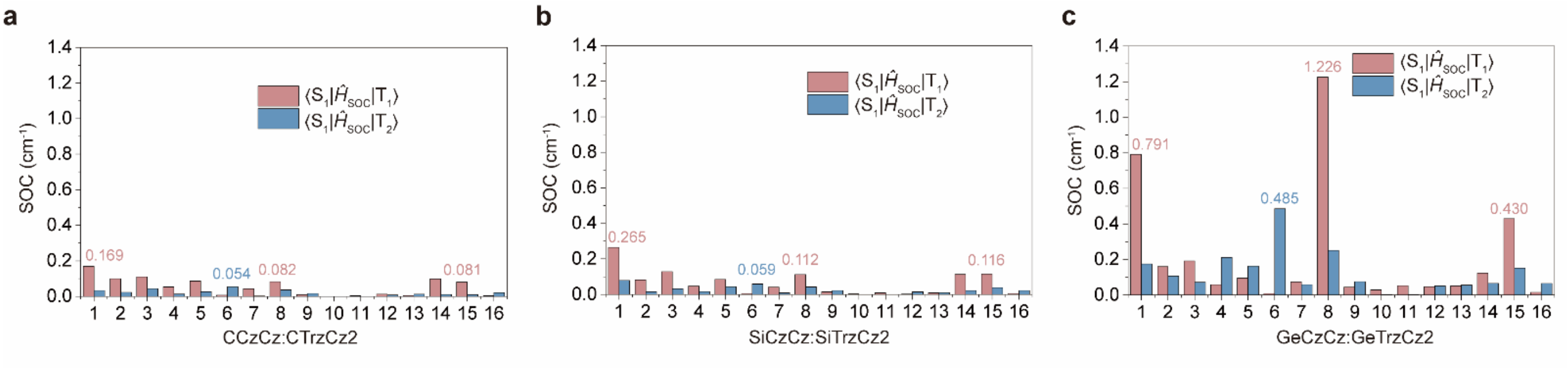

Supplementary Fig. 18. Calculated spin-orbit coupling (SOC) constants of donor-acceptor molecular pairs. (**a**) CCzCz:CTrzCz2. (**b**) SiCzCz:SiTrzCz2. (**c**) GeCzCz:GeTrzCz2.

Supplementary Table 7. Computational results for the CCzCz:CTrzCz2 exciplex. Calculated spin-orbit coupling matrix elements between S_1 and T_1, and between S_1 and T_2, for 16 representative conformers. The relative energy (ΔE) and the corresponding Boltzmann factor ($e^{-\Delta E/RT}$), alongside the structural deviation relative to the lowest-energy conformer expressed as root-mean-square deviations (RMSD), are also listed. The mean values, standard deviation (S.D.), and relative standard deviation (RSD) are provided for statistical analysis.

**CCzCz:CTrzCz2**	**$\langle S_1\|\hat{H}_{SOC}\|T_1\rangle$ / cm^{-1}**	**$\langle S_1\|\hat{H}_{SOC}\|T_2\rangle$ / cm^{-1}**	**ΔE / kcal·mol^{-1}**	**$e^{-\Delta E/RT}$**	***RMSD* / Å**
1	0.16874	0.03390	0.00000	1	0.000
2	0.09916	0.02220	4.00269	0.001158994	4.910
3	0.11068	0.04395	4.49955	0.000500771	5.966
4	0.05297	0.01277	9.22839	1.70254×10^{-7}	4.844
5	0.08648	0.02692	10.20627	3.26464×10^{-8}	5.959
6	0.00642	0.05411	9.28205	1.55503×10^{-7}	6.144
7	0.04313	0.00174	9.57875	9.42137×10^{-8}	6.710
8	0.08245	0.03540	14.61066	1.9199×10^{-11}	6.825
9	0.01040	0.01721	21.93126	8.19815×10^{-17}	6.358
10	0.00067	0.00061	18.00162	6.25244×10^{-14}	7.220
11	0.00362	0.00117	7.93451	1.51405×10^{-6}	5.590
12	0.01197	0.00795	33.85830	1.46347×10^{-25}	6.327
13	0.00493	0.01219	25.67383	1.47431×10^{-19}	6.088
14	0.09815	0.01090	12.21624	1.09535×10^{-9}	5.480
15	0.08060	0.01023	5.55057	8.48651×10^{-5}	5.069
16	0.00234	0.02116	6.95982	7.85352×10^{-6}	7.004
**Mean**	0.05392	0.01953	-	-	-

**S.D.**	0.04965	0.01526	-	-	-
**RSD**	92.08%	78.14%	-	-	-

Supplementary Table 8. Computational results for the SiCzCz:SiTrzCz2 exciplex. Calculated spin-orbit coupling matrix elements between S_1 and T_1, and between S_1 and T_2, for 16 representative conformers. The relative energy (ΔE) and the corresponding Boltzmann factor ($e^{-\Delta E/RT}$), alongside the structural deviation relative to the lowest-energy conformer expressed as root-mean-square deviations (RMSD), are also listed. The mean values, standard deviation (S.D.), and relative standard deviation (RSD) are provided for statistical analysis.

**SiCzCz:SiTrzCz2**	**$\langle S_1\|\hat{H}_{SOC}\|T_1\rangle$ / cm^{-1}**	**$\langle S_1\|\hat{H}_{SOC}\|T_2\rangle$ / cm^{-1}**	**ΔE / kcal·mol^{-1}**	**$e^{-\Delta E/RT}$**	***RMSD* / Å**
1	0.26543	0.08124	0.00000	1	0
2	0.08236	0.01613	5.92511	4.50829×10^{-5}	4.830
3	0.12803	0.03323	6.92196	8.37206×10^{-6}	6.112
4	0.04685	0.01711	12.47811	7.0384×10^{-10}	4.881
5	0.08415	0.04372	14.09930	4.55358×10^{-11}	6.561
6	0.00518	0.05929	12.74074	4.51687×10^{-10}	6.780
7	0.04150	0.00879	10.53546	1.87231×10^{-8}	6.871
8	0.11208	0.04238	16.95826	3.642×10^{-13}	6.807
9	0.01515	0.02341	23.52659	5.54061×10^{-18}	6.885
10	0.00392	0.00032	21.82230	9.85455×10^{-17}	7.752
11	0.00950	0.00061	13.63273	1.00133×10^{-10}	6.333
12	0.00298	0.01499	37.06810	6.47209×10^{-28}	6.704
13	0.00940	0.01114	34.23180	7.78803×10^{-26}	6.747
14	0.11374	0.02210	14.75718	1.49902×10^{-11}	5.592
15	0.11571	0.03940	8.34455	7.57507×10^{-7}	5.182
16	0.00563	0.02327	6.58050	1.49035×10^{-5}	7.518
**Mean**	0.06507	0.02724	-	-	-
**S.D.**	0.06893	0.02086	-	-	-
**RSD**	105.93%	76.58%	-	-	-

Supplementary Table 9. Computational results for the GeCzCz:GeTrzCz2 exciplex. Calculated spin-orbit coupling matrix elements between S_1 and T_1, and between S_1 and T_2, for 16 representative conformers. The corresponding Boltzmann factor ($e^{-\Delta E/RT}$) and the structural deviation relative to the lowest-energy conformer, expressed as root-mean-square deviations (RMSD), are also listed. The mean values, standard deviation (S.D.), and relative standard deviation (RSD) are provided for statistical analysis.

GeCzCz:GeTrzCz2	$\langle S_1\|\hat{H}_{SOC}\|T_1\rangle$ / cm^{-1}	$\langle S_1\|\hat{H}_{SOC}\|T_2\rangle$ / cm^{-1}	ΔE / kcal·mol^{-1}	$e^{-\Delta E/RT}$	*RMSD* / Å
1	0.79143	0.17433	0.00000	1	0.000
2	0.16029	0.10675	5.17048	0.000161259	5.524
3	0.19178	0.07142	6.19551	2.85544×10^{-5}	6.132
4	0.05882	0.21231	11.36845	4.58556×10^{-9}	5.926
5	0.09562	0.16273	13.75949	8.08348×10^{-11}	5.355
6	0.00980	0.48516	11.57260	3.24827×10^{-9}	6.276
7	0.07429	0.05909	9.46046	1.15048×10^{-7}	5.515
8	1.22599	0.25092	16.26596	1.17256×10^{-12}	6.873
9	0.04438	0.07668	21.88645	8.84258×10^{-17}	5.773
10	0.02876	0.00193	20.54772	8.4824×10^{-16}	7.616
11	0.05288	0.00342	12.46821	7.15702×10^{-10}	6.653
12	0.04708	0.05181	36.48192	1.74181×10^{-27}	8.140
13	0.05117	0.05680	33.67265	2.00244×10^{-25}	8.871
14	0.12324	0.06548	14.30816	3.20005×10^{-11}	6.705
15	0.42973	0.15214	7.26573	4.68476×10^{-6}	6.475
16	0.01486	0.06466	5.03800	0.000201695	6.048
**Mean**	0.21248	0.12471	-	-	-
**S.D.**	0.32573	0.11585	-	-	-
**RSD**	153.30%	92.90%	-	-	-

Comment 3: *The quantitative estimation of k_{DET} provides useful insight into exciton dynamics. However, based on Supplementary Table 2, the absolute k_{DET} values remain on the order of 10^6 s^{-1}, whereas k_{RISC} lies in the 10^4–10^5 s^{-1} regime. This suggests that Dexter energy transfer is kinetically faster than RISC in several cases. In addition, Figure 1a schematically presents the proposed energy-transfer pathway. Given that DET appears to be kinetically competitive—or even faster—than RISC in several systems, it would be helpful if the authors could clarify whether the schematic represents the dominant kinetic pathway or serves as a conceptual illustration. A clearer reconciliation between the measured rate constants and the pathway illustrated in*

Figure 1a would improve internal consistency.

Response: We appreciate the reviewer's careful reading and insightful feedback. We respectfully clarify that Figure 1a serves primarily as a conceptual illustration showing the co-existing energy-transfer and spin-flip processes, rather than a rate-determining kinetic flowchart. We completely agree that the fast k_{DET} leads to a rapid transfer of a significant portion of triplet excitons from exciplex host to the MR-TADF guest molecules. However, this does not represent a loss pathway for luminescence. The external heavy-atom effect originating from the germanium-based exciplex host plays a crucial role here. Through the external heavy-atom effect, these accumulated guest triplet excitons undergo an enhanced reverse intersystem crossing (RISC) into singlet excitons, which subsequently decay radiatively to emit light (*Angew. Chem. Int. Ed.* **64**, e202502380 (2025)). This RISC enhancement is quantitatively supported by our data, as the k_{RISC} for *t*-DABNA, *υ-DABNA* and *ω*-DABNA increased by 1.69-fold, 2.02-fold and 2.08-fold in the germanium-based exciplex host. In other words, **while the fast k_{DET} rapidly funnels triplet excitons to the MR-TADF guest, the external heavy-atom effect of the host ensures that these triplets arriving at guest are efficiently utilized rather than wasted**.

To improve internal consistency and clearly reconcile the measured rate constants with the proposed mechanism, we have updated Fig. 1a to explicitly include both the DET pathway and the subsequent spin-flip (RISC) process of the guest triplets.

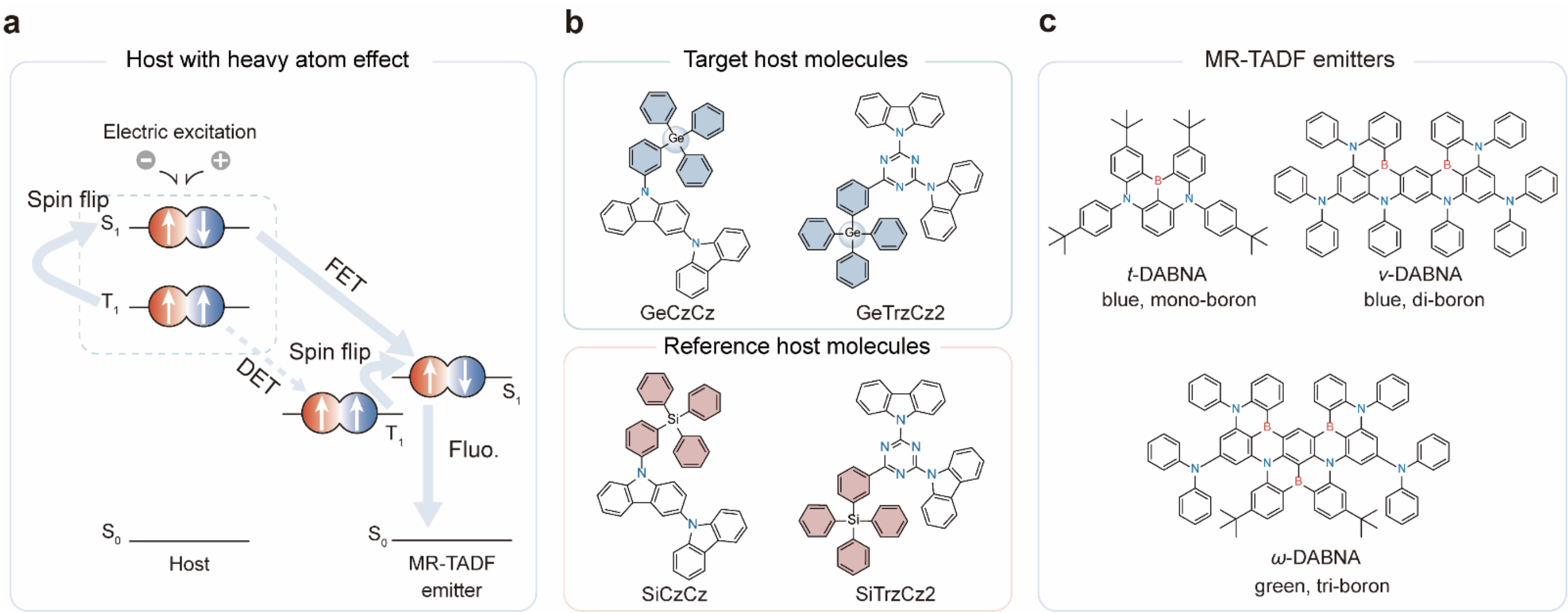

Fig. 1 | Host-guest system design. **a**, Conceptual illustration of reverse intersystem crossing (RISC), Förster-type energy transfer (FET) and Dexter-type energy transfer (DET) processes in EL devices. **b**, Chemical structures of the target germanium-based hosts (GeCzCz, GeTrzCz2) and reference silicon-based hosts (SiCzCz, SiTrzCz2). **c**, Chemical structures of the MR-TADF emitters used in this study.

Comment 4: *The systematic increase in horizontal dipole ratio from the carbon- to silicon- to germanium-based hosts is an interesting and potentially important observation. However, the current discussion remains largely descriptive, and the mechanistic origin of the orientation trend is not fully clarified. It would be helpful if the authors could address the following points: 1) What structural or electronic features of the Ge-based host are responsible for promoting improved horizontal alignment? For example, could differences in steric bulk, molecular polarizability, intermolecular interactions, or free volume contribute to the observed trend? 2) Is there any change in the intrinsic transition dipole moment (TDM) direction or magnitude of the emitters when embedded in different host environments? Providing computational analysis of TDM vectors or host–guest configurations would help clarify whether the improvement arises from host-induced alignment effects or intrinsic electronic changes. 3) Given that some reported differences in Θ// appear relatively small, could the authors provide uncertainty estimates, fitting sensitivity analysis, or reproducibility data to confirm that the observed C → Si → Ge trend is statistically meaningful? If possible, optical simulations incorporating the measured dipole orientation would help quantify how much of the EQE enhancement can be attributed to improved outcoupling versus intrinsic photophysical improvements. A more quantitative discussion linking molecular orientation, TDM behavior, and device efficiency would substantially strengthen the interpretation of the orientation-dependent performance enhancement.*

Response: We sincerely appreciate the reviewer's constructive and rigorous comments regarding the horizontal dipole ratio ($\Theta_{//}$) trend. Upon receiving your feedback, we have systematically re-evaluated our thin-film characterization and discovered that the originally reported values (particularly the exceptionally high $\Theta_{//}$ of 99.9% for *ω*-DABNA) suffered from an experimental artifact arising from the encapsulation process. Specifically, the UV-curable glue used for thin-film packaging partially dissolved the thin evaporated active layer, which altered the local refractive index and led to inaccurate dipole orientation measurements. To rectify this, we carefully prepared fresh, rigorously encapsulated thin-film samples and performed multiple independent measurements to obtain reliable results with statistical uncertainties. The updated and corrected $\Theta_{//}$ values are now provided in Fig. 4j and Supplementary Fig. 33. For the *t*-DABNA-doped films, the $\Theta_{//}$ are estimated to be 80.9±1.0%, 81.2±0.8% and 81.7±0.7% in the CCzCz:CTrzCz2, SiCzCz:SiTrzCz2 and GeCzCz:GeTrzCz2 hosts, respectively (Supplementary Fig. 33). For the *υ*-DABNA-doped films, the estimated $\Theta_{//}$ values are 87.9±1.8%, 87.1±0.8% and 88.6±2.8% in the carbon, silicon, and germanium-based exciplex hosts, respectively (Supplementary Fig. 33). For the *ω*-DABNA-doped films, the estimated $\Theta_{//}$ values are 88.0±1.7%, 88.2±1.7% and 89.1±2.0% in the carbon, silicon, and germanium-based exciplex hosts, respectively (Fig. 4j).

As clearly demonstrated by these standard deviations, **the minor differences (all less than 1.5%) in horizontal dipole ratios across the different hosts fall within the range of measurement uncertainty (up to 2.8%)**. The introduction of germanium into the exciplex host therefore **does not meaningfully alter** the molecular orientation or transition dipole moment (TDM) behavior of the guest emitters. This consistency is structurally expected because the isomorphic carbon, silicon, and germanium hosts share nearly identical molecular geometries, thereby maintaining an identical local environment for the guest emitters across all three matrixes. Consequently, the performance enhancement is not driven by host-induced alignment effects or intrinsic changes to the TDM vectors.

To further clarify the correlation between molecular orientation and device performance, we performed optical simulations (Supplementary Fig. 34) to determine the theoretical maximum external quantum efficiencies (EQE_{max}) for the corresponding device. Taking the *t*-DABNA guest as a representative example, the theoretical EQE_{max} are calculated to be 28.8%, 29.3% and 30.5% for the carbon, silicon, and germanium-based exciplex hosts, respectively. This indicates that from the carbon- to germanium-based host, the theoretically expected EQE increase is only 1.7%. By contrast, the experimentally measured EQE_{max} values are 16.8%, 26.9% and 30.1% for the carbon, silicon, and germanium-based exciplex hosts, respectively. The experimental EQE enhancement from the carbon- to germanium-based host is 13.3%. Consequently, the EQE enhancement attributed to improved outcoupling accounts for only ~12.8% (1.7%/13.3%×100%) of the totally observed EQE enhancement. A similar trend is also observed for the devices based on the *υ*-DABNA and the *ω*-DABNA guests. This quantitative comparison clearly indicates that the systematic enhancement in experimental device efficiency does not originate from outcoupling or orientation changes. Instead, it is predominantly driven by the differences in exciton utilization efficiency. The germanium-based host system allows the devices to have experimental EQE_{max} values remarkably close to their theoretical limits, confirming that its superior performance is governed by highly efficient triplet-to-singlet spin flip process facilitated by the heavy-atom effect.

We have updated all corresponding orientation data and included the optical simulation analyses in the revised manuscript and Supplementary Information.

#Modified sentences, (manuscript, page 11): "As illustrated in Fig. 4j and Supplementary Fig. 33, they showed comparably high horizontal emitting dipole orientation up to 89.1±2.0%."

Additional sentences, (manuscript, page 11): "Optical simulations performed on the corresponding devices

revealed that the theoretical maximum EQE varied only marginally across the three exciplex hosts (Supplementary Fig. 34). Notably, only the devices based on the GeCzCz:GeTrzCz2 host closely approached their theoretical EQE limits, confirming that their superior performance is driven by highly efficient triplet-to-singlet spin-flip process."

Additional sentence, (manuscript, page 15): "Optical simulations of OLED devices were performed with SETFOS 5.1 (Fluxim) program, using the refractive index, extinction coefficient, and thickness of each layer (obtained by ellipsometry) together with the photoluminescence spectrum of the emitting layer as input parameters."

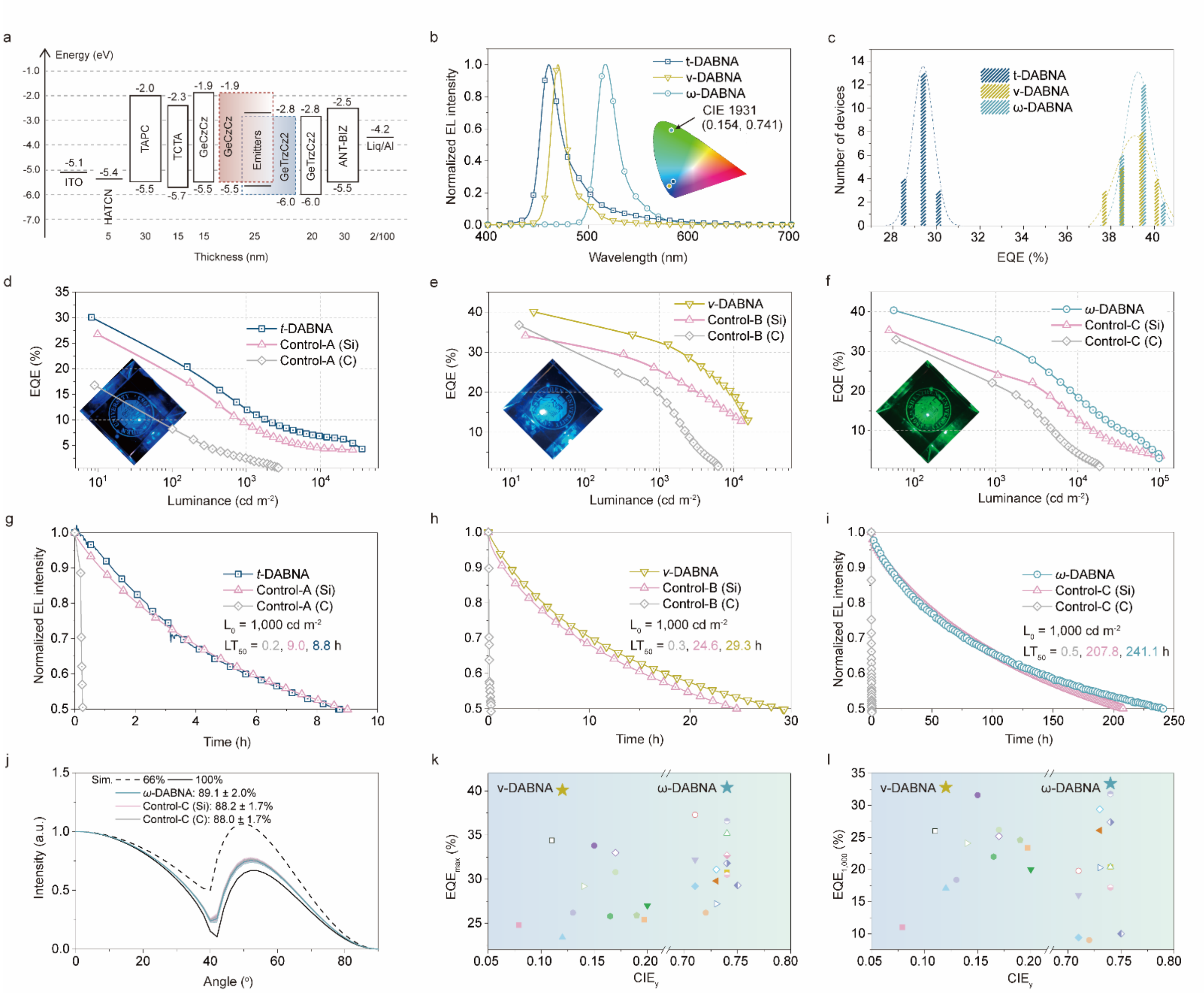

Fig. 4 | Optimized EL properties of OLEDs. a, Diagram of the germanium-based device structure. **b**, EL specrtra recorded at 1,000 cd·m^{-2}. c, Statistical histogram of EQEs for different devices. **d-f**, EQE versus luminance characteristics of target devices (*t*-DABNA (**d**), *υ*-DABNA (**e**), *ω*-DABNA (**f**)) and control devices (control-A (**d**), control-B (**e**), control-C (**f**)). **g**, Half-lifetime (LT_{50}) measurement of *t*-DABNA-based device

and control devices (control-A) at an initial luminance of 1,000 cd·m^{-2}. **h**, LT_{50} measurement of *υ*-DABNA-based device and control devices (control-B) at an initial luminance of 1,000 cd·m^{-2}. **i**, LT_{50} measurement of *ω*-DABNA-based device and control devices (control-C) at an initial luminance of 1,000 cd·m^{-2}. **j**, Angle-dependent PL spectra of *ω*-DABNA-doped films. $\Theta_{//}$, horizontal emitting dipole orientation. **k**,**l**, Summary of CIEy coordinates versus maximum EQE (**k**) values and EQE at 1,000 cd·m^{-2} (**l**) for state-of-the-art blue (CIEy ≤ 0.20) and green (CIEy ≥ 0.71) OLEDs.

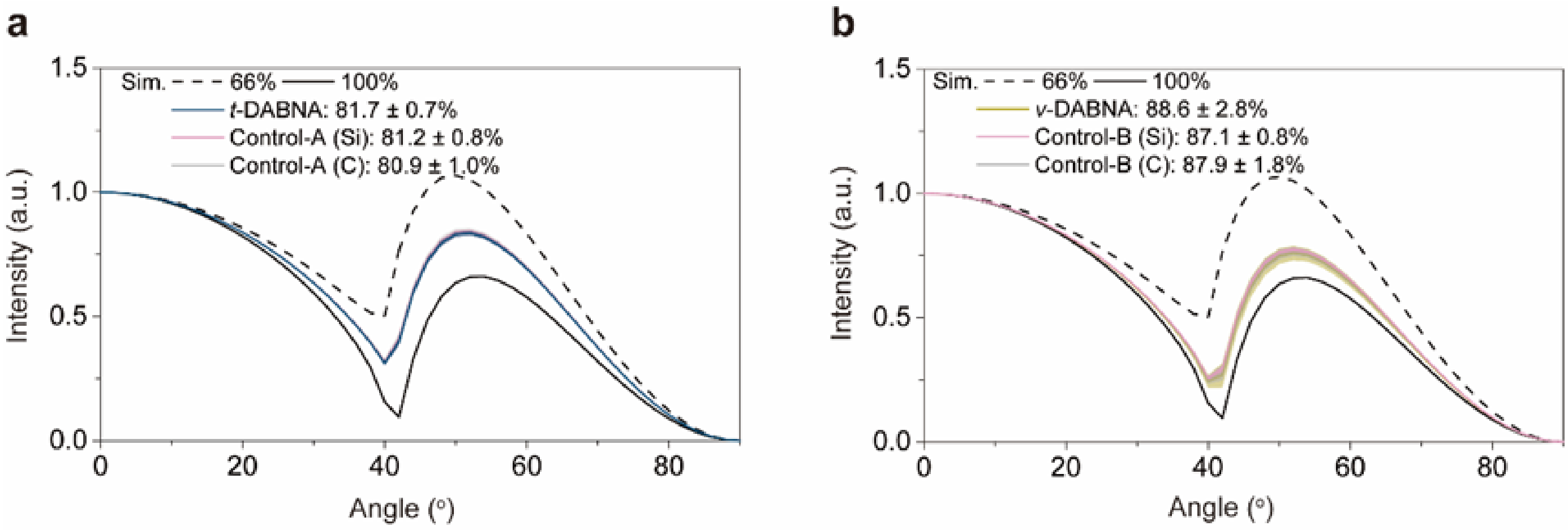

Supplementary Fig. 33. Angle-dependent PL spectra. (**a**) *t*-DABNA-doped films. (**b**) *υ*-DABNA-doped films.

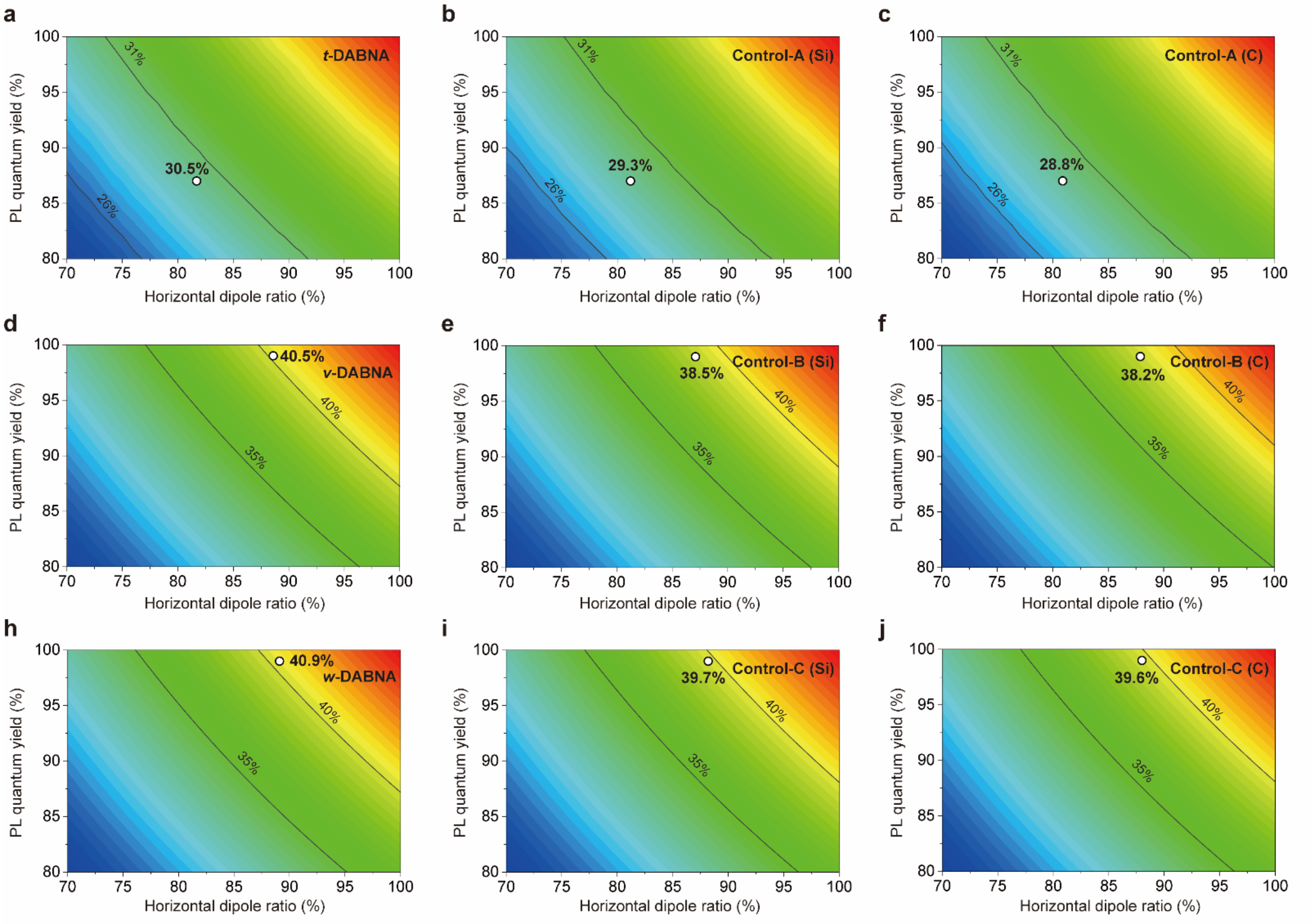

Supplementary Fig. 34. Theoretical maximum external quantum efficiency as a function of the emitting layer PL quantum yield and the horizontal dipole ratio of the emitter for devices. (**a**) *t*-DABNA-based device. (**b**) Control-A (Si) device. (**c**) Control-A (C) device. (**d**) *υ*-DABNA-based device. (**e**) Control-B (Si) device. (**f**) Control-B (C) device. (**h**) *ω*-DABNA-based device. (**i**) Control-C (Si) device. (**j**) Control-C (C) device. The charge balance factor was assumed to be unity.

Comment 5: *While the increase in $k_{r,S}$ and decrease in $k_{nr,S}$ quantitatively explain the enhancement in PLQY, the mechanistic origin of these changes remains insufficiently clarified. 1) In the rebuttal, the reduction in $k_{nr,S}$ is attributed to increased molecular packing inferred from higher crystal density. However, could the authors clarify how crystal density translates into suppressed non-radiative decay in amorphous exciplex films? 2) If tighter molecular packing is proposed to suppress non-radiative decay, how does this interpretation reconcile with the intramolecular reorganization energies reported in Supplementary Fig. 13? A more quantitative discussion linking reorganization energy, vibrational coupling, and non-radiative decay rates would significantly strengthen the mechanistic explanation for the enhanced PLQY. 3) Have mode-specific contributions (e.g., dominant vibrational modes or Huang-Rhys factors) been analyzed to determine whether*

vibrational coupling is indeed reduced in the Ge-based system?

Response: We thank the reviewer for these deeply insightful questions.

1) Although amorphous films lack long-range crystalline order, their local molecular packing density is governed by the same non-covalent intermolecular forces that dictate crystal packing density. Our atomic dipole moment corrected Hirshfeld atomic charge analysis (*J. Theor. Comput. Chem.* **11**, 163 (2012)) reveals that the Ge atom is more positively charged than the Si atom, which polarizes the surrounding molecular groups around Ge relatively more electron-rich (Supplementary Table 1). Since London dispersion forces operate between induced dipoles, these electron-rich groups strengthen the intermolecular London dispersion interactions (*J. Am. Chem. Soc.* **143**, 20837 (2021)). As a result, molecules containing Ge should likely form closer and tighter packing in amorphous exciplex films. This naturally leads to the observed decrease in $k_{nr,S}$.

2) Regarding the reconciliation between intermolecular packing and intramolecular reorganization energy, the tighter intermolecular packing provides a solid-state spatial confinement that further suppresses the geometry relaxations quantified by the intramolecular reorganization energies. As requested, we have performed quantum-chemical calculations of reorganization energies, Huang-Rhys factors, and the theoretical non-radiative decay rates (k_{nr}) for all hole-transport (HT) host and electron-transport (ET) host molecules. These results are presented in Supplementary Figs. 12 and 15.

For the HT hosts, the computed reorganization energies, total Huang-Rhys factors (S) and theoretical k_{nr} are virtually identical and consistently lower than those of the ET hosts, identifying the ET hosts as the primary source of the differences in non-radiative decay. For the ET hosts, CTrzCz2 exhibits a high reorganization energy of 1.03 eV, while both SiTrzCz2 and GeTrzCz2 exhibit identical and reduced reorganization energies of 0.93 eV (Supplementary Fig. 12). More significantly, the total S, which quantifies the overall vibrational coupling, drops dramatically from 103.87 in CTrzCz2 to 37.72 in SiTrzCz2 and further to 35.63 in GeTrzCz2 (Supplementary Fig. 15). Consequently, the theoretical k_{nr} follows a corresponding trend, dropping from 18.30 $\times 10^9$ s^{-1} (CTrzCz2) to 4.98 $\times 10^9$ s^{-1} (SiTrzCz2) and further to 1.44 $\times 10^9$ s^{-1} (GeTrzCz2), which is qualitatively consistent with the experimental enhancement in PLQY of the Ge-based system; meanwhile, the relatively larger theoretical k_{nr} (compared to the experimental k_{nr}) implies that the non-radiative decay is likely to be further suppressed by intermolecular packing in the films.

3) To identify which dominant vibrational modes are affected in the Ge-based system, we have performed a detailed mode-specific analysis (Supplementary Fig. 15). Our results show that the substitution of germanium suppresses the Huang-Rhys factor of several dominant skeletal vibrational modes, which are primarily associated with aromatic C-H in-plane bending coupled with C-N/C-C stretching in the carbazole

and triazine units). For instance, the Huang-Rhys factor for the skeletal mode around 1140 cm^{-1} is reduced from 0.12733 in SiTrzCz2 to 0.07084 in GeTrzCz2; similarly, the Huang-Rhys factor for the skeletal mode around 1146 cm^{-1} is reduced from 0.09338 in SiTrzCz2 to 0.06557 in GeTrzCz2 (Supplementary Table 3).

Taken together, these results indicate that the enhanced PLQY in the Ge-based system likely arises from a **synergistic interaction** between the **intermolecular** spatial confinement (driven by tighter packing) and reduced **intramolecular** vibrational coupling, which collectively suppress the non-radiative decay.

Modified sentence, (manuscript, page 5): "…germanium incorporation promotes closer molecular packing through stronger intermolecular dispersion interactions (Supplementary Table 1), which may in turn help to suppress the non-radiative decay."

Additional sentence, (manuscript, page 6): "The reduced $k_{nr,S}$ is likely driven by a synergistic combination of closer intermolecular packing and suppressed intramolecular vibrational coupling in GeTrzCz2 compared to SiTrzCz2 (Supplementary Fig. 15 and Supplementary Table 3)."

Additional sentence, (Supplementary Information, Page S54): "The atomic dipole moment corrected Hirshfeld atomic charge analysis reveals that the Ge atom is more positively charged than the Si atom, which polarizes the surrounding molecular groups around Ge relatively more electron-rich. Since London dispersion forces operate between induced dipoles, these electron-rich groups strengthen the intermolecular London dispersion interactions."

Supplementary Table 1. Calculated atomic dipole moment corrected Hirshfeld atomic charges for the individual atoms in the Ge- and Si-based hosts. The atomic numbering corresponds to the labeled positions in the depicted chemical structures.

Atoms	Number	GeTrzCz2	SiTrzCz2	Atoms	Number	GeCzCz	SiCzCz
**Ge/Si**	**-**	**0.43838**	**0.37829**	**Ge/Si**	**-**	**0.66454**	**0.52894**
C	1	-0.29945	-0.19723	C	1	-0.16306	-0.15245
C	2	-0.02483	-0.03618	C	2	-0.10109	-0.11121
C	3	-0.08559	-0.10089	C	3	0.08511	0.08364
C	4	-0.08374	-0.07590	C	4	-0.06485	-0.05969
C	5	-0.08769	-0.09131	C	5	-0.05575	-0.02871
C	6	-0.05763	-0.08075	C	6	-0.16554	-0.16555
C	7	-0.12927	-0.15235	C	7	-0.30057	-0.23181
C	8	-0.11387	-0.03838	C	8	-0.03698	-0.06403
C	9	-0.11978	-0.11455	C	9	-0.10472	-0.10549
C	10	-0.12408	-0.12990	C	10	-0.15041	-0.13040
C	11	-0.11408	-0.11468	C	11	-0.09899	-0.09731
C	12	-0.11153	-0.11322	C	12	-0.00305	-0.05434

C	13	-0.05123	-0.09434	C	13	-0.21336	-0.18135
C	14	-0.14805	-0.07453	C	14	-0.07680	-0.08553
C	15	-0.04061	-0.07206	C	15	-0.11212	-0.10769
C	16	-0.44770	-0.40916	C	16	-0.13736	-0.12418
C	17	-0.22673	-0.22549	C	17	-0.10886	-0.10915
C	18	-0.02867	0.0107	C	18	-0.08563	-0.09028
C	19	-0.15636	-0.14701	C	19	-0.34926	-0.27365
C	20	-0.05963	-0.07486	C	20	0.11063	0.01359
C	21	-0.11924	-0.13317	C	21	-0.09372	-0.07644
C	22	-0.14484	-0.15146	C	22	-0.15539	-0.12017
C	23	-0.11581	-0.01936	C	23	-0.09295	-0.16648
C	24	-0.00677	-0.04181	C	24	-0.07343	-0.07047

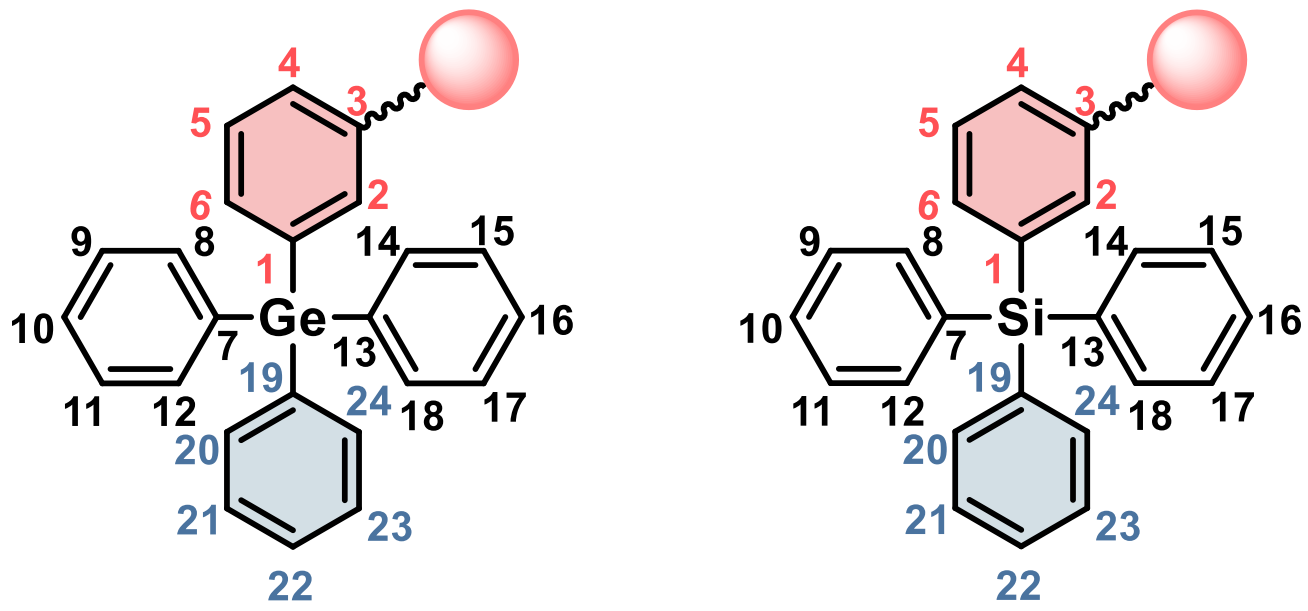

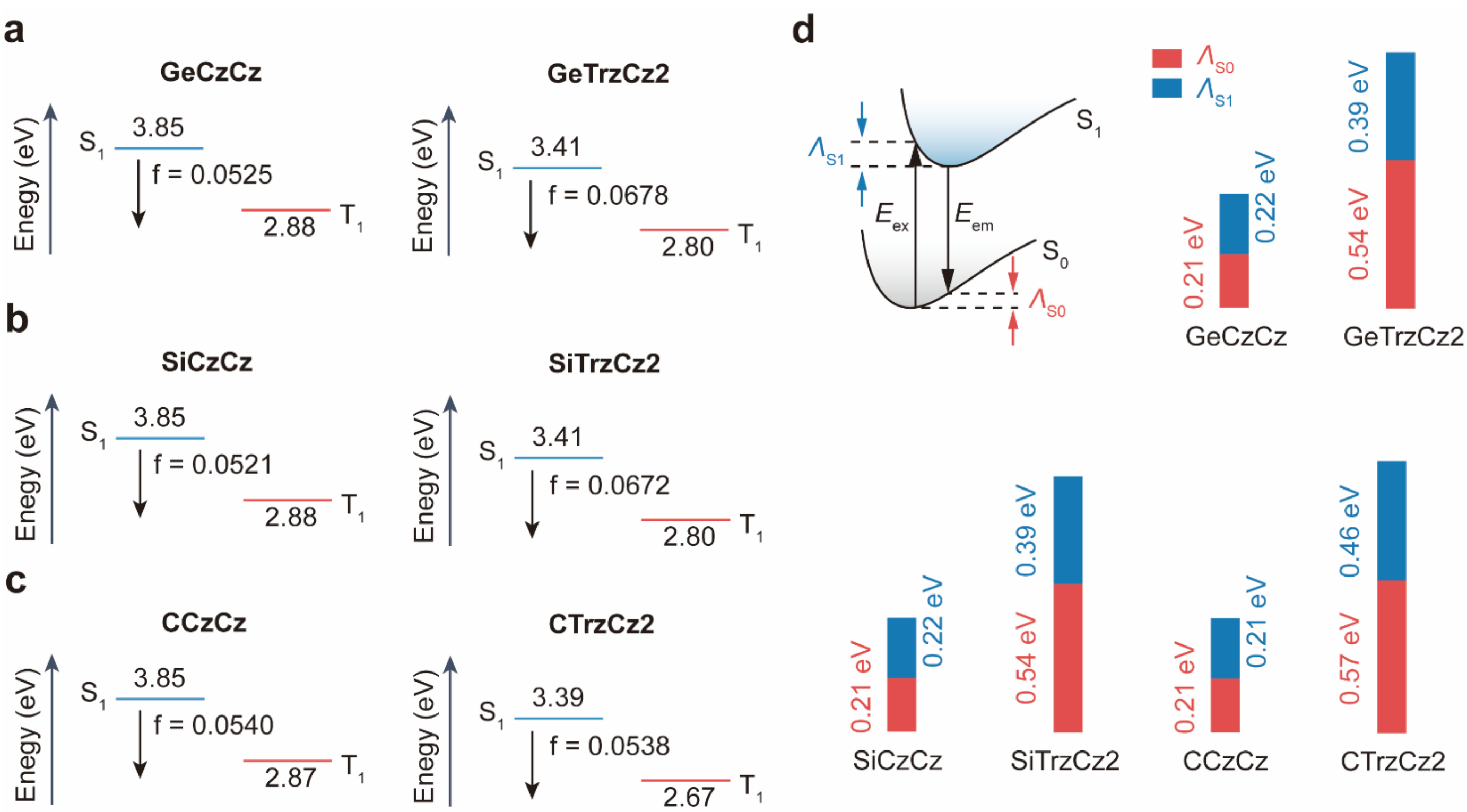

Supplementary Fig. 12. Theoretical calculations of the hosts. (**a**) Transition energies and oscillator strengths of GeCzCz and GeTrzCz2. (**b**) Transition energies and oscillator strengths of SiCzCz and SiTrzCz2. (**c**) Transition energies and oscillator strengths of CCzCz and CTrzCz2. (**d**) Reorganization energy.

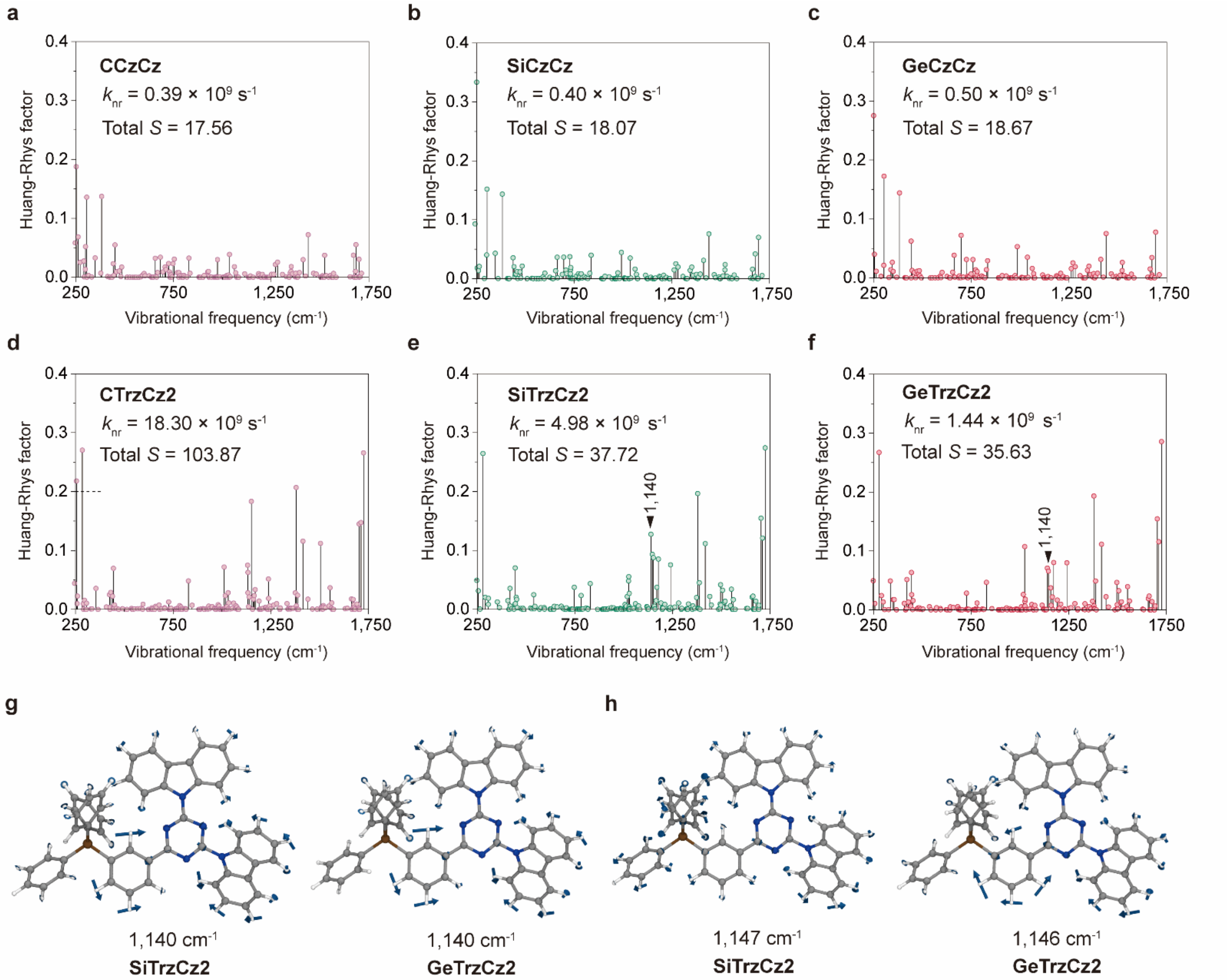

Supplementary Fig. 15. Theoretically calculated Huang-Rhys factor (*S*) and non-radiative decay rate (k_{nr}) for the $S_1 \rightarrow S_0$ electronic transitions plotted against the vibrational frequency. (**a**) CCzCz. (**b**) SiCzCz. (**c**) GeCzCz. (**d**) CTrzCz2. (**e**) SiTrzCz2. (**f**) GeTrzCz2. (**g**) Frequency 1,140 cm^{-1} plotted on the optimized S_1 geometry of SiTrzCz2 and GeTrzCZ2. (**h**) Frequency 1,147 cm^{-1} plotted on the optimized S_1 geometry of SiTrzCz2 and frequency 1,146 cm^{-1} plotted on the optimized S_1 geometry of GeTrzCz2.

Supplementary Table 3. Calculated Huang-Rhys factors (*S*) for the dominant skeletal vibrational modes (where *S* > 0.06) of SiTrzCz2 and GeTrzCz2.

SiTrzCz2		GeTrzCz2	
**Vibrational frequency / cm^{-1}**	**Huang-Rhys factor**	**Vibrational frequency / cm^{-1}**	**Huang-Rhys factor**
278.90	0.26444	278.44	0.26717
444.25	0.07025	444.11	0.06345
1139.58	0.12733	1140.32	0.07084

1146.80	0.09338	1146.23	0.06557
1177.92	0.08535	1173.02	0.08032
1240.36	0.07541	1240.40	0.07988
1379.29	0.19652	1379.17	0.19316
1417.93	0.11157	1417.97	0.11139
1703.76	0.15470	1703.85	0.15454
1711.48	0.12094	1711.68	0.11559
1725.06	0.27407	1725.34	0.28561

Comment 6: *Supplementary Fig. 20 includes hole-only and electron-only device (HOD/EOD) measurements for the Ge-based system. However, a direct comparison with the corresponding C- and Si-based analogues is not clearly presented. If feasible, it would be beneficial to fabricate and characterize HOD/EOD devices for the C- and Si-based host systems under identical conditions. Such comparative data would allow a more quantitative assessment of whether the improved performance in the Ge-based system indeed arises from enhanced charge transport or balance, rather than from purely photophysical effects.*

Response: We thank the reviewer for this very reasonable suggestion. In the revised manuscript, we have performed single-carrier transport analysis by fabricating both hole-only (HOD) and electron-only devices (EOD) for the SiCzCz:SiTrzCz2 and CCzCz:CTrzCz2 systems under identical conditions (Supplementary Fig. 21). The estimated hole and electron mobilities for SiCzCz:SiTrzCz2 are 2.87×10^{-5} $cm^2 \cdot V^{-1} \cdot s^{-1}$ and 5.78×10^{-5} $cm^2 \cdot V^{-1} \cdot s^{-1}$, while those of CCzCz:CTrzCz2 are 2.44×10^{-5} $cm^2 \cdot V^{-1} \cdot s^{-1}$ and 5.61×10^{-5} $cm^2 \cdot V^{-1} \cdot s^{-1}$, respectively. These values lie within the same order of magnitude as those of the GeCzCz:GeTrzCz2 system (hole mobility: 3.07×10^{-5} $cm^2 \cdot V^{-1} \cdot s^{-1}$; electron mobility: 5.69×10^{-5} $cm^2 \cdot V^{-1} \cdot s^{-1}$). Furthermore, we evaluated the charge balance by calculating the electron-to-hole mobility ratio, which yields 2.30 for CCzCz:CTrzCz2, 2.01 for SiCzCz:SiTrzCz2, and 1.85 for GeCzCz:GeTrzCz2. The Ge-based host exhibits a marginally more balanced carrier transport. Therefore, these results demonstrate that the improved performance of the Ge-based system predominantly originates from the heavy-atom effect of germanium, with the marginally improved charge balance acting as a minor, beneficial auxiliary factor.

Modified sentence, (manuscript, page 8): "…GeCzCz:GeTrzCz2 (0.50/0.50 w/w) host provides efficient and relatively more balanced charge transport…"

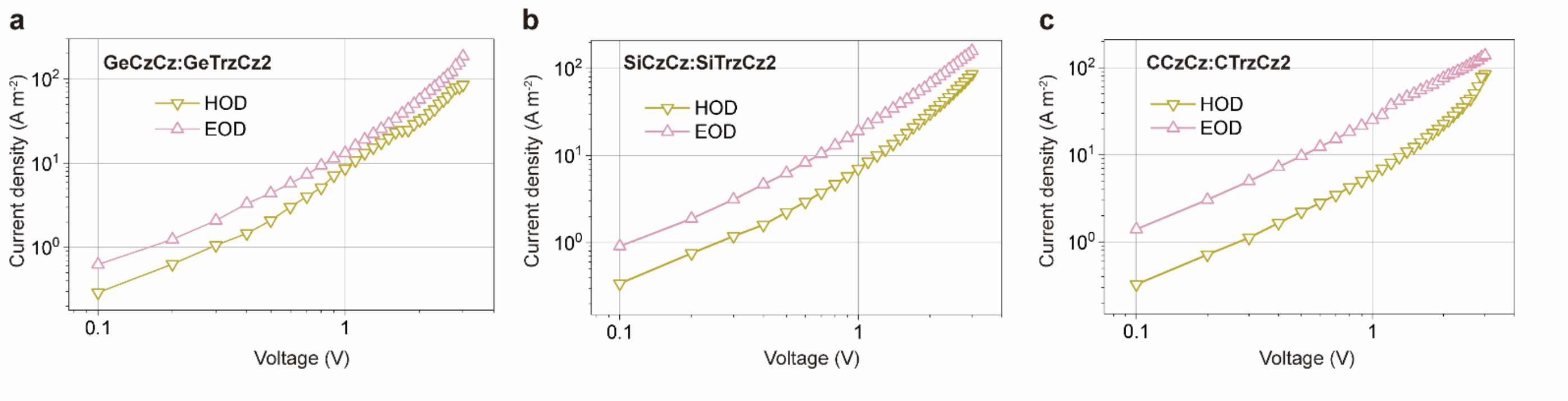

Supplementary Fig. 21. Current density-voltage characteristics of the hole-only device (HOD) and electron-only device (EOD). (**a**) The device structure of the HOD and EOD were constructed as ITO/HATCN (5 nm)/GeCzCz:GeTrzCz2 (100 nm, 0.50/0.50 w/w)/HATCN (20 nm)/Al (100 nm) and ITO/Al_2O_3 (30 nm)/Liq (2 nm)/GeCzCz:GeTrzCz2 (100 nm, 0.50/0.50 w/w)/Liq (2 nm)/Al (100 nm), respectively. (**b**) The device structure of the HOD and EOD were constructed as ITO/HATCN (5 nm)/SiCzCz:SiTrzCz2 (100 nm, 0.50/0.50 w/w)/HATCN (20 nm)/Al (100 nm) and ITO/Al_2O_3 (30 nm)/Liq (2 nm)/SiCzCz:SiTrzCz2 (100 nm, 0.50/0.50 w/w)/Liq (2 nm)/Al (100 nm), respectively. (**c**) The device structure of the HOD and EOD were constructed as ITO/HATCN (5 nm)/CCzCz:CTrzCz2 (100 nm, 0.50/0.50 w/w)/HATCN (20 nm)/Al (100 nm) and ITO/Al_2O_3 (30 nm)/Liq (2 nm)/CCzCz:CTrzCz2 (100 nm, 0.50/0.50 w/w)/Liq (2 nm)/Al (100 nm), respectively.

Comment 7: *Given that the carbon-based device exhibits the shortest operational lifetime, a clearer explanation of why the C system is more susceptible to degradation from an exciton-dynamics perspective would strengthen the argument. It would also be helpful to clarify whether differences in thin-film properties among the C-, Si-, and Ge-based systems contribute to the stability trend, enabling a more systematic comparison across the series.*

Response: We thank the reviewer for this constructive suggestion. To clarify why the carbon-based device shows the shortest operational lifetime from the perspective of exciton dynamics, we have measured the transient electroluminescence decay curves of the devices based on the CCzCz:CTrzCz2, SiCzCz:SiTrzCz2, and GeCzCz:GeTrzCz2 hosts, using the *υ*-DABNA guest as a representative example (Supplementary Fig. 35). The carbon-based device exhibited a delayed lifetime of 95.60 μs, which is significantly longer than those of the silicon-based device (67.30 μs) and the germanium-based device (34.20 μs). This prolonged delayed lifetime indicates a substantial accumulation of long-lived triplet excitons in the emitting layer of the carbon-based device, which promotes destructive bimolecular annihilation processes (e.g., triplet-triplet and triplet-polaron annihilation), thereby accelerating molecular degradation during device operation (*Adv. Mater.* **37**,

2419217 (2025); *Adv. Funct. Mater.* **36**, e27439 (2026)).

We also have measured the photoluminescence stability of *t*-DABNA, *υ*-DABNA, and *ω*-DABNA-doped thin films among the C-, Si-, and Ge-based systems under continuous 365 nm LED irradiation (12 W) at room temperature (Supplementary Fig. 36). For all three guests, the carbon-based films degraded most rapidly, following the same trend observed for the device lifetimes.

Additional sentences, (manuscript, page 12): "Using *υ*-DABNA as a representative guest to probe the underlying exciton dynamics, transient electroluminescence measurements (Supplementary Fig. 35) revealed a significantly longer delayed lifetime for the carbon-based device (95.60 μs) than those of the silicon-based device (67.30 μs) and germanium-based device (34.20 μs). This prolonged delayed lifetime indicates a substantial accumulation of long-lived triplet excitons in the carbon-based emitting layer, which promotes destructive bimolecular annihilation to accelerate molecular degradation. This behavior is further supported by photoluminescence stability tests under continuous UV irradiation, where the carbon-based films consistently degraded the most rapidly (Supplementary Fig. 36)."

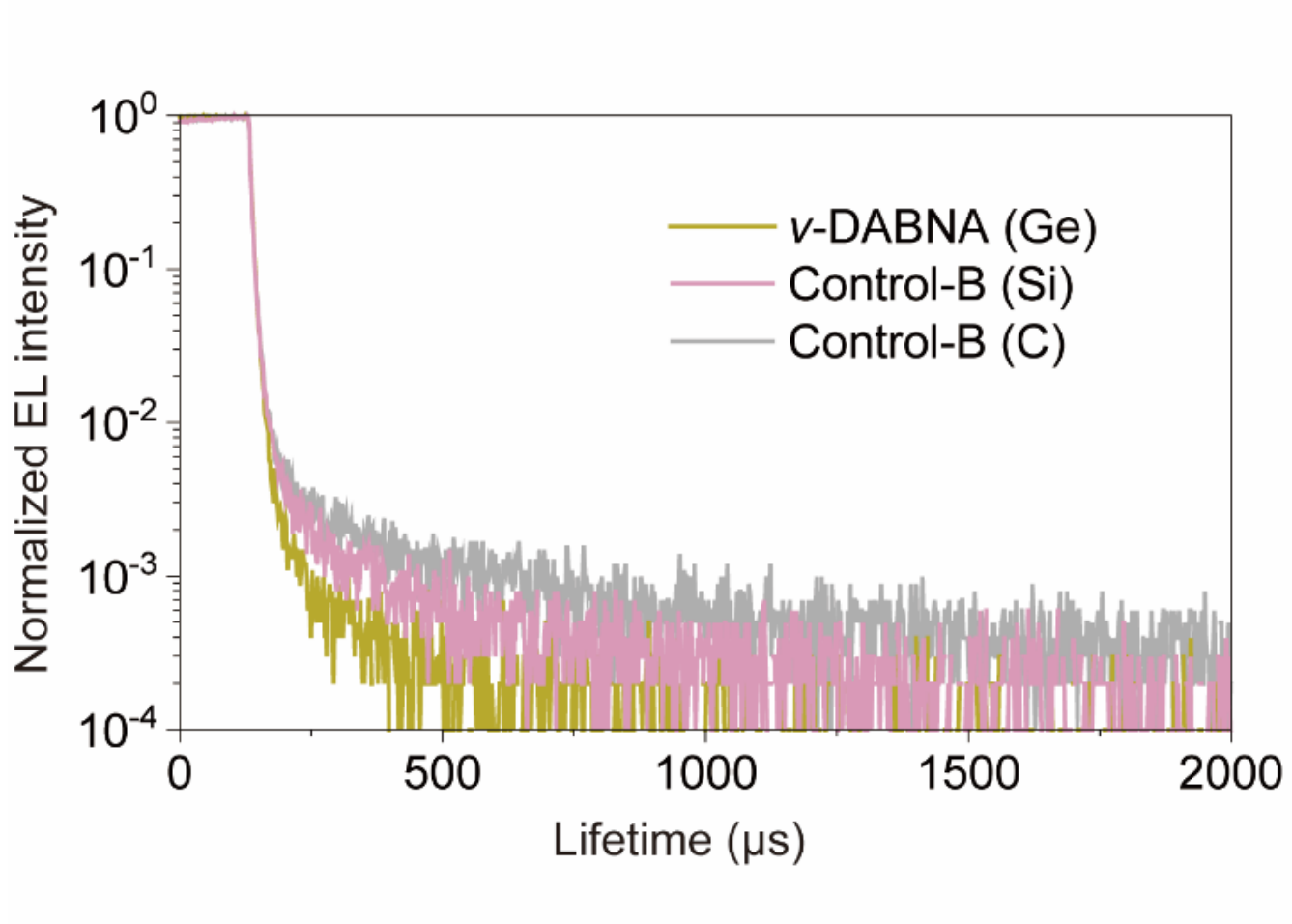

Supplementary Fig. 35. EL transient decay curves of *υ*-DABNA-based device and control devices (control-B), measured at their respective dominant emission peaks.

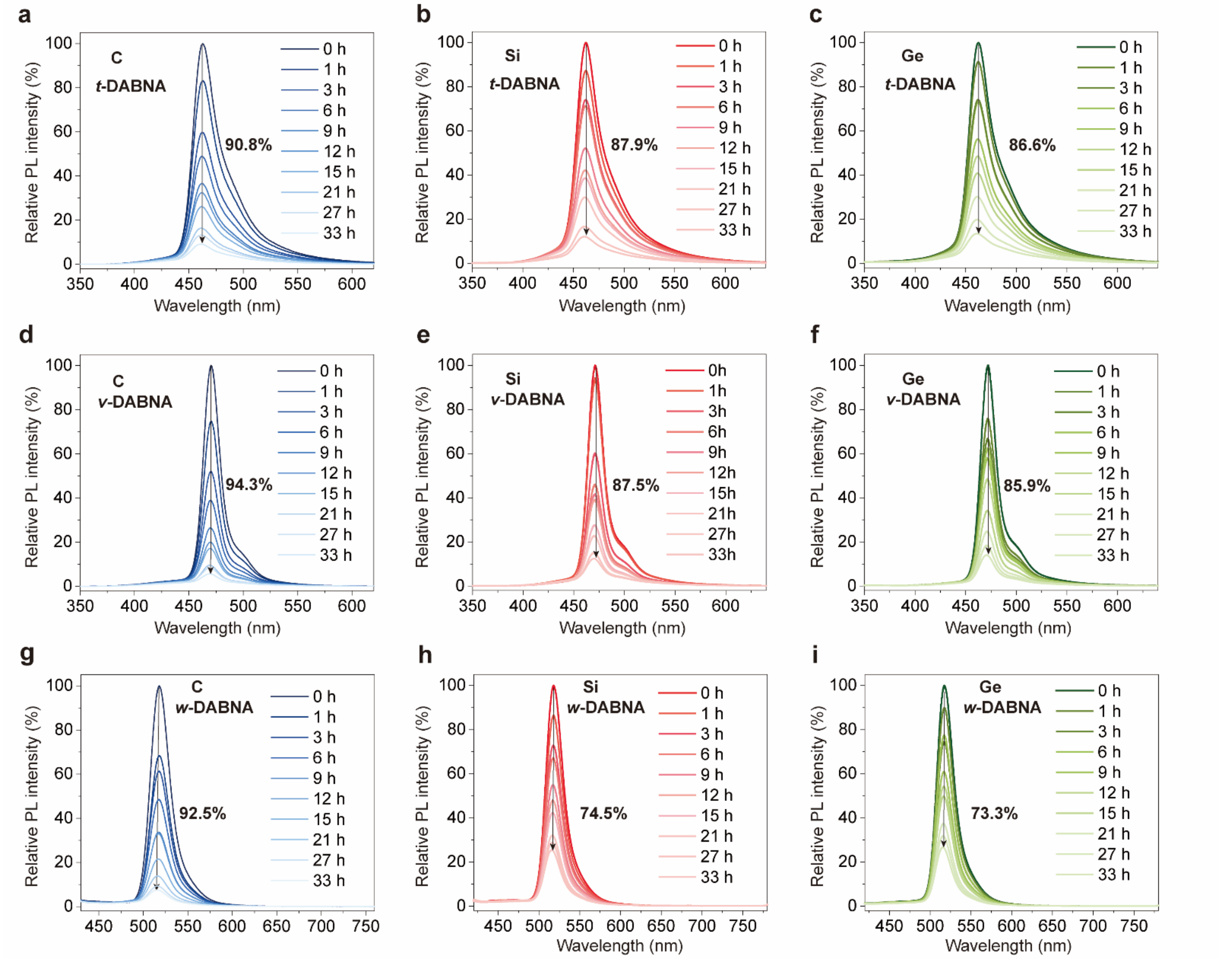

Supplementary Fig. 36. Photolysis results. PL spectra of **(a)** CCzCz:CTrzCz2:*t*-DABNA, **(b)** SiCzCz:SiTrzCz2:*t*-DABNA, **(c)** GeCzCz:GeTrzCz2:*t*-DABNA, **(d)** CCzCz:CTrzCz2:*υ*-DABNA, **(e)** SiCzCz:SiTrzCz2:*υ*-DABNA, **(f)** GeCzCz:GeTrzCz2:*υ*-DABNA, **(g)** CCzCz:CTrzCz2:*ω*-DABNA, **(h)** SiCzCz:SiTrzCz2:*ω*-DABNA, **(i)** GeCzCz:GeTrzCz2:*ω*-DABNA over 33 h under continuous 365 nm LED irradiation (12 W) at room temperature.

Comment 8: *Given that the carbon-based device exhibits the shortest operational lifetime, it would be helpful to clarify whether its electrochemical stability differs systematically from the Si- and Ge-based systems. If cyclic voltammetry (CV) data are available for the C analogue, a brief comparison of oxidation/reduction behavior may provide additional insight into its relative device stability. Even a short discussion on whether electrochemical robustness correlates with the observed lifetime trend would further strengthen the stability analysis.*

Response: We thank the reviewer for this helpful suggestion. We have repeated the cyclic voltammetry (CV)

measurements for the carbon-based compounds (CCzCz and CTrzCz2) over 20 consecutive cycles (Supplementary Fig. 37). Their electrochemical behavior is comparable to that of the silicon- and germanium-based materials, indicating the electrochemical stability does not correlate with the observed device lifetime trend in this series.

Additional sentence, (manuscript, page 12): "Meanwhile, consecutive cyclic voltammetry scans (Supplementary Fig. 37) revealed comparable electrochemical stability among these host materials, confirming that their electrochemical stability does not correlate with the observed device lifetime trend."

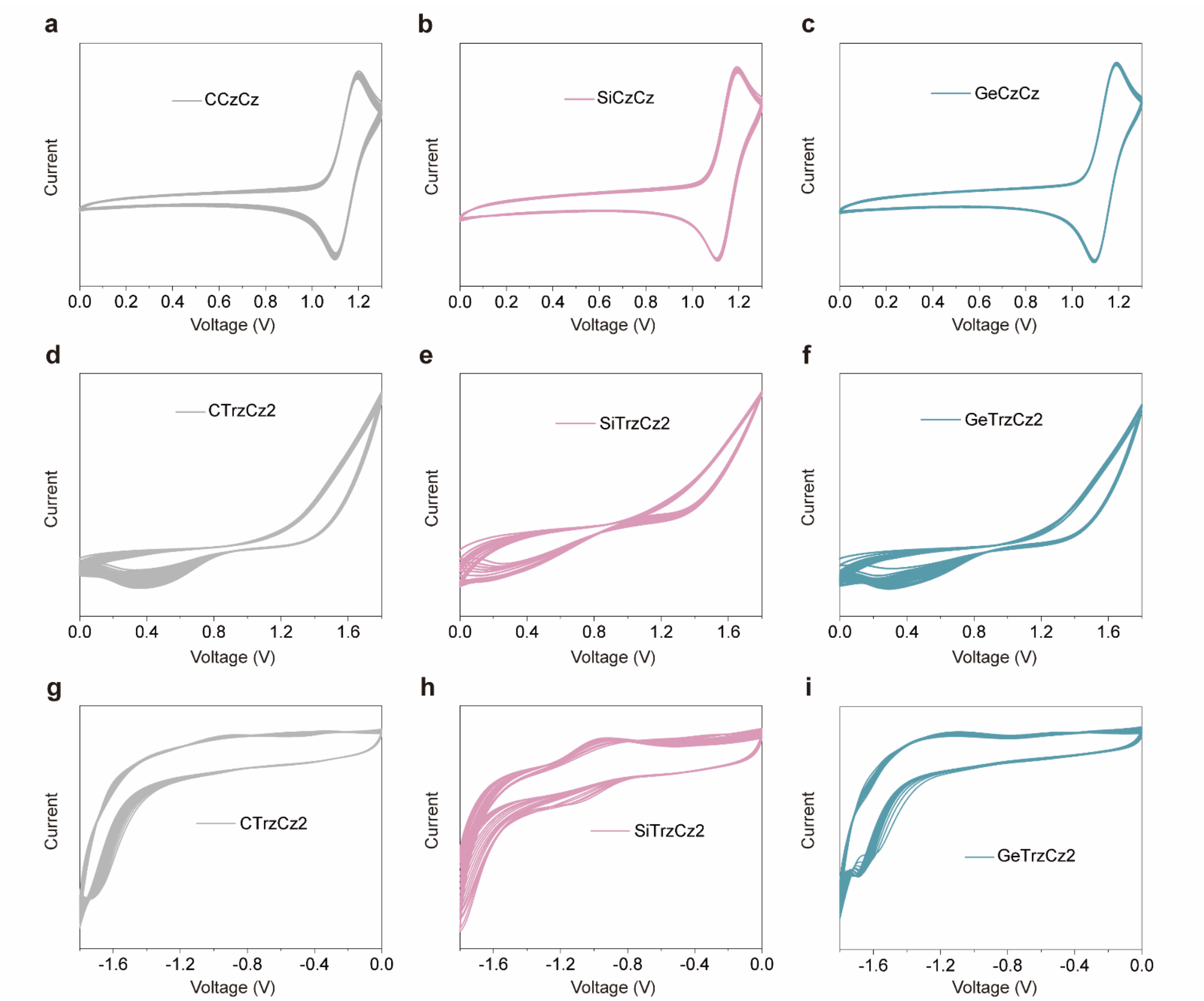

Supplementary Fig. 37. Cyclic voltammograms of the hosts. Oxidation waves of (**a**) CCzCz, (**b**) SiCzCz, (**c**) GeCzCz, (**d**) CTrzCz2, (**e**) SiTrzCz2, (**f**) GeTrzCz2 and reduction waves of (**g**) CTrzCz2, (**h**) SiTrzCz2, (**i**) GeTrzCz2.

Comment 9: *Minor points: Given the very high in-plane dipole orientation reported (up to ~99%), the authors state in the rebuttal that κ^2 = 2/3 was assumed in the R_{FRET} calculation. It would be helpful if the authors could clarify whether this isotropic assumption remains appropriate under such strongly anisotropic dipole distributions, and whether the use of κ^2 = 2/3 introduces any significant deviation in the estimated R_{FRET} values. A brief discussion on the potential impact of dipole alignment on the FRET analysis would improve internal consistency.*

Response: We appreciate the reviewer's valuable and insightful comment. The orientation factor κ^2 is strictly determined by the relative spatial angles between the transition dipole moments (TDMs) of the energy donor (host) and the acceptor (guest), **rather than the orientation of a single species relative to the substrate surface**. Although the acceptor guest molecules (such as *ω*-DABNA) exhibit a highly aligned horizontal TDM orientation relative to the substrate surface, the donor host molecules (CCzCz:CTrzCz2, SiCzCz:SiTrzCz2, and GeCzCz:GeTrzCz2) are completely amorphous and possess largely isotropic, random TDM orientations (*Chem. Mater.* **30**, 5648-5654 (2018)). Consequently, adopting κ^2 = 2/3 is a well-accepted consensus in the existing literatures for such amorphous host-guest systems (*Nat. Commun*. **11**, 1292 (2020); *Sci. Adv.* **8**, eabp9203 (2022)).

Additionally, the estimated Förster radius (R_{FRET}) is proportional to the sixth root of the orientation factor ($R_{FRET} \propto (\kappa^2)^{\frac{1}{6}}$). Due to this mathematical sixth-root relationship, any potential deviation in κ^2 only shifts the R_{FRET} value by less than a single decimal place. This minor variation has basically no impact on the established trends of our FRET analysis.

Taken together, given the amorphous nature of our host matrix, the isotropic assumption of κ^2 = 2/3 remains a reasonable approximation for this host-guest system.

Modified sentence, (manuscript, page 15): "…is assumed to be 2/3 because, despite the highly aligned dipoles of the guest acceptors, the completely amorphous donor hosts statistically ensure an isotropic relative orientation;"

Comment 10: *It would also be helpful to clarify whether the same FRET analysis was performed for the carbon-based analogue, to consistently establish the C → Si → Ge progression.*

Response: We sincerely thank the reviewer for the constructive comment. In the revised manuscript, we have performed the same FRET analysis for the carbon-based analogue. Based on spectral overlap between absorption spectrum of the emitter and PL spectra of the CCzCz:CTrzCz2 exciplex host (Supplementary Fig.

39), the Förster radius for *t*-DABNA, *υ*-DABNA, and *ω*-DABNA were calculated as 4.7 nm, 7.6 nm, and 8.3 nm, respectively. These values are remarkably close to those obtained for the silicon- and germanium-based host systems, which are 4.7 nm, 7.6 nm, and 8.5 nm for *t*-DABNA, *υ*-DABNA, and *ω*-DABNA guests, respectively.

Additional sentence, (Supplementary Information, Page S53): "Based on spectral overlap between absorption spectrum of the emitter and PL spectra of the CCzCz:CTrzCz2 exciplex host, the Förster radius for *t*-DABNA, *υ*-DABNA, and *ω*-DABNA were calculated as 4.7 nm, 7.6 nm, and 8.3 nm, respectively."

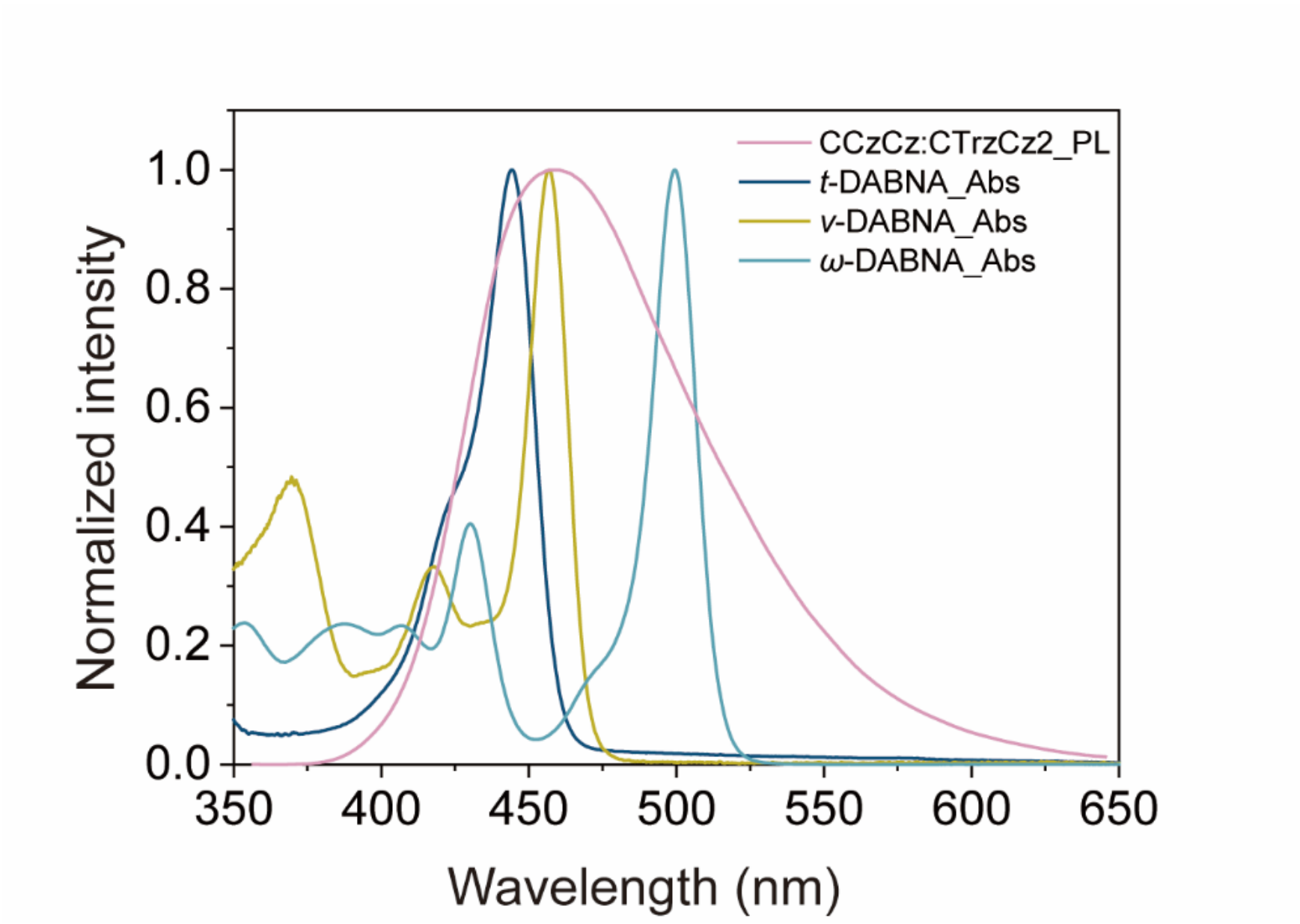

Supplementary Fig. 39. Normalized PL spectra of CCzCz:CTrzCz2 exciplex film and absorption spectrum of *t*-DABNA, *υ*-DABNA and *ω*-DABNA in toluene (10^{-5} mol·L^{-1}).

Comment 11: *It is stated that a refractive index of n = 1.7 was assumed in the R_{FRET} calculation. Since the Förster radius depends on n^4, the estimated R_{FRET} values can be sensitive to the choice of refractive index. It would be helpful if the authors could clarify whether the value n = 1.7 was experimentally measured for the present host films or adopted as a general assumption. If it is an assumed value, a brief discussion on the sensitivity of R_{FRET} to variations in n (e.g., within a realistic range of organic thin films) would strengthen the quantitative robustness of the FRET analysis.*

Response: We sincerely appreciate the reviewer's constructive and helpful suggestion. To ensure the quantitative accuracy and scientific rigor of our analysis, we have measured the actual refractive indices (*n*) of the CCzCz:CTrzCz2, SiCzCz:SiTrzCz2, and GeCzCz:GeTrzCz2 host films using spectroscopic

ellipsometry (Supplementary Fig. 38). Previously, assuming a constant refractive index of n = 1.7 yielded R_{FRET} values of 4.8 nm, 7.8 nm, and 8.6 nm for *t*-DABNA, *υ*-DABNA, and *ω*-DABNA in the germanium-based system, respectively. When we incorporated the experimentally determined wavelength-dependent refractive index curves into our calculations, the corrected R_{FRET} for *t*-DABNA, *υ*-DABNA, and *ω*-DABNA became 4.7 nm, 7.6 nm, and 8.5 nm, respectively. This comparison demonstrates that the deviation between the original and corrected R_{FRET} values is very small (approximately 2-3%), which does not alter the established trends or the conclusions of our study.

Modified section (Methods, Page 14):

"**Analyses of Förster radius**

The rate of Förster resonance energy transfer (k_{FRET}) can be expressed as:

$$k_{FRET} = k_{r,D}\frac{\kappa^2}{R^6}\left(\frac{9000(\ln 10)}{128\pi^5 N_A}\right)\int_0^\infty \frac{F_D(\lambda)\varepsilon_A(\lambda)\lambda^4}{[n(\lambda)]^4}d\lambda. \quad (1)$$

where R is the intermolecular distance of the donor (GeCzCz:GeTrzCz2 exciplex host, SiCzCz:SiTrzCz2 exciplex host and CCzCz:CTrzCz2 exciplex host) and acceptor (*t*-DABNA, *υ*-DABNA, and *ω*-DABNA); $k_{r,D}$ is the radiative decay rate constant of the donor (GeCzCz:GeTrzCz2 exciplex host, SiCzCz:SiTrzCz2 exciplex host and CCzCz:CTrzCz2 exciplex host) in absence of the acceptor (*t*-DABNA, *υ*-DABNA, and *ω*-DABNA); κ^2 is a configurational factor describing the relative orientation of electric transition dipole moments of the donor (GeCzCz:GeTrzCz2 exciplex host, SiCzCz:SiTrzCz2 exciplex host and CCzCz:CTrzCz2 exciplex host) and acceptor (*t*-DABNA, *υ*-DABNA, and *ω*-DABNA) and is assumed to be 2/3 because, despite the highly aligned dipoles of the guest acceptors, the completely amorphous donor hosts statistically ensure an isotropic relative orientation; N_A is the Avogadro constant; $n(\lambda)$ is the refractive index of the donor (Supplementary Fig. 38); $F_D(\lambda)$ is the normalized emission spectra of the donor (GeCzCz:GeTrzCz2 exciplex host, SiCzCz:SiTrzCz2 exciplex host and CCzCz:CTrzCz2 exciplex host); $\varepsilon_A(\lambda)$ is the molar absorption coefficient of the acceptor (*t*-DABNA, *υ*-DABNA, and *ω*-DABNA); and λ is the corresponding wavelength. The Förster radius (R_{FRET}) is defined as an intermolecular distance at which the k_{FRET} is equal to the $k_{r,D}$."

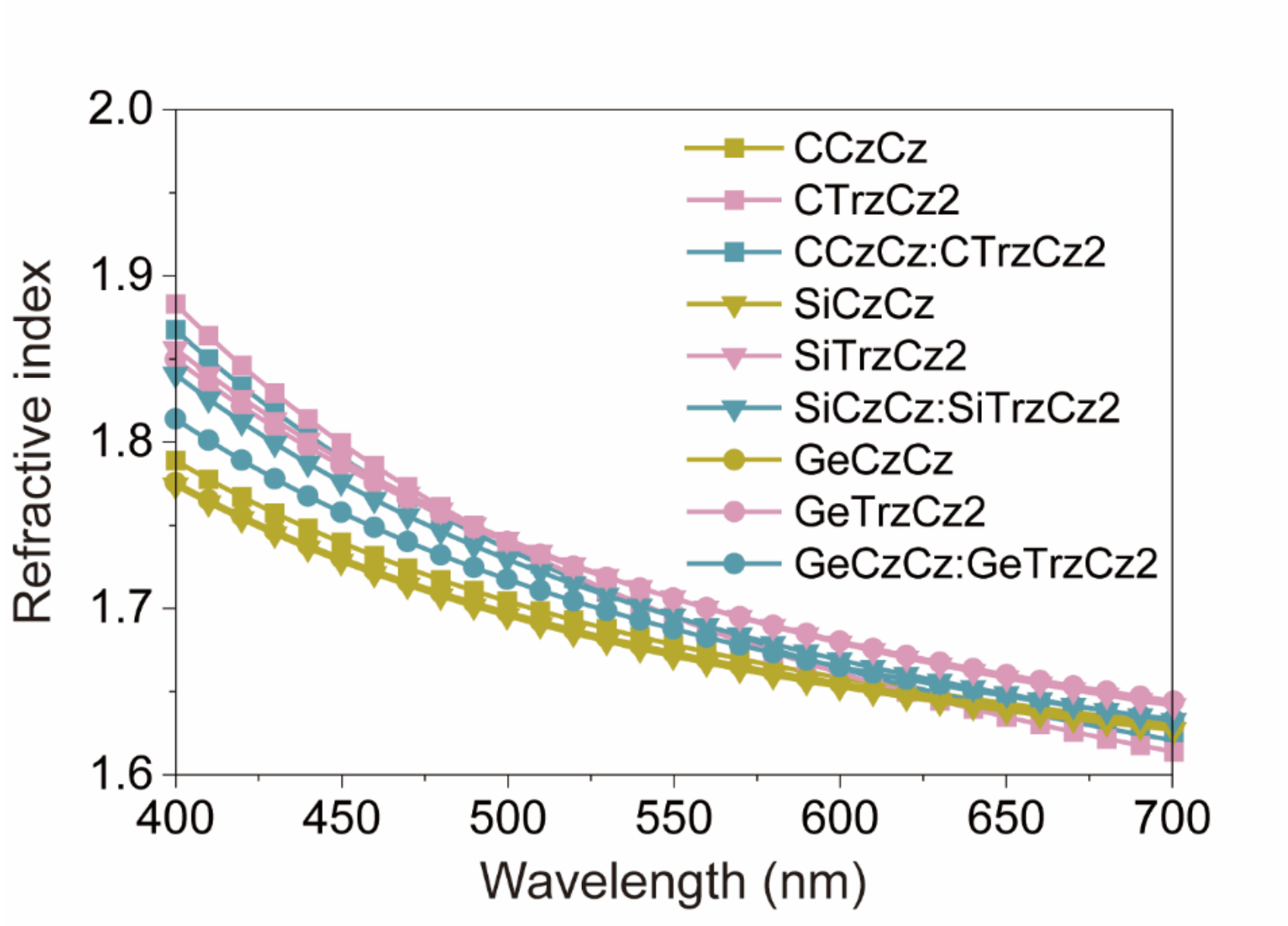

Supplementary Fig. 38. Refractive indices of the charge blocking layers and emitting layers.

Comment 12: *Supplementary Fig. 14 presents calculated bond dissociation energies (BDEs) for the host systems. It would be helpful if the authors could clarify in which electronic state the BDE calculations were performed.*

Response: We thank the reviewer for this careful observation. The bond dissociation energy calculations were performed in the lowest triplet state. This state is commonly used to evaluate the intrinsic molecular stability of OLED materials, as reported in existing literatures (*Nat. Commun*. **14**, 3927 (2023); *Chem. Mater.* **31**, 1507-1519 (2019)).

Modified sentence, (Supplementary Information, Page S60): "…**bond dissociation energies (eV) in the lowest triplet state**."

Comment 13: *Supplementary Fig. 13 presents the calculated parameters for the Ge-based system. For completeness and consistency, it would be helpful if the corresponding calculated data for the C- and Si-based analogues could also be provided in the same format. A direct comparison across the C → Si → Ge series would allow a more systematic evaluation of the trends and further strengthen the comparative discussion.*

Response: We sincerely thank the reviewer for the constructive suggestion. In response, we have performed the corresponding calculations for the carbon- and silicon-based analogues. The calculated singlet (S_1) and triplet (T_1) energies, as well as the reorganization energies, of SiCzCz and SiTrzCz2 are nearly identical to those of GeCzCz and GeTrzCz2. In contrast, the carbon-based analogues, particularly CTrzCz2, exhibit

noticeable differences. Specifically, CTrzCz2 possesses lower calculated S_1 and T_1 energies of 3.39 and 2.67 eV, a lower oscillator strength (f = 0.0538), and a significantly higher reorganization energy of 1.03 eV compared to its silicon and germanium counterparts. We have updated Supplementary Fig. 12 to clearly illustrate these comparative trends.

Additional sentence, (Supplementary Information, Page S26): "The calculated singlet (S_1) and triplet (T_1) energies, as well as the reorganization energies, of SiCzCz and SiTrzCz2 are nearly identical to those of GeCzCz and GeTrzCz2. In contrast, the carbon-based analogues, particularly CTrzCz2, exhibit noticeable differences. Specifically, CTrzCz2 possesses lower calculated S_1 and T_1 energies of 3.39 and 2.67 eV, a lower oscillator strength (f = 0.0538), and a significantly higher reorganization energy of 1.03 eV compared to its silicon and germanium counterparts."

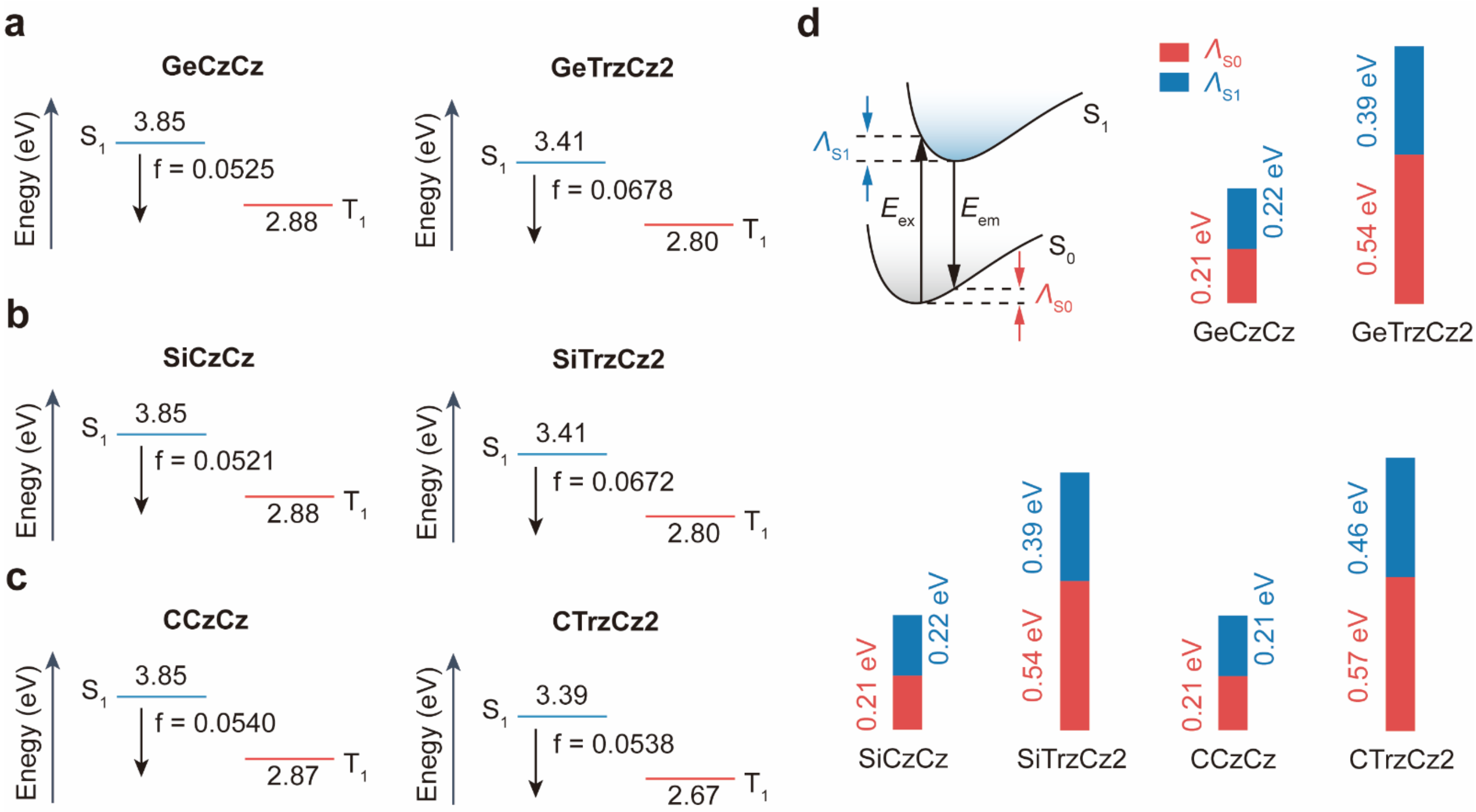

Supplementary Fig. 12. Theoretical calculations of the hosts. (**a**) Transition energies and oscillator strengths of GeCzCz and GeTrzCz2. (**b**) Transition energies and oscillator strengths of SiCzCz and SiTrzCz2. (**c**) Transition energies and oscillator strengths of CCzCz and CTrzCz2. (**d**) Reorganization energy.

Dear Reviewer,

Enclosed please find our revised manuscript entitled "**Germanium-integrated exciplex host for high-performance narrowband OLEDs with mitigated efficiency roll-off**" (Manuscript ID: **NCOMMS-25-25950C**) for publication as a research article in ***Nature Communications***. We sincerely thank the reviewer for helping us to improve our manuscript.

In response, we have meticulously revised the manuscript according to the Author checklist instruction and the point-by-point response to the reviewers' comments are provided. The major improvements upon revision include:

1. <u>We have prepared all figures and corresponding legends that comply with the requirement.</u>
2. <u>We have corrected the affiliation list by removing the previous second affiliation (College of Physical and Optoelectronic Engineering, Shenzhen University) to accurately reflect the institution where this research was exclusively conducted.</u>
3. <u>We have included a Funding statement in the manuscript as requested.</u>
4. <u>We have updated a Data Availability statement and prepared the source data files as requested.</u>

We hope that these revisions could satisfy the requirements for publication in ***Nature Communications***.

Yours Sincerely,

Chuluo Yang on behalf of the authors.

Corresponding author: **Professor Chuluo Yang**
at College of Materials Science and Engineering, Shenzhen University, Shenzhen 518060, People's Republic of China. Tel: +86-0755-86713985, E-mail: clyang@szu.edu.cn.

Point-by-Point Response to Reviewers' Comments

Reviewer #2:

General comment: *The authors have made a careful and substantial revision of the manuscript. The revised version has been significantly improved, and my previous concerns have been satisfactorily addressed. In particular, the clarified SOC calculation protocol, additional C/Si/Ge comparative and conformational analyses, corrected dipole-orientation measurements, optical simulations, and additional device/stability data have strengthened the mechanistic interpretation and internal consistency of the work. The claims are now appropriately moderated and sufficiently supported by the revised data. I have no further major concerns and recommend publication in Nature Communications.*

Response: We sincerely thank the reviewer for the highly positive feedback.
